# Global yellow fever vaccination coverage from 1970 to 2016: an adjusted retrospective analysis

**DOI:** 10.1016/S1473-3099(17)30419-X

**Published:** 2017-11

**Authors:** Freya M Shearer, Catherine L Moyes, David M Pigott, Oliver J Brady, Fatima Marinho, Aniruddha Deshpande, Joshua Longbottom, Annie J Browne, Moritz U G Kraemer, Kathleen M O'Reilly, Joachim Hombach, Sergio Yactayo, Valdelaine E M de Araújo, Aglaêr A da Nóbrega, Jonathan F Mosser, Jeffrey D Stanaway, Stephen S Lim, Simon I Hay, Nick Golding, Robert C Reiner

**Affiliations:** a Big Data Institute, Li Ka Shing Centre for Health Information and Discovery, University of Oxford, Oxford, UK; b Department of Zoology, University of Oxford, Oxford, UK; c Institute for Health Metrics and Evaluation, University of Washington, Seattle, WA, USA; d Division of Pediatric Infectious Diseases, Seattle Children's Hospital, University of Washington, Seattle, WA, USA; e Department of Infectious Disease Epidemiology, London School of Hygiene & Tropical Medicine, London, UK; f Department of Disease Control, London School of Hygiene & Tropical Medicine, London, UK; g University of State of Rio de Janeiro, Maracana, Rio de Janeiro, Brazil; h Harvard Medical School, Boston, MA, USA; i Boston Children's Hospital, Boston, MA, USA; j Initiative for Vaccine Research, Immunization, Vaccines and Biologicals, World Health Organization, Geneva, Switzerland; k Infectious Hazard Management, World Health Organization, Geneva, Switzerland; l Secretariat of Health Surveillance of the Ministry of Health of Brazil, Rio de Janeiro, Brazil; m Quantitative & Applied Ecology Group, School of BioSciences, University of Melbourne, Parkville, VIC, Australia

## Abstract

**Background:**

Substantial outbreaks of yellow fever in Angola and Brazil in the past 2 years, combined with global shortages in vaccine stockpiles, highlight a pressing need to assess present control strategies. The aims of this study were to estimate global yellow fever vaccination coverage from 1970 through to 2016 at high spatial resolution and to calculate the number of individuals still requiring vaccination to reach population coverage thresholds for outbreak prevention.

**Methods:**

For this adjusted retrospective analysis, we compiled data from a range of sources (eg, WHO reports and health-service-provider registeries) reporting on yellow fever vaccination activities between May 1, 1939, and Oct 29, 2016. To account for uncertainty in how vaccine campaigns were targeted, we calculated three population coverage values to encompass alternative scenarios. We combined these data with demographic information and tracked vaccination coverage through time to estimate the proportion of the population who had ever received a yellow fever vaccine for each second level administrative division across countries at risk of yellow fever virus transmission from 1970 to 2016.

**Findings:**

Overall, substantial increases in vaccine coverage have occurred since 1970, but notable gaps still exist in contemporary coverage within yellow fever risk zones. We estimate that between 393·7 million and 472·9 million people still require vaccination in areas at risk of yellow fever virus transmission to achieve the 80% population coverage threshold recommended by WHO; this represents between 43% and 52% of the population within yellow fever risk zones, compared with between 66% and 76% of the population who would have required vaccination in 1970.

**Interpretation:**

Our results highlight important gaps in yellow fever vaccination coverage, can contribute to improved quantification of outbreak risk, and help to guide planning of future vaccination efforts and emergency stockpiling.

**Funding:**

The Rhodes Trust, Bill & Melinda Gates Foundation, the Wellcome Trust, the National Library of Medicine of the National Institutes of Health, the European Union's Horizon 2020 research and innovation programme.

## Introduction

Substantial outbreaks of yellow fever in the past 2 years in Angola and Brazil, highlight a pressing need to assess present control efforts.^1^ Yellow fever is an acute viral haemorrhagic disease that is vaccine preventable, yet it is widely distributed in the tropics of Latin America and Africa where infections cause an estimated 29 000 to 60 000 deaths annually.^2^ Yellow fever virus is transmitted to human beings through the bites of infected mosquitoes (primarily in the genus *Aedes*), and is principally maintained by a sylvatic (jungle) transmission cycle involving non-human primate reservoirs. Urban yellow fever outbreaks occur when infected people introduce the virus into heavily populated areas with competent vector populations and insufficient vaccination coverage. The spectrum of human clinical disease caused by the virus is broad, ranging from asymptomatic infections and mild febrile illness to severe disease and death. A heavy emphasis is put on disease control to prevent infections because no specific antiviral drug exists for yellow fever.^3^

If control is insufficient, yellow fever virus can cause devastating epidemics, especially in populations where vaccine-derived or naturally acquired immunity is low, with high case-fatality rates.^4^ Although elimination of yellow fever is not feasible due to its sylvatic reservoir, control is achievable due to the availability of a safe, low-cost, and highly effective vaccine. The yellow fever vaccine is a live attenuated vaccine that rapidly stimulates immunity (within 30 days for 99% of people vaccinated) and can provide lifelong protection.^3^ Since the vaccine became available in 1937, multiple vaccination strategies have been implemented, including the introduction of the vaccine into routine childhood schedules, mass preventive and reactive campaigns, and the vaccination of travellers to yellow fever risk zones. Combined with vector control programmes, vaccination has led to a notable reduction in disease burden at targeted locations and times.^2^, ^4^ Yet in many yellow fever risk areas, vaccine coverage remains too low to prevent outbreaks. WHO recommends population vaccination coverage of 80% or more to prevent and control outbreaks.^5^

**Research in context**

**Evidence before this study**

We searched PubMed on May 24, 2017, using the search terms “yellow fever”[All Fields] AND (“vaccine coverage”[All Fields] OR “vaccination coverage”[All Fields] OR “immunization coverage”[All Fields] OR “immunisation coverage”[All Fields]), without any date or language restrictions. This search returned 65 articles, of which 12 contained spatial information on yellow fever vaccination coverage. Eight of these articles reported coverage estimates derived from single surveys done for individual nations at a particular timepoint. Two articles presented descriptive spatial analyses of vaccination coverage data and another analysed the cost-effectiveness of introducing routine yellow fever vaccination, which included simulated estimates of future coverage. Garske and colleagues estimated population-wide coverage across all at-risk countries on the African continent through the decades from 1960 to 2010, but not, however, for Latin America. In 2017, WHO published estimates of the number of vaccine doses required for each region to support the Global Strategy to Eliminate Yellow Fever Epidemics, 2017–2026.

**Added value of this study**

The aims of this study were to generate yellow fever vaccination coverage maps for both Africa and Latin America from 1970 to 2016, and to estimate the additional coverage needed to prevent further outbreaks. We used a similar approach to the previously published map for Africa, but importantly for this study, we assessed the range of estimates produced when alternative vaccination-targeting scenarios are considered. We also included more recent vaccination coverage data and extended the map to Latin America. Overall our study highlights the stark differences in vaccination coverage between Latin America and Africa. Our estimates of the number of individuals who still require vaccination to achieve target thresholds for the prevention of outbreaks are provided at district level, and these numbers can be recalculated with our coverage estimates and any new threshold.

**Implications of all the available evidence**

Our coverage map for 2016 highlights key gaps in present levels of vaccination coverage within yellow fever risk zones and enables the identification of areas that currently do not meet the WHO recommendation of 80% population coverage, and are therefore at risk of an outbreak. Our regional and global estimates of the number of individuals requiring vaccination to prevent future outbreaks corroborate WHO estimates, and our district estimates can assist in the planning of vaccine delivery, emergency stockpiling, and manufacturing surge capacity.

The outbreak that started in Angola in December, 2015, developed into the largest and most widespread outbreak of yellow fever reported in Africa in more than 20 years, with 884 confirmed cases in Angola and 78 in neighbouring Democratic Republic of the Congo.^6^, ^7^ Before 2015, yellow fever had not troubled Angola since 1988 when a much smaller outbreak resulted in 37 cases.^8^ Angola introduced the vaccine into routine infant immunisation programmes in 1999,^9^ but there had been no campaigns to protect adults born before 1999 since an outbreak response campaign in Luanda in 1988.^8^

The Angolan outbreak also exposed vulnerabilities in the preparedness of local and international agencies to respond to such emergencies. Efforts to control the outbreak through mass reactive vaccination campaigns were hampered by dwindling international vaccine supplies and other operational challenges.^10^ With the global yellow fever vaccine emergency stockpile depleted by mid-2016, WHO approved the temporary use of fractional doses to stretch remaining stocks.^11^

The course of the Angolan outbreak and international response reiterates the need for a sustained policy of preventive vaccination of at-risk populations to reduce the risk of epidemics. Vaccination campaigns and other control strategies require improved understanding of present vaccination coverage rates that have resulted from cumulative campaigns, within the two risk zones. Spatial estimates of vaccination coverage for yellow fever have been produced previously^2^, ^12^ but are often restricted to specific age cohorts or countries, with one study^2^ estimating population-wide coverage across all at-risk countries in Africa, but not for Latin America. The aims of this study were to generate yellow fever vaccination coverage maps for both Africa and Latin America from 1970 to 2016, and to estimate the additional coverage needed to prevent further outbreaks.

## Methods

### Data collation

Yellow fever vaccination is primarily delivered through three programme types: routine childhood immunisation targeting infants aged around 9 months in at-risk regions; periodically conducted mass preventive and outbreak response campaigns that target a broader age range; and vaccination of people travelling to high-risk areas. To track vaccination coverage of the entire population over time for this adjusted retrospective analysis, we compiled a dataset of the coverage level achieved by each of the first two classes of vaccination activity for each age group at specific locations and time periods from the earliest use of the vaccine in 1939 to 2016. Data on routine infant vaccination coverage were obtained from annual national estimates, based on health-service-provider registries of yellow fever vaccination coverage reported yearly by each at-risk country to WHO and UNICEF.^13^, ^14^ Data on mass preventive and outbreak response campaigns were primarily extracted from online sources, including the Weekly Epidemiological Records,^15^ Disease Outbreak News,^16^ Morbidity and Mortality Weekly Reports,^17^ and two WHO reports: one on mass vaccination in west and central Africa through the 1940s to 1960s,^18^ and another on the 2016 epidemic in the Democratic Republic of the Congo.^19^ The Brazilian national immunisation programme information system also provided more detailed data for Brazil from 2006 to 2015. Descriptions of each vaccination and demographic data type, along with details on data processing and collation, are provided in the appendix (pp 1–9). Table 1 shows a summary of datasets obtained via the websites of WHO,^13^, ^14^, ^15^, ^16^ United Nations World Population Prospects,^20^, ^21^ and the US Centers for Disease Control and Prevention.^17^

**Table 1:** Summary of online data sources

	**Years searched/available**	**Date accessed**	**Data extracted**
WHO Weekly Epidemiological Records^15^	1970–2016	Aug 26, 2016	Mass vaccination
WHO Disease Outbreak News^16^	1996–2016	Aug 26, 2016	Mass vaccination
CDC Morbidity and Mortality Weekly Reports^17^	1982–2016	Oct 3, 3016	Mass vaccination
WHO-UNICEF reviews of national immunisation coverage^14^	1980–2014	Aug 26, 2016	Routine vaccination
Country-level estimates of routine infant immunisation coverage from WHO-UNICEF Joint Reporting Form for yellow fever virus and DTP3^13^	1980–2015	Oct 3, 2016	Routine vaccination
UNWPP annual population by 5-year age groups^20^	1950–2015	Oct 13, 2016	Demographic
UNWPP infant mortality rate^21^	1950–2015	Oct 10, 2016	Demographic
UNWPP under-5 mortality^21^	1950–2015	Oct 10, 2016	Demographic

Vaccination activity and demographic data accessed via WHO, UNWPP, and CDC websites. Mass vaccination was regarded as preventive and outbreak response vaccination activities. CDC=Centers for Disease Control and Prevention. DTP3=third dose of diphtheria-tetanus-pertussis-containing vaccine. UNWPP=United Nations World Population Prospects.

### Application of correction terms to non-survey vaccination data

Where available, vaccination data from post-campaign coverage surveys were used in preference to administratively reported coverage data (ie, health service provider reported estimates) because these generally provide more reliable coverage estimates. Additionally, all administratively reported coverage data were multiplied by bias correction terms to adjust for variability in health system reporting reliability. These data included all routine vaccination data and any preventive or outbreak response data for which the percentage of the target population vaccinated was not estimated from a post-campaign coverage survey (only six campaigns reported results of a post-campaign survey). We calculated bias correction terms for each country and year as ratios of survey-derived Global Burden of Disease Study^22^ estimates of mean coverage for third dose of diphtheria-tetanus-pertussis-containing vaccine (DTP3) and WHO/UNICEF Joint Reporting Process administratively reported estimates.^13^, ^23^ Coverage for DTP is often used as the main indicator for performance of routine vaccination services.^24^ Data were available to calculate bias correction terms for each country from 1980 to 2015. For outbreak response campaigns done before 1980, bias corrections terms for 1980 were applied, and for 2016, correction terms for 2015 were used. WHO also corrects reported administrative data,^25^ but the DTP terms were more comprehensive in time and space. The bias correction terms for each country and year are available in Dryad.

### Estimation of vaccination coverage

Using the age, time, and location specific vaccination coverage and population datasets, we tracked each age cohort (from ages 0 to 99 years) in every district through time—from their birth year through to 2016 (ie, the earliest cohort was born in 1871 to track coverage of individuals aged 99 years in 1970)—updating the coverage level whenever a routine, preventive, or outbreak response campaign was conducted. We assumed that the mortality rate for vaccinated and unvaccinated individuals was the same and that no mixing of populations between districts occurred.

Because the strategies for within-population targeting of vaccinations were unknown, we generated three alternative vaccination coverage estimates, each corresponding to one of three targeting scenarios. (1) Untargeted, unbiased: assuming vaccination history was not taken into account and all individuals had an equal chance of receiving a vaccine irrespective of their previous vaccination status. (2) Targeted: assuming that vaccination history was taken into account and only non-vaccinated individuals were targeted by immunisation campaigns. (3) Untargeted, biased: assuming that vaccination history was not taken into account and that previously vaccinated individuals were more likely to be targeted inadvertently (ie, because of demographic biases in vaccination uptake). In the untargeted, biased scenario, for each vaccination campaign, we assumed that all previously vaccinated individuals received vaccines before any unvaccinated individuals. This scenario produces maximally conservative estimates of vaccination coverage, whereas estimates from the targeted scenario are maximally optimistic. Further detail on vaccination coverage calculations is provided in the appendix (p 9).

### Estimation of the number of people requiring vaccination in 2016 to meet targets

Using each of the three sets of values of vaccination coverage for 2016 based on optimistic through to conservative scenarios of historical targeting, we calculated three sets of estimates for the number of individuals across all ages who still require vaccination to reach the 80% population coverage threshold recommended by WHO to prevent outbreaks^5^ for each at-risk district (districts classified by the WHO range maps for yellow fever as either completely or partially endemic or as having low potential for exposure^26^). That is, the number of people who would require supplementary vaccination under the assumption that, in the future, unvaccinated individuals will be targeted (ie, the most optimistic scenario) and that the 80% coverage threshold applies across all populations. The resulting figures were then summed to obtain national, regional, and global estimates. The number of individuals still requiring vaccination for each at-risk district is provided in Dryad. We also calculated a global estimate of the number of people who would have required vaccination in 1970 for comparison.

### Role of the funding source

The funders of the study had no role in study design, data collection, data analysis, data interpretation, or writing of the report. The corresponding author had full access to all the data in the study and had final responsibility for the decision to submit for publication.

## Results

**Figure 1:**
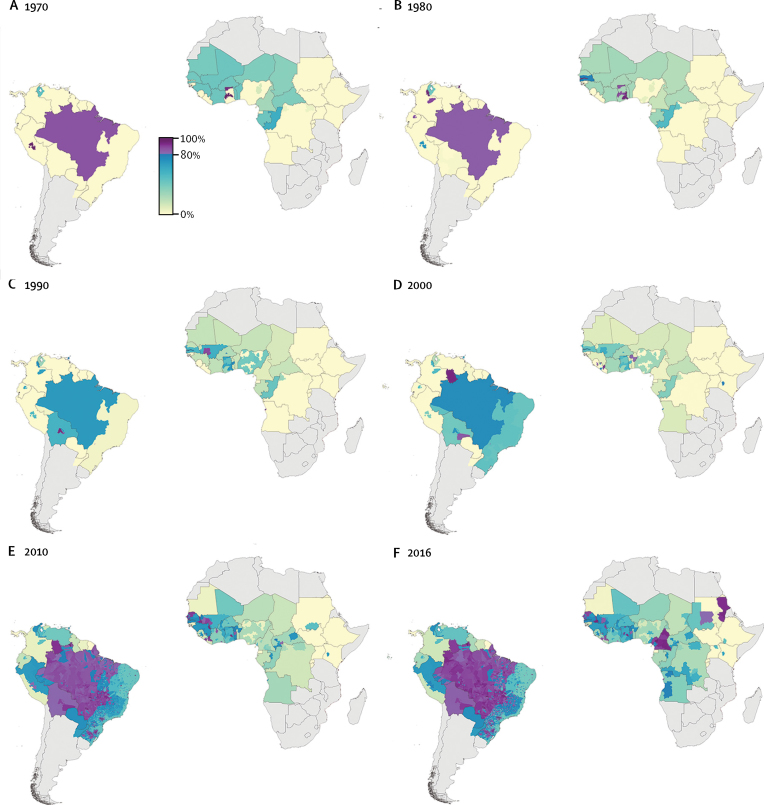
Yellow fever vaccination coverage from 1970 to 2016 Estimated proportion of the population across all age cohorts who have ever received a yellow fever vaccine at the beginning of each decade (and in 2016) at the second administrative level for countries at risk of yellow fever virus transmission, based on the untargeted, unbiased vaccination-targeting scenario.

Our estimates of yellow fever vaccination coverage, based on the untargeted, unbiased campaigns scenario, highlight substantial international and subnational variation, both spatially and temporally (figure 1). Population vaccination coverage rates in countries within risk zones ranged from a maximum of 100% in parts of Amazonas State, Brazil, to zero coverage in parts of central and east Africa (routine infant immunisation programmes have not yet been introduced in these areas). Throughout the decades, vaccination coverage rates were higher overall in Latin America compared with Africa. Coverage rates were particularly high in Brazil during the 1970s and 1980s, declined slightly in the 1990s, and were again very high in most parts of the country by 2016. A similar pattern of waxing and waning was seen for many countries in west and central Africa. Vaccination coverage rates were moderately high throughout these two regions in the 1970s, but coverage declined between 1970 and 2000, before reaching high rates again by 2016. However, several localised areas did achieve high coverage rates at various timepoints, including parts of Senegal, The Gambia, Ghana, and Mali.

When we considered contemporary coverage (ie, the 2016 coverage map; figure 1), important gaps were apparent within the risk zones of Africa, including large areas of central and east Africa and parts of Nigeria, Niger, Sierra Leone, Liberia, and Guinea-Bissau. In Latin America, low coverage was estimated for Guyana, Suriname, French Guiana, and Colombia. Vaccination coverage estimates for all at-risk districts for each year presented in figure 1 are provided in Dryad.

**Figure 2:**
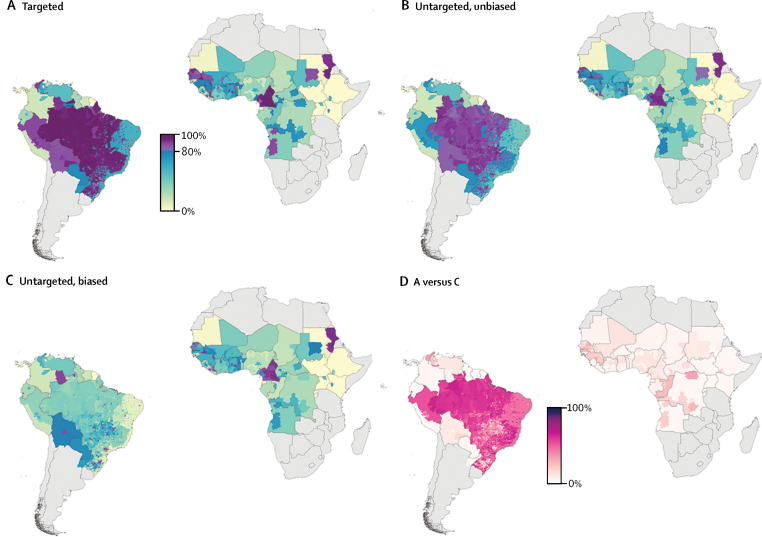
Contemporary yellow fever vaccination coverage scenarios Estimated proportion of the population in 2016 across all age cohorts who have ever received a yellow fever vaccine at the second administrative level in countries at risk of yellow fever virus transmission. Vaccination coverage was calculated using three alternative vaccination-targeting scenarios: (A) targeted; (B) untargeted, unbiased; and (C) untargeted, biased. (D) Percentage difference in coverage between targeting scenarios A and C.

We found substantial geographical variation in the sensitivity of estimated vaccination coverage rates to alternative assumptions for vaccination-targeting, which shows the potential effect of an important area of uncertainty in vaccination data. The percentage differences (figure 2) in coverage between the targeted versus the untargeted, biased alternative vaccination-targeting scenarios range from 0% difference in most of central and east Africa and most countries in Latin America, to up to 73% in a small number of municipalities within Brazil.

**Figure 3:**
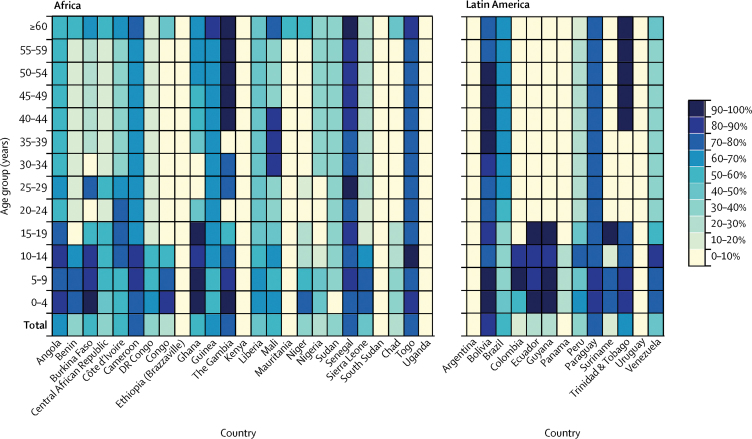
National yellow fever vaccination coverage by age group Estimated proportion of individuals within each age group in 2016 who have ever received a yellow fever vaccine for each country at risk of yellow fever virus transmission, based on the untargeted, unbiased vaccination-targeting scenario. Plots for the beginning of each decade from 1970 to 2010 are provided in the appendix (pp 10–11).

Country-level estimates of yellow fever vaccination coverage by age group in 2016 highlight the progress of routine infant immunisation programmes in protecting children and young adults on both continents (figure 3), but they also revealed coverage gaps in adult populations for most countries. Angola, Cameroon, Guinea, Senegal, Togo, Paraguay, Bolivia, and Brazil were exceptions to this, with moderate to high coverage estimated across all age groups. Estimates by age group from 1970 to 2010 are provided in the appendix (pp 10–11).

From the 2016 outputs, we estimated that between 393·7 million and 472·9 million individuals still require vaccination within at-risk districts globally, to achieve the 80% population coverage threshold recommended by WHO to prevent outbreaks (table 2). Between 361·4 and 396·0 million of these people live in Africa and between 32·2 and 76·9 million live in Latin America. The national estimates of the total number of individuals requiring vaccination within each country are provided in table 2 and disaggregated values for each district are available in Dryad. All countries except Bolivia, Peru, and Senegal contained at-risk districts with less than 80% population coverage under all three historical vaccination-targeting scenarios.

**Table 2:** Number of individuals in millions still requiring vaccination in 2016

	Only at-risk districts*			**All districts**†		
	Conservative	Untargeted	Optimistic	Conservative	Untargeted	Optimistic
Nigeria	112·0	101·2	95·9	112·0	101·2	95·9
Ethiopia	74·4	74·3	74·2	80·9	80·8	80·8
Kenya	32·4	32·4	32·4	37·6	37·6	37·6
Uganda	32·2	32·2	32·2	32·4	32·4	32·4
Democratic Republic of the Congo	30·4	28·6	26·9	34·9	32·5	30·3
Sudan	12·9	12·5	12·1	19·5	19·0	18·7
Rwanda	10·2	10·2	10·2	10·2	10·2	10·2
South Sudan	9·8	9·8	9·8	9·8	9·8	9·8
Niger	9·8	9·6	9·5	9·8	9·6	9·5
Burundi	8·9	8·9	8·9	8·9	8·9	8·9
Chad	8·4	8·2	8·0	8·5	8·3	8·1
Ghana	6·7	5·9	5·4	6·7	5·9	5·4
Côte d'Ivoire	6.4	5.9	5.5	6.4	5.9	5.5
Benin	4·8	4·6	4·4	4·8	4·6	4·4
Burkina Faso	4·3	3·9	3·6	4·3	3·9	3·6
Mali	4·7	3·7	3·2	4·7	3·7	3·2
Angola	4·6	3·5	3·3	4·6	3·5	3·3
Cameroon	3.3	3.0	2.8	3.3	3.0	2.8
Sierra Leone	2·5	2·5	2·5	2·5	2·5	2·5
Congo (Brazzaville)	3·0	2·2	1·7	3·0	2·2	1·7
Central African Republic	2·3	2·2	2·1	2·3	2·2	2·1
Mauritania	2·1	2·0	2·0	3·3	3·2	3·2
Guinea	2·9	1·6	0·2	2·9	1·6	0·2
Liberia	1·6	1·6	1·6	1·6	1·6	1·6
Guinea-Bissau	1·1	1·1	1·1	1·1	1·1	1·1
Gabon	1·0	1·0	1·0	1·0	1·0	1·0
Senegal	2·2	0·7	0·0	2·2	0·7	0·0
Equatorial Guinea	0·7	0·7	0·7	0·7	0·7	0·7
Togo	0·4	0·3	0·2	0·4	0·3	0·2
The Gambia	0·1	0·1	0·1	0·1	0·1	0·1
Tanzania	··	··	··	41.7	41·7	41·7
Eritrea	··	··	··	5·3	5·3	5·3
Somalia	··	··	··	4·7	4·7	4·7
Zambia	··	··	··	1·5	1·5	1·5
São Tomé and Príncipe	··	··	··	0·1	0·1	0·1
Totals for Africa	396·0	373·9	361·4	473·4	450·8	437·8
Colombia	23·5	23·0	22·7	29·9	29·3	29·0
Brazil	40.3	6.8	2.1	121.8	49.7	27.1
Venezuela	6·2	4·7	3·9	12·6	10·3	9·1
Panama	1·0	1·0	1·0	1·0	1·0	1·0
Peru	3·1	0·8	0·0	18·7	15·3	14·1
Ecuador	0·6	0·6	0·6	8·7	8·7	8·7
Paraguay	0·5	0·5	0·4	0·5	0·5	0·4
Guyana	0·4	0·4	0·4	0·4	0·4	0·4
Suriname	0·4	0·4	0·4	0·4	0·4	0·4
Argentina	0·2	0·2	0·2	1·3	1·3	1·3
Trinidad and Tobago	0·2	0·2	0·2	0·2	0·2	0·2
French Guiana	0·2	0·2	0·2	0·2	0·2	0·2
Bolivia	0·1	0·0	0·0	0·2	0·0	0·0
Totals for Latin America	76·9	38·8	32·2	196·1	117·4	92·1
Global totals	472·9	412·8	393·7	669·5	568·2	529·9

Estimated number of individuals (in millions) that still need to be vaccinated against yellow fever in 2016 to achieve the population coverage threshold of 80% recommended by WHO to prevent outbreaks, in every district or municipality in each country of Africa and Latin America that is classified as either completely or partially at risk for yellow fever. Separate estimates are given on the basis of the three alternative targeting scenarios for historical campaigns. Countries are ordered from greatest number needed to vaccinate to smallest across at-risk districts, for each continent. Estimates for each district or municipality are available in Dryad.

* Estimated number of individuals requiring vaccination based on districts within areas classified by WHO range map as endemic or transitional.^26^

† Estimated number of individuals requiring vaccination based on all districts in countries partially or completely classified as endemic or transitional by the WHO range map in addition to districts classified as having low potential for exposure to yellow fever virus, defined as areas where yellow fever has not been reported (but evidence of viral transmission in the past might exist), areas bordering endemic zones, or areas where yellow fever vectors and non-human primate hosts are present.^2^

## Discussion

We mapped population-wide vaccination coverage for yellow fever across all countries within risk zones from 1970 to 2016. Present vaccination coverage is the result of cumulative campaigns, therefore it is important to incorporate information on past vaccination strategies to understand the present position. Our coverage map for 2016 highlights key gaps in present levels of vaccination coverage within yellow fever risk zones and enables the identification of areas that currently do not meet the WHO recommendation of 80% population coverage, and are therefore at risk of an uncontrolled outbreak.

Our conservative estimates of the number of people requiring vaccination in 2016 to meet the 80% threshold are approximately 10% smaller for Africa and 3% greater for Latin America (table 2) than the WHO's estimates of doses required for mass campaigns as part of the Global Strategy to Eliminate Yellow Fever Epidemics, 2017–2026.^5^ Our district estimates can assist in the planning of vaccine delivery, and if combined with estimates of the number of doses required for future routine infant immunisation programmes, they can be used in the planning of emergency stockpiling and manufacturing surge capacity. Notably, the 80% threshold is an average; in reality, the critical vaccination coverage needed to prevent outbreaks is expected to vary substantially across different settings depending tightly on the basic reproduction number, R0.^27^ Using our estimates of the proportion vaccinated for each district, others can recalculate the number of individuals requiring vaccination to reach any new threshold.

The estimates of vaccination coverage over time show the cumulative effect of multiple different vaccination strategies combined with the different demographic profiles of the two at-risk regions. Our results for Africa from 1970 to 2010 corroborate the analysis by Garske and colleagues.^2^ We used a similar approach but, importantly for this study, we assessed the range of estimates produced when alternative vaccination-targeting scenarios are considered. We also included more recent vaccination coverage data and extended the map to Latin America. Overall our study highlights the stark differences between Latin America and Africa, which might, in part, account for the difference in the observed number of outbreaks between these two regions.^4^

Combined with vector control efforts, high levels of vaccination coverage in Brazil have led to a substantial reduction in the number of yellow fever cases since the 1930s, especially cases arising from urban transmission cycles. Coverage rates were particularly high in Brazil during the 1970s and 1980s, but then declined slightly, resulting in a resurgence of sylvatic transmission in the 1990s and early 2000s.^4^ This resurgence prompted further mass vaccination campaigns and the resulting 2016 vaccination coverage rates were estimated to be very high for most parts of the country. We estimated lower levels of vaccination coverage at the eastern edge of the risk zone in Brazil for 2016, including within the state of Minas Gerais, where a yellow fever outbreak arose in December, 2016.^1^

The moderately high vaccination coverage rates throughout much of west and central Africa in the 1970s (figure 1) were the result of mass preventive campaigns through the 1940s to 1960s,^18^ which reduced the number of outbreaks. Coverage waned between 1960 and 2000 in most areas due to limited vaccination activity, the birth of new unvaccinated cohorts, and the gradual reduction in the proportion of older protected cohorts through mortality. Vaccine stock shortages, which have been frequently reported in the African region,^5^ would have also contributed to lower than recommended coverage levels. Several localised areas, including parts of Senegal, The Gambia, Ghana, and Mali, were exceptions to this pattern because they conducted outbreak response campaigns that reached high levels of coverage. In 2010, vaccination coverage in Angola was estimated to be low across all districts, except Luanda with 64% coverage. Following outbreak response campaigns in 2016, coverage had reached nearly 90% for Luanda, and just under the 80% threshold across a number of other districts in the north and west of the country. Additionally, the most recent Disease Outbreak News report^28^ included in this study indicated that vaccination campaigns were ongoing in several of these districts.

Many countries in west and central Africa introduced the yellow fever vaccine into their routine infant immunisation schedules between the late 1980s and early 2000s,^9^ but the effect of these efforts will take time to affect population level coverage in the absence of campaigns targeting adults born before the late 1980s (figure 3). For adequate protection against outbreaks, the vaccine should be introduced into routine infant immunisation schedules, followed by catch-up campaigns for adult populations. The success of this approach is exemplified by The Gambia, which reported no locally acquired cases for more than two decades when in 1979 they conducted a post-outbreak mass vaccination campaign targeting all ages and in tandem introduced the vaccine into their routine infant immunisation schedule.^9^ Increased coverage rates were achieved overall in west and central Africa in 2010 and 2016 after large-scale preventive campaigns supported by the Yellow Fever Initiative and Gavi, the Vaccine Alliance, were implemented in 2006.^29^

Our dataset of vaccination coverage, compiled from a range of sources, highlights concerns about the completeness and accuracy of the reports on vaccination activity. To assess the effect of uncertainty regarding whether vaccination history was taken into account when targeting individuals in mass campaigns, we calculated vaccination coverage using three different vaccination coverage scenarios ranging from conservative to optimistic. Brazil was most sensitive to different assumptions about the vaccination-targeting scenarios (figure 2) because they have conducted sustained vaccination activities since the 1940s; when vaccination coverage in each age cohort was tracked through time, there was ample opportunity for alternative estimates to diverge from one another. Additionally, the vaccination activity data for Brazil used in our analysis were more complete, and spatially and temporally disaggregated, than they were for other countries. Lessler and colleagues^30^ describe an approach for estimating uncertainties associated with vaccination targeting by using administrative data and cross-sectional survey data, across a range of ages, on measles vaccination to calculate the size of the population systematically missed by vaccination activities in a particular country. However, insufficient cross-sectional survey data on yellow fever, needed for this method, are available, especially for adult populations.

Estimates of vaccination coverage at the country or province level, including most of the routine infant vaccination data and much of the preventive and reactive mass vaccination campaigns used in this study, often smooth out important sources of spatial heterogeneity. Characterisation of such heterogeneities is important for planning spatially targeted interventions. High-resolution vaccination coverage data, such as that used in this Article for Brazil, would improve certainty of coverage estimates for other countries. Data from household surveys would afford increased certainty and resolution of vaccination coverage estimates, but are only available for limited times, locations, and age cohorts, and as such require geospatial modelling to interpolate across time and space.

Our maps of vaccination coverage do not necessarily translate into an absolute measure of vaccine-derived immunity because vaccine efficacy is not 100% (although evidence suggests that yellow fever vaccine efficacy is close to 100% and WHO concluded that a single dose gives lifelong protection).^3^ Likewise, the proportion of individuals with immunity to yellow fever virus is not necessarily equivalent to the proportion of individuals with vaccine-derived immunity; this is due to the possible acquisition of immunity via natural infection, especially where outbreaks have occurred. Our coverage estimates could be combined with estimates of vaccine efficacy, and of natural infection rates to estimate the number of individuals susceptible to symptomatic yellow fever infection and to quantify the effect of vaccination on yellow fever incidence and outbreak potential.

Yellow fever cannot be eliminated due to the presence of non-human wildlife reservoirs, which maintain the sylvatic transmission cycle of the virus in non-urban settings, but the risk of a yellow fever epidemic can be eliminated if effective vector control, vaccination, and disease surveillance are enforced and maintained. The results of this study highlight both important progress and gaps in yellow fever vaccination coverage within risk zones and provide credible estimates of the doses required for supplementary campaigns. This information should be coupled with fine-scale estimates of geographical disease risk to identify populations most susceptible to yellow fever infection. Only then can policy makers begin to develop the most effective vaccination strategies to prevent outbreaks.
