# Supplementary material for: Global yellow fever vaccination coverage from 1970 to 2016: an adjusted retrospective analysis
Source: Lancet Infect Dis. 2017 Nov;17(11):1209–17. doi: 10.1016/S1473-3099(17)30419-X (PMC5666204; doi:10.1016/S1473-3099(17)30419-X)
Supplement: Supplementary appendix [file mmc1.pdf]

THE LANCET Infectious Diseases

Supplementary webappendix

This webappendix formed part of the original submission and has been peer reviewed. We post it as supplied by the authors.

Supplement to: Shearer FM, Moyes CL, Pigott DM, et al. Global yellow fever vaccination coverage from 1970 to 2016: an adjusted retrospective analysis. *Lancet Infect Dis* 2017; published online August 16. http://dx.doi.org/10.1016/S1473-3099(17)30419-X.

Appendices to "Estimating global yellow fever vaccination coverage from 1970 to 2016: an empirical analysis tracking vaccination in all age cohorts"

Freya M Shearer[1*], Catherine L Moyes[1], David M Pigott[2], Oliver J Brady[3], Fatima Marinho[4], Aniruddha Deshpande[2], Joshua Longbottom[1], Annie J Browne[1], Moritz UG Kraemer[5,6,7], Kathleen M O'Reilly[8], Joachim Hombach[9], Sergio Yactayo[10], Valdelaine EM de Araújo[11], Algaêr A da Nóbrega[11], Jonathan F Mosser[2,12], Jeffrey D Stanaway[2], Stephen S Lim[2], Simon I Hay[1,2], Nick Golding[13], Robert C Reiner Jr[2]

[1]Big Data Institute, Li Ka Shing Centre for Health Information and Discovery, University of Oxford, Oxford OX3 7LF, United Kingdom
[2]Institute for Health Metrics and Evaluation, University of Washington, 2301 5th Avenue, Suite 600, Seattle, WA, 98121, United States of America
[3]Department of Infectious Disease Epidemiology, London School of Hygiene and Tropical Medicine, London, WC1E 7HT, United Kingdom
[4] University of State of Rio de Janeiro, Maracana, Rio de Janeiro 20550-900, Brazil
[5]Department of Zoology, University of Oxford, New Radcliffe House, Radcliffe Observatory Quarter, 6GG, Woodstock Rd, Oxford OX2, United Kingdom
[6]Harvard Medical School, 25 Shattuck St, Boston, MA 02115, United States of America
[7]Boston Children's Hospital, 300 Longwood Ave, Boston, MA 02115, United States of America
[8]Department of Disease Control, London School of Hygiene and Tropical Medicine, London WC1E 7HT, United Kingdom
[9]World Health Organization, Initiative for Vaccine Research (IVR), Immunization, Vaccines and Biologicals (IVB), 20 Av Appia, 1211 Geneva 27. Switzerland
[10]World Health Organization, Infectious Hazard Management (IHM), 20 Av Appia, 1211 Geneva 27. Switzerland
[11]Secretariat of Health Surveillance of the Ministry of Health of Brazil, SRTVN 701, Via W5 Norte, Ed. PO700, 6º andar, CEP 70723-040, Brazil
[12]Division of Pediatric Infectious Diseases, Seattle Children's Hospital/University of Washington, PO Box 5371, Seattle, WA 981452, United States of America
[13]Quantitative & Applied Ecology Group, School of BioSciences, University of Melbourne, Parkville, VIC 3010, Australia

*Corresponding author:
Freya M Shearer
Email: freya.m.shearer@gmail.com
Phone: +447 575 335 558

Methods appendix

Data collation and extraction

Routine childhood vaccination data

Data on routine infant vaccination coverage were obtained from annual national estimates, based on health-service-provider registries of yellow fever (YF) coverage reported yearly by each at-risk country to the World Health Organization (WHO) and United Nations International Children's Emergency Fund (UNICEF) using the joint reporting form (JRF) on immunisation.[1] The data were available from the year of YF vaccine introduction into the country's routine immunisation schedule up to 2015. Province or state level data, as estimated by the WHO/UNICEF[2] based on data reported in the JRF, were also available for certain countries with localised areas of YF risk, namely, Panama, Argentina, and Kenya.[3] The WHO/UNICEF estimates for Brazil indicated that vaccines were only administered to infants in risk zones and we assumed these estimates only applied to municipalities within areas classified as 'endemic' by WHO range maps.[4] Since we used other data types reporting up to 2016, to obtain estimates of all routine coverage for 2016, we assumed that coverage was the same as for 2015.

Preventive and outbreak response vaccination campaign data

Data on mass preventive and outbreak response campaigns were primarily extracted from published online sources including the Weekly Epidemiological Records (WERs), Disease Outbreak News (DONs), Morbidity

and Mortality Weekly Reports (MMWRs), and two WHO reports: one on mass vaccination in West and Central Africa through the 1940s to 1960s,[5] and another on the 2016 epidemic in the Democratic Republic of Congo[6]. For the WERs, DONs, and MMWRs, we searched all reports within the specified date range (see manuscript Table 1) for the keywords "yellow fever", of which we downloaded those containing information on YF vaccination activities. The detail in these reports varied greatly from campaign to campaign and was reported at a variety of spatial scales. If vaccination coverage for a campaign was not given, it was estimated using other information, such as the number of vaccine doses administered, combined with population estimates (see 'Processing demographic data', below). The following information was recorded for each vaccination campaign, where available: campaign time period, target location(s), target age range, number of vaccine doses (either administered or supplied), target population size, proportion of target population vaccinated (coverage), and whether coverage was estimated from a post-campaign survey or administrative data (ie, health-service-provider reported estimates).

Where available, coverage values were derived from post-campaign coverage surveys that asked participants whether they could recall having ever received a YF vaccine (and/or their children) and whether they could present an official YF vaccination record. Post-campaign coverage surveys were used in preference of administrative data because these generally provide more reliable coverage estimates.[7] Finally, duplicate data were removed for campaign records covering the same location and time period, retaining the most precise records. A description of the data issues encountered and actions taken is included in Supplementary table 1 and a summary of the information extracted from the WERs, DONs, MMWRs and WHO reports can be found in Supplementary table 2.

Supplementary table 1: Extraction issues with preventive and outbreak response vaccination campaign data and actions taken.

Data issue	Action taken
Campaign described as "planned" or "in progress" at the time of the report but the number of vaccine doses to be administered was specified	Assumed that one dose was administered per person in targeted locations
Campaign described as "planned" and only the target population size was specified	Campaign was excluded
Target age group was described as "children"	Assumed that this referred to children less than five years of age
Target age groups not specified	Assumed that individuals in all age groups had an equal chance of receiving a vaccine dose
Coverage was reported to be greater than 100%	Coverage resolved to 100%
Coverage range was stated (eg, 90–95%)	Recorded lower limit (eg, 90%)
The report quoted the number of people vaccinated rather than number of doses or coverage	Recorded as the number of vaccine doses
Coverage was not directly reported (ie, only the number of vaccine doses administered, number of people vaccinated or target population size *etc.* was provided)	Estimated coverage by calculating the size of target population (denominator) using WorldPop and UN WPP data
Coverage reported without specifying the size of the target population	Assumed that target population size equalled population of target locations
Target population size reported without specifying the coverage value	Coverage was assumed to be 100%
The number of vaccines doses, the number of people vaccinated or the size of the target population was reported to be greater than the total estimated number of people in the specified target locations	Target population size was replaced with estimated population size of target locations, meaning that coverage was 100%
The number of vaccine doses was provided without reporting the achieved coverage or the target population size	Assumed that number of people vaccinated equalled number of vaccine doses
Target population size not reported	Assumed target population size equalled population of target

	locations
Coverage or number of vaccine doses was aggregated over a number of target locations	Assumed that individuals in each location had an equal chance of receiving a vaccine dose
Unclear whether the campaign was targeting locations at the third or fourth order administrative unit (ie, locations have the same name and it was not clear which was targeted in the report)	Assumed campaign targeted the third order administrative unit
Target locations provided at the third or fourth order administrative level	Coverage at the second administrative level was calculated
Target locations not specified	Divided number of people vaccinated by estimated population size within broad target locations or within the entire country
Target locations listed as "endemic" or "at risk" regions	Divided number of people vaccinated by estimated population size within districts classified as endemic or at-risk by the WHO[4]
Number of vaccine doses aggregated over multiple years	Assumed that an equal number of doses were administered each year
Different coverage values reported from a post-campaign survey based on 1) combination of respondent recall and the presence of official vaccination records and 2) vaccination records only	Used coverage value based on combination of respondent recall and presence of official vaccination records

Supplementary table 2: Preventive and outbreak response campaigns reported in each Weekly Epidemiological Report (WER)[8], Disease Outbreak News (DON)[9], Morbidity and Mortality Weekly Report (MMWR), and two World Health Organization reports: one on mass immunization in West and Central Africa in the 1940s and 1950s[5] and another on the 2016 YF epidemic in the Democratic Republic of Congo[6]. The DONs are identified by the date of issue in the format ddmmyy and the WERs by four digits representing the volume and issue numbers.

Data source	Target country	Target subnational location/s (administrative level/s of target location/s)	Campaign start year	Campaign end year
DON011102	Senegal	Mbacké, Bambey (2)	2002	2002
DON031105	Mali	Kadiolo, Kolondieba (2)	2005	2005
DON031213	Sudan	Western Darfur, Southern Darfur, Nothern Darfur (1)	2012	2012
DON070601	Peru	Puinahua, San Pablo , Iquitos (3)	2001	2001
DON080110	Côte d'Ivoire	Kaniso, Sandégué, Minignan, Samantiguila (3)	2009	2009
DON080808	Côte d'Ivoire	Abidjan (2)	2001	2001
DON081013	Cameroon	Dibombari, Edéa, Loum, Manjo, Manoka, Mbanga, Melong, Ndom, Ngambé, Nkondjock, Nkongsamba, Pouma, Yabass (3)	2013	2013
DON110304	Liberia	Margibi, Grand Bassa, Grand Gedeh (1)	2004	2004
DON281105	Mali	Kangaba, Kati, Kayes, Kéniébe, Kita, Bafoulabé (2)	2005	2005
DON300903	Sierra Leone	Tonkolili (2)	2003	2003
DON260816	Angola	Luanda, Benguela, Cuanza Sul, Huambo, Huíla, Uíge (1)	2016	2016
Durieux, 1956	Mauritania	NA	1939	1953
	Senegal	NA	1939	1953
	Mali	NA	1939	1953
	Guinea	NA	1939	1953

	Côte d'Ivoire	NA	1939	1953
	Burkina Faso	NA	1939	1953
	Benin	NA	1939	1953
	Niger	NA	1939	1953
	Togo	NA	1940	1953
	Chad	NA	1944	1953
	Central African Republic	NA	1944	1953
	Republic of Congo	NA	1944	1953
	Gabon	NA	1944	1953
WER4543	Peru	Oxapampa, Tarma, Satipo (2)	1969	1970
WER4631	Ghana	Upper East, Upper West (1)	1969	1969
	Ghana	Brong-Ahafo Region (1)	1970	1970
	Nigeria	Plateau, Bauchi (1)	1969	1969
WER4724	Democratic Republic of the Congo	Gemena, Dungu (3)	1958	1958
	Democratic Republic of the Congo	Gemena (3)	1972	1972
WER5034	Nigeria	Abia, Anambra, Ebonyi, Enugu, Imo, Delta, Rivers (1)	1974	1974
WER5139	Bolivia	Chuquisaca, La Paz, Santa Cruz, Cochabamba, Tarija (1)	1975	1975
	Ecuador	Napo Province (1)	1975	1975
WER5224	Colombia	Casanare (1)	1976	1976
WER5423	Gambia	NA	1978	1979
WER5440	Colombia	Norte de Santander (1)	1979	1979
WER5440	Venezuela	Zulia, Trujillo, Mérida (1)	1941	1978
WER5511	Ghana	Volta, Eastern, Brong-Ahafo, Upper East, Upper West (1)	1979	1979
WER5545	Senegal	Fatick, Kaolack, Kaffrine, Tambacounda, Kédougou, Kolda, Sédhiou, Ziguinchor (1)	1979	1979
WER5545	Trinidad and Tobago	NA	1979	1979
WER5633	Cameroon	Diamaré (2)	1980	1980
WER5739	Bolivia	NA	1981	1982
	Brazil	Maranhão, Acre, Amazonas, Pará, Mato Grosso, Mato Grosso del Sul, Goiás, Rondonia, Federal District, Roraima, Amapá (1)	1937	1983
	Senegal	Thiès (1)	1981	1981
WER5841	Ghana	NA	1980	1982
WER5943	Burkina Faso	Centre (1)	1983	1983
	Ghana	Northern, Upper East (1)	1983	1983

WER6043	Togo	Central Region (1)	1983	1983
WER6056	Brazil	Presidente Prudente (1)	1985	1985
WER6406	Mali	Kita, Kati, Kangaba, Kolokani, Bafoulabé, Diéma, Kéniéba, Nioro du Sahel, Yélimané, Banamba, Dioïla, Koulikoro, Nara, Mopti, Ségou, Sikasso (2)	1987	1987
WER6423	Bolivia	Santa Cruz, Montero, Carrasco, Ichilo (3)	1985	1989
WER6528	Angola	Luanda (1)	1988	1988
WER6733	Nigeria	Abuja, Akwa Ibam, Anamba, Bauchi, Delta, Edo, Benue, Borno, Cross River, Adamawa, Taraba, Imo, Kaduna, Kano, Katsina, Kwara, Lagos, Niger, Oyo, Ogun, Ondo, Plateau, Rivers, Sokoto (1)	1986	1989
WER7010	Kenya	Baringo, Elgeyo Marakwet (2)	1993	1993
WER7015	Nigeria	Orsu (2)	1994	1994
WER7050	Liberia	Grand Bassa (1)	1995	1995
WER7051/52	Sierra Leone	Kenema (2)	1995	1995
WER7124	Gambia	NA	1978	1979
WER7142	Liberia	Buchanan, Grand Bassa (1)	1995	1995
WER7145	Benin	Atakora, Borgou (1)	1996	1996
WER7211	Bolivia	Cochabamba, La Paz, Beni, Santa Cruz (1)	1997	1997
WER7335	Brazil	Afuá (3)	1998	1998
WER7345	Venezuela	Amazonas (1)	1998	1998
WER7346	Benin	Atakora, Borgou (1)	1998	1998
	Ghana	Upper East Region (1)	1996	1997
	Liberia	Voinjama, Kolahun, Foya (2)	1997	1997
WER7401	Burkina Faso	Batié (3)	1998	1998
WER7540	Bolivia	Cordillera (2)	1999	1999
	Brazil	NA	1990	2001
	Liberia	Nimba (1)	1998	1998
WER7743	Senegal	NA	2002	2002
WER8029	Burkina Faso	Bobo-Dioulasso, Nouna, Solenzo, Sindou, Gaoua (3)	2004	2004
	Colombia	Cesar, Magdalena, La Guajira (1)	2003	2004
	Liberia	Bong, Nimba (1)	2004	2004
	Mali	Kita (2)	2004	2004
	Venezuela	Unspecified high risk areas	2006	2006
	Venezuela	Unspecified enzootic areas	2002	2004
WER8051/52	Cote d'Ivoire	Bondoukou, Bouna (3)	2005	2005
WER8133	Burkina Faso	Batié, Gaoua, Banfora (3)	2005	2005
	Senegal	Bakel (2)	2005	2005
	Sudan	South Kordofan (1)	2005	2005
WER8307	Brazil	Federal District, Goiás, Mato Grosso do Sul (1)	2008	2008

WER8308	Cameroon	NA	2006	2006
	Central African Republic	Yaloke-Bossembele (2)	2006	2006
	Côte d'Ivoire	Ouragahio, Korhogo (4)	2006	2006
	Ghana	Sene (2)	2006	2006
	Mali	Yanfolila (2)	2005	2006
WER8340	Guinea	NA	2005	2005
WER8350	Mali	NA	2005	2005
	Senegal	NA	2002	2006
WER8413	Bolivia	NA	2007	2007
	Cameroon	Akonolinga, Zoétélé (3)	2007	2007
	Senegal	Dagana (and Richard Toll), Podor (and Pete), Linguère, Kédougou, Salémata, Saraya, Thiès (and Pout), Kaolack (and Ndofane), Foundiougne (and Sokone), Guinguinéo, Mbour (and Poponguine, Joal and Dioffior), Tivaouana (and Mekhe), Louga, Saint Louis, Saraya (2)	2007	2007
	Togo	Savanes, Kara , Centrale, Plateaux, Maritime (1)	2007	2007
WER8604	Argentina	Bella Vista, Berón de Astrada, Capital, Concepción, Curuzú Cuatiá, Empedrado, Esquina, Ganeral Paz, General Alvear, Goya, Itatí, Ituzaingó, Lavalle, Mburucuy, Mercedes, Monte Caseros, Paso de los Libres, Saladas, San Cosme, San Luis del Palmar, San Martin, San Miguel, San Roque, Santo Tomé, Sauce, 25 de Mayo, Apóstoles, Cainguás, Candelaria, Eldorado, General Manuel Belgrano, Guarani, Iguazu, Leandro N. Alem, Libertador General San Ma, Montecarlo, Oberá, San Ignacio, San Javier, San Pedro (2)	2008	2008
	Burkina Faso	Ouahigouya (3)	2008	2008
	Cameroon	Abong Mbang, Nguelemendouka (3)	2008	2008
	Cameroon	Buea, Mbongue (3)	2009	2009
	Central African Republic	Bozoum, Lobaye, Sangha Mbaéré, Boali, Damara, Bria, Ouadda, Yalinga (2)	2008	2008
	Central African Republic	Yaloke-Bossembelle (2)	2009	2009
	Côte d'Ivoire	Abidjan (2)	2008	2008
	Côte d'Ivoire	Madinani, Minignan (3)	2009	2009
	Guinea	Bounouma, Urbain, Kouankan (3)	2008	2008
	Guinea	Mandiana (2)	2009	2009
	Guinea	Faranah (2)	2009	2009
	Liberia	Nimba, Zorzor, Voinjama (1,2)	2009	2009
	Paraguay	NA	2008	2008
	Peru	Unspecified endemic areas	2004	2008
	Republic of Congo	Cuvette-Ouest (1)	2009	2009
	Sierra Leone	Bo (2)	2008	2008
WER8634	Cameroon	Bandjoun, Foumbot, Kumba, Muyunka (3)	2010	2010

	Democratic Republic of the Congo	Titule, Buta (3)	2010	2010
	Guinea	NA	2010	2010
WER8828	Cameroon	NA	2012	2012
	Chad	Biltine, Assongha, Sila (1)	2012	2012
	Côte d'Ivoire	Béoumi, Katiola, Mankon, Séguéla (3)	2011	2011
	Ghana	Builsa, Kassena-Nankana-West, Kintampo-South (2)	2012	2012
	Senegal	Kédougou, Salémata, Saraya (2)	2012	2012
	Sudan	Algeniema, Beida, Habila, Krenik, Azoum, Nertati, Wadi-Salith, Zalingi, Kass, Sharg-Aljebel, Alsiraif, Saraj-Omgra, Greater Bandasi, Forbranga, Mkjar, Nyala, Shataia, Edd Elfirsan, Elwihda, Kabkabyaia, Shaeriya (2)	2012	2012
	Uganda	Abim, Agago, Ktigum, Lamwo, Pader (1)	2011	2011
WER8927	Cameroon	Dschang, Ngoumou (3)	2014	2014
	Democratic Republic of the Congo	Lubao, Kamana, Ludimbi-Lukula (3)	2013	2013
	Ethiopia	South Ari, Bena Tsmay, Dasenech, Gnangatom, Hammer, Jinka City, Malle, North Ari, Selamago (3)	2013	2013
	Nigeria	Akwa Ibbom, Cross Rivers, Nasarawa (1)	2013	2013
	Sudan	Northern Kordofan, Southern Kordofan (1)	2013	2013
WER9026	Cameroon	West Region, Southwest Region (1)	2014	2014
	Democratic Republic of the Congo	Bondo, Buta, Kinkondja (3)	2014	2014
WER9132	Cameroon	Adamaoua, Centre, East, North, North-West, West, South (1)	2015	2015
	Sudan	Gedarif, Kassala, North Kordofan, Red Sea (1)	2015	2015
WMMR020482	Ghana	NA	1981	1981
WHO, 2016	Democratic Republic of the Congo	Kwango, Cataractes Lukaya, Lulua, Lualaba, Kinshasa, Bas-Fleuve, Kasai (2)	2016	2016

Processing supplementary data for Brazil

The Brazilian national immunization program information system provided more detailed data for Brazil that contained the number of vaccine doses administered in each second administrative level (municipality) in one-year age bands (from zero to four years), five-year age bands (five to 14 years) and broader age groups, 15 to 59 years and 60 plus years annually from 2006 to 2015. We assumed that this data captured all doses administered to the Brazilian population between 2006 and 2015 and thus routine and mass preventive and outbreak campaign data from other sources during this period were excluded from coverage calculations. The WHO/UNICEF estimates for Brazil reported routine coverage of infants in YF at-risk areas from 1998 onwards.[3] We included the WHO/UNICEF estimates from 1998 to 2005.

To assess the difference between YF vaccination coverage estimates for Brazil when using these two different data sources for the years from 2006 to 2015, we produced two sets of coverage maps (Supplementary figure 1) and two sets of estimates of the number of individuals requiring vaccination (Supplementary table 3).

Supplementary table 3: Estimated number of individuals (in millions) that still need to be vaccinated against yellow fever in 2016 in order to achieve the population coverage threshold of 80% recommended by the WHO

to prevent outbreaks in every municipality of Brazil. Estimates were calculated using vaccination data either from the Brazilian NIP or from a range of sources listed in manuscript Table 1.

	At-risk municipalities only			All municipalities		
	Conservative	Untargeted	Optimistic	Conservative	Untargeted	Optimistic
Brazilian NIP data	40.3	6.8	2.1	121.8	49.7	27.1
Data sources from Table 1	40.0	2.0	0	122.3	26.3	0

Supplementary figure 1: Estimated proportion of the population in 2016 across all age cohorts who have ever received a yellow fever (YF) vaccine at municipality level in Brazil. Vaccination coverage was calculated using the three alternative vaccination-targeting scenarios; targeted (A & D), untargeted, unbiased (B & E), and untargeted, biased (C & F). The panels also display estimates calculated using vaccination data either from the Brazilian NIP (A-C) or from a range of sources listed in manuscript table 1 (D-F).

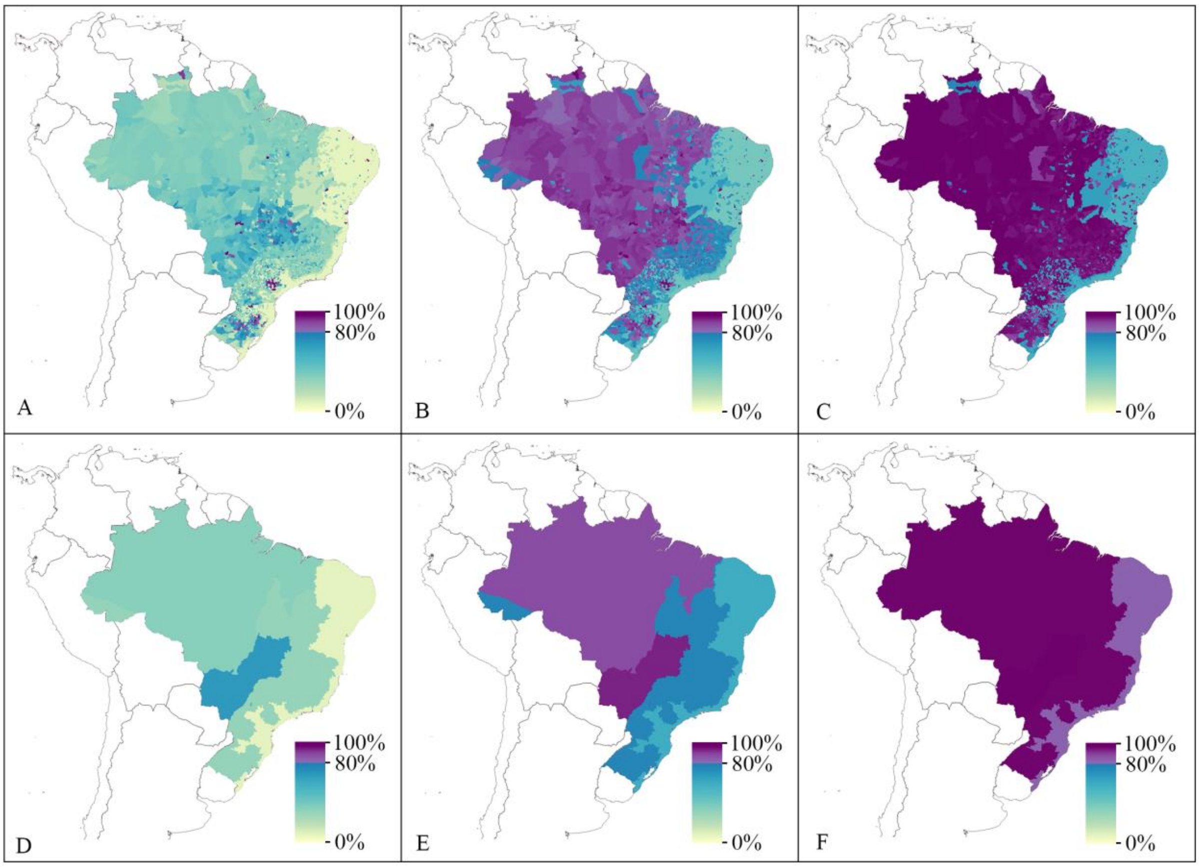

Processing demographic data

Annual age-structured population counts for each district were required to calculate population vaccination coverage and to estimate denominators for preventive and outbreak response vaccination campaigns where coverage values could not be directly extracted from the data source. Estimates of these population counts were derived from a combination of United Nations World Population Prospects (UNWPP) (https://esa.un.org/unpd/wpp/),[10] WorldPop Project (http://www.worldpop.org.uk/)[11] and Gridded Population of the World (GPW) (http://sedac.ciesin.columbia.edu/data/collection/gpw-v4)[12] data. The latter two data sets were mosaicked together, with WorldPop data used in preference where they overlapped, to produce a fine scale human population density data surface for 2015 for each 1 km × 1 km grid square.

The UNWPP[10] estimates of the population size by country in five-year age bands for each year between 1950 and 2015 were disaggregated into annual birth cohorts using the methods described by Garske and colleagues[13]. This method required UNWPP data on infant and child mortality rates. To achieve higher spatial resolution of population distribution, these estimates were combined with the fine scale human population density data for 2015. By allocating each 1 km × 1 km grid square (or portion thereof) to a district, the proportion of each

country's population living in any particular district was estimated. It was assumed that age distributions and population growth were homogeneous within each country, and that the population proportions for each district obtained from WorldPop and GPW were applicable for all other years eg, trends in urbanisation were not captured. To estimate the population in each district for 2016, we assumed the same growth rate as the previous year.

Vaccination probability and coverage estimates

We tracked each age cohort (from ages zero to 99) in every district through time – from their birth year through to 2016 (ie, the earliest cohort was born in 1871 in order to track coverage of individuals aged 99 in 1970) – updating the coverage level whenever a routine, preventive or outbreak response campaign was conducted. The equations below show how the proportion vaccinated, P, for each age cohort, was calculated under each of the three vaccination-targeting scenarios, given any new vaccination activity, V.

Untargeted, unbiased: assuming vaccination history was not taken into account and all individuals had an equal chance of receiving a vaccine regardless of their previous vaccination status.

$$P = P_{(\text{proportion of cohort previously vaccinated})} + P_{(\text{proportion of cohort vaccinated in V})} - P_{(\text{proportion of cohort previously vaccinated})} \times P_{(\text{proportion of cohort vaccinated in V})}$$

Targeted: assuming that vaccination history was taken into account and only non-vaccinated individuals were targeted by immunisation campaigns.

$$P = P_{(\text{proportion of cohort previously vaccinated})} + P_{(\text{proportion of cohort vaccinated in V})}$$

Untargeted, biased: assuming that vaccination history was not taken into account and that previously vaccinated individuals were more likely to be targeted inadvertently.

$$P = \text{maximum}\ (P_{(\text{proportion of cohort previously vaccinated})}\ ,\ P_{(\text{proportion of cohort vaccinated in V})})$$

To estimate population-wide vaccination under each scenario, we calculated the weighted mean proportion vaccinated across all age cohorts, weighted by population size.

Results appendix

Supplementary figures 2-5: Estimated proportion of individuals within each age group that have ever received a YF vaccine (vaccination coverage) for each country at risk of YFV transmission, based on the *untargeted, unbiased* vaccination-targeting scenario. Separate plots are shown for the beginning of each decade from 1970 to 2010.

Supplementary figure 2

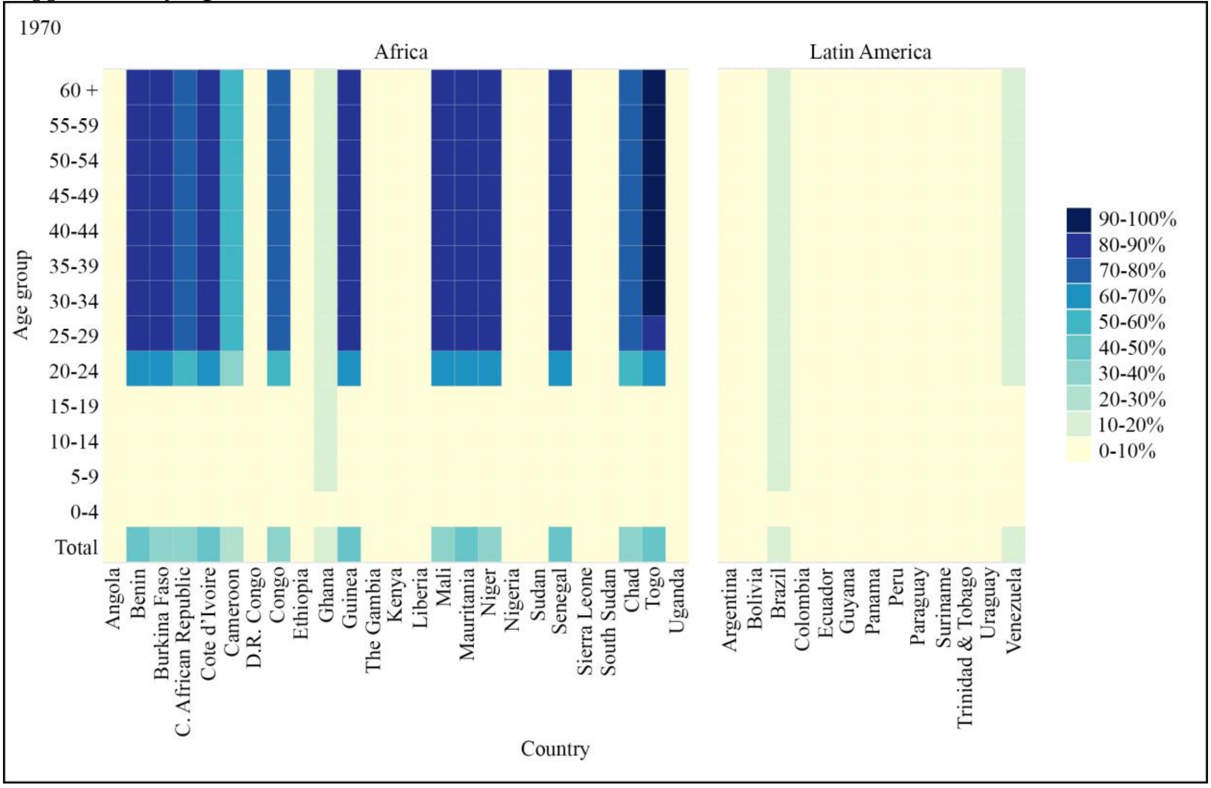

Supplementary figure 3

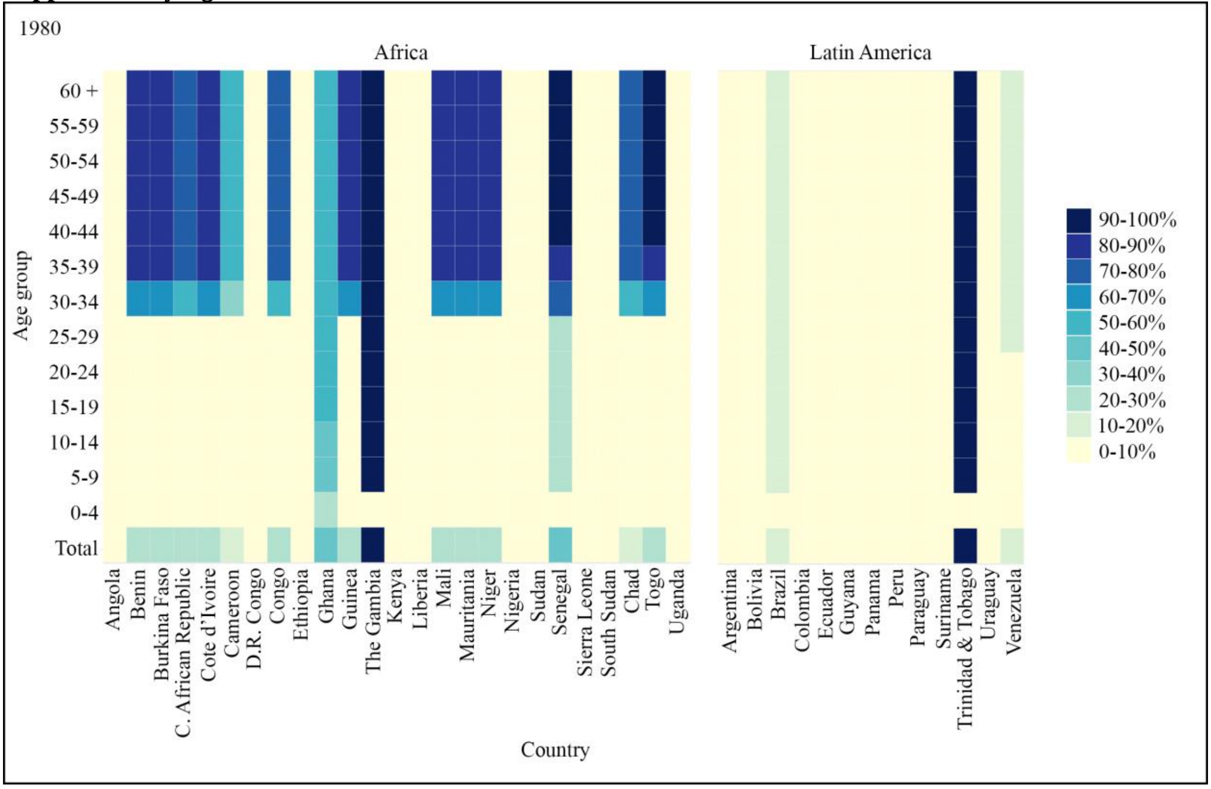

Supplementary figure 4

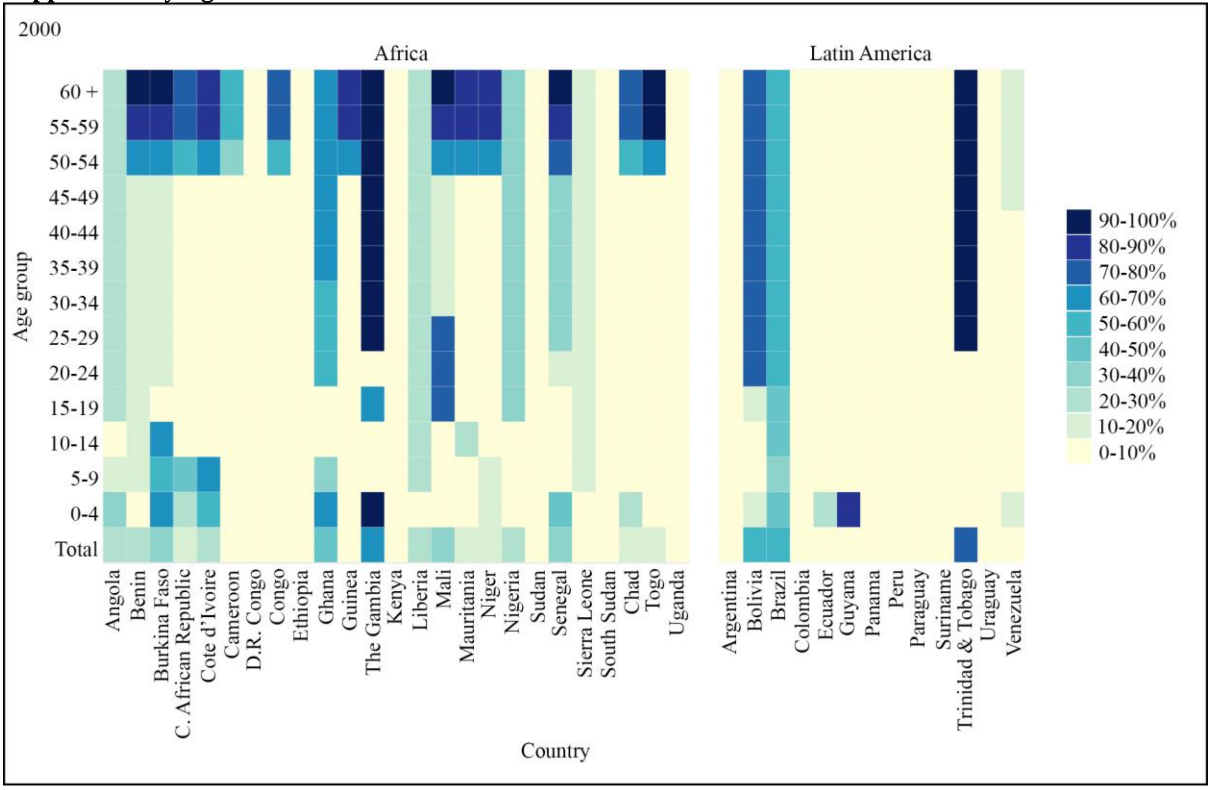

Supplementary figure 5

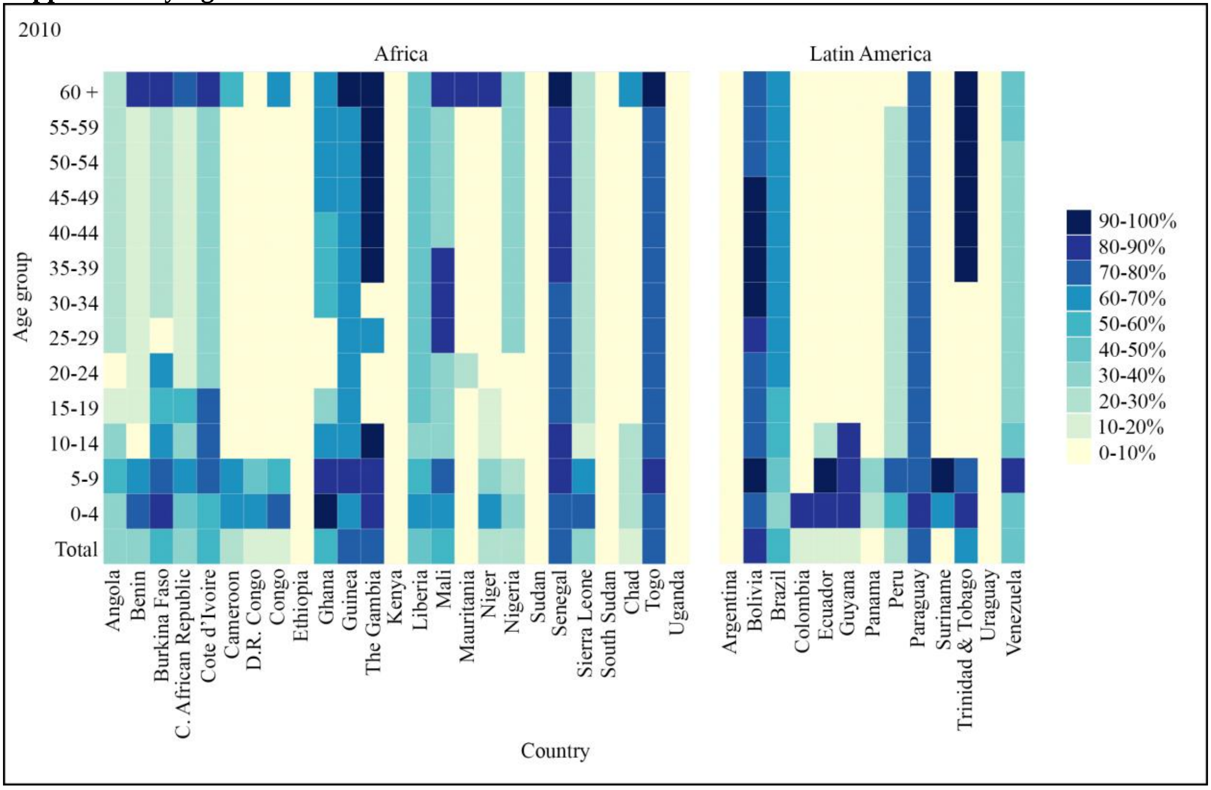

References

1. World Health Organization. WHO/UNICEF Joint Reporting Process. http://www.who.int/immunization/monitoring_surveillance/routine/reporting/reporting/en/. (accessed April 24, 2017).
2. Burton A, Monasch R, Lautenbach B, Gacic-Dobo M, Neill M, Karimov R, et al. WHO and UNICEF estimates of national infant immunization coverage: methods and processes. *Bull World Health Organ* 2009; **87**: 535–41.
3. World Health Organization. WHO UNICEF review of national immunization coverage, 1980-2015. http://apps.who.int/immunization_monitoring/globalsummary/wucoveragecountrylist.html. (accessed August 26, 2016).
4. Jentes ES, Poumerol G, Gershman MD, Hill DR, Lemarchand J, Lewis RF, et al. The revised global yellow fever risk map and recommendations for vaccination, 2010: consensus of the Informal WHO Working Group on Geographic Risk for Yellow Fever. *Lancet Infect Dis* 2011; **11**: 622–32.
5. Durieux K. Mass yellow fever vaccination in French Africa south of the Sahara. *Monograph Series World Health Organisation* 1956; **30**: 115–22.
6. World Health Organization. Report on the fight against the yellow fever epidemic 2016 in the Democratic Republic of Congo, World Health Organisation, Kinshasa, 2016.
7. Lim S, Stein D, Charrow A, Murray C. Tracking progress towards universal childhood immunisation and the impact of global initiatives: a systematic analysis of three-dose diphtheria, tetanus, and pertussis immunisation coverage. *Lancet* 2008; **372**: 2031–46
8. World Health Organization. The Weekly Epidemiological Record (WER). http://www.who.int/wer/en/ (accessed August 26, 2016).
9. World Health Organization. Disease Outbreak News (DONs). http://www.who.int/csr/don/en/ (accessed August 26, 2016).
10. United Nations. World Population Prospects, the 2015 Revision. https://esa.un.org/unpd/wpp/Download/Standard/Population/ (accessed October 13, 2016).
11. WorldPop. Gridded Population Distributions, 2015. http://www.worldpop.org.uk/ (accessed January, 2016)
12. Center for International Earth Science Information Network - CIESIN - Columbia University, United Nations Food + Agriculture Programme - FAO, Centro Internacional de Agricultura Tropical - CIAT. Gridded Population of the World, Version 4 (GPWv4): Population Count Grid. Palisades, NY: NASA Socioeconomic Data and Applications Center (SEDAC); 2005.
13. Garske T, Van Kerkhove MD, Yactayo S, Ronveaux O, Lewis RF, Staples JE, et al. Yellow fever in Africa: estimating the burden of disease and impact of mass vaccination from outbreak and serological data. *Plos Med* 2014; **11**: e1001638.

Supplementary table 4: Vaccination coverage estimates and the estimated number of individuals that still need to be vaccinated against YF in 2016 in every district of all countries at risk of YF virus transmission (ie, classified as 'endemic', 'transitional' or 'low potential risk of exposure' by the WHO range maps) to achieve the 80% population coverage threshold recommended by the WHO to prevent outbreaks. An excel version of this data is also available in Dryad Digital Repository [link to be inserted upon manuscript acceptance].

				Vaccination coverage 2016			Individuals requiring vaccination to meet 80% target		
Country ID	Country name	GAUL code	District name	Conservative	Untargeted	Optimistic	Conservative	Untargeted	Optimistic
AGO	Angola	4206	Ambriz	0.36	0.36	0.36	9573	9573	9573
AGO	Angola	4207	Bula Atumba	0.36	0.36	0.36	10821	10821	10821
AGO	Angola	4208	Dande	0.36	0.36	0.36	47601	47601	47601
AGO	Angola	4209	Dembos	0.36	0.36	0.36	11617	11617	11617
AGO	Angola	4210	Icolo E Bengo	0.36	0.36	0.36	35086	35086	35086
AGO	Angola	4211	Nambuangongo	0.36	0.36	0.36	62872	62872	62872
AGO	Angola	4212	Pango Aluquem	0.36	0.36	0.36	9227	9227	9227
AGO	Angola	4213	Quissama	0.36	0.36	0.36	17678	17678	17678
AGO	Angola	4214	Baia Farta	0.67	0.78	0.88	12823	2209	0
AGO	Angola	4215	Balombo	0.67	0.78	0.88	3640	627	0
AGO	Angola	4216	Benguela	0.67	0.78	0.88	76385	13159	0
AGO	Angola	4217	Bocoio	0.67	0.78	0.88	7335	1264	0
AGO	Angola	4218	Caimbambo	0.67	0.78	0.88	5788	997	0
AGO	Angola	4219	Chongoroi	0.67	0.78	0.88	9836	1694	0
AGO	Angola	4220	Cubal	0.67	0.78	0.88	29602	5100	0
AGO	Angola	4221	Ganda	0.67	0.78	0.88	24496	4220	0
AGO	Angola	4222	Lobito	0.67	0.78	0.88	114739	19766	0
AGO	Angola	4223	Andulo	0.36	0.36	0.36	86974	86974	86974
AGO	Angola	4224	Camacupa	0.36	0.36	0.36	82752	82752	82752
AGO	Angola	4225	Catabola	0.36	0.36	0.36	60040	60040	60040
AGO	Angola	4226	Chinguar	0.36	0.36	0.36	82422	82422	82422
AGO	Angola	4227	Chitembo	0.36	0.36	0.36	53171	53171	53171
AGO	Angola	4228	Cuemba	0.36	0.36	0.36	33976	33976	33976
AGO	Angola	4229	Cunhinga	0.36	0.36	0.36	11699	11699	11699
AGO	Angola	4230	Kuito	0.36	0.36	0.36	146979	146979	146979
AGO	Angola	4231	N'harea	0.36	0.36	0.36	11801	11801	11801
AGO	Angola	4232	Belize	0.36	0.36	0.36	13058	13058	13058
AGO	Angola	4233	Buco-zau	0.36	0.36	0.36	32020	32020	32020
AGO	Angola	4234	Cabinda	0.36	0.36	0.36	144274	144274	144274
AGO	Angola	4235	Cacongo (landana)	0.36	0.36	0.36	22111	22111	22111
AGO	Angola	4236	Calai	0.36	0.36	0.36	25952	25952	25952

AGO	Angola	4237	Cuangar	0.36	0.36	0.36	6423	6423	6423
AGO	Angola	4238	Cuchi	0.36	0.36	0.36	18391	18391	18391
AGO	Angola	4239	Cuito Cuanavale	0.36	0.36	0.36	19065	19065	19065
AGO	Angola	4240	Dirico	0.36	0.36	0.36	12080	12080	12080
AGO	Angola	4241	Mavinga	0.36	0.36	0.36	8653	8653	8653
AGO	Angola	4242	Menongue	0.36	0.36	0.36	81484	81484	81484
AGO	Angola	4243	Nankova	0.36	0.36	0.36	6024	6024	6024
AGO	Angola	4244	Rivungo	0.36	0.36	0.36	15644	15644	15644
AGO	Angola	4245	Amboim	0.67	0.78	0.88	23734	4089	0
AGO	Angola	4246	Cassongue	0.67	0.78	0.88	12849	2214	0
AGO	Angola	4247	Conda	0.67	0.78	0.88	7504	1293	0
AGO	Angola	4248	Ebo	0.67	0.78	0.88	14225	2451	0
AGO	Angola	4249	Libolo	0.67	0.78	0.88	13680	2357	0
AGO	Angola	4250	Mussende	0.67	0.78	0.88	9233	1591	0
AGO	Angola	4251	Porto Amboim	0.67	0.78	0.88	17352	2989	0
AGO	Angola	4252	Quibala	0.67	0.78	0.88	18592	3203	0
AGO	Angola	4253	Quilenda	0.67	0.78	0.88	8695	1498	0
AGO	Angola	4254	Seles	0.67	0.78	0.88	22072	3802	0
AGO	Angola	4255	Sumbe	0.67	0.78	0.88	26553	4574	0
AGO	Angola	4256	Waku Kungu	0.67	0.78	0.88	16282	2805	0
AGO	Angola	4257	Cahama	0.36	0.36	0.36	33218	33218	33218
AGO	Angola	4258	Cuanhama	0.36	0.36	0.36	97138	97138	97138
AGO	Angola	4259	Curoca	0.36	0.36	0.36	25908	25908	25908
AGO	Angola	4260	Cuvelai	0.36	0.36	0.36	22692	22692	22692
AGO	Angola	4261	Namacunde	0.36	0.36	0.36	68673	68673	68673
AGO	Angola	4262	Ombadja	0.36	0.36	0.36	67885	67885	67885
AGO	Angola	4263	Bailundo	0.67	0.78	0.88	7377	1271	0
AGO	Angola	4264	Caala	0.67	0.78	0.88	52130	8981	0
AGO	Angola	4265	Ekunha	0.67	0.78	0.88	11759	2026	0
AGO	Angola	4266	Huambo	0.67	0.78	0.88	93033	16027	0
AGO	Angola	4267	Katchiungo	0.67	0.78	0.88	9011	1552	0
AGO	Angola	4268	Londuimbali	0.67	0.78	0.88	20728	3571	0
AGO	Angola	4269	Longonjo	0.67	0.78	0.88	11686	2013	0

AGO	Angola	4270	Mungo	0.67	0.78	0.88	3945	680	0
AGO	Angola	4271	Tchikala-tcholo.	0.67	0.78	0.88	9418	1622	0
AGO	Angola	4272	Tchindjenje	0.67	0.78	0.88	1233	212	0
AGO	Angola	4273	Ukuma	0.67	0.78	0.88	9332	1608	0
AGO	Angola	4274	Caconda	0.67	0.78	0.88	33851	5832	0
AGO	Angola	4275	Cacula	0.67	0.78	0.88	9339	1609	0
AGO	Angola	4276	Caluquembe	0.67	0.78	0.88	44082	7594	0
AGO	Angola	4277	Chibia	0.67	0.78	0.88	25317	4361	0
AGO	Angola	4278	Chicomba	0.67	0.78	0.88	20410	3516	0
AGO	Angola	4279	Chipindo	0.67	0.78	0.88	6120	1054	0
AGO	Angola	4280	Gambos	0.67	0.78	0.88	9188	1583	0
AGO	Angola	4281	Humpata	0.67	0.78	0.88	3339	575	0
AGO	Angola	4282	Jamba	0.67	0.78	0.88	9162	1578	0
AGO	Angola	4283	Kuvango	0.67	0.78	0.88	10356	1784	0
AGO	Angola	4284	Lubango	0.67	0.78	0.88	65925	11357	0
AGO	Angola	4285	Matala	0.67	0.78	0.88	25386	4373	0
AGO	Angola	4286	Quilengues	0.67	0.78	0.88	20592	3547	0
AGO	Angola	4287	Quipungo	0.67	0.78	0.88	14734	2538	0
AGO	Angola	4288	Ambaca	0.36	0.36	0.36	23736	23736	23736
AGO	Angola	4289	Banga	0.36	0.36	0.36	2984	2984	2984
AGO	Angola	4290	Bolongongo	0.36	0.36	0.36	7370	7370	7370
AGO	Angola	4291	Cambambe	0.36	0.36	0.36	17874	17874	17874
AGO	Angola	4292	Cazengo (ndalatando)	0.36	0.36	0.36	51782	51782	51782
AGO	Angola	4293	Golungo Alto	0.36	0.36	0.36	13327	13327	13327
AGO	Angola	4294	Gonguembo	0.36	0.36	0.36	7162	7162	7162
AGO	Angola	4295	Lucala	0.36	0.36	0.36	7969	7969	7969
AGO	Angola	4296	Quiculungo	0.36	0.36	0.36	5548	5548	5548
AGO	Angola	4297	Samba Caju	0.36	0.36	0.36	18247	18247	18247
AGO	Angola	4298	Cacuaco	0.77	0.88	0.98	23813	0	0
AGO	Angola	4299	Luanda	0.77	0.88	0.98	103684	0	0
AGO	Angola	4300	Viana	0.77	0.88	0.98	59230	0	0
AGO	Angola	4301	Cambulo	0.36	0.36	0.36	40599	40599	40599
AGO	Angola	4302	Capenda Camulemba	0.36	0.36	0.36	42152	42152	42152

AGO	Angola	4303	Caungula	0.36	0.36	0.36	34050	34050	34050
AGO	Angola	4304	Chitato	0.36	0.36	0.36	56571	56571	56571
AGO	Angola	4305	Cuango	0.36	0.36	0.36	34232	34232	34232
AGO	Angola	4306	Cuilo	0.36	0.36	0.36	15081	15081	15081
AGO	Angola	4307	Lubalo	0.36	0.36	0.36	20666	20666	20666
AGO	Angola	4308	Lucapa	0.36	0.36	0.36	44751	44751	44751
AGO	Angola	4309	Xa-muteba	0.36	0.36	0.36	87652	87652	87652
AGO	Angola	4310	Cacolo	0.36	0.36	0.36	25945	25945	25945
AGO	Angola	4311	Dala	0.36	0.36	0.36	34475	34475	34475
AGO	Angola	4312	Muconda	0.36	0.36	0.36	50320	50320	50320
AGO	Angola	4313	Saurimo	0.36	0.36	0.36	83696	83696	83696
AGO	Angola	4314	Cacuso	0.36	0.36	0.36	31382	31382	31382
AGO	Angola	4315	Calandula	0.36	0.36	0.36	63145	63145	63145
AGO	Angola	4316	Cambundi-catembo	0.36	0.36	0.36	15536	15536	15536
AGO	Angola	4317	Cangandala	0.36	0.36	0.36	10120	10120	10120
AGO	Angola	4318	Caombo	0.36	0.36	0.36	17027	17027	17027
AGO	Angola	4319	Kiwaba N'zogi	0.36	0.36	0.36	15144	15144	15144
AGO	Angola	4320	Kunda Dia-baze	0.36	0.36	0.36	9677	9677	9677
AGO	Angola	4321	Luquembo	0.36	0.36	0.36	10258	10258	10258
AGO	Angola	4322	Malanje	0.36	0.36	0.36	135262	135262	135262
AGO	Angola	4323	Marimba	0.36	0.36	0.36	9760	9760	9760
AGO	Angola	4324	Massango	0.36	0.36	0.36	13004	13004	13004
AGO	Angola	4325	Mucari	0.36	0.36	0.36	8493	8493	8493
AGO	Angola	4326	Quela	0.36	0.36	0.36	24831	24831	24831
AGO	Angola	4327	Quirima	0.36	0.36	0.36	11388	11388	11388
AGO	Angola	4328	Alto Zambeze	0.36	0.36	0.36	14940	14940	14940
AGO	Angola	4329	Camanongue	0.36	0.36	0.36	13262	13262	13262
AGO	Angola	4330	Kameia Lumege	0.36	0.36	0.36	29267	29267	29267
AGO	Angola	4331	Leua	0.36	0.36	0.36	22241	22241	22241
AGO	Angola	4332	Luacano	0.36	0.36	0.36	21867	21867	21867
AGO	Angola	4333	Luau	0.36	0.36	0.36	22573	22573	22573
AGO	Angola	4334	Luchazes	0.36	0.36	0.36	17904	17904	17904
AGO	Angola	4335	Luena (moxico)	0.36	0.36	0.36	131183	131183	131183

AGO	Angola	4336	Lumbala N'guimbo	0.36	0.36	0.36	2722	2722	2722
AGO	Angola	4337	Bibala	0.36	0.36	0.36	30126	30126	30126
AGO	Angola	4338	Camacuio	0.36	0.36	0.36	23922	23922	23922
AGO	Angola	4339	Namibe	0.36	0.36	0.36	103723	103723	103723
AGO	Angola	4340	Tombua	0.36	0.36	0.36	20733	20733	20733
AGO	Angola	4341	Virei	0.36	0.36	0.36	4953	4953	4953
AGO	Angola	4342	Alto Cauale	0.67	0.78	0.88	6163	1062	0
AGO	Angola	4343	Ambuila	0.67	0.78	0.88	1121	193	0
AGO	Angola	4344	Bembe	0.67	0.78	0.88	2988	515	0
AGO	Angola	4345	Buengas	0.67	0.78	0.88	4485	773	0
AGO	Angola	4346	Bungo	0.67	0.78	0.88	2723	469	0
AGO	Angola	4347	Damba	0.67	0.78	0.88	7701	1327	0
AGO	Angola	4348	Maquela Do Zombo	0.67	0.78	0.88	15068	2596	0
AGO	Angola	4349	Mucaba	0.67	0.78	0.88	3228	556	0
AGO	Angola	4350	Negage	0.67	0.78	0.88	13281	2288	0
AGO	Angola	4351	Puri	0.67	0.78	0.88	31136	5364	0
AGO	Angola	4352	Quimbele	0.67	0.78	0.88	9295	1601	0
AGO	Angola	4353	Quitexe	0.67	0.78	0.88	1747	301	0
AGO	Angola	4354	Santa Cruz	0.67	0.78	0.88	4600	792	0
AGO	Angola	4355	Sanza Pombo	0.67	0.78	0.88	891	153	0
AGO	Angola	4356	Songo	0.67	0.78	0.88	28511	4912	0
AGO	Angola	4357	Songo/mucaba	0.67	0.78	0.88	4640	799	0
AGO	Angola	4358	Uige	0.67	0.78	0.88	17963	3095	0
AGO	Angola	4359	Cuimba	0.36	0.36	0.36	15430	15430	15430
AGO	Angola	4360	M'banza Congo	0.36	0.36	0.36	46149	46149	46149
AGO	Angola	4361	N'zeto	0.36	0.36	0.36	24604	24604	24604
AGO	Angola	4362	Noqui	0.36	0.36	0.36	26196	26196	26196
AGO	Angola	4363	Soyo	0.36	0.36	0.36	65570	65570	65570
AGO	Angola	4364	Tomboco	0.36	0.36	0.36	22875	22875	22875
ARG	Argentina	4531	Bermejo	0.00	0.00	0.00	21489	21489	21489
ARG	Argentina	4595	Beron De Astrada	0.67	0.68	0.68	409	392	376
ARG	Argentina	4596	Capital	0.67	0.68	0.68	47135	45200	43276
ARG	Argentina	4601	Ganeral Paz	0.67	0.68	0.68	1951	1871	1792

ARG	Argentina	4602	General Alvear	0.67	0.68	0.68	1094	1049	1004
ARG	Argentina	4604	Itati	0.67	0.68	0.68	1106	1061	1015
ARG	Argentina	4605	Ituzaingo	0.67	0.68	0.68	4751	4556	4362
ARG	Argentina	4607	Mburucuy	0.67	0.68	0.68	1227	1176	1126
ARG	Argentina	4610	Paso De Los Libres	0.67	0.68	0.68	6739	6462	6187
ARG	Argentina	4612	San Cosme	0.67	0.68	0.68	2644	2536	2428
ARG	Argentina	4613	San Luis Del Palmar	0.67	0.68	0.68	2705	2594	2483
ARG	Argentina	4614	San Martin	0.67	0.68	0.68	1986	1905	1824
ARG	Argentina	4615	San Miguel	0.67	0.68	0.68	1397	1340	1283
ARG	Argentina	4617	Santo Tome	0.67	0.68	0.68	9034	8663	8294
ARG	Argentina	4636	Bermejo	0.00	0.00	0.00	12506	12506	12506
ARG	Argentina	4637	Formosa	0.00	0.00	0.00	202972	202972	202972
ARG	Argentina	4638	Laishi	0.00	0.00	0.00	15623	15623	15623
ARG	Argentina	4639	Matacos	0.00	0.00	0.00	12932	12932	12932
ARG	Argentina	4640	Patino	0.00	0.00	0.00	60522	60522	60522
ARG	Argentina	4641	Pilagas	0.00	0.00	0.00	14851	14851	14851
ARG	Argentina	4642	Pilcomayo	0.00	0.00	0.00	75757	75757	75757
ARG	Argentina	4643	Pirane	0.00	0.00	0.00	52665	52665	52665
ARG	Argentina	4644	Ramon Lista	0.00	0.00	0.00	12719	12719	12719
ARG	Argentina	4647	El Carmen	0.00	0.00	0.00	81533	81533	81533
ARG	Argentina	4649	Ledesma	0.00	0.00	0.00	68002	68002	68002
ARG	Argentina	4650	Palpala	0.00	0.00	0.00	41917	41917	41917
ARG	Argentina	4653	San Pedro	0.00	0.00	0.00	63490	63490	63490
ARG	Argentina	4654	Santa Barbara	0.00	0.00	0.00	16013	16013	16013
ARG	Argentina	4659	Valle Grande	0.00	0.00	0.00	4379	4379	4379
ARG	Argentina	4719	25 De Mayo	0.67	0.68	0.68	3724	3571	3419
ARG	Argentina	4720	Apostoles	0.67	0.68	0.68	6189	5935	5683
ARG	Argentina	4721	Cainguas	0.67	0.68	0.68	7593	7281	6971
ARG	Argentina	4722	Candelaria	0.67	0.68	0.68	4281	4106	3931
ARG	Argentina	4723	Capital	0.67	0.68	0.68	46238	44340	42452
ARG	Argentina	4724	Concepcion	0.67	0.68	0.68	1318	1264	1210
ARG	Argentina	4725	Eldorado	0.67	0.68	0.68	11228	10767	10308
ARG	Argentina	4726	General Manuel Belgrano	0.67	0.68	0.68	10248	9828	9409

ARG	Argentina	4727	Guarani	0.67	0.68	0.68	9831	9427	9026
ARG	Argentina	4728	Iguazu	0.67	0.68	0.68	12318	11813	11310
ARG	Argentina	4729	Leandro N. Alem	0.67	0.68	0.68	6378	6116	5855
ARG	Argentina	4730	Libertador General San Ma	0.67	0.68	0.68	6631	6359	6088
ARG	Argentina	4731	Montecarlo	0.67	0.68	0.68	5188	4975	4763
ARG	Argentina	4732	Obera	0.67	0.68	0.68	15162	14540	13921
ARG	Argentina	4733	San Ignacio	0.67	0.68	0.68	7949	7623	7298
ARG	Argentina	4734	San Javier	0.67	0.68	0.68	2958	2836	2716
ARG	Argentina	4735	San Pedro	0.67	0.68	0.68	4886	4686	4486
ARG	Argentina	4765	Anta	0.00	0.00	0.00	50981	50981	50981
ARG	Argentina	4773	General Jose De San Marti	0.00	0.00	0.00	138642	138642	138642
ARG	Argentina	4781	Oran	0.00	0.00	0.00	121633	121633	121633
ARG	Argentina	4783	Rivadavia	0.00	0.00	0.00	29087	29087	29087
BDI	Burundi	40559	Bubanza	0.00	0.00	0.00	90922	90922	90922
BDI	Burundi	40561	Mpanda	0.00	0.00	0.00	62116	62116	62116
BDI	Burundi	40562	Musigati	0.00	0.00	0.00	92505	92505	92505
BDI	Burundi	40563	Rugazi	0.00	0.00	0.00	65156	65156	65156
BDI	Burundi	40564	Admin unit not available	0.00	0.00	0.00	2080	2080	2080
BDI	Burundi	40565	Buterere	0.00	0.00	0.00	33645	33645	33645
BDI	Burundi	40566	Buyenzi	0.00	0.00	0.00	34941	34941	34941
BDI	Burundi	40567	Bwiza	0.00	0.00	0.00	44921	44921	44921
BDI	Burundi	40568	Cibitoke	0.00	0.00	0.00	42374	42374	42374
BDI	Burundi	40569	Gihosha	0.00	0.00	0.00	80334	80334	80334
BDI	Burundi	40570	Kamenge	0.00	0.00	0.00	60225	60225	60225
BDI	Burundi	40571	Kanyosha	0.00	0.00	0.00	92669	92669	92669
BDI	Burundi	40572	Kinama	0.00	0.00	0.00	67132	67132	67132
BDI	Burundi	40573	Kinindo	0.00	0.00	0.00	22937	22937	22937
BDI	Burundi	40574	Musaga	0.00	0.00	0.00	57765	57765	57765
BDI	Burundi	40575	Ngagara	0.00	0.00	0.00	44834	44834	44834
BDI	Burundi	40576	Nyakabiga	0.00	0.00	0.00	27046	27046	27046
BDI	Burundi	40577	Rohero	0.00	0.00	0.00	46076	46076	46076
BDI	Burundi	40578	Admin unit not available	0.00	0.00	0.00	3387	3387	3387
BDI	Burundi	40579	Admin unit not available	0.00	0.00	0.00	2749	2749	2749

BDI	Burundi	40580	Bugarama	0.00	0.00	0.00	29887	29887	29887
BDI	Burundi	40581	Isare	0.00	0.00	0.00	89725	89725	89725
BDI	Burundi	40582	Kabezi	0.00	0.00	0.00	51872	51872	51872
BDI	Burundi	40584	Mubimbi	0.00	0.00	0.00	43650	43650	43650
BDI	Burundi	40586	Muhuta	0.00	0.00	0.00	65542	65542	65542
BDI	Burundi	40589	Mutimbuzi	0.00	0.00	0.00	73931	73931	73931
BDI	Burundi	40590	Admin unit not available	0.00	0.00	0.00	5574	5574	5574
BDI	Burundi	40591	Burambi	0.00	0.00	0.00	61653	61653	61653
BDI	Burundi	40592	Bururi	0.00	0.00	0.00	93259	93259	93259
BDI	Burundi	40593	Buyengero	0.00	0.00	0.00	62649	62649	62649
BDI	Burundi	40594	Matana	0.00	0.00	0.00	47437	47437	47437
BDI	Burundi	40595	Mugamba	0.00	0.00	0.00	65932	65932	65932
BDI	Burundi	40596	Rumonge	0.00	0.00	0.00	151720	151720	151720
BDI	Burundi	40597	Rutovu	0.00	0.00	0.00	50528	50528	50528
BDI	Burundi	40598	Songa	0.00	0.00	0.00	58719	58719	58719
BDI	Burundi	40599	Vyanda	0.00	0.00	0.00	32959	32959	32959
BDI	Burundi	40600	Cankuzo	0.00	0.00	0.00	54804	54804	54804
BDI	Burundi	40601	Cendajuru	0.00	0.00	0.00	35779	35779	35779
BDI	Burundi	40602	Gisagara	0.00	0.00	0.00	60179	60179	60179
BDI	Burundi	40603	Kigamba	0.00	0.00	0.00	43114	43114	43114
BDI	Burundi	40604	Mishiha	0.00	0.00	0.00	48653	48653	48653
BDI	Burundi	40605	Buganda	0.00	0.00	0.00	74255	74255	74255
BDI	Burundi	40606	Bukinanvana	0.00	0.00	0.00	83053	83053	83053
BDI	Burundi	40607	Mabayi	0.00	0.00	0.00	74485	74485	74485
BDI	Burundi	40608	Mugina	0.00	0.00	0.00	92446	92446	92446
BDI	Burundi	40609	Murwi	0.00	0.00	0.00	90707	90707	90707
BDI	Burundi	40610	Rugombo	0.00	0.00	0.00	82213	82213	82213
BDI	Burundi	40611	Bugendana	0.00	0.00	0.00	117834	117834	117834
BDI	Burundi	40612	Bukirasazi	0.00	0.00	0.00	33872	33872	33872
BDI	Burundi	40613	Buraza	0.00	0.00	0.00	53011	53011	53011
BDI	Burundi	40614	Giheta	0.00	0.00	0.00	79970	79970	79970
BDI	Burundi	40615	Gishubi	0.00	0.00	0.00	63907	63907	63907
BDI	Burundi	40616	Gitega	0.00	0.00	0.00	178673	178673	178673

BDI	Burundi	40617	Itaba	0.00	0.00	0.00	58417	58417	58417
BDI	Burundi	40618	Makebuko	0.00	0.00	0.00	66926	66926	66926
BDI	Burundi	40619	Mutaho	0.00	0.00	0.00	69931	69931	69931
BDI	Burundi	40620	Nyarusange	0.00	0.00	0.00	117919	117919	117919
BDI	Burundi	40621	Ryansoro	0.00	0.00	0.00	38526	38526	38526
BDI	Burundi	40622	Bugenyuzi	0.00	0.00	0.00	88744	88744	88744
BDI	Burundi	40623	Buhiga	0.00	0.00	0.00	61439	61439	61439
BDI	Burundi	40624	Gihogazi	0.00	0.00	0.00	75273	75273	75273
BDI	Burundi	40625	Gitaramuka	0.00	0.00	0.00	85399	85399	85399
BDI	Burundi	40626	Mutumba	0.00	0.00	0.00	45249	45249	45249
BDI	Burundi	40627	Nyabikere	0.00	0.00	0.00	54307	54307	54307
BDI	Burundi	40628	Shombo	0.00	0.00	0.00	54082	54082	54082
BDI	Burundi	40629	Butaganzwal	0.00	0.00	0.00	56805	56805	56805
BDI	Burundi	40630	Gahombo	0.00	0.00	0.00	44324	44324	44324
BDI	Burundi	40631	Gatara	0.00	0.00	0.00	71719	71719	71719
BDI	Burundi	40632	Kabarore	0.00	0.00	0.00	62045	62045	62045
BDI	Burundi	40633	Kayanza	0.00	0.00	0.00	106240	106240	106240
BDI	Burundi	40634	Matongo	0.00	0.00	0.00	75411	75411	75411
BDI	Burundi	40635	Muhanga	0.00	0.00	0.00	70089	70089	70089
BDI	Burundi	40636	Muruta	0.00	0.00	0.00	61860	61860	61860
BDI	Burundi	40637	Rango	0.00	0.00	0.00	87782	87782	87782
BDI	Burundi	40638	Bugabira	0.00	0.00	0.00	96432	96432	96432
BDI	Burundi	40639	Busoni	0.00	0.00	0.00	153565	153565	153565
BDI	Burundi	40640	Bwambarangwe	0.00	0.00	0.00	70925	70925	70925
BDI	Burundi	40641	Gitobe	0.00	0.00	0.00	66389	66389	66389
BDI	Burundi	40642	Kirundo	0.00	0.00	0.00	104023	104023	104023
BDI	Burundi	40643	Ntega	0.00	0.00	0.00	105745	105745	105745
BDI	Burundi	40644	Vumbi	0.00	0.00	0.00	82466	82466	82466
BDI	Burundi	40645	Admin unit not available	0.00	0.00	0.00	484	484	484
BDI	Burundi	40646	Kayogoro	0.00	0.00	0.00	95926	95926	95926
BDI	Burundi	40647	Kibago	0.00	0.00	0.00	49178	49178	49178
BDI	Burundi	40648	Mabanda	0.00	0.00	0.00	48861	48861	48861
BDI	Burundi	40649	Makamba	0.00	0.00	0.00	107679	107679	107679

BDI	Burundi	40650	Nyanza Lac	0.00	0.00	0.00	45231	45231	45231
BDI	Burundi	40651	Vugizo	0.00	0.00	0.00	48992	48992	48992
BDI	Burundi	40652	Bukeye	0.00	0.00	0.00	71538	71538	71538
BDI	Burundi	40653	Kiganda	0.00	0.00	0.00	53063	53063	53063
BDI	Burundi	40654	Mbuye	0.00	0.00	0.00	59870	59870	59870
BDI	Burundi	40655	Muramviya	0.00	0.00	0.00	91170	91170	91170
BDI	Burundi	40656	Rutegama	0.00	0.00	0.00	46103	46103	46103
BDI	Burundi	40657	Buhinvuza	0.00	0.00	0.00	78896	78896	78896
BDI	Burundi	40658	Butihinda	0.00	0.00	0.00	106550	106550	106550
BDI	Burundi	40659	Gashoho	0.00	0.00	0.00	72068	72068	72068
BDI	Burundi	40660	Gasorwe	0.00	0.00	0.00	85702	85702	85702
BDI	Burundi	40661	Giteranyi	0.00	0.00	0.00	154107	154107	154107
BDI	Burundi	40662	Muyinga	0.00	0.00	0.00	155545	155545	155545
BDI	Burundi	40663	Mwakiro	0.00	0.00	0.00	47647	47647	47647
BDI	Burundi	40664	Bisoro	0.00	0.00	0.00	38641	38641	38641
BDI	Burundi	40665	Gisozi	0.00	0.00	0.00	31224	31224	31224
BDI	Burundi	40666	Kayokwe	0.00	0.00	0.00	53502	53502	53502
BDI	Burundi	40667	Ndava	0.00	0.00	0.00	62649	62649	62649
BDI	Burundi	40668	Nyabihanga	0.00	0.00	0.00	65750	65750	65750
BDI	Burundi	40669	Rusaka	0.00	0.00	0.00	49731	49731	49731
BDI	Burundi	40670	Busiga	0.00	0.00	0.00	67232	67232	67232
BDI	Burundi	40671	Gashikanwa	0.00	0.00	0.00	64747	64747	64747
BDI	Burundi	40672	Kiremba	0.00	0.00	0.00	102909	102909	102909
BDI	Burundi	40673	Marangara	0.00	0.00	0.00	72453	72453	72453
BDI	Burundi	40674	Mwumba	0.00	0.00	0.00	62797	62797	62797
BDI	Burundi	40675	Ngozi	0.00	0.00	0.00	148943	148943	148943
BDI	Burundi	40676	Nvamurenza	0.00	0.00	0.00	59754	59754	59754
BDI	Burundi	40677	Ruhororo	0.00	0.00	0.00	69604	69604	69604
BDI	Burundi	40678	Tangara	0.00	0.00	0.00	84237	84237	84237
BDI	Burundi	40679	Bukemba	0.00	0.00	0.00	39177	39177	39177
BDI	Burundi	40680	Giharo	0.00	0.00	0.00	102436	102436	102436
BDI	Burundi	40681	Gitanga	0.00	0.00	0.00	49137	49137	49137
BDI	Burundi	40682	Mpinga-Kayove	0.00	0.00	0.00	59693	59693	59693

BDI	Burundi	40683	Musongati	0.00	0.00	0.00	57345	57345	57345
BDI	Burundi	40684	Rutana	0.00	0.00	0.00	60839	60839	60839
BDI	Burundi	40685	Butaganzwa	0.00	0.00	0.00	67907	67907	67907
BDI	Burundi	40686	Butezi	0.00	0.00	0.00	43649	43649	43649
BDI	Burundi	40687	Bweru	0.00	0.00	0.00	42075	42075	42075
BDI	Burundi	40688	Gisuru	0.00	0.00	0.00	109256	109256	109256
BDI	Burundi	40689	Kinyinya	0.00	0.00	0.00	56211	56211	56211
BDI	Burundi	40690	Nyabitsinda	0.00	0.00	0.00	57386	57386	57386
BDI	Burundi	40691	Ruyigi	0.00	0.00	0.00	64801	64801	64801
BDI	Burundi	40753	Kanyosha	0.00	0.00	0.00	72611	72611	72611
BDI	Burundi	40754	Mugongomanga	0.00	0.00	0.00	32627	32627	32627
BDI	Burundi	40755	Mukike	0.00	0.00	0.00	28171	28171	28171
BDI	Burundi	40756	Mutambu	0.00	0.00	0.00	49683	49683	49683
BDI	Burundi	40757	Nyabiraba	0.00	0.00	0.00	54638	54638	54638
BDI	Burundi	48356	Gihanga	0.00	0.00	0.00	59192	59192	59192
BEN	Benin	5861	Banikoara	0.30	0.31	0.32	123063	119586	118236
BEN	Benin	5862	Gogounou	0.30	0.31	0.32	63204	61419	60725
BEN	Benin	5863	Kandi	0.30	0.31	0.32	81547	79244	78349
BEN	Benin	5864	Karimama	0.30	0.31	0.32	31384	30497	30153
BEN	Benin	5865	Malanville	0.30	0.31	0.32	86439	83997	83049
BEN	Benin	5866	Segbana	0.30	0.31	0.32	44138	42891	42407
BEN	Benin	5867	Boukoumbe	0.72	0.73	0.79	7590	6128	573
BEN	Benin	5868	Kerou	0.72	0.73	0.79	7955	6423	600
BEN	Benin	5869	Kobli	0.72	0.73	0.79	5876	4745	444
BEN	Benin	5870	Kouande	0.72	0.73	0.79	10176	8216	768
BEN	Benin	5871	Materi	0.72	0.73	0.79	10551	8519	796
BEN	Benin	5872	Natitingou	0.72	0.73	0.79	12001	9690	906
BEN	Benin	5873	Pehonko	0.72	0.73	0.79	7050	5693	532
BEN	Benin	5874	Tanguieta	0.72	0.73	0.79	7607	6142	574
BEN	Benin	5875	Toukountouna	0.72	0.73	0.79	3766	3041	284
BEN	Benin	5876	Abomey-calavi	0.30	0.31	0.32	303797	295214	291881
BEN	Benin	5877	Allada	0.30	0.31	0.32	77438	75250	74401
BEN	Benin	5878	Kpomasse	0.30	0.31	0.32	46623	45306	44795

BEN	Benin	5879	Ouidah	0.30	0.31	0.32	69387	67427	66666
BEN	Benin	5880	So-ava	0.30	0.31	0.32	57375	55754	55124
BEN	Benin	5881	Toffo	0.30	0.31	0.32	60597	58885	58221
BEN	Benin	5882	Tori-bossito	0.30	0.31	0.32	36973	35929	35523
BEN	Benin	5883	Ze	0.30	0.31	0.32	59441	57762	57110
BEN	Benin	5884	Bembereke	0.72	0.73	0.77	12998	11085	4350
BEN	Benin	5885	Kalale	0.72	0.73	0.77	13002	11088	4351
BEN	Benin	5886	Ndali	0.72	0.73	0.77	9279	7913	3105
BEN	Benin	5887	Nikki	0.72	0.73	0.77	14159	12074	4738
BEN	Benin	5888	Parakou	0.72	0.73	0.77	23719	20227	7938
BEN	Benin	5889	Perere	0.72	0.73	0.77	5573	4752	1865
BEN	Benin	5890	Sinende	0.72	0.73	0.77	8260	7044	2764
BEN	Benin	5891	Tchaourou	0.72	0.73	0.77	14954	12753	5004
BEN	Benin	5892	Bante	0.30	0.31	0.32	63323	61534	60840
BEN	Benin	5893	Dassa	0.30	0.31	0.32	76620	74456	73615
BEN	Benin	5894	Glazoue	0.30	0.31	0.32	69446	67484	66723
BEN	Benin	5895	Ouesse	0.30	0.31	0.32	74620	72511	71693
BEN	Benin	5896	Savalou	0.30	0.31	0.32	86353	83913	82966
BEN	Benin	5897	Save	0.30	0.31	0.32	63888	62083	61382
BEN	Benin	5898	Aplahoue	0.30	0.31	0.32	94942	92260	91218
BEN	Benin	5899	Djakotome	0.30	0.31	0.32	72831	70773	69974
BEN	Benin	5900	Dogbo-tota	0.30	0.31	0.32	67772	65857	65114
BEN	Benin	5901	Klouekanme	0.30	0.31	0.32	72747	70692	69894
BEN	Benin	5902	Lalo	0.30	0.31	0.32	63017	61237	60546
BEN	Benin	5903	Toviklin	0.30	0.31	0.32	47947	46593	46067
BEN	Benin	5904	Bassila	0.30	0.31	0.32	58552	56898	56256
BEN	Benin	5905	Djougou	0.30	0.31	0.32	149936	145700	144055
BEN	Benin	5906	Kopargo	0.30	0.31	0.32	37967	36895	36478
BEN	Benin	5907	Ouake	0.30	0.31	0.32	34280	33312	32936
BEN	Benin	5908	Cotonou	0.30	0.31	0.32	588402	571779	565324
BEN	Benin	5909	Athieme	0.30	0.31	0.32	29055	28234	27915
BEN	Benin	5910	Bopa	0.30	0.31	0.32	52643	51156	50579
BEN	Benin	5911	Come	0.30	0.31	0.32	48528	47157	46625

BEN	Benin	5912	Grand-popo	0.30	0.31	0.32	27688	26906	26602
BEN	Benin	5913	Houeyogbe	0.30	0.31	0.32	54021	52495	51902
BEN	Benin	5914	Lokossa	0.30	0.31	0.32	62897	61120	60430
BEN	Benin	5915	Adjara	0.30	0.31	0.32	60081	58383	57724
BEN	Benin	5916	Adjohoun	0.30	0.31	0.32	42958	41745	41274
BEN	Benin	5917	Aguegue	0.30	0.31	0.32	20927	20336	20106
BEN	Benin	5918	Akpro-misserete	0.30	0.31	0.32	55416	53851	53243
BEN	Benin	5919	Avrankou	0.30	0.31	0.32	59519	57837	57184
BEN	Benin	5920	Bonou	0.30	0.31	0.32	22495	21860	21613
BEN	Benin	5921	Dangbo	0.30	0.31	0.32	52737	51247	50669
BEN	Benin	5922	Porto-novo	0.30	0.31	0.32	220514	214284	211865
BEN	Benin	5923	Seme-kpodji	0.30	0.31	0.32	114540	111304	110048
BEN	Benin	5924	Adja-ouere	0.30	0.31	0.32	60796	59079	58412
BEN	Benin	5925	Ifangni	0.30	0.31	0.32	53150	51649	51066
BEN	Benin	5926	Ketou	0.30	0.31	0.32	78321	76108	75249
BEN	Benin	5927	Pobe	0.30	0.31	0.32	68260	66331	65583
BEN	Benin	5928	Sakete	0.30	0.31	0.32	59802	58113	57457
BEN	Benin	5929	Abomey	0.30	0.31	0.32	70483	68492	67719
BEN	Benin	5930	Agbangnizoun	0.30	0.31	0.32	40712	39562	39115
BEN	Benin	5931	Bohicon	0.30	0.31	0.32	98222	95447	94370
BEN	Benin	5932	Cove	0.30	0.31	0.32	29629	28792	28467
BEN	Benin	5933	Djidja	0.30	0.31	0.32	63351	61561	60866
BEN	Benin	5934	Ouinhi	0.30	0.31	0.32	28557	27750	27437
BEN	Benin	5935	Za-kpota	0.30	0.31	0.32	64101	62290	61587
BEN	Benin	5936	Zangnanado	0.30	0.31	0.32	27277	26507	26208
BEN	Benin	5937	Zogbodome	0.30	0.31	0.32	54451	52913	52315
BFA	Burkina Faso	12111	Bam	0.51	0.52	0.53	98036	94077	92526
BFA	Burkina Faso	12112	Banwa	0.67	0.74	0.80	44954	20806	0
BFA	Burkina Faso	12113	Bazega	0.51	0.52	0.53	83203	79843	78527
BFA	Burkina Faso	12114	Bougouriba	0.51	0.52	0.53	37358	35850	35259
BFA	Burkina Faso	12115	Boulgou	0.51	0.52	0.53	212644	204057	200693
BFA	Burkina Faso	12116	Boulkiemde	0.51	0.52	0.53	192499	184726	181680
BFA	Burkina Faso	12117	Comoe	0.58	0.63	0.69	121256	93032	63166

BFA	Burkina Faso	12118	Ganzourgou	0.51	0.52	0.53	112510	107967	106187
BFA	Burkina Faso	12119	Gnagna	0.51	0.52	0.53	143574	137776	135505
BFA	Burkina Faso	12120	Gourma	0.51	0.52	0.53	111412	106913	105150
BFA	Burkina Faso	12121	Houet	0.67	0.74	0.80	178593	82658	0
BFA	Burkina Faso	12122	Ioba	0.51	0.52	0.53	70405	67562	66448
BFA	Burkina Faso	12123	Kadiogo	0.72	0.72	0.72	237707	233512	231577
BFA	Burkina Faso	12124	Kenedougou	0.51	0.52	0.53	96410	92516	90991
BFA	Burkina Faso	12125	Komonjdjari	0.51	0.52	0.53	29514	28322	27856
BFA	Burkina Faso	12126	Kompienga	0.51	0.52	0.53	25857	24813	24404
BFA	Burkina Faso	12127	Kossi	0.67	0.74	0.80	43978	20354	0
BFA	Burkina Faso	12128	Koulpelogo	0.51	0.52	0.53	88107	84549	83156
BFA	Burkina Faso	12129	Kouritenga	0.51	0.52	0.53	127663	122508	120489
BFA	Burkina Faso	12130	Kourweogo	0.51	0.52	0.53	51531	49450	48635
BFA	Burkina Faso	12131	Leraba	0.55	0.58	0.61	38999	34117	29864
BFA	Burkina Faso	12132	Bale	0.51	0.52	0.53	80914	77647	76367
BFA	Burkina Faso	12133	Loroum	0.51	0.52	0.53	48389	46435	45669
BFA	Burkina Faso	12134	Mouhoun	0.51	0.52	0.53	115056	110409	108589
BFA	Burkina Faso	12135	Nahouri	0.51	0.52	0.53	61194	58723	57755
BFA	Burkina Faso	12136	Namentenga	0.51	0.52	0.53	114273	109658	107850
BFA	Burkina Faso	12137	Nayala	0.51	0.52	0.53	58164	55815	54895
BFA	Burkina Faso	12138	Noumbiel	0.74	0.82	0.92	5207	0	0
BFA	Burkina Faso	12139	Oubritenga	0.51	0.52	0.53	83044	79690	78376
BFA	Burkina Faso	12140	Oudalan	0.51	0.52	0.53	67546	64819	63750
BFA	Burkina Faso	12141	Passore	0.51	0.52	0.53	116499	111795	109952
BFA	Burkina Faso	12142	Poni	0.58	0.70	0.79	65215	30255	2803
BFA	Burkina Faso	12143	Sanguie	0.51	0.52	0.53	106218	101928	100248
BFA	Burkina Faso	12144	Sanmatenga	0.51	0.52	0.53	213550	204927	201548
BFA	Burkina Faso	12145	Seno	0.51	0.52	0.53	96536	92638	91111
BFA	Burkina Faso	12146	Sissili	0.51	0.52	0.53	74335	71333	70157
BFA	Burkina Faso	12147	Soum	0.51	0.52	0.53	122018	117091	115161
BFA	Burkina Faso	12148	Sourou	0.51	0.52	0.53	77965	74817	73583
BFA	Burkina Faso	12149	Tapoa	0.51	0.52	0.53	118050	113283	111415
BFA	Burkina Faso	12150	Tuy	0.51	0.52	0.53	84322	80917	79583

BFA	Burkina Faso	12151	Yagha	0.51	0.52	0.53	54185	51997	51139
BFA	Burkina Faso	12152	Yatenga	0.58	0.63	0.69	152466	116757	74433
BFA	Burkina Faso	12153	Ziro	0.51	0.52	0.53	59981	57559	56610
BFA	Burkina Faso	12154	Zondoma	0.51	0.52	0.53	59910	57491	56543
BFA	Burkina Faso	12155	Zoundweogo	0.51	0.52	0.53	86454	82963	81595
BOL	Bolivia	6141	Cercado	0.78	0.82	0.85	2718	0	0
BOL	Bolivia	6142	General Jose Ballivian	0.78	0.82	0.85	1802	0	0
BOL	Bolivia	6143	Itenez	0.78	0.82	0.85	538	0	0
BOL	Bolivia	6144	Mamore	0.78	0.82	0.85	287	0	0
BOL	Bolivia	6147	Vaca Diez	0.78	0.82	0.85	2895	0	0
BOL	Bolivia	6148	Yacuma	0.78	0.82	0.85	926	0	0
BOL	Bolivia	6174	Camacho	0.78	0.82	0.86	1309	0	0
BOL	Bolivia	6175	Franz Tamayo	0.78	0.82	0.86	800	0	0
BOL	Bolivia	6180	Abel Iturralde	0.78	0.82	0.86	644	0	0
BOL	Bolivia	6181	Larecaja	0.78	0.82	0.86	1853	0	0
BOL	Bolivia	6183	Manco Kapac	0.78	0.82	0.86	634	0	0
BOL	Bolivia	6184	Munecas	0.78	0.82	0.86	769	0	0
BOL	Bolivia	6185	Murillo	0.78	0.82	0.86	37001	0	0
BOL	Bolivia	6187	Omasuyos	0.78	0.82	0.86	2214	0	0
BOL	Bolivia	6189	Bautista Saavedra	0.78	0.82	0.86	293	0	0
BOL	Bolivia	6190	Sud Yungas	0.78	0.82	0.86	2221	0	0
BOL	Bolivia	6191	Admin unit not available	0.78	0.82	0.86	160	0	0
BOL	Bolivia	6203	General Federico Roman	0.78	0.82	0.84	252	0	0
BOL	Bolivia	6204	Madre De Dios	0.78	0.82	0.84	691	0	0
BOL	Bolivia	6205	Manuripi	0.78	0.82	0.84	459	0	0
BOL	Bolivia	6222	Angel Sandoval	0.78	0.82	0.86	557	0	0
BOL	Bolivia	6233	Velasco	0.78	0.82	0.86	1839	0	0
BOL	Bolivia	40451	Marban	0.78	0.82	0.85	1020	0	0
BOL	Bolivia	40452	Moxos	0.78	0.82	0.85	1011	0	0
BOL	Bolivia	40453	Azurduy	0.78	0.82	0.85	641	0	0
BOL	Bolivia	40454	Belisario Boeto	0.78	0.82	0.85	235	0	0
BOL	Bolivia	40455	Hernando Siles	0.78	0.82	0.85	674	0	0
BOL	Bolivia	40456	Luis Calvo	0.78	0.82	0.85	515	0	0

BOL	Bolivia	40457	Nor Cinti	0.78	0.82	0.85	1868	0	0
BOL	Bolivia	40458	Oropeza	0.78	0.82	0.85	6079	0	0
BOL	Bolivia	40459	Sud Cinti	0.78	0.82	0.85	625	0	0
BOL	Bolivia	40460	Tomina	0.78	0.82	0.85	766	0	0
BOL	Bolivia	40461	Yamparaez	0.78	0.82	0.85	837	0	0
BOL	Bolivia	40462	Zudanez	0.78	0.82	0.85	1678	0	0
BOL	Bolivia	40463	Arani	0.78	0.82	0.86	395	0	0
BOL	Bolivia	40464	Arque	0.78	0.82	0.86	476	0	0
BOL	Bolivia	40465	Ayopaya	0.78	0.82	0.86	1385	0	0
BOL	Bolivia	40466	Bolivar	0.78	0.82	0.86	177	0	0
BOL	Bolivia	40467	Campero	0.78	0.82	0.86	908	0	0
BOL	Bolivia	40468	Capinota	0.78	0.82	0.86	2776	0	0
BOL	Bolivia	40469	Carrasco	0.88	0.90	0.91	0	0	0
BOL	Bolivia	40470	Cercado	0.78	0.82	0.86	14117	0	0
BOL	Bolivia	40471	Chapare	0.78	0.82	0.86	5742	0	0
BOL	Bolivia	40472	Esteban Arce	0.78	0.82	0.86	858	0	0
BOL	Bolivia	40473	German Jordan	0.78	0.82	0.86	805	0	0
BOL	Bolivia	40474	Mizque	0.78	0.82	0.86	800	0	0
BOL	Bolivia	40475	Punata	0.78	0.82	0.86	1634	0	0
BOL	Bolivia	40476	Quillacollo	0.78	0.82	0.86	8465	0	0
BOL	Bolivia	40477	Tapacari	0.78	0.82	0.86	592	0	0
BOL	Bolivia	40478	Tiraque	0.78	0.82	0.86	892	0	0
BOL	Bolivia	40479	Aroma	0.78	0.82	0.86	2601	0	0
BOL	Bolivia	40480	Caranavi	0.78	0.82	0.86	2515	0	0
BOL	Bolivia	40481	General J.M. Pando	0.78	0.82	0.86	174	0	0
BOL	Bolivia	40482	Gualberto Villarroel	0.78	0.82	0.86	405	0	0
BOL	Bolivia	40483	Ingavi	0.78	0.82	0.86	4267	0	0
BOL	Bolivia	40484	Inquisivi	0.78	0.82	0.86	1685	0	0
BOL	Bolivia	40485	Loayza	0.78	0.82	0.86	1439	0	0
BOL	Bolivia	40486	Los Andes	0.78	0.82	0.86	1410	0	0
BOL	Bolivia	40487	Nor Yungas	0.78	0.82	0.86	2844	0	0
BOL	Bolivia	40488	Pacajes	0.78	0.82	0.86	1285	0	0
BOL	Bolivia	40489	Atahuallpa	0.78	0.82	0.84	352	0	0

BOL	Bolivia	40490	Avaroa	0.78	0.82	0.84	774	0	0
BOL	Bolivia	40491	Carangas	0.78	0.82	0.84	279	0	0
BOL	Bolivia	40492	Cercado	0.78	0.82	0.84	7779	0	0
BOL	Bolivia	40493	Ladislao Cabrera	0.78	0.82	0.84	375	0	0
BOL	Bolivia	40494	Litoral	0.78	0.82	0.84	262	0	0
BOL	Bolivia	40495	Mejillones	0.78	0.82	0.84	59	0	0
BOL	Bolivia	40496	Nor Carangas	0.78	0.82	0.84	129	0	0
BOL	Bolivia	40497	Pantaleon Dalence	0.78	0.82	0.84	910	0	0
BOL	Bolivia	40498	Poopo	0.78	0.82	0.84	440	0	0
BOL	Bolivia	40499	Sajama	0.78	0.82	0.84	233	0	0
BOL	Bolivia	40500	San Pedro de Totora	0.78	0.82	0.84	146	0	0
BOL	Bolivia	40501	Saucari	0.78	0.82	0.84	291	0	0
BOL	Bolivia	40502	Sebastian Pagador	0.78	0.82	0.84	346	0	0
BOL	Bolivia	40503	Sud Carangas	0.78	0.82	0.84	178	0	0
BOL	Bolivia	40504	Tomas Barron	0.78	0.82	0.84	123	0	0
BOL	Bolivia	40505	Abuna	0.78	0.82	0.84	174	0	0
BOL	Bolivia	40506	Nicolas Suarez	0.78	0.82	0.84	1667	0	0
BOL	Bolivia	40507	Alonso de Ibanez	0.78	0.82	0.84	646	0	0
BOL	Bolivia	40508	Antonio Quijarro	0.78	0.82	0.84	1508	0	0
BOL	Bolivia	40509	Charcas	0.78	0.82	0.84	1068	0	0
BOL	Bolivia	40510	Chayanta	0.78	0.82	0.84	2479	0	0
BOL	Bolivia	40511	Cornelio Saavedra	0.78	0.82	0.84	1602	0	0
BOL	Bolivia	40512	Daniel Campos	0.78	0.82	0.84	132	0	0
BOL	Bolivia	40513	Enrique Valdivieso	0.78	0.82	0.84	43	0	0
BOL	Bolivia	40514	General Bilbao	0.78	0.82	0.84	321	0	0
BOL	Bolivia	40515	Jose Maria Linares	0.78	0.82	0.84	1582	0	0
BOL	Bolivia	40516	Modesto Omiste	0.78	0.82	0.84	988	0	0
BOL	Bolivia	40517	Nor Chichas	0.78	0.82	0.84	1057	0	0
BOL	Bolivia	40518	Nor Lipez	0.78	0.82	0.84	373	0	0
BOL	Bolivia	40519	Rafael Bustillo	0.78	0.82	0.84	1898	0	0
BOL	Bolivia	40520	Sud Chichas	0.78	0.82	0.84	1444	0	0
BOL	Bolivia	40521	Sud Lipez	0.78	0.82	0.84	186	0	0
BOL	Bolivia	40522	Tomas Frias	0.78	0.82	0.84	4921	0	0

BOL	Bolivia	40523	Andres Ibanez	0.79	0.88	0.91	20324	0	0
BOL	Bolivia	40524	Chiquitos	0.78	0.82	0.86	1899	0	0
BOL	Bolivia	40525	Cordillera	0.78	0.91	0.95	2826	0	0
BOL	Bolivia	40526	Florida	0.78	0.82	0.86	671	0	0
BOL	Bolivia	40527	German Busch	0.78	0.82	0.86	863	0	0
BOL	Bolivia	40528	Guarayos	0.78	0.82	0.86	1392	0	0
BOL	Bolivia	40529	Ichilo	0.88	0.90	0.91	0	0	0
BOL	Bolivia	40530	Manuel Maria Caballero	0.78	0.82	0.86	642	0	0
BOL	Bolivia	40531	Nuflo De Chavez	0.78	0.82	0.86	2777	0	0
BOL	Bolivia	40532	Obispo Santiesteban	0.78	0.85	0.91	4583	0	0
BOL	Bolivia	40533	Sara (Gutierrez)	0.78	0.82	0.86	1330	0	0
BOL	Bolivia	40534	Vallegrande	0.78	0.82	0.86	618	0	0
BOL	Bolivia	40535	Warnes	0.78	0.82	0.86	2664	0	0
BOL	Bolivia	40536	Aniceto Arce	0.78	0.82	0.85	1212	0	0
BOL	Bolivia	40537	Avilez	0.78	0.82	0.85	534	0	0
BOL	Bolivia	40538	Cercado	0.78	0.82	0.85	4640	0	0
BOL	Bolivia	40539	Gran Chaco	0.78	0.82	0.85	3639	0	0
BOL	Bolivia	40540	Mendez	0.78	0.82	0.85	1034	0	0
BOL	Bolivia	40541	O'Connor	0.78	0.82	0.85	954	0	0
BRA	Brazil	6333	Acrelandia	0.35	0.80	1.00	6522	15	0
BRA	Brazil	6334	Assis Brasil	0.50	0.87	1.00	2040	0	0
BRA	Brazil	6335	Brasileia	0.39	0.82	1.00	9686	0	0
BRA	Brazil	6336	Bujari	0.32	0.78	1.00	5202	247	0
BRA	Brazil	6337	Capixaba	0.36	0.80	1.00	5225	0	0
BRA	Brazil	6338	Cruzeiro Do Sul	0.43	0.85	1.00	23478	0	0
BRA	Brazil	6339	Epitaciolandia	0.32	0.79	0.99	10647	199	0
BRA	Brazil	6340	Feijo	0.36	0.81	1.00	14268	0	0
BRA	Brazil	6341	Jordao	0.33	0.78	1.00	3964	130	0
BRA	Brazil	6342	Mancio Lima	0.36	0.76	1.00	7672	615	0
BRA	Brazil	6343	Manoel Urbano	0.32	0.76	1.00	4301	351	0
BRA	Brazil	6344	Marechal Thaumaturgo	0.32	0.75	1.00	8844	891	0
BRA	Brazil	6345	Placido De Castro	0.41	0.83	1.00	6627	0	0
BRA	Brazil	6346	Porto Acre	0.35	0.74	1.00	7946	1027	0

BRA	Brazil	6347	Porto Walter	0.34	0.78	1.00	5576	261	0
BRA	Brazil	6348	Rio Branco	0.33	0.80	1.00	184707	0	0
BRA	Brazil	6349	Rodrigues Alves	0.31	0.73	0.99	9643	1287	0
BRA	Brazil	6350	Santa Rosa Do Purus	0.33	0.79	1.00	3150	101	0
BRA	Brazil	6351	Sena Madureira	0.35	0.79	1.00	20211	430	0
BRA	Brazil	6352	Senador Guiomard	0.38	0.76	0.99	11036	987	0
BRA	Brazil	6353	Tarauaca	0.32	0.79	1.00	18660	412	0
BRA	Brazil	6354	Xapuri	0.37	0.82	1.00	8103	0	0
BRA	Brazil	6355	Agua Branca	0.08	0.40	0.55	14551	8028	4989
BRA	Brazil	6356	Anadia	0.08	0.40	0.55	12710	7013	4358
BRA	Brazil	6357	Arapiraca	0.08	0.40	0.55	171883	94832	58933
BRA	Brazil	6358	Atalaia	0.08	0.40	0.55	34513	19042	11834
BRA	Brazil	6359	Barra De Santo Antonio	0.08	0.40	0.55	11710	6461	4015
BRA	Brazil	6360	Barra De Sao Miguel	0.08	0.40	0.55	6296	3473	2159
BRA	Brazil	6361	Batalha	0.08	0.40	0.55	14100	7779	4834
BRA	Brazil	6362	Belem	1.00	1.00	1.00	0	0	0
BRA	Brazil	6363	Belo Monte	0.08	0.40	0.55	5067	2795	1737
BRA	Brazil	6364	Boca Da Mata	0.08	0.40	0.55	19771	10908	6779
BRA	Brazil	6365	Branquinha	0.08	0.40	0.55	7765	4284	2662
BRA	Brazil	6366	Cacimbinhas	0.08	0.40	0.55	7879	4347	2701
BRA	Brazil	6367	Cajueiro	0.08	0.40	0.55	15712	8668	5387
BRA	Brazil	6368	Campestre	0.41	0.81	0.95	2872	0	0
BRA	Brazil	6369	Campo Alegre	0.10	0.45	0.62	36973	18492	9573
BRA	Brazil	6370	Campo Grande	1.00	1.00	1.00	0	0	0
BRA	Brazil	6371	Canapi	0.08	0.40	0.55	13276	7325	4552
BRA	Brazil	6372	Capela	0.08	0.40	0.55	11776	6497	4038
BRA	Brazil	6373	Carneiros	0.08	0.40	0.55	6926	3821	2375
BRA	Brazil	6374	Cha Preta	0.08	0.40	0.55	5550	3062	1903
BRA	Brazil	6375	Coite Do Noia	0.08	0.40	0.55	7705	4251	2642
BRA	Brazil	6376	Colonia Leopoldina	0.08	0.40	0.55	16947	9350	5811
BRA	Brazil	6377	Coqueiro Seco	0.08	0.40	0.55	4217	2327	1446
BRA	Brazil	6378	Coruripe	0.08	0.40	0.55	45513	25111	15605
BRA	Brazil	6379	Craibas	0.08	0.40	0.55	17622	9722	6042

BRA	Brazil	6380	Delmiro Gouveia	0.08	0.40	0.55	38283	21122	13126
BRA	Brazil	6381	Dois Riachos	0.08	0.40	0.55	7934	4377	2720
BRA	Brazil	6382	Estrela De Alagoas	0.08	0.40	0.55	13347	7364	4576
BRA	Brazil	6383	Feira Grande	0.08	0.40	0.55	14566	8036	4994
BRA	Brazil	6384	Feliz Deserto	0.08	0.40	0.55	3482	1921	1194
BRA	Brazil	6385	Flexeiras	0.08	0.40	0.55	10895	6011	3736
BRA	Brazil	6386	Girau Do Ponciano	0.08	0.40	0.55	31608	17439	10837
BRA	Brazil	6387	Ibateguara	0.08	0.40	0.55	10901	6014	3738
BRA	Brazil	6388	Igaci	0.08	0.40	0.55	18781	10362	6440
BRA	Brazil	6389	Igreja Nova	0.08	0.40	0.55	18290	10091	6271
BRA	Brazil	6390	Inhapi	0.08	0.40	0.55	13202	7284	4527
BRA	Brazil	6391	Jacare Dos Homens	0.08	0.40	0.55	4028	2222	1381
BRA	Brazil	6392	Jacuipe	0.08	0.40	0.55	5027	2774	1724
BRA	Brazil	6393	Japaratinga	0.08	0.40	0.55	6297	3474	2159
BRA	Brazil	6394	Jaramataia	0.08	0.40	0.55	4065	2243	1394
BRA	Brazil	6395	Joaquim Gomes	0.08	0.40	0.55	14734	8129	5052
BRA	Brazil	6396	Jundia	0.08	0.40	0.55	3402	1877	1167
BRA	Brazil	6397	Junqueiro	0.08	0.40	0.55	16603	9161	5693
BRA	Brazil	6398	Lagoa Da Canoa	0.08	0.40	0.55	14012	7731	4804
BRA	Brazil	6399	Limoeiro De Anadia	0.08	0.40	0.55	21963	12117	7530
BRA	Brazil	6400	Maceio	0.08	0.40	0.55	749365	413443	256932
BRA	Brazil	6401	Major Isidoro	0.08	0.40	0.55	14535	8019	4984
BRA	Brazil	6402	Mar Vermelho	0.08	0.40	0.55	2298	1268	788
BRA	Brazil	6403	Maragogi	0.08	0.40	0.55	24418	13472	8372
BRA	Brazil	6404	Maravilha	0.71	0.95	0.99	789	0	0
BRA	Brazil	6405	Marechal Deodoro	0.08	0.40	0.55	39024	21531	13380
BRA	Brazil	6406	Maribondo	0.08	0.40	0.55	9437	5207	3236
BRA	Brazil	6407	Mata Grande	0.08	0.40	0.55	19765	10905	6777
BRA	Brazil	6408	Matriz De Camaragibe	0.08	0.40	0.55	19345	10673	6633
BRA	Brazil	6409	Messias	0.08	0.40	0.55	13374	7379	4585
BRA	Brazil	6410	Minador Do Negrao	0.08	0.40	0.55	3887	2145	1333
BRA	Brazil	6411	Monteiropolis	0.08	0.40	0.55	5346	2949	1833
BRA	Brazil	6412	Murici	0.08	0.40	0.55	20923	11544	7174

BRA	Brazil	6413	Novo Lino	0.08	0.40	0.55	10393	5734	3564
BRA	Brazil	6414	Olho D'agua Das Flores	0.08	0.40	0.55	15228	8402	5221
BRA	Brazil	6415	Olho D'agua Do Casado	0.08	0.40	0.55	6953	3836	2384
BRA	Brazil	6416	Olho D'agua Grande	0.08	0.40	0.55	3886	2144	1332
BRA	Brazil	6417	Olivenca	0.08	0.40	0.55	8409	4640	2883
BRA	Brazil	6418	Ouro Branco	0.73	0.91	0.95	789	0	0
BRA	Brazil	6419	Palestina	0.65	0.93	1.00	784	0	0
BRA	Brazil	6420	Palmeira Dos Indios	0.08	0.40	0.55	53310	29413	18278
BRA	Brazil	6421	Pao De Acucar	0.08	0.40	0.55	17423	9613	5974
BRA	Brazil	6422	Pariconha	0.08	0.40	0.55	7938	4379	2722
BRA	Brazil	6423	Paripueira	0.08	0.40	0.55	9840	5429	3374
BRA	Brazil	6424	Passo De Camaragibe	0.08	0.40	0.55	10363	5718	3553
BRA	Brazil	6425	Paulo Jacinto	0.08	0.40	0.55	5216	2878	1789
BRA	Brazil	6426	Penedo	0.08	0.40	0.55	47555	26238	16305
BRA	Brazil	6427	Piacabucu	0.08	0.40	0.55	12647	6978	4336
BRA	Brazil	6428	Pilar	0.08	0.40	0.55	25633	14143	8789
BRA	Brazil	6429	Pindoba	0.08	0.40	0.55	1893	1045	649
BRA	Brazil	6430	Piranhas	0.25	0.61	0.82	13004	4348	0
BRA	Brazil	6431	Poco Das Trincheiras	0.08	0.40	0.55	10440	5760	3580
BRA	Brazil	6432	Porto Calvo	0.08	0.40	0.55	18336	10116	6287
BRA	Brazil	6433	Porto De Pedras	0.08	0.40	0.55	6960	3840	2386
BRA	Brazil	6434	Porto Real Do Colegio	0.08	0.40	0.55	15239	8408	5225
BRA	Brazil	6435	Quebrangulo	0.08	0.40	0.55	8074	4454	2768
BRA	Brazil	6436	Rio Largo	0.08	0.40	0.55	51106	28196	17523
BRA	Brazil	6437	Roteiro	0.08	0.40	0.55	4725	2607	1620
BRA	Brazil	6438	Santa Luzia Do Norte	0.08	0.40	0.55	6666	3678	2286
BRA	Brazil	6439	Santana Do Ipanema	0.08	0.40	0.55	35005	19313	12002
BRA	Brazil	6440	Santana Do Mundau	0.08	0.40	0.55	7385	4075	2532
BRA	Brazil	6441	Sao Bras	0.08	0.40	0.55	5168	2851	1772
BRA	Brazil	6442	Sao Jose Da Laje	0.08	0.40	0.55	17985	9923	6166
BRA	Brazil	6443	Sao Jose Da Tapera	0.08	0.40	0.55	25499	14068	8743
BRA	Brazil	6444	Sao Luis Do Quitunde	0.08	0.40	0.55	25055	13824	8591
BRA	Brazil	6445	Sao Miguel Dos Campos	0.08	0.40	0.55	54082	29838	18543

BRA	Brazil	6446	Sao Miguel Dos Milagres	0.08	0.40	0.55	5353	2954	1835
BRA	Brazil	6447	Sao Sebastiao	0.09	0.56	0.76	24011	8181	1436
BRA	Brazil	6448	Satuba	0.08	0.40	0.55	14439	7967	4951
BRA	Brazil	6449	Senador Rui Palmeira	0.08	0.40	0.55	9833	5425	3372
BRA	Brazil	6450	Tanque D'arca	0.08	0.40	0.55	5201	2870	1783
BRA	Brazil	6451	Taquarana	0.08	0.40	0.55	15142	8354	5192
BRA	Brazil	6452	Teotonio Vilela	0.08	0.40	0.55	32160	17744	11027
BRA	Brazil	6453	Traipu	0.08	0.40	0.55	19885	10971	6818
BRA	Brazil	6454	Uniao Dos Palmares	0.08	0.40	0.55	47759	26350	16375
BRA	Brazil	6455	Vicosa	0.40	0.85	0.96	10412	0	0
BRA	Brazil	6456	Amapa	0.37	0.88	1.00	3794	0	0
BRA	Brazil	6457	Calcoene	0.37	0.86	1.00	4656	0	0
BRA	Brazil	6458	Cutias	0.41	0.86	0.98	2153	0	0
BRA	Brazil	6459	Ferreira Gomes	0.37	0.86	0.98	3293	0	0
BRA	Brazil	6460	Itaubal	0.45	0.89	0.99	1603	0	0
BRA	Brazil	6461	Laranjal Do Jari	0.45	0.94	1.00	4672	0	0
BRA	Brazil	6462	Macapa	0.37	0.88	0.99	207498	0	0
BRA	Brazil	6463	Mazagao	0.33	0.80	0.93	9904	0	0
BRA	Brazil	6464	Oiapoque	0.33	0.84	0.97	11619	0	0
BRA	Brazil	6465	Pedra Branca Do Amapari	0.31	0.83	0.96	8948	0	0
BRA	Brazil	6466	Porto Grande	0.34	0.84	0.97	9733	0	0
BRA	Brazil	6467	Pracuuba	0.31	0.80	0.94	2526	10	0
BRA	Brazil	6468	Santana	0.40	0.88	1.00	44942	0	0
BRA	Brazil	6469	Serra Do Navio	0.52	0.93	0.98	1469	0	0
BRA	Brazil	6470	Tartarugalzinho	0.34	0.83	0.97	8116	0	0
BRA	Brazil	6471	Vitoria Do Jari	0.26	0.73	0.84	21307	2854	0
BRA	Brazil	6472	Alvaraes	0.37	0.90	1.00	6676	0	0
BRA	Brazil	6473	Amatura	0.39	0.92	1.00	4552	0	0
BRA	Brazil	6474	Anama	0.35	0.86	1.00	5930	0	0
BRA	Brazil	6475	Anori	0.34	0.85	0.99	9317	0	0
BRA	Brazil	6476	Apui	0.35	0.87	0.98	9597	0	0
BRA	Brazil	6477	Atalaia Do Norte	0.34	0.86	1.00	8724	0	0
BRA	Brazil	6478	Autazes	0.36	0.89	1.00	16508	0	0

BRA	Brazil	6479	Barcelos	0.29	0.80	0.96	13947	0	0
BRA	Brazil	6480	Barreirinha	0.38	0.90	1.00	13019	0	0
BRA	Brazil	6481	Benjamin Constant	0.38	0.92	1.00	15891	0	0
BRA	Brazil	6482	Beruri	0.36	0.87	1.00	8414	0	0
BRA	Brazil	6483	Boa Vista Do Ramos	0.38	0.89	1.00	7772	0	0
BRA	Brazil	6484	Boca Do Acre	0.36	0.89	1.00	14707	0	0
BRA	Brazil	6485	Borba	0.37	0.91	1.00	17032	0	0
BRA	Brazil	6486	Caapiranga	0.33	0.84	0.99	5820	0	0
BRA	Brazil	6487	Canutama	0.32	0.83	0.99	7148	0	0
BRA	Brazil	6488	Carauari	0.36	0.90	1.00	12322	0	0
BRA	Brazil	6489	Careiro	0.32	0.87	1.00	17779	0	0
BRA	Brazil	6490	Careiro Da Varzea	0.36	0.89	1.00	12819	0	0
BRA	Brazil	6491	Coari	0.37	0.91	1.00	35196	0	0
BRA	Brazil	6492	Codajas	0.33	0.86	1.00	13476	0	0
BRA	Brazil	6493	Eirunepe	0.37	0.88	1.00	14988	0	0
BRA	Brazil	6494	Envira	0.34	0.84	0.98	10597	0	0
BRA	Brazil	6495	Fonte Boa	0.38	0.90	1.00	8337	0	0
BRA	Brazil	6496	Guajara	0.26	0.79	0.95	21855	329	0
BRA	Brazil	6497	Humaita	0.36	0.89	1.00	23102	0	0
BRA	Brazil	6498	Ipixuna	0.30	0.80	0.95	14804	0	0
BRA	Brazil	6499	Iranduba	0.38	0.90	1.00	19611	0	0
BRA	Brazil	6500	Itacoatiara	0.38	0.91	1.00	41645	0	0
BRA	Brazil	6501	Itamarati	0.36	0.89	1.00	3571	0	0
BRA	Brazil	6502	Itapiranga	0.38	0.92	1.00	3768	0	0
BRA	Brazil	6503	Japura	0.43	0.95	1.00	2412	0	0
BRA	Brazil	6504	Jurua	0.34	0.86	1.00	6596	0	0
BRA	Brazil	6505	Jutai	0.37	0.89	1.00	7180	0	0
BRA	Brazil	6506	Labrea	0.37	0.91	1.00	18961	0	0
BRA	Brazil	6507	Manacapuru	0.47	0.94	1.00	31431	0	0
BRA	Brazil	6508	Manaquiri	0.31	0.83	0.96	15189	0	0
BRA	Brazil	6509	Manaus	0.37	0.90	1.00	902850	0	0
BRA	Brazil	6510	Manicore	0.35	0.88	1.00	24321	0	0
BRA	Brazil	6511	Maraa	0.36	0.90	1.00	7975	0	0

BRA	Brazil	6512	Maues	0.37	0.90	1.00	26599	0	0
BRA	Brazil	6513	Nhamunda	0.36	0.87	1.00	9196	0	0
BRA	Brazil	6514	Nova Olinda Do Norte	0.35	0.85	1.00	16436	0	0
BRA	Brazil	6515	Novo Airao	0.34	0.85	0.99	8684	0	0
BRA	Brazil	6516	Novo Aripuana	0.37	0.88	1.00	10634	0	0
BRA	Brazil	6517	Parintins	0.36	0.88	1.00	48983	0	0
BRA	Brazil	6518	Pauini	0.36	0.87	1.00	8634	0	0
BRA	Brazil	6519	Presidente Figueiredo	0.37	0.92	1.00	14931	0	0
BRA	Brazil	6520	Rio Preto Da Eva	0.35	0.89	1.00	14390	0	0
BRA	Brazil	6521	Santa Isabel Do Rio Negro	0.32	0.85	1.00	11655	0	0
BRA	Brazil	6522	Santo Antonio Do Ica	0.38	0.91	1.00	10368	0	0
BRA	Brazil	6523	Sao Gabriel Da Cachoeira	0.40	0.92	1.00	17405	0	0
BRA	Brazil	6524	Sao Paulo De Olivenca	0.35	0.89	1.00	19099	0	0
BRA	Brazil	6525	Sao Sebastiao Do Uatuma	0.35	0.85	0.98	6069	0	0
BRA	Brazil	6526	Silves	0.38	0.91	1.00	3823	0	0
BRA	Brazil	6527	Tabatinga	0.37	0.93	1.00	25138	0	0
BRA	Brazil	6528	Tapaua	0.38	0.92	1.00	7746	0	0
BRA	Brazil	6529	Tefe	0.38	0.93	1.00	25967	0	0
BRA	Brazil	6530	Tonantins	0.37	0.90	1.00	7834	0	0
BRA	Brazil	6531	Uarini	0.37	0.91	1.00	5701	0	0
BRA	Brazil	6532	Urucara	0.39	0.91	1.00	7020	0	0
BRA	Brazil	6533	Urucurituba	0.36	0.87	1.00	9643	0	0
BRA	Brazil	6534	Abaira	0.08	0.40	0.55	6423	3544	2202
BRA	Brazil	6535	Abare	0.08	0.40	0.55	14399	7944	4937
BRA	Brazil	6536	Acajutiba	0.08	0.40	0.55	11925	6579	4089
BRA	Brazil	6537	Adustina	0.08	0.40	0.55	11986	6613	4109
BRA	Brazil	6538	Agua Fria	0.08	0.40	0.55	12419	6852	4258
BRA	Brazil	6539	Aiquara	0.08	0.40	0.55	3895	2149	1335
BRA	Brazil	6540	Alagoinhas	0.08	0.40	0.55	109421	60371	37517
BRA	Brazil	6541	Alcobaca	0.08	0.40	0.55	16025	8841	5494
BRA	Brazil	6542	Almadina	0.08	0.40	0.55	4389	2422	1505
BRA	Brazil	6543	Amargosa	0.08	0.40	0.55	26671	14715	9145
BRA	Brazil	6544	Amelia Rodrigues	0.08	0.40	0.55	26729	14747	9164

BRA	Brazil	6545	America Dourada	0.08	0.40	0.55	10947	6040	3753
BRA	Brazil	6546	Anage	0.08	0.40	0.55	16662	9193	5713
BRA	Brazil	6547	Andarai	0.08	0.40	0.55	10255	5658	3516
BRA	Brazil	6548	Andorinha	0.22	0.60	0.80	8159	2897	53
BRA	Brazil	6549	Angical	0.17	0.49	0.65	9550	4667	2278
BRA	Brazil	6550	Anguera	0.08	0.40	0.55	7732	4266	2651
BRA	Brazil	6551	Antas	0.08	0.40	0.55	13277	7325	4552
BRA	Brazil	6552	Antonio Cardoso	0.08	0.40	0.55	8498	4689	2914
BRA	Brazil	6553	Antonio Goncalves	0.08	0.40	0.55	9683	5342	3320
BRA	Brazil	6554	Apora	0.08	0.40	0.55	14028	7740	4810
BRA	Brazil	6555	Apuarema	0.08	0.40	0.55	5136	2834	1761
BRA	Brazil	6556	Aracas	0.08	0.40	0.55	8452	4663	2898
BRA	Brazil	6557	Aracatu	0.08	0.40	0.55	28011	15454	9604
BRA	Brazil	6558	Araci	0.08	0.40	0.55	40059	22101	13735
BRA	Brazil	6559	Aramari	0.08	0.40	0.55	7993	4410	2741
BRA	Brazil	6560	Arataca	0.08	0.40	0.55	7673	4233	2631
BRA	Brazil	6561	Aratuipe	0.08	0.40	0.55	6164	3401	2113
BRA	Brazil	6562	Aurelino Leal	0.24	0.62	0.83	7807	2510	0
BRA	Brazil	6563	Baianopolis	0.17	0.49	0.65	9433	4609	2251
BRA	Brazil	6564	Baixa Grande	0.08	0.40	0.55	14953	8250	5127
BRA	Brazil	6565	Banzae	0.25	0.69	0.95	6204	1235	0
BRA	Brazil	6566	Barra	0.17	0.49	0.65	35023	17113	8356
BRA	Brazil	6567	Barra Da Estiva	0.08	0.40	0.55	14941	8243	5123
BRA	Brazil	6568	Barra Do Choca	0.08	0.40	0.55	24611	13578	8438
BRA	Brazil	6569	Barra Do Mendes	0.08	0.40	0.55	11879	6554	4073
BRA	Brazil	6570	Barra Do Rocha	0.34	0.80	0.98	2336	0	0
BRA	Brazil	6571	Barreiras	0.17	0.49	0.65	157385	76905	37548
BRA	Brazil	6572	Barro Alto	0.45	0.73	0.86	4073	860	0
BRA	Brazil	6573	Barro Preto	0.08	0.40	0.55	3235	1785	1109
BRA	Brazil	6574	Belmonte	0.08	0.40	0.55	16452	9077	5641
BRA	Brazil	6575	Belo Campo	0.08	0.40	0.55	10557	5824	3619
BRA	Brazil	6576	Biritinga	0.08	0.40	0.55	11325	6248	3883
BRA	Brazil	6577	Boa Nova	0.08	0.40	0.55	10506	5797	3602

BRA	Brazil	6578	Boa Vista Do Tupim	0.23	0.62	0.85	10529	3229	0
BRA	Brazil	6579	Bom Jesus Da Lapa	0.17	0.49	0.65	43721	21364	10431
BRA	Brazil	6580	Bom Jesus Da Serra	0.08	0.40	0.55	7106	3921	2436
BRA	Brazil	6581	Boninal	0.08	0.40	0.55	9405	5189	3225
BRA	Brazil	6582	Bonito	0.32	0.84	0.99	5871	0	0
BRA	Brazil	6583	Boquira	0.17	0.49	0.65	14827	7245	3537
BRA	Brazil	6584	Botupora	0.08	0.40	0.55	7323	4040	2511
BRA	Brazil	6585	Brejoes	0.27	0.77	0.98	7343	408	0
BRA	Brazil	6586	Brejolandia	0.17	0.49	0.65	7237	3536	1727
BRA	Brazil	6587	Brotas De Macaubas	0.17	0.49	0.65	6903	3373	1647
BRA	Brazil	6588	Brumado	0.08	0.40	0.55	48790	26919	16729
BRA	Brazil	6589	Buerarema	0.25	0.71	0.95	10187	1723	0
BRA	Brazil	6590	Buritirama	0.17	0.49	0.65	13199	6450	3149
BRA	Brazil	6591	Caatiba	0.08	0.40	0.55	7960	4392	2729
BRA	Brazil	6592	Cabaceiras Do Paraguacu	0.08	0.40	0.55	12781	7051	4382
BRA	Brazil	6593	Cachoeira	0.08	0.40	0.55	26458	14598	9072
BRA	Brazil	6594	Cacule	0.08	0.40	0.55	17089	9428	5859
BRA	Brazil	6595	Caem	0.08	0.40	0.55	6993	3858	2398
BRA	Brazil	6596	Caetite	0.08	0.40	0.55	37344	20604	12804
BRA	Brazil	6597	Cafarnaum	0.08	0.40	0.55	16769	9252	5750
BRA	Brazil	6598	Cairu	0.08	0.40	0.55	12454	6871	4270
BRA	Brazil	6599	Caldeirao Grande	0.08	0.40	0.55	10529	5809	3610
BRA	Brazil	6600	Camacan	0.08	0.40	0.55	23746	13101	8142
BRA	Brazil	6601	Camacari	0.08	0.40	0.55	222557	122791	76308
BRA	Brazil	6602	Camamu	0.24	0.65	0.89	20293	5337	0
BRA	Brazil	6603	Campo Alegre De Lourdes	0.17	0.49	0.65	18463	9022	4405
BRA	Brazil	6604	Campo Formoso	0.22	0.60	0.80	39942	14085	332
BRA	Brazil	6605	Canapolis	0.56	0.88	0.99	2324	0	0
BRA	Brazil	6606	Canarana	0.30	0.71	0.95	14285	2590	0
BRA	Brazil	6607	Canavieiras	0.08	0.40	0.55	23003	12691	7887
BRA	Brazil	6608	Candeal	0.08	0.40	0.55	6242	3444	2140
BRA	Brazil	6609	Candeias	0.10	0.44	0.62	61203	30998	15823
BRA	Brazil	6610	Candiba	0.08	0.40	0.55	10093	5569	3461

BRA	Brazil	6611	Candido Sales	0.08	0.40	0.55	17721	9777	6076
BRA	Brazil	6612	Cansancao	0.08	0.40	0.55	24766	13664	8491
BRA	Brazil	6613	Canudos	0.08	0.40	0.55	12355	6816	4236
BRA	Brazil	6614	Capela Do Alto Alegre	0.08	0.40	0.55	8524	4703	2923
BRA	Brazil	6615	Capim Grosso	0.08	0.40	0.55	20973	11571	7191
BRA	Brazil	6616	Caravelas	0.08	0.40	0.55	16446	9074	5639
BRA	Brazil	6617	Cardeal Da Silva	0.23	0.61	0.83	4597	1504	0
BRA	Brazil	6618	Carinhanha	0.43	0.81	1.00	10905	0	0
BRA	Brazil	6619	Casa Nova	0.17	0.49	0.65	45668	22315	10895
BRA	Brazil	6620	Castro Alves	0.21	0.61	0.84	16281	5344	0
BRA	Brazil	6621	Catolandia	0.17	0.49	0.65	1786	873	426
BRA	Brazil	6622	Catu	0.08	0.40	0.55	40941	22588	14037
BRA	Brazil	6623	Caturama	0.08	0.40	0.55	7133	3935	2446
BRA	Brazil	6624	Central	0.17	0.49	0.65	9060	4427	2161
BRA	Brazil	6625	Chorrocho	0.08	0.40	0.55	8161	4503	2798
BRA	Brazil	6626	Cicero Dantas	0.08	0.40	0.55	26096	14398	8947
BRA	Brazil	6627	Cipo	0.08	0.40	0.55	12355	6817	4236
BRA	Brazil	6628	Coaraci	0.25	0.69	0.93	10340	2055	0
BRA	Brazil	6629	Cocos	0.17	0.49	0.65	12013	5870	2866
BRA	Brazil	6630	Conceicao Da Feira	0.08	0.40	0.55	15624	8620	5357
BRA	Brazil	6631	Conceicao Do Almeida	0.08	0.40	0.55	13758	7591	4717
BRA	Brazil	6632	Conceicao Do Coite	0.08	0.40	0.55	49440	27277	16951
BRA	Brazil	6633	Conceicao Do Jacuipe	0.08	0.40	0.55	15705	8665	5385
BRA	Brazil	6634	Conde	0.08	0.40	0.55	18562	10241	6364
BRA	Brazil	6635	Condeuba	0.08	0.40	0.55	12176	6718	4175
BRA	Brazil	6636	Contendas Do Sincora	0.08	0.40	0.55	3221	1777	1104
BRA	Brazil	6637	Coracao De Maria	0.08	0.40	0.55	17814	9828	6108
BRA	Brazil	6638	Cordeiros	0.28	0.68	0.93	4585	1039	0
BRA	Brazil	6639	Coribe	0.17	0.49	0.65	9056	4425	2161
BRA	Brazil	6640	Coronel Joao Sa	0.24	0.65	0.88	9030	2479	0
BRA	Brazil	6641	Correntina	0.36	0.78	1.00	13916	626	0
BRA	Brazil	6642	Cotegipe	0.17	0.49	0.65	9176	4484	2189
BRA	Brazil	6643	Cravolandia	0.08	0.40	0.55	3503	1932	1201

BRA	Brazil	6644	Crisopolis	0.24	0.63	0.87	11262	3448	0
BRA	Brazil	6645	Cristopolis	0.17	0.49	0.65	7520	3675	1794
BRA	Brazil	6646	Cruz Das Almas	0.08	0.40	0.55	47969	26466	16447
BRA	Brazil	6647	Curaca	0.08	0.40	0.55	24673	13613	8460
BRA	Brazil	6648	Dario Meira	0.08	0.40	0.55	7211	3979	2472
BRA	Brazil	6649	Dias D'avila	0.08	0.40	0.55	59074	32592	20254
BRA	Brazil	6650	Dom Basilio	0.08	0.40	0.55	8571	4729	2939
BRA	Brazil	6651	Dom Macedo Costa	0.08	0.40	0.55	2925	1614	1003
BRA	Brazil	6652	Elisio Medrado	0.08	0.40	0.55	6036	3330	2069
BRA	Brazil	6653	Encruzilhada	0.08	0.40	0.55	17268	9527	5921
BRA	Brazil	6654	Entre Rios	0.08	0.40	0.55	28009	15453	9603
BRA	Brazil	6655	Erico Cardoso	0.08	0.40	0.55	7561	4171	2592
BRA	Brazil	6656	Esplanada	0.08	0.40	0.55	28048	15475	9617
BRA	Brazil	6657	Euclides Da Cunha	0.08	0.40	0.55	45065	24863	15451
BRA	Brazil	6658	Eunapolis	0.08	0.40	0.55	81332	44873	27886
BRA	Brazil	6659	Fatima	0.39	0.83	0.97	6476	0	0
BRA	Brazil	6660	Feira Da Mata	0.17	0.49	0.65	4307	2105	1028
BRA	Brazil	6661	Feira De Santana	0.08	0.40	0.55	449072	247765	153972
BRA	Brazil	6662	Filadelfia	0.17	0.60	0.86	11240	3484	0
BRA	Brazil	6663	Firmino Alves	0.08	0.40	0.55	4084	2253	1400
BRA	Brazil	6664	Floresta Azul	0.24	0.65	0.91	6039	1619	0
BRA	Brazil	6665	Formosa Do Rio Preto	0.17	0.49	0.65	16147	7890	3852
BRA	Brazil	6666	Gandu	0.08	0.40	0.55	23664	13056	8113
BRA	Brazil	6667	Gaviao	0.08	0.40	0.55	2934	1619	1006
BRA	Brazil	6668	Gentio Do Ouro	0.17	0.49	0.65	7117	3478	1698
BRA	Brazil	6669	Gloria	0.08	0.40	0.55	10352	5712	3549
BRA	Brazil	6670	Gongogi	0.08	0.40	0.55	5368	2962	1841
BRA	Brazil	6671	Governador Mangabeira	0.08	0.40	0.55	14041	7747	4814
BRA	Brazil	6672	Guajeru	0.08	0.40	0.55	5319	2935	1824
BRA	Brazil	6673	Guanambi	0.08	0.40	0.55	60134	33177	20618
BRA	Brazil	6674	Guaratinga	0.17	0.49	0.65	14734	7200	3515
BRA	Brazil	6675	Heliopolis	0.08	0.40	0.55	9463	5221	3245
BRA	Brazil	6676	Iacu	0.08	0.40	0.55	17793	9817	6101

BRA	Brazil	6677	Ibiassuce	0.08	0.40	0.55	6454	3561	2213
BRA	Brazil	6678	Ibicarai	0.08	0.40	0.55	16398	9047	5622
BRA	Brazil	6679	Ibicoara	0.08	0.40	0.55	14263	7869	4890
BRA	Brazil	6680	Ibicui	0.08	0.40	0.55	12416	6850	4257
BRA	Brazil	6681	Ibipeba	0.08	0.40	0.55	13083	7218	4486
BRA	Brazil	6682	Ibipitanga	0.08	0.40	0.55	10619	5859	3641
BRA	Brazil	6683	Ibiquera	0.08	0.40	0.55	3806	2100	1305
BRA	Brazil	6684	Ibirapitanga	0.08	0.40	0.55	16906	9327	5796
BRA	Brazil	6685	Ibirapua	0.08	0.40	0.55	6271	3460	2150
BRA	Brazil	6686	Ibirataia	0.08	0.40	0.55	11917	6575	4086
BRA	Brazil	6687	Ibitiara	0.08	0.40	0.55	11534	6363	3955
BRA	Brazil	6688	Ibitita	0.20	0.56	0.74	11104	4547	1154
BRA	Brazil	6689	Ibotirama	0.17	0.49	0.65	16550	8087	3948
BRA	Brazil	6690	Ichu	0.08	0.40	0.55	4215	2325	1445
BRA	Brazil	6691	Igapora	0.08	0.40	0.55	11271	6219	3865
BRA	Brazil	6692	Igrapiuna	0.08	0.40	0.55	8999	4965	3085
BRA	Brazil	6693	Iguai	0.08	0.40	0.55	19456	10734	6671
BRA	Brazil	6694	Ilheus	0.08	0.40	0.55	124512	68696	42691
BRA	Brazil	6695	Inhambupe	0.08	0.40	0.55	30447	16798	10439
BRA	Brazil	6696	Ipecaeta	0.08	0.40	0.55	11022	6081	3779
BRA	Brazil	6697	Ipiau	0.08	0.40	0.55	29699	16386	10183
BRA	Brazil	6698	Ipira	0.08	0.41	0.57	43896	23613	13979
BRA	Brazil	6699	Ipupiara	0.17	0.49	0.65	6154	3007	1468
BRA	Brazil	6700	Irajuba	0.08	0.40	0.55	5193	2865	1781
BRA	Brazil	6701	Iramaia	0.08	0.40	0.55	7447	4109	2553
BRA	Brazil	6702	Iraquara	0.08	0.40	0.55	15541	8574	5328
BRA	Brazil	6703	Irara	0.08	0.40	0.55	20369	11238	6984
BRA	Brazil	6704	Irece	0.08	0.40	0.55	53322	29419	18282
BRA	Brazil	6705	Itabela	0.08	0.40	0.55	22035	12157	7555
BRA	Brazil	6706	Itaberaba	0.08	0.40	0.55	47173	26026	16174
BRA	Brazil	6707	Itabuna	0.08	0.40	0.55	155717	85913	53390
BRA	Brazil	6708	Itacare	0.08	0.40	0.55	20734	11439	7109
BRA	Brazil	6709	Itaete	0.08	0.40	0.55	10812	5965	3707

BRA	Brazil	6710	Itagi	0.08	0.40	0.55	9323	5144	3196
BRA	Brazil	6711	Itagiba	0.08	0.40	0.55	15090	8325	5174
BRA	Brazil	6712	Itagimirim	0.34	0.74	0.97	3256	400	0
BRA	Brazil	6713	Itaguacu Da Bahia	0.17	0.49	0.65	10748	5252	2564
BRA	Brazil	6714	Itaju Do Colonia	0.08	0.40	0.55	4621	2549	1584
BRA	Brazil	6715	Itajuipe	0.08	0.40	0.55	15206	8389	5213
BRA	Brazil	6716	Itamaraju	0.08	0.40	0.55	46386	25592	15904
BRA	Brazil	6717	Itamari	0.08	0.40	0.55	6042	3333	2072
BRA	Brazil	6718	Itambe	0.11	0.51	0.74	15414	6562	1435
BRA	Brazil	6719	Itanagra	0.08	0.40	0.55	5099	2813	1748
BRA	Brazil	6720	Itanhem	0.17	0.49	0.65	13434	6564	3205
BRA	Brazil	6721	Itaparica	0.08	0.40	0.55	13837	7634	4744
BRA	Brazil	6722	Itape	0.08	0.40	0.55	7395	4080	2535
BRA	Brazil	6723	Itapebi	0.08	0.40	0.55	7562	4172	2593
BRA	Brazil	6724	Itapetinga	0.08	0.40	0.55	55428	30581	19005
BRA	Brazil	6725	Itapicuru	0.08	0.40	0.55	25248	13930	8657
BRA	Brazil	6726	Itapitanga	0.08	0.40	0.55	7547	4164	2587
BRA	Brazil	6727	Itaquara	0.08	0.40	0.55	6100	3365	2091
BRA	Brazil	6728	Itarantim	0.08	0.40	0.55	15286	8434	5241
BRA	Brazil	6729	Itatim	0.08	0.40	0.55	11463	6325	3930
BRA	Brazil	6730	Itirucu	0.08	0.40	0.55	11873	6551	4071
BRA	Brazil	6731	Itiuba	0.08	0.40	0.55	27241	15030	9340
BRA	Brazil	6732	Itororo	0.08	0.40	0.55	15011	8282	5147
BRA	Brazil	6733	Ituacu	0.08	0.40	0.55	13966	7706	4789
BRA	Brazil	6734	Itubera	0.24	0.64	0.89	15454	4295	0
BRA	Brazil	6735	Iuiu	0.23	0.63	0.86	6002	1837	0
BRA	Brazil	6736	Jaborandi	0.27	0.76	0.95	5479	466	0
BRA	Brazil	6737	Jacaraci	0.08	0.40	0.55	10251	5656	3515
BRA	Brazil	6738	Jacobina	0.08	0.40	0.55	59868	33031	20527
BRA	Brazil	6739	Jaguaquara	0.08	0.40	0.55	39445	21763	13525
BRA	Brazil	6740	Jaguarari	0.08	0.40	0.55	24737	13648	8481
BRA	Brazil	6741	Jaguaripe	0.08	0.40	0.55	13450	7421	4612
BRA	Brazil	6742	Jandaira	0.08	0.40	0.55	8010	4419	2746

BRA	Brazil	6743	Jequie	0.08	0.40	0.55	113910	62847	39056
BRA	Brazil	6744	Jeremoabo	0.08	0.40	0.55	28959	15977	9929
BRA	Brazil	6745	Jiquirica	0.08	0.40	0.55	10454	5768	3584
BRA	Brazil	6746	Jitauna	0.08	0.40	0.55	8950	4938	3069
BRA	Brazil	6747	Joao Dourado	0.08	0.40	0.55	19348	10675	6634
BRA	Brazil	6748	Juazeiro	0.08	0.40	0.55	155435	85757	53294
BRA	Brazil	6749	Jucurucu	0.17	0.49	0.65	6628	3239	1581
BRA	Brazil	6750	Jussara	0.47	0.88	1.00	4733	0	0
BRA	Brazil	6751	Jussari	0.08	0.40	0.55	5138	2835	1762
BRA	Brazil	6752	Jussiape	0.08	0.40	0.55	4948	2730	1697
BRA	Brazil	6753	Lafaiete Coutinho	0.08	0.40	0.55	2469	1362	847
BRA	Brazil	6754	Lagoa Real	0.08	0.40	0.55	11285	6226	3869
BRA	Brazil	6755	Laje	0.08	0.40	0.55	18549	10234	6360
BRA	Brazil	6756	Lajedao	0.08	0.40	0.55	2813	1552	964
BRA	Brazil	6757	Lajedinho	0.08	0.40	0.55	2777	1532	952
BRA	Brazil	6758	Lajedo Do Tabocal	0.08	0.40	0.55	3759	2074	1289
BRA	Brazil	6759	Lamarao	0.08	0.40	0.55	7672	4233	2631
BRA	Brazil	6760	Lapao	0.08	0.40	0.55	19483	10749	6680
BRA	Brazil	6761	Lauro De Freitas	0.08	0.40	0.55	130979	72264	44908
BRA	Brazil	6762	Lencois	0.08	0.40	0.55	8266	4560	2834
BRA	Brazil	6763	Licinio De Almeida	0.08	0.40	0.55	9378	5174	3216
BRA	Brazil	6764	Livramento Do Brumado	0.23	0.70	0.92	27398	4683	0
BRA	Brazil	6765	Macajuba	0.08	0.40	0.55	8364	4614	2868
BRA	Brazil	6766	Macarani	0.08	0.40	0.55	14559	8032	4992
BRA	Brazil	6767	Macaubas	0.17	0.49	0.65	32810	16032	7828
BRA	Brazil	6768	Macurure	0.08	0.40	0.55	5737	3165	1967
BRA	Brazil	6769	Madre De Deus	0.08	0.40	0.55	15152	8360	5195
BRA	Brazil	6770	Maetinga	0.08	0.40	0.55	3102	1711	1063
BRA	Brazil	6771	Maiquinique	0.08	0.40	0.55	6032	3328	2068
BRA	Brazil	6772	Mairi	0.28	0.76	0.94	9827	734	0
BRA	Brazil	6773	Malhada	0.17	0.49	0.65	11068	5408	2641
BRA	Brazil	6774	Malhada De Pedras	0.08	0.40	0.55	5852	3229	2007
BRA	Brazil	6775	Manoel Vitorino	0.25	0.67	0.94	8403	1909	0

BRA	Brazil	6776	Mansidao	0.17	0.49	0.65	8697	4250	2075
BRA	Brazil	6777	Maracas	0.08	0.40	0.55	16450	9076	5640
BRA	Brazil	6778	Maragogipe	0.08	0.40	0.55	32612	17993	11181
BRA	Brazil	6779	Marau	1.00	1.00	1.00	0	0	0
BRA	Brazil	6780	Marcionilio Souza	0.08	0.40	0.55	7560	4171	2592
BRA	Brazil	6781	Mascote	0.08	0.40	0.55	10442	5761	3580
BRA	Brazil	6782	Mata De Sao Joao	0.08	0.40	0.55	40706	22458	13957
BRA	Brazil	6783	Matina	0.08	0.40	0.55	8931	4927	3062
BRA	Brazil	6784	Medeiros Neto	0.08	0.40	0.55	15999	8827	5486
BRA	Brazil	6785	Miguel Calmon	0.08	0.40	0.55	19216	10602	6589
BRA	Brazil	6786	Milagres	0.08	0.40	0.55	6899	3806	2365
BRA	Brazil	6787	Mirangaba	0.08	0.40	0.55	13346	7364	4576
BRA	Brazil	6788	Mirante	0.08	0.40	0.55	5941	3278	2037
BRA	Brazil	6789	Monte Santo	0.24	0.61	0.81	29891	10008	0
BRA	Brazil	6790	Morpara	0.17	0.49	0.65	5182	2532	1236
BRA	Brazil	6791	Morro Do Chapeu	0.08	0.40	0.55	27915	15402	9571
BRA	Brazil	6792	Mortugaba	0.08	0.40	0.55	9555	5272	3276
BRA	Brazil	6793	Mucuge	0.08	0.40	0.55	7008	3867	2403
BRA	Brazil	6794	Mucuri	0.08	0.40	0.55	30043	16576	10301
BRA	Brazil	6795	Mulungu Do Morro	0.08	0.40	0.55	7493	4134	2569
BRA	Brazil	6796	Mundo Novo	0.50	0.77	0.93	7989	898	0
BRA	Brazil	6797	Muniz Ferreira	0.19	0.60	0.85	4840	1579	0
BRA	Brazil	6798	Muquem De Sao Francisco	0.17	0.49	0.65	5734	2802	1368
BRA	Brazil	6799	Muritiba	0.08	0.40	0.55	24478	13505	8393
BRA	Brazil	6800	Mutuipe	0.08	0.40	0.55	16253	8967	5573
BRA	Brazil	6801	Nazare	0.10	0.47	0.66	20397	9715	4111
BRA	Brazil	6802	Nilo Pecanha	0.08	0.40	0.55	9967	5499	3418
BRA	Brazil	6803	Nordestina	0.08	0.40	0.55	9302	5132	3189
BRA	Brazil	6804	Nova Canaa	0.08	0.40	0.55	9017	4975	3092
BRA	Brazil	6805	Nova Fatima	0.29	0.71	0.96	4136	714	0
BRA	Brazil	6806	Nova Ibia	0.08	0.40	0.55	4731	2610	1622
BRA	Brazil	6807	Nova Itarana	0.08	0.40	0.55	6082	3356	2085
BRA	Brazil	6808	Nova Redencao	0.08	0.40	0.55	6400	3531	2194

BRA	Brazil	6809	Nova Soure	0.08	0.40	0.55	17276	9532	5923
BRA	Brazil	6810	Nova Vicosa	0.08	0.40	0.55	31513	17387	10805
BRA	Brazil	6811	Novo Horizonte	0.87	0.98	1.00	0	0	0
BRA	Brazil	6812	Novo Triunfo	0.08	0.40	0.55	8961	4944	3072
BRA	Brazil	6813	Olindina	0.08	0.40	0.55	18103	9988	6207
BRA	Brazil	6814	Oliveira Dos Brejinhos	0.17	0.49	0.65	14758	7211	3521
BRA	Brazil	6815	Ouricangas	0.08	0.40	0.55	6624	3655	2271
BRA	Brazil	6816	Ourolandia	0.08	0.40	0.55	10640	5871	3648
BRA	Brazil	6817	Palmas De Monte Alto	0.08	0.40	0.55	15690	8657	5380
BRA	Brazil	6818	Palmeiras	0.08	0.40	0.55	6740	3719	2311
BRA	Brazil	6819	Paramirim	0.20	0.60	0.85	13621	4480	0
BRA	Brazil	6820	Paratinga	0.17	0.49	0.65	19431	9495	4636
BRA	Brazil	6821	Paripiranga	0.08	0.40	0.55	22998	12688	7885
BRA	Brazil	6822	Pau Brasil	0.08	0.40	0.55	7345	4053	2519
BRA	Brazil	6823	Paulo Afonso	0.08	0.40	0.55	85713	47290	29388
BRA	Brazil	6824	Pe De Serra	0.08	0.40	0.55	9892	5458	3392
BRA	Brazil	6825	Pedrao	0.08	0.40	0.55	4475	2469	1534
BRA	Brazil	6826	Pedro Alexandre	0.08	0.40	0.55	13901	7670	4766
BRA	Brazil	6827	Piata	0.24	0.60	0.79	9686	3356	199
BRA	Brazil	6828	Pilao Arcado	0.17	0.49	0.65	22161	10829	5287
BRA	Brazil	6829	Pindai	0.08	0.40	0.55	11760	6488	4032
BRA	Brazil	6830	Pindobacu	0.08	0.40	0.55	14438	7966	4950
BRA	Brazil	6831	Pintadas	0.08	0.40	0.55	7352	4056	2521
BRA	Brazil	6832	Pirai Do Norte	0.08	0.40	0.55	7825	4317	2683
BRA	Brazil	6833	Piripa	0.08	0.40	0.55	8686	4793	2978
BRA	Brazil	6834	Piritiba	0.08	0.40	0.55	17751	9794	6086
BRA	Brazil	6835	Planaltino	0.08	0.40	0.55	6880	3796	2359
BRA	Brazil	6836	Planalto	0.30	0.77	0.94	14134	800	0
BRA	Brazil	6837	Pocoes	0.08	0.40	0.55	34789	19194	11928
BRA	Brazil	6838	Pojuca	0.08	0.40	0.55	24111	13302	8267
BRA	Brazil	6839	Ponto Novo	0.08	0.40	0.55	9839	5429	3374
BRA	Brazil	6840	Porto Seguro	0.08	0.40	0.55	107485	59302	36853
BRA	Brazil	6841	Potiragua	0.08	0.40	0.55	4805	2651	1647

BRA	Brazil	6842	Prado	0.08	0.40	0.55	20083	11080	6886
BRA	Brazil	6843	Presidente Dutra	0.50	0.83	0.99	4675	0	0
BRA	Brazil	6844	Presidente Janio Quadros	0.08	0.40	0.55	9354	5161	3207
BRA	Brazil	6845	Presidente Tancredo Neves	0.08	0.40	0.55	19612	10820	6724
BRA	Brazil	6846	Queimadas	0.08	0.40	0.55	19042	10506	6529
BRA	Brazil	6847	Quijingue	0.08	0.40	0.55	20039	11056	6871
BRA	Brazil	6848	Quixabeira	0.08	0.40	0.55	7187	3965	2464
BRA	Brazil	6849	Rafael Jambeiro	0.22	0.68	0.93	14454	3064	0
BRA	Brazil	6850	Remanso	0.17	0.49	0.65	26343	12872	6285
BRA	Brazil	6851	Retirolandia	0.23	0.65	0.91	8145	2101	0
BRA	Brazil	6852	Riachao Das Neves	0.17	0.49	0.65	14166	6922	3380
BRA	Brazil	6853	Riachao Do Jacuipe	0.08	0.40	0.55	25087	13841	8601
BRA	Brazil	6854	Riacho De Santana	0.17	0.49	0.65	21009	10266	5012
BRA	Brazil	6855	Ribeira Do Amparo	0.08	0.40	0.55	10434	5757	3578
BRA	Brazil	6856	Ribeira Do Pombal	0.08	0.40	0.55	37148	20495	12737
BRA	Brazil	6857	Ribeirao Do Largo	0.08	0.40	0.55	5217	2878	1789
BRA	Brazil	6858	Rio De Contas	0.08	0.40	0.55	9365	5167	3211
BRA	Brazil	6859	Rio Do Antonio	0.08	0.40	0.55	12027	6635	4124
BRA	Brazil	6860	Rio Do Pires	0.08	0.40	0.55	8448	4661	2896
BRA	Brazil	6861	Rio Real	0.08	0.40	0.55	29326	16180	10055
BRA	Brazil	6862	Rodelas	0.08	0.40	0.55	6762	3731	2318
BRA	Brazil	6863	Ruy Barbosa	0.08	0.40	0.55	22781	12569	7811
BRA	Brazil	6864	Salinas Da Margarida	0.08	0.40	0.55	10930	6030	3747
BRA	Brazil	6865	Salvador	0.08	0.40	0.55	2028637	1119250	695553
BRA	Brazil	6866	Santa Barbara	0.26	0.74	0.97	10703	1277	0
BRA	Brazil	6867	Santa Brigida	0.08	0.40	0.55	10531	5810	3611
BRA	Brazil	6868	Santa Cruz Cabralia	0.08	0.40	0.55	21084	11633	7229
BRA	Brazil	6869	Santa Cruz Da Vitoria	0.08	0.40	0.55	4534	2501	1555
BRA	Brazil	6870	Santa Ines	0.67	0.97	0.99	1425	0	0
BRA	Brazil	6871	Santa Luzia	1.00	1.00	1.00	0	0	0
BRA	Brazil	6872	Santa Maria Da Vitoria	0.30	0.72	0.98	20147	3063	0
BRA	Brazil	6873	Santa Rita De Cassia	0.17	0.49	0.65	17960	8776	4285
BRA	Brazil	6874	Santa Teresinha	0.08	0.40	0.55	7705	4251	2642

BRA	Brazil	6875	Santaluz	0.08	0.40	0.55	26391	14561	9049
BRA	Brazil	6876	Santana	0.17	0.49	0.65	17012	8313	4059
BRA	Brazil	6877	Santanopolis	0.20	0.58	0.79	5490	2014	92
BRA	Brazil	6878	Santo Amaro	0.08	0.40	0.55	44363	24476	15211
BRA	Brazil	6879	Santo Antonio De Jesus	0.08	0.40	0.55	72833	40184	24972
BRA	Brazil	6880	Santo Estevao	0.24	0.69	0.95	29885	5900	0
BRA	Brazil	6881	Sao Desiderio	0.17	0.49	0.65	21501	10506	5130
BRA	Brazil	6882	Sao Domingos	0.57	0.85	0.96	1877	0	0
BRA	Brazil	6883	Sao Felipe	0.08	0.40	0.55	14849	8193	5091
BRA	Brazil	6884	Sao Felix	0.23	0.65	0.89	6472	1727	0
BRA	Brazil	6885	Sao Felix Do Coribe	0.17	0.49	0.65	8932	4365	2131
BRA	Brazil	6886	Sao Francisco Do Conde	0.08	0.40	0.55	23284	12846	7983
BRA	Brazil	6887	Sao Gabriel	1.00	1.00	1.00	0	0	0
BRA	Brazil	6888	Sao Goncalo Dos Campos	0.08	0.40	0.55	24487	13510	8396
BRA	Brazil	6889	Sao Jose Da Vitoria	0.08	0.40	0.55	3092	1706	1060
BRA	Brazil	6890	Sao Jose Do Jacuipe	0.08	0.40	0.55	7863	4338	2696
BRA	Brazil	6891	Sao Miguel Das Matas	0.08	0.40	0.55	7835	4323	2686
BRA	Brazil	6892	Sao Sebastiao Do Passe	0.08	0.40	0.55	32208	17770	11043
BRA	Brazil	6893	Sapeacu	0.08	0.40	0.55	10357	5714	3551
BRA	Brazil	6894	Satiro Dias	0.08	0.40	0.55	12454	6871	4270
BRA	Brazil	6895	Saubara	0.08	0.40	0.55	8421	4646	2887
BRA	Brazil	6896	Saude	0.08	0.40	0.55	9090	5015	3117
BRA	Brazil	6897	Seabra	0.08	0.40	0.55	34628	19105	11873
BRA	Brazil	6898	Sebastiao Laranjeiras	0.08	0.40	0.55	8446	4660	2896
BRA	Brazil	6899	Senhor Do Bonfim	0.23	0.60	0.79	45141	15629	438
BRA	Brazil	6900	Sento Se	0.31	0.68	0.90	20435	5074	0
BRA	Brazil	6901	Serra Do Ramalho	0.33	0.74	1.00	16044	1980	0
BRA	Brazil	6902	Serra Dourada	0.17	0.49	0.65	11027	5388	2631
BRA	Brazil	6903	Serra Preta	0.08	0.40	0.55	10075	5558	3454
BRA	Brazil	6904	Serrinha	0.08	0.40	0.55	71105	39230	24380
BRA	Brazil	6905	Serrolandia	0.08	0.40	0.55	10455	5768	3585
BRA	Brazil	6906	Simoes Filho	0.20	0.62	0.87	81159	24928	0
BRA	Brazil	6907	Sitio Do Mato	0.17	0.49	0.65	7705	3765	1838

BRA	Brazil	6908	Sitio Do Quinto	0.25	0.73	0.96	6601	846	0
BRA	Brazil	6909	Sobradinho	0.47	0.72	0.92	7637	1818	0
BRA	Brazil	6910	Souto Soares	0.25	0.62	0.81	10165	3310	0
BRA	Brazil	6911	Tabocas Do Brejo Velho	0.17	0.49	0.65	8303	4057	1981
BRA	Brazil	6912	Tanhacu	0.08	0.40	0.55	14750	8138	5057
BRA	Brazil	6913	Tanque Novo	0.08	0.40	0.55	13116	7236	4497
BRA	Brazil	6914	Tanquinho	0.08	0.40	0.55	5808	3204	1991
BRA	Brazil	6915	Taperoa	0.08	0.40	0.55	15205	8389	5213
BRA	Brazil	6916	Tapiramuta	0.08	0.40	0.55	12126	6690	4158
BRA	Brazil	6917	Teixeira De Freitas	0.08	0.40	0.55	116941	64519	40095
BRA	Brazil	6918	Teodoro Sampaio	0.72	0.98	1.00	658	0	0
BRA	Brazil	6919	Teofilandia	0.08	0.40	0.55	14356	7921	4922
BRA	Brazil	6920	Teolandia	0.08	0.40	0.55	10619	5859	3641
BRA	Brazil	6921	Terra Nova	0.08	0.40	0.55	9513	5249	3262
BRA	Brazil	6922	Tremedal	0.08	0.40	0.55	13283	7329	4554
BRA	Brazil	6923	Tucano	0.08	0.40	0.55	41049	22648	14074
BRA	Brazil	6924	Uaua	0.08	0.40	0.55	17285	9536	5926
BRA	Brazil	6925	Ubaira	0.08	0.40	0.55	15350	8469	5263
BRA	Brazil	6926	Ubaitaba	0.08	0.40	0.55	16501	9104	5658
BRA	Brazil	6927	Ubata	0.08	0.40	0.55	18287	10089	6270
BRA	Brazil	6928	Uibai	0.08	0.40	0.55	10465	5774	3588
BRA	Brazil	6929	Umburanas	0.17	0.49	0.65	12274	5997	2928
BRA	Brazil	6930	Una	0.08	0.40	0.55	16464	9084	5645
BRA	Brazil	6931	Urandi	0.08	0.40	0.55	12271	6770	4207
BRA	Brazil	6932	Urucuca	0.08	0.40	0.55	14117	7789	4840
BRA	Brazil	6933	Utinga	0.08	0.40	0.55	14648	8081	5022
BRA	Brazil	6934	Valenca	0.08	0.40	0.55	70343	38810	24118
BRA	Brazil	6935	Valente	0.08	0.40	0.55	20390	11250	6991
BRA	Brazil	6936	Varzea Da Roca	0.08	0.40	0.55	10835	5978	3715
BRA	Brazil	6937	Varzea Do Poco	0.08	0.40	0.55	5959	3288	2043
BRA	Brazil	6938	Varzea Nova	0.08	0.40	0.55	9294	5128	3187
BRA	Brazil	6939	Varzedo	0.08	0.40	0.55	6068	3348	2081
BRA	Brazil	6940	Vera Cruz	0.41	0.65	0.84	17787	6960	0

BRA	Brazil	6941	Vereda	0.08	0.40	0.55	4239	2339	1453
BRA	Brazil	6942	Vitoria Da Conquista	0.08	0.40	0.55	245614	135511	84213
BRA	Brazil	6943	Wagner	0.24	0.68	0.93	5205	1096	0
BRA	Brazil	6944	Wanderley	0.17	0.49	0.65	7965	3892	1900
BRA	Brazil	6945	Wenceslau Guimaraes	0.25	0.70	0.96	12284	2281	0
BRA	Brazil	6946	Xique-xique	0.31	0.67	0.87	23356	6117	0
BRA	Brazil	6947	Abaiara	0.08	0.40	0.55	8892	4906	3049
BRA	Brazil	6948	Acarape	0.08	0.40	0.55	11310	6240	3878
BRA	Brazil	6949	Acarau	0.08	0.40	0.55	46348	25571	15891
BRA	Brazil	6950	Acopiara	0.08	0.40	0.55	40029	22085	13725
BRA	Brazil	6951	Aiuaba	0.08	0.40	0.55	12903	7119	4424
BRA	Brazil	6952	Alcantaras	0.08	0.40	0.55	8149	4496	2794
BRA	Brazil	6953	Altaneira	0.08	0.40	0.55	5661	3123	1941
BRA	Brazil	6954	Alto Santo	0.08	0.40	0.55	16531	9121	5668
BRA	Brazil	6955	Amontada	0.08	0.40	0.55	94969	52397	32562
BRA	Brazil	6956	Antonina Do Norte	0.08	0.40	0.55	5293	2920	1815
BRA	Brazil	6957	Apuiares	0.08	0.40	0.55	11138	6145	3819
BRA	Brazil	6958	Aquiraz	0.08	0.40	0.55	60858	33577	20866
BRA	Brazil	6959	Aracati	0.08	0.40	0.55	55285	30502	18955
BRA	Brazil	6960	Aracoiaba	0.08	0.40	0.55	19311	10654	6621
BRA	Brazil	6961	Ararenda	0.08	0.40	0.55	8149	4496	2794
BRA	Brazil	6962	Araripe	0.08	0.40	0.55	15715	8670	5388
BRA	Brazil	6963	Aratuba	0.08	0.40	0.55	9574	5282	3283
BRA	Brazil	6964	Arneiroz	0.08	0.40	0.55	5537	3055	1899
BRA	Brazil	6965	Assare	0.08	0.40	0.55	17680	9754	6062
BRA	Brazil	6966	Aurora	0.08	0.40	0.55	18034	9950	6183
BRA	Brazil	6967	Baixio	0.08	0.40	0.55	4524	2496	1551
BRA	Brazil	6968	Banabuiu	0.08	0.40	0.55	13661	7537	4684
BRA	Brazil	6969	Barbalha	0.08	0.40	0.55	43240	23857	14826
BRA	Brazil	6970	Barreira	0.08	0.40	0.55	15172	8371	5202
BRA	Brazil	6971	Barro	0.08	0.40	0.55	16511	9110	5661
BRA	Brazil	6972	Barroquinha	0.08	0.40	0.55	10698	5902	3668
BRA	Brazil	6973	Baturite	0.08	0.40	0.55	27414	15125	9399

BRA	Brazil	6974	Beberibe	0.08	0.40	0.55	39452	21767	13527
BRA	Brazil	6975	Bela Cruz	0.08	0.40	0.55	24229	13368	8307
BRA	Brazil	6976	Boa Viagem	0.08	0.40	0.55	38973	21502	13363
BRA	Brazil	6977	Brejo Santo	0.08	0.40	0.55	38594	21293	13233
BRA	Brazil	6978	Camocim	0.08	0.40	0.55	46511	25662	15947
BRA	Brazil	6979	Campos Sales	0.08	0.40	0.55	20238	11166	6939
BRA	Brazil	6980	Caninde	0.08	0.40	0.55	57432	31687	19692
BRA	Brazil	6981	Capistrano	0.08	0.40	0.55	12225	6745	4191
BRA	Brazil	6982	Caridade	0.08	0.40	0.55	16674	9199	5717
BRA	Brazil	6983	Carire	0.08	0.40	0.55	12999	7172	4457
BRA	Brazil	6984	Caririacu	0.08	0.40	0.55	20946	11557	7182
BRA	Brazil	6985	Carius	0.08	0.40	0.55	13857	7645	4751
BRA	Brazil	6986	Carnaubal	0.08	0.40	0.55	12672	6992	4345
BRA	Brazil	6987	Cascavel	0.92	0.99	1.00	0	0	0
BRA	Brazil	6988	Catarina	0.08	0.40	0.55	15240	8408	5225
BRA	Brazil	6989	Catunda	0.08	0.40	0.55	7771	4287	2664
BRA	Brazil	6990	Caucaia	0.08	0.40	0.55	280161	154572	96058
BRA	Brazil	6991	Cedro	0.08	0.40	0.55	18068	9968	6195
BRA	Brazil	6992	Chaval	0.08	0.40	0.55	9331	5148	3199
BRA	Brazil	6993	Choro	0.08	0.40	0.55	9512	5248	3261
BRA	Brazil	6994	Chorozinho	0.08	0.40	0.55	14876	8208	5101
BRA	Brazil	6995	Coreau	0.08	0.40	0.55	17652	9739	6052
BRA	Brazil	6996	Crateus	0.08	0.40	0.55	55655	30706	19082
BRA	Brazil	6997	Crato	0.08	0.40	0.55	100447	55419	34440
BRA	Brazil	6998	Croata	0.08	0.40	0.55	12762	7041	4376
BRA	Brazil	6999	Cruz	0.08	0.40	0.55	18316	10105	6280
BRA	Brazil	7000	Deputado Irapuan Pinheiro	0.08	0.40	0.55	7428	4098	2547
BRA	Brazil	7001	Erere	0.08	0.40	0.55	4625	2552	1586
BRA	Brazil	7002	Eusebio	0.08	0.40	0.55	42744	23583	14655
BRA	Brazil	7003	Farias Brito	0.08	0.40	0.55	12936	7137	4435
BRA	Brazil	7004	Forquilha	0.08	0.40	0.55	18293	10093	6272
BRA	Brazil	7005	Fortaleza	0.08	0.40	0.55	1927502	1063452	660877
BRA	Brazil	7006	Fortim	0.08	0.40	0.55	12119	6686	4155

BRA	Brazil	7007	Frecheirinha	0.08	0.40	0.55	9314	5139	3193
BRA	Brazil	7008	General Sampaio	0.08	0.40	0.55	5087	2806	1744
BRA	Brazil	7009	Graca	0.08	0.40	0.55	10835	5978	3715
BRA	Brazil	7010	Granja	0.08	0.40	0.55	43102	23780	14778
BRA	Brazil	7011	Granjeiro	0.08	0.40	0.55	2762	1524	947
BRA	Brazil	7012	Groairas	0.08	0.40	0.55	8243	4548	2826
BRA	Brazil	7013	Guaiuba	0.08	0.40	0.55	20127	11105	6901
BRA	Brazil	7014	Guaraciaba Do Norte	0.08	0.40	0.55	29318	16176	10052
BRA	Brazil	7015	Guaramiranga	0.08	0.40	0.55	4768	2631	1635
BRA	Brazil	7016	Hidrolandia	0.48	0.75	0.90	6698	1056	0
BRA	Brazil	7017	Horizonte	0.08	0.40	0.55	55006	30348	18860
BRA	Brazil	7018	Ibaretama	0.08	0.40	0.55	9405	5189	3225
BRA	Brazil	7019	Ibiapina	0.08	0.40	0.55	18816	10381	6451
BRA	Brazil	7020	Ibicuitinga	0.08	0.40	0.55	8765	4836	3005
BRA	Brazil	7021	Icapui	0.08	0.40	0.55	14735	8130	5052
BRA	Brazil	7022	Ico	0.08	0.40	0.55	52373	28896	17957
BRA	Brazil	7023	Iguatu	0.08	0.41	0.57	75946	41030	24386
BRA	Brazil	7024	Independencia	0.19	0.53	0.77	16055	7006	715
BRA	Brazil	7025	Ipaporanga	0.08	0.40	0.55	8364	4615	2868
BRA	Brazil	7026	Ipaumirim	0.08	0.40	0.55	9606	5300	3294
BRA	Brazil	7027	Ipu	0.08	0.40	0.55	29748	16413	10200
BRA	Brazil	7028	Ipueiras	0.09	0.43	0.59	27596	14456	8045
BRA	Brazil	7029	Iracema	0.20	0.68	0.94	8793	1773	0
BRA	Brazil	7030	Iraucuba	0.08	0.40	0.55	15247	8412	5228
BRA	Brazil	7031	Itaicaba	0.08	0.40	0.55	5633	3108	1931
BRA	Brazil	7032	Itaitinga	0.08	0.40	0.55	31377	17311	10758
BRA	Brazil	7033	Itapage	0.08	0.40	0.55	37462	20669	12844
BRA	Brazil	7034	Itapipoca	0.08	0.40	0.55	32451	17904	11126
BRA	Brazil	7035	Itapiuna	0.08	0.40	0.55	14837	8186	5087
BRA	Brazil	7036	Itarema	0.08	0.40	0.55	31140	17181	10677
BRA	Brazil	7037	Itatira	0.08	0.40	0.55	15116	8340	5183
BRA	Brazil	7038	Jaguaretama	0.08	0.40	0.55	14177	7822	4861
BRA	Brazil	7039	Jaguaribara	0.08	0.40	0.55	2185	1206	749

BRA	Brazil	7040	Jaguaribe	0.08	0.40	0.55	25142	13872	8620
BRA	Brazil	7041	Jaguaruana	0.08	0.40	0.55	23967	13223	8217
BRA	Brazil	7042	Jardim	0.62	0.84	0.96	4534	0	0
BRA	Brazil	7043	Jati	0.08	0.40	0.55	4994	2755	1712
BRA	Brazil	7044	Jijoca De Jericoacoara	0.08	0.40	0.55	13888	7662	4762
BRA	Brazil	7045	Juazeiro Do Norte	0.08	0.40	0.55	200029	110361	68583
BRA	Brazil	7046	Jucas	0.08	0.40	0.55	18309	10101	6278
BRA	Brazil	7047	Lavras Da Mangabeira	0.08	0.40	0.55	23519	12976	8064
BRA	Brazil	7048	Limoeiro Do Norte	0.08	0.40	0.55	48680	26858	16691
BRA	Brazil	7049	Madalena	0.08	0.40	0.55	15174	8372	5203
BRA	Brazil	7050	Maracanau	0.08	0.40	0.55	159281	87879	54612
BRA	Brazil	7051	Maranguape	0.08	0.40	0.55	96849	53434	33206
BRA	Brazil	7052	Marco	0.08	0.40	0.55	19913	10987	6828
BRA	Brazil	7053	Martinopole	0.08	0.40	0.55	7967	4396	2732
BRA	Brazil	7054	Massape	0.08	0.40	0.55	27928	15409	9576
BRA	Brazil	7055	Mauriti	0.08	0.40	0.55	33173	18302	11374
BRA	Brazil	7056	Meruoca	0.08	0.40	0.55	10918	6024	3743
BRA	Brazil	7057	Milagres	0.08	0.40	0.55	21886	12075	7504
BRA	Brazil	7058	Milha	0.08	0.40	0.55	9838	5428	3373
BRA	Brazil	7059	Miraima	0.08	0.40	0.55	11674	6441	4003
BRA	Brazil	7060	Missao Velha	0.08	0.40	0.55	24435	13481	8378
BRA	Brazil	7061	Mombaca	0.08	0.40	0.55	32433	17894	11120
BRA	Brazil	7062	Monsenhor Tabosa	0.08	0.40	0.55	12275	6772	4209
BRA	Brazil	7063	Morada Nova	0.08	0.40	0.55	46772	25805	16036
BRA	Brazil	7064	Moraujo	0.08	0.40	0.55	6838	3773	2344
BRA	Brazil	7065	Morrinhos	0.90	0.92	0.94	0	0	0
BRA	Brazil	7066	Mucambo	0.08	0.40	0.55	11859	6543	4066
BRA	Brazil	7067	Mulungu	0.08	0.40	0.55	6423	3544	2202
BRA	Brazil	7068	Nova Olinda	0.26	0.64	0.90	8693	2509	0
BRA	Brazil	7069	Nova Russas	0.08	0.40	0.55	23823	13144	8168
BRA	Brazil	7070	Novo Oriente	0.08	0.40	0.55	20938	11552	7179
BRA	Brazil	7071	Ocara	0.08	0.40	0.55	18962	10462	6502
BRA	Brazil	7072	Oros	0.08	0.40	0.55	15733	8680	5394

BRA	Brazil	7073	Pacajus	0.08	0.40	0.55	52561	28999	18021
BRA	Brazil	7074	Pacatuba	0.08	0.40	0.55	65414	36091	22428
BRA	Brazil	7075	Pacoti	0.08	0.40	0.55	8124	4482	2785
BRA	Brazil	7076	Pacuja	0.08	0.40	0.55	4404	2430	1510
BRA	Brazil	7077	Palhano	0.08	0.40	0.55	6740	3718	2311
BRA	Brazil	7078	Palmacia	0.08	0.40	0.55	11178	6167	3833
BRA	Brazil	7079	Paracuru	0.08	0.40	0.55	24518	13527	8407
BRA	Brazil	7080	Paraipaba	0.08	0.40	0.55	24781	13672	8497
BRA	Brazil	7081	Parambu	0.08	0.40	0.55	23589	13015	8088
BRA	Brazil	7082	Paramoti	0.08	0.40	0.55	8782	4845	3011
BRA	Brazil	7083	Pedra Branca	0.08	0.40	0.55	31391	17319	10763
BRA	Brazil	7084	Penaforte	0.08	0.40	0.55	7378	4070	2530
BRA	Brazil	7085	Pentecoste	0.08	0.40	0.55	28244	15583	9684
BRA	Brazil	7086	Pereiro	0.08	0.40	0.55	10496	5791	3599
BRA	Brazil	7087	Pindoretama	0.08	0.40	0.55	15025	8290	5152
BRA	Brazil	7088	Piquet Carneiro	0.08	0.40	0.55	12218	6741	4189
BRA	Brazil	7089	Pires Ferreira	0.08	0.40	0.55	8517	4699	2920
BRA	Brazil	7090	Poranga	0.08	0.40	0.55	7745	4273	2655
BRA	Brazil	7091	Porteiras	0.08	0.40	0.55	9826	5421	3369
BRA	Brazil	7092	Potengi	0.08	0.40	0.55	7992	4409	2740
BRA	Brazil	7093	Potiretama	0.08	0.40	0.55	5023	2771	1722
BRA	Brazil	7094	Quiterianopolis	0.08	0.40	0.55	15407	8501	5283
BRA	Brazil	7095	Quixada	0.08	0.40	0.55	65051	35890	22304
BRA	Brazil	7096	Quixelo	0.08	0.40	0.55	10928	6029	3747
BRA	Brazil	7097	Quixeramobim	0.08	0.40	0.55	59419	32783	20373
BRA	Brazil	7098	Quixere	0.08	0.40	0.55	15548	8578	5331
BRA	Brazil	7099	Redencao	0.69	0.88	0.98	3279	0	0
BRA	Brazil	7100	Reriutaba	0.08	0.40	0.55	13678	7547	4690
BRA	Brazil	7101	Russas	0.08	0.40	0.55	54719	30190	18761
BRA	Brazil	7102	Saboeiro	0.08	0.40	0.55	11656	6431	3996
BRA	Brazil	7103	Salitre	0.08	0.40	0.55	12016	6630	4120
BRA	Brazil	7104	Santa Quiteria	0.08	0.40	0.55	32348	17847	11091
BRA	Brazil	7105	Santana Do Acarau	0.08	0.40	0.55	24949	13765	8554

BRA	Brazil	7106	Santana Do Cariri	0.08	0.40	0.55	12524	6910	4294
BRA	Brazil	7107	Sao Benedito	0.08	0.40	0.55	34806	19203	11934
BRA	Brazil	7108	Sao Goncalo Do Amarante	0.08	0.40	0.55	36142	19940	12392
BRA	Brazil	7109	Sao Joao Do Jaguaribe	0.08	0.40	0.55	5661	3123	1941
BRA	Brazil	7110	Sao Luis Do Curu	0.08	0.40	0.55	9503	5243	3258
BRA	Brazil	7111	Senador Pompeu	0.08	0.40	0.55	20060	11068	6878
BRA	Brazil	7112	Senador Sa	0.08	0.40	0.55	5851	3228	2006
BRA	Brazil	7113	Sobral	0.08	0.40	0.55	153713	84808	52703
BRA	Brazil	7114	Solonopole	0.08	0.40	0.55	12961	7151	4444
BRA	Brazil	7115	Tabuleiro Do Norte	0.08	0.40	0.55	22553	12443	7733
BRA	Brazil	7116	Tamboril	0.08	0.40	0.55	19231	10610	6594
BRA	Brazil	7117	Tarrafas	0.08	0.40	0.55	6617	3651	2269
BRA	Brazil	7118	Taua	0.08	0.40	0.55	42385	23385	14532
BRA	Brazil	7119	Tejucuoca	0.08	0.40	0.55	14612	8062	5010
BRA	Brazil	7120	Tiangua	0.08	0.40	0.55	52422	28922	17974
BRA	Brazil	7121	Trairi	0.08	0.40	0.55	40723	22468	13963
BRA	Brazil	7122	Tururu	0.08	0.40	0.55	11095	6122	3804
BRA	Brazil	7123	Ubajara	0.08	0.40	0.55	24874	13724	8529
BRA	Brazil	7124	Umari	0.08	0.40	0.55	5692	3140	1952
BRA	Brazil	7125	Umirim	0.08	0.40	0.55	15494	8548	5312
BRA	Brazil	7126	Uruburetama	0.08	0.40	0.55	17974	9917	6163
BRA	Brazil	7127	Uruoca	0.08	0.40	0.55	10360	5716	3552
BRA	Brazil	7128	Varjota	0.08	0.40	0.55	14211	7840	4872
BRA	Brazil	7129	Varzea Alegre	0.08	0.40	0.55	29671	16370	10173
BRA	Brazil	7130	Vicosa Do Ceara	0.08	0.40	0.55	48535	26778	16641
BRA	Brazil	7131	Brasilia	0.67	0.92	0.99	396666	0	0
BRA	Brazil	7132	Afonso Claudio	0.17	0.49	0.65	19918	9733	4752
BRA	Brazil	7133	Agua Doce Do Norte	0.17	0.49	0.65	7384	3608	1762
BRA	Brazil	7134	Aguia Branca	0.08	0.40	0.55	7022	3874	2408
BRA	Brazil	7135	Alegre	0.08	0.40	0.55	22661	12503	7770
BRA	Brazil	7136	Alfredo Chaves	0.08	0.40	0.55	10646	5874	3650
BRA	Brazil	7137	Alto Rio Novo	0.17	0.49	0.65	4866	2378	1161
BRA	Brazil	7138	Anchieta	0.08	0.40	0.55	20326	11215	6969

BRA	Brazil	7139	Apiaca	0.08	0.40	0.55	5563	3069	1907
BRA	Brazil	7140	Aracruz	0.08	0.40	0.55	68511	37799	23490
BRA	Brazil	7141	Atilio Vivacqua	0.08	0.40	0.55	8020	4425	2750
BRA	Brazil	7142	Baixo Guandu	0.17	0.49	0.65	19336	9448	4613
BRA	Brazil	7143	Barra De Sao Francisco	0.08	0.40	0.55	30953	17077	10613
BRA	Brazil	7144	Boa Esperanca	0.55	0.90	0.98	3691	0	0
BRA	Brazil	7145	Bom Jesus Do Norte	0.08	0.40	0.55	7741	4271	2654
BRA	Brazil	7146	Brejetuba	0.17	0.49	0.65	7701	3763	1837
BRA	Brazil	7147	Cachoeiro De Itapemirim	0.08	0.40	0.55	147800	81545	50676
BRA	Brazil	7148	Cariacica	0.08	0.40	0.55	275218	151845	94363
BRA	Brazil	7149	Castelo	0.08	0.40	0.55	26799	14786	9189
BRA	Brazil	7150	Colatina	0.08	0.40	0.55	95441	52657	32724
BRA	Brazil	7151	Conceicao Da Barra	0.08	0.40	0.55	22384	12350	7675
BRA	Brazil	7152	Conceicao Do Castelo	0.08	0.40	0.55	9096	5019	3119
BRA	Brazil	7153	Divino De Sao Lourenco	0.17	0.49	0.65	2898	1416	691
BRA	Brazil	7154	Domingos Martins	0.08	0.40	0.55	24361	13441	8353
BRA	Brazil	7155	Dores Do Rio Preto	0.17	0.49	0.65	3802	1858	907
BRA	Brazil	7156	Ecoporanga	0.17	0.49	0.65	15067	7362	3595
BRA	Brazil	7157	Fundao	0.08	0.40	0.55	14529	8016	4982
BRA	Brazil	7158	Guacui	0.17	0.49	0.65	18939	9254	4518
BRA	Brazil	7159	Guarapari	0.08	0.40	0.55	83633	46142	28675
BRA	Brazil	7160	Ibatiba	0.17	0.49	0.65	15377	7514	3669
BRA	Brazil	7161	Ibiracu	0.08	0.40	0.55	8753	4829	3001
BRA	Brazil	7162	Ibitirama	0.17	0.49	0.65	5652	2762	1348
BRA	Brazil	7163	Iconha	0.08	0.40	0.55	9685	5343	3321
BRA	Brazil	7164	Irupi	0.17	0.49	0.65	8530	4168	2035
BRA	Brazil	7165	Itaguacu	0.08	0.40	0.55	10416	5747	3571
BRA	Brazil	7167	Itarana	0.08	0.40	0.55	7928	4374	2718
BRA	Brazil	7168	Iuna	0.17	0.49	0.65	17558	8579	4189
BRA	Brazil	7169	Jaguare	0.08	0.40	0.55	20929	11547	7176
BRA	Brazil	7170	Jeronimo Monteiro	0.08	0.40	0.55	8348	4606	2862
BRA	Brazil	7171	Joao Neiva	0.08	0.40	0.55	11829	6526	4056
BRA	Brazil	7172	Laranja Da Terra	0.17	0.49	0.65	6890	3366	1644

BRA	Brazil	7173	Linhares	0.08	0.40	0.55	117917	65058	40430
BRA	Brazil	7174	Mantenopolis	0.17	0.49	0.65	9317	4553	2223
BRA	Brazil	7175	Marataizes	0.08	0.40	0.55	26293	14506	9015
BRA	Brazil	7176	Marechal Floriano	0.08	0.40	0.55	11316	6243	3880
BRA	Brazil	7177	Marilandia	0.08	0.40	0.55	8631	4762	2959
BRA	Brazil	7178	Mimoso Do Sul	0.08	0.40	0.55	19293	10645	6615
BRA	Brazil	7179	Montanha	0.17	0.49	0.65	11872	5801	2832
BRA	Brazil	7180	Mucurici	0.17	0.49	0.65	3710	1813	885
BRA	Brazil	7181	Muniz Freire	0.17	0.49	0.65	11681	5708	2787
BRA	Brazil	7182	Muqui	0.08	0.40	0.55	10802	5960	3704
BRA	Brazil	7183	Nova Venecia	0.08	0.40	0.55	35274	19461	12094
BRA	Brazil	7184	Pancas	0.08	0.40	0.55	16392	9044	5620
BRA	Brazil	7185	Pedro Canario	0.08	0.40	0.55	17628	9726	6044
BRA	Brazil	7186	Pinheiros	0.08	0.40	0.55	19203	10595	6584
BRA	Brazil	7187	Piuma	0.08	0.40	0.55	14646	8081	5022
BRA	Brazil	7188	Ponto Belo	0.08	0.40	0.55	5528	3050	1895
BRA	Brazil	7189	Presidente Kennedy	0.15	0.56	0.82	7466	2768	0
BRA	Brazil	7190	Rio Bananal	0.08	0.40	0.55	13958	7701	4786
BRA	Brazil	7191	Rio Novo Do Sul	0.08	0.40	0.55	8473	4675	2905
BRA	Brazil	7192	Santa Leopoldina	0.08	0.40	0.55	9007	4969	3088
BRA	Brazil	7193	Santa Maria De Jetiba	0.08	0.40	0.55	27548	15199	9445
BRA	Brazil	7194	Santa Teresa	0.08	0.40	0.55	16994	9376	5827
BRA	Brazil	7195	Sao Domingos Do Norte	0.08	0.40	0.55	6334	3495	2172
BRA	Brazil	7196	Sao Gabriel Da Palha	0.08	0.40	0.55	25734	14198	8823
BRA	Brazil	7197	Sao Jose Do Calcado	0.08	0.40	0.55	8006	4417	2745
BRA	Brazil	7198	Sao Mateus	0.08	0.40	0.55	88275	48704	30267
BRA	Brazil	7199	Sao Roque Do Canaa	0.08	0.40	0.55	8680	4789	2976
BRA	Brazil	7200	Serra	0.08	0.40	0.55	339824	187489	116514
BRA	Brazil	7201	Sooretama	0.08	0.40	0.55	20099	11089	6891
BRA	Brazil	7202	Vargem Alta	0.08	0.40	0.55	14643	8079	5021
BRA	Brazil	7203	Venda Nova Do Imigrante	0.08	0.40	0.55	16904	9326	5796
BRA	Brazil	7204	Viana	0.25	0.67	0.93	39650	9479	0
BRA	Brazil	7205	Vila Pavao	0.08	0.40	0.55	6611	3648	2267

BRA	Brazil	7206	Vila Valerio	0.08	0.40	0.55	10232	5645	3508
BRA	Brazil	7207	Vila Velha	0.08	0.40	0.55	329720	181915	113050
BRA	Brazil	7208	Vitoria	0.08	0.40	0.55	242277	133670	83069
BRA	Brazil	7209	Abadia De Goias	0.71	0.93	0.98	727	0	0
BRA	Brazil	7210	Abadiania	0.53	0.85	0.95	5251	0	0
BRA	Brazil	7211	Acreuna	0.60	0.91	0.98	4353	0	0
BRA	Brazil	7212	Adelandia	0.33	0.84	0.97	1165	0	0
BRA	Brazil	7213	Agua Fria De Goias	0.69	0.91	0.96	639	0	0
BRA	Brazil	7214	Agua Limpa	0.88	0.94	0.96	0	0	0
BRA	Brazil	7215	Aguas Lindas De Goias	0.51	0.89	0.99	59165	0	0
BRA	Brazil	7216	Alexania	0.79	0.95	1.00	139	0	0
BRA	Brazil	7217	Aloandia	0.79	0.93	0.96	21	0	0
BRA	Brazil	7218	Alto Horizonte	0.32	0.84	0.96	2927	0	0
BRA	Brazil	7219	Alto Paraiso De Goias	0.63	0.97	0.99	1309	0	0
BRA	Brazil	7220	Alvorada Do Norte	0.77	0.95	0.99	278	0	0
BRA	Brazil	7221	Amaralina	0.30	0.78	0.91	1939	61	0
BRA	Brazil	7222	Americano Do Brasil	0.78	0.93	0.97	106	0	0
BRA	Brazil	7223	Amorinopolis	0.43	0.87	0.96	1269	0	0
BRA	Brazil	7224	Anapolis	0.70	0.92	0.99	36947	0	0
BRA	Brazil	7225	Anhanguera	0.95	0.97	0.98	0	0	0
BRA	Brazil	7226	Anicuns	0.45	0.86	0.96	7682	0	0
BRA	Brazil	7227	Aparecida De Goiania	0.64	0.94	1.00	87245	0	0
BRA	Brazil	7228	Aparecida Do Rio Doce	0.92	0.98	1.00	0	0	0
BRA	Brazil	7229	Apore	0.61	0.93	0.99	909	0	0
BRA	Brazil	7230	Aracu	0.60	0.93	0.99	779	0	0
BRA	Brazil	7231	Aragarcas	0.65	0.90	0.98	3000	0	0
BRA	Brazil	7232	Aragoiania	0.50	0.87	0.95	2929	0	0
BRA	Brazil	7233	Araguapaz	0.83	0.94	0.97	0	0	0
BRA	Brazil	7234	Arenopolis	0.62	0.92	0.98	539	0	0
BRA	Brazil	7235	Aruana	0.34	0.86	0.98	4208	0	0
BRA	Brazil	7236	Aurilandia	0.59	0.90	0.97	705	0	0
BRA	Brazil	7237	Avelinopolis	0.83	0.94	0.99	0	0	0
BRA	Brazil	7238	Baliza	0.29	0.76	0.89	2478	205	0

BRA	Brazil	7239	Barro Alto	0.21	0.66	0.75	6420	1568	507
BRA	Brazil	7240	Bela Vista De Goias	0.52	0.89	0.98	8162	0	0
BRA	Brazil	7241	Bom Jardim De Goias	0.62	0.88	0.97	1544	0	0
BRA	Brazil	7242	Bom Jesus De Goias	0.68	0.93	0.99	2524	0	0
BRA	Brazil	7243	Bonfinopolis	0.55	0.88	0.98	2276	0	0
BRA	Brazil	7244	Bonopolis	0.48	0.90	0.98	1333	0	0
BRA	Brazil	7245	Brazabrantes	0.36	0.84	0.96	1503	0	0
BRA	Brazil	7246	Britania	0.47	0.91	0.99	1958	0	0
BRA	Brazil	7247	Buriti Alegre	0.54	0.88	0.99	2505	0	0
BRA	Brazil	7248	Buriti De Goias	0.56	0.91	0.98	620	0	0
BRA	Brazil	7249	Buritinopolis	0.49	0.90	0.98	1100	0	0
BRA	Brazil	7250	Cabeceiras	0.52	0.90	0.99	2188	0	0
BRA	Brazil	7251	Cachoeira Alta	0.47	0.89	0.96	4162	0	0
BRA	Brazil	7252	Cachoeira De Goias	0.40	0.88	0.97	575	0	0
BRA	Brazil	7253	Cachoeira Dourada	0.80	0.96	0.99	16	0	0
BRA	Brazil	7254	Cacu	0.50	0.88	0.95	4563	0	0
BRA	Brazil	7255	Caiaponia	0.55	0.88	0.96	4603	0	0
BRA	Brazil	7256	Caldas Novas	0.66	0.91	0.98	12052	0	0
BRA	Brazil	7257	Caldazinha	0.45	0.86	0.97	1392	0	0
BRA	Brazil	7258	Campestre De Goias	0.56	0.91	0.98	869	0	0
BRA	Brazil	7259	Campinacu	0.31	0.79	0.92	1839	42	0
BRA	Brazil	7260	Campinorte	0.48	0.86	0.97	4553	0	0
BRA	Brazil	7261	Campo Alegre De Goias	0.59	0.88	0.96	1555	0	0
BRA	Brazil	7262	Campos Belos	0.75	0.95	1.00	990	0	0
BRA	Brazil	7263	Campos Verdes	0.78	0.97	1.00	74	0	0
BRA	Brazil	7264	Carmo Do Rio Verde	0.46	0.89	0.97	3485	0	0
BRA	Brazil	7265	Castelandia	0.77	0.93	0.98	123	0	0
BRA	Brazil	7266	Catalao	0.63	0.92	0.99	17832	0	0
BRA	Brazil	7267	Caturai	0.56	0.90	0.99	1156	0	0
BRA	Brazil	7268	Cavalcante	0.64	0.93	1.00	1535	0	0
BRA	Brazil	7269	Ceres	0.62	0.88	0.95	4735	0	0
BRA	Brazil	7270	Cezarina	0.74	0.93	0.98	507	0	0
BRA	Brazil	7271	Chapadao Do Ceu	0.50	0.90	0.98	2961	0	0

BRA	Brazil	7272	Cidade Ocidental	0.58	0.91	1.00	14562	0	0
BRA	Brazil	7273	Cocalzinho De Goias	0.57	0.91	0.99	4597	0	0
BRA	Brazil	7274	Colinas Do Sul	0.71	0.95	1.00	324	0	0
BRA	Brazil	7275	Corrego Do Ouro	0.42	0.88	0.97	955	0	0
BRA	Brazil	7276	Corumba De Goias	0.54	0.85	0.95	2909	0	0
BRA	Brazil	7277	Corumbaiba	0.77	0.91	0.95	242	0	0
BRA	Brazil	7278	Cristalina	0.55	0.91	0.99	13847	0	0
BRA	Brazil	7279	Cristianopolis	0.70	0.92	0.97	294	0	0
BRA	Brazil	7280	Crixas	0.62	0.91	0.98	3143	0	0
BRA	Brazil	7281	Crominia	0.82	0.93	0.96	0	0	0
BRA	Brazil	7282	Cumari	0.58	0.87	0.94	722	0	0
BRA	Brazil	7283	Damianopolis	0.54	0.92	0.99	894	0	0
BRA	Brazil	7284	Damolandia	0.79	0.92	0.97	19	0	0
BRA	Brazil	7285	Davinopolis	1.00	1.00	1.00	0	0	0
BRA	Brazil	7286	Diorama	0.66	0.91	0.96	357	0	0
BRA	Brazil	7287	Divinopolis De Goias	0.61	0.95	1.00	952	0	0
BRA	Brazil	7288	Doverlandia	0.50	0.91	0.98	2449	0	0
BRA	Brazil	7289	Edealina	0.60	0.91	0.98	771	0	0
BRA	Brazil	7290	Edeia	0.59	0.88	0.95	2546	0	0
BRA	Brazil	7291	Estrela Do Norte	0.68	0.97	1.00	422	0	0
BRA	Brazil	7292	Faina	0.68	0.89	0.95	826	0	0
BRA	Brazil	7293	Fazenda Nova	0.54	0.88	0.95	1608	0	0
BRA	Brazil	7294	Firminopolis	0.39	0.83	0.94	5332	0	0
BRA	Brazil	7295	Flores De Goias	0.40	0.82	0.94	6233	0	0
BRA	Brazil	7296	Formosa	0.75	0.96	1.00	6186	0	0
BRA	Brazil	7297	Formoso	0.51	0.95	1.00	1386	0	0
BRA	Brazil	7298	Goianapolis	0.95	0.98	1.00	0	0	0
BRA	Brazil	7299	Goiandira	0.70	0.92	0.98	551	0	0
BRA	Brazil	7300	Goianesia	0.50	0.89	0.98	20609	0	0
BRA	Brazil	7301	Goiania	0.56	0.90	0.99	365252	0	0
BRA	Brazil	7302	Goianira	0.56	0.92	0.99	9299	0	0
BRA	Brazil	7303	Goias	0.70	0.90	0.95	2343	0	0
BRA	Brazil	7304	Goiatuba	0.54	0.91	0.98	8936	0	0

BRA	Brazil	7305	Gouvelandia	0.64	0.93	0.98	914	0	0
BRA	Brazil	7306	Guapo	0.64	0.92	0.99	2332	0	0
BRA	Brazil	7307	Guaraita	0.77	0.92	0.96	76	0	0
BRA	Brazil	7308	Guarani De Goias	0.37	0.84	0.98	1957	0	0
BRA	Brazil	7309	Guarinos	0.56	0.90	0.96	510	0	0
BRA	Brazil	7310	Heitorai	0.58	0.91	0.97	806	0	0
BRA	Brazil	7311	Hidrolandia	0.21	0.66	0.75	12742	3112	1006
BRA	Brazil	7312	Hidrolina	0.51	0.89	0.96	1125	0	0
BRA	Brazil	7313	Iaciara	0.72	0.92	0.97	1038	0	0
BRA	Brazil	7314	Inaciolandia	0.61	0.92	0.99	1142	0	0
BRA	Brazil	7315	Indiara	0.63	0.91	0.98	2601	0	0
BRA	Brazil	7316	Inhumas	0.59	0.91	0.97	11273	0	0
BRA	Brazil	7317	Ipameri	0.78	0.92	0.98	453	0	0
BRA	Brazil	7318	Ipora	0.68	0.94	1.00	3728	0	0
BRA	Brazil	7319	Israelandia	0.59	0.87	0.95	608	0	0
BRA	Brazil	7320	Itaberai	0.55	0.90	0.99	10252	0	0
BRA	Brazil	7321	Itaguari	0.49	0.92	0.99	1434	0	0
BRA	Brazil	7322	Itaguaru	0.70	0.91	0.96	537	0	0
BRA	Brazil	7323	Itaja	0.69	0.90	0.96	694	0	0
BRA	Brazil	7324	Itapaci	0.50	0.91	0.98	6562	0	0
BRA	Brazil	7325	Itapirapua	0.56	0.91	0.99	1644	0	0
BRA	Brazil	7326	Itapuranga	0.78	0.94	0.98	544	0	0
BRA	Brazil	7327	Itaruma	0.32	0.83	0.96	3340	0	0
BRA	Brazil	7328	Itaucu	0.65	0.90	0.97	1339	0	0
BRA	Brazil	7329	Itumbiara	0.52	0.88	0.97	29152	0	0
BRA	Brazil	7330	Ivolandia	0.48	0.86	0.95	834	0	0
BRA	Brazil	7331	Jandaia	0.80	0.93	0.97	9	0	0
BRA	Brazil	7332	Jaragua	0.58	0.89	0.98	10734	0	0
BRA	Brazil	7333	Jatai	0.43	0.92	1.00	36066	0	0
BRA	Brazil	7334	Jaupaci	0.75	0.92	0.98	152	0	0
BRA	Brazil	7335	Jesupolis	0.55	0.89	0.97	634	0	0
BRA	Brazil	7336	Joviania	0.61	0.90	0.97	1398	0	0
BRA	Brazil	7337	Jussara	0.21	0.66	0.75	11436	2793	903

BRA	Brazil	7338	Leopoldo De Bulhoes	0.59	0.88	0.94	1940	0	0
BRA	Brazil	7339	Luziania	0.58	0.91	1.00	44272	0	0
BRA	Brazil	7340	Mairipotaba	0.83	0.91	0.96	0	0	0
BRA	Brazil	7341	Mambai	0.38	0.89	1.00	3395	0	0
BRA	Brazil	7342	Mara Rosa	0.87	0.97	1.00	0	0	0
BRA	Brazil	7343	Marzagao	0.94	0.95	0.97	0	0	0
BRA	Brazil	7344	Matrincha	0.84	0.94	0.98	0	0	0
BRA	Brazil	7345	Maurilandia	0.73	0.95	0.99	840	0	0
BRA	Brazil	7346	Mimoso De Goias	0.43	0.87	0.96	999	0	0
BRA	Brazil	7347	Minacu	0.72	0.95	1.00	2392	0	0
BRA	Brazil	7348	Mineiros	0.58	0.92	0.98	14014	0	0
BRA	Brazil	7349	Moipora	0.65	0.88	0.94	258	0	0
BRA	Brazil	7350	Monte Alegre De Goias	0.45	0.86	0.97	2936	0	0
BRA	Brazil	7351	Montes Claros De Goias	0.45	0.88	0.97	2858	0	0
BRA	Brazil	7352	Montividiu	0.55	0.91	0.99	3185	0	0
BRA	Brazil	7353	Montividiu Do Norte	0.62	0.93	0.97	819	0	0
BRA	Brazil	7354	Morrinhos	0.21	0.66	0.75	26823	6551	2118
BRA	Brazil	7355	Morro Agudo De Goias	0.96	0.97	0.98	0	0	0
BRA	Brazil	7356	Mossamedes	0.60	0.85	0.94	981	0	0
BRA	Brazil	7357	Mozarlandia	0.61	0.92	0.99	2844	0	0
BRA	Brazil	7358	Mundo Novo	0.21	0.66	0.75	3515	859	278
BRA	Brazil	7359	Mutunopolis	0.62	0.91	0.97	675	0	0
BRA	Brazil	7360	Nazario	0.61	0.89	0.97	1683	0	0
BRA	Brazil	7361	Neropolis	0.64	0.92	0.99	4420	0	0
BRA	Brazil	7362	Niquelandia	0.52	0.91	0.98	12964	0	0
BRA	Brazil	7363	Nova America	0.64	0.93	0.98	374	0	0
BRA	Brazil	7364	Nova Aurora	0.99	1.00	1.00	0	0	0
BRA	Brazil	7365	Nova Crixas	0.76	0.92	0.98	561	0	0
BRA	Brazil	7366	Nova Gloria	0.71	0.92	0.97	778	0	0
BRA	Brazil	7367	Nova Iguacu De Goias	0.62	0.92	0.99	541	0	0
BRA	Brazil	7368	Nova Roma	0.56	0.89	0.97	824	0	0
BRA	Brazil	7369	Nova Veneza	0.68	0.89	0.96	1152	0	0
BRA	Brazil	7370	Novo Brasil	0.83	0.92	0.96	0	0	0

BRA	Brazil	7371	Novo Gama	0.47	0.89	0.99	33190	0	0
BRA	Brazil	7372	Novo Planalto	0.54	0.88	0.97	1127	0	0
BRA	Brazil	7373	Orizona	0.61	0.88	0.96	2945	0	0
BRA	Brazil	7374	Ouro Verde De Goias	0.67	0.90	0.97	529	0	0
BRA	Brazil	7375	Ouvidor	0.50	0.86	0.96	1895	0	0
BRA	Brazil	7376	Padre Bernardo	0.56	0.87	0.98	7518	0	0
BRA	Brazil	7377	Palestina De Goias	0.52	0.88	0.96	981	0	0
BRA	Brazil	7378	Palmeiras De Goias	0.60	0.90	0.96	5613	0	0
BRA	Brazil	7379	Palmelo	0.58	0.90	0.97	444	0	0
BRA	Brazil	7380	Palminopolis	0.46	0.88	0.96	1235	0	0
BRA	Brazil	7381	Panama	0.56	0.90	0.97	629	0	0
BRA	Brazil	7382	Paranaiguara	0.48	0.88	0.97	2988	0	0
BRA	Brazil	7383	Parauna	0.62	0.93	0.99	2035	0	0
BRA	Brazil	7384	Perolandia	0.67	0.92	0.99	420	0	0
BRA	Brazil	7385	Petrolina De Goias	0.67	0.89	0.95	1437	0	0
BRA	Brazil	7386	Pilar De Goias	0.77	0.90	0.94	77	0	0
BRA	Brazil	7387	Piracanjuba	0.71	0.90	0.96	2161	0	0
BRA	Brazil	7388	Piranhas	0.21	0.66	0.75	6596	1611	521
BRA	Brazil	7389	Pirenopolis	0.64	0.90	0.98	3868	0	0
BRA	Brazil	7390	Pires Do Rio	0.74	0.91	0.97	1956	0	0
BRA	Brazil	7391	Planaltina	0.83	0.96	1.00	0	0	0
BRA	Brazil	7392	Pontalina	0.54	0.88	0.97	4636	0	0
BRA	Brazil	7393	Porangatu	0.58	0.90	0.98	10149	0	0
BRA	Brazil	7394	Porteirao	0.71	0.92	0.98	343	0	0
BRA	Brazil	7395	Portelandia	0.65	0.91	0.98	618	0	0
BRA	Brazil	7396	Posse	0.71	0.95	1.00	3325	0	0
BRA	Brazil	7397	Professor Jamil	0.49	0.85	0.96	1045	0	0
BRA	Brazil	7398	Quirinopolis	0.50	0.89	0.97	14787	0	0
BRA	Brazil	7399	Rialma	0.90	0.94	0.96	0	0	0
BRA	Brazil	7400	Rianapolis	0.74	0.94	0.99	250	0	0
BRA	Brazil	7401	Rio Quente	0.92	0.95	0.98	0	0	0
BRA	Brazil	7402	Rio Verde	0.53	0.90	0.98	61355	0	0
BRA	Brazil	7403	Rubiataba	0.67	0.90	0.98	2521	0	0

BRA	Brazil	7404	Sanclerlandia	0.59	0.90	0.97	1636	0	0
BRA	Brazil	7405	Santa Barbara De Goias	0.58	0.94	0.99	1376	0	0
BRA	Brazil	7406	Santa Cruz De Goias	0.66	0.87	0.92	487	0	0
BRA	Brazil	7407	Santa Fe De Goias	0.70	0.93	0.98	517	0	0
BRA	Brazil	7408	Santa Helena De Goias	0.66	0.92	0.98	5228	0	0
BRA	Brazil	7409	Santa Isabel	0.92	0.96	0.99	0	0	0
BRA	Brazil	7410	Santa Rita Do Araguaia	0.57	0.87	0.96	2010	0	0
BRA	Brazil	7411	Santa Rita Do Novo Destino	0.79	0.90	0.94	20	0	0
BRA	Brazil	7412	Santa Rosa De Goias	0.60	0.91	0.98	558	0	0
BRA	Brazil	7413	Santa Tereza De Goias	0.53	0.93	0.99	1026	0	0
BRA	Brazil	7414	Santa Terezinha De Goias	0.73	0.92	0.97	673	0	0
BRA	Brazil	7415	Santo Antonio Da Barra	0.68	0.91	0.98	588	0	0
BRA	Brazil	7416	Santo Antonio De Goias	0.54	0.91	0.99	1600	0	0
BRA	Brazil	7417	Santo Antonio Do Descoberto	0.47	0.89	1.00	22614	0	0
BRA	Brazil	7418	Sao Domingos	0.21	0.66	0.75	7479	1827	591
BRA	Brazil	7419	Sao Francisco De Goias	0.57	0.88	0.97	1427	0	0
BRA	Brazil	7420	Sao Joao D'alianca	0.49	0.86	0.96	4113	0	0
BRA	Brazil	7421	Sao Joao Da Parauna	0.67	0.92	0.99	217	0	0
BRA	Brazil	7422	Sao Luis De Montes Belos	0.43	0.85	0.95	12112	0	0
BRA	Brazil	7423	Sao Luiz Do Norte	0.51	0.90	0.98	1481	0	0
BRA	Brazil	7424	Sao Miguel Do Araguaia	0.72	0.93	0.99	1838	0	0
BRA	Brazil	7425	Sao Miguel Do Passa Quatro	0.50	0.91	0.98	1215	0	0
BRA	Brazil	7426	Sao Patricio	0.47	0.86	0.97	642	0	0
BRA	Brazil	7427	Sao Simao	0.49	0.88	0.96	6281	0	0
BRA	Brazil	7428	Senador Canedo	0.63	0.92	0.99	17398	0	0
BRA	Brazil	7429	Serranopolis	0.61	0.91	0.99	1567	0	0
BRA	Brazil	7430	Silvania	0.38	0.86	0.95	12656	0	0
BRA	Brazil	7431	Simolandia	0.61	0.90	0.99	1190	0	0
BRA	Brazil	7432	Sitio D'abadia	0.32	0.83	0.95	1469	0	0
BRA	Brazil	7433	Taquaral De Goias	0.43	0.88	0.97	1355	0	0
BRA	Brazil	7434	Teresina De Goias	0.65	0.94	1.00	490	0	0
BRA	Brazil	7435	Terezopolis De Goias	0.60	0.91	0.98	1514	0	0
BRA	Brazil	7436	Tres Ranchos	0.71	0.92	0.97	268	0	0

BRA	Brazil	7437	Trindade	0.39	0.87	1.00	47726	0	0
BRA	Brazil	7438	Trombas	0.60	0.88	0.95	702	0	0
BRA	Brazil	7439	Turvania	0.68	0.92	0.97	605	0	0
BRA	Brazil	7440	Turvelandia	0.56	0.88	0.97	1561	0	0
BRA	Brazil	7441	Uirapuru	0.51	0.91	0.98	855	0	0
BRA	Brazil	7442	Uruacu	0.73	0.93	0.98	2837	0	0
BRA	Brazil	7443	Uruana	0.53	0.89	0.98	3790	0	0
BRA	Brazil	7444	Urutai	0.69	0.88	0.94	338	0	0
BRA	Brazil	7445	Valparaiso De Goias	0.50	0.90	1.00	47916	0	0
BRA	Brazil	7446	Varjao	0.57	0.90	0.97	841	0	0
BRA	Brazil	7447	Vianopolis	0.63	0.92	0.99	2303	0	0
BRA	Brazil	7448	Vicentinopolis	0.57	0.94	0.99	1895	0	0
BRA	Brazil	7449	Vila Boa	0.70	0.93	1.00	603	0	0
BRA	Brazil	7450	Vila Propicio	0.52	0.86	0.95	1578	0	0
BRA	Brazil	7451	Acailandia	0.38	0.92	1.00	49326	0	0
BRA	Brazil	7452	Afonso Cunha	0.36	0.85	0.98	2770	0	0
BRA	Brazil	7453	Agua Doce Do Maranhao	0.35	0.85	0.99	5643	0	0
BRA	Brazil	7454	Alcantara	0.34	0.87	0.98	10285	0	0
BRA	Brazil	7455	Aldeias Altas	0.35	0.82	0.94	12628	0	0
BRA	Brazil	7456	Altamira Do Maranhao	0.46	0.93	1.00	1488	0	0
BRA	Brazil	7457	Alto Alegre Do Maranhao	0.35	0.85	0.99	13035	0	0
BRA	Brazil	7458	Alto Alegre Do Pindare	0.35	0.87	0.99	16001	0	0
BRA	Brazil	7459	Alto Parnaiba	0.35	0.85	0.99	5093	0	0
BRA	Brazil	7460	Amapa Do Maranhao	0.39	0.92	1.00	3663	0	0
BRA	Brazil	7461	Amarante Do Maranhao	0.35	0.86	1.00	20344	0	0
BRA	Brazil	7462	Anajatuba	0.33	0.83	0.98	15312	0	0
BRA	Brazil	7463	Anapurus	0.35	0.86	0.99	7387	0	0
BRA	Brazil	7464	Apicum-acu	0.34	0.81	0.94	7031	0	0
BRA	Brazil	7465	Araguana	0.35	0.86	0.99	8060	0	0
BRA	Brazil	7466	Araioses	0.35	0.84	0.97	21144	0	0
BRA	Brazil	7467	Arame	0.34	0.85	0.98	12195	0	0
BRA	Brazil	7468	Arari	0.36	0.89	1.00	13405	0	0
BRA	Brazil	7469	Axixa	0.36	0.87	1.00	5622	0	0

BRA	Brazil	7470	Bacabal	0.36	0.86	1.00	43037	0	0
BRA	Brazil	7471	Bacabeira	0.40	0.87	0.98	7771	0	0
BRA	Brazil	7472	Bacuri	0.35	0.81	0.93	8015	0	0
BRA	Brazil	7473	Bacurituba	0.36	0.86	0.99	2167	0	0
BRA	Brazil	7474	Balsas	0.39	0.89	1.00	41618	0	0
BRA	Brazil	7475	Barao De Grajau	0.33	0.81	0.94	9723	0	0
BRA	Brazil	7476	Barra Do Corda	0.36	0.86	1.00	42401	0	0
BRA	Brazil	7477	Barreirinhas	0.36	0.88	1.00	25352	0	0
BRA	Brazil	7478	Bela Vista Do Maranhao	0.35	0.84	0.98	5949	0	0
BRA	Brazil	7479	Belagua	0.36	0.83	0.95	3800	0	0
BRA	Brazil	7480	Benedito Leite	0.40	0.89	1.00	2546	0	0
BRA	Brazil	7481	Bequimao	0.35	0.83	0.95	9649	0	0
BRA	Brazil	7482	Bernardo Do Mearim	0.35	0.82	0.94	3263	0	0
BRA	Brazil	7483	Boa Vista Do Gurupi	0.37	0.88	1.00	3386	0	0
BRA	Brazil	7484	Bom Jardim	0.34	0.83	0.97	22221	0	0
BRA	Brazil	7485	Bom Jesus Das Selvas	0.34	0.88	1.00	18529	0	0
BRA	Brazil	7486	Bom Lugar	0.40	0.89	1.00	5394	0	0
BRA	Brazil	7487	Brejo	0.35	0.84	0.97	16939	0	0
BRA	Brazil	7488	Brejo De Areia	0.43	0.91	1.00	1603	0	0
BRA	Brazil	7489	Buriti	0.36	0.86	0.99	13186	0	0
BRA	Brazil	7490	Buriti Bravo	0.34	0.81	0.93	11897	0	0
BRA	Brazil	7491	Buriticupu	0.35	0.86	1.00	34118	0	0
BRA	Brazil	7492	Buritirana	0.41	0.91	1.00	4200	0	0
BRA	Brazil	7493	Cachoeira Grande	0.36	0.87	0.99	4326	0	0
BRA	Brazil	7494	Cajapio	0.35	0.86	0.99	5268	0	0
BRA	Brazil	7495	Cajari	0.38	0.89	1.00	6459	0	0
BRA	Brazil	7496	Campestre Do Maranhao	0.39	0.92	1.00	6174	0	0
BRA	Brazil	7497	Candido Mendes	0.34	0.83	0.98	11168	0	0
BRA	Brazil	7498	Cantanhede	0.36	0.84	0.97	10380	0	0
BRA	Brazil	7499	Capinzal Do Norte	0.36	0.88	1.00	5058	0	0
BRA	Brazil	7500	Carolina	0.36	0.89	1.00	10952	0	0
BRA	Brazil	7501	Carutapera	0.36	0.90	1.00	10400	0	0
BRA	Brazil	7502	Caxias	0.35	0.83	0.96	76645	0	0

BRA	Brazil	7503	Cedral	0.40	0.91	1.00	3809	0	0
BRA	Brazil	7504	Central Do Maranhao	0.35	0.88	0.99	4123	0	0
BRA	Brazil	7505	Centro Do Guilherme	0.34	0.84	0.99	7861	0	0
BRA	Brazil	7506	Centro Novo Do Maranhao	0.35	0.87	1.00	8613	0	0
BRA	Brazil	7507	Chapadinha	0.36	0.86	0.99	36755	0	0
BRA	Brazil	7508	Cidelandia	0.38	0.91	1.00	6449	0	0
BRA	Brazil	7509	Codo	0.36	0.86	1.00	54805	0	0
BRA	Brazil	7510	Coelho Neto	0.35	0.84	0.97	22648	0	0
BRA	Brazil	7511	Colinas	0.40	0.91	1.00	15387	0	0
BRA	Brazil	7512	Conceicao Do Lago-acu	0.34	0.84	0.98	7785	0	0
BRA	Brazil	7513	Coroata	0.35	0.85	0.99	29906	0	0
BRA	Brazil	7514	Cururupu	0.41	0.93	1.00	10723	0	0
BRA	Brazil	7515	Davinopolis	0.21	0.66	0.75	7328	1790	579
BRA	Brazil	7516	Dom Pedro	0.35	0.86	0.99	13620	0	0
BRA	Brazil	7517	Duque Bacelar	0.36	0.88	1.00	5078	0	0
BRA	Brazil	7518	Esperantinopolis	0.34	0.81	0.92	9880	0	0
BRA	Brazil	7519	Estreito	0.39	0.88	1.00	18868	0	0
BRA	Brazil	7520	Feira Nova Do Maranhao	0.36	0.86	0.99	4139	0	0
BRA	Brazil	7521	Fernando Falcao	0.38	0.89	1.00	4283	0	0
BRA	Brazil	7522	Formosa Da Serra Negra	0.34	0.85	0.99	9319	0	0
BRA	Brazil	7523	Fortaleza Dos Nogueiras	0.36	0.85	0.98	5304	0	0
BRA	Brazil	7524	Fortuna	0.36	0.88	1.00	6827	0	0
BRA	Brazil	7525	Godofredo Viana	0.33	0.84	0.98	5602	0	0
BRA	Brazil	7526	Goncalves Dias	0.35	0.85	0.99	8904	0	0
BRA	Brazil	7527	Governador Archer	0.36	0.86	1.00	4805	0	0
BRA	Brazil	7528	Governador Edison Lobao	0.37	0.89	1.00	8245	0	0
BRA	Brazil	7529	Governador Eugenio Barros	0.36	0.85	0.98	6713	0	0
BRA	Brazil	7530	Governador Luiz Rocha	0.40	0.91	1.00	2917	0	0
BRA	Brazil	7531	Governador Newton Bello	0.28	0.77	0.91	11140	604	0
BRA	Brazil	7532	Governador Nunes Freire	0.36	0.88	1.00	11942	0	0
BRA	Brazil	7533	Graca Aranha	0.38	0.86	0.98	3630	0	0
BRA	Brazil	7534	Grajau	0.37	0.89	1.00	30331	0	0
BRA	Brazil	7535	Guimaraes	0.34	0.84	0.98	5526	0	0

BRA	Brazil	7536	Humberto De Campos	0.36	0.85	0.98	12082	0	0
BRA	Brazil	7537	Icatu	0.33	0.82	0.95	13341	0	0
BRA	Brazil	7538	Igarape Do Meio	0.34	0.82	0.95	5145	0	0
BRA	Brazil	7539	Igarape Grande	0.39	0.86	0.99	3951	0	0
BRA	Brazil	7540	Imperatriz	0.36	0.89	1.00	116547	0	0
BRA	Brazil	7541	Itaipava Do Grajau	0.28	0.78	0.92	20042	874	0
BRA	Brazil	7542	Itapecuru Mirim	0.35	0.86	0.99	29727	0	0
BRA	Brazil	7543	Itinga Do Maranhao	0.42	0.90	1.00	8025	0	0
BRA	Brazil	7544	Jatoba	0.60	0.93	0.99	1642	0	0
BRA	Brazil	7545	Jenipapo Dos Vieiras	0.47	0.95	1.00	3656	0	0
BRA	Brazil	7546	Joao Lisboa	0.36	0.88	1.00	9504	0	0
BRA	Brazil	7547	Joselandia	0.39	0.89	1.00	6806	0	0
BRA	Brazil	7548	Junco Do Maranhao	0.41	0.93	1.00	1869	0	0
BRA	Brazil	7549	Lago Da Pedra	0.34	0.85	0.99	38705	0	0
BRA	Brazil	7550	Lago Do Junco	0.30	0.81	0.95	10105	0	0
BRA	Brazil	7551	Lago Dos Rodrigues	0.61	0.98	1.00	503	0	0
BRA	Brazil	7552	Lago Verde	0.40	0.90	1.00	2479	0	0
BRA	Brazil	7553	Lagoa Do Mato	0.36	0.84	0.97	4767	0	0
BRA	Brazil	7554	Lagoa Grande Do Maranhao	0.38	0.88	1.00	3588	0	0
BRA	Brazil	7555	Lajeado Novo	0.57	0.93	1.00	1830	0	0
BRA	Brazil	7556	Lima Campos	0.34	0.86	0.99	5459	0	0
BRA	Brazil	7557	Loreto	0.37	0.86	0.99	5683	0	0
BRA	Brazil	7558	Luis Domingues	0.38	0.90	1.00	2823	0	0
BRA	Brazil	7559	Magalhaes De Almeida	0.31	0.78	0.90	11024	403	0
BRA	Brazil	7560	Maracacume	0.35	0.86	0.99	11899	0	0
BRA	Brazil	7561	Maraja Do Sena	0.50	0.92	1.00	1839	0	0
BRA	Brazil	7562	Maranhaozinho	0.34	0.86	0.99	8487	0	0
BRA	Brazil	7563	Mata Roma	0.35	0.82	0.94	7949	0	0
BRA	Brazil	7564	Matinha	0.34	0.83	0.96	11033	0	0
BRA	Brazil	7565	Matoes	0.35	0.85	0.98	14613	0	0
BRA	Brazil	7566	Matoes Do Norte	0.29	0.79	0.94	8301	116	0
BRA	Brazil	7567	Milagres Do Maranhao	0.36	0.87	0.99	4577	0	0
BRA	Brazil	7568	Mirador	0.39	0.90	0.99	8869	0	0

BRA	Brazil	7569	Miranda Do Norte	0.31	0.79	0.93	16587	180	0
BRA	Brazil	7570	Mirinzal	0.34	0.81	0.93	6952	0	0
BRA	Brazil	7571	Moncao	0.33	0.81	0.94	18753	0	0
BRA	Brazil	7572	Montes Altos	0.43	0.89	1.00	3372	0	0
BRA	Brazil	7573	Morros	0.34	0.85	0.99	8373	0	0
BRA	Brazil	7574	Nina Rodrigues	0.35	0.84	0.97	6876	0	0
BRA	Brazil	7575	Nova Colinas	0.38	0.88	1.00	2442	0	0
BRA	Brazil	7576	Nova Iorque	0.48	0.88	0.99	1486	0	0
BRA	Brazil	7577	Nova Olinda Do Maranhao	0.35	0.84	0.97	8910	0	0
BRA	Brazil	7578	Olho D'agua Das Cunhas	0.34	0.83	0.96	10215	0	0
BRA	Brazil	7579	Olinda Nova Do Maranhao	0.34	0.82	0.95	7085	0	0
BRA	Brazil	7580	Paco Do Lumiar	0.32	0.82	0.97	61105	0	0
BRA	Brazil	7581	Palmeirandia	0.34	0.83	0.96	8355	0	0
BRA	Brazil	7582	Paraibano	0.38	0.89	1.00	9325	0	0
BRA	Brazil	7583	Parnarama	0.35	0.83	0.95	17012	0	0
BRA	Brazil	7584	Passagem Franca	0.23	0.69	0.80	11089	2116	68
BRA	Brazil	7585	Pastos Bons	0.36	0.86	1.00	9899	0	0
BRA	Brazil	7586	Paulino Neves	0.32	0.83	0.97	9870	0	0
BRA	Brazil	7587	Paulo Ramos	0.35	0.86	0.99	10260	0	0
BRA	Brazil	7588	Pedreiras	0.36	0.88	0.99	20419	0	0
BRA	Brazil	7589	Pedro Do Rosario	0.33	0.82	0.95	11439	0	0
BRA	Brazil	7590	Penalva	0.35	0.83	0.97	18091	0	0
BRA	Brazil	7591	Peri Mirim	0.34	0.85	0.99	6536	0	0
BRA	Brazil	7592	Peritoro	0.35	0.84	0.97	9785	0	0
BRA	Brazil	7593	Pindare Mirim	0.30	0.78	0.91	31475	1379	0
BRA	Brazil	7594	Pinheiro	0.35	0.86	1.00	39320	0	0
BRA	Brazil	7595	Pio Xii	0.55	0.97	1.00	856	0	0
BRA	Brazil	7596	Pirapemas	0.35	0.86	1.00	9241	0	0
BRA	Brazil	7597	Pocao De Pedras	0.35	0.85	0.99	10805	0	0
BRA	Brazil	7598	Porto Franco	0.38	0.89	1.00	10699	0	0
BRA	Brazil	7599	Porto Rico Do Maranhao	0.35	0.84	0.97	2910	0	0
BRA	Brazil	7600	Presidente Dutra	0.21	0.66	0.75	29073	7101	2296
BRA	Brazil	7601	Presidente Juscelino	0.37	0.90	1.00	6355	0	0

BRA	Brazil	7602	Presidente Medici	0.83	0.99	1.00	0	0	0
BRA	Brazil	7603	Presidente Sarney	0.32	0.84	0.98	9203	0	0
BRA	Brazil	7604	Presidente Vargas	0.37	0.88	1.00	4685	0	0
BRA	Brazil	7605	Primeira Cruz	0.31	0.82	0.98	10104	0	0
BRA	Brazil	7606	Raposa	0.34	0.83	0.97	14276	0	0
BRA	Brazil	7607	Riachao	0.40	0.90	1.00	7887	0	0
BRA	Brazil	7608	Ribamar Fiquene	0.38	0.88	1.00	3312	0	0
BRA	Brazil	7609	Rosario	0.35	0.85	0.99	19571	0	0
BRA	Brazil	7610	Sambaiba	0.33	0.85	0.98	2972	0	0
BRA	Brazil	7611	Santa Filomena Do Maranhao	0.40	0.90	1.00	2688	0	0
BRA	Brazil	7612	Santa Helena	0.40	0.90	1.00	18525	0	0
BRA	Brazil	7613	Santa Ines	0.21	0.66	0.75	31388	7666	2478
BRA	Brazil	7614	Santa Luzia	0.21	0.66	0.75	49249	12029	3889
BRA	Brazil	7615	Santa Luzia Do Parua	0.37	0.89	1.00	12075	0	0
BRA	Brazil	7616	Santa Quiteria Do Maranhao	0.37	0.89	1.00	12957	0	0
BRA	Brazil	7617	Santa Rita	0.35	0.84	0.97	18137	0	0
BRA	Brazil	7618	Santana Do Maranhao	0.35	0.81	0.93	9027	0	0
BRA	Brazil	7619	Santo Amaro Do Maranhao	0.34	0.85	0.99	6276	0	0
BRA	Brazil	7620	Santo Antonio Dos Lopes	0.53	0.95	1.00	2651	0	0
BRA	Brazil	7621	Sao Benedito Do Rio Preto	0.35	0.82	0.94	8918	0	0
BRA	Brazil	7622	Sao Bento	0.33	0.80	0.93	23017	5	0
BRA	Brazil	7623	Sao Bernardo	0.36	0.85	0.99	14510	0	0
BRA	Brazil	7624	Sao Domingos Do Azeitao	0.38	0.89	1.00	2598	0	0
BRA	Brazil	7625	Sao Domingos Do Maranhao	0.40	0.88	1.00	14875	0	0
BRA	Brazil	7626	Sao Felix De Balsas	0.46	0.88	0.99	2093	0	0
BRA	Brazil	7627	Sao Francisco Do Brejao	0.36	0.89	1.00	5325	0	0
BRA	Brazil	7628	Sao Francisco Do Maranhao	0.32	0.82	0.96	6667	0	0
BRA	Brazil	7629	Sao Joao Batista	0.34	0.80	0.91	9870	0	0
BRA	Brazil	7630	Sao Joao Do Caru	0.36	0.89	1.00	5361	0	0
BRA	Brazil	7631	Sao Joao Do Paraiso	0.75	0.98	1.00	617	0	0
BRA	Brazil	7632	Sao Joao Do Soter	0.35	0.84	0.98	9243	0	0
BRA	Brazil	7633	Sao Joao Dos Patos	0.35	0.87	1.00	12151	0	0
BRA	Brazil	7634	Sao Jose De Ribamar	0.33	0.84	0.99	106678	0	0

BRA	Brazil	7635	Sao Jose Dos Basilios	0.44	0.90	1.00	2968	0	0
BRA	Brazil	7636	Sao Luis	0.36	0.88	1.00	483059	0	0
BRA	Brazil	7637	Sao Luis Gonzaga Do Maranhao	0.31	0.81	0.96	17372	0	0
BRA	Brazil	7638	Sao Mateus Do Maranhao	0.35	0.86	0.99	18387	0	0
BRA	Brazil	7639	Sao Pedro Da Agua Branca	0.45	0.93	1.00	3396	0	0
BRA	Brazil	7640	Sao Pedro Dos Crentes	0.50	0.92	1.00	1387	0	0
BRA	Brazil	7641	Sao Raimundo Das Mangabeiras	0.35	0.84	0.97	8730	0	0
BRA	Brazil	7642	Sao Raimundo Do Doca Bezerra	0.43	0.91	1.00	1861	0	0
BRA	Brazil	7643	Sao Roberto	0.35	0.81	0.92	3242	0	0
BRA	Brazil	7644	Sao Vicente Ferrer	0.35	0.85	0.99	10271	0	0
BRA	Brazil	7645	Satubinha	0.38	0.88	0.99	1917	0	0
BRA	Brazil	7646	Senador Alexandre Costa	0.37	0.85	0.98	4575	0	0
BRA	Brazil	7647	Senador La Rocque	0.36	0.87	1.00	9986	0	0
BRA	Brazil	7648	Serrano Do Maranhao	0.26	0.75	0.89	10153	908	0
BRA	Brazil	7649	Sitio Novo	0.35	0.87	1.00	8093	0	0
BRA	Brazil	7650	Sucupira Do Norte	0.40	0.86	0.98	3418	0	0
BRA	Brazil	7651	Sucupira Do Riachao	0.38	0.89	1.00	2085	0	0
BRA	Brazil	7652	Tasso Fragoso	0.37	0.85	0.98	3686	0	0
BRA	Brazil	7653	Timbiras	0.34	0.81	0.93	12763	0	0
BRA	Brazil	7654	Timon	0.34	0.81	0.94	83983	0	0
BRA	Brazil	7655	Trizidela Do Vale	0.36	0.88	1.00	6080	0	0
BRA	Brazil	7656	Tufilandia	0.45	0.93	1.00	1035	0	0
BRA	Brazil	7657	Tuntum	0.36	0.86	1.00	20923	0	0
BRA	Brazil	7658	Turiacu	0.35	0.87	1.00	16399	0	0
BRA	Brazil	7659	Turilandia	0.35	0.84	0.97	10458	0	0
BRA	Brazil	7660	Tutoia	0.36	0.89	1.00	24919	0	0
BRA	Brazil	7661	Urbano Santos	0.36	0.85	0.99	13713	0	0
BRA	Brazil	7662	Vargem Grande	0.35	0.84	0.97	27632	0	0
BRA	Brazil	7663	Viana	0.21	0.66	0.75	32530	7945	2569
BRA	Brazil	7664	Vila Nova Dos Martirios	0.34	0.86	0.99	6935	0	0
BRA	Brazil	7665	Vitoria Do Mearim	0.35	0.86	1.00	18233	0	0
BRA	Brazil	7666	Vitorino Freire	0.43	0.91	0.99	6730	0	0
BRA	Brazil	7667	Ze Doca	0.21	0.66	0.75	24848	6069	1962

BRA	Brazil	7668	Agua Clara	0.65	0.92	0.99	2440	0	0
BRA	Brazil	7669	Alcinopolis	0.39	0.88	0.99	2172	0	0
BRA	Brazil	7670	Amambai	0.47	0.91	1.00	12923	0	0
BRA	Brazil	7671	Anastacio	0.50	0.88	0.99	7785	0	0
BRA	Brazil	7672	Anaurilandia	0.42	0.87	0.98	3446	0	0
BRA	Brazil	7673	Angelica	0.39	0.89	0.99	4325	0	0
BRA	Brazil	7674	Antonio Joao	0.55	0.93	1.00	2393	0	0
BRA	Brazil	7675	Aparecida Do Taboado	0.49	0.88	0.98	7796	0	0
BRA	Brazil	7676	Aquidauana	0.57	0.92	1.00	11282	0	0
BRA	Brazil	7677	Aral Moreira	0.45	0.89	1.00	4181	0	0
BRA	Brazil	7678	Bandeirantes	0.88	1.00	1.00	0	0	0
BRA	Brazil	7679	Bataguassu	0.53	0.89	0.99	6104	0	0
BRA	Brazil	7680	Bataipora	0.59	0.92	1.00	2365	0	0
BRA	Brazil	7681	Bela Vista	0.64	0.93	1.00	3854	0	0
BRA	Brazil	7682	Bodoquena	0.67	0.94	1.00	1076	0	0
BRA	Brazil	7683	Bonito	0.21	0.66	0.75	12886	3147	1017
BRA	Brazil	7684	Brasilandia	0.45	0.91	1.00	4196	0	0
BRA	Brazil	7685	Caarapo	0.50	0.91	1.00	8768	0	0
BRA	Brazil	7686	Camapua	0.45	0.91	1.00	6281	0	0
BRA	Brazil	7687	Campo Grande	0.21	0.66	0.75	523800	127937	41359
BRA	Brazil	7688	Caracol	0.56	0.95	1.00	1444	0	0
BRA	Brazil	7689	Cassilandia	0.52	0.91	0.99	5971	0	0
BRA	Brazil	7690	Chapadao Do Sul	0.46	0.89	0.99	8977	0	0
BRA	Brazil	7691	Corguinho	0.48	0.85	0.94	1996	0	0
BRA	Brazil	7692	Coronel Sapucaia	0.53	0.93	1.00	3832	0	0
BRA	Brazil	7693	Corumba	0.45	0.92	1.00	38323	0	0
BRA	Brazil	7694	Costa Rica	0.50	0.93	1.00	6601	0	0
BRA	Brazil	7695	Coxim	0.55	0.93	1.00	8257	0	0
BRA	Brazil	7696	Deodapolis	0.47	0.92	1.00	4291	0	0
BRA	Brazil	7697	Dois Irmaos Do Buriti	0.52	0.90	0.99	3217	0	0
BRA	Brazil	7698	Douradina	0.85	0.98	1.00	0	0	0
BRA	Brazil	7699	Dourados	0.48	0.90	1.00	69787	0	0
BRA	Brazil	7700	Eldorado	0.55	0.92	1.00	3122	0	0

BRA	Brazil	7701	Fatima Do Sul	0.44	0.89	1.00	7016	0	0
BRA	Brazil	7702	Gloria De Dourados	0.48	0.87	0.97	3235	0	0
BRA	Brazil	7703	Guia Lopes Da Laguna	0.64	0.93	1.00	1720	0	0
BRA	Brazil	7704	Iguatemi	0.47	0.92	1.00	5331	0	0
BRA	Brazil	7705	Inocencia	0.58	0.91	0.98	1759	0	0
BRA	Brazil	7706	Itapora	0.42	0.87	0.99	9158	0	0
BRA	Brazil	7707	Itaquirai	0.45	0.86	0.98	7250	0	0
BRA	Brazil	7708	Ivinhema	0.53	0.90	0.99	6376	0	0
BRA	Brazil	7709	Japora	0.44	0.89	0.99	3298	0	0
BRA	Brazil	7710	Jaraguari	0.46	0.83	0.94	2396	0	0
BRA	Brazil	7711	Jardim	0.21	0.66	0.75	15326	3743	1210
BRA	Brazil	7712	Jatei	0.55	0.94	1.00	1036	0	0
BRA	Brazil	7713	Juti	0.63	0.92	1.00	1146	0	0
BRA	Brazil	7714	Ladario	0.39	0.87	1.00	10225	0	0
BRA	Brazil	7715	Laguna Carapa	0.52	0.92	1.00	1985	0	0
BRA	Brazil	7716	Maracaju	0.63	0.90	0.98	7798	0	0
BRA	Brazil	7717	Miranda	0.58	0.92	0.99	6239	0	0
BRA	Brazil	7718	Mundo Novo	0.21	0.66	0.75	10807	2640	853
BRA	Brazil	7719	Navirai	0.50	0.89	0.99	16337	0	0
BRA	Brazil	7720	Nioaque	0.58	0.92	1.00	3179	0	0
BRA	Brazil	7721	Nova Alvorada Do Sul	0.46	0.90	1.00	7496	0	0
BRA	Brazil	7722	Nova Andradina	0.57	0.90	0.99	12161	0	0
BRA	Brazil	7723	Novo Horizonte Do Sul	0.64	0.94	1.00	709	0	0
BRA	Brazil	7724	Paranaiba	0.61	0.90	0.98	8065	0	0
BRA	Brazil	7725	Paranhos	0.42	0.91	1.00	5164	0	0
BRA	Brazil	7726	Pedro Gomes	0.64	0.94	1.00	1259	0	0
BRA	Brazil	7727	Ponta Pora	0.54	0.91	1.00	22689	0	0
BRA	Brazil	7728	Porto Murtinho	0.40	0.89	1.00	6726	0	0
BRA	Brazil	7729	Ribas Do Rio Pardo	0.49	0.88	0.98	7801	0	0
BRA	Brazil	7730	Rio Brilhante	0.47	0.91	1.00	12292	0	0
BRA	Brazil	7731	Rio Negro	1.00	1.00	1.00	0	0	0
BRA	Brazil	7732	Rio Verde De Mato Grosso	0.43	0.90	1.00	7808	0	0
BRA	Brazil	7733	Rochedo	0.65	0.93	0.98	727	0	0

BRA	Brazil	7734	Santa Rita Do Pardo	0.54	0.88	0.96	2123	0	0
BRA	Brazil	7735	Sao Gabriel Do Oeste	0.48	0.91	0.99	8477	0	0
BRA	Brazil	7736	Selviria	0.74	0.94	0.99	419	0	0
BRA	Brazil	7737	Sete Quedas	0.50	0.92	1.00	3236	0	0
BRA	Brazil	7738	Sidrolandia	0.38	0.86	0.98	24300	0	0
BRA	Brazil	7739	Sonora	0.61	0.95	1.00	3631	0	0
BRA	Brazil	7740	Tacuru	0.46	0.91	1.00	3882	0	0
BRA	Brazil	7741	Taquarussu	0.54	0.91	0.99	974	0	0
BRA	Brazil	7742	Terenos	0.35	0.82	0.95	9513	0	0
BRA	Brazil	7743	Tres Lagoas	0.57	0.91	0.99	27602	0	0
BRA	Brazil	7744	Vicentina	0.55	0.91	0.99	1540	0	0
BRA	Brazil	7745	Acorizal	0.50	0.92	1.00	1637	0	0
BRA	Brazil	7746	Agua Boa	0.40	0.90	1.00	11660	0	0
BRA	Brazil	7747	Alta Floresta	0.49	0.93	1.00	16188	0	0
BRA	Brazil	7748	Alto Araguaia	0.53	0.88	0.96	5024	0	0
BRA	Brazil	7749	Alto Boa Vista	0.40	0.87	0.99	5961	0	0
BRA	Brazil	7750	Alto Garcas	0.55	0.89	0.98	3011	0	0
BRA	Brazil	7751	Alto Paraguai	0.34	0.83	0.95	5106	0	0
BRA	Brazil	7752	Alto Taquari	0.47	0.90	0.99	3743	0	0
BRA	Brazil	7753	Apiacas	0.40	0.90	1.00	4048	0	0
BRA	Brazil	7754	Araguaiana	0.55	0.89	0.96	801	0	0
BRA	Brazil	7755	Araguainha	0.99	0.99	1.00	0	0	0
BRA	Brazil	7756	Araputanga	0.40	0.90	1.00	6723	0	0
BRA	Brazil	7757	Arenapolis	0.38	0.85	0.98	4599	0	0
BRA	Brazil	7758	Aripuana	0.37	0.90	1.00	28416	0	0
BRA	Brazil	7759	Barao De Melgaco	0.33	0.83	0.96	3412	0	0
BRA	Brazil	7760	Barra Do Bugres	0.41	0.90	1.00	13377	0	0
BRA	Brazil	7761	Barra Do Garcas	0.56	0.90	0.99	14427	0	0
BRA	Brazil	7762	Brasnorte	0.37	0.85	0.99	8426	0	0
BRA	Brazil	7763	Caceres	0.40	0.91	1.00	39031	0	0
BRA	Brazil	7764	Campinapolis	0.67	0.94	1.00	2054	0	0
BRA	Brazil	7765	Campo Novo Do Parecis	0.39	0.91	1.00	14516	0	0
BRA	Brazil	7766	Campo Verde	0.40	0.89	0.99	17508	0	0

BRA	Brazil	7767	Campos De Julio	0.52	0.95	1.00	1926	0	0
BRA	Brazil	7768	Cana Brava Do Norte	0.54	0.92	1.00	1237	0	0
BRA	Brazil	7769	Canarana	0.21	0.66	0.75	12613	3081	996
BRA	Brazil	7770	Carlinda	0.41	0.90	0.99	4157	0	0
BRA	Brazil	7771	Castanheira	0.43	0.88	0.99	3151	0	0
BRA	Brazil	7772	Chapada Dos Guimaraes	0.48	0.90	1.00	5840	0	0
BRA	Brazil	7773	Claudia	0.47	0.88	0.98	4436	0	0
BRA	Brazil	7774	Cocalinho	0.55	0.88	0.98	1790	0	0
BRA	Brazil	7775	Colider	0.39	0.89	1.00	13621	0	0
BRA	Brazil	7776	Comodoro	0.48	0.93	1.00	6801	0	0
BRA	Brazil	7777	Confresa	0.42	0.87	0.99	11626	0	0
BRA	Brazil	7778	Cotriguacu	0.36	0.87	0.98	8943	0	0
BRA	Brazil	7779	Cuiaba	0.39	0.89	1.00	249226	0	0
BRA	Brazil	7780	Denise	0.40	0.88	0.99	3773	0	0
BRA	Brazil	7781	Diamantino	0.46	0.94	1.00	7532	0	0
BRA	Brazil	7782	Dom Aquino	0.43	0.85	0.97	3130	0	0
BRA	Brazil	7783	Feliz Natal	0.38	0.90	0.99	6057	0	0
BRA	Brazil	7784	Figueiropolis D'oeste	0.47	0.88	0.98	1218	0	0
BRA	Brazil	7785	Gaucha Do Norte	0.37	0.89	1.00	3233	0	0
BRA	Brazil	7786	General Carneiro	0.98	1.00	1.00	0	0	0
BRA	Brazil	7787	Gloria D'oeste	0.50	0.85	0.95	1020	0	0
BRA	Brazil	7788	Guaranta Do Norte	0.47	0.92	1.00	10967	0	0
BRA	Brazil	7789	Guiratinga	0.45	0.86	0.95	5321	0	0
BRA	Brazil	7790	Indiavai	0.47	0.90	0.99	893	0	0
BRA	Brazil	7791	Itauba	0.62	0.95	1.00	1375	0	0
BRA	Brazil	7792	Itiquira	0.52	0.90	0.99	3669	0	0
BRA	Brazil	7793	Jaciara	0.49	0.91	1.00	8469	0	0
BRA	Brazil	7794	Jangada	0.49	0.92	0.99	2594	0	0
BRA	Brazil	7795	Jauru	0.46	0.91	1.00	3255	0	0
BRA	Brazil	7796	Juara	0.53	0.93	1.00	9509	0	0
BRA	Brazil	7797	Juina	0.44	0.90	1.00	14926	0	0
BRA	Brazil	7798	Juruena	0.32	0.85	0.97	8131	0	0
BRA	Brazil	7799	Juscimeira	0.44	0.87	0.99	4137	0	0

BRA	Brazil	7800	Lambari D'oeste	0.50	0.91	0.99	1887	0	0
BRA	Brazil	7801	Lucas Do Rio Verde	0.36	0.86	0.98	31855	0	0
BRA	Brazil	7802	Luciara	0.64	0.93	1.00	369	0	0
BRA	Brazil	7803	Marcelandia	0.53	0.93	1.00	3198	0	0
BRA	Brazil	7804	Matupa	0.37	0.88	0.99	7831	0	0
BRA	Brazil	7805	Mirassol D'oeste	0.38	0.88	1.00	12873	0	0
BRA	Brazil	7806	Nobres	0.43	0.91	1.00	7012	0	0
BRA	Brazil	7807	Nortelandia	0.46	0.90	1.00	1816	0	0
BRA	Brazil	7808	Nossa Senhora Do Livramento	0.43	0.88	1.00	4612	0	0
BRA	Brazil	7809	Nova Bandeirantes	0.36	0.87	0.99	6799	0	0
BRA	Brazil	7810	Nova Brasilandia	0.58	0.93	1.00	939	0	0
BRA	Brazil	7811	Nova Canaa Do Norte	0.41	0.91	0.99	4960	0	0
BRA	Brazil	7812	Nova Guarita	0.48	0.91	0.99	1492	0	0
BRA	Brazil	7813	Nova Lacerda	0.43	0.91	1.00	2380	0	0
BRA	Brazil	7814	Nova Marilandia	0.35	0.88	0.98	1514	0	0
BRA	Brazil	7815	Nova Maringa	0.36	0.87	0.99	3893	0	0
BRA	Brazil	7816	Nova Monte Verde	0.39	0.92	0.99	3748	0	0
BRA	Brazil	7817	Nova Mutum	0.37	0.89	0.98	21358	0	0
BRA	Brazil	7818	Nova Olimpia	0.46	0.93	1.00	6876	0	0
BRA	Brazil	7819	Nova Ubirata	0.45	0.89	0.98	5048	0	0
BRA	Brazil	7820	Nova Xavantina	0.61	0.88	0.97	4139	0	0
BRA	Brazil	7821	Novo Horizonte Do Norte	0.39	0.85	0.96	1817	0	0
BRA	Brazil	7822	Novo Mundo	0.34	0.86	0.97	4165	0	0
BRA	Brazil	7823	Novo Sao Joaquim	0.66	0.92	0.99	1403	0	0
BRA	Brazil	7824	Paranaita	0.49	0.96	1.00	3506	0	0
BRA	Brazil	7825	Paranatinga	0.43	0.92	1.00	8266	0	0
BRA	Brazil	7826	Pedra Preta	0.41	0.88	0.99	6788	0	0
BRA	Brazil	7827	Peixoto De Azevedo	0.38	0.88	0.99	14343	0	0
BRA	Brazil	7828	Planalto Da Serra	0.65	0.92	0.98	404	0	0
BRA	Brazil	7829	Pocone	0.42	0.90	1.00	12594	0	0
BRA	Brazil	7830	Pontal Do Araguaia	0.57	0.87	0.95	1802	0	0
BRA	Brazil	7831	Ponte Branca	0.83	0.96	1.00	0	0	0
BRA	Brazil	7832	Pontes E Lacerda	0.43	0.91	1.00	18578	0	0

BRA	Brazil	7833	Porto Alegre Do Norte	0.44	0.88	1.00	4368	0	0
BRA	Brazil	7834	Porto Dos Gauchos	0.50	0.92	1.00	1505	0	0
BRA	Brazil	7835	Porto Esperidiao	0.54	0.94	1.00	2995	0	0
BRA	Brazil	7836	Porto Estrela	0.49	0.94	1.00	1026	0	0
BRA	Brazil	7837	Poxoreo	0.40	0.84	0.97	7749	0	0
BRA	Brazil	7838	Primavera Do Leste	0.45	0.90	1.00	20530	0	0
BRA	Brazil	7839	Querencia	0.39	0.87	0.99	7335	0	0
BRA	Brazil	7840	Reserva Do Cabacal	0.45	0.90	1.00	843	0	0
BRA	Brazil	7841	Ribeirao Cascalheira	0.42	0.86	0.99	4270	0	0
BRA	Brazil	7842	Ribeiraozinho	0.67	0.92	0.98	308	0	0
BRA	Brazil	7843	Rio Branco	0.21	0.66	0.75	3081	752	243
BRA	Brazil	7844	Rondonopolis	0.39	0.87	0.99	94234	0	0
BRA	Brazil	7845	Rosario Oeste	0.37	0.88	0.99	7857	0	0
BRA	Brazil	7846	Salto Do Ceu	0.50	0.91	1.00	1076	0	0
BRA	Brazil	7847	Santa Carmem	0.51	0.93	1.00	1292	0	0
BRA	Brazil	7848	Santa Terezinha	0.45	0.86	0.98	2901	0	0
BRA	Brazil	7849	Santo Afonso	0.40	0.87	1.00	1222	0	0
BRA	Brazil	7850	Santo Antonio Do Leverger	0.39	0.84	0.97	7897	0	0
BRA	Brazil	7851	Sao Felix Do Araguaia	0.40	0.86	0.99	5260	0	0
BRA	Brazil	7852	Sao Jose Do Povo	0.35	0.83	0.95	1789	0	0
BRA	Brazil	7853	Sao Jose Do Rio Claro	0.42	0.91	1.00	7785	0	0
BRA	Brazil	7854	Sao Jose Do Xingu	0.46	0.89	1.00	2763	0	0
BRA	Brazil	7855	Sao Jose Dos Quatro Marcos	0.43	0.89	0.99	7061	0	0
BRA	Brazil	7856	Sao Pedro Da Cipa	0.54	0.92	1.00	1079	0	0
BRA	Brazil	7857	Sapezal	0.35	0.86	0.98	12556	0	0
BRA	Brazil	7858	Sinop	0.43	0.89	1.00	52600	0	0
BRA	Brazil	7859	Sorriso	0.38	0.88	0.99	38584	0	0
BRA	Brazil	7860	Tabapora	0.45	0.90	1.00	3478	0	0
BRA	Brazil	7861	Tangara Da Serra	0.38	0.88	1.00	43557	0	0
BRA	Brazil	7862	Tapurah	0.34	0.83	0.95	13800	0	0
BRA	Brazil	7863	Terra Nova Do Norte	0.52	0.93	1.00	2896	0	0
BRA	Brazil	7864	Tesouro	0.37	0.82	0.93	1709	0	0
BRA	Brazil	7865	Torixoreu	0.57	0.90	0.98	904	0	0

BRA	Brazil	7866	Uniao Do Sul	0.65	0.95	1.00	558	0	0
BRA	Brazil	7867	Varzea Grande	0.39	0.88	1.00	117904	0	0
BRA	Brazil	7868	Vera	0.48	0.89	0.99	3626	0	0
BRA	Brazil	7869	Vila Bela Da Santissima Trindade	0.37	0.88	1.00	7065	0	0
BRA	Brazil	7870	Vila Rica	0.44	0.89	0.99	9413	0	0
BRA	Brazil	7871	Abadia Dos Dourados	0.43	0.75	0.95	2602	347	0
BRA	Brazil	7872	Abaete	0.35	0.76	0.96	10675	946	0
BRA	Brazil	7873	Abre Campo	0.28	0.67	0.94	7191	1720	0
BRA	Brazil	7874	Acaiaca	0.31	0.71	0.97	1981	365	0
BRA	Brazil	7875	Acucena	0.33	0.81	1.00	4453	0	0
BRA	Brazil	7876	Agua Boa	0.17	0.49	0.65	9198	4494	2194
BRA	Brazil	7877	Agua Comprida	0.75	0.89	0.97	97	0	0
BRA	Brazil	7878	Aguanil	0.39	0.77	0.96	1825	145	0
BRA	Brazil	7879	Aguas Formosas	0.30	0.71	0.97	9652	1741	0
BRA	Brazil	7880	Aguas Vermelhas	0.32	0.69	0.92	6595	1478	0
BRA	Brazil	7881	Aimores	0.31	0.74	0.99	12549	1648	0
BRA	Brazil	7882	Aiuruoca	0.26	0.64	0.89	3371	972	0
BRA	Brazil	7883	Alagoa	0.62	0.84	0.97	476	0	0
BRA	Brazil	7884	Albertina	0.29	0.68	0.94	1568	360	0
BRA	Brazil	7885	Alem Paraiba	0.30	0.68	0.92	17938	4333	0
BRA	Brazil	7886	Alfenas	0.29	0.75	0.98	40567	3839	0
BRA	Brazil	7887	Alfredo Vasconcelos	0.50	0.77	0.97	1922	176	0
BRA	Brazil	7888	Almenara	0.31	0.68	0.89	20731	5197	0
BRA	Brazil	7889	Alpercata	0.31	0.71	0.97	3630	687	0
BRA	Brazil	7890	Alpinopolis	0.31	0.72	0.98	9782	1552	0
BRA	Brazil	7891	Alterosa	0.32	0.76	0.99	6994	607	0
BRA	Brazil	7892	Alto Caparao	0.42	0.80	0.99	2092	16	0
BRA	Brazil	7893	Alto Jequitiba	0.33	0.69	0.93	4024	946	0
BRA	Brazil	7894	Alto Rio Doce	0.35	0.70	0.95	5259	1183	0
BRA	Brazil	7895	Alvarenga	0.30	0.74	0.98	2271	254	0
BRA	Brazil	7896	Alvinopolis	0.29	0.67	0.91	7979	1984	0
BRA	Brazil	7897	Alvorada De Minas	0.40	0.80	1.00	1479	0	0
BRA	Brazil	7898	Amparo Do Serra	0.32	0.75	0.98	2393	248	0

BRA	Brazil	7899	Andradas	0.27	0.71	0.97	21477	3849	0
BRA	Brazil	7900	Andrelandia	0.34	0.75	0.99	5792	623	0
BRA	Brazil	7901	Angelandia	0.30	0.72	0.98	6058	1011	0
BRA	Brazil	7902	Antonio Carlos	0.32	0.71	0.96	5377	1048	0
BRA	Brazil	7903	Antonio Dias	0.27	0.66	0.92	6086	1573	0
BRA	Brazil	7904	Antonio Prado De Minas	0.28	0.70	0.96	846	158	0
BRA	Brazil	7905	Aracai	0.55	0.78	0.96	598	52	0
BRA	Brazil	7906	Aracitaba	0.27	0.63	0.85	1124	358	0
BRA	Brazil	7907	Aracuai	0.30	0.70	0.95	18730	3895	0
BRA	Brazil	7908	Araguari	0.41	0.76	0.98	46006	4557	0
BRA	Brazil	7909	Arantina	0.36	0.76	0.98	1258	121	0
BRA	Brazil	7910	Araponga	0.51	0.81	0.99	2456	0	0
BRA	Brazil	7911	Arapora	0.54	0.83	0.96	2197	0	0
BRA	Brazil	7912	Arapua	0.90	0.98	1.00	0	0	0
BRA	Brazil	7913	Araujos	0.34	0.75	0.97	4210	420	0
BRA	Brazil	7914	Araxa	0.33	0.74	0.98	49648	6310	0
BRA	Brazil	7915	Arceburgo	0.34	0.78	0.98	4870	224	0
BRA	Brazil	7916	Arcos	0.31	0.72	0.96	19567	3078	0
BRA	Brazil	7917	Areado	0.34	0.75	0.98	6926	790	0
BRA	Brazil	7918	Argirita	0.34	0.74	0.97	1334	184	0
BRA	Brazil	7919	Aricanduva	0.54	0.80	1.00	1267	0	0
BRA	Brazil	7920	Arinos	0.79	0.90	0.98	134	0	0
BRA	Brazil	7921	Astolfo Dutra	0.45	0.81	0.98	4921	0	0
BRA	Brazil	7922	Ataleia	0.28	0.70	0.96	7197	1421	0
BRA	Brazil	7923	Augusto De Lima	0.52	0.81	0.98	1389	0	0
BRA	Brazil	7924	Baependi	0.34	0.76	0.97	9042	774	0
BRA	Brazil	7925	Baldim	0.36	0.75	0.98	3587	383	0
BRA	Brazil	7926	Bambui	0.28	0.70	0.96	12442	2358	0
BRA	Brazil	7927	Bandeira	0.30	0.69	0.94	2508	564	0
BRA	Brazil	7928	Bandeira Do Sul	0.31	0.72	0.95	2818	488	0
BRA	Brazil	7929	Barao De Cocais	0.36	0.79	1.00	14054	197	0
BRA	Brazil	7930	Barao De Monte Alto	0.52	0.84	0.98	1586	0	0
BRA	Brazil	7931	Barbacena	0.29	0.69	0.95	70879	14644	0

BRA	Brazil	7932	Barra Longa	0.27	0.64	0.85	3075	945	0
BRA	Brazil	7933	Barroso	0.30	0.71	0.96	10406	1902	0
BRA	Brazil	7934	Bela Vista De Minas	0.31	0.73	0.98	5211	795	0
BRA	Brazil	7935	Belmiro Braga	0.32	0.75	0.98	1802	193	0
BRA	Brazil	7936	Belo Horizonte	0.30	0.72	0.95	1273264	206960	0
BRA	Brazil	7937	Belo Oriente	0.32	0.77	1.00	12972	831	0
BRA	Brazil	7938	Belo Vale	0.28	0.65	0.88	4223	1194	0
BRA	Brazil	7939	Berilo	0.31	0.74	0.97	5934	757	0
BRA	Brazil	7940	Berizal	0.33	0.73	0.98	2244	331	0
BRA	Brazil	7941	Bertopolis	0.35	0.82	1.00	1724	0	0
BRA	Brazil	7942	Betim	0.30	0.75	0.99	220049	21520	0
BRA	Brazil	7943	Bias Fortes	0.30	0.69	0.93	1798	402	0
BRA	Brazil	7944	Bicas	0.29	0.68	0.92	7132	1714	0
BRA	Brazil	7945	Biquinhas	0.49	0.79	0.95	796	26	0
BRA	Brazil	7946	Boa Esperanca	0.17	0.49	0.65	25466	12444	6076
BRA	Brazil	7947	Bocaina De Minas	0.23	0.59	0.80	3248	1183	19
BRA	Brazil	7948	Bocaiuva	0.38	0.77	1.00	21082	1463	0
BRA	Brazil	7949	Bom Despacho	0.33	0.78	0.98	23806	818	0
BRA	Brazil	7950	Bom Jardim De Minas	0.56	0.84	1.00	1568	0	0
BRA	Brazil	7951	Bom Jesus Da Penha	0.42	0.80	0.98	1605	7	0
BRA	Brazil	7952	Bom Jesus Do Amparo	0.32	0.72	0.96	2901	487	0
BRA	Brazil	7953	Bom Jesus Do Galho	0.29	0.69	0.94	8004	1721	0
BRA	Brazil	7954	Bom Repouso	0.56	0.79	0.98	2598	128	0
BRA	Brazil	7955	Bom Sucesso	0.34	0.73	0.98	8305	1224	0
BRA	Brazil	7956	Bonfim	0.64	0.97	1.00	1110	0	0
BRA	Brazil	7957	Bonfinopolis De Minas	0.83	0.90	0.96	0	0	0
BRA	Brazil	7958	Bonito De Minas	0.38	0.78	1.00	4663	179	0
BRA	Brazil	7959	Borda Da Mata	0.35	0.69	0.94	8571	2157	0
BRA	Brazil	7960	Botelhos	0.39	0.78	0.98	6313	329	0
BRA	Brazil	7961	Botumirim	0.36	0.80	1.00	2815	0	0
BRA	Brazil	7962	Bras Pires	0.48	0.80	0.96	1465	0	0
BRA	Brazil	7963	Brasilandia De Minas	0.30	0.72	0.98	8132	1311	0
BRA	Brazil	7964	Brasilia De Minas	0.40	0.78	1.00	12987	697	0

BRA	Brazil	7965	Brasopolis	0.31	0.77	0.98	7202	499	0
BRA	Brazil	7966	Braunas	0.30	0.70	0.94	2486	503	0
BRA	Brazil	7967	Brumadinho	0.29	0.75	0.98	20448	2006	0
BRA	Brazil	7968	Bueno Brandao	0.30	0.71	0.96	5625	1009	0
BRA	Brazil	7969	Buenopolis	0.51	0.80	0.97	3088	0	0
BRA	Brazil	7970	Bugre	0.30	0.71	0.97	2278	397	0
BRA	Brazil	7971	Buritis	0.81	0.97	1.00	0	0	0
BRA	Brazil	7972	Buritizeiro	0.41	0.75	0.99	11578	1596	0
BRA	Brazil	7973	Cabeceira Grande	0.80	0.91	0.99	13	0	0
BRA	Brazil	7974	Cabo Verde	0.31	0.70	0.94	6969	1448	0
BRA	Brazil	7975	Cachoeira Da Prata	0.39	0.78	0.96	1544	71	0
BRA	Brazil	7976	Cachoeira De Minas	0.34	0.73	0.97	5410	810	0
BRA	Brazil	7977	Cachoeira De Pajeu	0.44	0.77	0.98	3320	282	0
BRA	Brazil	7978	Cachoeira Dourada	0.17	0.49	0.65	1734	847	414
BRA	Brazil	7979	Caetanopolis	0.29	0.73	0.95	5992	851	0
BRA	Brazil	7980	Caete	0.28	0.71	0.97	23007	3921	0
BRA	Brazil	7981	Caiana	0.27	0.62	0.82	2999	1005	0
BRA	Brazil	7982	Cajuri	0.32	0.76	0.97	2066	153	0
BRA	Brazil	7983	Caldas	0.34	0.73	0.96	6697	1095	0
BRA	Brazil	7984	Camacho	0.52	0.81	0.96	843	0	0
BRA	Brazil	7985	Camanducaia	0.32	0.72	0.97	10595	1805	0
BRA	Brazil	7986	Cambui	0.27	0.72	0.97	15522	2431	0
BRA	Brazil	7987	Cambuquira	0.29	0.67	0.91	6750	1760	0
BRA	Brazil	7988	Campanario	0.34	0.75	0.99	1717	192	0
BRA	Brazil	7989	Campanha	0.29	0.67	0.91	8599	2203	0
BRA	Brazil	7990	Campestre	0.17	0.49	0.65	13472	6583	3214
BRA	Brazil	7991	Campina Verde	0.36	0.73	0.95	8883	1372	0
BRA	Brazil	7992	Campo Azul	0.52	0.84	0.99	1082	0	0
BRA	Brazil	7993	Campo Belo	0.32	0.73	0.96	26381	3824	0
BRA	Brazil	7994	Campo Do Meio	0.34	0.80	0.99	5474	0	0
BRA	Brazil	7995	Campo Florido	0.49	0.83	0.99	2459	0	0
BRA	Brazil	7996	Campos Altos	0.32	0.76	0.98	7369	691	0
BRA	Brazil	7997	Campos Gerais	0.49	0.81	0.98	9067	0	0

BRA	Brazil	7998	Cana Verde	0.37	0.72	0.96	2424	475	0
BRA	Brazil	7999	Canaa	0.29	0.63	0.81	2386	799	0
BRA	Brazil	8000	Canapolis	0.17	0.49	0.65	7655	3740	1826
BRA	Brazil	8001	Candeias	0.17	0.49	0.65	9519	4651	2271
BRA	Brazil	8002	Cantagalo	0.98	1.00	1.00	0	0	0
BRA	Brazil	8003	Caparao	0.34	0.74	0.98	2717	370	0
BRA	Brazil	8004	Capela Nova	0.54	0.77	0.95	1242	144	0
BRA	Brazil	8005	Capelinha	0.34	0.75	0.99	17672	1834	0
BRA	Brazil	8006	Capetinga	0.34	0.76	0.98	3300	274	0
BRA	Brazil	8007	Capim Branco	0.33	0.73	0.96	4320	674	0
BRA	Brazil	8008	Capinopolis	0.42	0.81	0.98	6108	0	0
BRA	Brazil	8009	Capitao Andrade	0.32	0.74	0.99	2610	333	0
BRA	Brazil	8010	Capitao Eneas	0.53	0.81	0.99	4195	0	0
BRA	Brazil	8011	Capitolio	0.35	0.71	0.95	3879	781	0
BRA	Brazil	8012	Caputira	0.40	0.72	0.97	3768	751	0
BRA	Brazil	8013	Carai	0.29	0.71	0.98	12330	2237	0
BRA	Brazil	8014	Caranaiba	0.46	0.74	0.95	1195	227	0
BRA	Brazil	8015	Carandai	0.47	0.77	0.98	8250	691	0
BRA	Brazil	8016	Carangola	0.28	0.69	0.97	17501	3568	0
BRA	Brazil	8017	Caratinga	0.30	0.73	0.98	46402	6881	0
BRA	Brazil	8018	Carbonita	0.58	0.85	0.99	2119	0	0
BRA	Brazil	8019	Careacu	0.32	0.72	0.98	2739	427	0
BRA	Brazil	8020	Carlos Chagas	0.29	0.74	0.97	9955	1075	0
BRA	Brazil	8021	Carmesia	0.59	0.87	1.00	548	0	0
BRA	Brazil	8022	Carmo Da Cachoeira	0.31	0.74	0.98	6042	802	0
BRA	Brazil	8023	Carmo Da Mata	0.46	0.82	0.97	3896	0	0
BRA	Brazil	8024	Carmo De Minas	0.27	0.64	0.84	8030	2504	0
BRA	Brazil	8025	Carmo Do Cajuru	0.30	0.77	1.00	11047	724	0
BRA	Brazil	8026	Carmo Do Paranaiba	0.43	0.82	0.98	11478	0	0
BRA	Brazil	8027	Carmo Do Rio Claro	0.27	0.67	0.94	11424	2687	0
BRA	Brazil	8028	Carmopolis De Minas	0.29	0.71	0.96	9812	1637	0
BRA	Brazil	8029	Carneirinho	0.52	0.85	0.98	2818	0	0
BRA	Brazil	8030	Carrancas	0.28	0.63	0.83	2145	687	0

BRA	Brazil	8031	Carvalhopolis	0.32	0.74	0.98	1697	210	0
BRA	Brazil	8032	Carvalhos	0.45	0.77	0.96	1616	144	0
BRA	Brazil	8033	Casa Grande	0.29	0.69	0.93	1186	263	0
BRA	Brazil	8034	Cascalho Rico	0.70	0.85	0.97	316	0	0
BRA	Brazil	8035	Cassia	0.31	0.71	0.97	8842	1671	0
BRA	Brazil	8036	Cataguases	0.27	0.71	0.97	39287	6647	0
BRA	Brazil	8037	Catas Altas	0.33	0.78	0.99	2534	111	0
BRA	Brazil	8038	Catas Altas Da Noruega	0.41	0.74	0.97	1422	233	0
BRA	Brazil	8039	Catuji	0.32	0.70	0.94	3453	695	0
BRA	Brazil	8040	Catuti	0.49	0.84	0.99	1597	0	0
BRA	Brazil	8041	Caxambu	0.30	0.72	0.97	10961	1751	0
BRA	Brazil	8042	Cedro Do Abaete	0.69	0.86	0.97	134	0	0
BRA	Brazil	8043	Central De Minas	0.30	0.73	0.98	3560	481	0
BRA	Brazil	8044	Centralina	0.41	0.75	0.95	4089	508	0
BRA	Brazil	8045	Chacara	0.28	0.70	0.93	2880	543	0
BRA	Brazil	8046	Chale	0.33	0.73	0.99	2759	408	0
BRA	Brazil	8047	Chapada Do Norte	0.35	0.76	0.98	7259	692	0
BRA	Brazil	8048	Chapada Gaucha	0.79	0.92	1.00	67	0	0
BRA	Brazil	8049	Chiador	0.36	0.78	0.94	1893	78	0
BRA	Brazil	8050	Cipotanea	0.44	0.74	0.95	2413	396	0
BRA	Brazil	8051	Claraval	0.47	0.78	0.97	1267	95	0
BRA	Brazil	8052	Claro Dos Pocoes	0.44	0.77	0.97	2785	204	0
BRA	Brazil	8053	Claudio	0.35	0.74	0.98	12801	1723	0
BRA	Brazil	8054	Coimbra	0.56	0.83	0.97	1799	0	0
BRA	Brazil	8055	Coluna	0.37	0.80	0.99	3961	0	0
BRA	Brazil	8056	Comendador Gomes	0.50	0.82	0.98	939	0	0
BRA	Brazil	8057	Comercinho	0.39	0.80	0.98	3231	0	0
BRA	Brazil	8058	Conceicao Da Aparecida	0.33	0.75	0.98	4858	557	0
BRA	Brazil	8059	Conceicao Da Barra De Minas	0.43	0.79	0.96	1482	52	0
BRA	Brazil	8060	Conceicao Das Alagoas	0.40	0.79	0.99	10971	150	0
BRA	Brazil	8061	Conceicao Das Pedras	0.40	0.78	0.97	1156	44	0
BRA	Brazil	8062	Conceicao De Ipanema	0.31	0.71	0.99	2254	391	0
BRA	Brazil	8063	Conceicao Do Mato Dentro	0.46	0.90	1.00	6228	0	0

BRA	Brazil	8064	Conceicao Do Para	0.33	0.75	0.95	2680	262	0
BRA	Brazil	8065	Conceicao Do Rio Verde	0.31	0.71	0.97	6808	1298	0
BRA	Brazil	8066	Conceicao Dos Ouros	0.33	0.71	0.97	5439	1068	0
BRA	Brazil	8067	Conego Marinho	0.36	0.81	1.00	3233	0	0
BRA	Brazil	8068	Confins	0.33	0.72	0.95	3042	537	0
BRA	Brazil	8069	Congonhal	0.26	0.65	0.89	6432	1809	0
BRA	Brazil	8070	Congonhas	0.34	0.78	0.99	25701	1077	0
BRA	Brazil	8071	Congonhas Do Norte	0.47	0.83	0.99	1770	0	0
BRA	Brazil	8072	Conquista	0.39	0.82	0.97	3015	0	0
BRA	Brazil	8073	Conselheiro Lafaiete	0.38	0.76	0.98	53544	5549	0
BRA	Brazil	8074	Conselheiro Pena	0.31	0.77	1.00	11105	635	0
BRA	Brazil	8075	Consolacao	0.35	0.74	0.98	818	108	0
BRA	Brazil	8076	Contagem	0.30	0.75	0.99	326917	35000	0
BRA	Brazil	8077	Coqueiral	0.40	0.79	0.98	3762	77	0
BRA	Brazil	8078	Coracao De Jesus	0.37	0.75	0.98	11635	1290	0
BRA	Brazil	8079	Cordisburgo	0.50	0.77	0.95	2784	284	0
BRA	Brazil	8080	Cordislandia	0.30	0.68	0.91	1740	418	0
BRA	Brazil	8081	Corinto	0.40	0.78	0.99	9626	525	0
BRA	Brazil	8082	Coroaci	0.31	0.73	0.97	5064	705	0
BRA	Brazil	8083	Coromandel	0.38	0.76	0.96	11816	1241	0
BRA	Brazil	8084	Coronel Fabriciano	0.31	0.75	0.99	51375	4896	0
BRA	Brazil	8085	Coronel Murta	0.28	0.65	0.86	4757	1419	0
BRA	Brazil	8086	Coronel Pacheco	0.28	0.70	0.93	1901	366	0
BRA	Brazil	8087	Coronel Xavier Chaves	0.46	0.76	0.97	1214	140	0
BRA	Brazil	8088	Corrego Danta	0.38	0.72	0.92	1452	261	0
BRA	Brazil	8089	Corrego Do Bom Jesus	0.30	0.73	0.96	1911	270	0
BRA	Brazil	8090	Corrego Fundo	0.41	0.73	0.97	2461	426	0
BRA	Brazil	8091	Corrego Novo	0.31	0.70	0.94	1430	292	0
BRA	Brazil	8092	Couto De Magalhaes De Minas	0.31	0.74	0.99	2216	270	0
BRA	Brazil	8093	Crisolita	0.33	0.76	0.99	3163	261	0
BRA	Brazil	8094	Cristais	0.37	0.72	0.96	5422	1004	0
BRA	Brazil	8095	Cristalia	0.38	0.74	0.98	2532	381	0
BRA	Brazil	8096	Cristiano Otoni	0.40	0.73	0.97	2102	383	0

BRA	Brazil	8097	Cristina	0.30	0.70	0.96	5246	1028	0
BRA	Brazil	8098	Crucilandia	0.57	0.81	0.96	1166	0	0
BRA	Brazil	8099	Cruzeiro Da Fortaleza	0.67	0.86	1.00	502	0	0
BRA	Brazil	8100	Cruzilia	0.41	0.77	0.97	6040	429	0
BRA	Brazil	8101	Cuparaque	0.33	0.71	0.95	2407	462	0
BRA	Brazil	8102	Curral De Dentro	0.48	0.83	1.00	2339	0	0
BRA	Brazil	8103	Curvelo	0.33	0.73	0.97	37402	5229	0
BRA	Brazil	8104	Datas	0.31	0.69	0.92	2534	573	0
BRA	Brazil	8105	Delfim Moreira	0.44	0.77	0.98	2921	245	0
BRA	Brazil	8106	Delfinopolis	0.36	0.74	0.98	3158	396	0
BRA	Brazil	8107	Delta	0.63	0.90	1.00	1782	0	0
BRA	Brazil	8108	Descoberto	0.29	0.69	0.95	2583	548	0
BRA	Brazil	8109	Desterro De Entre Rios	0.36	0.73	0.96	3210	510	0
BRA	Brazil	8110	Desterro Do Melo	0.37	0.75	0.98	1300	164	0
BRA	Brazil	8111	Diamantina	0.31	0.71	0.98	23665	4223	0
BRA	Brazil	8112	Diogo De Vasconcelos	0.33	0.72	0.97	1788	297	0
BRA	Brazil	8113	Dionisio	0.30	0.68	0.93	4163	982	0
BRA	Brazil	8114	Divinesia	0.31	0.73	0.98	1789	240	0
BRA	Brazil	8115	Divino	0.31	0.70	0.94	9870	2056	0
BRA	Brazil	8116	Divino Das Laranjeiras	0.36	0.79	0.99	2258	72	0
BRA	Brazil	8117	Divinolandia De Minas	0.32	0.76	0.99	3748	332	0
BRA	Brazil	8118	Divinopolis	0.29	0.75	0.97	121721	12092	0
BRA	Brazil	8119	Divisa Alegre	0.31	0.70	0.95	3264	662	0
BRA	Brazil	8120	Divisa Nova	0.34	0.77	0.99	2826	208	0
BRA	Brazil	8121	Divisopolis	0.29	0.67	0.90	5363	1363	0
BRA	Brazil	8122	Dom Bosco	0.67	0.86	0.97	507	0	0
BRA	Brazil	8123	Dom Cavati	0.32	0.74	0.98	2585	314	0
BRA	Brazil	8124	Dom Joaquim	0.45	0.85	0.99	1539	0	0
BRA	Brazil	8125	Dom Silverio	0.28	0.67	0.93	2806	675	0
BRA	Brazil	8126	Dom Vicoso	0.29	0.69	0.95	1542	330	0
BRA	Brazil	8127	Dona Euzebia	0.51	0.80	0.99	1717	0	0
BRA	Brazil	8128	Dores De Campos	0.27	0.68	0.94	5364	1202	0
BRA	Brazil	8129	Dores De Guanhaes	0.31	0.78	0.99	2553	112	0

BRA	Brazil	8130	Dores Do Indaia	0.39	0.76	0.95	5769	493	0
BRA	Brazil	8131	Dores Do Turvo	0.80	0.89	0.95	1	0	0
BRA	Brazil	8132	Doresopolis	0.53	0.85	0.98	415	0	0
BRA	Brazil	8133	Douradoquara	0.56	0.81	0.96	467	0	0
BRA	Brazil	8134	Durande	0.33	0.70	0.93	3698	813	0
BRA	Brazil	8135	Eloi Mendes	0.30	0.71	0.97	14017	2448	0
BRA	Brazil	8136	Engenheiro Caldas	0.31	0.67	0.88	5372	1414	0
BRA	Brazil	8137	Engenheiro Navarro	0.58	0.85	1.00	1620	0	0
BRA	Brazil	8138	Entre Folhas	0.32	0.73	0.99	2409	338	0
BRA	Brazil	8139	Entre Rios De Minas	0.21	0.60	0.85	8997	2994	0
BRA	Brazil	8140	Ervalia	0.54	0.80	0.98	4942	0	0
BRA	Brazil	8141	Esmeraldas	0.29	0.73	0.98	38448	5356	0
BRA	Brazil	8142	Espera Feliz	0.30	0.75	0.99	12725	1173	0
BRA	Brazil	8143	Espinosa	0.45	0.77	0.99	11241	975	0
BRA	Brazil	8144	Espirito Santo Do Dourado	0.28	0.64	0.85	2468	762	0
BRA	Brazil	8145	Estiva	0.45	0.79	0.97	3979	97	0
BRA	Brazil	8146	Estrela Dalva	0.28	0.71	0.95	1301	235	0
BRA	Brazil	8147	Estrela Do Indaia	0.46	0.76	0.93	1238	131	0
BRA	Brazil	8148	Estrela Do Sul	0.46	0.74	0.93	2653	462	0
BRA	Brazil	8149	Eugenopolis	0.28	0.69	0.95	5870	1288	0
BRA	Brazil	8150	Ewbank Da Camara	0.32	0.72	0.97	1889	298	0
BRA	Brazil	8151	Extrema	0.32	0.71	0.96	17325	3070	0
BRA	Brazil	8152	Fama	0.40	0.76	0.95	936	104	0
BRA	Brazil	8153	Faria Lemos	0.52	0.80	0.99	955	0	0
BRA	Brazil	8154	Felicio Dos Santos	0.43	0.82	1.00	1859	0	0
BRA	Brazil	8155	Felisburgo	0.46	0.77	0.99	2508	209	0
BRA	Brazil	8156	Felixlandia	0.30	0.70	0.94	7713	1475	0
BRA	Brazil	8157	Fernandes Tourinho	0.39	0.75	0.96	1525	169	0
BRA	Brazil	8158	Ferros	0.35	0.77	0.98	4778	327	0
BRA	Brazil	8159	Fervedouro	0.52	0.79	0.99	2918	141	0
BRA	Brazil	8160	Florestal	0.28	0.72	0.96	3890	609	0
BRA	Brazil	8161	Formiga	0.34	0.78	0.97	31276	1339	0
BRA	Brazil	8162	Formoso	0.17	0.49	0.65	8577	4191	2046

BRA	Brazil	8163	Fortaleza De Minas	0.41	0.79	0.97	1673	55	0
BRA	Brazil	8164	Fortuna De Minas	0.44	0.83	0.97	1043	0	0
BRA	Brazil	8165	Francisco Badaro	0.30	0.72	0.95	5210	820	0
BRA	Brazil	8166	Francisco Dumont	0.37	0.79	1.00	2251	54	0
BRA	Brazil	8167	Francisco Sa	0.32	0.73	0.97	12827	1941	0
BRA	Brazil	8168	Franciscopolis	0.30	0.71	0.97	2844	518	0
BRA	Brazil	8169	Frei Gaspar	0.32	0.77	0.99	2898	196	0
BRA	Brazil	8170	Frei Inocencio	0.31	0.71	0.97	4833	896	0
BRA	Brazil	8171	Frei Lagonegro	0.43	0.82	0.99	1302	0	0
BRA	Brazil	8172	Fronteira	0.43	0.78	0.97	6738	395	0
BRA	Brazil	8173	Fronteira Dos Vales	0.31	0.72	0.98	2319	397	0
BRA	Brazil	8174	Fruta De Leite	0.33	0.76	0.99	2628	213	0
BRA	Brazil	8175	Frutal	0.41	0.75	0.96	22915	2751	0
BRA	Brazil	8176	Funilandia	0.32	0.72	0.97	2029	358	0
BRA	Brazil	8177	Galileia	0.32	0.73	0.97	3477	502	0
BRA	Brazil	8178	Gameleiras	0.44	0.84	0.99	1938	0	0
BRA	Brazil	8179	Glaucilandia	0.64	0.86	0.99	436	0	0
BRA	Brazil	8180	Goiabeira	0.30	0.67	0.90	1651	420	0
BRA	Brazil	8181	Goiana	0.28	0.68	0.93	2047	472	0
BRA	Brazil	8182	Goncalves	0.38	0.82	0.99	1847	0	0
BRA	Brazil	8183	Gonzaga	0.30	0.68	0.90	3076	768	0
BRA	Brazil	8184	Gouvea	0.37	0.74	0.97	5142	693	0
BRA	Brazil	8185	Governador Valadares	0.32	0.75	0.99	134200	13020	0
BRA	Brazil	8186	Grao Mogol	0.34	0.74	0.98	7410	895	0
BRA	Brazil	8187	Grupiara	0.69	0.87	0.95	156	0	0
BRA	Brazil	8188	Guanhaes	0.41	0.81	0.99	13292	0	0
BRA	Brazil	8189	Guape	0.41	0.79	0.97	5627	146	0
BRA	Brazil	8190	Guaraciaba	0.38	0.79	0.96	4407	80	0
BRA	Brazil	8191	Guaraciama	0.63	0.85	0.99	872	0	0
BRA	Brazil	8192	Guaranesia	0.29	0.72	0.98	9973	1592	0
BRA	Brazil	8193	Guarani	0.33	0.73	0.97	4219	626	0
BRA	Brazil	8194	Guarara	0.40	0.74	0.98	1790	269	0
BRA	Brazil	8195	Guarda-mor	0.60	0.83	0.99	1316	0	0

BRA	Brazil	8196	Guaxupe	0.29	0.73	0.98	26560	3873	0
BRA	Brazil	8197	Guidoval	0.29	0.70	0.96	3720	711	0
BRA	Brazil	8198	Guimarania	0.39	0.81	0.99	3014	0	0
BRA	Brazil	8199	Guiricema	0.52	0.81	0.95	2473	0	0
BRA	Brazil	8200	Gurinhata	0.55	0.80	0.96	1489	0	0
BRA	Brazil	8201	Heliodora	0.30	0.70	0.96	3316	677	0
BRA	Brazil	8202	Iapu	0.47	0.79	0.99	3609	99	0
BRA	Brazil	8203	Ibertioga	0.56	0.80	0.98	1256	12	0
BRA	Brazil	8204	Ibia	0.31	0.70	0.95	12249	2625	0
BRA	Brazil	8205	Ibiai	0.40	0.74	0.99	3358	538	0
BRA	Brazil	8206	Ibiracatu	0.45	0.85	1.00	2067	0	0
BRA	Brazil	8207	Ibiraci	0.34	0.74	0.99	6477	867	0
BRA	Brazil	8208	Ibirite	0.31	0.74	0.99	91313	12046	0
BRA	Brazil	8209	Ibitiura De Minas	0.28	0.67	0.92	1837	462	0
BRA	Brazil	8210	Ibituruna	0.66	0.83	0.97	425	0	0
BRA	Brazil	8211	Icarai De Minas	0.42	0.78	0.99	4444	232	0
BRA	Brazil	8212	Igarape	0.30	0.73	0.98	20746	2894	0
BRA	Brazil	8213	Igaratinga	0.28	0.72	0.96	5530	891	0
BRA	Brazil	8214	Iguatama	0.30	0.70	0.94	4104	788	0
BRA	Brazil	8215	Ijaci	0.35	0.72	0.97	2919	490	0
BRA	Brazil	8216	Ilicinea	0.30	0.71	0.97	6146	1169	0
BRA	Brazil	8217	Imbe De Minas	0.29	0.69	0.93	3300	749	0
BRA	Brazil	8218	Inconfidentes	0.27	0.66	0.90	3893	1040	0
BRA	Brazil	8219	Indaiabira	0.41	0.75	0.97	2916	358	0
BRA	Brazil	8220	Indianopolis	0.56	0.90	1.00	1649	0	0
BRA	Brazil	8221	Ingai	0.47	0.77	0.97	931	90	0
BRA	Brazil	8222	Inhapim	0.35	0.73	0.98	10921	1594	0
BRA	Brazil	8223	Inhauma	0.31	0.74	0.97	3066	349	0
BRA	Brazil	8224	Inimutaba	0.46	0.78	0.96	2438	177	0
BRA	Brazil	8225	Ipaba	0.30	0.75	0.98	8816	960	0
BRA	Brazil	8226	Ipanema	0.28	0.67	0.93	10446	2531	0
BRA	Brazil	8227	Ipatinga	0.28	0.72	0.96	132685	21000	0
BRA	Brazil	8228	Ipiacu	0.56	0.84	0.96	1032	0	0

BRA	Brazil	8229	Ipuiuna	0.35	0.75	0.96	4519	537	0
BRA	Brazil	8230	Irai De Minas	0.62	0.85	0.99	1131	0	0
BRA	Brazil	8231	Itabira	0.29	0.74	0.97	61196	7515	0
BRA	Brazil	8232	Itabirinha De Mantena	0.31	0.72	0.97	5629	933	0
BRA	Brazil	8233	Itabirito	0.29	0.74	0.98	26112	2960	0
BRA	Brazil	8234	Itacambira	0.39	0.72	0.96	2232	454	0
BRA	Brazil	8235	Itacarambi	0.36	0.77	1.00	8473	520	0
BRA	Brazil	8236	Itaguara	0.37	0.76	0.96	5707	563	0
BRA	Brazil	8237	Itaipe	0.33	0.76	1.00	5900	526	0
BRA	Brazil	8238	Itajuba	0.32	0.77	0.99	47111	2701	0
BRA	Brazil	8239	Itamarandiba	0.31	0.72	0.99	17070	2737	0
BRA	Brazil	8240	Itamarati De Minas	0.27	0.67	0.92	2444	621	0
BRA	Brazil	8241	Itambacuri	0.31	0.70	0.96	11661	2274	0
BRA	Brazil	8242	Itambe Do Mato Dentro	0.41	0.78	0.97	864	56	0
BRA	Brazil	8243	Itamogi	0.31	0.73	0.98	5123	739	0
BRA	Brazil	8244	Itamonte	0.28	0.66	0.90	7993	2141	0
BRA	Brazil	8245	Itanhandu	0.31	0.71	0.96	7609	1342	0
BRA	Brazil	8246	Itanhomi	0.36	0.76	0.99	5507	464	0
BRA	Brazil	8247	Itaobim	0.44	0.76	0.99	7814	901	0
BRA	Brazil	8248	Itapagipe	0.31	0.70	0.93	7379	1585	0
BRA	Brazil	8249	Itapecerica	0.28	0.73	0.96	11390	1647	0
BRA	Brazil	8250	Itapeva	0.30	0.72	0.98	4860	799	0
BRA	Brazil	8251	Itatiaiucu	0.41	0.82	1.00	4314	0	0
BRA	Brazil	8252	Itau De Minas	0.35	0.71	0.95	7211	1434	0
BRA	Brazil	8253	Itauna	0.29	0.75	0.97	47147	4726	0
BRA	Brazil	8254	Itaverava	0.46	0.78	0.97	1918	132	0
BRA	Brazil	8255	Itinga	0.33	0.74	0.98	7112	974	0
BRA	Brazil	8256	Itueta	0.34	0.78	0.99	2817	137	0
BRA	Brazil	8257	Ituiutaba	0.35	0.74	0.96	46786	6282	0
BRA	Brazil	8258	Itumirim	0.33	0.76	0.98	2807	250	0
BRA	Brazil	8259	Iturama	0.42	0.79	0.99	14908	520	0
BRA	Brazil	8260	Itutinga	0.30	0.74	0.96	2054	232	0
BRA	Brazil	8261	Jaboticatubas	0.27	0.68	0.95	10569	2430	0

BRA	Brazil	8262	Jacinto	0.30	0.69	0.93	6314	1381	0
BRA	Brazil	8263	Jacui	0.34	0.76	0.99	3567	280	0
BRA	Brazil	8264	Jacutinga	0.29	0.69	0.94	13000	2895	0
BRA	Brazil	8265	Jaguaracu	0.26	0.68	0.92	2143	476	0
BRA	Brazil	8266	Jaiba	0.39	0.76	0.99	15233	1683	0
BRA	Brazil	8267	Jampruca	0.31	0.71	0.96	2463	452	0
BRA	Brazil	8268	Janauba	0.38	0.76	0.99	29508	2756	0
BRA	Brazil	8269	Januaria	0.35	0.78	1.00	31644	1191	0
BRA	Brazil	8270	Japaraiba	0.51	0.82	0.99	1260	0	0
BRA	Brazil	8271	Japonvar	0.45	0.82	0.99	2947	0	0
BRA	Brazil	8272	Jeceaba	0.42	0.79	0.96	1903	29	0
BRA	Brazil	8273	Jenipapo De Minas	0.33	0.73	0.98	3557	536	0
BRA	Brazil	8274	Jequeri	0.30	0.69	0.95	6580	1483	0
BRA	Brazil	8275	Jequitai	0.56	0.81	0.97	1873	0	0
BRA	Brazil	8276	Jequitiba	0.36	0.78	0.97	2312	121	0
BRA	Brazil	8277	Jequitinhonha	0.32	0.71	0.96	12397	2277	0
BRA	Brazil	8278	Jesuania	0.28	0.67	0.92	2524	621	0
BRA	Brazil	8279	Joaima	0.31	0.69	0.92	7630	1753	0
BRA	Brazil	8280	Joanesia	0.54	0.77	0.96	1281	141	0
BRA	Brazil	8281	Joao Monlevade	0.32	0.78	1.00	37770	1955	0
BRA	Brazil	8282	Joao Pinheiro	0.70	0.89	1.00	4723	0	0
BRA	Brazil	8283	Joaquim Felicio	0.41	0.76	0.97	1829	186	0
BRA	Brazil	8284	Jordania	0.29	0.68	0.93	5892	1402	0
BRA	Brazil	8285	Jose Goncalves De Minas	0.44	0.81	1.00	1695	0	0
BRA	Brazil	8286	Jose Raydan	0.33	0.78	1.00	2295	100	0
BRA	Brazil	8287	Josenopolis	0.41	0.76	0.98	1900	186	0
BRA	Brazil	8288	Juatuba	0.33	0.77	0.98	12357	758	0
BRA	Brazil	8289	Juiz De Fora	0.27	0.71	0.96	298616	51478	0
BRA	Brazil	8290	Juramento	0.38	0.76	0.98	1810	196	0
BRA	Brazil	8291	Juruaia	0.55	0.79	0.97	2525	96	0
BRA	Brazil	8292	Juvenilia	0.37	0.76	0.98	2768	233	0
BRA	Brazil	8293	Ladainha	0.31	0.74	0.99	8801	1081	0
BRA	Brazil	8294	Lagamar	0.59	0.82	0.95	1619	0	0

BRA	Brazil	8295	Lagoa Da Prata	0.33	0.75	0.96	24270	2504	0
BRA	Brazil	8296	Lagoa Dos Patos	0.45	0.77	0.98	1501	124	0
BRA	Brazil	8297	Lagoa Dourada	0.49	0.77	0.98	4087	426	0
BRA	Brazil	8298	Lagoa Formosa	0.45	0.77	0.96	6376	579	0
BRA	Brazil	8299	Lagoa Grande	0.54	0.81	0.97	2406	0	0
BRA	Brazil	8300	Lagoa Santa	0.28	0.72	0.96	34798	5557	0
BRA	Brazil	8301	Lajinha	0.30	0.68	0.91	10217	2507	0
BRA	Brazil	8302	Lambari	0.34	0.75	0.98	9451	971	0
BRA	Brazil	8303	Lamim	0.61	0.83	0.98	666	0	0
BRA	Brazil	8304	Laranjal	0.67	0.90	1.00	864	0	0
BRA	Brazil	8305	Lassance	0.68	0.87	0.98	816	0	0
BRA	Brazil	8306	Lavras	0.28	0.73	0.97	53419	7431	0
BRA	Brazil	8307	Leandro Ferreira	0.84	0.94	1.00	0	0	0
BRA	Brazil	8308	Leme Do Prado	0.43	0.80	1.00	1805	0	0
BRA	Brazil	8309	Leopoldina	0.31	0.74	0.97	26349	3393	0
BRA	Brazil	8310	Liberdade	0.28	0.70	0.96	2760	509	0
BRA	Brazil	8311	Lima Duarte	0.29	0.70	0.96	8687	1691	0
BRA	Brazil	8312	Limeira Do Oeste	0.49	0.84	0.99	2308	0	0
BRA	Brazil	8313	Lontra	0.36	0.74	0.97	4052	543	0
BRA	Brazil	8314	Luisburgo	0.60	0.87	1.00	1310	0	0
BRA	Brazil	8315	Luislandia	0.85	0.93	0.99	0	0	0
BRA	Brazil	8316	Luminarias	0.29	0.67	0.91	2862	725	0
BRA	Brazil	8317	Luz	0.43	0.83	0.97	6863	0	0
BRA	Brazil	8318	Machacalis	0.32	0.79	0.99	3439	71	0
BRA	Brazil	8319	Machado	0.34	0.75	0.99	19566	2000	0
BRA	Brazil	8320	Madre De Deus De Minas	0.31	0.68	0.92	2528	608	0
BRA	Brazil	8321	Malacacheta	0.30	0.66	0.86	9402	2644	0
BRA	Brazil	8322	Mamonas	0.43	0.78	0.97	2397	125	0
BRA	Brazil	8323	Manga	0.37	0.76	1.00	8704	859	0
BRA	Brazil	8324	Manhuacu	0.31	0.71	0.97	43615	7685	0
BRA	Brazil	8325	Manhumirim	0.29	0.66	0.88	11746	3219	0
BRA	Brazil	8326	Mantena	0.29	0.72	0.98	14478	2248	0
BRA	Brazil	8327	Mar De Espanha	0.29	0.68	0.94	6511	1497	0

BRA	Brazil	8328	Maravilhas	0.52	0.82	0.98	2224	0	0
BRA	Brazil	8329	Maria Da Fe	0.65	0.84	0.99	2214	0	0
BRA	Brazil	8330	Mariana	0.30	0.77	1.00	30290	1977	0
BRA	Brazil	8331	Marilac	0.32	0.74	0.99	2047	261	0
BRA	Brazil	8332	Mario Campos	0.32	0.73	0.99	6755	962	0
BRA	Brazil	8333	Maripa De Minas	0.30	0.71	0.96	1477	265	0
BRA	Brazil	8334	Marlieria	0.30	0.68	0.93	2189	508	0
BRA	Brazil	8335	Marmelopolis	0.32	0.69	0.96	1403	308	0
BRA	Brazil	8336	Martinho Campos	0.37	0.78	0.97	5791	308	0
BRA	Brazil	8337	Martins Soares	0.29	0.68	0.93	4409	1037	0
BRA	Brazil	8338	Mata Verde	0.33	0.73	0.98	3478	517	0
BRA	Brazil	8339	Materlandia	0.30	0.67	0.88	2315	619	0
BRA	Brazil	8340	Mateus Leme	0.30	0.74	0.98	15304	1975	0
BRA	Brazil	8341	Mathias Lobato	0.32	0.74	0.99	1700	226	0
BRA	Brazil	8342	Matias Barbosa	0.30	0.71	0.96	7486	1316	0
BRA	Brazil	8343	Matias Cardoso	0.34	0.75	0.99	5120	613	0
BRA	Brazil	8344	Matipo	0.30	0.70	0.96	9661	1962	0
BRA	Brazil	8345	Mato Verde	0.37	0.73	0.98	5479	845	0
BRA	Brazil	8346	Matozinhos	0.37	0.77	0.99	16070	1252	0
BRA	Brazil	8347	Matutina	0.39	0.75	0.98	1590	177	0
BRA	Brazil	8348	Medeiros	0.42	0.74	0.96	1432	221	0
BRA	Brazil	8349	Medina	0.31	0.69	0.93	10399	2270	0
BRA	Brazil	8350	Mendes Pimentel	0.36	0.77	0.95	2872	194	0
BRA	Brazil	8351	Merces	0.34	0.70	0.94	5025	1104	0
BRA	Brazil	8352	Mesquita	0.31	0.76	1.00	2940	257	0
BRA	Brazil	8353	Minas Novas	0.33	0.73	0.98	15374	2181	0
BRA	Brazil	8354	Minduri	0.39	0.79	0.98	1642	29	0
BRA	Brazil	8355	Mirabela	0.40	0.77	0.99	5520	424	0
BRA	Brazil	8356	Miradouro	0.35	0.72	0.97	4917	859	0
BRA	Brazil	8357	Mirai	0.30	0.76	0.96	7537	648	0
BRA	Brazil	8358	Miravania	0.38	0.79	1.00	2031	36	0
BRA	Brazil	8359	Moeda	0.39	0.78	0.99	1881	83	0
BRA	Brazil	8360	Moema	0.34	0.77	0.99	3429	216	0

BRA	Brazil	8361	Monjolos	0.30	0.70	0.97	1164	225	0
BRA	Brazil	8362	Monsenhor Paulo	0.46	0.77	0.97	2995	224	0
BRA	Brazil	8363	Montalvania	0.38	0.80	1.00	6069	51	0
BRA	Brazil	8364	Monte Alegre De Minas	0.36	0.73	0.95	9332	1494	0
BRA	Brazil	8365	Monte Azul	0.49	0.80	0.98	6971	0	0
BRA	Brazil	8366	Monte Belo	0.32	0.77	0.97	6507	363	0
BRA	Brazil	8367	Monte Carmelo	0.42	0.82	0.99	18559	0	0
BRA	Brazil	8368	Monte Formoso	0.34	0.76	0.99	2275	214	0
BRA	Brazil	8369	Monte Santo De Minas	0.36	0.78	0.98	9582	397	0
BRA	Brazil	8370	Monte Siao	0.27	0.69	0.96	12249	2558	0
BRA	Brazil	8371	Montes Claros	0.37	0.73	0.98	173268	26547	0
BRA	Brazil	8372	Montezuma	0.38	0.75	0.99	3475	406	0
BRA	Brazil	8373	Morada Nova De Minas	0.46	0.82	0.98	3012	0	0
BRA	Brazil	8374	Morro Da Garca	0.40	0.81	0.99	1024	0	0
BRA	Brazil	8375	Morro Do Pilar	0.38	0.82	0.99	1422	0	0
BRA	Brazil	8376	Munhoz	0.30	0.70	0.96	3145	649	0
BRA	Brazil	8377	Muriae	0.30	0.71	0.97	54879	9431	0
BRA	Brazil	8378	Mutum	0.30	0.71	0.97	13704	2459	0
BRA	Brazil	8379	Muzambinho	0.31	0.75	0.98	10418	1139	0
BRA	Brazil	8380	Nacip Raydan	0.43	0.76	0.98	1202	140	0
BRA	Brazil	8381	Nanuque	0.31	0.71	0.97	20916	3767	0
BRA	Brazil	8382	Naque	0.33	0.81	1.00	3021	0	0
BRA	Brazil	8383	Natalandia	0.96	0.97	0.99	0	0	0
BRA	Brazil	8384	Natercia	0.47	0.76	0.96	1629	194	0
BRA	Brazil	8385	Nazareno	0.38	0.75	0.98	3586	427	0
BRA	Brazil	8386	Nepomuceno	0.32	0.75	0.98	13114	1243	0
BRA	Brazil	8387	Ninheira	0.33	0.75	0.98	5088	587	0
BRA	Brazil	8388	Nova Belem	0.36	0.77	0.99	1529	90	0
BRA	Brazil	8389	Nova Era	0.41	0.80	0.99	7206	45	0
BRA	Brazil	8390	Nova Lima	0.30	0.76	0.99	47358	3354	0
BRA	Brazil	8391	Nova Modica	0.31	0.70	0.94	1868	392	0
BRA	Brazil	8392	Nova Ponte	0.44	0.78	0.98	5503	313	0
BRA	Brazil	8393	Nova Porteirinha	0.36	0.73	0.97	4356	681	0

BRA	Brazil	8394	Nova Resende	0.36	0.81	0.99	7306	0	0
BRA	Brazil	8395	Nova Serrana	0.28	0.67	0.92	55876	13943	0
BRA	Brazil	8396	Nova Uniao	0.56	0.90	1.00	1347	0	0
BRA	Brazil	8397	Novo Cruzeiro	0.33	0.75	0.99	14967	1747	0
BRA	Brazil	8398	Novo Oriente De Minas	0.31	0.73	0.98	5301	788	0
BRA	Brazil	8399	Novorizonte	0.31	0.72	0.98	2437	398	0
BRA	Brazil	8400	Olaria	0.47	0.83	0.98	622	0	0
BRA	Brazil	8401	Olhos-d'agua	0.31	0.72	0.98	2929	487	0
BRA	Brazil	8402	Olimpio Noronha	0.36	0.77	0.99	1220	71	0
BRA	Brazil	8403	Oliveira	0.27	0.71	0.96	22197	3817	0
BRA	Brazil	8404	Oliveira Fortes	0.32	0.70	0.95	1050	224	0
BRA	Brazil	8405	Onca De Pitangui	0.53	0.82	0.99	886	0	0
BRA	Brazil	8406	Oratorios	0.38	0.78	0.99	1978	102	0
BRA	Brazil	8407	Orizania	0.31	0.69	0.93	3911	899	0
BRA	Brazil	8408	Ouro Branco	0.17	0.49	0.65	23538	11502	5616
BRA	Brazil	8409	Ouro Fino	0.27	0.67	0.93	17913	4523	0
BRA	Brazil	8410	Ouro Preto	0.31	0.78	1.00	36751	1674	0
BRA	Brazil	8411	Ouro Verde De Minas	0.33	0.74	1.00	2860	353	0
BRA	Brazil	8412	Padre Carvalho	0.41	0.78	0.99	2453	140	0
BRA	Brazil	8413	Padre Paraiso	0.30	0.71	0.97	9665	1828	0
BRA	Brazil	8414	Pai Pedro	0.31	0.71	0.98	3050	536	0
BRA	Brazil	8415	Paineiras	0.48	0.78	0.95	1505	101	0
BRA	Brazil	8416	Pains	0.49	0.81	0.96	2642	0	0
BRA	Brazil	8417	Paiva	0.39	0.75	0.95	641	86	0
BRA	Brazil	8418	Palma	0.31	0.73	0.95	3318	485	0
BRA	Brazil	8419	Palmopolis	0.45	0.85	0.99	2188	0	0
BRA	Brazil	8420	Papagaios	0.38	0.81	0.99	6544	0	0
BRA	Brazil	8421	Para De Minas	0.36	0.83	0.97	40877	0	0
BRA	Brazil	8422	Paracatu	0.57	0.83	0.98	21493	0	0
BRA	Brazil	8423	Paraguacu	0.36	0.76	0.98	9444	820	0
BRA	Brazil	8424	Paraisopolis	0.29	0.69	0.94	10781	2390	0
BRA	Brazil	8425	Paraopeba	0.42	0.80	0.98	9281	0	0
BRA	Brazil	8426	Passa Quatro	0.29	0.69	0.95	8439	1871	0

BRA	Brazil	8427	Passa Tempo	0.42	0.77	0.95	3202	216	0
BRA	Brazil	8428	Passa Vinte	0.41	0.76	0.96	835	77	0
BRA	Brazil	8429	Passabem	0.97	1.00	1.00	0	0	0
BRA	Brazil	8430	Passos	0.32	0.73	0.98	54921	8590	0
BRA	Brazil	8431	Patis	0.34	0.73	0.99	2787	394	0
BRA	Brazil	8432	Patos De Minas	0.48	0.81	0.99	47940	0	0
BRA	Brazil	8433	Patrocinio	0.39	0.77	0.99	36875	2461	0
BRA	Brazil	8434	Patrocinio Do Muriae	0.32	0.72	0.97	2706	424	0
BRA	Brazil	8435	Paula Candido	0.28	0.66	0.88	5004	1382	0
BRA	Brazil	8436	Paulistas	0.53	0.85	1.00	1344	0	0
BRA	Brazil	8437	Pavao	0.30	0.65	0.84	4460	1358	0
BRA	Brazil	8438	Pecanha	0.31	0.73	0.98	8907	1346	0
BRA	Brazil	8439	Pedra Azul	0.32	0.71	0.97	11979	2131	0
BRA	Brazil	8440	Pedra Bonita	0.44	0.79	0.99	2368	58	0
BRA	Brazil	8441	Pedra Do Anta	0.28	0.64	0.82	1690	541	0
BRA	Brazil	8442	Pedra Do Indaia	0.40	0.77	0.95	1674	133	0
BRA	Brazil	8443	Pedra Dourada	0.51	0.76	0.97	721	98	0
BRA	Brazil	8444	Pedralva	0.31	0.74	0.97	5602	659	0
BRA	Brazil	8445	Pedras De Maria Da Cruz	0.33	0.73	0.99	5761	887	0
BRA	Brazil	8446	Pedrinopolis	0.55	0.85	0.99	902	0	0
BRA	Brazil	8447	Pedro Leopoldo	0.30	0.72	0.97	33054	5545	0
BRA	Brazil	8448	Pedro Teixeira	0.30	0.72	0.98	913	149	0
BRA	Brazil	8449	Pequeri	0.35	0.77	0.98	1529	96	0
BRA	Brazil	8450	Pequi	0.46	0.80	0.97	1519	12	0
BRA	Brazil	8451	Perdigao	0.35	0.74	0.98	5185	693	0
BRA	Brazil	8452	Perdizes	0.35	0.73	0.96	7166	1054	0
BRA	Brazil	8453	Perdoes	0.39	0.74	0.97	8910	1221	0
BRA	Brazil	8454	Periquito	0.32	0.70	0.95	3134	626	0
BRA	Brazil	8455	Pescador	0.32	0.75	0.99	2087	210	0
BRA	Brazil	8456	Piau	0.32	0.70	0.93	1349	292	0
BRA	Brazil	8457	Piedade De Caratinga	0.32	0.73	0.98	4253	591	0
BRA	Brazil	8458	Piedade De Ponte Nova	0.34	0.73	0.98	1950	309	0
BRA	Brazil	8459	Piedade Do Rio Grande	0.40	0.78	0.96	1861	113	0

BRA	Brazil	8460	Piedade Dos Gerais	0.29	0.70	0.95	2538	487	0
BRA	Brazil	8461	Pimenta	0.38	0.81	0.97	3685	0	0
BRA	Brazil	8462	Pingo D'agua	0.33	0.72	0.97	2217	359	0
BRA	Brazil	8463	Pintopolis	0.42	0.78	0.99	2917	124	0
BRA	Brazil	8464	Piracema	0.45	0.78	0.96	2287	110	0
BRA	Brazil	8465	Pirajuba	0.40	0.75	0.97	2524	332	0
BRA	Brazil	8466	Piranga	0.41	0.72	0.96	6932	1344	0
BRA	Brazil	8467	Pirangucu	0.28	0.67	0.93	2985	728	0
BRA	Brazil	8468	Piranguinho	0.39	0.80	1.00	3168	0	0
BRA	Brazil	8469	Pirapetinga	0.30	0.71	0.98	5320	918	0
BRA	Brazil	8470	Pirapora	0.46	0.79	1.00	18974	313	0
BRA	Brazil	8471	Pirauba	0.31	0.68	0.93	5600	1401	0
BRA	Brazil	8472	Pitangui	0.38	0.79	0.99	11683	320	0
BRA	Brazil	8473	Piui	0.39	0.77	0.99	14288	1123	0
BRA	Brazil	8474	Planura	0.42	0.77	0.98	4603	365	0
BRA	Brazil	8475	Poco Fundo	0.28	0.69	0.94	8812	1932	0
BRA	Brazil	8476	Pocos De Caldas	0.29	0.70	0.96	85444	16055	0
BRA	Brazil	8477	Pocrane	0.29	0.68	0.93	4614	1080	0
BRA	Brazil	8478	Pompeu	0.37	0.80	0.99	13610	18	0
BRA	Brazil	8479	Ponte Nova	0.29	0.71	0.96	30566	5409	0
BRA	Brazil	8480	Ponto Chique	0.69	0.85	0.98	484	0	0
BRA	Brazil	8481	Ponto Dos Volantes	0.35	0.77	0.99	5504	424	0
BRA	Brazil	8482	Porteirinha	0.37	0.78	0.99	16483	743	0
BRA	Brazil	8483	Porto Firme	0.27	0.65	0.90	6017	1648	0
BRA	Brazil	8484	Pote	0.30	0.70	0.96	8441	1660	0
BRA	Brazil	8485	Pouso Alegre	0.27	0.69	0.96	78927	16433	0
BRA	Brazil	8486	Pouso Alto	0.37	0.71	0.95	2733	568	0
BRA	Brazil	8487	Prados	0.38	0.75	0.96	3839	476	0
BRA	Brazil	8488	Prata	0.40	0.78	0.99	11132	428	0
BRA	Brazil	8489	Pratapolis	0.28	0.69	0.96	4584	943	0
BRA	Brazil	8490	Pratinha	0.36	0.70	0.95	1586	342	0
BRA	Brazil	8491	Presidente Bernardes	0.80	0.99	1.00	1	0	0
BRA	Brazil	8492	Presidente Juscelino	0.17	0.49	0.65	2375	1160	567

BRA	Brazil	8493	Presidente Kubitschek	0.43	0.84	1.00	1139	0	0
BRA	Brazil	8494	Presidente Olegario	0.41	0.74	0.95	7934	1226	0
BRA	Brazil	8495	Prudente De Morais	0.35	0.77	0.98	4656	324	0
BRA	Brazil	8496	Quartel Geral	0.40	0.75	0.96	1424	178	0
BRA	Brazil	8497	Queluzita	0.52	0.76	0.94	564	87	0
BRA	Brazil	8498	Raposos	0.29	0.74	0.97	8354	1050	0
BRA	Brazil	8499	Raul Soares	0.30	0.71	0.95	12110	2233	0
BRA	Brazil	8500	Recreio	0.28	0.72	0.95	5542	877	0
BRA	Brazil	8501	Reduto	0.30	0.69	0.93	3635	818	0
BRA	Brazil	8502	Resende Costa	0.51	0.80	0.98	3337	44	0
BRA	Brazil	8503	Resplendor	0.30	0.77	0.99	8800	603	0
BRA	Brazil	8504	Ressaquinha	0.62	0.81	0.96	902	0	0
BRA	Brazil	8505	Riachinho	0.79	0.93	1.00	53	0	0
BRA	Brazil	8506	Riacho Dos Machados	0.49	0.83	0.99	3051	0	0
BRA	Brazil	8507	Ribeirao Das Neves	0.30	0.72	0.99	161839	24871	0
BRA	Brazil	8508	Ribeirao Vermelho	0.71	0.86	0.98	311	0	0
BRA	Brazil	8509	Rio Acima	0.34	0.81	0.99	4759	0	0
BRA	Brazil	8510	Rio Casca	0.36	0.76	0.99	6189	506	0
BRA	Brazil	8511	Rio Do Prado	0.47	0.82	0.99	1759	0	0
BRA	Brazil	8512	Rio Doce	0.31	0.69	0.93	1287	289	0
BRA	Brazil	8513	Rio Espera	0.46	0.75	0.94	2007	314	0
BRA	Brazil	8514	Rio Manso	0.37	0.72	0.93	2521	454	0
BRA	Brazil	8515	Rio Novo	0.30	0.73	0.98	4535	592	0
BRA	Brazil	8516	Rio Paranaiba	0.36	0.74	0.96	5746	788	0
BRA	Brazil	8517	Rio Pardo De Minas	0.30	0.71	0.97	15643	2859	0
BRA	Brazil	8518	Rio Piracicaba	0.29	0.70	0.96	7311	1428	0
BRA	Brazil	8519	Rio Pomba	0.26	0.63	0.86	9783	2992	0
BRA	Brazil	8520	Rio Preto	0.40	0.77	0.99	1909	140	0
BRA	Brazil	8521	Rio Vermelho	0.32	0.73	0.98	6456	944	0
BRA	Brazil	8522	Ritapolis	0.49	0.78	0.96	1489	103	0
BRA	Brazil	8523	Rochedo De Minas	0.33	0.73	0.98	1080	162	0
BRA	Brazil	8524	Rodeiro	0.39	0.76	0.95	3161	296	0
BRA	Brazil	8525	Romaria	0.60	0.83	0.97	742	0	0

BRA	Brazil	8526	Rosario Da Limeira	0.37	0.73	0.97	1957	321	0
BRA	Brazil	8527	Rubelita	0.31	0.69	0.91	3439	804	0
BRA	Brazil	8528	Rubim	0.34	0.75	0.98	4779	508	0
BRA	Brazil	8529	Sabara	0.29	0.71	0.97	73671	12305	0
BRA	Brazil	8530	Sabinopolis	0.49	0.84	1.00	4927	0	0
BRA	Brazil	8531	Sacramento	0.33	0.76	0.97	12195	1147	0
BRA	Brazil	8532	Salinas	0.32	0.73	0.99	20208	2998	0
BRA	Brazil	8533	Salto Da Divisa	0.43	0.77	0.99	2610	206	0
BRA	Brazil	8534	Santa Barbara	0.17	0.49	0.65	19816	9683	4728
BRA	Brazil	8535	Santa Barbara Do Leste	0.37	0.77	1.00	3518	268	0
BRA	Brazil	8536	Santa Barbara Do Monte Verde	0.27	0.68	0.94	1644	386	0
BRA	Brazil	8537	Santa Barbara Do Tugurio	0.62	0.83	0.98	704	0	0
BRA	Brazil	8538	Santa Cruz De Minas	0.38	0.80	1.00	2726	0	0
BRA	Brazil	8539	Santa Cruz De Salinas	0.40	0.81	1.00	1618	0	0
BRA	Brazil	8540	Santa Cruz Do Escalvado	0.37	0.80	0.96	2117	0	0
BRA	Brazil	8541	Santa Efigenia De Minas	0.39	0.78	1.00	1931	81	0
BRA	Brazil	8542	Santa Fe De Minas	0.68	0.86	0.99	485	0	0
BRA	Brazil	8543	Santa Helena De Minas	0.35	0.78	1.00	2878	132	0
BRA	Brazil	8544	Santa Juliana	0.36	0.78	0.98	6051	333	0
BRA	Brazil	8545	Santa Luzia	0.17	0.49	0.65	130973	63998	31247
BRA	Brazil	8546	Santa Margarida	0.49	0.80	0.99	5055	0	0
BRA	Brazil	8547	Santa Maria De Itabira	0.39	0.78	0.99	4417	200	0
BRA	Brazil	8548	Santa Maria Do Salto	0.48	0.76	0.96	1751	214	0
BRA	Brazil	8549	Santa Maria Do Suacui	0.34	0.78	0.99	6887	350	0
BRA	Brazil	8550	Santa Rita De Caldas	0.38	0.77	0.97	3844	315	0
BRA	Brazil	8551	Santa Rita De Ibitipoca	0.50	0.75	0.96	1083	168	0
BRA	Brazil	8552	Santa Rita De Jacutinga	0.39	0.75	0.97	2056	266	0
BRA	Brazil	8553	Santa Rita De Minas	0.32	0.71	0.95	3582	694	0
BRA	Brazil	8554	Santa Rita Do Itueto	0.34	0.75	0.99	2651	312	0
BRA	Brazil	8555	Santa Rita Do Sapucai	0.35	0.76	0.98	19457	1836	0
BRA	Brazil	8556	Santa Rosa Da Serra	0.32	0.71	0.95	1630	308	0
BRA	Brazil	8557	Santa Vitoria	0.40	0.76	0.96	7931	847	0
BRA	Brazil	8558	Santana Da Vargem	0.39	0.77	0.98	3017	226	0

BRA	Brazil	8559	Santana De Cataguases	0.52	0.79	0.96	1080	40	0
BRA	Brazil	8560	Santana De Pirapama	0.44	0.80	0.98	2732	0	0
BRA	Brazil	8561	Santana Do Deserto	0.30	0.71	0.97	2358	420	0
BRA	Brazil	8562	Santana Do Garambeu	0.28	0.64	0.85	1273	390	0
BRA	Brazil	8563	Santana Do Jacare	0.33	0.72	0.96	1892	303	0
BRA	Brazil	8564	Santana Do Manhuacu	0.29	0.65	0.86	4540	1311	0
BRA	Brazil	8565	Santana Do Paraiso	0.27	0.68	0.95	19547	4562	0
BRA	Brazil	8566	Santana Do Riacho	0.43	0.78	0.98	1544	94	0
BRA	Brazil	8567	Santana Dos Montes	0.45	0.74	0.96	1393	253	0
BRA	Brazil	8568	Santo Antonio Do Amparo	0.30	0.73	0.97	9192	1311	0
BRA	Brazil	8569	Santo Antonio Do Aventureiro	0.34	0.69	0.94	1660	398	0
BRA	Brazil	8570	Santo Antonio Do Grama	0.35	0.75	0.98	1837	194	0
BRA	Brazil	8571	Santo Antonio Do Itambe	0.55	0.86	0.99	1007	0	0
BRA	Brazil	8572	Santo Antonio Do Jacinto	0.44	0.79	1.00	3936	80	0
BRA	Brazil	8573	Santo Antonio Do Monte	0.31	0.76	0.99	13875	1021	0
BRA	Brazil	8574	Santo Antonio Do Retiro	0.44	0.82	1.00	2624	0	0
BRA	Brazil	8575	Santo Antonio Do Rio Abaixo	0.37	0.72	0.97	765	142	0
BRA	Brazil	8576	Santo Hipolito	0.36	0.75	0.97	1418	161	0
BRA	Brazil	8577	Santos Dumont	0.32	0.72	0.97	22810	3823	0
BRA	Brazil	8578	Sao Bento Abade	0.67	0.85	0.99	691	0	0
BRA	Brazil	8579	Sao Bras Do Suacui	0.39	0.76	0.96	1602	157	0
BRA	Brazil	8580	Sao Domingos Das Dores	0.39	0.80	1.00	2296	0	0
BRA	Brazil	8581	Sao Domingos Do Prata	0.30	0.77	1.00	8870	449	0
BRA	Brazil	8582	Sao Felix De Minas	0.41	0.83	0.99	1358	0	0
BRA	Brazil	8583	Sao Francisco	0.37	0.81	1.00	24331	0	0
BRA	Brazil	8584	Sao Francisco De Paula	0.98	0.99	1.00	0	0	0
BRA	Brazil	8585	Sao Francisco De Sales	0.39	0.76	0.97	2542	281	0
BRA	Brazil	8586	Sao Francisco Do Gloria	0.36	0.69	0.91	2495	624	0
BRA	Brazil	8587	Sao Geraldo	0.25	0.65	0.90	6895	1883	0
BRA	Brazil	8588	Sao Geraldo Da Piedade	0.35	0.77	0.99	1969	120	0
BRA	Brazil	8589	Sao Geraldo Do Baixio	0.34	0.70	0.93	1809	409	0
BRA	Brazil	8590	Sao Goncalo Do Abaete	0.43	0.74	0.95	3085	492	0
BRA	Brazil	8591	Sao Goncalo Do Para	0.29	0.73	0.97	6243	811	0

BRA	Brazil	8592	Sao Goncalo Do Rio Abaixo	0.34	0.76	0.99	5124	401	0
BRA	Brazil	8593	Sao Goncalo Do Rio Preto	0.54	0.82	0.99	818	0	0
BRA	Brazil	8594	Sao Goncalo Do Sapucai	0.39	0.75	0.99	10361	1287	0
BRA	Brazil	8595	Sao Gotardo	0.41	0.79	1.00	13295	236	0
BRA	Brazil	8596	Sao Joao Batista Do Gloria	0.44	0.80	0.97	2729	0	0
BRA	Brazil	8597	Sao Joao Da Lagoa	0.39	0.81	0.98	2036	0	0
BRA	Brazil	8598	Sao Joao Da Mata	0.41	0.73	0.97	1088	200	0
BRA	Brazil	8599	Sao Joao Da Ponte	0.41	0.81	1.00	10040	0	0
BRA	Brazil	8600	Sao Joao Das Missoes	0.35	0.84	1.00	5473	0	0
BRA	Brazil	8601	Sao Joao Del Rei	0.28	0.71	0.96	47967	8715	0
BRA	Brazil	8602	Sao Joao Do Manhuacu	0.37	0.76	0.98	4945	498	0
BRA	Brazil	8603	Sao Joao Do Manteninha	0.33	0.75	1.00	2570	257	0
BRA	Brazil	8604	Sao Joao Do Oriente	0.34	0.79	1.00	3569	67	0
BRA	Brazil	8605	Sao Joao Do Pacui	0.48	0.82	0.98	1400	0	0
BRA	Brazil	8606	Sao Joao Do Paraiso	0.17	0.49	0.65	14954	7307	3568
BRA	Brazil	8607	Sao Joao Evangelista	0.30	0.74	0.99	8021	989	0
BRA	Brazil	8608	Sao Joao Nepomuceno	0.30	0.71	0.96	13265	2294	0
BRA	Brazil	8609	Sao Joaquim De Bicas	0.28	0.70	0.97	17431	3253	0
BRA	Brazil	8610	Sao Jose Da Barra	0.48	0.79	0.98	2270	72	0
BRA	Brazil	8611	Sao Jose Da Lapa	0.30	0.73	0.99	12212	1778	0
BRA	Brazil	8612	Sao Jose Da Safira	0.33	0.76	1.00	1949	179	0
BRA	Brazil	8613	Sao Jose Da Varginha	0.27	0.77	0.96	2636	175	0
BRA	Brazil	8614	Sao Jose Do Alegre	0.29	0.69	0.95	2266	501	0
BRA	Brazil	8615	Sao Jose Do Divino	0.31	0.71	0.96	1929	345	0
BRA	Brazil	8616	Sao Jose Do Goiabal	0.37	0.80	0.99	2378	17	0
BRA	Brazil	8617	Sao Jose Do Jacuri	0.31	0.74	0.99	3260	383	0
BRA	Brazil	8618	Sao Jose Do Mantimento	0.33	0.72	0.98	1282	230	0
BRA	Brazil	8619	Sao Lourenco	0.28	0.70	0.96	23330	4500	0
BRA	Brazil	8620	Sao Miguel Do Anta	0.29	0.72	0.97	3601	565	0
BRA	Brazil	8621	Sao Pedro Da Uniao	0.33	0.76	0.96	2328	201	0
BRA	Brazil	8622	Sao Pedro Do Suacui	0.28	0.68	0.94	2850	651	0
BRA	Brazil	8623	Sao Pedro Dos Ferros	0.29	0.69	0.96	4262	883	0
BRA	Brazil	8624	Sao Romao	0.35	0.72	0.97	5416	932	0

BRA	Brazil	8625	Sao Roque De Minas	0.28	0.68	0.94	3852	859	0
BRA	Brazil	8626	Sao Sebastiao Da Bela Vista	0.36	0.71	0.97	2378	465	0
BRA	Brazil	8627	Sao Sebastiao Da Vargem Alegre	0.47	0.79	0.98	959	17	0
BRA	Brazil	8628	Sao Sebastiao Do Anta	0.31	0.71	0.97	3201	578	0
BRA	Brazil	8629	Sao Sebastiao Do Maranhao	0.27	0.61	0.79	5537	1963	73
BRA	Brazil	8630	Sao Sebastiao Do Oeste	0.40	0.81	0.98	2698	0	0
BRA	Brazil	8631	Sao Sebastiao Do Paraiso	0.32	0.76	0.99	33828	3123	0
BRA	Brazil	8632	Sao Sebastiao Do Rio Preto	0.45	0.79	0.96	549	11	0
BRA	Brazil	8633	Sao Sebastiao Do Rio Verde	0.35	0.72	0.96	958	175	0
BRA	Brazil	8634	Sao Thome Das Letras	0.35	0.74	0.97	3184	430	0
BRA	Brazil	8635	Sao Tiago	0.50	0.80	0.96	3286	53	0
BRA	Brazil	8636	Sao Tomas De Aquino	0.40	0.84	1.00	2871	0	0
BRA	Brazil	8637	Sao Vicente De Minas	0.28	0.68	0.93	3979	953	0
BRA	Brazil	8638	Sapucai-mirim	0.50	0.79	0.98	2109	100	0
BRA	Brazil	8639	Sardoa	0.37	0.78	1.00	2608	122	0
BRA	Brazil	8640	Sarzedo	0.31	0.77	1.00	15364	970	0
BRA	Brazil	8641	Sem-peixe	0.27	0.69	0.94	1468	318	0
BRA	Brazil	8642	Senador Amaral	0.52	0.79	1.00	1523	43	0
BRA	Brazil	8643	Senador Cortes	0.31	0.66	0.85	991	289	0
BRA	Brazil	8644	Senador Firmino	0.40	0.76	0.97	3076	301	0
BRA	Brazil	8645	Senador Jose Bento	0.28	0.68	0.95	904	204	0
BRA	Brazil	8646	Senador Modestino Goncalves	0.66	0.86	0.98	640	0	0
BRA	Brazil	8647	Senhora De Oliveira	0.76	0.88	0.98	256	0	0
BRA	Brazil	8648	Senhora Do Porto	0.35	0.77	0.98	1625	102	0
BRA	Brazil	8649	Senhora Dos Remedios	0.45	0.74	0.96	3689	617	0
BRA	Brazil	8650	Sericita	0.33	0.73	0.99	3466	508	0
BRA	Brazil	8651	Seritinga	0.45	0.76	0.97	658	77	0
BRA	Brazil	8652	Serra Azul De Minas	0.37	0.80	1.00	1889	0	0
BRA	Brazil	8653	Serra Da Saudade	0.44	0.76	0.94	297	33	0
BRA	Brazil	8654	Serra Do Salitre	0.33	0.74	0.98	5489	749	0
BRA	Brazil	8655	Serra Dos Aimores	0.31	0.75	0.99	4116	419	0
BRA	Brazil	8656	Serrania	0.42	0.82	0.99	2963	0	0
BRA	Brazil	8657	Serranopolis De Minas	0.47	0.81	0.98	1583	0	0

BRA	Brazil	8658	Serranos	0.44	0.77	0.97	733	67	0
BRA	Brazil	8659	Serro	0.43	0.83	0.99	7950	0	0
BRA	Brazil	8660	Sete Lagoas	0.30	0.75	0.98	118700	11913	0
BRA	Brazil	8661	Setubinha	0.36	0.74	0.98	3674	523	0
BRA	Brazil	8662	Silveirania	0.47	0.78	0.96	765	36	0
BRA	Brazil	8663	Silvianopolis	0.30	0.67	0.93	3768	991	0
BRA	Brazil	8664	Simao Pereira	0.31	0.73	0.98	1378	191	0
BRA	Brazil	8665	Simonesia	0.31	0.68	0.89	9515	2367	0
BRA	Brazil	8666	Sobralia	0.32	0.75	0.99	2830	287	0
BRA	Brazil	8667	Soledade De Minas	0.27	0.64	0.87	3326	985	0
BRA	Brazil	8668	Tabuleiro	0.28	0.68	0.94	2080	478	0
BRA	Brazil	8669	Taiobeiras	0.32	0.72	0.97	16252	2657	0
BRA	Brazil	8670	Taparuba	0.34	0.73	0.98	1450	218	0
BRA	Brazil	8671	Tapira	0.57	0.92	1.00	1084	0	0
BRA	Brazil	8672	Tapirai	0.98	0.98	0.98	0	0	0
BRA	Brazil	8673	Taquaracu De Minas	0.39	0.74	0.95	1713	268	0
BRA	Brazil	8674	Tarumirim	0.29	0.70	0.97	7584	1423	0
BRA	Brazil	8675	Teixeiras	0.27	0.65	0.89	6265	1756	0
BRA	Brazil	8676	Teofilo Otoni	0.32	0.77	0.99	67642	3701	0
BRA	Brazil	8677	Timoteo	0.29	0.71	0.96	49479	8549	0
BRA	Brazil	8678	Tiradentes	0.40	0.70	0.93	3134	749	0
BRA	Brazil	8679	Tiros	0.38	0.78	0.98	2848	139	0
BRA	Brazil	8680	Tocantins	0.37	0.74	0.97	7367	1024	0
BRA	Brazil	8681	Tocos Do Moji	0.44	0.74	0.95	1480	258	0
BRA	Brazil	8682	Toledo	1.00	1.00	1.00	0	0	0
BRA	Brazil	8683	Tombos	0.28	0.67	0.91	4615	1179	0
BRA	Brazil	8684	Tres Coracoes	0.29	0.73	0.98	39999	5931	0
BRA	Brazil	8685	Tres Marias	0.48	0.78	0.97	10384	637	0
BRA	Brazil	8686	Tres Pontas	0.33	0.77	0.98	26902	1710	0
BRA	Brazil	8687	Tumiritinga	0.31	0.74	0.99	3357	401	0
BRA	Brazil	8688	Tupaciguara	0.48	0.78	0.97	8216	520	0
BRA	Brazil	8689	Turmalina	0.35	0.82	1.00	8627	0	0
BRA	Brazil	8690	Turvolandia	0.53	0.78	0.98	1343	88	0

BRA	Brazil	8691	Uba	0.51	0.80	0.99	32725	0	0
BRA	Brazil	8692	Ubai	0.40	0.79	1.00	5044	170	0
BRA	Brazil	8693	Ubaporanga	0.29	0.68	0.93	6391	1453	0
BRA	Brazil	8694	Uberaba	0.40	0.77	0.97	133358	11075	0
BRA	Brazil	8695	Uberlandia	0.35	0.79	0.99	308254	6309	0
BRA	Brazil	8696	Umburatiba	0.42	0.85	1.00	858	0	0
BRA	Brazil	8697	Unai	0.62	0.84	0.98	15329	0	0
BRA	Brazil	8698	Uniao De Minas	0.50	0.85	0.99	1341	0	0
BRA	Brazil	8699	Uruana De Minas	0.70	0.88	1.00	327	0	0
BRA	Brazil	8700	Urucania	0.38	0.78	0.99	4430	211	0
BRA	Brazil	8701	Urucuia	0.34	0.72	0.95	7399	1228	0
BRA	Brazil	8702	Vargem Alegre	0.34	0.77	1.00	3054	203	0
BRA	Brazil	8703	Vargem Bonita	0.49	0.81	0.99	584	0	0
BRA	Brazil	8704	Vargem Grande Do Rio Pardo	0.51	0.85	0.99	1498	0	0
BRA	Brazil	8705	Varginha	0.30	0.72	0.96	66994	10831	0
BRA	Brazil	8706	Varjao De Minas	0.50	0.84	1.00	1851	0	0
BRA	Brazil	8707	Varzea Da Palma	0.50	0.82	0.99	11516	0	0
BRA	Brazil	8708	Varzelandia	0.39	0.79	1.00	8022	208	0
BRA	Brazil	8709	Vazante	0.49	0.79	0.97	6431	249	0
BRA	Brazil	8710	Verdelandia	0.41	0.77	0.99	3824	248	0
BRA	Brazil	8711	Veredinha	0.40	0.75	0.96	2366	322	0
BRA	Brazil	8712	Verissimo	0.38	0.72	0.96	1688	340	0
BRA	Brazil	8713	Vermelho Novo	0.37	0.80	0.99	2103	0	0
BRA	Brazil	8714	Vespasiano	0.30	0.71	0.98	59266	10694	0
BRA	Brazil	8715	Vicosa	0.17	0.49	0.65	49472	24174	11803
BRA	Brazil	8716	Vieiras	0.28	0.68	0.94	1899	437	0
BRA	Brazil	8717	Virgem Da Lapa	0.30	0.71	0.96	7119	1305	0
BRA	Brazil	8718	Virginia	0.47	0.79	0.97	2941	101	0
BRA	Brazil	8719	Virginopolis	0.42	0.81	0.98	4057	0	0
BRA	Brazil	8720	Virgolandia	0.30	0.74	0.98	2787	361	0
BRA	Brazil	8721	Visconde Do Rio Branco	0.30	0.73	0.96	21017	3131	0
BRA	Brazil	8722	Volta Grande	0.26	0.62	0.82	2936	998	0
BRA	Brazil	8723	Wenceslau Braz	1.00	1.00	1.00	0	0	0

BRA	Brazil	8724	Abaetetuba	0.35	0.83	0.97	69819	0	0
BRA	Brazil	8725	Abel Figueiredo	0.36	0.83	0.96	4726	0	0
BRA	Brazil	8726	Acara	0.36	0.87	1.00	25098	0	0
BRA	Brazil	8727	Afua	0.36	0.86	0.99	16977	0	0
BRA	Brazil	8728	Agua Azul Do Norte	0.32	0.81	0.95	14237	0	0
BRA	Brazil	8729	Alenquer	0.34	0.82	0.94	26993	0	0
BRA	Brazil	8730	Almeirim	0.37	0.89	1.00	19617	0	0
BRA	Brazil	8731	Altamira	0.37	0.91	1.00	52389	0	0
BRA	Brazil	8732	Anajas	0.35	0.83	0.95	13363	0	0
BRA	Brazil	8733	Ananindeua	0.34	0.83	0.96	249365	0	0
BRA	Brazil	8734	Anapu	0.33	0.85	1.00	14701	0	0
BRA	Brazil	8735	Augusto Correa	0.35	0.83	0.96	22327	0	0
BRA	Brazil	8736	Aurora Do Para	0.34	0.82	0.94	14719	0	0
BRA	Brazil	8737	Aveiro	0.34	0.87	1.00	7572	0	0
BRA	Brazil	8738	Bagre	0.31	0.79	0.91	15840	439	0
BRA	Brazil	8739	Baiao	0.33	0.82	0.96	20591	0	0
BRA	Brazil	8740	Bannach	0.40	0.89	1.00	1363	0	0
BRA	Brazil	8741	Barcarena	0.35	0.86	0.98	58641	0	0
BRA	Brazil	8742	Belem	0.21	0.66	0.75	873286	213298	68955
BRA	Brazil	8743	Belterra	0.38	0.90	1.00	7093	0	0
BRA	Brazil	8744	Benevides	0.35	0.84	0.98	26042	0	0
BRA	Brazil	8745	Bom Jesus Do Tocantins	0.40	0.89	1.00	6791	0	0
BRA	Brazil	8746	Bonito	0.21	0.66	0.75	9868	2410	779
BRA	Brazil	8747	Braganca	0.35	0.84	0.97	57549	0	0
BRA	Brazil	8748	Brasil Novo	0.45	0.91	1.00	5904	0	0
BRA	Brazil	8749	Brejo Grande Do Araguaia	0.42	0.84	0.97	2913	0	0
BRA	Brazil	8750	Breu Branco	0.31	0.81	0.95	35718	0	0
BRA	Brazil	8751	Breves	0.37	0.87	0.99	44070	0	0
BRA	Brazil	8752	Bujaru	0.35	0.84	0.97	12992	0	0
BRA	Brazil	8753	Cachoeira Do Arari	0.31	0.80	0.95	11944	0	0
BRA	Brazil	8754	Cachoeira Do Piria	0.30	0.82	0.97	18561	0	0
BRA	Brazil	8755	Cameta	0.35	0.84	0.97	62199	0	0
BRA	Brazil	8756	Canaa Dos Carajas	0.35	0.87	0.99	19581	0	0

BRA	Brazil	8757	Capanema	0.35	0.86	0.99	30382	0	0
BRA	Brazil	8758	Capitao Poco	0.34	0.80	0.92	24666	0	0
BRA	Brazil	8759	Castanhal	0.35	0.85	0.99	91806	0	0
BRA	Brazil	8760	Chaves	0.28	0.82	0.98	11966	0	0
BRA	Brazil	8761	Colares	0.38	0.87	0.99	5749	0	0
BRA	Brazil	8762	Conceicao Do Araguaia	0.38	0.88	0.99	20251	0	0
BRA	Brazil	8763	Concordia Do Para	0.35	0.83	0.96	15362	0	0
BRA	Brazil	8764	Cumaru Do Norte	0.31	0.84	0.98	6956	0	0
BRA	Brazil	8765	Curionopolis	0.36	0.87	0.99	8065	0	0
BRA	Brazil	8766	Curralinho	0.35	0.83	0.95	15063	0	0
BRA	Brazil	8767	Curua	0.34	0.82	0.95	6861	0	0
BRA	Brazil	8768	Curuca	0.33	0.81	0.94	18941	0	0
BRA	Brazil	8769	Dom Eliseu	0.34	0.82	0.95	27729	0	0
BRA	Brazil	8770	Eldorado Dos Carajas	0.36	0.87	0.99	14937	0	0
BRA	Brazil	8771	Faro	0.36	0.85	0.98	2909	0	0
BRA	Brazil	8772	Floresta Do Araguaia	0.39	0.86	0.97	8354	0	0
BRA	Brazil	8773	Garrafao Do Norte	0.39	0.89	1.00	10973	0	0
BRA	Brazil	8774	Goianesia Do Para	0.34	0.83	0.97	16756	0	0
BRA	Brazil	8775	Gurupa	0.35	0.83	0.95	14999	0	0
BRA	Brazil	8776	Igarape-acu	0.29	0.78	0.91	20043	921	0
BRA	Brazil	8777	Igarape-miri	0.34	0.82	0.94	28842	0	0
BRA	Brazil	8778	Inhangapi	0.33	0.81	0.94	5465	0	0
BRA	Brazil	8779	Ipixuna Do Para	0.30	0.79	0.93	35885	803	0
BRA	Brazil	8780	Irituia	0.34	0.81	0.93	15421	0	0
BRA	Brazil	8781	Itaituba	0.37	0.88	1.00	43809	0	0
BRA	Brazil	8782	Itupiranga	0.36	0.87	1.00	23520	0	0
BRA	Brazil	8783	Jacareacanga	0.41	0.92	1.00	4322	0	0
BRA	Brazil	8784	Jacunda	0.37	0.85	0.98	25557	0	0
BRA	Brazil	8785	Juruti	0.35	0.85	0.99	27007	0	0
BRA	Brazil	8786	Limoeiro Do Ajuru	0.35	0.85	0.99	12274	0	0
BRA	Brazil	8787	Mae Do Rio	0.35	0.83	0.95	13575	0	0
BRA	Brazil	8788	Magalhaes Barata	0.33	0.80	0.91	4187	25	0
BRA	Brazil	8789	Maraba	0.34	0.85	0.99	130275	0	0

BRA	Brazil	8790	Maracana	0.34	0.81	0.93	13210	0	0
BRA	Brazil	8791	Marapanim	0.34	0.82	0.95	12902	0	0
BRA	Brazil	8792	Marituba	0.35	0.85	0.99	54244	0	0
BRA	Brazil	8793	Medicilandia	0.35	0.84	0.98	14452	0	0
BRA	Brazil	8794	Melgaco	0.36	0.87	0.99	12117	0	0
BRA	Brazil	8795	Mocajuba	0.36	0.86	0.99	13935	0	0
BRA	Brazil	8796	Moju	0.33	0.82	0.96	42393	0	0
BRA	Brazil	8797	Monte Alegre	0.35	0.83	0.96	27599	0	0
BRA	Brazil	8798	Muana	0.34	0.81	0.93	18630	0	0
BRA	Brazil	8799	Nova Esperanca Do Piria	0.36	0.83	0.95	9537	0	0
BRA	Brazil	8800	Nova Ipixuna	0.35	0.84	0.98	7671	0	0
BRA	Brazil	8801	Nova Timboteua	0.32	0.81	0.95	7443	0	0
BRA	Brazil	8802	Novo Progresso	0.38	0.89	1.00	10838	0	0
BRA	Brazil	8803	Novo Repartimento	0.37	0.86	0.99	33530	0	0
BRA	Brazil	8804	Obidos	0.35	0.86	1.00	22686	0	0
BRA	Brazil	8805	Oeiras Do Para	0.35	0.81	0.92	14921	0	0
BRA	Brazil	8806	Oriximina	0.36	0.86	1.00	32518	0	0
BRA	Brazil	8807	Ourem	0.37	0.86	0.99	7588	0	0
BRA	Brazil	8808	Ourilandia Do Norte	0.38	0.90	0.99	12974	0	0
BRA	Brazil	8809	Pacaja	0.37	0.88	0.99	20760	0	0
BRA	Brazil	8810	Palestina Do Para	0.58	0.90	0.99	1619	0	0
BRA	Brazil	8811	Paragominas	0.35	0.87	1.00	51171	0	0
BRA	Brazil	8812	Parauapebas	0.35	0.89	1.00	103931	0	0
BRA	Brazil	8813	Pau D'arco	0.60	0.95	1.00	1111	0	0
BRA	Brazil	8814	Peixe-boi	0.32	0.80	0.94	4596	0	0
BRA	Brazil	8815	Picarra	0.53	0.93	1.00	3562	0	0
BRA	Brazil	8816	Placas	0.31	0.80	0.95	16101	0	0
BRA	Brazil	8817	Ponta De Pedras	0.33	0.81	0.94	15260	0	0
BRA	Brazil	8818	Portel	0.35	0.86	1.00	28155	0	0
BRA	Brazil	8819	Porto De Moz	0.34	0.84	0.98	19138	0	0
BRA	Brazil	8820	Prainha	0.34	0.85	1.00	14087	0	0
BRA	Brazil	8821	Primavera	0.37	0.86	1.00	4832	0	0
BRA	Brazil	8822	Quatipuru	0.35	0.86	0.99	5922	0	0

BRA	Brazil	8823	Redencao	0.21	0.66	0.75	50496	12334	3987
BRA	Brazil	8824	Rio Maria	0.47	0.88	0.99	6026	0	0
BRA	Brazil	8825	Rondon Do Para	0.39	0.86	0.98	21553	0	0
BRA	Brazil	8826	Ruropolis	0.30	0.80	0.94	26098	250	0
BRA	Brazil	8827	Salinopolis	0.35	0.84	0.98	16117	0	0
BRA	Brazil	8828	Salvaterra	0.35	0.84	0.99	11135	0	0
BRA	Brazil	8829	Santa Barbara Do Para	0.33	0.82	0.96	10182	0	0
BRA	Brazil	8830	Santa Cruz Do Arari	0.34	0.81	0.93	3926	0	0
BRA	Brazil	8831	Santa Isabel Do Para	0.33	0.83	0.97	34130	0	0
BRA	Brazil	8832	Santa Luzia Do Para	0.36	0.88	1.00	8520	0	0
BRA	Brazil	8833	Santa Maria Das Barreiras	0.33	0.86	0.98	10521	0	0
BRA	Brazil	8834	Santa Maria Do Para	0.34	0.82	0.95	11497	0	0
BRA	Brazil	8835	Santana Do Araguaia	0.29	0.78	0.92	39720	1598	0
BRA	Brazil	8836	Santarem	0.36	0.89	1.00	139513	0	0
BRA	Brazil	8837	Santarem Novo	0.35	0.83	0.96	2869	0	0
BRA	Brazil	8838	Santo Antonio Do Taua	0.35	0.84	0.97	14212	0	0
BRA	Brazil	8839	Sao Caetano De Odivelas	0.35	0.85	0.99	8087	0	0
BRA	Brazil	8840	Sao Domingos Do Araguaia	0.35	0.85	0.99	11613	0	0
BRA	Brazil	8841	Sao Domingos Do Capim	0.36	0.84	0.97	14036	0	0
BRA	Brazil	8842	Sao Felix Do Xingu	0.27	0.77	0.90	80731	5261	0
BRA	Brazil	8843	Sao Francisco Do Para	0.35	0.84	0.97	6986	0	0
BRA	Brazil	8844	Sao Geraldo Do Araguaia	0.35	0.84	0.97	11571	0	0
BRA	Brazil	8845	Sao Joao Da Ponta	0.34	0.81	0.94	2805	0	0
BRA	Brazil	8846	Sao Joao De Pirabas	0.33	0.81	0.93	10670	0	0
BRA	Brazil	8847	Sao Joao Do Araguaia	0.42	0.88	0.99	5177	0	0
BRA	Brazil	8848	Sao Miguel Do Guama	0.35	0.82	0.93	26508	0	0
BRA	Brazil	8849	Sao Sebastiao Da Boa Vista	0.35	0.82	0.94	11183	0	0
BRA	Brazil	8850	Sapucaia	1.00	1.00	1.00	0	0	0
BRA	Brazil	8851	Senador Jose Porfirio	0.49	0.93	1.00	3141	0	0
BRA	Brazil	8852	Soure	0.35	0.83	0.95	10835	0	0
BRA	Brazil	8853	Tailandia	0.34	0.85	0.99	53732	0	0
BRA	Brazil	8854	Terra Alta	0.35	0.84	0.97	5042	0	0
BRA	Brazil	8855	Terra Santa	0.33	0.81	0.95	9320	0	0

BRA	Brazil	8856	Tome-acu	0.35	0.86	0.99	28565	0	0
BRA	Brazil	8857	Tracuateua	0.35	0.83	0.97	12784	0	0
BRA	Brazil	8858	Trairao	0.33	0.84	0.98	8962	0	0
BRA	Brazil	8859	Tucuma	0.35	0.85	0.99	18052	0	0
BRA	Brazil	8860	Tucurui	0.38	0.88	0.99	48049	0	0
BRA	Brazil	8861	Ulianopolis	0.29	0.78	0.92	34544	1054	0
BRA	Brazil	8862	Uruara	0.36	0.86	0.99	20366	0	0
BRA	Brazil	8863	Vigia	0.34	0.82	0.95	24304	0	0
BRA	Brazil	8864	Viseu	0.35	0.85	0.99	27858	0	0
BRA	Brazil	8865	Vitoria Do Xingu	0.36	0.87	0.99	6738	0	0
BRA	Brazil	8866	Xinguara	0.36	0.87	0.99	19833	0	0
BRA	Brazil	8867	Agua Branca	0.08	0.40	0.55	8797	4854	3016
BRA	Brazil	8868	Aguiar	0.08	0.40	0.55	4485	2474	1538
BRA	Brazil	8869	Alagoa Grande	0.08	0.40	0.55	21371	11791	7327
BRA	Brazil	8870	Alagoa Nova	0.08	0.40	0.55	13737	7579	4710
BRA	Brazil	8871	Alagoinha	0.08	0.40	0.55	10568	5830	3623
BRA	Brazil	8872	Alcantil	0.08	0.40	0.55	3302	1822	1132
BRA	Brazil	8873	Algodao De Jandaira	0.08	0.40	0.55	5887	3248	2018
BRA	Brazil	8874	Alhandra	0.08	0.40	0.55	15730	8679	5393
BRA	Brazil	8875	Amparo	0.08	0.40	0.55	1620	894	555
BRA	Brazil	8876	Aparecida	0.13	0.52	0.75	4727	1950	375
BRA	Brazil	8877	Aracagi	0.08	0.40	0.55	12367	6823	4240
BRA	Brazil	8878	Arara	0.08	0.40	0.55	7445	4107	2553
BRA	Brazil	8879	Araruna	0.08	0.40	0.55	16497	9102	5656
BRA	Brazil	8880	Areia	0.08	0.40	0.55	16941	9347	5809
BRA	Brazil	8881	Areia De Baraunas	0.08	0.40	0.55	1468	810	503
BRA	Brazil	8882	Areial	0.08	0.40	0.55	6535	3606	2241
BRA	Brazil	8883	Aroeiras	0.08	0.40	0.55	14042	7747	4815
BRA	Brazil	8884	Assis Chateaubriand	0.70	0.95	1.00	1266	0	0
BRA	Brazil	8885	Assuncao	0.08	0.40	0.55	3355	1851	1150
BRA	Brazil	8886	Baia Da Traicao	0.08	0.40	0.55	6535	3605	2241
BRA	Brazil	8887	Bananeiras	0.08	0.40	0.55	20643	11389	7078
BRA	Brazil	8888	Barauna	0.08	0.40	0.55	3674	2027	1260

BRA	Brazil	8889	Barra De Santa Rosa	0.08	0.40	0.55	11353	6264	3893
BRA	Brazil	8890	Barra De Santana	0.08	0.40	0.55	5808	3204	1991
BRA	Brazil	8891	Barra De Sao Miguel	0.08	0.40	0.55	4392	2423	1506
BRA	Brazil	8892	Bayeux	0.08	0.40	0.55	70647	38978	24223
BRA	Brazil	8893	Belem	0.08	0.40	0.55	10811	5965	3707
BRA	Brazil	8894	Belem Do Brejo Do Cruz	0.08	0.40	0.55	5427	2994	1861
BRA	Brazil	8895	Bernardino Batista	0.08	0.40	0.55	2137	1179	733
BRA	Brazil	8896	Boa Ventura	0.08	0.40	0.55	3969	2190	1361
BRA	Brazil	8897	Boa Vista	1.00	1.00	1.00	0	0	0
BRA	Brazil	8898	Bom Jesus	1.00	1.00	1.00	0	0	0
BRA	Brazil	8899	Bom Sucesso	0.08	0.40	0.55	3902	2153	1338
BRA	Brazil	8900	Bonito De Santa Fe	0.08	0.40	0.55	8532	4707	2925
BRA	Brazil	8901	Boqueirao	0.08	0.40	0.55	13053	7201	4475
BRA	Brazil	8902	Borborema	0.32	0.84	0.97	4375	0	0
BRA	Brazil	8903	Brejo Do Cruz	0.08	0.40	0.55	10433	5756	3577
BRA	Brazil	8904	Brejo Dos Santos	0.08	0.40	0.55	4940	2726	1694
BRA	Brazil	8905	Caapora	0.08	0.40	0.55	16443	9072	5638
BRA	Brazil	8906	Cabaceiras	0.08	0.40	0.55	3854	2126	1321
BRA	Brazil	8907	Cabedelo	0.08	0.40	0.55	48954	27009	16785
BRA	Brazil	8908	Cachoeira Dos Indios	0.08	0.40	0.55	7440	4105	2551
BRA	Brazil	8909	Cacimba De Areia	0.08	0.40	0.55	2825	1559	969
BRA	Brazil	8910	Cacimba De Dentro	0.08	0.40	0.55	13510	7454	4632
BRA	Brazil	8911	Cacimbas	0.08	0.40	0.55	4951	2732	1698
BRA	Brazil	8912	Caicara	0.20	0.58	0.82	4493	1614	0
BRA	Brazil	8913	Cajazeiras	0.08	0.40	0.55	45629	25175	15645
BRA	Brazil	8914	Cajazeirinhas	0.08	0.40	0.55	2341	1292	803
BRA	Brazil	8915	Caldas Brandao	0.08	0.40	0.55	2733	1508	937
BRA	Brazil	8916	Camalau	0.08	0.40	0.55	4524	2496	1551
BRA	Brazil	8917	Campina Grande	0.08	0.40	0.55	293485	161923	100626
BRA	Brazil	8918	Capim	0.08	0.40	0.55	5173	2854	1774
BRA	Brazil	8919	Caraubas	0.08	0.40	0.55	2922	1612	1002
BRA	Brazil	8920	Carrapateira	0.08	0.40	0.55	1858	1025	637
BRA	Brazil	8921	Casserengue	0.08	0.40	0.55	5492	3030	1883

BRA	Brazil	8922	Catingueira	0.08	0.40	0.55	3245	1790	1113
BRA	Brazil	8923	Catole Do Rocha	0.08	0.40	0.55	21827	12043	7484
BRA	Brazil	8924	Caturite	0.08	0.40	0.55	3513	1938	1204
BRA	Brazil	8925	Conceicao	0.08	0.40	0.55	13924	7682	4774
BRA	Brazil	8926	Condado	0.08	0.40	0.55	5721	3157	1962
BRA	Brazil	8927	Conde	0.08	0.40	0.55	16557	9135	5677
BRA	Brazil	8928	Congo	0.08	0.40	0.55	3636	2006	1247
BRA	Brazil	8929	Coremas	0.08	0.40	0.55	11764	6491	4034
BRA	Brazil	8930	Coxixola	0.08	0.40	0.55	1147	633	393
BRA	Brazil	8931	Cruz Do Espirito Santo	0.08	0.40	0.55	12808	7067	4392
BRA	Brazil	8932	Cubati	0.08	0.40	0.55	5785	3192	1984
BRA	Brazil	8933	Cuite	0.08	0.40	0.55	14309	7895	4906
BRA	Brazil	8934	Cuite De Mamanguape	0.08	0.40	0.55	4619	2548	1584
BRA	Brazil	8935	Cuitegi	0.08	0.40	0.55	5361	2958	1838
BRA	Brazil	8936	Curral De Cima	0.08	0.40	0.55	3678	2029	1261
BRA	Brazil	8937	Curral Velho	0.08	0.40	0.55	1738	959	596
BRA	Brazil	8938	Damiao	0.08	0.40	0.55	3620	1997	1241
BRA	Brazil	8939	Desterro	0.08	0.40	0.55	5581	3079	1914
BRA	Brazil	8940	Diamante	0.08	0.40	0.55	4428	2443	1518
BRA	Brazil	8941	Dona Ines	0.08	0.40	0.55	5596	3087	1919
BRA	Brazil	8942	Duas Estradas	0.08	0.40	0.55	1730	954	593
BRA	Brazil	8943	Emas	0.08	0.40	0.55	2588	1428	887
BRA	Brazil	8944	Esperanca	0.08	0.40	0.55	23455	12941	8042
BRA	Brazil	8945	Fagundes	0.08	0.40	0.55	9537	5262	3270
BRA	Brazil	8946	Frei Martinho	0.08	0.40	0.55	2175	1200	746
BRA	Brazil	8947	Gado Bravo	0.08	0.40	0.55	6332	3494	2171
BRA	Brazil	8948	Guarabira	0.08	0.40	0.55	42517	23458	14578
BRA	Brazil	8949	Gurinhem	0.08	0.40	0.55	9689	5346	3322
BRA	Brazil	8950	Gurjao	0.08	0.40	0.55	2863	1580	982
BRA	Brazil	8951	Ibiara	0.08	0.40	0.55	4350	2400	1492
BRA	Brazil	8952	Igaracy	0.08	0.40	0.55	4388	2421	1504
BRA	Brazil	8953	Imaculada	0.08	0.40	0.55	6657	3673	2282
BRA	Brazil	8954	Inga	0.08	0.40	0.55	10451	5766	3583

BRA	Brazil	8955	Itabaiana	0.08	0.40	0.55	17819	9831	6110
BRA	Brazil	8956	Itaporanga	0.59	0.76	0.92	5334	889	0
BRA	Brazil	8957	Itapororoca	0.08	0.40	0.55	13773	7599	4722
BRA	Brazil	8958	Itatuba	0.08	0.40	0.55	8688	4793	2979
BRA	Brazil	8959	Jacarau	0.08	0.40	0.55	10250	5655	3514
BRA	Brazil	8960	Jerico	0.08	0.40	0.55	5317	2933	1823
BRA	Brazil	8961	Joao Pessoa	0.08	0.40	0.55	587626	324208	201478
BRA	Brazil	8962	Juarez Tavora	0.08	0.40	0.55	4105	2265	1407
BRA	Brazil	8963	Juazeirinho	0.08	0.40	0.55	12950	7145	4440
BRA	Brazil	8964	Junco Do Serido	0.08	0.40	0.55	4794	2645	1644
BRA	Brazil	8965	Juripiranga	0.08	0.40	0.55	7454	4113	2556
BRA	Brazil	8966	Juru	0.08	0.40	0.55	8226	4539	2821
BRA	Brazil	8967	Lagoa	0.08	0.40	0.55	3188	1759	1093
BRA	Brazil	8968	Lagoa De Dentro	0.08	0.40	0.55	5741	3167	1968
BRA	Brazil	8969	Lagoa Seca	0.08	0.40	0.55	14511	8006	4975
BRA	Brazil	8970	Lastro	0.08	0.40	0.55	2679	1478	918
BRA	Brazil	8971	Livramento	0.08	0.40	0.55	5158	2846	1768
BRA	Brazil	8972	Logradouro	0.08	0.40	0.55	5862	3234	2010
BRA	Brazil	8973	Lucena	0.08	0.40	0.55	9239	5097	3168
BRA	Brazil	8974	Mae D'agua	0.08	0.40	0.55	3533	1949	1211
BRA	Brazil	8975	Malta	0.08	0.40	0.55	4228	2333	1450
BRA	Brazil	8976	Mamanguape	0.08	0.40	0.55	32827	18111	11255
BRA	Brazil	8977	Manaira	0.08	0.40	0.55	8660	4778	2969
BRA	Brazil	8978	Marcacao	0.08	0.40	0.55	3981	2197	1365
BRA	Brazil	8979	Mari	0.08	0.40	0.55	16833	9287	5771
BRA	Brazil	8980	Marizopolis	0.08	0.40	0.55	8257	4556	2831
BRA	Brazil	8981	Massaranduba	0.08	0.45	0.65	11035	5383	2331
BRA	Brazil	8982	Mataraca	0.08	0.40	0.55	5952	3284	2041
BRA	Brazil	8983	Matinhas	0.08	0.40	0.55	2271	1253	779
BRA	Brazil	8984	Mato Grosso	0.08	0.40	0.55	994	548	341
BRA	Brazil	8985	Matureia	0.08	0.40	0.55	4926	2718	1689
BRA	Brazil	8986	Mogeiro	0.08	0.40	0.55	9166	5057	3143
BRA	Brazil	8987	Montadas	0.08	0.40	0.55	6627	3656	2272

BRA	Brazil	8988	Monte Horebe	0.08	0.40	0.55	3419	1886	1172
BRA	Brazil	8989	Monteiro	0.08	0.40	0.55	24285	13398	8326
BRA	Brazil	8990	Mulungu	0.08	0.40	0.55	7631	4210	2616
BRA	Brazil	8991	Natuba	0.08	0.40	0.55	8045	4438	2758
BRA	Brazil	8992	Nazarezinho	0.08	0.40	0.55	5847	3226	2005
BRA	Brazil	8993	Nova Floresta	0.08	0.40	0.55	10357	5714	3551
BRA	Brazil	8994	Nova Olinda	0.08	0.40	0.55	4763	2628	1633
BRA	Brazil	8995	Nova Palmeira	0.08	0.40	0.55	2961	1634	1015
BRA	Brazil	8996	Olho D'agua	0.08	0.40	0.55	4899	2703	1680
BRA	Brazil	8997	Olivedos	0.08	0.40	0.55	2765	1526	948
BRA	Brazil	8998	Ouro Velho	0.08	0.40	0.55	2417	1334	829
BRA	Brazil	8999	Parari	0.08	0.40	0.55	1022	564	351
BRA	Brazil	9000	Passagem	0.08	0.40	0.55	1926	1062	660
BRA	Brazil	9001	Patos	0.08	0.40	0.55	78788	43469	27014
BRA	Brazil	9002	Paulista	0.08	0.40	0.55	9841	5430	3374
BRA	Brazil	9003	Pedra Branca	0.08	0.40	0.55	3357	1852	1151
BRA	Brazil	9004	Pedra Lavrada	0.08	0.40	0.55	5883	3246	2017
BRA	Brazil	9005	Pedras De Fogo	0.08	0.40	0.55	23364	12890	8011
BRA	Brazil	9006	Pedro Regio	0.08	0.40	0.55	4899	2703	1680
BRA	Brazil	9007	Pianco	0.08	0.40	0.55	11712	6462	4016
BRA	Brazil	9008	Picui	0.08	0.40	0.55	14464	7980	4959
BRA	Brazil	9009	Pilar	0.08	0.40	0.55	8401	4635	2880
BRA	Brazil	9010	Piloes	0.08	0.40	0.55	4149	2289	1423
BRA	Brazil	9011	Piloezinhos	0.08	0.40	0.55	3921	2164	1345
BRA	Brazil	9012	Pirpirituba	0.08	0.40	0.55	9317	5140	3194
BRA	Brazil	9013	Pitimbu	0.08	0.40	0.55	12611	6958	4324
BRA	Brazil	9014	Pocinhos	0.08	0.40	0.55	14835	8185	5086
BRA	Brazil	9015	Poco Dantas	0.08	0.40	0.55	2088	1152	716
BRA	Brazil	9016	Poco De Jose De Moura	0.08	0.40	0.55	3370	1860	1156
BRA	Brazil	9017	Pombal	0.08	0.40	0.55	22966	12671	7874
BRA	Brazil	9018	Prata	0.08	0.40	0.55	2972	1640	1019
BRA	Brazil	9019	Princesa Isabel	0.08	0.40	0.55	17221	9501	5904
BRA	Brazil	9020	Puxinana	0.08	0.40	0.55	16556	9134	5676

BRA	Brazil	9021	Queimadas	0.08	0.40	0.55	32471	17915	11133
BRA	Brazil	9022	Quixaba	0.08	0.40	0.55	1139	628	390
BRA	Brazil	9023	Remigio	0.08	0.40	0.55	13137	7248	4504
BRA	Brazil	9024	Riachao	0.08	0.40	0.55	2452	1353	841
BRA	Brazil	9025	Riachao Do Poco	0.08	0.40	0.55	6663	3676	2284
BRA	Brazil	9026	Riacho De Santo Antonio	0.08	0.40	0.55	1480	816	507
BRA	Brazil	9027	Riacho Dos Cavalos	0.08	0.40	0.55	6583	3632	2257
BRA	Brazil	9028	Rio Tinto	0.08	0.40	0.55	20082	11080	6886
BRA	Brazil	9029	Salgadinho	0.08	0.40	0.55	2730	1506	936
BRA	Brazil	9030	Salgado De Sao Felix	0.08	0.40	0.55	7177	3960	2461
BRA	Brazil	9031	Santa Cecilia	0.19	0.60	0.83	4410	1459	0
BRA	Brazil	9032	Santa Cruz	0.08	0.40	0.55	5521	3046	1893
BRA	Brazil	9033	Santa Helena	0.08	0.40	0.55	3849	2123	1320
BRA	Brazil	9034	Santa Ines	0.08	0.40	0.55	2647	1461	908
BRA	Brazil	9035	Santa Luzia	0.08	0.40	0.55	11268	6217	3863
BRA	Brazil	9036	Santa Rita	0.08	0.40	0.55	100020	55183	34293
BRA	Brazil	9037	Santa Teresinha	0.08	0.40	0.55	3133	1729	1074
BRA	Brazil	9038	Santana De Mangueira	0.08	0.40	0.55	4138	2283	1419
BRA	Brazil	9039	Santana Dos Garrotes	0.08	0.40	0.55	4007	2211	1374
BRA	Brazil	9040	Santarem	0.08	0.40	0.55	1835	1013	629
BRA	Brazil	9041	Santo Andre	1.00	1.00	1.00	0	0	0
BRA	Brazil	9042	Sao Bento	0.08	0.40	0.55	24070	13280	8253
BRA	Brazil	9043	Sao Bento De Pombal	0.08	0.40	0.55	2839	1566	973
BRA	Brazil	9044	Sao Domingos De Pombal	0.08	0.40	0.55	2635	1454	903
BRA	Brazil	9045	Sao Domingos Do Cariri	0.08	0.40	0.55	1926	1062	660
BRA	Brazil	9046	Sao Francisco	0.08	0.40	0.55	2238	1235	767
BRA	Brazil	9047	Sao Joao Do Cariri	0.08	0.40	0.55	3250	1793	1114
BRA	Brazil	9048	Sao Joao Do Rio Do Peixe	0.08	0.40	0.55	13521	7460	4636
BRA	Brazil	9049	Sao Joao Do Tigre	0.08	0.40	0.55	3066	1691	1051
BRA	Brazil	9050	Sao Jose Da Lagoa Tapada	0.08	0.40	0.55	5698	3144	1954
BRA	Brazil	9051	Sao Jose De Caiana	0.08	0.40	0.55	4697	2592	1611
BRA	Brazil	9052	Sao Jose De Espinharas	0.08	0.40	0.55	3532	1949	1211
BRA	Brazil	9053	Sao Jose De Piranhas	0.08	0.40	0.55	15035	8295	5155

BRA	Brazil	9054	Sao Jose De Princesa	0.08	0.40	0.55	2945	1625	1010
BRA	Brazil	9055	Sao Jose Do Bonfim	0.08	0.40	0.55	2420	1335	830
BRA	Brazil	9056	Sao Jose Do Brejo Do Cruz	0.08	0.40	0.55	1280	706	439
BRA	Brazil	9057	Sao Jose Do Sabugi	0.08	0.40	0.55	3026	1670	1038
BRA	Brazil	9058	Sao Jose Dos Cordeiros	0.08	0.40	0.55	3537	1951	1213
BRA	Brazil	9059	Sao Jose Dos Ramos	0.08	0.40	0.55	3911	2158	1341
BRA	Brazil	9060	Sao Mamede	0.08	0.40	0.55	5990	3305	2054
BRA	Brazil	9061	Sao Miguel De Taipu	0.08	0.40	0.55	4044	2231	1387
BRA	Brazil	9062	Sao Sebastiao De Lagoa De Roca	0.08	0.40	0.55	9310	5136	3192
BRA	Brazil	9063	Sao Sebastiao Do Umbuzeiro	0.08	0.40	0.55	2331	1286	799
BRA	Brazil	9064	Sape	0.08	0.40	0.55	37774	20841	12951
BRA	Brazil	9065	Serido	0.08	0.40	0.55	7514	4145	2576
BRA	Brazil	9066	Serra Branca	0.08	0.40	0.55	9619	5307	3298
BRA	Brazil	9067	Serra Da Raiz	0.08	0.40	0.55	2509	1384	860
BRA	Brazil	9068	Serra Grande	0.08	0.40	0.55	2219	1224	761
BRA	Brazil	9069	Serra Redonda	0.08	0.40	0.55	4753	2623	1630
BRA	Brazil	9070	Serraria	0.08	0.40	0.55	3423	1888	1173
BRA	Brazil	9071	Sertaozinho	0.08	0.40	0.55	2112	1165	724
BRA	Brazil	9072	Sobrado	0.08	0.40	0.55	3974	2193	1363
BRA	Brazil	9073	Solanea	0.08	0.40	0.55	14856	8196	5094
BRA	Brazil	9074	Soledade	0.98	0.99	1.00	0	0	0
BRA	Brazil	9075	Sossego	0.08	0.40	0.55	2278	1257	781
BRA	Brazil	9076	Sousa	0.08	0.40	0.55	47965	26463	16446
BRA	Brazil	9077	Sume	0.08	0.40	0.55	12617	6961	4326
BRA	Brazil	9078	Tacima	0.08	0.40	0.55	9505	5244	3259
BRA	Brazil	9079	Taperoa	0.08	0.40	0.55	11414	6298	3914
BRA	Brazil	9080	Tavares	0.08	0.42	0.59	9573	5035	2767
BRA	Brazil	9081	Teixeira	0.08	0.40	0.55	11489	6339	3939
BRA	Brazil	9082	Tenorio	0.08	0.40	0.55	1849	1020	634
BRA	Brazil	9083	Triunfo	0.87	0.95	1.00	0	0	0
BRA	Brazil	9084	Uirauna	0.08	0.40	0.55	11905	6568	4082
BRA	Brazil	9085	Umbuzeiro	0.08	0.40	0.55	7292	4023	2500
BRA	Brazil	9086	Varzea	0.08	0.40	0.55	1942	1071	666

BRA	Brazil	9087	Vieiropolis	0.08	0.40	0.55	3104	1713	1064
BRA	Brazil	9088	Vista Serrana	0.08	0.40	0.55	2542	1403	872
BRA	Brazil	9089	Zabele	0.08	0.40	0.55	1843	1017	632
BRA	Brazil	9090	Abatia	0.08	0.40	0.55	5742	3168	1969
BRA	Brazil	9091	Adrianopolis	0.08	0.40	0.55	4541	2506	1557
BRA	Brazil	9092	Agudos Do Sul	0.08	0.40	0.55	6560	3619	2249
BRA	Brazil	9093	Almirante Tamandare	0.24	0.64	0.88	64298	18611	0
BRA	Brazil	9094	Altamira Do Parana	0.76	0.91	1.00	154	0	0
BRA	Brazil	9095	Alto Parana	0.39	0.80	0.97	5963	11	0
BRA	Brazil	9096	Alto Piquiri	0.37	0.73	0.94	4363	751	0
BRA	Brazil	9097	Altonia	0.35	0.77	0.97	9926	576	0
BRA	Brazil	9098	Alvorada Do Sul	0.28	0.72	0.95	5809	916	0
BRA	Brazil	9099	Amapora	0.32	0.71	0.97	2914	548	0
BRA	Brazil	9100	Ampere	0.32	0.77	0.98	9132	632	0
BRA	Brazil	9101	Anahy	0.51	0.90	0.99	653	0	0
BRA	Brazil	9102	Andira	0.27	0.77	0.96	11000	679	0
BRA	Brazil	9103	Angulo	0.08	0.40	0.55	2129	1175	730
BRA	Brazil	9104	Antonina	0.21	0.59	0.82	11027	3826	0
BRA	Brazil	9105	Antonio Olinto	0.29	0.62	0.85	3806	1316	0
BRA	Brazil	9106	Apucarana	0.23	0.65	0.91	75774	19705	0
BRA	Brazil	9107	Arapongas	0.27	0.67	0.91	62912	15452	0
BRA	Brazil	9108	Arapoti	0.33	0.75	0.97	12996	1404	0
BRA	Brazil	9109	Arapua	0.17	0.49	0.65	2127	1039	507
BRA	Brazil	9110	Araruna	0.17	0.49	0.65	8848	4323	2111
BRA	Brazil	9111	Araucaria	0.23	0.66	0.93	80498	20468	0
BRA	Brazil	9112	Ariranha Do Ivai	0.53	0.83	0.98	613	0	0
BRA	Brazil	9113	Assai	0.39	0.72	0.91	6635	1346	0
BRA	Brazil	9114	Assis Chateaubriand	0.17	0.49	0.65	21486	10499	5126
BRA	Brazil	9115	Astorga	0.22	0.68	0.91	15078	3243	0
BRA	Brazil	9116	Atalaia	0.17	0.49	0.65	2502	1223	597
BRA	Brazil	9117	Balsa Nova	0.22	0.67	0.95	6691	1558	0
BRA	Brazil	9118	Bandeirantes	0.08	0.40	0.55	23393	12906	8021
BRA	Brazil	9119	Barbosa Ferraz	0.42	0.75	0.97	4402	547	0

BRA	Brazil	9120	Barra Do Jacare	0.34	0.74	0.92	1274	167	0
BRA	Brazil	9121	Barracao	1.00	1.00	1.00	0	0	0
BRA	Brazil	9122	Bela Vista Do Caroba	0.54	0.87	0.99	959	0	0
BRA	Brazil	9123	Bela Vista Do Paraiso	0.30	0.70	0.90	7755	1630	0
BRA	Brazil	9124	Bituruna	0.71	0.88	0.99	1504	0	0
BRA	Brazil	9125	Boa Esperanca	0.17	0.49	0.65	2825	1380	674
BRA	Brazil	9126	Boa Esperanca Do Iguacu	0.53	0.83	0.98	747	0	0
BRA	Brazil	9127	Boa Ventura De Sao Roque	0.47	0.81	0.98	2221	0	0
BRA	Brazil	9128	Boa Vista Da Aparecida	0.17	0.49	0.65	4996	2441	1192
BRA	Brazil	9129	Bocaiuva Do Sul	0.24	0.63	0.88	6879	2026	0
BRA	Brazil	9130	Bom Jesus Do Sul	0.46	0.87	1.00	1214	0	0
BRA	Brazil	9131	Bom Sucesso	0.08	0.40	0.55	5056	2789	1733
BRA	Brazil	9132	Bom Sucesso Do Sul	0.41	0.74	0.97	1203	174	0
BRA	Brazil	9133	Borrazopolis	0.31	0.72	0.96	3711	624	0
BRA	Brazil	9134	Braganey	0.38	0.80	0.98	2445	16	0
BRA	Brazil	9135	Brasilandia Do Sul	0.53	0.84	0.98	784	0	0
BRA	Brazil	9136	Cafeara	0.28	0.71	0.93	1507	251	0
BRA	Brazil	9137	Cafelandia	0.47	0.88	1.00	5607	0	0
BRA	Brazil	9138	Cafezal Do Sul	0.37	0.80	0.98	1841	12	0
BRA	Brazil	9139	California	0.25	0.65	0.89	4634	1270	0
BRA	Brazil	9140	Cambara	0.44	0.74	0.93	9162	1544	0
BRA	Brazil	9141	Cambe	0.25	0.72	0.95	57131	7939	0
BRA	Brazil	9142	Cambira	0.28	0.64	0.87	4024	1206	0
BRA	Brazil	9143	Campina Da Lagoa	0.36	0.76	0.98	6608	607	0
BRA	Brazil	9144	Campina Do Simao	0.17	0.49	0.65	2537	1240	605
BRA	Brazil	9145	Campina Grande Do Sul	0.26	0.66	0.90	22757	5936	0
BRA	Brazil	9146	Campo Bonito	0.17	0.49	0.65	2667	1303	636
BRA	Brazil	9147	Campo Do Tenente	0.08	0.40	0.55	5661	3123	1941
BRA	Brazil	9148	Campo Largo	0.08	0.40	0.55	92458	51011	31701
BRA	Brazil	9149	Campo Magro	0.22	0.61	0.83	14885	4947	0
BRA	Brazil	9150	Campo Mourao	0.39	0.73	0.97	38084	6100	0
BRA	Brazil	9151	Candido De Abreu	0.44	0.83	1.00	5741	0	0
BRA	Brazil	9152	Candoi	0.69	0.88	0.99	1715	0	0

BRA	Brazil	9153	Cantagalo	0.17	0.49	0.65	8471	4139	2021
BRA	Brazil	9154	Capanema	0.17	0.49	0.65	11931	5830	2846
BRA	Brazil	9155	Capitao Leonidas Marques	0.37	0.81	0.98	6399	0	0
BRA	Brazil	9156	Carambei	0.24	0.64	0.89	12696	3520	0
BRA	Brazil	9157	Carlopolis	0.76	0.89	0.99	514	0	0
BRA	Brazil	9158	Cascavel	0.17	0.49	0.65	200852	98144	47919
BRA	Brazil	9159	Castro	0.25	0.68	0.94	39086	8178	0
BRA	Brazil	9160	Catanduvas	0.38	0.85	1.00	4296	0	0
BRA	Brazil	9161	Centenario Do Sul	0.17	0.49	0.65	7114	3476	1697
BRA	Brazil	9162	Cerro Azul	0.23	0.60	0.81	10126	3501	0
BRA	Brazil	9163	Ceu Azul	0.30	0.73	0.94	6610	894	0
BRA	Brazil	9164	Chopinzinho	0.72	0.87	0.99	1614	0	0
BRA	Brazil	9165	Cianorte	0.30	0.74	0.97	39430	4861	0
BRA	Brazil	9166	Cidade Gaucha	0.36	0.78	0.98	5463	278	0
BRA	Brazil	9167	Clevelandia	0.17	0.49	0.65	10878	5315	2595
BRA	Brazil	9168	Colombo	0.24	0.62	0.83	129465	41798	0
BRA	Brazil	9169	Colorado	0.35	0.82	0.98	10736	0	0
BRA	Brazil	9170	Congonhinhas	0.27	0.69	0.95	4667	968	0
BRA	Brazil	9171	Conselheiro Mairinck	0.08	0.40	0.55	2639	1456	905
BRA	Brazil	9172	Contenda	0.19	0.58	0.80	11324	4073	76
BRA	Brazil	9173	Corbelia	0.38	0.78	0.97	7339	419	0
BRA	Brazil	9174	Cornelio Procopio	0.24	0.69	0.91	26922	5332	0
BRA	Brazil	9175	Coronel Domingos Soares	0.38	0.76	0.98	3166	294	0
BRA	Brazil	9176	Coronel Vivida	0.64	0.84	0.99	3530	0	0
BRA	Brazil	9177	Corumbatai Do Sul	0.30	0.69	0.94	2304	522	0
BRA	Brazil	9178	Cruz Machado	0.50	0.80	0.98	5708	43	0
BRA	Brazil	9179	Cruzeiro Do Iguacu	0.39	0.81	0.99	1780	0	0
BRA	Brazil	9180	Cruzeiro Do Oeste	0.31	0.71	0.96	10311	1942	0
BRA	Brazil	9181	Cruzeiro Do Sul	0.17	0.49	0.65	2827	1381	674
BRA	Brazil	9182	Cruzmaltina	0.17	0.49	0.65	1957	956	467
BRA	Brazil	9183	Curitiba	0.08	0.47	0.69	1350833	624227	210605
BRA	Brazil	9184	Curiuva	0.24	0.64	0.89	8431	2397	0
BRA	Brazil	9185	Diamante D'oeste	0.41	0.80	0.99	2041	0	0

BRA	Brazil	9186	Diamante Do Norte	0.40	0.83	0.97	2164	0	0
BRA	Brazil	9187	Diamante Do Sul	0.42	0.83	0.99	1302	0	0
BRA	Brazil	9188	Dois Vizinhos	0.45	0.79	0.98	13697	492	0
BRA	Brazil	9189	Douradina	0.17	0.49	0.65	5335	2607	1273
BRA	Brazil	9190	Doutor Camargo	0.22	0.64	0.88	3540	993	0
BRA	Brazil	9191	Doutor Ulysses	0.24	0.59	0.76	3243	1241	229
BRA	Brazil	9192	Eneas Marques	0.35	0.80	0.96	2744	0	0
BRA	Brazil	9193	Engenheiro Beltrao	0.39	0.81	0.97	5901	0	0
BRA	Brazil	9194	Entre Rios Do Oeste	0.24	0.62	0.85	2530	813	0
BRA	Brazil	9195	Esperanca Nova	0.38	0.78	0.96	777	28	0
BRA	Brazil	9196	Espigao Alto Do Iguacu	0.41	0.81	0.99	1832	0	0
BRA	Brazil	9197	Farol	0.41	0.77	0.97	1300	116	0
BRA	Brazil	9198	Faxinal	0.08	0.40	0.55	12744	7031	4370
BRA	Brazil	9199	Fazenda Rio Grande	0.25	0.64	0.87	52846	15846	0
BRA	Brazil	9200	Fenix	0.31	0.73	0.96	2382	344	0
BRA	Brazil	9201	Fernandes Pinheiro	0.40	0.73	0.91	2424	407	0
BRA	Brazil	9202	Figueira	0.25	0.65	0.90	4407	1204	0
BRA	Brazil	9203	Flor Da Serra Do Sul	0.52	0.87	1.00	1194	0	0
BRA	Brazil	9204	Florai	0.39	0.78	0.95	2099	87	0
BRA	Brazil	9205	Floresta	0.23	0.69	0.92	3786	732	0
BRA	Brazil	9206	Florestopolis	0.27	0.70	0.92	5920	1064	0
BRA	Brazil	9207	Florida	0.39	0.78	0.91	1110	59	0
BRA	Brazil	9208	Formosa Do Oeste	0.36	0.79	0.96	3187	105	0
BRA	Brazil	9209	Foz Do Iguacu	0.50	0.85	1.00	76447	0	0
BRA	Brazil	9210	Foz Do Jordao	0.51	0.84	1.00	1537	0	0
BRA	Brazil	9211	Francisco Alves	0.34	0.75	0.97	2911	335	0
BRA	Brazil	9212	Francisco Beltrao	0.33	0.73	0.96	41596	6215	0
BRA	Brazil	9213	General Carneiro	0.08	0.40	0.55	9971	5501	3419
BRA	Brazil	9214	Godoy Moreira	0.81	0.92	0.99	0	0	0
BRA	Brazil	9215	Goioere	0.38	0.76	0.96	12582	1295	0
BRA	Brazil	9216	Goioxim	0.45	0.81	1.00	2619	0	0
BRA	Brazil	9217	Grandes Rios	0.36	0.76	0.99	2696	274	0
BRA	Brazil	9218	Guaira	0.58	0.93	1.00	7172	0	0

BRA	Brazil	9219	Guairaca	0.42	0.83	0.98	2469	0	0
BRA	Brazil	9220	Guamiranga	0.42	0.84	1.00	2860	0	0
BRA	Brazil	9221	Guapirama	0.64	0.88	1.00	613	0	0
BRA	Brazil	9222	Guaporema	0.30	0.73	0.96	1152	160	0
BRA	Brazil	9223	Guaraci	0.67	0.97	1.00	708	0	0
BRA	Brazil	9224	Guaraniacu	0.46	0.82	0.97	4784	0	0
BRA	Brazil	9225	Guarapuava	0.17	0.49	0.65	113346	55385	27041
BRA	Brazil	9226	Guaraquecaba	0.08	0.40	0.55	5476	3021	1878
BRA	Brazil	9227	Guaratuba	0.22	0.65	0.92	20818	5243	0
BRA	Brazil	9228	Honorio Serpa	0.38	0.73	0.98	2510	414	0
BRA	Brazil	9229	Ibaiti	0.24	0.66	0.92	17283	4415	0
BRA	Brazil	9230	Ibema	0.40	0.77	0.98	2601	218	0
BRA	Brazil	9231	Ibipora	0.23	0.68	0.94	30978	6214	0
BRA	Brazil	9232	Icaraima	0.36	0.82	0.99	3730	0	0
BRA	Brazil	9233	Iguaracu	0.32	0.70	0.92	2094	434	0
BRA	Brazil	9234	Iguatu	0.17	0.49	0.65	1795	877	428
BRA	Brazil	9235	Imbau	0.24	0.62	0.85	7082	2242	0
BRA	Brazil	9236	Imbituva	0.41	0.76	0.95	12762	1384	0
BRA	Brazil	9237	Inacio Martins	0.47	0.79	0.99	3710	101	0
BRA	Brazil	9238	Inaja	0.35	0.77	0.98	1411	92	0
BRA	Brazil	9239	Indianopolis	0.17	0.49	0.65	2806	1371	669
BRA	Brazil	9240	Ipiranga	0.28	0.73	0.95	7830	1100	0
BRA	Brazil	9241	Ipora	0.17	0.49	0.65	9264	4527	2210
BRA	Brazil	9242	Iracema Do Oeste	0.48	0.84	0.97	806	0	0
BRA	Brazil	9243	Irati	0.61	0.83	0.98	11203	0	0
BRA	Brazil	9244	Iretama	0.29	0.67	0.89	5378	1409	0
BRA	Brazil	9245	Itaguaje	0.34	0.76	0.97	2139	183	0
BRA	Brazil	9246	Itaipulandia	0.32	0.78	0.97	5183	209	0
BRA	Brazil	9247	Itambaraca	0.39	0.81	0.95	2802	0	0
BRA	Brazil	9248	Itambe	0.08	0.40	0.55	4485	2474	1538
BRA	Brazil	9249	Itapejara D'oeste	0.17	0.49	0.65	7393	3612	1764
BRA	Brazil	9250	Itaperucu	0.46	0.75	0.97	8271	1329	0
BRA	Brazil	9251	Itauna Do Sul	0.41	0.84	1.00	1276	0	0

BRA	Brazil	9252	Ivai	0.28	0.69	0.96	7067	1520	0
BRA	Brazil	9253	Ivaipora	0.41	0.79	0.99	12678	243	0
BRA	Brazil	9254	Ivate	0.39	0.77	0.98	3350	203	0
BRA	Brazil	9255	Ivatuba	0.23	0.67	0.86	1760	386	0
BRA	Brazil	9256	Jaboti	0.47	0.78	0.96	1760	122	0
BRA	Brazil	9257	Jacarezinho	0.43	0.79	0.99	14664	326	0
BRA	Brazil	9258	Jaguapita	0.25	0.70	0.93	7325	1349	0
BRA	Brazil	9259	Jaguariaiva	0.33	0.71	0.93	16367	3092	0
BRA	Brazil	9260	Jandaia Do Sul	0.29	0.73	0.93	10703	1571	0
BRA	Brazil	9261	Janiopolis	0.36	0.81	0.99	2558	0	0
BRA	Brazil	9262	Japira	0.31	0.72	0.94	2579	411	0
BRA	Brazil	9263	Japura	0.17	0.49	0.65	5837	2852	1393
BRA	Brazil	9264	Jardim Alegre	0.51	0.82	0.98	3459	0	0
BRA	Brazil	9265	Jardim Olinda	0.54	0.85	0.98	351	0	0
BRA	Brazil	9266	Jataizinho	0.30	0.76	0.96	6001	469	0
BRA	Brazil	9267	Jesuitas	0.38	0.81	0.97	3798	0	0
BRA	Brazil	9268	Joaquim Tavora	0.31	0.74	0.98	5690	737	0
BRA	Brazil	9269	Jundiai Do Sul	0.35	0.74	0.95	1475	188	0
BRA	Brazil	9270	Juranda	0.50	0.81	0.97	2244	0	0
BRA	Brazil	9271	Jussara	0.17	0.49	0.65	4439	2169	1059
BRA	Brazil	9272	Kalore	0.08	0.40	0.55	3192	1761	1094
BRA	Brazil	9273	Lapa	0.21	0.62	0.88	28499	8835	0
BRA	Brazil	9274	Laranjal	0.17	0.49	0.65	4619	2257	1102
BRA	Brazil	9275	Laranjeiras Do Sul	0.44	0.85	1.00	11262	0	0
BRA	Brazil	9276	Leopolis	0.35	0.70	0.90	1892	409	0
BRA	Brazil	9277	Lidianopolis	0.43	0.82	0.99	1454	0	0
BRA	Brazil	9278	Lindoeste	0.34	0.77	0.99	2316	153	0
BRA	Brazil	9279	Loanda	0.30	0.70	0.94	11688	2299	0
BRA	Brazil	9280	Lobato	0.24	0.70	0.92	2640	496	0
BRA	Brazil	9281	Londrina	0.23	0.68	0.92	318926	67926	0
BRA	Brazil	9282	Luiziana	0.45	0.80	0.99	2588	17	0
BRA	Brazil	9283	Lunardelli	0.40	0.82	0.98	1989	0	0
BRA	Brazil	9284	Lupionopolis	0.39	0.75	0.96	2016	236	0

BRA	Brazil	9285	Mallet	0.47	0.77	0.95	4726	458	0
BRA	Brazil	9286	Mambore	0.55	0.82	0.99	3439	0	0
BRA	Brazil	9287	Mandaguacu	0.24	0.71	0.94	12577	1986	0
BRA	Brazil	9288	Mandaguari	0.08	0.40	0.55	24923	13750	8545
BRA	Brazil	9289	Mandirituba	0.23	0.63	0.88	15075	4487	0
BRA	Brazil	9290	Manfrinopolis	0.35	0.79	0.98	1352	39	0
BRA	Brazil	9291	Mangueirinha	0.49	0.81	0.99	5313	0	0
BRA	Brazil	9292	Manoel Ribas	0.34	0.81	1.00	6183	0	0
BRA	Brazil	9293	Marechal Candido Rondon	0.35	0.78	0.97	22881	1103	0
BRA	Brazil	9294	Maria Helena	0.32	0.75	0.95	2874	269	0
BRA	Brazil	9295	Marialva	0.33	0.79	0.96	16288	466	0
BRA	Brazil	9296	Marilandia Do Sul	0.33	0.70	0.90	4280	946	0
BRA	Brazil	9297	Marilena	0.42	0.83	0.99	2654	0	0
BRA	Brazil	9298	Mariluz	0.33	0.74	0.98	4973	659	0
BRA	Brazil	9299	Maringa	0.25	0.75	0.95	227006	21865	0
BRA	Brazil	9300	Mariopolis	0.41	0.72	0.95	2550	503	0
BRA	Brazil	9301	Maripa	0.41	0.81	0.97	2310	0	0
BRA	Brazil	9302	Marmeleiro	0.29	0.75	0.97	7754	804	0
BRA	Brazil	9303	Marquinho	0.48	0.87	1.00	1524	0	0
BRA	Brazil	9304	Marumbi	0.21	0.65	0.91	2802	719	0
BRA	Brazil	9305	Matelandia	0.17	0.49	0.65	9925	4850	2368
BRA	Brazil	9306	Matinhos	0.20	0.64	0.91	19893	5497	0
BRA	Brazil	9307	Mato Rico	0.48	0.82	0.98	1162	0	0
BRA	Brazil	9308	Maua Da Serra	0.25	0.64	0.88	5272	1537	0
BRA	Brazil	9309	Medianeira	0.44	0.80	0.99	15890	181	0
BRA	Brazil	9310	Mercedes	0.41	0.82	0.98	2128	0	0
BRA	Brazil	9311	Mirador	0.17	0.49	0.65	1395	682	333
BRA	Brazil	9312	Miraselva	0.32	0.69	0.92	888	201	0
BRA	Brazil	9313	Missal	0.36	0.82	0.97	4816	0	0
BRA	Brazil	9314	Moreira Sales	0.17	0.49	0.65	7628	3727	1820
BRA	Brazil	9315	Morretes	0.22	0.60	0.82	9516	3271	0
BRA	Brazil	9316	Munhoz De Melo	0.30	0.73	0.91	1963	275	0
BRA	Brazil	9317	Nossa Senhora Das Gracas	0.21	0.65	0.89	2337	608	0

BRA	Brazil	9318	Nova Alianca Do Ivai	0.52	0.83	0.99	435	0	0
BRA	Brazil	9319	Nova America Da Colina	0.48	0.78	0.96	1100	85	0
BRA	Brazil	9320	Nova Aurora	0.17	0.49	0.65	7397	3614	1765
BRA	Brazil	9321	Nova Cantu	0.51	0.80	0.97	1894	3	0
BRA	Brazil	9322	Nova Esperanca	0.35	0.80	0.97	12670	34	0
BRA	Brazil	9323	Nova Esperanca Do Sudoeste	0.39	0.77	0.99	2119	179	0
BRA	Brazil	9324	Nova Fatima	0.08	0.40	0.55	5990	3305	2054
BRA	Brazil	9325	Nova Laranjeiras	0.39	0.84	1.00	5051	0	0
BRA	Brazil	9326	Nova Londrina	0.36	0.79	0.98	5865	143	0
BRA	Brazil	9327	Nova Olimpia	0.17	0.49	0.65	3633	1775	867
BRA	Brazil	9328	Nova Prata Do Iguacu	0.47	0.80	0.98	3512	0	0
BRA	Brazil	9329	Nova Santa Barbara	0.46	0.77	0.96	1435	119	0
BRA	Brazil	9330	Nova Santa Rosa	0.46	0.83	0.98	2736	0	0
BRA	Brazil	9331	Nova Tebas	0.59	0.83	0.98	1435	0	0
BRA	Brazil	9332	Novo Itacolomi	0.30	0.66	0.88	1462	404	0
BRA	Brazil	9333	Ortigueira	0.38	0.72	0.94	9790	1942	0
BRA	Brazil	9334	Ourizona	0.31	0.73	0.92	1714	231	0
BRA	Brazil	9335	Ouro Verde Do Oeste	0.36	0.78	0.98	2648	117	0
BRA	Brazil	9336	Paicandu	0.25	0.74	0.98	20305	2289	0
BRA	Brazil	9337	Palmas	0.99	1.00	1.00	0	0	0
BRA	Brazil	9338	Palmeira	0.26	0.75	0.98	18246	1766	0
BRA	Brazil	9339	Palmital	0.64	0.97	1.00	2133	0	0
BRA	Brazil	9340	Palotina	0.40	0.78	0.99	12239	687	0
BRA	Brazil	9341	Paraiso Do Norte	0.46	0.77	0.97	4540	421	0
BRA	Brazil	9342	Paranacity	0.17	0.49	0.65	7144	3491	1704
BRA	Brazil	9343	Paranagua	0.23	0.66	0.91	83636	20963	0
BRA	Brazil	9344	Paranapoema	0.35	0.77	0.98	1406	102	0
BRA	Brazil	9345	Paranavai	0.31	0.80	0.99	42831	335	0
BRA	Brazil	9346	Pato Bragado	0.50	0.82	0.98	1571	0	0
BRA	Brazil	9347	Pato Branco	0.56	0.81	0.98	18914	0	0
BRA	Brazil	9348	Paula Freitas	0.60	0.80	0.91	1181	0	0
BRA	Brazil	9349	Paulo Frontin	0.63	0.78	0.87	1229	162	0
BRA	Brazil	9350	Peabiru	0.39	0.75	0.96	5824	737	0

BRA	Brazil	9351	Perobal	0.39	0.77	0.96	2642	199	0
BRA	Brazil	9352	Perola	0.28	0.67	0.93	5742	1389	0
BRA	Brazil	9353	Perola D'oeste	0.17	0.49	0.65	4451	2175	1062
BRA	Brazil	9354	Pien	0.08	0.40	0.55	9109	5025	3123
BRA	Brazil	9355	Pinhais	0.22	0.63	0.88	79434	23009	0
BRA	Brazil	9356	Pinhal De Sao Bento	0.43	0.84	0.99	1020	0	0
BRA	Brazil	9357	Pinhalao	0.48	0.83	0.98	2009	0	0
BRA	Brazil	9358	Pinhao	0.38	0.76	0.99	13326	1263	0
BRA	Brazil	9359	Pirai Do Sul	0.08	0.40	0.55	18118	9996	6212
BRA	Brazil	9360	Piraquara	0.23	0.62	0.84	68557	21971	0
BRA	Brazil	9361	Pitanga	0.82	0.93	0.99	0	0	0
BRA	Brazil	9362	Pitangueiras	1.00	1.00	1.00	0	0	0
BRA	Brazil	9363	Planaltina Do Parana	0.34	0.78	0.98	1973	105	0
BRA	Brazil	9364	Planalto	0.17	0.49	0.65	8683	4243	2071
BRA	Brazil	9365	Ponta Grossa	0.08	0.40	0.55	247848	136744	84979
BRA	Brazil	9366	Pontal Do Parana	0.20	0.63	0.90	15121	4383	0
BRA	Brazil	9367	Porecatu	0.38	0.76	0.97	5753	496	0
BRA	Brazil	9368	Porto Amazonas	0.53	0.78	0.96	1223	99	0
BRA	Brazil	9369	Porto Barreiro	0.53	0.87	1.00	965	0	0
BRA	Brazil	9370	Porto Rico	0.36	0.78	0.97	1151	62	0
BRA	Brazil	9371	Porto Vitoria	0.59	0.78	0.92	812	92	0
BRA	Brazil	9372	Prado Ferreira	0.60	0.82	0.94	757	0	0
BRA	Brazil	9373	Pranchita	0.71	0.88	0.98	526	0	0
BRA	Brazil	9374	Presidente Castelo Branco	0.34	0.76	0.93	2405	217	0
BRA	Brazil	9375	Primeiro De Maio	0.31	0.72	0.95	5465	946	0
BRA	Brazil	9376	Prudentopolis	0.44	0.83	0.99	18908	0	0
BRA	Brazil	9377	Quarto Centenario	0.45	0.80	0.97	1738	0	0
BRA	Brazil	9378	Quatigua	0.17	0.49	0.65	4728	2311	1128
BRA	Brazil	9379	Quatro Barras	0.22	0.64	0.90	12754	3403	0
BRA	Brazil	9380	Quatro Pontes	0.28	0.70	0.94	2713	531	0
BRA	Brazil	9381	Quedas Do Iguacu	0.34	0.78	0.99	15200	510	0
BRA	Brazil	9382	Querencia Do Norte	0.36	0.81	0.99	5371	0	0
BRA	Brazil	9383	Quinta Do Sol	0.17	0.49	0.65	3106	1518	741

BRA	Brazil	9384	Quitandinha	0.24	0.66	0.92	10164	2611	0
BRA	Brazil	9385	Ramilandia	0.35	0.75	0.96	2455	282	0
BRA	Brazil	9386	Rancho Alegre	0.31	0.72	0.93	1951	321	0
BRA	Brazil	9387	Rancho Alegre D'oeste	0.39	0.77	0.97	1169	92	0
BRA	Brazil	9388	Realeza	0.36	0.76	0.97	7582	713	0
BRA	Brazil	9389	Reboucas	0.42	0.74	0.94	5672	960	0
BRA	Brazil	9390	Renascenca	0.31	0.75	0.97	4037	378	0
BRA	Brazil	9391	Reserva	0.32	0.76	0.99	12714	1115	0
BRA	Brazil	9392	Reserva Do Iguacu	0.63	0.87	1.00	1368	0	0
BRA	Brazil	9393	Ribeirao Claro	0.81	0.92	0.99	0	0	0
BRA	Brazil	9394	Ribeirao Do Pinhal	0.40	0.75	0.95	5450	656	0
BRA	Brazil	9395	Rio Azul	0.44	0.76	0.95	5148	583	0
BRA	Brazil	9396	Rio Bom	0.39	0.77	0.93	1368	100	0
BRA	Brazil	9397	Rio Bonito Do Iguacu	0.48	0.86	1.00	4417	0	0
BRA	Brazil	9398	Rio Branco Do Ivai	0.17	0.49	0.65	2208	1079	527
BRA	Brazil	9399	Rio Branco Do Sul	0.08	0.40	0.55	24838	13704	8516
BRA	Brazil	9400	Rio Negro	0.08	0.40	0.55	24047	13268	8245
BRA	Brazil	9401	Rolandia	0.23	0.68	0.92	37245	7936	0
BRA	Brazil	9402	Roncador	0.51	0.79	0.97	3193	78	0
BRA	Brazil	9403	Rondon	0.30	0.73	0.98	4834	630	0
BRA	Brazil	9404	Rosario Do Ivai	0.53	0.80	0.98	1607	12	0
BRA	Brazil	9405	Sabaudia	0.29	0.65	0.88	3410	969	0
BRA	Brazil	9406	Salgado Filho	0.49	0.83	0.97	1287	0	0
BRA	Brazil	9407	Salto Do Itarare	0.95	0.96	0.98	0	0	0
BRA	Brazil	9408	Salto Do Lontra	0.34	0.75	0.97	6776	695	0
BRA	Brazil	9409	Santa Amelia	0.31	0.72	0.95	1783	280	0
BRA	Brazil	9410	Santa Cecilia Do Pavao	0.48	0.76	0.93	1119	147	0
BRA	Brazil	9411	Santa Cruz De Monte Castelo	0.35	0.78	0.98	3645	197	0
BRA	Brazil	9412	Santa Fe	0.30	0.71	0.90	5876	1083	0
BRA	Brazil	9413	Santa Helena	0.17	0.49	0.65	16206	7919	3866
BRA	Brazil	9414	Santa Ines	0.17	0.49	0.65	1098	536	262
BRA	Brazil	9415	Santa Isabel Do Ivai	0.34	0.77	0.97	3834	222	0
BRA	Brazil	9416	Santa Izabel Do Oeste	0.45	0.81	0.97	4983	0	0

BRA	Brazil	9417	Santa Lucia	0.35	0.75	0.96	2100	248	0
BRA	Brazil	9418	Santa Maria Do Oeste	0.51	0.79	0.98	3118	60	0
BRA	Brazil	9419	Santa Mariana	0.47	0.75	0.93	4133	645	0
BRA	Brazil	9420	Santa Monica	0.44	0.80	0.98	1421	0	0
BRA	Brazil	9421	Santa Tereza Do Oeste	0.34	0.79	0.99	4845	146	0
BRA	Brazil	9422	Santa Terezinha De Itaipu	0.34	0.75	0.97	13587	1378	0
BRA	Brazil	9423	Santana Do Itarare	0.85	0.93	0.98	0	0	0
BRA	Brazil	9424	Santo Antonio Da Platina	0.31	0.74	0.97	22278	2902	0
BRA	Brazil	9425	Santo Antonio Do Caiua	0.41	0.82	0.98	1076	0	0
BRA	Brazil	9426	Santo Antonio Do Paraiso	0.53	0.78	0.94	616	35	0
BRA	Brazil	9427	Santo Antonio Do Sudoeste	0.41	0.79	1.00	5087	78	0
BRA	Brazil	9428	Santo Inacio	0.40	0.82	0.98	2178	0	0
BRA	Brazil	9429	Sao Carlos Do Ivai	0.36	0.74	0.97	2956	385	0
BRA	Brazil	9430	Sao Jeronimo Da Serra	0.27	0.71	0.96	6057	988	0
BRA	Brazil	9431	Sao Joao	0.36	0.69	0.94	4680	1205	0
BRA	Brazil	9432	Sao Joao Do Caiua	0.33	0.77	0.98	2796	176	0
BRA	Brazil	9433	Sao Joao Do Ivai	0.39	0.74	0.98	4570	631	0
BRA	Brazil	9434	Sao Joao Do Triunfo	0.22	0.62	0.86	8596	2667	0
BRA	Brazil	9435	Sao Jorge D'oeste	0.42	0.77	0.97	3536	249	0
BRA	Brazil	9436	Sao Jorge Do Ivai	0.48	0.78	0.96	1827	120	0
BRA	Brazil	9437	Sao Jorge Do Patrocinio	0.35	0.75	0.96	2693	277	0
BRA	Brazil	9438	Sao Jose Da Boa Vista	0.75	0.88	0.98	314	0	0
BRA	Brazil	9439	Sao Jose Das Palmeiras	0.17	0.49	0.65	2378	1162	567
BRA	Brazil	9440	Sao Jose Dos Pinhais	0.21	0.63	0.91	183399	51734	0
BRA	Brazil	9441	Sao Manoel Do Parana	0.48	0.78	0.96	667	34	0
BRA	Brazil	9442	Sao Mateus Do Sul	0.54	0.77	0.93	11959	1320	0
BRA	Brazil	9443	Sao Miguel Do Iguacu	0.30	0.75	0.98	13711	1281	0
BRA	Brazil	9444	Sao Pedro Do Iguacu	0.43	0.80	0.97	2272	0	0
BRA	Brazil	9445	Sao Pedro Do Ivai	0.25	0.65	0.90	5997	1668	0
BRA	Brazil	9446	Sao Pedro Do Parana	0.47	0.81	0.97	827	0	0
BRA	Brazil	9447	Sao Sebastiao Da Amoreira	0.31	0.71	0.93	4377	854	0
BRA	Brazil	9448	Sao Tome	0.46	0.82	0.98	1976	0	0
BRA	Brazil	9449	Sapopema	0.44	0.72	0.90	2425	565	0

BRA	Brazil	9450	Sarandi	0.25	0.77	0.98	50420	2801	0
BRA	Brazil	9451	Saudade Do Iguacu	0.55	0.80	0.98	1319	24	0
BRA	Brazil	9452	Senges	0.41	0.81	1.00	7594	0	0
BRA	Brazil	9453	Serranopolis Do Iguacu	0.39	0.80	0.97	1898	8	0
BRA	Brazil	9454	Sertaneja	0.29	0.71	0.95	2901	511	0
BRA	Brazil	9455	Sertanopolis	0.50	0.75	0.89	4970	781	0
BRA	Brazil	9456	Siqueira Campos	0.72	0.86	0.97	1526	0	0
BRA	Brazil	9457	Sulina	0.54	0.80	0.98	826	0	0
BRA	Brazil	9458	Tamarana	0.29	0.73	0.94	5901	786	0
BRA	Brazil	9459	Tamboara	0.34	0.75	0.97	2337	230	0
BRA	Brazil	9460	Tapejara	0.79	0.92	0.99	95	0	0
BRA	Brazil	9461	Tapira	0.17	0.49	0.65	3658	1787	873
BRA	Brazil	9462	Teixeira Soares	0.49	0.76	0.93	3656	488	0
BRA	Brazil	9463	Telemaco Borba	0.24	0.65	0.90	43442	11429	0
BRA	Brazil	9464	Terra Boa	0.30	0.70	0.96	8513	1629	0
BRA	Brazil	9465	Terra Rica	0.35	0.82	0.98	7398	0	0
BRA	Brazil	9466	Terra Roxa	0.35	0.76	0.98	8033	708	0
BRA	Brazil	9467	Tibagi	0.08	0.40	0.55	15029	8292	5153
BRA	Brazil	9468	Tijucas Do Sul	0.30	0.67	0.91	8084	2133	0
BRA	Brazil	9469	Toledo	0.17	0.49	0.65	85497	41777	20397
BRA	Brazil	9470	Tomazina	0.34	0.73	0.97	4023	616	0
BRA	Brazil	9471	Tres Barras Do Parana	0.17	0.49	0.65	7705	3765	1838
BRA	Brazil	9472	Tunas Do Parana	0.30	0.72	0.97	4220	648	0
BRA	Brazil	9473	Tuneiras Do Oeste	0.29	0.68	0.92	4460	1093	0
BRA	Brazil	9474	Tupassi	0.32	0.76	0.98	3879	289	0
BRA	Brazil	9475	Turvo	0.50	0.89	1.00	3943	0	0
BRA	Brazil	9476	Ubirata	0.43	0.81	0.98	8055	0	0
BRA	Brazil	9477	Umuarama	0.34	0.72	0.95	50632	8442	0
BRA	Brazil	9478	Uniao Da Vitoria	0.76	0.89	0.98	2513	0	0
BRA	Brazil	9479	Uniflor	0.38	0.80	0.98	1077	0	0
BRA	Brazil	9480	Urai	0.46	0.77	0.94	3918	349	0
BRA	Brazil	9481	Ventania	0.23	0.61	0.81	6494	2215	0
BRA	Brazil	9482	Vera Cruz Do Oeste	0.45	0.83	0.98	3052	0	0

BRA	Brazil	9483	Vere	0.33	0.75	0.96	3751	403	0
BRA	Brazil	9484	Vila Alta	0.17	0.49	0.65	1953	954	466
BRA	Brazil	9485	Virmond	0.44	0.86	0.99	1481	0	0
BRA	Brazil	9486	Vitorino	0.49	0.74	0.93	2284	453	0
BRA	Brazil	9487	Wenceslau Braz	0.08	0.40	0.55	14237	7855	4881
BRA	Brazil	9488	Xambre	0.33	0.72	0.97	2763	462	0
BRA	Brazil	9489	Abreu E Lima	0.08	0.40	0.55	71506	39452	24517
BRA	Brazil	9490	Afogados Da Ingazeira	0.08	0.40	0.55	27195	15004	9324
BRA	Brazil	9491	Afranio	0.08	0.40	0.55	13992	7720	4797
BRA	Brazil	9492	Agrestina	0.08	0.40	0.55	17905	9879	6139
BRA	Brazil	9493	Agua Preta	0.08	0.40	0.55	26558	14653	9106
BRA	Brazil	9494	Aguas Belas	0.08	0.40	0.55	31290	17264	10728
BRA	Brazil	9495	Alagoinha	0.08	0.40	0.55	9089	5015	3116
BRA	Brazil	9496	Alianca	0.08	0.40	0.55	27276	15049	9352
BRA	Brazil	9497	Altinho	0.08	0.40	0.55	16893	9320	5792
BRA	Brazil	9498	Amaraji	0.08	0.40	0.55	17029	9395	5839
BRA	Brazil	9499	Angelim	0.08	0.40	0.55	8364	4615	2868
BRA	Brazil	9500	Aracoiaba	0.08	0.40	0.55	14853	8195	5093
BRA	Brazil	9501	Araripina	0.08	0.40	0.55	59669	32921	20459
BRA	Brazil	9502	Arcoverde	0.08	0.40	0.55	54388	30007	18648
BRA	Brazil	9503	Barra De Guabiraba	0.08	0.40	0.55	10350	5710	3549
BRA	Brazil	9504	Barreiros	0.08	0.40	0.55	31058	17135	10649
BRA	Brazil	9505	Belem De Maria	0.08	0.40	0.55	5277	2912	1809
BRA	Brazil	9506	Belem De Sao Francisco	0.08	0.40	0.55	15638	8628	5362
BRA	Brazil	9507	Belo Jardim	0.08	0.40	0.55	55530	30637	19039
BRA	Brazil	9508	Betania	0.08	0.40	0.55	9457	5218	3242
BRA	Brazil	9509	Bezerros	0.08	0.40	0.55	45415	25056	15571
BRA	Brazil	9510	Bodoco	0.08	0.40	0.55	26443	14589	9066
BRA	Brazil	9511	Bom Conselho	0.08	0.40	0.55	35361	19509	12124
BRA	Brazil	9512	Bom Jardim	0.08	0.40	0.55	26361	14544	9038
BRA	Brazil	9513	Bonito	0.08	0.40	0.55	32102	17711	11007
BRA	Brazil	9514	Brejao	0.08	0.40	0.55	6550	3614	2246
BRA	Brazil	9515	Brejinho	0.08	0.40	0.55	3897	2150	1336

BRA	Brazil	9516	Brejo Da Madre De Deus	0.08	0.40	0.55	40410	22295	13855
BRA	Brazil	9517	Buenos Aires	0.08	0.40	0.55	9279	5119	3181
BRA	Brazil	9518	Buique	0.08	0.40	0.55	41892	23113	14363
BRA	Brazil	9519	Cabo De Santo Agostinho	0.08	0.40	0.55	141752	78208	48602
BRA	Brazil	9520	Cabrobo	0.08	0.40	0.55	25272	13943	8665
BRA	Brazil	9521	Cachoeirinha	1.00	1.00	1.00	0	0	0
BRA	Brazil	9522	Caetes	0.08	0.40	0.55	17045	9404	5844
BRA	Brazil	9523	Calcado	0.08	0.40	0.55	4941	2726	1694
BRA	Brazil	9524	Calumbi	0.08	0.40	0.55	4397	2426	1507
BRA	Brazil	9525	Camaragibe	0.08	0.40	0.55	93556	51617	32077
BRA	Brazil	9526	Camocim De Sao Felix	0.08	0.40	0.55	12788	7056	4385
BRA	Brazil	9527	Camutanga	0.08	0.40	0.55	4778	2636	1638
BRA	Brazil	9528	Canhotinho	0.08	0.40	0.55	18773	10358	6437
BRA	Brazil	9529	Capoeiras	0.08	0.40	0.55	16734	9232	5737
BRA	Brazil	9530	Carnaiba	0.08	0.40	0.55	14641	8078	5020
BRA	Brazil	9531	Carnaubeira Da Penha	0.08	0.40	0.55	9331	5148	3199
BRA	Brazil	9532	Carpina	0.08	0.40	0.55	60968	33637	20904
BRA	Brazil	9533	Caruaru	0.08	0.40	0.55	266723	147158	91451
BRA	Brazil	9534	Casinhas	0.08	0.40	0.55	9347	5157	3205
BRA	Brazil	9535	Catende	0.08	0.40	0.55	28927	15960	9918
BRA	Brazil	9536	Cedro	0.08	0.40	0.55	8657	4776	2968
BRA	Brazil	9537	Cha De Alegria	0.08	0.40	0.55	9659	5329	3312
BRA	Brazil	9538	Cha Grande	0.08	0.40	0.55	15057	8308	5163
BRA	Brazil	9539	Condado	0.08	0.40	0.55	19429	10720	6662
BRA	Brazil	9540	Correntes	0.08	0.40	0.55	13448	7419	4611
BRA	Brazil	9541	Cortes	0.08	0.40	0.55	4641	2561	1591
BRA	Brazil	9542	Cumaru	0.08	0.40	0.55	9613	5304	3296
BRA	Brazil	9543	Cupira	0.08	0.40	0.55	18139	10008	6219
BRA	Brazil	9544	Custodia	0.08	0.40	0.55	27102	14953	9293
BRA	Brazil	9545	Dormentes	0.08	0.40	0.55	12993	7169	4455
BRA	Brazil	9546	Escada	0.08	0.40	0.55	51707	28528	17729
BRA	Brazil	9547	Exu	0.08	0.40	0.55	23807	13135	8163
BRA	Brazil	9548	Feira Nova	0.08	0.40	0.55	16521	9115	5664

BRA	Brazil	9550	Ferreiros	0.08	0.40	0.55	9534	5260	3269
BRA	Brazil	9551	Flores	0.08	0.40	0.55	16465	9084	5645
BRA	Brazil	9552	Floresta	0.08	0.40	0.55	23843	13155	8175
BRA	Brazil	9553	Frei Miguelinho	0.08	0.40	0.55	11681	6445	4005
BRA	Brazil	9554	Gameleira	0.08	0.40	0.55	22422	12371	7688
BRA	Brazil	9555	Garanhuns	0.08	0.40	0.55	100310	55344	34393
BRA	Brazil	9556	Gloria Do Goita	0.08	0.40	0.55	20306	11203	6962
BRA	Brazil	9557	Goiana	0.08	0.40	0.55	57489	31718	19711
BRA	Brazil	9558	Granito	0.08	0.40	0.55	5095	2811	1747
BRA	Brazil	9559	Gravata	0.08	0.40	0.55	60800	33545	20846
BRA	Brazil	9560	Iati	0.08	0.40	0.55	13074	7213	4483
BRA	Brazil	9561	Ibimirim	0.08	0.40	0.55	21460	11840	7358
BRA	Brazil	9562	Ibirajuba	0.08	0.40	0.55	6001	3311	2057
BRA	Brazil	9563	Igarassu	0.08	0.40	0.55	85549	47200	29332
BRA	Brazil	9564	Iguaraci	0.08	0.40	0.55	8672	4785	2973
BRA	Brazil	9565	Inaja	0.08	0.40	0.55	14489	7994	4968
BRA	Brazil	9566	Ingazeira	0.08	0.40	0.55	3129	1727	1073
BRA	Brazil	9567	Ipojuca	0.08	0.40	0.55	68330	37699	23428
BRA	Brazil	9568	Ipubi	0.08	0.40	0.55	25269	13942	8664
BRA	Brazil	9569	Itacuruba	0.08	0.40	0.55	3561	1965	1221
BRA	Brazil	9570	Itaiba	0.08	0.40	0.55	18750	10345	6429
BRA	Brazil	9571	Itamaraca	0.08	0.40	0.55	18890	10422	6477
BRA	Brazil	9572	Itambe	0.08	0.40	0.55	26073	14385	8940
BRA	Brazil	9573	Itapetim	0.08	0.40	0.55	10670	5887	3658
BRA	Brazil	9574	Itapissuma	0.08	0.40	0.55	18733	10335	6423
BRA	Brazil	9575	Itaquitinga	0.08	0.40	0.55	10900	6014	3737
BRA	Brazil	9576	Jaboatao Dos Guararapes	0.08	0.40	0.55	499648	275668	171313
BRA	Brazil	9577	Jaqueira	0.08	0.40	0.55	12433	6860	4263
BRA	Brazil	9578	Jatauba	0.08	0.40	0.55	12618	6962	4326
BRA	Brazil	9579	Jatoba	0.08	0.40	0.55	10871	5998	3727
BRA	Brazil	9580	Joao Alfredo	0.08	0.40	0.55	25068	13831	8595
BRA	Brazil	9581	Joaquim Nabuco	0.08	0.40	0.55	11605	6403	3979
BRA	Brazil	9582	Jucati	0.08	0.40	0.55	8078	4457	2770

BRA	Brazil	9583	Jupi	0.08	0.40	0.55	12689	7001	4350
BRA	Brazil	9584	Jurema	0.08	0.40	0.55	11191	6175	3837
BRA	Brazil	9585	Lagoa Do Carro	0.08	0.40	0.55	12392	6837	4249
BRA	Brazil	9586	Lagoa Do Itaenga	0.08	0.40	0.55	17160	9468	5884
BRA	Brazil	9587	Lagoa Do Ouro	0.08	0.40	0.55	9778	5395	3353
BRA	Brazil	9588	Lagoa Dos Gatos	0.08	0.40	0.55	13871	7653	4756
BRA	Brazil	9589	Lagoa Grande	0.08	0.40	0.55	17621	9722	6042
BRA	Brazil	9590	Lajedo	0.08	0.40	0.55	30190	16656	10351
BRA	Brazil	9591	Limoeiro	0.08	0.40	0.55	42807	23618	14677
BRA	Brazil	9592	Macaparana	0.08	0.40	0.55	17161	9468	5884
BRA	Brazil	9593	Machados	0.08	0.40	0.55	7450	4110	2554
BRA	Brazil	9594	Manari	0.08	0.40	0.55	18091	9981	6203
BRA	Brazil	9595	Maraial	0.08	0.40	0.55	5300	2924	1817
BRA	Brazil	9596	Mirandiba	0.08	0.40	0.55	10845	5983	3718
BRA	Brazil	9597	Moreilandia	0.08	0.40	0.55	10085	5564	3458
BRA	Brazil	9598	Moreno	0.08	0.40	0.55	45033	24846	15440
BRA	Brazil	9599	Nazare Da Mata	0.08	0.40	0.55	23921	13198	8202
BRA	Brazil	9600	Olinda	0.08	0.40	0.55	247082	136321	84716
BRA	Brazil	9601	Orobo	0.08	0.40	0.55	15873	8757	5442
BRA	Brazil	9602	Oroco	0.08	0.40	0.55	10900	6014	3737
BRA	Brazil	9603	Ouricuri	0.08	0.40	0.55	50185	27688	17207
BRA	Brazil	9604	Palmares	0.08	0.40	0.55	47004	25934	16116
BRA	Brazil	9605	Palmeirina	0.08	0.40	0.55	6043	3334	2072
BRA	Brazil	9606	Panelas	0.08	0.40	0.55	18977	10470	6506
BRA	Brazil	9607	Paranatama	0.08	0.40	0.55	11324	6248	3883
BRA	Brazil	9608	Parnamirim	0.08	0.40	0.55	15466	8533	5303
BRA	Brazil	9609	Passira	0.08	0.40	0.55	23148	12771	7937
BRA	Brazil	9610	Paudalho	0.08	0.40	0.55	41376	22828	14186
BRA	Brazil	9611	Paulista	0.08	0.40	0.55	273507	150901	93777
BRA	Brazil	9612	Pedra	0.08	0.40	0.55	16498	9102	5656
BRA	Brazil	9613	Pesqueira	0.08	0.40	0.55	50705	27975	17385
BRA	Brazil	9614	Petrolandia	0.08	0.40	0.55	25996	14343	8913
BRA	Brazil	9615	Petrolina	0.08	0.40	0.55	256754	141657	88032

BRA	Brazil	9616	Pocao	0.08	0.40	0.55	7900	4359	2709
BRA	Brazil	9617	Pombos	0.08	0.40	0.55	23164	12780	7942
BRA	Brazil	9618	Primavera	0.08	0.40	0.55	8267	4561	2835
BRA	Brazil	9619	Quipapa	0.08	0.40	0.55	18265	10077	6262
BRA	Brazil	9620	Quixaba	0.08	0.40	0.55	5217	2878	1789
BRA	Brazil	9621	Recife	0.08	0.40	0.55	1203183	663826	412532
BRA	Brazil	9622	Riacho Das Almas	0.08	0.40	0.55	14340	7912	4917
BRA	Brazil	9623	Ribeirao	0.08	0.40	0.55	34791	19195	11929
BRA	Brazil	9624	Rio Formoso	0.08	0.40	0.55	19292	10644	6614
BRA	Brazil	9625	Saire	0.08	0.40	0.55	8558	4722	2934
BRA	Brazil	9626	Salgadinho	0.08	0.40	0.55	6121	3377	2099
BRA	Brazil	9627	Salgueiro	0.08	0.40	0.55	44391	24492	15220
BRA	Brazil	9628	Saloa	0.08	0.40	0.55	12190	6726	4180
BRA	Brazil	9629	Sanharo	0.08	0.40	0.55	18394	10149	6307
BRA	Brazil	9630	Santa Cruz	0.08	0.40	0.55	12197	6729	4182
BRA	Brazil	9631	Santa Cruz Da Baixa Verde	0.08	0.40	0.55	8296	4577	2844
BRA	Brazil	9632	Santa Cruz Do Capibaribe	0.08	0.40	0.55	76625	42276	26272
BRA	Brazil	9633	Santa Filomena	0.16	0.51	0.69	8477	3798	1463
BRA	Brazil	9634	Santa Maria Da Boa Vista	0.08	0.40	0.55	30971	17088	10619
BRA	Brazil	9635	Santa Maria Do Cambuca	0.08	0.40	0.55	9998	5516	3428
BRA	Brazil	9636	Santa Terezinha	0.08	0.40	0.55	9320	5142	3196
BRA	Brazil	9637	Sao Benedito Do Sul	0.08	0.40	0.55	12607	6955	4322
BRA	Brazil	9638	Sao Bento Do Una	0.08	0.40	0.55	43170	23818	14802
BRA	Brazil	9639	Sao Caitano	0.08	0.40	0.55	27262	15041	9347
BRA	Brazil	9640	Sao Joao	0.08	0.40	0.55	15934	8791	5463
BRA	Brazil	9641	Sao Joaquim Do Monte	0.08	0.40	0.55	16123	8896	5528
BRA	Brazil	9642	Sao Jose Da Coroa Grande	0.08	0.40	0.55	14876	8208	5101
BRA	Brazil	9643	Sao Jose Do Belmonte	0.08	0.40	0.55	24794	13679	8501
BRA	Brazil	9644	Sao Jose Do Egito	0.08	0.40	0.55	25719	14190	8818
BRA	Brazil	9645	Sao Lourenco Da Mata	0.08	0.40	0.55	99409	54846	34084
BRA	Brazil	9646	Sao Vicente Ferrer	0.08	0.40	0.55	17265	9526	5920
BRA	Brazil	9647	Serra Talhada	0.08	0.40	0.55	63176	34856	21661
BRA	Brazil	9648	Serrita	0.08	0.40	0.55	15717	8671	5389

BRA	Brazil	9649	Sertania	0.08	0.40	0.55	25515	14077	8748
BRA	Brazil	9650	Sirinhaem	0.08	0.40	0.55	33718	18603	11561
BRA	Brazil	9651	Solidao	0.08	0.40	0.55	3973	2192	1362
BRA	Brazil	9652	Surubim	0.08	0.40	0.55	47631	26279	16331
BRA	Brazil	9653	Tabira	0.08	0.40	0.55	20936	11551	7178
BRA	Brazil	9654	Tacaimbo	0.08	0.40	0.55	8958	4943	3072
BRA	Brazil	9655	Tacaratu	0.08	0.40	0.55	19085	10529	6543
BRA	Brazil	9656	Tamandare	0.08	0.40	0.55	13320	7349	4567
BRA	Brazil	9657	Taquaritinga Do Norte	0.08	0.40	0.55	20551	11339	7046
BRA	Brazil	9658	Terezinha	0.08	0.40	0.55	4987	2751	1710
BRA	Brazil	9659	Terra Nova	0.08	0.40	0.55	7066	3899	2423
BRA	Brazil	9660	Timbauba	0.08	0.40	0.55	40671	22439	13945
BRA	Brazil	9661	Toritama	0.08	0.40	0.55	29702	16387	10184
BRA	Brazil	9662	Tracunhaem	0.08	0.40	0.55	10706	5907	3671
BRA	Brazil	9663	Trindade	0.08	0.40	0.55	21268	11734	7292
BRA	Brazil	9664	Triunfo	0.08	0.40	0.55	10612	5855	3638
BRA	Brazil	9665	Tupanatinga	0.08	0.40	0.55	17770	9804	6093
BRA	Brazil	9666	Tuparetama	0.08	0.40	0.55	5024	2772	1722
BRA	Brazil	9667	Venturosa	0.08	0.40	0.55	13255	7313	4545
BRA	Brazil	9668	Verdejante	0.08	0.40	0.55	6697	3695	2296
BRA	Brazil	9669	Vertente Do Lerio	0.08	0.40	0.55	6275	3462	2152
BRA	Brazil	9670	Vertentes	0.08	0.40	0.55	13698	7558	4697
BRA	Brazil	9671	Vicencia	0.08	0.40	0.55	24245	13376	8313
BRA	Brazil	9672	Vitoria De Santo Antao	0.08	0.40	0.55	97094	53569	33290
BRA	Brazil	9673	Xexeu	0.08	0.40	0.55	10081	5562	3456
BRA	Brazil	9674	Acaua	0.08	0.40	0.55	4598	2537	1577
BRA	Brazil	9675	Agricolandia	0.08	0.40	0.55	3827	2111	1312
BRA	Brazil	9676	Agua Branca	0.08	0.40	0.55	12456	6872	4271
BRA	Brazil	9677	Alagoinha Do Piaui	0.08	0.40	0.55	5256	2900	1802
BRA	Brazil	9678	Alegrete Do Piaui	0.08	0.40	0.55	3511	1937	1204
BRA	Brazil	9679	Alto Longa	0.08	0.40	0.55	10662	5882	3656
BRA	Brazil	9680	Altos	0.21	0.57	0.76	26698	10364	2029
BRA	Brazil	9681	Alvorada Do Gurgueia	0.17	0.49	0.65	3547	1733	846

BRA	Brazil	9682	Amarante	0.17	0.49	0.65	11490	5615	2741
BRA	Brazil	9683	Angical Do Piaui	0.24	0.67	0.92	3822	897	0
BRA	Brazil	9684	Anisio De Abreu	0.51	0.77	0.97	2850	303	0
BRA	Brazil	9685	Antonio Almeida	0.17	0.49	0.65	2025	989	483
BRA	Brazil	9686	Aroazes	0.08	0.40	0.55	4250	2345	1457
BRA	Brazil	9687	Arraial	0.08	0.40	0.55	3314	1829	1136
BRA	Brazil	9688	Assuncao Do Piaui	0.24	0.58	0.75	4398	1716	386
BRA	Brazil	9689	Avelino Lopes	0.31	0.68	0.89	5761	1453	0
BRA	Brazil	9690	Baixa Grande Do Ribeiro	0.17	0.49	0.65	7982	3900	1904
BRA	Brazil	9691	Barra D'alcantara	0.08	0.40	0.55	3015	1664	1034
BRA	Brazil	9692	Barras	0.23	0.63	0.87	28262	8275	0
BRA	Brazil	9693	Barreiras Do Piaui	0.17	0.49	0.65	2095	1024	500
BRA	Brazil	9694	Barro Duro	0.08	0.40	0.55	4884	2695	1675
BRA	Brazil	9695	Batalha	0.08	0.40	0.55	19600	10814	6720
BRA	Brazil	9696	Bela Vista Do Piaui	0.08	0.40	0.55	3061	1689	1050
BRA	Brazil	9697	Belem Do Piaui	0.08	0.40	0.55	2669	1473	915
BRA	Brazil	9698	Beneditinos	0.21	0.59	0.79	6089	2222	115
BRA	Brazil	9699	Bertolinia	0.17	0.49	0.65	3508	1714	837
BRA	Brazil	9700	Betania Do Piaui	0.08	0.40	0.55	6384	3522	2189
BRA	Brazil	9701	Boa Hora	0.08	0.40	0.55	5121	2825	1756
BRA	Brazil	9702	Bocaina	0.55	0.94	1.00	1141	0	0
BRA	Brazil	9703	Bom Jesus	0.17	0.49	0.65	17692	8645	4221
BRA	Brazil	9704	Bom Principio Do Piaui	0.23	0.60	0.82	4896	1695	0
BRA	Brazil	9705	Bonfim Do Piaui	0.17	0.49	0.65	4411	2156	1052
BRA	Brazil	9706	Boqueirao Do Piaui	0.08	0.40	0.55	4929	2719	1690
BRA	Brazil	9707	Brasileira	0.21	0.63	0.89	5581	1595	0
BRA	Brazil	9708	Brejo Do Piaui	0.17	0.49	0.65	1708	835	407
BRA	Brazil	9709	Buriti Dos Lopes	0.17	0.49	0.65	11632	5684	2775
BRA	Brazil	9710	Buriti Dos Montes	0.08	0.40	0.55	5710	3150	1958
BRA	Brazil	9711	Cabeceiras Do Piaui	0.08	0.40	0.55	8947	4936	3068
BRA	Brazil	9712	Cajazeiras Do Piaui	0.08	0.40	0.55	2771	1529	950
BRA	Brazil	9713	Cajueiro Da Praia	0.08	0.40	0.55	5496	3032	1884
BRA	Brazil	9714	Caldeirao Grande Do Piaui	0.08	0.40	0.55	3991	2202	1368

BRA	Brazil	9715	Campinas Do Piaui	0.08	0.40	0.55	4254	2347	1459
BRA	Brazil	9716	Campo Alegre Do Fidalgo	0.08	0.40	0.55	3974	2193	1363
BRA	Brazil	9717	Campo Grande Do Piaui	0.08	0.40	0.55	4585	2530	1572
BRA	Brazil	9718	Campo Largo Do Piaui	0.08	0.40	0.55	5056	2789	1733
BRA	Brazil	9719	Campo Maior	0.22	0.64	0.91	27469	7406	0
BRA	Brazil	9720	Canavieira	0.31	0.68	0.89	1929	485	0
BRA	Brazil	9721	Canto Do Buriti	0.17	0.49	0.65	13801	6744	3293
BRA	Brazil	9722	Capitao De Campos	0.08	0.40	0.55	8378	4623	2873
BRA	Brazil	9723	Capitao Gervasio Oliveira	0.18	0.55	0.76	3266	1303	226
BRA	Brazil	9724	Caracol	0.17	0.49	0.65	6712	3280	1601
BRA	Brazil	9725	Caraubas Do Piaui	0.08	0.40	0.55	4344	2397	1490
BRA	Brazil	9726	Caridade Do Piaui	0.08	0.40	0.55	3459	1908	1186
BRA	Brazil	9727	Castelo Do Piaui	0.08	0.40	0.55	14013	7731	4804
BRA	Brazil	9728	Caxingo	0.08	0.40	0.55	4164	2298	1428
BRA	Brazil	9729	Cocal	0.08	0.40	0.55	16972	9364	5819
BRA	Brazil	9730	Cocal De Telha	0.08	0.40	0.55	3724	2054	1277
BRA	Brazil	9731	Cocal Dos Alves	0.08	0.40	0.55	4268	2355	1463
BRA	Brazil	9732	Coivaras	0.20	0.53	0.68	2971	1347	575
BRA	Brazil	9733	Colonia Do Gurgueia	0.17	0.49	0.65	4297	2100	1025
BRA	Brazil	9734	Colonia Do Piaui	0.08	0.40	0.55	5588	3083	1916
BRA	Brazil	9735	Conceicao Do Caninde	0.25	0.59	0.78	2563	961	105
BRA	Brazil	9736	Coronel Jose Dias	0.64	0.78	0.88	677	78	0
BRA	Brazil	9737	Corrente	0.31	0.70	0.93	13301	2836	0
BRA	Brazil	9738	Cristalandia Do Piaui	0.39	0.71	0.96	3690	792	0
BRA	Brazil	9739	Cristino Castro	0.30	0.65	0.84	5459	1646	0
BRA	Brazil	9740	Curimata	0.32	0.67	0.87	5738	1521	0
BRA	Brazil	9741	Currais	0.17	0.49	0.65	3163	1546	755
BRA	Brazil	9742	Curral Novo Do Piaui	0.08	0.40	0.55	4161	2296	1427
BRA	Brazil	9743	Curralinhos	0.08	0.40	0.55	3443	1899	1180
BRA	Brazil	9744	Demerval Lobao	0.24	0.62	0.84	8173	2558	0
BRA	Brazil	9745	Dirceu Arcoverde	0.17	0.49	0.65	4606	2251	1099
BRA	Brazil	9746	Dom Expedito Lopes	0.08	0.40	0.55	4924	2717	1688
BRA	Brazil	9747	Dom Inocencio	0.08	0.40	0.55	6128	3381	2101

BRA	Brazil	9748	Domingos Mourao	0.23	0.59	0.78	2764	1028	115
BRA	Brazil	9749	Elesbao Veloso	0.21	0.57	0.75	8389	3315	675
BRA	Brazil	9750	Eliseu Martins	0.17	0.49	0.65	3166	1547	755
BRA	Brazil	9751	Esperantina	0.24	0.66	0.91	22749	5478	0
BRA	Brazil	9752	Fartura Do Piaui	0.17	0.49	0.65	3526	1723	841
BRA	Brazil	9753	Flores Do Piaui	0.17	0.49	0.65	2947	1440	703
BRA	Brazil	9754	Floresta Do Piaui	0.08	0.40	0.55	1672	922	573
BRA	Brazil	9755	Floriano	0.17	0.49	0.65	37787	18464	9015
BRA	Brazil	9756	Francinopolis	0.08	0.40	0.55	3843	2120	1318
BRA	Brazil	9757	Francisco Ayres	0.08	0.40	0.55	3050	1683	1046
BRA	Brazil	9758	Francisco Macedo	0.08	0.40	0.55	2209	1219	757
BRA	Brazil	9759	Francisco Santos	0.08	0.40	0.55	7533	4156	2583
BRA	Brazil	9760	Fronteiras	0.08	0.40	0.55	9102	5022	3121
BRA	Brazil	9761	Geminiano	0.17	0.62	0.86	4459	1265	0
BRA	Brazil	9762	Gilbues	0.32	0.76	1.00	5280	489	0
BRA	Brazil	9763	Guadalupe	0.31	0.70	0.94	5130	1070	0
BRA	Brazil	9764	Guaribas	0.17	0.49	0.65	3446	1684	822
BRA	Brazil	9765	Hugo Napoleao	0.08	0.40	0.55	3040	1677	1042
BRA	Brazil	9766	Ilha Grande	0.17	0.49	0.65	6191	3025	1477
BRA	Brazil	9767	Inhuma	0.08	0.40	0.55	11289	6229	3871
BRA	Brazil	9768	Ipiranga Do Piaui	0.08	0.40	0.55	7061	3896	2421
BRA	Brazil	9769	Isaias Coelho	0.08	0.40	0.55	6027	3325	2066
BRA	Brazil	9770	Itainopolis	0.19	0.55	0.74	6624	2696	614
BRA	Brazil	9771	Itaueira	0.17	0.49	0.65	7102	3470	1694
BRA	Brazil	9772	Jacobina Do Piaui	0.08	0.40	0.55	4776	2635	1637
BRA	Brazil	9773	Jaicos	0.08	0.40	0.55	14450	7972	4954
BRA	Brazil	9774	Jardim Do Mulato	0.08	0.40	0.55	3147	1736	1079
BRA	Brazil	9775	Jatoba Do Piaui	0.24	0.66	0.92	2789	709	0
BRA	Brazil	9776	Jerumenha	0.32	0.66	0.88	2019	574	0
BRA	Brazil	9777	Joao Costa	0.08	0.40	0.55	2248	1240	771
BRA	Brazil	9778	Joaquim Pires	0.17	0.49	0.65	9441	4613	2252
BRA	Brazil	9779	Joca Marques	0.17	0.49	0.65	3324	1624	793
BRA	Brazil	9780	Jose De Freitas	0.08	0.40	0.55	30256	16693	10374

BRA	Brazil	9781	Juazeiro Do Piaui	0.23	0.58	0.75	2753	1067	248
BRA	Brazil	9782	Julio Borges	0.30	0.67	0.89	2949	767	0
BRA	Brazil	9783	Jurema	0.17	0.49	0.65	2860	1398	682
BRA	Brazil	9784	Lagoa Alegre	0.08	0.40	0.55	4907	2707	1682
BRA	Brazil	9785	Lagoa De Sao Francisco	0.08	0.40	0.55	5005	2761	1716
BRA	Brazil	9786	Lagoa Do Barro Do Piaui	0.08	0.40	0.55	3659	2019	1255
BRA	Brazil	9787	Lagoa Do Piaui	0.08	0.40	0.55	3200	1766	1097
BRA	Brazil	9788	Lagoa Do Sitio	0.25	0.65	0.89	2934	782	0
BRA	Brazil	9789	Lagoinha Do Piaui	0.08	0.40	0.55	2459	1357	843
BRA	Brazil	9790	Landri Sales	0.17	0.49	0.65	3390	1657	809
BRA	Brazil	9791	Luis Correia	0.08	0.40	0.55	20388	11249	6990
BRA	Brazil	9792	Luzilandia	0.17	0.49	0.65	16561	8093	3951
BRA	Brazil	9793	Madeiro	0.30	0.66	0.87	4554	1260	0
BRA	Brazil	9794	Manoel Emidio	0.17	0.49	0.65	3436	1679	820
BRA	Brazil	9795	Marcolandia	0.27	0.74	0.96	5167	619	0
BRA	Brazil	9796	Marcos Parente	0.17	0.49	0.65	2870	1402	685
BRA	Brazil	9797	Massape Do Piaui	0.08	0.40	0.55	4620	2549	1584
BRA	Brazil	9798	Matias Olimpio	0.17	0.49	0.65	7143	3490	1704
BRA	Brazil	9799	Miguel Alves	0.17	0.49	0.65	22440	10965	5354
BRA	Brazil	9800	Miguel Leao	0.08	0.40	0.55	903	498	310
BRA	Brazil	9801	Milton Brandao	0.08	0.40	0.55	4506	2486	1545
BRA	Brazil	9802	Monsenhor Gil	0.08	0.40	0.55	7618	4203	2612
BRA	Brazil	9803	Monsenhor Hipolito	0.24	0.61	0.81	4190	1408	0
BRA	Brazil	9804	Monte Alegre Do Piaui	0.30	0.66	0.86	5221	1460	0
BRA	Brazil	9805	Morro Cabeca No Tempo	0.17	0.49	0.65	2805	1370	669
BRA	Brazil	9806	Morro Do Chapeu Do Piaui	0.08	0.40	0.55	4462	2462	1530
BRA	Brazil	9807	Murici Dos Portelas	0.17	0.49	0.65	5925	2895	1414
BRA	Brazil	9808	Nazare Do Piaui	0.22	0.57	0.75	4213	1672	384
BRA	Brazil	9809	Nossa Senhora De Nazare	0.08	0.40	0.55	3608	1990	1237
BRA	Brazil	9810	Nossa Senhora Dos Remedios	0.08	0.40	0.55	6131	3383	2102
BRA	Brazil	9811	Novo Oriente Do Piaui	0.08	0.40	0.55	4736	2613	1624
BRA	Brazil	9812	Novo Santo Antonio	0.28	0.67	0.92	2003	504	0
BRA	Brazil	9813	Oeiras	0.08	0.40	0.55	27526	15187	9438

BRA	Brazil	9814	Olho D'agua Do Piaui	0.08	0.40	0.55	2128	1174	730
BRA	Brazil	9815	Padre Marcos	0.08	0.40	0.55	5193	2865	1780
BRA	Brazil	9816	Paes Landim	0.08	0.40	0.55	2756	1521	945
BRA	Brazil	9817	Pajeu Do Piaui	0.28	0.65	0.91	2093	586	0
BRA	Brazil	9818	Palmeira Do Piaui	0.17	0.49	0.65	3231	1579	771
BRA	Brazil	9819	Palmeirais	0.17	0.49	0.65	9847	4812	2349
BRA	Brazil	9820	Paqueta	0.20	0.55	0.73	2567	1071	322
BRA	Brazil	9821	Parnagua	0.17	0.49	0.65	6829	3337	1629
BRA	Brazil	9822	Parnaiba	0.17	0.49	0.65	100729	49220	24031
BRA	Brazil	9823	Passagem Franca Do Piaui	0.08	0.40	0.55	3866	2133	1326
BRA	Brazil	9824	Patos Do Piaui	0.08	0.40	0.55	4887	2696	1676
BRA	Brazil	9825	Paulistana	0.27	0.66	0.90	10391	2802	0
BRA	Brazil	9826	Pavussu	0.17	0.49	0.65	2440	1192	582
BRA	Brazil	9827	Pedro Ii	0.08	0.40	0.55	30972	17088	10619
BRA	Brazil	9828	Pedro Laurentino	0.08	0.40	0.55	1887	1041	647
BRA	Brazil	9829	Petronio Portela	0.08	0.40	0.55	3436	1896	1178
BRA	Brazil	9830	Picos	0.08	0.40	0.55	58959	32529	20215
BRA	Brazil	9831	Pimenteiras	0.08	0.40	0.55	8934	4929	3063
BRA	Brazil	9832	Pio Ix	0.08	0.40	0.55	13517	7457	4634
BRA	Brazil	9833	Piracuruca	0.08	0.40	0.55	20869	11514	7155
BRA	Brazil	9834	Piripiri	0.08	0.40	0.55	46285	25536	15869
BRA	Brazil	9835	Porto	0.30	0.65	0.84	6073	1808	0
BRA	Brazil	9836	Porto Alegre Do Piaui	0.17	0.49	0.65	1819	889	434
BRA	Brazil	9837	Prata Do Piaui	0.08	0.40	0.55	2023	1116	694
BRA	Brazil	9838	Queimada Nova	0.37	0.70	0.92	4173	973	0
BRA	Brazil	9839	Redencao Do Gurgueia	0.17	0.49	0.65	5612	2742	1339
BRA	Brazil	9840	Regeneracao	0.24	0.61	0.83	10043	3425	0
BRA	Brazil	9841	Riacho Frio	0.17	0.49	0.65	2828	1382	675
BRA	Brazil	9842	Ribeira Do Piaui	0.18	0.54	0.72	2929	1238	371
BRA	Brazil	9843	Ribeiro Goncalves	0.41	0.76	0.98	2770	287	0
BRA	Brazil	9844	Rio Grande Do Piaui	0.17	0.49	0.65	4130	2018	985
BRA	Brazil	9845	Santa Cruz Do Piaui	0.08	0.40	0.55	4645	2563	1593
BRA	Brazil	9846	Santa Cruz Dos Milagres	0.18	0.54	0.73	2445	1027	276

BRA	Brazil	9847	Santa Filomena	0.17	0.49	0.65	4294	2098	1025
BRA	Brazil	9848	Santa Luz	0.17	0.49	0.65	3762	1838	898
BRA	Brazil	9849	Santa Rosa Do Piaui	0.08	0.40	0.55	3813	2104	1307
BRA	Brazil	9850	Santana Do Piaui	0.08	0.40	0.55	3966	2188	1360
BRA	Brazil	9851	Santo Antonio De Lisboa	0.08	0.40	0.55	4832	2666	1657
BRA	Brazil	9852	Santo Antonio Dos Milagres	0.08	0.40	0.55	1553	857	532
BRA	Brazil	9853	Santo Inacio Do Piaui	0.20	0.58	0.79	2426	908	23
BRA	Brazil	9854	Sao Braz Do Piaui	0.17	0.49	0.65	1929	943	460
BRA	Brazil	9855	Sao Felix Do Piaui	0.08	0.40	0.55	2182	1204	748
BRA	Brazil	9856	Sao Francisco De Assis Do Piaui	0.08	0.40	0.55	4386	2420	1504
BRA	Brazil	9857	Sao Francisco Do Piaui	0.18	0.52	0.68	4088	1873	810
BRA	Brazil	9858	Sao Goncalo Do Gurgueia	0.17	0.49	0.65	1911	934	456
BRA	Brazil	9859	Sao Goncalo Do Piaui	0.08	0.40	0.55	3698	2040	1268
BRA	Brazil	9860	Sao Joao Da Canabrava	0.08	0.40	0.55	4111	2268	1409
BRA	Brazil	9861	Sao Joao Da Fronteira	0.08	0.40	0.55	6363	3511	2182
BRA	Brazil	9862	Sao Joao Da Serra	0.08	0.40	0.55	4308	2377	1477
BRA	Brazil	9863	Sao Joao Da Varjota	0.08	0.40	0.55	3488	1925	1196
BRA	Brazil	9864	Sao Joao Do Arraial	0.25	0.69	0.94	4596	894	0
BRA	Brazil	9865	Sao Joao Do Piaui	0.08	0.40	0.55	15229	8402	5222
BRA	Brazil	9866	Sao Jose Do Divino	0.08	0.40	0.55	3931	2169	1348
BRA	Brazil	9867	Sao Jose Do Peixe	0.08	0.40	0.55	3150	1738	1080
BRA	Brazil	9868	Sao Jose Do Piaui	0.08	0.40	0.55	4448	2454	1525
BRA	Brazil	9869	Sao Juliao	0.23	0.64	0.89	3582	1009	0
BRA	Brazil	9870	Sao Lourenco Do Piaui	0.17	0.49	0.65	2919	1426	696
BRA	Brazil	9871	Sao Luis Do Piaui	0.08	0.40	0.55	1893	1045	649
BRA	Brazil	9872	Sao Miguel Da Baixa Grande	0.08	0.40	0.55	1263	697	433
BRA	Brazil	9873	Sao Miguel Do Fidalgo	0.08	0.40	0.55	2178	1202	747
BRA	Brazil	9874	Sao Miguel Do Tapuio	0.08	0.40	0.55	13870	7653	4756
BRA	Brazil	9875	Sao Pedro Do Piaui	0.22	0.59	0.78	8572	3173	230
BRA	Brazil	9876	Sao Raimundo Nonato	0.48	0.77	0.98	11953	1159	0
BRA	Brazil	9877	Sebastiao Barros	0.32	0.69	0.93	1795	402	0
BRA	Brazil	9878	Sebastiao Leal	0.17	0.49	0.65	2757	1347	658
BRA	Brazil	9879	Sigefredo Pacheco	0.08	0.40	0.55	7409	4088	2540

BRA	Brazil	9880	Simoes	0.08	0.40	0.55	11193	6175	3838
BRA	Brazil	9881	Simplicio Mendes	0.08	0.40	0.55	9681	5341	3319
BRA	Brazil	9882	Socorro Do Piaui	0.08	0.40	0.55	2880	1589	988
BRA	Brazil	9883	Sussuapara	0.19	0.55	0.72	4325	1808	546
BRA	Brazil	9884	Tamboril Do Piaui	0.17	0.49	0.65	2190	1070	523
BRA	Brazil	9885	Tanque Do Piaui	0.08	0.40	0.55	2108	1163	723
BRA	Brazil	9886	Teresina	0.17	0.49	0.65	568395	277740	135605
BRA	Brazil	9887	Uniao	0.31	0.69	0.92	21744	4898	0
BRA	Brazil	9888	Urucui	0.17	0.49	0.65	13814	6750	3296
BRA	Brazil	9889	Valenca Do Piaui	0.23	0.63	0.87	12079	3573	0
BRA	Brazil	9890	Varzea Branca	0.17	0.49	0.65	3597	1758	858
BRA	Brazil	9891	Varzea Grande	0.08	0.40	0.55	3276	1807	1123
BRA	Brazil	9892	Vera Mendes	0.08	0.40	0.55	2130	1175	730
BRA	Brazil	9893	Vila Nova Do Piaui	0.08	0.40	0.55	2160	1192	741
BRA	Brazil	9894	Wall Ferraz	0.08	0.40	0.55	3196	1763	1096
BRA	Brazil	9895	Angra Dos Reis	0.08	0.40	0.55	144864	79925	49669
BRA	Brazil	9896	Aperibe	0.08	0.40	0.55	8491	4685	2911
BRA	Brazil	9897	Araruama	0.08	0.40	0.55	97214	53636	33332
BRA	Brazil	9898	Areal	0.17	0.49	0.65	7659	3742	1827
BRA	Brazil	9899	Armacao De Buzios	0.08	0.40	0.55	23943	13210	8209
BRA	Brazil	9900	Arraial Do Cabo	0.08	0.40	0.55	21108	11646	7237
BRA	Brazil	9901	Barra Do Pirai	0.08	0.40	0.55	73332	40459	25143
BRA	Brazil	9902	Barra Mansa	0.08	0.40	0.55	137878	76071	47274
BRA	Brazil	9903	Belford Roxo	0.08	0.40	0.55	381794	210646	130905
BRA	Brazil	9904	Bom Jardim	0.08	0.40	0.55	20027	11050	6867
BRA	Brazil	9905	Bom Jesus Do Itabapoana	0.08	0.40	0.55	26455	14596	9071
BRA	Brazil	9906	Cabo Frio	0.08	0.40	0.55	165584	91357	56773
BRA	Brazil	9907	Cachoeiras De Macacu	0.08	0.40	0.55	43247	23861	14828
BRA	Brazil	9908	Cambuci	0.08	0.40	0.55	10891	6009	3734
BRA	Brazil	9909	Campos Dos Goytacazes	0.08	0.40	0.55	368567	203348	126369
BRA	Brazil	9910	Cantagalo	0.17	0.49	0.65	11381	5561	2715
BRA	Brazil	9911	Carapebus	0.08	0.40	0.55	12306	6789	4219
BRA	Brazil	9912	Cardoso Moreira	0.08	0.40	0.55	9423	5199	3231

BRA	Brazil	9913	Carmo	0.17	0.49	0.65	11979	5854	2858
BRA	Brazil	9914	Casimiro De Abreu	0.08	0.40	0.55	36257	20004	12431
BRA	Brazil	9915	Comendador Levy Gasparian	0.17	0.49	0.65	4734	2313	1129
BRA	Brazil	9916	Conceicao De Macabu	0.08	0.40	0.55	16667	9196	5715
BRA	Brazil	9917	Cordeiro	0.08	0.40	0.55	16266	8975	5577
BRA	Brazil	9918	Duas Barras	0.17	0.49	0.65	7209	3523	1720
BRA	Brazil	9919	Duque De Caxias	0.08	0.40	0.55	663524	366083	227500
BRA	Brazil	9920	Engenheiro Paulo De Frontin	0.08	0.40	0.55	10971	6053	3761
BRA	Brazil	9921	Guapimirim	0.08	0.40	0.55	46030	25396	15782
BRA	Brazil	9922	Iguaba Grande	0.08	0.40	0.55	18900	10427	6480
BRA	Brazil	9923	Itaborai	0.08	0.40	0.55	174604	96334	59866
BRA	Brazil	9924	Itaguai	0.08	0.40	0.55	96652	53325	33139
BRA	Brazil	9925	Italva	0.08	0.40	0.55	11184	6170	3835
BRA	Brazil	9926	Itaocara	0.08	0.40	0.55	16899	9323	5794
BRA	Brazil	9927	Itaperuna	0.17	0.49	0.65	65761	32133	15689
BRA	Brazil	9928	Itatiaia	0.17	0.49	0.65	20321	9930	4848
BRA	Brazil	9929	Japeri	0.08	0.40	0.55	69873	38551	23957
BRA	Brazil	9930	Laje Do Muriae	0.17	0.49	0.65	4593	2244	1096
BRA	Brazil	9931	Macae	0.08	0.40	0.55	190876	105311	65445
BRA	Brazil	9932	Macuco	0.08	0.40	0.55	5470	3018	1876
BRA	Brazil	9933	Mage	0.08	0.40	0.55	178343	98396	61148
BRA	Brazil	9934	Mangaratiba	0.08	0.40	0.55	28602	15780	9807
BRA	Brazil	9935	Marica	0.08	0.40	0.55	121946	67281	41811
BRA	Brazil	9936	Mendes	0.08	0.40	0.55	12295	6783	4215
BRA	Brazil	9937	Miguel Pereira	0.08	0.40	0.55	18085	9978	6201
BRA	Brazil	9938	Miracema	0.17	0.49	0.65	17377	8491	4146
BRA	Brazil	9939	Natividade	0.31	0.73	0.96	7701	1145	0
BRA	Brazil	9940	Nilopolis	0.08	0.40	0.55	120993	66755	41485
BRA	Brazil	9941	Niteroi	0.08	0.40	0.55	364993	201376	125144
BRA	Brazil	9942	Nova Friburgo	0.08	0.40	0.55	138686	76516	47551
BRA	Brazil	9943	Nova Iguacu	0.08	0.40	0.55	729335	402392	250065
BRA	Brazil	9944	Paracambi	0.08	0.40	0.55	38453	21215	13184
BRA	Brazil	9945	Paraiba Do Sul	0.17	0.49	0.65	28148	13754	6715

BRA	Brazil	9946	Parati	0.08	0.40	0.55	33060	18240	11335
BRA	Brazil	9947	Paty Do Alferes	0.08	0.40	0.55	20681	11410	7091
BRA	Brazil	9948	Petropolis	0.17	0.49	0.65	194474	95028	46397
BRA	Brazil	9949	Pinheiral	0.08	0.40	0.55	17009	9384	5832
BRA	Brazil	9950	Pirai	0.08	0.40	0.55	21997	12136	7542
BRA	Brazil	9951	Porciuncula	0.17	0.49	0.65	12239	5981	2920
BRA	Brazil	9952	Porto Real	0.08	0.40	0.55	13841	7636	4746
BRA	Brazil	9953	Quatis	0.17	0.49	0.65	9446	4616	2254
BRA	Brazil	9954	Queimados	0.08	0.40	0.55	112140	61871	38449
BRA	Brazil	9955	Quissama	0.08	0.40	0.55	18113	9993	6210
BRA	Brazil	9956	Resende	0.17	0.49	0.65	83089	40600	19823
BRA	Brazil	9957	Rio Bonito	0.08	0.40	0.55	44418	24507	15230
BRA	Brazil	9958	Rio Claro	0.08	0.40	0.55	13641	7526	4677
BRA	Brazil	9959	Rio Das Flores	0.17	0.49	0.65	5676	2774	1354
BRA	Brazil	9960	Rio Das Ostras	0.08	0.40	0.55	130039	71746	44586
BRA	Brazil	9961	Rio De Janeiro	0.08	0.40	0.55	4829400	2661302	1650584
BRA	Brazil	9962	Santa Maria Madalena	0.08	0.40	0.55	7670	4231	2630
BRA	Brazil	9963	Santo Antonio De Padua	0.17	0.49	0.65	27198	13290	6489
BRA	Brazil	9964	Sao Fidelis	0.08	0.40	0.55	28695	15832	9839
BRA	Brazil	9965	Sao Francisco De Itabapoana	0.08	0.40	0.55	30481	16817	10451
BRA	Brazil	9966	Sao Goncalo	0.08	0.40	0.55	783134	432075	268511
BRA	Brazil	9967	Sao Joao Da Barra	0.08	0.40	0.55	26115	14408	8954
BRA	Brazil	9968	Sao Joao De Meriti	0.08	0.40	0.55	334813	184725	114796
BRA	Brazil	9969	Sao Jose De Uba	0.08	0.40	0.55	5370	2963	1841
BRA	Brazil	9970	Sao Jose Do Vale Do Rio Preto	0.17	0.49	0.65	13557	6625	3234
BRA	Brazil	9971	Sao Pedro Da Aldeia	0.08	0.40	0.55	79328	43767	27199
BRA	Brazil	9972	Sao Sebastiao Do Alto	0.08	0.40	0.55	6641	3664	2277
BRA	Brazil	9973	Sapucaia	0.17	0.49	0.65	10889	5321	2598
BRA	Brazil	9974	Saquarema	0.08	0.40	0.55	65454	36113	22442
BRA	Brazil	9975	Seropedica	0.08	0.40	0.55	61937	34172	21236
BRA	Brazil	9976	Silva Jardim	0.08	0.40	0.55	15946	8798	5467
BRA	Brazil	9977	Sumidouro	0.17	0.49	0.65	10125	4947	2416
BRA	Brazil	9978	Tangua	0.08	0.40	0.55	24187	13345	8293

BRA	Brazil	9979	Teresopolis	0.08	0.40	0.55	132560	73137	45451
BRA	Brazil	9980	Trajano De Morais	0.08	0.40	0.55	7809	4308	2677
BRA	Brazil	9981	Tres Rios	0.17	0.49	0.65	52353	25582	12490
BRA	Brazil	9982	Valenca	0.17	0.49	0.65	49217	24049	11742
BRA	Brazil	9983	Varre-sai	0.17	0.49	0.65	6706	3277	1600
BRA	Brazil	9984	Vassouras	0.08	0.40	0.55	27183	14998	9320
BRA	Brazil	9985	Volta Redonda	0.08	0.43	0.61	196237	100021	50504
BRA	Brazil	9986	Acari	0.08	0.40	0.55	8200	4524	2811
BRA	Brazil	9987	Acu	0.08	0.40	0.55	41720	23018	14304
BRA	Brazil	9988	Afonso Bezerra	0.08	0.40	0.55	7781	4293	2668
BRA	Brazil	9989	Agua Nova	0.08	0.40	0.55	2225	1227	763
BRA	Brazil	9990	Alexandria	0.08	0.40	0.55	10365	5719	3554
BRA	Brazil	9991	Almino Afonso	0.08	0.40	0.55	4045	2232	1387
BRA	Brazil	9992	Alto Do Rodrigues	0.08	0.40	0.55	10578	5836	3627
BRA	Brazil	9993	Angicos	0.08	0.40	0.55	9333	5149	3200
BRA	Brazil	9994	Antonio Martins	0.08	0.40	0.55	5191	2864	1780
BRA	Brazil	9995	Apodi	0.08	0.40	0.55	26387	14558	9047
BRA	Brazil	9996	Areia Branca	0.08	0.40	0.55	18483	10198	6337
BRA	Brazil	9997	Ares	0.08	0.40	0.55	9726	5366	3335
BRA	Brazil	9998	Augusto Severo	0.08	0.40	0.55	6914	3815	2371
BRA	Brazil	9999	Baia Formosa	0.08	0.40	0.55	6885	3799	2361
BRA	Brazil	10000	Barauna	0.08	0.40	0.55	20647	11391	7079
BRA	Brazil	10001	Barcelona	0.08	0.40	0.55	2847	1571	976
BRA	Brazil	10002	Bento Fernandes	0.08	0.40	0.55	4477	2470	1535
BRA	Brazil	10003	Bodo	0.08	0.40	0.55	1728	953	592
BRA	Brazil	10004	Bom Jesus	0.08	0.40	0.55	7435	4102	2549
BRA	Brazil	10005	Brejinho	0.08	0.40	0.55	5575	3076	1912
BRA	Brazil	10006	Caicara Do Norte	0.08	0.40	0.55	2364	1304	810
BRA	Brazil	10007	Caicara Do Rio Do Vento	0.08	0.40	0.55	2814	1552	965
BRA	Brazil	10008	Caico	0.08	0.40	0.55	48954	27009	16785
BRA	Brazil	10009	Campo Redondo	0.08	0.40	0.55	8223	4537	2819
BRA	Brazil	10010	Canguaretama	0.08	0.40	0.55	24280	13396	8325
BRA	Brazil	10011	Caraubas	0.08	0.40	0.55	14905	8223	5110

BRA	Brazil	10012	Carnauba Dos Dantas	0.08	0.40	0.55	5879	3243	2016
BRA	Brazil	10013	Carnaubais	0.08	0.40	0.55	8597	4743	2948
BRA	Brazil	10014	Ceara-mirim	0.08	0.40	0.55	53882	29728	18474
BRA	Brazil	10015	Cerro Cora	0.08	0.40	0.55	8213	4531	2816
BRA	Brazil	10016	Coronel Ezequiel	0.08	0.40	0.55	4244	2342	1455
BRA	Brazil	10017	Coronel Joao Pessoa	0.08	0.40	0.55	3581	1976	1228
BRA	Brazil	10018	Cruzeta	0.08	0.40	0.55	5814	3208	1993
BRA	Brazil	10019	Currais Novos	0.08	0.40	0.55	33085	18254	11344
BRA	Brazil	10020	Doutor Severiano	0.08	0.40	0.55	5071	2798	1739
BRA	Brazil	10021	Encanto	0.08	0.40	0.55	3996	2205	1370
BRA	Brazil	10022	Equador	0.08	0.40	0.55	5014	2767	1719
BRA	Brazil	10023	Espirito Santo	0.08	0.40	0.55	8974	4951	3077
BRA	Brazil	10024	Extremoz	0.08	0.40	0.55	21550	11890	7389
BRA	Brazil	10025	Felipe Guerra	0.08	0.40	0.55	4484	2474	1537
BRA	Brazil	10026	Fernando Pedroza	0.08	0.40	0.55	1833	1011	629
BRA	Brazil	10027	Florania	0.08	0.40	0.55	7151	3945	2452
BRA	Brazil	10028	Francisco Dantas	0.08	0.40	0.55	2535	1399	869
BRA	Brazil	10029	Frutuoso Gomes	0.08	0.40	0.55	3130	1727	1073
BRA	Brazil	10030	Galinhos	0.08	0.40	0.55	1880	1037	645
BRA	Brazil	10031	Goianinha	0.08	0.40	0.55	18195	10039	6239
BRA	Brazil	10032	Governador Dix-sept Rosado	0.08	0.40	0.55	9827	5422	3369
BRA	Brazil	10033	Grossos	0.08	0.40	0.55	8527	4704	2924
BRA	Brazil	10034	Guamare	0.08	0.40	0.55	10763	5938	3690
BRA	Brazil	10035	Ielmo Marinho	0.08	0.40	0.55	9919	5473	3401
BRA	Brazil	10036	Ipanguacu	0.08	0.40	0.55	10883	6005	3732
BRA	Brazil	10037	Ipueira	0.08	0.40	0.55	1737	958	596
BRA	Brazil	10038	Itaja	0.08	0.40	0.55	5442	3003	1866
BRA	Brazil	10039	Itau	0.08	0.40	0.55	4110	2267	1409
BRA	Brazil	10040	Jacana	0.08	0.40	0.55	4004	2209	1373
BRA	Brazil	10041	Jandaira	0.08	0.40	0.55	4968	2741	1703
BRA	Brazil	10042	Janduis	0.08	0.40	0.55	4004	2209	1373
BRA	Brazil	10043	Januario Cicco	0.08	0.40	0.55	7030	3878	2410
BRA	Brazil	10044	Japi	0.08	0.40	0.55	3912	2159	1341

BRA	Brazil	10045	Jardim De Angicos	0.08	0.40	0.55	1831	1010	628
BRA	Brazil	10046	Jardim De Piranhas	0.08	0.40	0.55	11420	6301	3916
BRA	Brazil	10047	Jardim Do Serido	0.08	0.40	0.55	9097	5019	3119
BRA	Brazil	10048	Joao Camara	0.08	0.40	0.55	25966	14326	8903
BRA	Brazil	10049	Joao Dias	0.08	0.40	0.55	1825	1007	626
BRA	Brazil	10050	Jose Da Penha	0.08	0.40	0.55	3777	2084	1295
BRA	Brazil	10051	Jucurutu	0.08	0.40	0.55	13289	7332	4556
BRA	Brazil	10052	Lagoa D'anta	0.08	0.40	0.55	4772	2633	1636
BRA	Brazil	10053	Lagoa De Pedras	0.08	0.40	0.55	5545	3060	1901
BRA	Brazil	10054	Lagoa De Velhos	0.08	0.40	0.55	2087	1152	716
BRA	Brazil	10055	Lagoa Nova	0.08	0.40	0.55	10382	5728	3560
BRA	Brazil	10056	Lagoa Salgada	0.08	0.40	0.55	4629	2554	1587
BRA	Brazil	10057	Lajes	0.08	0.40	0.55	8090	4463	2774
BRA	Brazil	10058	Lajes Pintadas	0.08	0.40	0.55	3258	1798	1117
BRA	Brazil	10059	Lucrecia	0.08	0.40	0.55	2252	1242	772
BRA	Brazil	10060	Luis Gomes	0.08	0.40	0.55	6683	3687	2291
BRA	Brazil	10061	Macaiba	0.08	0.40	0.55	54755	30210	18774
BRA	Brazil	10062	Macau	0.08	0.40	0.55	22902	12636	7852
BRA	Brazil	10063	Major Sales	0.08	0.40	0.55	3065	1691	1051
BRA	Brazil	10064	Marcelino Vieira	0.08	0.40	0.55	6319	3487	2167
BRA	Brazil	10065	Martins	0.08	0.40	0.55	6487	3579	2224
BRA	Brazil	10066	Maxaranguape	0.08	0.40	0.55	8296	4577	2844
BRA	Brazil	10067	Messias Targino	0.08	0.40	0.55	3352	1849	1149
BRA	Brazil	10068	Montanhas	0.08	0.40	0.55	7743	4272	2655
BRA	Brazil	10069	Monte Alegre	0.08	0.40	0.55	19648	10840	6737
BRA	Brazil	10070	Monte Das Gameleiras	0.08	0.40	0.55	1746	964	599
BRA	Brazil	10071	Mossoro	0.08	0.40	0.55	213014	117525	73036
BRA	Brazil	10072	Natal	0.08	0.40	0.55	645485	356130	221316
BRA	Brazil	10073	Nisia Floresta	0.08	0.40	0.55	25089	13842	8602
BRA	Brazil	10074	Nova Cruz	0.08	0.40	0.55	23718	13086	8132
BRA	Brazil	10075	Olho-d'agua Do Borges	0.08	0.40	0.55	3178	1753	1090
BRA	Brazil	10076	Ouro Branco	0.08	0.40	0.55	3361	1854	1152
BRA	Brazil	10077	Parana	0.60	0.90	0.97	784	0	0

BRA	Brazil	10078	Parau	0.08	0.40	0.55	3048	1681	1045
BRA	Brazil	10079	Parazinho	0.08	0.40	0.55	2985	1647	1023
BRA	Brazil	10080	Parelhas	0.08	0.40	0.55	15596	8605	5347
BRA	Brazil	10081	Parnamirim	0.08	0.40	0.55	176278	97257	60440
BRA	Brazil	10082	Passa E Fica	0.08	0.40	0.55	9125	5034	3129
BRA	Brazil	10083	Passagem	0.08	0.40	0.55	2607	1439	894
BRA	Brazil	10084	Patu	0.08	0.40	0.55	9199	5075	3154
BRA	Brazil	10085	Pau Dos Ferros	0.08	0.40	0.55	22017	12147	7549
BRA	Brazil	10086	Pedra Grande	0.08	0.40	0.55	2135	1178	732
BRA	Brazil	10087	Pedra Preta	0.08	0.40	0.55	1808	998	620
BRA	Brazil	10088	Pedro Avelino	0.08	0.40	0.55	5536	3054	1898
BRA	Brazil	10089	Pedro Velho	0.08	0.40	0.55	11282	6224	3868
BRA	Brazil	10090	Pendencias	0.08	0.40	0.55	10309	5688	3535
BRA	Brazil	10091	Piloes	0.08	0.40	0.55	2630	1451	902
BRA	Brazil	10092	Poco Branco	0.08	0.40	0.55	10037	5538	3441
BRA	Brazil	10093	Portalegre	0.08	0.40	0.55	5367	2961	1840
BRA	Brazil	10094	Porto Do Mangue	0.08	0.40	0.55	4850	2676	1663
BRA	Brazil	10095	Presidente Juscelino	0.08	0.40	0.55	7392	4079	2535
BRA	Brazil	10096	Pureza	0.08	0.40	0.55	6900	3807	2366
BRA	Brazil	10097	Rafael Fernandes	0.08	0.40	0.55	3720	2052	1275
BRA	Brazil	10098	Rafael Godeiro	0.08	0.40	0.55	2295	1266	787
BRA	Brazil	10099	Riacho Da Cruz	0.08	0.40	0.55	2543	1403	872
BRA	Brazil	10100	Riacho De Santana	0.08	0.40	0.55	3115	1719	1068
BRA	Brazil	10101	Riachuelo	0.08	0.40	0.55	5851	3228	2006
BRA	Brazil	10102	Rio Do Fogo	0.08	0.40	0.55	6438	3552	2207
BRA	Brazil	10103	Rodolfo Fernandes	0.08	0.40	0.55	3237	1786	1110
BRA	Brazil	10104	Ruy Barbosa	0.08	0.40	0.55	2612	1441	896
BRA	Brazil	10105	Santa Cruz	0.08	0.40	0.55	28436	15689	9750
BRA	Brazil	10106	Santa Maria	1.00	1.00	1.00	0	0	0
BRA	Brazil	10107	Santana Do Matos	0.08	0.40	0.55	9601	5297	3292
BRA	Brazil	10108	Santana Do Serido	0.08	0.40	0.55	1894	1045	649
BRA	Brazil	10109	Santo Antonio	0.08	0.40	0.55	16857	9300	5780
BRA	Brazil	10110	Sao Bento Do Norte	0.08	0.40	0.55	3739	2063	1282

BRA	Brazil	10111	Sao Bento Do Trairi	0.08	0.40	0.55	2995	1653	1027
BRA	Brazil	10112	Sao Fernando	0.08	0.40	0.55	2702	1490	926
BRA	Brazil	10113	Sao Francisco Do Oeste	0.08	0.40	0.55	2815	1553	965
BRA	Brazil	10114	Sao Goncalo Do Amarante	0.08	0.40	0.55	79559	43895	27278
BRA	Brazil	10115	Sao Joao Do Sabugi	0.08	0.40	0.55	4514	2490	1548
BRA	Brazil	10116	Sao Jose De Mipibu	0.08	0.40	0.55	29776	16428	10209
BRA	Brazil	10117	Sao Jose Do Campestre	0.08	0.40	0.55	9462	5221	3244
BRA	Brazil	10118	Sao Jose Do Serido	0.08	0.40	0.55	3390	1870	1162
BRA	Brazil	10119	Sao Miguel	0.08	0.40	0.55	17172	9474	5888
BRA	Brazil	10120	Sao Miguel De Touros	0.08	0.40	0.55	5503	3036	1887
BRA	Brazil	10121	Sao Paulo Do Potengi	0.08	0.40	0.55	12262	6765	4204
BRA	Brazil	10122	Sao Pedro	0.53	0.77	0.88	1631	172	0
BRA	Brazil	10123	Sao Rafael	0.08	0.40	0.55	5754	3175	1973
BRA	Brazil	10124	Sao Tome	0.08	0.40	0.55	8210	4530	2815
BRA	Brazil	10125	Sao Vicente	0.08	0.40	0.55	5269	2907	1806
BRA	Brazil	10126	Senador Eloi De Souza	0.08	0.40	0.55	4467	2465	1532
BRA	Brazil	10127	Senador Georgino Avelino	0.08	0.40	0.55	3527	1946	1209
BRA	Brazil	10128	Serra De Sao Bento	0.08	0.40	0.55	4185	2309	1435
BRA	Brazil	10129	Serra Do Mel	0.08	0.40	0.55	8412	4641	2884
BRA	Brazil	10130	Serra Negra Do Norte	0.08	0.40	0.55	5852	3229	2007
BRA	Brazil	10131	Serrinha	0.08	0.40	0.55	4707	2597	1614
BRA	Brazil	10132	Serrinha Dos Pintos	0.08	0.40	0.55	3355	1851	1150
BRA	Brazil	10133	Severiano Melo	0.08	0.40	0.55	3087	1703	1059
BRA	Brazil	10134	Sitio Novo	0.08	0.40	0.55	3895	2149	1336
BRA	Brazil	10135	Taboleiro Grande	0.08	0.40	0.55	1810	998	620
BRA	Brazil	10136	Taipu	0.08	0.40	0.55	8795	4853	3016
BRA	Brazil	10137	Tangara	0.24	0.62	0.83	9075	2990	0
BRA	Brazil	10138	Tenente Ananias	0.08	0.40	0.55	7786	4296	2669
BRA	Brazil	10139	Tenente Laurentino Cruz	0.08	0.40	0.55	3724	2055	1277
BRA	Brazil	10140	Tibau	0.08	0.40	0.55	3027	1670	1038
BRA	Brazil	10141	Tibau Do Sul	0.08	0.40	0.55	9933	5480	3406
BRA	Brazil	10142	Timbauba Dos Batistas	0.08	0.40	0.55	1783	984	611
BRA	Brazil	10143	Touros	0.08	0.40	0.55	27189	15001	9322

BRA	Brazil	10144	Triunfo Potiguar	0.08	0.40	0.55	2416	1333	828
BRA	Brazil	10145	Umarizal	0.08	0.40	0.55	7884	4350	2703
BRA	Brazil	10146	Upanema	0.08	0.40	0.55	10170	5611	3487
BRA	Brazil	10147	Varzea	0.08	0.40	0.55	9478	5229	3250
BRA	Brazil	10148	Venha-ver	0.08	0.40	0.55	3124	1724	1071
BRA	Brazil	10149	Vera Cruz	0.08	0.40	0.55	4970	2742	1704
BRA	Brazil	10150	Vicosa	0.08	0.40	0.55	1342	740	460
BRA	Brazil	10151	Vila Flor	0.08	0.40	0.55	2399	1324	823
BRA	Brazil	10152	Agua Santa	0.50	0.75	0.92	1391	231	0
BRA	Brazil	10153	Agudo	0.68	0.83	0.95	1962	0	0
BRA	Brazil	10154	Ajuricaba	0.62	0.87	0.96	1308	0	0
BRA	Brazil	10155	Alecrim	0.63	0.83	0.96	1158	0	0
BRA	Brazil	10156	Alegrete	0.67	0.85	0.98	10223	0	0
BRA	Brazil	10157	Alegria	0.68	0.83	0.93	464	0	0
BRA	Brazil	10158	Alpestre	0.44	0.79	0.95	2696	40	0
BRA	Brazil	10159	Alto Alegre	1.00	1.00	1.00	0	0	0
BRA	Brazil	10160	Alto Feliz	0.64	0.80	0.93	570	17	0
BRA	Brazil	10161	Alvorada	0.29	0.65	0.92	103399	30770	0
BRA	Brazil	10162	Amaral Ferrador	0.17	0.51	0.69	4273	1977	771
BRA	Brazil	10163	Ametista Do Sul	0.59	0.83	0.99	1443	0	0
BRA	Brazil	10164	Andre Da Rocha	0.79	0.89	0.97	9	0	0
BRA	Brazil	10165	Anta Gorda	0.44	0.69	0.90	2209	640	0
BRA	Brazil	10166	Antonio Prado	0.87	0.92	0.96	0	0	0
BRA	Brazil	10167	Arambare	0.17	0.51	0.69	2379	1084	400
BRA	Brazil	10168	Ararica	0.32	0.66	0.93	3075	871	0
BRA	Brazil	10169	Aratiba	0.66	0.80	0.90	878	2	0
BRA	Brazil	10170	Arroio Do Meio	0.17	0.49	0.65	12848	6278	3065
BRA	Brazil	10171	Arroio Do Sal	0.08	0.40	0.55	7372	4067	2528
BRA	Brazil	10172	Arroio Do Tigre	0.17	0.49	0.65	8273	4042	1974
BRA	Brazil	10173	Arroio Dos Ratos	0.59	0.80	0.98	2927	15	0
BRA	Brazil	10174	Arroio Grande	0.08	0.43	0.61	13509	6911	3516
BRA	Brazil	10175	Arvorezinha	0.77	0.87	0.94	285	0	0
BRA	Brazil	10176	Augusto Pestana	0.71	0.85	0.94	704	0	0

BRA	Brazil	10177	Aurea	0.40	0.69	0.89	1452	394	0
BRA	Brazil	10178	Bage	0.17	0.50	0.66	79172	37799	17247
BRA	Brazil	10179	Balneario Pinhal	0.08	0.41	0.58	9488	5073	2929
BRA	Brazil	10180	Barao	0.22	0.60	0.84	3460	1190	0
BRA	Brazil	10181	Barao De Cotegipe	0.13	0.53	0.76	4941	1967	291
BRA	Brazil	10182	Barao Do Triunfo	0.66	0.82	0.95	1054	0	0
BRA	Brazil	10183	Barra Do Guarita	0.32	0.74	0.98	1568	200	0
BRA	Brazil	10184	Barra Do Quarai	0.26	0.62	0.86	2284	772	0
BRA	Brazil	10185	Barra Do Ribeiro	0.68	0.84	0.97	1601	0	0
BRA	Brazil	10186	Barra Do Rio Azul	0.28	0.66	0.88	966	254	0
BRA	Brazil	10187	Barra Funda	0.25	0.63	0.85	1404	427	0
BRA	Brazil	10188	Barracao	0.17	0.49	0.65	3462	1692	826
BRA	Brazil	10189	Barros Cassal	0.87	0.91	0.94	0	0	0
BRA	Brazil	10190	Benjamin Constant Do Sul	0.32	0.76	0.96	1165	107	0
BRA	Brazil	10191	Bento Goncalves	0.32	0.68	0.93	57206	14565	0
BRA	Brazil	10192	Boa Vista Das Missoes	0.17	0.60	0.86	1315	419	0
BRA	Brazil	10193	Boa Vista Do Burica	0.67	0.86	0.96	878	0	0
BRA	Brazil	10194	Boa Vista Do Sul	0.83	0.88	0.92	0	0	0
BRA	Brazil	10195	Bom Jesus	0.17	0.49	0.65	7409	3620	1768
BRA	Brazil	10196	Bom Principio	0.50	0.75	0.94	4081	700	0
BRA	Brazil	10197	Bom Progresso	0.46	0.80	0.97	727	0	0
BRA	Brazil	10198	Bom Retiro Do Sul	0.44	0.71	0.92	4104	1077	0
BRA	Brazil	10199	Boqueirao Do Leao	0.84	0.92	0.98	0	0	0
BRA	Brazil	10200	Bossoroca	0.50	0.79	0.96	2023	87	0
BRA	Brazil	10201	Braga	0.45	0.79	0.98	1259	44	0
BRA	Brazil	10202	Brochier	0.31	0.64	0.89	2503	808	0
BRA	Brazil	10203	Butia	0.39	0.71	0.96	8598	1940	0
BRA	Brazil	10204	Cacapava Do Sul	0.62	0.80	0.95	6169	0	0
BRA	Brazil	10205	Cacequi	0.59	0.79	0.96	2831	122	0
BRA	Brazil	10206	Cachoeira Do Sul	0.77	0.88	0.96	2387	0	0
BRA	Brazil	10207	Cachoeirinha	0.08	0.40	0.55	90815	50105	31137
BRA	Brazil	10208	Cacique Doble	0.64	0.84	0.96	802	0	0
BRA	Brazil	10209	Caibate	0.87	0.93	0.96	0	0	0

BRA	Brazil	10210	Caicara	0.17	0.49	0.65	3151	1540	752
BRA	Brazil	10211	Camaqua	0.19	0.55	0.75	39957	16179	3147
BRA	Brazil	10212	Camargo	0.79	0.88	0.96	24	0	0
BRA	Brazil	10213	Cambara Do Sul	0.43	0.68	0.88	2425	778	0
BRA	Brazil	10214	Campestre Da Serra	0.90	0.93	0.96	0	0	0
BRA	Brazil	10215	Campina Das Missoes	0.56	0.79	0.94	1446	46	0
BRA	Brazil	10216	Campinas Do Sul	0.30	0.64	0.87	3823	1192	0
BRA	Brazil	10217	Campo Bom	0.37	0.69	0.92	29358	7714	0
BRA	Brazil	10218	Campo Novo	0.48	0.81	0.97	1658	0	0
BRA	Brazil	10219	Campos Borges	0.94	0.95	0.96	0	0	0
BRA	Brazil	10220	Candelaria	0.68	0.83	0.96	3866	0	0
BRA	Brazil	10221	Candido Godoi	0.17	0.49	0.65	4080	1994	973
BRA	Brazil	10222	Candiota	0.24	0.62	0.86	4278	1358	0
BRA	Brazil	10223	Canela	0.64	0.83	0.99	7249	0	0
BRA	Brazil	10224	Cangucu	0.22	0.60	0.81	32673	11436	0
BRA	Brazil	10225	Canoas	0.43	0.73	0.96	129945	24049	0
BRA	Brazil	10226	Capao Da Canoa	0.08	0.43	0.61	35295	18041	9129
BRA	Brazil	10227	Capao Do Leao	0.08	0.41	0.56	18508	10119	6142
BRA	Brazil	10228	Capela De Santana	0.18	0.52	0.69	8601	3906	1524
BRA	Brazil	10229	Capitao	0.45	0.71	0.92	948	254	0
BRA	Brazil	10230	Capivari Do Sul	0.34	0.70	0.93	2132	471	0
BRA	Brazil	10231	Caraa	0.10	0.45	0.63	5612	2794	1333
BRA	Brazil	10232	Carazinho	0.13	0.55	0.79	42813	16273	760
BRA	Brazil	10233	Carlos Barbosa	0.41	0.70	0.92	10905	2857	0
BRA	Brazil	10234	Carlos Gomes	0.53	0.77	0.93	407	44	0
BRA	Brazil	10235	Casca	0.82	0.87	0.92	0	0	0
BRA	Brazil	10236	Caseiros	0.88	0.93	0.97	0	0	0
BRA	Brazil	10237	Catuipe	0.59	0.86	0.96	1957	0	0
BRA	Brazil	10238	Caxias Do Sul	0.52	0.77	0.97	137358	13296	0
BRA	Brazil	10239	Centenario	0.35	0.81	0.98	1338	0	0
BRA	Brazil	10240	Cerrito	0.08	0.40	0.55	4551	2511	1561
BRA	Brazil	10241	Cerro Branco	0.69	0.83	0.95	520	0	0
BRA	Brazil	10242	Cerro Grande	0.20	0.65	0.89	1470	368	0

BRA	Brazil	10243	Cerro Grande Do Sul	0.26	0.65	0.92	6288	1728	0
BRA	Brazil	10244	Cerro Largo	0.58	0.85	0.96	3041	0	0
BRA	Brazil	10245	Chapada	0.23	0.63	0.86	5405	1600	0
BRA	Brazil	10246	Charqueadas	0.64	0.81	0.96	6138	0	0
BRA	Brazil	10247	Charrua	0.43	0.81	0.97	1270	0	0
BRA	Brazil	10248	Chiapeta	0.17	0.49	0.65	2509	1226	599
BRA	Brazil	10249	Chui	0.08	0.48	0.73	6896	3028	706
BRA	Brazil	10250	Chuvisca	0.17	0.49	0.65	3246	1586	774
BRA	Brazil	10251	Cidreira	0.09	0.43	0.60	10547	5494	2965
BRA	Brazil	10252	Ciriaco	0.64	0.81	0.95	795	0	0
BRA	Brazil	10253	Colinas	0.17	0.49	0.65	1789	874	427
BRA	Brazil	10254	Colorado	0.08	0.40	0.55	2465	1360	845
BRA	Brazil	10255	Condor	0.49	0.79	0.98	2111	52	0
BRA	Brazil	10256	Constantina	0.21	0.67	0.90	6957	1578	0
BRA	Brazil	10257	Coqueiros Do Sul	0.44	0.67	0.84	788	292	0
BRA	Brazil	10258	Coronel Barros	0.91	0.93	0.95	0	0	0
BRA	Brazil	10259	Coronel Bicaco	0.25	0.65	0.92	4208	1130	0
BRA	Brazil	10260	Cotipora	0.75	0.84	0.91	224	0	0
BRA	Brazil	10261	Coxilha	0.13	0.50	0.70	1942	863	303
BRA	Brazil	10262	Crissiumal	0.45	0.78	0.97	4923	232	0
BRA	Brazil	10263	Cristal	0.13	0.51	0.71	5234	2288	669
BRA	Brazil	10264	Cristal Do Sul	0.28	0.63	0.87	1557	516	0
BRA	Brazil	10265	Cruz Alta	0.81	0.92	0.98	0	0	0
BRA	Brazil	10266	Cruzeiro Do Sul	0.17	0.49	0.65	8600	4202	2052
BRA	Brazil	10267	David Canabarro	0.25	0.57	0.80	2630	1096	20
BRA	Brazil	10268	Derrubadas	0.48	0.77	0.96	1043	88	0
BRA	Brazil	10269	Dezesseis De Novembro	0.66	0.84	0.94	386	0	0
BRA	Brazil	10270	Dilermando De Aguiar	0.81	0.89	0.96	0	0	0
BRA	Brazil	10271	Dois Irmaos	0.33	0.68	0.91	15211	3933	0
BRA	Brazil	10272	Dois Irmaos Das Missoes	0.36	0.73	0.94	967	149	0
BRA	Brazil	10273	Dois Lajeados	0.22	0.57	0.79	1863	734	36
BRA	Brazil	10274	Dom Feliciano	0.17	0.49	0.65	9595	4688	2289
BRA	Brazil	10275	Dom Pedrito	0.46	0.74	0.97	13550	2320	0

BRA	Brazil	10276	Dom Pedro De Alcantara	0.08	0.43	0.61	1879	972	505
BRA	Brazil	10277	Dona Francisca	0.95	0.96	0.97	0	0	0
BRA	Brazil	10278	Doutor Mauricio Cardoso	0.65	0.82	0.94	740	0	0
BRA	Brazil	10279	Doutor Ricardo	0.36	0.65	0.88	925	309	0
BRA	Brazil	10280	Eldorado Do Sul	0.65	0.82	0.97	5902	0	0
BRA	Brazil	10281	Encantado	0.43	0.71	0.93	8102	1927	0
BRA	Brazil	10282	Encruzilhada Do Sul	0.66	0.81	0.93	3544	0	0
BRA	Brazil	10283	Engenho Velho	0.29	0.78	0.98	729	22	0
BRA	Brazil	10284	Entre Rios Do Sul	0.38	0.67	0.89	1248	382	0
BRA	Brazil	10285	Entre-ijuis	0.35	0.75	0.97	4114	468	0
BRA	Brazil	10286	Erebango	0.35	0.71	0.93	1392	284	0
BRA	Brazil	10287	Erechim	0.15	0.59	0.85	69038	22029	0
BRA	Brazil	10288	Ernestina	0.17	0.49	0.65	2649	1294	632
BRA	Brazil	10289	Erval Grande	0.40	0.70	0.89	2061	510	0
BRA	Brazil	10290	Erval Seco	0.23	0.59	0.81	4341	1628	0
BRA	Brazil	10291	Esmeralda	0.90	0.94	0.97	0	0	0
BRA	Brazil	10292	Esperanca Do Sul	0.37	0.79	0.98	1341	40	0
BRA	Brazil	10293	Espumoso	0.88	0.92	0.96	0	0	0
BRA	Brazil	10294	Estacao	0.24	0.65	0.88	3655	1003	0
BRA	Brazil	10295	Estancia Velha	0.33	0.68	0.94	23673	5766	0
BRA	Brazil	10296	Esteio	0.40	0.71	0.94	30418	6798	0
BRA	Brazil	10297	Estrela	0.60	0.80	0.95	6873	0	0
BRA	Brazil	10298	Estrela Velha	0.94	0.95	0.96	0	0	0
BRA	Brazil	10299	Eugenio De Castro	0.84	0.91	0.96	0	0	0
BRA	Brazil	10300	Fagundes Varela	0.75	0.85	0.95	145	0	0
BRA	Brazil	10301	Farroupilha	0.62	0.81	0.96	13651	0	0
BRA	Brazil	10302	Faxinal Do Soturno	0.91	0.93	0.95	0	0	0
BRA	Brazil	10303	Faxinalzinho	0.17	0.61	0.84	1433	442	0
BRA	Brazil	10304	Fazenda Vilanova	0.39	0.69	0.93	1825	485	0
BRA	Brazil	10305	Feliz	0.55	0.77	0.94	3278	397	0
BRA	Brazil	10306	Flores Da Cunha	0.65	0.82	0.96	4757	0	0
BRA	Brazil	10307	Floriano Peixoto	0.45	0.71	0.91	660	180	0
BRA	Brazil	10308	Fontoura Xavier	0.78	0.88	0.97	210	0	0

BRA	Brazil	10309	Formigueiro	0.69	0.85	0.97	744	0	0
BRA	Brazil	10310	Fortaleza Dos Valos	0.77	0.89	0.96	132	0	0
BRA	Brazil	10311	Frederico Westphalen	0.37	0.77	0.98	13112	936	0
BRA	Brazil	10312	Garibaldi	0.60	0.78	0.93	7295	599	0
BRA	Brazil	10313	Garruchos	0.56	0.82	0.97	785	0	0
BRA	Brazil	10314	Gaurama	0.13	0.54	0.76	3839	1520	209
BRA	Brazil	10315	General Camara	0.62	0.80	0.93	1632	40	0
BRA	Brazil	10316	Gentil	0.72	0.84	0.94	141	0	0
BRA	Brazil	10317	Getulio Vargas	0.20	0.62	0.86	9532	2861	0
BRA	Brazil	10318	Girua	0.56	0.82	0.94	4051	0	0
BRA	Brazil	10319	Glorinha	0.34	0.67	0.91	3557	1017	0
BRA	Brazil	10320	Gramado	0.84	0.90	0.97	0	0	0
BRA	Brazil	10321	Gramado Dos Loureiros	0.58	0.84	0.97	463	0	0
BRA	Brazil	10322	Gramado Xavier	0.81	0.90	0.97	0	0	0
BRA	Brazil	10323	Gravatai	0.26	0.62	0.87	149814	48708	0
BRA	Brazil	10324	Guabiju	0.91	0.93	0.94	0	0	0
BRA	Brazil	10325	Guaiba	0.78	0.88	0.97	2180	0	0
BRA	Brazil	10326	Guapore	0.57	0.78	0.95	5906	404	0
BRA	Brazil	10327	Guarani Das Missoes	0.64	0.87	0.96	1258	0	0
BRA	Brazil	10328	Harmonia	0.25	0.63	0.87	2717	842	0
BRA	Brazil	10329	Herval	0.17	0.50	0.67	5122	2443	1092
BRA	Brazil	10330	Herveiras	0.93	0.96	0.98	0	0	0
BRA	Brazil	10331	Horizontina	0.17	0.49	0.65	12081	5903	2882
BRA	Brazil	10332	Hulha Negra	0.19	0.53	0.72	5124	2213	632
BRA	Brazil	10333	Humaita	0.17	0.49	0.65	3139	1534	749
BRA	Brazil	10334	Ibarama	0.72	0.85	0.97	373	0	0
BRA	Brazil	10335	Ibiaca	0.66	0.80	0.90	754	26	0
BRA	Brazil	10336	Ibiraiaras	0.93	0.95	0.97	0	0	0
BRA	Brazil	10337	Ibirapuita	0.67	0.78	0.88	707	105	0
BRA	Brazil	10338	Ibiruba	0.76	0.88	0.97	842	0	0
BRA	Brazil	10339	Igrejinha	0.29	0.66	0.91	19219	5259	0
BRA	Brazil	10340	Ijui	0.59	0.84	0.97	17427	0	0
BRA	Brazil	10341	Ilopolis	0.17	0.49	0.65	2590	1265	618

BRA	Brazil	10342	Imbe	0.08	0.43	0.61	15875	8173	4229
BRA	Brazil	10343	Imigrante	0.18	0.51	0.67	2657	1244	549
BRA	Brazil	10344	Independencia	0.17	0.49	0.65	4125	2016	984
BRA	Brazil	10345	Inhacora	0.66	0.85	0.97	318	0	0
BRA	Brazil	10346	Ipe	0.92	0.94	0.96	0	0	0
BRA	Brazil	10347	Ipiranga Do Sul	0.50	0.72	0.88	586	156	0
BRA	Brazil	10348	Irai	0.37	0.78	0.99	3534	190	0
BRA	Brazil	10349	Itaara	0.85	0.92	0.97	0	0	0
BRA	Brazil	10350	Itacurubi	0.31	0.72	0.96	1728	268	0
BRA	Brazil	10351	Itapuca	0.85	0.91	0.95	0	0	0
BRA	Brazil	10352	Itaqui	0.58	0.80	0.96	8284	159	0
BRA	Brazil	10353	Itatiba Do Sul	0.36	0.67	0.90	1708	520	0
BRA	Brazil	10354	Ivora	0.91	0.93	0.95	0	0	0
BRA	Brazil	10355	Ivoti	0.56	0.79	0.94	5351	286	0
BRA	Brazil	10356	Jaboticaba	0.50	0.79	0.97	1198	51	0
BRA	Brazil	10357	Jacutinga	0.08	0.40	0.55	3093	1706	1060
BRA	Brazil	10358	Jaguarao	0.08	0.45	0.66	20301	9798	3971
BRA	Brazil	10359	Jaguari	0.30	0.67	0.92	5700	1475	0
BRA	Brazil	10360	Jaquirana	0.87	0.93	0.99	0	0	0
BRA	Brazil	10361	Jari	0.27	0.66	0.91	1984	524	0
BRA	Brazil	10362	Joia	0.77	0.90	0.97	218	0	0
BRA	Brazil	10363	Julio De Castilhos	0.69	0.90	0.98	2213	0	0
BRA	Brazil	10364	Lagoa Dos Tres Cantos	0.58	0.76	0.90	367	70	0
BRA	Brazil	10365	Lagoa Vermelha	0.67	0.83	0.97	3902	0	0
BRA	Brazil	10366	Lagoao	0.89	0.93	0.97	0	0	0
BRA	Brazil	10367	Lajeado	0.54	0.79	0.97	20955	994	0
BRA	Brazil	10368	Lajeado Do Bugre	0.18	0.60	0.86	1651	546	0
BRA	Brazil	10369	Lavras Do Sul	0.60	0.79	0.95	1575	42	0
BRA	Brazil	10370	Liberato Salzano	0.22	0.61	0.85	3247	1067	0
BRA	Brazil	10371	Lindolfo Collor	0.57	0.80	0.97	1311	0	0
BRA	Brazil	10372	Linha Nova	0.60	0.79	0.93	360	26	0
BRA	Brazil	10373	Macambara	0.17	0.49	0.65	2830	1383	675
BRA	Brazil	10374	Machadinho	0.80	0.90	0.98	0	0	0

BRA	Brazil	10375	Mampituba	0.08	0.43	0.61	2231	1142	588
BRA	Brazil	10376	Manoel Viana	0.72	0.86	0.97	591	0	0
BRA	Brazil	10377	Maquine	0.16	0.51	0.73	4468	1997	489
BRA	Brazil	10378	Marata	0.35	0.66	0.89	1314	410	0
BRA	Brazil	10379	Marau	0.17	0.49	0.65	26596	12996	6345
BRA	Brazil	10380	Marcelino Ramos	0.21	0.61	0.86	2785	901	0
BRA	Brazil	10381	Mariana Pimentel	0.74	0.85	0.94	220	0	0
BRA	Brazil	10382	Mariano Moro	0.37	0.65	0.84	949	325	0
BRA	Brazil	10383	Marques De Souza	0.80	0.86	0.91	7	0	0
BRA	Brazil	10384	Mata	0.39	0.73	0.95	2039	331	0
BRA	Brazil	10385	Mato Castelhano	0.28	0.60	0.82	1344	516	0
BRA	Brazil	10386	Mato Leitao	0.74	0.86	0.96	247	0	0
BRA	Brazil	10387	Maximiliano De Almeida	0.33	0.69	0.92	2269	525	0
BRA	Brazil	10388	Minas Do Leao	0.65	0.82	0.97	1210	0	0
BRA	Brazil	10389	Miraguai	0.81	0.92	0.98	0	0	0
BRA	Brazil	10390	Montauri	0.92	0.93	0.94	0	0	0
BRA	Brazil	10391	Monte Alegre Dos Campos	0.82	0.91	0.98	0	0	0
BRA	Brazil	10392	Monte Belo Do Sul	0.64	0.79	0.91	426	28	0
BRA	Brazil	10393	Montenegro	0.48	0.74	0.95	20998	3799	0
BRA	Brazil	10394	Mormaco	0.17	0.49	0.65	1865	911	445
BRA	Brazil	10395	Morrinhos Do Sul	0.08	0.40	0.55	2287	1262	784
BRA	Brazil	10396	Morro Redondo	0.08	0.41	0.58	4721	2528	1460
BRA	Brazil	10397	Morro Reuter	0.17	0.49	0.65	3847	1880	918
BRA	Brazil	10398	Mostardas	0.08	0.43	0.61	9141	4667	2364
BRA	Brazil	10399	Mucum	0.43	0.71	0.94	1446	360	0
BRA	Brazil	10400	Muitos Capoes	0.97	0.98	0.99	0	0	0
BRA	Brazil	10401	Muliterno	0.76	0.88	0.98	70	0	0
BRA	Brazil	10402	Nao-me-toque	0.33	0.67	0.91	8062	2284	0
BRA	Brazil	10403	Nicolau Vergueiro	0.28	0.64	0.90	892	267	0
BRA	Brazil	10404	Nonoai	0.24	0.66	0.90	6834	1651	0
BRA	Brazil	10405	Nova Alvorada	0.92	0.94	0.96	0	0	0
BRA	Brazil	10406	Nova Araca	0.26	0.61	0.80	1570	556	11
BRA	Brazil	10407	Nova Bassano	0.63	0.79	0.94	1966	69	0

BRA	Brazil	10408	Nova Boa Vista	0.24	0.68	0.88	1116	229	0
BRA	Brazil	10409	Nova Brescia	0.40	0.66	0.87	1893	658	0
BRA	Brazil	10410	Nova Candelaria	0.68	0.84	0.95	330	0	0
BRA	Brazil	10411	Nova Esperanca Do Sul	0.31	0.70	0.94	2541	545	0
BRA	Brazil	10412	Nova Hartz	0.26	0.64	0.90	10803	3306	0
BRA	Brazil	10413	Nova Padua	0.76	0.85	0.93	98	0	0
BRA	Brazil	10414	Nova Palma	0.92	0.95	0.98	0	0	0
BRA	Brazil	10415	Nova Petropolis	0.58	0.78	0.94	4601	369	0
BRA	Brazil	10416	Nova Prata	0.72	0.85	0.95	2090	0	0
BRA	Brazil	10417	Nova Ramada	0.54	0.83	0.95	617	0	0
BRA	Brazil	10418	Nova Roma Do Sul	0.76	0.87	0.95	162	0	0
BRA	Brazil	10419	Nova Santa Rita	0.49	0.77	0.98	8668	939	0
BRA	Brazil	10420	Novo Barreiro	0.31	0.64	0.89	2041	653	0
BRA	Brazil	10421	Novo Cabrais	0.65	0.82	0.95	623	0	0
BRA	Brazil	10422	Novo Hamburgo	0.32	0.64	0.87	114086	38117	0
BRA	Brazil	10423	Novo Machado	0.74	0.85	0.94	231	0	0
BRA	Brazil	10424	Novo Tiradentes	0.27	0.61	0.84	1179	419	0
BRA	Brazil	10425	Osorio	0.18	0.54	0.77	29026	12217	1411
BRA	Brazil	10426	Paim Filho	0.17	0.49	0.65	2570	1256	613
BRA	Brazil	10427	Palmares Do Sul	0.18	0.53	0.73	7492	3266	880
BRA	Brazil	10428	Palmeira Das Missoes	0.24	0.67	0.92	20529	4769	0
BRA	Brazil	10429	Palmitinho	0.50	0.78	0.97	2144	126	0
BRA	Brazil	10430	Panambi	0.61	0.82	0.98	8138	0	0
BRA	Brazil	10431	Pantano Grande	0.88	0.94	0.98	0	0	0
BRA	Brazil	10432	Parai	0.79	0.88	0.96	86	0	0
BRA	Brazil	10433	Paraiso Do Sul	0.57	0.78	0.93	1778	194	0
BRA	Brazil	10434	Pareci Novo	0.26	0.63	0.89	2379	743	0
BRA	Brazil	10435	Parobe	0.28	0.65	0.91	29766	8797	0
BRA	Brazil	10436	Passa Sete	0.63	0.81	0.95	922	0	0
BRA	Brazil	10437	Passo Do Sobrado	0.60	0.77	0.92	1433	194	0
BRA	Brazil	10438	Passo Fundo	0.13	0.52	0.76	133432	55401	8162
BRA	Brazil	10439	Paverama	0.17	0.49	0.65	5827	2847	1390
BRA	Brazil	10440	Pedro Osorio	0.17	0.50	0.66	4966	2373	1066

BRA	Brazil	10441	Pejucara	0.17	0.49	0.65	2543	1243	607
BRA	Brazil	10442	Pelotas	0.08	0.46	0.67	246700	116626	43418
BRA	Brazil	10443	Picada Cafe	0.68	0.83	0.94	663	0	0
BRA	Brazil	10444	Pinhal	0.29	0.63	0.87	1356	449	0
BRA	Brazil	10445	Pinhal Grande	0.89	0.94	0.96	0	0	0
BRA	Brazil	10446	Pinheirinho Do Vale	0.37	0.72	0.95	2290	400	0
BRA	Brazil	10447	Pinheiro Machado	0.32	0.67	0.94	6514	1777	0
BRA	Brazil	10448	Pirapo	0.76	0.88	0.96	112	0	0
BRA	Brazil	10449	Piratini	0.59	0.78	0.94	4413	359	0
BRA	Brazil	10450	Planalto	0.17	0.49	0.65	6644	3246	1585
BRA	Brazil	10451	Poco Das Antas	0.46	0.72	0.93	688	170	0
BRA	Brazil	10452	Pontao	0.14	0.60	0.85	2743	846	0
BRA	Brazil	10453	Ponte Preta	0.38	0.66	0.85	799	273	0
BRA	Brazil	10454	Portao	0.29	0.64	0.89	18813	6056	0
BRA	Brazil	10455	Porto Alegre	0.42	0.72	0.93	565091	114088	0
BRA	Brazil	10456	Porto Lucena	0.53	0.80	0.95	1357	15	0
BRA	Brazil	10457	Porto Maua	0.62	0.83	0.96	455	0	0
BRA	Brazil	10458	Porto Vera Cruz	0.48	0.76	0.94	543	65	0
BRA	Brazil	10459	Porto Xavier	0.38	0.81	0.98	4338	0	0
BRA	Brazil	10460	Pouso Novo	0.47	0.70	0.89	607	183	0
BRA	Brazil	10461	Presidente Lucena	0.46	0.72	0.92	999	238	0
BRA	Brazil	10462	Progresso	0.71	0.82	0.92	664	0	0
BRA	Brazil	10463	Protasio Alves	0.23	0.56	0.74	1147	474	128
BRA	Brazil	10464	Putinga	0.35	0.65	0.89	1863	612	0
BRA	Brazil	10465	Quarai	0.27	0.63	0.88	13645	4261	0
BRA	Brazil	10466	Quevedos	0.59	0.81	0.96	595	0	0
BRA	Brazil	10467	Quinze De Novembro	0.91	0.94	0.98	0	0	0
BRA	Brazil	10468	Redentora	0.25	0.70	0.97	6413	1182	0
BRA	Brazil	10469	Relvado	0.38	0.66	0.88	935	321	0
BRA	Brazil	10470	Restinga Seca	0.52	0.75	0.93	4474	816	0
BRA	Brazil	10471	Rio Dos Indios	0.08	0.40	0.55	2408	1328	825
BRA	Brazil	10472	Rio Grande	0.08	0.53	0.74	146376	54817	13112
BRA	Brazil	10473	Rio Pardo	0.71	0.84	0.95	3673	0	0

BRA	Brazil	10474	Riozinho	0.35	0.64	0.87	2044	709	0
BRA	Brazil	10475	Roca Sales	0.34	0.64	0.87	5892	2088	0
BRA	Brazil	10476	Rodeio Bonito	0.24	0.63	0.88	3341	1016	0
BRA	Brazil	10477	Rolante	0.26	0.65	0.90	10643	3052	0
BRA	Brazil	10478	Ronda Alta	0.21	0.67	0.91	6246	1346	0
BRA	Brazil	10479	Rondinha	0.42	0.73	0.90	2085	395	0
BRA	Brazil	10480	Roque Gonzales	0.62	0.85	0.96	1340	0	0
BRA	Brazil	10481	Rosario Do Sul	0.33	0.66	0.91	18895	5733	0
BRA	Brazil	10482	Sagrada Familia	0.54	0.77	0.93	681	89	0
BRA	Brazil	10483	Saldanha Marinho	0.54	0.78	0.95	722	60	0
BRA	Brazil	10484	Salto Do Jacui	0.79	0.90	0.98	191	0	0
BRA	Brazil	10485	Salvador Das Missoes	0.53	0.80	0.93	774	2	0
BRA	Brazil	10486	Salvador Do Sul	0.51	0.76	0.95	2473	303	0
BRA	Brazil	10487	Sananduva	0.74	0.85	0.94	1037	0	0
BRA	Brazil	10488	Santa Barbara Do Sul	0.90	0.94	0.98	0	0	0
BRA	Brazil	10489	Santa Clara Do Sul	0.51	0.75	0.94	1909	327	0
BRA	Brazil	10490	Santa Cruz Do Sul	0.85	0.91	0.97	0	0	0
BRA	Brazil	10491	Santa Maria	0.17	0.49	0.65	175769	85888	41934
BRA	Brazil	10492	Santa Maria Do Herval	0.75	0.83	0.90	332	0	0
BRA	Brazil	10493	Santa Rosa	0.48	0.83	0.98	23408	0	0
BRA	Brazil	10494	Santa Tereza	0.17	0.49	0.65	1142	558	272
BRA	Brazil	10495	Santa Vitoria Do Palmar	0.08	0.41	0.57	22331	12050	7077
BRA	Brazil	10496	Santana Da Boa Vista	0.67	0.82	0.94	1109	0	0
BRA	Brazil	10497	Santana Do Livramento	0.24	0.63	0.87	49368	14986	0
BRA	Brazil	10498	Santiago	0.38	0.72	0.94	22348	4051	0
BRA	Brazil	10499	Santo Angelo	0.62	0.88	0.98	14112	0	0
BRA	Brazil	10500	Santo Antonio Da Patrulha	0.19	0.54	0.72	26547	11356	3347
BRA	Brazil	10501	Santo Antonio Das Missoes	0.44	0.80	0.99	3861	47	0
BRA	Brazil	10502	Santo Antonio Do Palma	0.72	0.84	0.92	179	0	0
BRA	Brazil	10503	Santo Antonio Do Planalto	0.54	0.75	0.92	539	93	0
BRA	Brazil	10504	Santo Augusto	0.57	0.78	0.93	3195	279	0
BRA	Brazil	10505	Santo Cristo	0.38	0.79	0.96	6097	175	0
BRA	Brazil	10506	Santo Expedito Do Sul	0.21	0.57	0.77	1453	571	70

BRA	Brazil	10507	Sao Borja	0.34	0.77	0.99	28317	2018	0
BRA	Brazil	10508	Sao Domingos Do Sul	0.85	0.90	0.94	0	0	0
BRA	Brazil	10509	Sao Francisco De Assis	0.35	0.72	0.97	8547	1501	0
BRA	Brazil	10510	Sao Francisco De Paula	0.17	0.49	0.65	13735	6712	3277
BRA	Brazil	10511	Sao Gabriel	0.17	0.50	0.67	40957	19486	8691
BRA	Brazil	10512	Sao Jeronimo	0.45	0.72	0.95	9224	2040	0
BRA	Brazil	10513	Sao Joao Da Urtiga	0.70	0.84	0.95	491	0	0
BRA	Brazil	10514	Sao Joao Do Polesine	0.94	0.95	0.96	0	0	0
BRA	Brazil	10515	Sao Jorge	0.92	0.94	0.96	0	0	0
BRA	Brazil	10516	Sao Jose Das Missoes	0.23	0.68	0.91	1506	309	0
BRA	Brazil	10517	Sao Jose Do Herval	0.32	0.64	0.88	1018	349	0
BRA	Brazil	10518	Sao Jose Do Hortencio	0.59	0.79	0.94	962	61	0
BRA	Brazil	10519	Sao Jose Do Inhacora	0.17	0.49	0.65	1365	667	326
BRA	Brazil	10520	Sao Jose Do Norte	0.08	0.45	0.66	15762	7653	3143
BRA	Brazil	10521	Sao Jose Do Ouro	0.85	0.91	0.95	0	0	0
BRA	Brazil	10522	Sao Jose Dos Ausentes	0.90	0.94	0.98	0	0	0
BRA	Brazil	10523	Sao Leopoldo	0.26	0.64	0.91	127530	37236	0
BRA	Brazil	10524	Sao Lourenco Do Sul	0.11	0.52	0.74	30219	12374	2770
BRA	Brazil	10525	Sao Luiz Gonzaga	0.38	0.77	0.98	15619	953	0
BRA	Brazil	10526	Sao Marcos	0.68	0.84	0.97	2570	0	0
BRA	Brazil	10527	Sao Martinho	0.53	0.81	0.96	1517	0	0
BRA	Brazil	10528	Sao Martinho Da Serra	0.88	0.92	0.95	0	0	0
BRA	Brazil	10529	Sao Miguel Das Missoes	0.61	0.87	0.97	1474	0	0
BRA	Brazil	10530	Sao Nicolau	0.50	0.82	0.98	1712	0	0
BRA	Brazil	10531	Sao Paulo Das Missoes	0.69	0.84	0.95	684	0	0
BRA	Brazil	10532	Sao Pedro Da Serra	0.47	0.76	0.96	1178	129	0
BRA	Brazil	10533	Sao Pedro Do Butia	0.38	0.70	0.92	1232	301	0
BRA	Brazil	10534	Sao Pedro Do Sul	0.33	0.67	0.93	7812	2121	0
BRA	Brazil	10535	Sao Sebastiao Do Cai	0.20	0.58	0.78	13798	5128	351
BRA	Brazil	10536	Sao Sepe	0.47	0.74	0.95	8063	1428	0
BRA	Brazil	10537	Sao Valentim	0.13	0.54	0.77	2328	920	92
BRA	Brazil	10538	Sao Valentim Do Sul	0.48	0.70	0.87	707	225	0
BRA	Brazil	10539	Sao Valerio Do Sul	0.27	0.73	0.95	1479	188	0

BRA	Brazil	10540	Sao Vendelino	0.56	0.76	0.93	602	92	0
BRA	Brazil	10541	Sao Vicente Do Sul	0.28	0.68	0.93	4621	1088	0
BRA	Brazil	10542	Sapiranga	0.37	0.67	0.90	32487	9881	0
BRA	Brazil	10543	Sapucaia Do Sul	0.18	0.51	0.68	85796	40097	16957
BRA	Brazil	10544	Sarandi	0.08	0.40	0.55	17055	9410	5848
BRA	Brazil	10545	Seberi	0.25	0.62	0.84	6169	2025	0
BRA	Brazil	10546	Sede Nova	0.54	0.81	0.96	773	0	0
BRA	Brazil	10547	Segredo	0.79	0.89	0.97	97	0	0
BRA	Brazil	10548	Selbach	0.76	0.88	0.96	224	0	0
BRA	Brazil	10549	Senador Salgado Filho	0.76	0.89	0.95	125	0	0
BRA	Brazil	10550	Sentinela Do Sul	0.18	0.54	0.73	3446	1470	364
BRA	Brazil	10551	Serafina Correa	0.68	0.83	0.94	2071	0	0
BRA	Brazil	10552	Serio	0.71	0.83	0.92	187	0	0
BRA	Brazil	10553	Sertao	0.11	0.49	0.70	4189	1856	619
BRA	Brazil	10554	Sertao Santana	0.65	0.81	0.95	920	0	0
BRA	Brazil	10555	Sete De Setembro	0.65	0.85	0.95	327	0	0
BRA	Brazil	10556	Severiano De Almeida	0.58	0.78	0.93	802	68	0
BRA	Brazil	10557	Silveira Martins	0.87	0.91	0.94	0	0	0
BRA	Brazil	10558	Sinimbu	0.73	0.85	0.94	716	0	0
BRA	Brazil	10559	Sobradinho	0.21	0.56	0.77	10570	4332	519
BRA	Brazil	10560	Soledade	0.17	0.49	0.65	19760	9655	4714
BRA	Brazil	10561	Tabai	0.28	0.60	0.85	2324	874	0
BRA	Brazil	10562	Tapejara	0.17	0.52	0.70	14230	6421	2282
BRA	Brazil	10563	Tapera	0.79	0.88	0.95	155	0	0
BRA	Brazil	10564	Tapes	0.21	0.56	0.76	10246	4090	682
BRA	Brazil	10565	Taquara	0.29	0.66	0.93	28829	7712	0
BRA	Brazil	10566	Taquari	0.49	0.71	0.89	8425	2288	0
BRA	Brazil	10567	Taquarucu Do Sul	0.17	0.49	0.65	1940	948	463
BRA	Brazil	10568	Tavares	0.08	0.40	0.55	3991	2202	1368
BRA	Brazil	10569	Tenente Portela	0.17	0.49	0.65	8772	4287	2093
BRA	Brazil	10570	Terra De Areia	0.08	0.41	0.58	9055	4847	2783
BRA	Brazil	10571	Teutonia	0.50	0.76	0.96	9882	1267	0
BRA	Brazil	10572	Tiradentes Do Sul	0.34	0.74	0.96	2880	345	0

BRA	Brazil	10573	Toropi	0.17	0.49	0.65	1683	822	402
BRA	Brazil	10574	Torres	0.08	0.40	0.55	26448	14592	9068
BRA	Brazil	10575	Tramandai	0.08	0.44	0.63	34862	17449	8234
BRA	Brazil	10576	Travesseiro	0.50	0.71	0.88	788	244	0
BRA	Brazil	10577	Tres Arroios	0.08	0.40	0.55	2111	1164	724
BRA	Brazil	10578	Tres Cachoeiras	0.08	0.43	0.61	7914	4073	2107
BRA	Brazil	10579	Tres Coroas	0.30	0.68	0.95	12493	3139	0
BRA	Brazil	10580	Tres De Maio	0.95	0.97	0.98	0	0	0
BRA	Brazil	10581	Tres Forquilhas	0.13	0.48	0.66	1944	937	412
BRA	Brazil	10582	Tres Palmeiras	0.26	0.69	0.92	2362	469	0
BRA	Brazil	10583	Tres Passos	0.37	0.75	0.96	10583	1150	0
BRA	Brazil	10584	Trindade Do Sul	0.28	0.71	0.94	3120	519	0
BRA	Brazil	10585	Triunfo	0.17	0.49	0.65	18118	8853	4323
BRA	Brazil	10586	Tucunduva	0.50	0.80	0.95	1768	19	0
BRA	Brazil	10587	Tunas	0.95	0.97	0.98	0	0	0
BRA	Brazil	10588	Tupanci Do Sul	0.92	0.94	0.95	0	0	0
BRA	Brazil	10589	Tupancireta	0.64	0.86	0.98	3792	0	0
BRA	Brazil	10590	Tupandi	0.42	0.71	0.94	1887	432	0
BRA	Brazil	10591	Tuparendi	0.17	0.49	0.65	5302	2591	1265
BRA	Brazil	10592	Turucu	0.08	0.42	0.60	2565	1335	709
BRA	Brazil	10593	Ubiretama	0.66	0.86	0.94	306	0	0
BRA	Brazil	10594	Uniao Da Serra	0.70	0.81	0.91	143	0	0
BRA	Brazil	10595	Unistalda	0.91	0.95	0.97	0	0	0
BRA	Brazil	10596	Uruguaiana	0.29	0.71	0.98	65064	11166	0
BRA	Brazil	10597	Vacaria	0.82	0.91	0.99	0	0	0
BRA	Brazil	10598	Vale Do Sol	0.77	0.87	0.95	327	0	0
BRA	Brazil	10599	Vale Real	0.66	0.83	0.96	773	0	0
BRA	Brazil	10600	Vale Verde	0.75	0.85	0.94	162	0	0
BRA	Brazil	10601	Vanini	0.84	0.89	0.92	0	0	0
BRA	Brazil	10602	Venancio Aires	0.81	0.89	0.96	0	0	0
BRA	Brazil	10603	Vera Cruz	0.17	0.49	0.65	16506	8066	3938
BRA	Brazil	10604	Veranopolis	0.82	0.90	0.96	0	0	0
BRA	Brazil	10605	Vespasiano Correa	0.53	0.74	0.91	556	119	0

BRA	Brazil	10606	Viadutos	0.21	0.57	0.77	3053	1197	153
BRA	Brazil	10607	Viamao	0.35	0.68	0.94	112539	30744	0
BRA	Brazil	10608	Vicente Dutra	0.33	0.71	0.96	2443	458	0
BRA	Brazil	10609	Victor Graeff	0.94	0.97	0.99	0	0	0
BRA	Brazil	10610	Vila Flores	0.74	0.85	0.94	220	0	0
BRA	Brazil	10611	Vila Langaro	0.16	0.51	0.74	1364	624	123
BRA	Brazil	10612	Vila Maria	0.68	0.82	0.94	541	0	0
BRA	Brazil	10613	Vila Nova Do Sul	0.68	0.84	0.96	501	0	0
BRA	Brazil	10614	Vista Alegre	0.48	0.78	0.96	890	56	0
BRA	Brazil	10615	Vista Alegre Do Prata	0.65	0.81	0.94	244	0	0
BRA	Brazil	10616	Vista Gaucha	0.37	0.75	0.94	1196	141	0
BRA	Brazil	10617	Vitoria Das Missoes	0.80	0.87	0.93	0	0	0
BRA	Brazil	10618	Xangri-la	0.08	0.41	0.58	11747	6279	3611
BRA	Brazil	10619	Alta Floresta D'oeste	0.43	0.92	1.00	9128	0	0
BRA	Brazil	10620	Alto Alegre Dos Parecis	0.40	0.90	1.00	5165	0	0
BRA	Brazil	10621	Alto Paraiso	0.37	0.89	0.99	8697	0	0
BRA	Brazil	10622	Alvorada D'oeste	0.36	0.91	1.00	7041	0	0
BRA	Brazil	10623	Ariquemes	0.42	0.93	1.00	40334	0	0
BRA	Brazil	10624	Buritis	0.21	0.66	0.75	22188	5419	1752
BRA	Brazil	10625	Cabixi	0.40	0.88	0.99	2685	0	0
BRA	Brazil	10626	Cacaulandia	0.45	0.90	0.99	2216	0	0
BRA	Brazil	10627	Cacoal	0.39	0.91	1.00	34224	0	0
BRA	Brazil	10628	Campo Novo De Rondonia	0.33	0.85	0.95	6427	0	0
BRA	Brazil	10629	Candeias Do Jamari	0.36	0.90	1.00	10175	0	0
BRA	Brazil	10630	Castanheiras	0.42	0.91	0.98	1300	0	0
BRA	Brazil	10631	Cerejeiras	0.47	0.92	1.00	5528	0	0
BRA	Brazil	10632	Chupinguaia	0.39	0.91	1.00	4253	0	0
BRA	Brazil	10633	Colorado Do Oeste	0.47	0.92	1.00	5677	0	0
BRA	Brazil	10634	Corumbiara	0.39	0.93	1.00	3426	0	0
BRA	Brazil	10635	Costa Marques	0.39	0.88	1.00	6681	0	0
BRA	Brazil	10636	Cujubim	0.34	0.83	0.97	11707	0	0
BRA	Brazil	10637	Espigao D'oeste	0.39	0.90	1.00	12810	0	0
BRA	Brazil	10638	Governador Jorge Teixeira	0.47	0.93	1.00	3186	0	0

BRA	Brazil	10639	Guajara-mirim	0.45	0.93	1.00	16131	0	0
BRA	Brazil	10640	Jamari	0.44	0.93	1.00	3545	0	0
BRA	Brazil	10641	Jaru	0.36	0.88	1.00	23172	0	0
BRA	Brazil	10642	Ji-parana	0.39	0.89	1.00	51116	0	0
BRA	Brazil	10643	Machadinho D'oeste	0.37	0.89	1.00	16165	0	0
BRA	Brazil	10644	Ministro Andreazza	0.40	0.90	1.00	4227	0	0
BRA	Brazil	10645	Mirante Da Serra	0.44	0.90	0.99	4163	0	0
BRA	Brazil	10646	Monte Negro	0.45	0.95	1.00	4478	0	0
BRA	Brazil	10647	Nova Brasilandia D'oeste	0.39	0.92	1.00	7773	0	0
BRA	Brazil	10648	Nova Mamore	0.36	0.90	1.00	12451	0	0
BRA	Brazil	10649	Nova Uniao	0.21	0.66	0.75	4345	1061	343
BRA	Brazil	10650	Novo Horizonte Do Oeste	0.44	0.91	0.99	3393	0	0
BRA	Brazil	10651	Ouro Preto Do Oeste	0.38	0.91	1.00	15796	0	0
BRA	Brazil	10652	Parecis	0.42	0.89	0.99	2177	0	0
BRA	Brazil	10653	Pimenta Bueno	0.41	0.91	1.00	14225	0	0
BRA	Brazil	10654	Pimenteiras Do Oeste	0.70	0.95	1.00	156	0	0
BRA	Brazil	10655	Porto Velho	0.37	0.91	1.00	214113	0	0
BRA	Brazil	10656	Presidente Medici	0.21	0.66	0.75	12336	3013	974
BRA	Brazil	10657	Primavera De Rondonia	0.49	0.95	1.00	1042	0	0
BRA	Brazil	10658	Rio Crespo	0.61	0.93	0.99	715	0	0
BRA	Brazil	10659	Rolim De Moura	0.39	0.90	1.00	22338	0	0
BRA	Brazil	10660	Santa Luzia D'oeste	0.44	0.91	1.00	2879	0	0
BRA	Brazil	10661	Sao Felipe D'oeste	0.43	0.93	1.00	2099	0	0
BRA	Brazil	10662	Sao Francisco Do Guapore	0.36	0.87	1.00	8681	0	0
BRA	Brazil	10663	Sao Miguel Do Guapore	0.38	0.88	0.99	10427	0	0
BRA	Brazil	10664	Seringueiras	0.38	0.91	1.00	4981	0	0
BRA	Brazil	10665	Teixeiropolis	0.45	0.93	1.00	1638	0	0
BRA	Brazil	10666	Theobroma	0.35	0.87	0.99	4820	0	0
BRA	Brazil	10667	Urupa	0.39	0.90	1.00	5142	0	0
BRA	Brazil	10668	Vale Do Anari	0.35	0.86	0.98	4763	0	0
BRA	Brazil	10669	Vale Do Paraiso	0.40	0.91	1.00	3039	0	0
BRA	Brazil	10670	Vilhena	0.40	0.89	1.00	36956	0	0
BRA	Brazil	10671	Alto Alegre	0.21	0.66	0.75	9637	2354	761

BRA	Brazil	10672	Amajari	0.36	0.90	1.00	5756	0	0
BRA	Brazil	10673	Boa Vista	0.21	0.66	0.75	206713	50489	16322
BRA	Brazil	10674	Bonfim	0.21	0.66	0.75	7112	1737	562
BRA	Brazil	10675	Canta	0.32	0.86	0.99	8987	0	0
BRA	Brazil	10676	Caracarai	0.36	0.89	1.00	9560	0	0
BRA	Brazil	10677	Caroebe	0.40	0.91	1.00	3951	0	0
BRA	Brazil	10678	Iracema	0.21	0.66	0.75	7028	1717	555
BRA	Brazil	10679	Mucajai	0.35	0.89	1.00	7801	0	0
BRA	Brazil	10680	Normandia	0.38	0.91	1.00	4693	0	0
BRA	Brazil	10681	Pacaraima	0.91	0.99	1.00	0	0	0
BRA	Brazil	10682	Rorainopolis	0.35	0.86	0.99	13650	0	0
BRA	Brazil	10683	Sao Joao Da Baliza	0.38	0.89	1.00	3412	0	0
BRA	Brazil	10684	Sao Luiz	0.37	0.85	0.96	3392	0	0
BRA	Brazil	10685	Uiramuta	0.41	0.93	1.00	4009	0	0
BRA	Brazil	10686	Abdon Batista	0.80	0.90	0.99	0	0	0
BRA	Brazil	10687	Abelardo Luz	0.17	0.49	0.65	11951	5840	2851
BRA	Brazil	10688	Agrolandia	0.08	0.40	0.55	7571	4177	2596
BRA	Brazil	10689	Agronomica	0.08	0.42	0.60	4009	2085	1095
BRA	Brazil	10690	Agua Doce	0.38	0.80	0.97	3096	0	0
BRA	Brazil	10691	Aguas De Chapeco	0.36	0.80	0.95	2716	0	0
BRA	Brazil	10692	Aguas Frias	0.08	0.40	0.55	1773	978	608
BRA	Brazil	10693	Aguas Mornas	0.08	0.40	0.55	3997	2206	1371
BRA	Brazil	10694	Alfredo Wagner	0.08	0.42	0.58	7189	3819	2160
BRA	Brazil	10695	Alto Bela Vista	0.47	0.73	0.90	728	156	0
BRA	Brazil	10696	Anchieta	0.17	0.49	0.65	3935	1923	939
BRA	Brazil	10697	Angelina	0.08	0.41	0.56	3867	2105	1264
BRA	Brazil	10698	Anita Garibaldi	0.17	0.49	0.65	5248	2564	1252
BRA	Brazil	10699	Anitapolis	0.08	0.40	0.55	2552	1408	875
BRA	Brazil	10700	Antonio Carlos	0.08	0.40	0.55	6342	3499	2175
BRA	Brazil	10701	Apiuna	0.08	0.42	0.58	7380	3921	2218
BRA	Brazil	10702	Arabuta	0.08	0.40	0.55	2994	1652	1026
BRA	Brazil	10703	Araquari	0.08	0.40	0.55	27808	15342	9534
BRA	Brazil	10704	Ararangua	0.08	0.40	0.55	48113	26545	16496

BRA	Brazil	10705	Armazem	0.08	0.42	0.60	5606	2926	1571
BRA	Brazil	10706	Arroio Trinta	0.21	0.64	0.87	2286	631	0
BRA	Brazil	10707	Arvoredo	0.08	0.40	0.55	1606	886	551
BRA	Brazil	10708	Ascurra	0.08	0.40	0.55	6105	3368	2093
BRA	Brazil	10709	Atalanta	0.08	0.40	0.55	2339	1290	802
BRA	Brazil	10710	Aurora	0.08	0.40	0.55	4538	2504	1556
BRA	Brazil	10711	Balneario Arroio Do Silva	0.08	0.40	0.55	8951	4938	3069
BRA	Brazil	10712	Balneario Barra Do Sul	0.08	0.40	0.55	7105	3920	2436
BRA	Brazil	10713	Balneario Camboriu	0.08	0.40	0.55	92419	50990	31687
BRA	Brazil	10714	Balneario Gaivota	0.08	0.43	0.60	7533	3876	2051
BRA	Brazil	10715	Bandeirante	0.76	0.89	0.98	122	0	0
BRA	Brazil	10716	Barra Bonita	1.00	1.00	1.00	0	0	0
BRA	Brazil	10717	Barra Velha	0.08	0.42	0.59	19535	10242	5597
BRA	Brazil	10718	Bela Vista Do Toldo	0.27	0.67	0.93	3376	825	0
BRA	Brazil	10719	Belmonte	0.17	0.49	0.65	1732	846	413
BRA	Brazil	10720	Benedito Novo	0.08	0.40	0.55	8108	4473	2780
BRA	Brazil	10721	Biguacu	0.08	0.40	0.55	42261	23317	14490
BRA	Brazil	10722	Blumenau	0.08	0.44	0.63	248095	123650	57348
BRA	Brazil	10723	Bocaina Do Sul	0.08	0.40	0.55	2510	1385	860
BRA	Brazil	10724	Bom Jardim Da Serra	0.30	0.62	0.85	2353	863	0
BRA	Brazil	10725	Bom Jesus	0.08	0.40	0.55	2105	1161	722
BRA	Brazil	10726	Bom Jesus Do Oeste	0.64	0.80	0.92	353	9	0
BRA	Brazil	10727	Bom Retiro	0.08	0.40	0.55	7268	4010	2492
BRA	Brazil	10728	Bombinhas	0.08	0.44	0.63	12778	6416	3081
BRA	Brazil	10729	Botuvera	0.08	0.40	0.55	4222	2329	1447
BRA	Brazil	10730	Braco Do Norte	0.08	0.40	0.55	20033	11052	6868
BRA	Brazil	10731	Braco Do Trombudo	0.08	0.42	0.58	2624	1393	787
BRA	Brazil	10732	Brunopolis	0.17	0.49	0.65	1713	837	409
BRA	Brazil	10733	Brusque	0.08	0.44	0.63	93733	47081	22374
BRA	Brazil	10734	Cacador	0.20	0.62	0.88	46629	13843	0
BRA	Brazil	10735	Caibi	0.42	0.80	0.97	2465	0	0
BRA	Brazil	10736	Calmon	0.08	0.40	0.55	2551	1408	875
BRA	Brazil	10737	Camboriu	0.08	0.42	0.59	58549	30980	17427

BRA	Brazil	10738	Campo Alegre	0.08	0.40	0.55	9378	5174	3215
BRA	Brazil	10739	Campo Belo Do Sul	0.99	0.99	1.00	0	0	0
BRA	Brazil	10740	Campo Ere	0.36	0.82	0.99	3879	0	0
BRA	Brazil	10741	Campos Novos	0.17	0.49	0.65	23708	11585	5656
BRA	Brazil	10742	Canelinha	0.08	0.41	0.57	8694	4672	2722
BRA	Brazil	10743	Canoinhas	0.19	0.59	0.81	33869	11864	0
BRA	Brazil	10744	Capao Alto	0.77	0.89	0.97	69	0	0
BRA	Brazil	10745	Capinzal	0.17	0.49	0.65	13656	6673	3258
BRA	Brazil	10746	Capivari De Baixo	0.08	0.44	0.64	17186	8494	3726
BRA	Brazil	10747	Catanduvas	0.08	0.40	0.55	7647	4219	2622
BRA	Brazil	10748	Caxambu Do Sul	0.23	0.67	0.90	2415	557	0
BRA	Brazil	10749	Celso Ramos	0.92	0.96	0.99	0	0	0
BRA	Brazil	10750	Cerro Negro	0.17	0.49	0.65	2154	1052	514
BRA	Brazil	10751	Chapadao Do Lageado	0.08	0.41	0.57	1988	1074	636
BRA	Brazil	10752	Chapeco	0.08	0.40	0.55	152511	84144	52291
BRA	Brazil	10753	Cocal Do Sul	0.08	0.44	0.63	12525	6318	3016
BRA	Brazil	10754	Concordia	0.19	0.68	0.91	45462	8860	0
BRA	Brazil	10755	Cordilheira Alta	0.24	0.72	0.90	2463	358	0
BRA	Brazil	10756	Coronel Freitas	0.19	0.58	0.80	6284	2261	0
BRA	Brazil	10757	Coronel Martins	0.17	0.49	0.65	1523	744	363
BRA	Brazil	10758	Correia Pinto	0.08	0.40	0.55	9999	5517	3428
BRA	Brazil	10759	Corupa	0.08	0.45	0.65	11111	5450	2366
BRA	Brazil	10760	Criciuma	0.08	0.44	0.63	147523	74076	34885
BRA	Brazil	10761	Cunha Pora	0.56	0.81	0.96	2715	0	0
BRA	Brazil	10762	Cunhatai	0.53	0.79	0.93	521	21	0
BRA	Brazil	10763	Curitibanos	0.19	0.61	0.86	24176	7415	0
BRA	Brazil	10764	Descanso	0.70	0.86	0.97	833	0	0
BRA	Brazil	10765	Dionisio Cerqueira	0.17	0.49	0.65	2377	1162	567
BRA	Brazil	10766	Dona Emma	0.08	0.40	0.55	2813	1552	964
BRA	Brazil	10767	Doutor Pedrinho	0.12	0.47	0.65	2608	1257	565
BRA	Brazil	10768	Entre Rios	0.08	0.40	0.55	2304	1271	790
BRA	Brazil	10769	Ermo	0.08	0.43	0.62	1602	815	401
BRA	Brazil	10770	Erval Velho	0.33	0.70	0.95	2209	457	0

BRA	Brazil	10771	Faxinal Dos Guedes	0.34	0.75	0.96	4887	546	0
BRA	Brazil	10772	Flor Do Sertao	0.17	0.49	0.65	1088	532	260
BRA	Brazil	10773	Florianopolis	0.08	0.48	0.72	340208	152875	39472
BRA	Brazil	10774	Formosa Do Sul	0.08	0.40	0.55	1861	1026	638
BRA	Brazil	10775	Forquilhinha	0.08	0.42	0.59	18720	9825	5370
BRA	Brazil	10776	Fraiburgo	0.32	0.80	1.00	13990	0	0
BRA	Brazil	10777	Frei Rogerio	0.45	0.78	0.96	788	51	0
BRA	Brazil	10778	Galvao	0.17	0.49	0.65	2134	1043	509
BRA	Brazil	10779	Garopaba	0.08	0.40	0.55	14619	8066	5012
BRA	Brazil	10780	Garuva	0.08	0.40	0.55	12434	6860	4263
BRA	Brazil	10781	Gaspar	0.08	0.40	0.55	46663	25745	15999
BRA	Brazil	10782	Governador Celso Ramos	0.08	0.40	0.55	10522	5805	3608
BRA	Brazil	10783	Grao Para	0.08	0.40	0.55	4456	2459	1528
BRA	Brazil	10784	Gravatal	0.08	0.40	0.55	8368	4617	2869
BRA	Brazil	10785	Guabiruba	0.08	0.43	0.61	16432	8418	4310
BRA	Brazil	10786	Guaraciaba	0.17	0.49	0.65	6870	3357	1639
BRA	Brazil	10787	Guaramirim	0.08	0.45	0.65	30281	14640	6170
BRA	Brazil	10788	Guaruja Do Sul	0.60	0.83	0.97	1039	0	0
BRA	Brazil	10789	Guatambu	0.26	0.71	0.92	2664	418	0
BRA	Brazil	10790	Herval D'oeste	0.22	0.63	0.89	12748	3752	0
BRA	Brazil	10791	Ibiam	0.28	0.72	0.94	1024	151	0
BRA	Brazil	10792	Ibicare	0.08	0.40	0.55	2559	1412	877
BRA	Brazil	10793	Ibirama	0.08	0.40	0.55	13446	7418	4610
BRA	Brazil	10794	Icara	0.08	0.40	0.55	52250	28828	17915
BRA	Brazil	10795	Ilhota	0.08	0.40	0.55	9511	5248	3261
BRA	Brazil	10796	Imarui	0.08	0.40	0.55	8086	4461	2772
BRA	Brazil	10797	Imbituba	0.08	0.40	0.55	31982	17645	10965
BRA	Brazil	10798	Imbuia	0.08	0.40	0.55	4229	2333	1450
BRA	Brazil	10799	Indaial	0.08	0.42	0.59	47974	25362	14148
BRA	Brazil	10800	Iomere	0.37	0.77	0.93	1236	74	0
BRA	Brazil	10801	Ipira	0.08	0.40	0.55	3748	2068	1285
BRA	Brazil	10802	Ipora Do Oeste	0.76	0.89	0.98	341	0	0
BRA	Brazil	10803	Ipuacu	0.32	0.80	1.00	3497	24	0

BRA	Brazil	10804	Ipumirim	0.22	0.70	0.92	4316	784	0
BRA	Brazil	10805	Iraceminha	0.41	0.73	0.96	1593	296	0
BRA	Brazil	10806	Irani	0.51	0.78	0.95	3011	217	0
BRA	Brazil	10807	Irati	0.08	0.40	0.55	1505	831	516
BRA	Brazil	10808	Irineopolis	0.26	0.73	0.96	5925	792	0
BRA	Brazil	10809	Ita	0.33	0.74	0.95	2997	362	0
BRA	Brazil	10810	Itaiopolis	0.08	0.40	0.55	15486	8544	5310
BRA	Brazil	10811	Itajai	0.08	0.40	0.55	156074	86110	53513
BRA	Brazil	10812	Itapema	0.08	0.45	0.65	39828	19370	8272
BRA	Brazil	10813	Itapiranga	0.17	0.49	0.65	10344	5055	2468
BRA	Brazil	10814	Itapoa	0.08	0.40	0.55	13361	7372	4581
BRA	Brazil	10815	Ituporanga	0.08	0.42	0.59	17713	9305	5093
BRA	Brazil	10816	Jabora	0.08	0.40	0.55	2923	1613	1002
BRA	Brazil	10817	Jacinto Machado	0.08	0.40	0.55	7421	4094	2544
BRA	Brazil	10818	Jaguaruna	0.08	0.40	0.55	13891	7664	4763
BRA	Brazil	10819	Jaragua Do Sul	0.08	0.45	0.65	122721	59808	26144
BRA	Brazil	10820	Jardinopolis	0.23	0.69	0.92	991	190	0
BRA	Brazil	10821	Joacaba	0.08	0.40	0.55	21681	11962	7434
BRA	Brazil	10822	Joinville	0.08	0.40	0.55	399559	220447	136996
BRA	Brazil	10823	Jose Boiteux	0.16	0.54	0.75	2624	1060	209
BRA	Brazil	10824	Jupia	0.32	0.78	0.97	1028	45	0
BRA	Brazil	10825	Lacerdopolis	0.48	0.79	0.97	671	14	0
BRA	Brazil	10826	Lages	0.17	0.49	0.65	101770	49729	24280
BRA	Brazil	10827	Laguna	0.08	0.43	0.60	38396	19905	10439
BRA	Brazil	10828	Lajeado Grande	0.08	0.40	0.55	1110	613	381
BRA	Brazil	10829	Laurentino	0.08	0.40	0.55	4726	2608	1620
BRA	Brazil	10830	Lauro Muller	0.08	0.43	0.61	10799	5562	2887
BRA	Brazil	10831	Lebon Regis	0.08	0.40	0.55	9089	5015	3116
BRA	Brazil	10832	Leoberto Leal	0.08	0.41	0.56	2424	1323	799
BRA	Brazil	10833	Lindoia Do Sul	0.08	0.40	0.55	3345	1845	1147
BRA	Brazil	10834	Lontras	0.08	0.40	0.55	8642	4768	2963
BRA	Brazil	10835	Luiz Alves	0.08	0.43	0.60	8831	4585	2448
BRA	Brazil	10836	Luzerna	0.31	0.64	0.85	2764	912	0

BRA	Brazil	10837	Macieira	0.38	0.79	0.97	727	22	0
BRA	Brazil	10838	Mafra	0.08	0.40	0.55	42445	23418	14553
BRA	Brazil	10839	Major Gercino	0.08	0.42	0.59	2478	1312	730
BRA	Brazil	10840	Major Vieira	0.08	0.40	0.55	5904	3257	2024
BRA	Brazil	10841	Maracaja	0.08	0.42	0.60	5198	2721	1462
BRA	Brazil	10842	Maravilha	0.17	0.49	0.65	15600	7623	3722
BRA	Brazil	10843	Marema	0.08	0.40	0.55	1463	807	502
BRA	Brazil	10844	Massaranduba	0.08	0.40	0.55	12462	6876	4273
BRA	Brazil	10845	Matos Costa	0.08	0.40	0.55	1993	1100	683
BRA	Brazil	10846	Meleiro	0.08	0.40	0.55	5127	2829	1758
BRA	Brazil	10847	Mirim Doce	0.08	0.43	0.61	1814	932	478
BRA	Brazil	10848	Modelo	0.36	0.73	0.90	1993	307	0
BRA	Brazil	10849	Mondai	0.59	0.82	0.96	2390	0	0
BRA	Brazil	10850	Monte Carlo	0.24	0.63	0.85	5550	1708	0
BRA	Brazil	10851	Monte Castelo	0.08	0.40	0.55	5877	3243	2015
BRA	Brazil	10852	Morro Da Fumaca	0.08	0.40	0.55	12439	6863	4265
BRA	Brazil	10853	Morro Grande	0.08	0.40	0.55	2103	1161	721
BRA	Brazil	10854	Navegantes	0.08	0.46	0.68	51643	24507	8751
BRA	Brazil	10855	Nova Erechim	0.19	0.58	0.81	2901	1036	0
BRA	Brazil	10856	Nova Itaberaba	0.08	0.40	0.55	3179	1754	1090
BRA	Brazil	10857	Nova Trento	0.08	0.40	0.55	10121	5584	3470
BRA	Brazil	10858	Nova Veneza	0.08	0.40	0.55	10025	5531	3437
BRA	Brazil	10859	Novo Horizonte	0.17	0.49	0.65	1701	831	406
BRA	Brazil	10860	Orleans	0.08	0.42	0.59	19582	10296	5700
BRA	Brazil	10861	Otacilio Costa	0.26	0.66	0.90	9896	2485	0
BRA	Brazil	10862	Ouro	0.45	0.77	0.94	2314	177	0
BRA	Brazil	10863	Ouro Verde	0.92	0.99	1.00	0	0	0
BRA	Brazil	10864	Paial	0.67	0.84	0.95	217	0	0
BRA	Brazil	10865	Painel	0.08	0.40	0.55	1836	1013	630
BRA	Brazil	10866	Palhoca	0.08	0.43	0.60	109255	56563	29966
BRA	Brazil	10867	Palma Sola	0.56	0.84	0.99	2040	0	0
BRA	Brazil	10868	Palmeira	0.08	0.40	0.55	2159	1191	740
BRA	Brazil	10869	Palmitos	0.49	0.81	0.98	5174	0	0

BRA	Brazil	10870	Papanduva	0.44	0.76	0.96	7150	823	0
BRA	Brazil	10871	Paraiso	0.54	0.92	1.00	1025	0	0
BRA	Brazil	10872	Passo De Torres	0.08	0.41	0.58	6475	3468	2005
BRA	Brazil	10873	Passos Maia	0.17	0.49	0.65	2507	1225	598
BRA	Brazil	10874	Paulo Lopes	0.08	0.40	0.55	5517	3044	1892
BRA	Brazil	10875	Pedras Grandes	0.08	0.40	0.55	2979	1644	1021
BRA	Brazil	10876	Penha	0.08	0.43	0.62	21235	10790	5251
BRA	Brazil	10877	Peritiba	0.08	0.40	0.55	2159	1191	740
BRA	Brazil	10878	Petrolandia	0.08	0.40	0.55	4475	2469	1534
BRA	Brazil	10879	Picarras	0.08	0.45	0.65	14257	6913	2875
BRA	Brazil	10880	Pinhalzinho	0.28	0.76	0.94	10070	867	0
BRA	Brazil	10881	Pinheiro Preto	0.20	0.61	0.86	2078	640	0
BRA	Brazil	10882	Piratuba	0.97	0.98	0.98	0	0	0
BRA	Brazil	10883	Planalto Alegre	0.08	0.40	0.55	1999	1103	685
BRA	Brazil	10884	Pomerode	0.08	0.44	0.64	23528	11709	5322
BRA	Brazil	10885	Ponte Alta	0.17	0.53	0.72	3157	1341	409
BRA	Brazil	10886	Ponte Alta Do Norte	0.31	0.71	0.97	1696	318	0
BRA	Brazil	10887	Ponte Serrada	0.08	0.40	0.55	8457	4666	2900
BRA	Brazil	10888	Porto Belo	0.08	0.42	0.60	17163	8963	4850
BRA	Brazil	10889	Porto Uniao	0.08	0.40	0.55	25955	14320	8899
BRA	Brazil	10890	Pouso Redondo	0.08	0.40	0.55	12105	6678	4150
BRA	Brazil	10891	Praia Grande	0.32	0.80	0.89	3443	0	0
BRA	Brazil	10892	Presidente Castelo Branco	0.08	0.40	0.55	1077	594	369
BRA	Brazil	10893	Presidente Getulio	0.08	0.40	0.55	12474	6882	4277
BRA	Brazil	10894	Presidente Nereu	0.08	0.41	0.57	1714	929	551
BRA	Brazil	10895	Princesa	0.67	0.86	0.98	398	0	0
BRA	Brazil	10896	Quilombo	0.08	0.40	0.55	7478	4126	2564
BRA	Brazil	10897	Rancho Queimado	0.08	0.43	0.61	2021	1043	537
BRA	Brazil	10898	Rio Das Antas	0.08	0.40	0.55	4749	2620	1628
BRA	Brazil	10899	Rio Do Campo	0.08	0.40	0.55	4361	2406	1495
BRA	Brazil	10900	Rio Do Oeste	0.08	0.40	0.55	5488	3028	1882
BRA	Brazil	10901	Rio Do Sul	0.08	0.40	0.55	49248	27171	16885
BRA	Brazil	10902	Rio Dos Cedros	0.08	0.40	0.55	7946	4384	2724

BRA	Brazil	10903	Rio Fortuna	0.08	0.46	0.67	3224	1539	571
BRA	Brazil	10904	Rio Negrinho	0.23	0.67	0.92	22949	5408	0
BRA	Brazil	10905	Rio Rufino	0.13	0.47	0.64	1734	854	416
BRA	Brazil	10906	Riqueza	0.17	0.49	0.65	2958	1446	706
BRA	Brazil	10907	Rodeio	0.08	0.42	0.60	8501	4446	2402
BRA	Brazil	10908	Romelandia	0.17	0.49	0.65	3495	1708	834
BRA	Brazil	10909	Salete	0.08	0.42	0.59	5414	2857	1587
BRA	Brazil	10910	Saltinho	0.25	0.66	0.89	2122	530	0
BRA	Brazil	10911	Salto Veloso	0.08	0.40	0.55	3333	1839	1143
BRA	Brazil	10912	Sangao	0.08	0.42	0.58	8612	4589	2633
BRA	Brazil	10913	Santa Cecilia	0.08	0.40	0.55	12164	6711	4171
BRA	Brazil	10914	Santa Helena	0.17	0.49	0.65	1485	726	354
BRA	Brazil	10915	Santa Rosa De Lima	0.08	0.40	0.55	1530	844	525
BRA	Brazil	10916	Santa Rosa Do Sul	0.08	0.40	0.55	6257	3452	2145
BRA	Brazil	10917	Santa Terezinha	0.08	0.40	0.55	6579	3630	2256
BRA	Brazil	10918	Santa Terezinha Do Progresso	0.66	0.85	0.97	371	0	0
BRA	Brazil	10919	Santiago Do Sul	0.08	0.40	0.55	1049	579	360
BRA	Brazil	10920	Santo Amaro Da Imperatriz	0.08	0.42	0.59	17309	9115	5037
BRA	Brazil	10921	Sao Bento Do Sul	0.08	0.40	0.55	59000	32552	20229
BRA	Brazil	10922	Sao Bernardino	0.27	0.67	0.93	1702	419	0
BRA	Brazil	10923	Sao Bonifacio	0.08	0.42	0.59	2106	1115	622
BRA	Brazil	10924	Sao Carlos	1.00	1.00	1.00	0	0	0
BRA	Brazil	10925	Sao Cristovao Do Sul	0.18	0.54	0.73	3371	1418	401
BRA	Brazil	10926	Sao Domingos	0.17	0.49	0.65	6145	3003	1466
BRA	Brazil	10927	Sao Francisco Do Sul	0.08	0.46	0.69	46337	21871	7371
BRA	Brazil	10928	Sao Joao Batista	0.08	0.40	0.55	26007	14349	8917
BRA	Brazil	10929	Sao Joao Do Itaperiu	0.08	0.40	0.55	2670	1473	915
BRA	Brazil	10930	Sao Joao Do Oeste	0.17	0.49	0.65	3974	1942	948
BRA	Brazil	10931	Sao Joao Do Sul	0.08	0.42	0.59	5336	2809	1552
BRA	Brazil	10932	Sao Joaquim	0.08	0.40	0.55	19117	10547	6555
BRA	Brazil	10933	Sao Jose	0.08	0.43	0.61	175780	90408	46607
BRA	Brazil	10934	Sao Jose Do Cedro	0.58	0.84	0.98	3033	0	0
BRA	Brazil	10935	Sao Jose Do Cerrito	0.81	0.90	0.96	0	0	0

BRA	Brazil	10936	Sao Lourenco Do Oeste	0.17	0.49	0.65	14232	6954	3395
BRA	Brazil	10937	Sao Ludgero	0.08	0.40	0.55	10316	5692	3537
BRA	Brazil	10938	Sao Martinho	0.08	0.40	0.55	2495	1376	855
BRA	Brazil	10939	Sao Miguel D'oeste	0.17	0.49	0.65	25021	12226	5969
BRA	Brazil	10940	Sao Miguel Da Boa Vista	0.59	0.82	0.95	408	0	0
BRA	Brazil	10941	Sao Pedro De Alcantara	0.08	0.42	0.58	4283	2271	1314
BRA	Brazil	10942	Saudades	0.08	0.40	0.55	6882	3797	2360
BRA	Brazil	10943	Schroeder	0.08	0.43	0.62	13091	6644	3300
BRA	Brazil	10944	Seara	0.21	0.67	0.89	10427	2262	0
BRA	Brazil	10945	Serra Alta	0.08	0.40	0.55	2485	1371	852
BRA	Brazil	10946	Sideropolis	0.08	0.40	0.55	10452	5767	3584
BRA	Brazil	10947	Sombrio	0.08	0.43	0.62	21500	10892	5341
BRA	Brazil	10948	Sul Brasil	0.08	0.40	0.55	1746	963	599
BRA	Brazil	10949	Taio	0.08	0.42	0.60	13578	7111	3846
BRA	Brazil	10950	Tangara	0.08	0.40	0.55	11275	6220	3866
BRA	Brazil	10951	Tigrinhos	0.08	0.40	0.55	1265	698	434
BRA	Brazil	10952	Tijucas	0.08	0.40	0.55	25381	14003	8702
BRA	Brazil	10953	Timbe Do Sul	0.08	0.40	0.55	3910	2157	1341
BRA	Brazil	10954	Timbo	0.08	0.43	0.62	31278	15858	7844
BRA	Brazil	10955	Timbo Grande	0.23	0.59	0.78	4224	1548	127
BRA	Brazil	10956	Tres Barras	0.08	0.40	0.55	13355	7369	4579
BRA	Brazil	10957	Treviso	0.08	0.40	0.55	2773	1530	951
BRA	Brazil	10958	Treze De Maio	0.08	0.41	0.58	5598	2990	1710
BRA	Brazil	10959	Treze Tilias	0.08	0.40	0.55	5309	2929	1820
BRA	Brazil	10960	Trombudo Central	0.08	0.40	0.55	5041	2781	1728
BRA	Brazil	10961	Tubarao	0.08	0.43	0.62	75717	38652	19235
BRA	Brazil	10962	Tunapolis	0.17	0.49	0.65	3012	1472	719
BRA	Brazil	10963	Turvo	0.08	0.40	0.55	9315	5139	3194
BRA	Brazil	10964	Uniao Do Oeste	0.22	0.65	0.88	1561	413	0
BRA	Brazil	10965	Urubici	0.16	0.51	0.69	7256	3276	1265
BRA	Brazil	10966	Urupema	0.08	0.40	0.55	1695	935	581
BRA	Brazil	10967	Urussanga	0.08	0.44	0.63	15028	7551	3512
BRA	Brazil	10968	Vargeao	0.25	0.70	0.94	1853	326	0

BRA	Brazil	10969	Vargem	0.42	0.82	0.96	1099	0	0
BRA	Brazil	10970	Vargem Bonita	0.08	0.40	0.55	3471	1915	1190
BRA	Brazil	10971	Vidal Ramos	0.08	0.43	0.62	4718	2398	1158
BRA	Brazil	10972	Videira	0.08	0.40	0.55	37486	20682	12853
BRA	Brazil	10973	Vitor Meireles	0.08	0.40	0.55	4394	2424	1507
BRA	Brazil	10974	Witmarsum	0.08	0.43	0.61	2479	1280	661
BRA	Brazil	10975	Xanxere	0.17	0.58	0.83	31372	10967	0
BRA	Brazil	10976	Xavantina	0.36	0.72	0.94	1809	315	0
BRA	Brazil	10977	Xaxim	0.18	0.67	0.90	17596	3800	0
BRA	Brazil	10978	Zortea	0.17	0.49	0.65	2105	1028	502
BRA	Brazil	10979	Adamantina	0.31	0.79	0.97	16787	393	0
BRA	Brazil	10980	Adolfo	0.72	0.90	0.98	303	0	0
BRA	Brazil	10981	Aguai	0.09	0.47	0.68	25236	11715	4408
BRA	Brazil	10982	Aguas Da Prata	0.17	0.49	0.65	5016	2451	1197
BRA	Brazil	10983	Aguas De Lindoia	0.17	0.52	0.70	11592	5243	1843
BRA	Brazil	10984	Aguas De Santa Barbara	0.17	0.49	0.65	3761	1838	897
BRA	Brazil	10985	Aguas De Sao Pedro	0.09	0.55	0.77	1361	482	60
BRA	Brazil	10986	Agudos	0.17	0.49	0.65	23174	11324	5529
BRA	Brazil	10987	Alambari	0.55	0.82	0.97	1453	0	0
BRA	Brazil	10988	Alfredo Marcondes	0.40	0.87	0.97	1634	0	0
BRA	Brazil	10989	Altair	0.39	0.83	0.98	1681	0	0
BRA	Brazil	10990	Altinopolis	0.35	0.77	0.98	7231	455	0
BRA	Brazil	10991	Alto Alegre	0.17	0.49	0.65	2613	1277	623
BRA	Brazil	10992	Aluminio	0.21	0.59	0.83	10437	3671	0
BRA	Brazil	10993	Alvares Florence	0.49	0.85	0.97	1190	0	0
BRA	Brazil	10994	Alvares Machado	0.36	0.82	1.00	11064	0	0
BRA	Brazil	10995	Alvaro De Carvalho	0.31	0.70	0.93	2456	513	0
BRA	Brazil	10996	Alvinlandia	0.08	0.40	0.55	2293	1265	786
BRA	Brazil	10997	Americana	0.08	0.44	0.63	156982	78483	37317
BRA	Brazil	10998	Americo Brasiliense	0.72	0.90	1.00	3031	0	0
BRA	Brazil	10999	Americo De Campos	0.17	0.49	0.65	3739	1827	892
BRA	Brazil	11000	Amparo	0.08	0.40	0.55	51124	28207	17529
BRA	Brazil	11001	Analandia	0.13	0.53	0.78	3278	1320	76

BRA	Brazil	11002	Andradina	0.38	0.82	0.99	24294	0	0
BRA	Brazil	11003	Angatuba	0.95	0.98	1.00	0	0	0
BRA	Brazil	11004	Anhembi	0.67	0.86	1.00	829	0	0
BRA	Brazil	11005	Anhumas	0.17	0.49	0.65	2576	1259	614
BRA	Brazil	11006	Aparecida	0.08	0.40	0.55	26246	14481	8999
BRA	Brazil	11007	Aparecida D'oeste	0.42	0.86	0.97	1638	0	0
BRA	Brazil	11008	Apiai	0.17	0.49	0.65	15747	7695	3757
BRA	Brazil	11009	Aracariguama	0.14	0.51	0.76	14361	6286	917
BRA	Brazil	11010	Aracatuba	0.17	0.49	0.65	121954	59591	29095
BRA	Brazil	11011	Aracoiaba Da Serra	0.25	0.57	0.81	17898	7335	0
BRA	Brazil	11012	Aramina	0.30	0.71	0.96	2755	491	0
BRA	Brazil	11013	Arandu	0.17	0.49	0.65	4078	1993	973
BRA	Brazil	11014	Arapei	0.08	0.40	0.55	1732	956	594
BRA	Brazil	11015	Araraquara	0.17	0.49	0.65	147343	71998	35153
BRA	Brazil	11016	Araras	0.08	0.46	0.67	94450	45075	17267
BRA	Brazil	11017	Arco-iris	0.37	0.85	0.99	801	0	0
BRA	Brazil	11018	Arealva	0.17	0.49	0.65	5283	2582	1260
BRA	Brazil	11019	Areias	0.08	0.40	0.55	2788	1537	954
BRA	Brazil	11020	Areiopolis	0.80	0.92	1.00	0	0	0
BRA	Brazil	11021	Ariranha	0.17	0.49	0.65	5949	2907	1419
BRA	Brazil	11022	Artur Nogueira	0.08	0.40	0.55	38058	20997	13049
BRA	Brazil	11023	Aruja	0.08	0.43	0.61	66835	34241	17710
BRA	Brazil	11024	Aspasia	0.37	0.81	0.96	793	0	0
BRA	Brazil	11025	Assis	0.08	0.40	0.55	73983	40818	25366
BRA	Brazil	11026	Atibaia	0.08	0.43	0.61	100099	51006	25874
BRA	Brazil	11027	Auriflama	0.38	0.75	0.95	6378	804	0
BRA	Brazil	11028	Avai	0.41	0.78	0.99	2082	81	0
BRA	Brazil	11029	Avanhandava	0.30	0.72	0.95	6683	1034	0
BRA	Brazil	11030	Avare	0.94	0.96	0.99	0	0	0
BRA	Brazil	11031	Bady Bassitt	0.53	0.83	0.97	4513	0	0
BRA	Brazil	11032	Balbinos	0.25	0.62	0.85	3656	1176	0
BRA	Brazil	11033	Balsamo	0.17	0.49	0.65	5597	2735	1335
BRA	Brazil	11034	Bananal	0.08	0.42	0.59	7824	4115	2255

BRA	Brazil	11035	Barao De Antonina	0.75	0.89	0.97	177	0	0
BRA	Brazil	11036	Barbosa	0.50	0.86	0.99	2196	0	0
BRA	Brazil	11037	Bariri	0.31	0.72	0.96	17013	2657	0
BRA	Brazil	11038	Barra Bonita	0.17	0.49	0.65	23058	11267	5501
BRA	Brazil	11039	Barra Do Chapeu	0.17	0.49	0.65	3523	1722	841
BRA	Brazil	11040	Barra Do Turvo	0.10	0.45	0.63	5161	2554	1237
BRA	Brazil	11041	Barretos	0.33	0.78	0.98	56981	1939	0
BRA	Brazil	11042	Barrinha	0.17	0.49	0.65	19845	9697	4734
BRA	Brazil	11043	Barueri	0.08	0.45	0.66	188822	90955	37473
BRA	Brazil	11044	Bastos	0.39	0.87	1.00	8634	0	0
BRA	Brazil	11045	Batatais	0.33	0.75	0.98	28903	2879	0
BRA	Brazil	11046	Bauru	0.28	0.70	0.95	192009	36330	0
BRA	Brazil	11047	Bebedouro	0.37	0.79	0.98	33206	1018	0
BRA	Brazil	11048	Bento De Abreu	0.41	0.82	0.98	1145	0	0
BRA	Brazil	11049	Bernardino De Campos	0.17	0.49	0.65	7029	3435	1677
BRA	Brazil	11050	Bertioga	0.08	0.42	0.59	44418	23330	13058
BRA	Brazil	11051	Bilac	0.41	0.80	0.97	3067	33	0
BRA	Brazil	11052	Birigui	0.17	0.49	0.65	74425	36367	17756
BRA	Brazil	11053	Biritiba-mirim	0.08	0.42	0.58	22976	12186	6905
BRA	Brazil	11054	Boa Esperanca Do Sul	0.30	0.76	0.98	7370	529	0
BRA	Brazil	11055	Bocaina	0.17	0.49	0.65	7579	3704	1808
BRA	Brazil	11056	Bofete	0.17	0.49	0.65	7039	3439	1679
BRA	Brazil	11057	Boituva	0.08	0.40	0.55	42937	23689	14722
BRA	Brazil	11058	Bom Jesus Dos Perdoes	0.08	0.42	0.59	17888	9389	5196
BRA	Brazil	11059	Bom Sucesso De Itarare	0.64	0.83	0.99	624	0	0
BRA	Brazil	11060	Bora	0.82	0.90	0.95	0	0	0
BRA	Brazil	11061	Boraceia	0.46	0.84	0.99	1603	0	0
BRA	Brazil	11062	Borborema	0.17	0.49	0.65	9925	4850	2368
BRA	Brazil	11063	Borebi	0.57	0.80	0.97	582	3	0
BRA	Brazil	11064	Botucatu	0.17	0.49	0.65	89704	43833	21401
BRA	Brazil	11065	Braganca Paulista	0.08	0.43	0.60	118631	61491	32688
BRA	Brazil	11066	Brauna	0.42	0.87	0.99	2112	0	0
BRA	Brazil	11067	Brejo Alegre	0.78	0.90	0.97	62	0	0

BRA	Brazil	11068	Brodosqui	0.36	0.75	0.98	10618	1140	0
BRA	Brazil	11069	Brotas	0.56	0.81	0.97	5835	0	0
BRA	Brazil	11070	Buri	0.96	0.98	1.00	0	0	0
BRA	Brazil	11071	Buritama	0.33	0.77	0.98	7859	428	0
BRA	Brazil	11072	Buritizal	0.31	0.79	0.97	2130	62	0
BRA	Brazil	11073	Cabralia Paulista	0.17	0.49	0.65	2757	1347	658
BRA	Brazil	11074	Cabreuva	0.08	0.40	0.55	34714	19152	11902
BRA	Brazil	11075	Cacapava	0.08	0.40	0.55	66792	36851	22901
BRA	Brazil	11076	Cachoeira Paulista	0.17	0.51	0.68	20529	9569	3890
BRA	Brazil	11077	Caconde	0.33	0.65	0.89	8975	2893	0
BRA	Brazil	11078	Cafelandia	0.17	0.49	0.65	11069	5409	2641
BRA	Brazil	11079	Caiabu	0.17	0.49	0.65	2660	1300	635
BRA	Brazil	11080	Caieiras	0.08	0.42	0.58	65939	35229	20343
BRA	Brazil	11081	Caiua	0.17	0.49	0.65	3539	1729	844
BRA	Brazil	11082	Cajamar	0.08	0.40	0.55	48665	26850	16686
BRA	Brazil	11083	Cajati	0.08	0.41	0.58	20679	11050	6369
BRA	Brazil	11084	Cajobi	0.33	0.81	0.97	4833	0	0
BRA	Brazil	11085	Cajuru	0.32	0.75	0.99	12311	1153	0
BRA	Brazil	11086	Campina Do Monte Alegre	0.17	0.49	0.65	3563	1741	850
BRA	Brazil	11087	Campinas	0.08	0.40	0.55	859465	474189	294682
BRA	Brazil	11088	Campo Limpo Paulista	0.08	0.40	0.55	59494	32825	20399
BRA	Brazil	11089	Campos Do Jordao	0.17	0.52	0.70	32184	14559	5099
BRA	Brazil	11090	Campos Novos Paulista	0.08	0.40	0.55	3525	1945	1209
BRA	Brazil	11091	Cananeia	0.08	0.40	0.55	9196	5074	3153
BRA	Brazil	11092	Canas	0.08	0.40	0.55	3540	1953	1214
BRA	Brazil	11093	Candido Mota	0.22	0.69	0.92	18171	3295	0
BRA	Brazil	11094	Candido Rodrigues	0.38	0.74	0.94	1158	166	0
BRA	Brazil	11095	Canitar	0.55	0.82	0.99	1286	0	0
BRA	Brazil	11096	Capao Bonito	0.17	0.49	0.65	30007	14663	7159
BRA	Brazil	11097	Capela Do Alto	0.08	0.40	0.55	14435	7964	4949
BRA	Brazil	11098	Capivari	0.08	0.40	0.55	38429	21202	13176
BRA	Brazil	11099	Caraguatatuba	0.08	0.40	0.55	83151	45877	28510
BRA	Brazil	11100	Carapicuiba	0.08	0.42	0.58	277892	146639	83113

BRA	Brazil	11101	Cardoso	0.40	0.80	0.98	4949	0	0
BRA	Brazil	11102	Casa Branca	0.10	0.49	0.70	20936	9316	3124
BRA	Brazil	11103	Cassia Dos Coqueiros	0.53	0.87	0.99	713	0	0
BRA	Brazil	11104	Castilho	0.38	0.86	0.99	8510	0	0
BRA	Brazil	11105	Catanduva	0.41	0.81	0.98	47436	0	0
BRA	Brazil	11106	Catigua	0.43	0.83	0.98	2857	0	0
BRA	Brazil	11107	Cedral	0.17	0.49	0.65	5679	2775	1355
BRA	Brazil	11108	Cerqueira Cesar	0.17	0.49	0.65	12251	5986	2923
BRA	Brazil	11109	Cerquilho	0.08	0.47	0.70	33919	15484	4920
BRA	Brazil	11110	Cesario Lange	0.08	0.40	0.55	12554	6927	4304
BRA	Brazil	11111	Charqueada	0.08	0.43	0.61	11846	6084	3151
BRA	Brazil	11112	Chavantes	0.93	0.97	1.00	0	0	0
BRA	Brazil	11113	Clementina	0.39	0.87	0.99	3423	0	0
BRA	Brazil	11114	Colina	0.34	0.83	0.99	8423	0	0
BRA	Brazil	11115	Colombia	0.17	0.49	0.65	3868	1890	923
BRA	Brazil	11116	Conchal	0.09	0.47	0.70	19549	8999	2788
BRA	Brazil	11117	Conchas	0.08	0.40	0.55	12676	6994	4346
BRA	Brazil	11118	Cordeiropolis	0.11	0.49	0.72	16396	7285	1795
BRA	Brazil	11119	Coroados	0.29	0.68	0.92	4536	1019	0
BRA	Brazil	11120	Coronel Macedo	0.17	0.49	0.65	3158	1543	754
BRA	Brazil	11121	Corumbatai	0.08	0.40	0.55	2941	1623	1008
BRA	Brazil	11122	Cosmopolis	0.08	0.44	0.62	50836	25648	12399
BRA	Brazil	11123	Cosmorama	0.17	0.49	0.65	4619	2257	1102
BRA	Brazil	11124	Cotia	0.08	0.42	0.59	179296	93805	51861
BRA	Brazil	11125	Cravinhos	0.68	0.89	1.00	4181	0	0
BRA	Brazil	11126	Cristais Paulista	0.31	0.77	0.99	4576	260	0
BRA	Brazil	11127	Cruzalia	0.17	0.49	0.65	1379	674	329
BRA	Brazil	11128	Cruzeiro	0.17	0.49	0.65	50952	24897	12156
BRA	Brazil	11129	Cubatao	0.08	0.40	0.55	90003	49657	30859
BRA	Brazil	11130	Cunha	0.08	0.40	0.56	15794	8664	5307
BRA	Brazil	11131	Descalvado	0.17	0.49	0.65	20947	10236	4997
BRA	Brazil	11132	Diadema	0.08	0.42	0.59	293329	153284	84313
BRA	Brazil	11133	Dirce Reis	0.50	0.83	0.96	514	0	0

BRA	Brazil	11134	Divinolandia	0.37	0.64	0.83	4813	1830	0
BRA	Brazil	11135	Dobrada	0.39	0.80	1.00	3624	2	0
BRA	Brazil	11136	Dois Corregos	0.38	0.78	0.99	11265	656	0
BRA	Brazil	11137	Dolcinopolis	0.47	0.83	0.95	718	0	0
BRA	Brazil	11138	Dourado	0.34	0.68	0.91	4077	1074	0
BRA	Brazil	11139	Dracena	0.34	0.78	0.97	21293	851	0
BRA	Brazil	11140	Duartina	0.39	0.76	0.97	5182	529	0
BRA	Brazil	11141	Dumont	0.34	0.80	0.98	4418	23	0
BRA	Brazil	11142	Echapora	0.08	0.40	0.55	4527	2498	1552
BRA	Brazil	11143	Eldorado	0.17	0.49	0.65	9741	4760	2324
BRA	Brazil	11144	Elias Fausto	0.08	0.43	0.60	12669	6555	3441
BRA	Brazil	11145	Elisiario	0.52	0.85	0.98	1006	0	0
BRA	Brazil	11146	Embauba	0.17	0.49	0.65	1558	761	372
BRA	Brazil	11147	Embu	0.08	0.40	0.55	199162	109883	68286
BRA	Brazil	11148	Embu-guacu	0.08	0.42	0.58	48154	25579	14635
BRA	Brazil	11149	Emilianopolis	0.17	0.49	0.65	2008	981	479
BRA	Brazil	11150	Engenheiro Coelho	0.11	0.52	0.76	13846	5567	863
BRA	Brazil	11151	Espirito Santo Do Pinhal	0.20	0.59	0.83	26588	9342	0
BRA	Brazil	11152	Espirito Santo Do Turvo	0.55	0.88	1.00	1167	0	0
BRA	Brazil	11153	Estiva Gerbi	0.09	0.46	0.68	7711	3617	1328
BRA	Brazil	11154	Estrela D'oeste	0.50	0.84	0.97	2529	0	0
BRA	Brazil	11155	Estrela Do Norte	0.17	0.49	0.65	1738	849	415
BRA	Brazil	11156	Euclides Da Cunha Paulista	0.34	0.82	0.99	4394	0	0
BRA	Brazil	11157	Fartura	0.95	0.97	0.99	0	0	0
BRA	Brazil	11158	Fernando Prestes	0.56	0.85	0.98	1334	0	0
BRA	Brazil	11159	Fernandopolis	0.41	0.80	0.96	26993	0	0
BRA	Brazil	11160	Fernao	0.17	0.49	0.65	1047	512	250
BRA	Brazil	11161	Ferraz De Vasconcelos	0.08	0.42	0.59	130588	68750	38488
BRA	Brazil	11162	Flora Rica	0.36	0.81	0.96	712	0	0
BRA	Brazil	11163	Floreal	0.36	0.73	0.93	1330	202	0
BRA	Brazil	11164	Florida Paulista	0.32	0.79	0.96	6886	94	0
BRA	Brazil	11165	Florinia	0.32	0.74	0.93	1333	174	0
BRA	Brazil	11166	Franca	0.17	0.49	0.65	217963	106505	52001

BRA	Brazil	11167	Francisco Morato	0.08	0.40	0.55	122612	67648	42040
BRA	Brazil	11168	Franco Da Rocha	0.08	0.43	0.61	113045	58017	29619
BRA	Brazil	11169	Gabriel Monteiro	0.42	0.85	0.99	1045	0	0
BRA	Brazil	11170	Galia	0.40	0.79	0.96	2774	35	0
BRA	Brazil	11171	Garca	0.34	0.76	0.98	20434	1833	0
BRA	Brazil	11172	Gastao Vidigal	0.51	0.86	0.98	1357	0	0
BRA	Brazil	11173	Gaviao Peixoto	0.26	0.74	0.93	2509	284	0
BRA	Brazil	11174	General Salgado	0.45	0.83	0.97	3775	0	0
BRA	Brazil	11175	Getulina	0.42	0.78	0.95	4321	196	0
BRA	Brazil	11176	Glicerio	0.60	0.87	0.98	959	0	0
BRA	Brazil	11177	Guaicara	0.39	0.79	0.98	4757	81	0
BRA	Brazil	11178	Guaimbe	0.70	0.89	0.99	593	0	0
BRA	Brazil	11179	Guaira	0.17	0.49	0.65	25296	12361	6035
BRA	Brazil	11180	Guapiacu	0.34	0.77	0.98	9373	655	0
BRA	Brazil	11181	Guapiara	0.99	0.99	1.00	0	0	0
BRA	Brazil	11182	Guara	0.33	0.80	0.99	9871	0	0
BRA	Brazil	11183	Guaracai	0.40	0.82	0.96	3404	0	0
BRA	Brazil	11184	Guaraci	0.17	0.49	0.65	6875	3360	1640
BRA	Brazil	11185	Guarani D'oeste	0.34	0.79	0.96	938	12	0
BRA	Brazil	11186	Guaranta	0.55	0.83	0.97	1637	0	0
BRA	Brazil	11187	Guararapes	0.17	0.49	0.65	20496	10015	4890
BRA	Brazil	11188	Guararema	0.08	0.40	0.55	20851	11504	7149
BRA	Brazil	11189	Guaratingueta	0.17	0.55	0.79	75493	29373	700
BRA	Brazil	11190	Guarei	0.82	0.89	0.95	0	0	0
BRA	Brazil	11191	Guariba	0.17	0.49	0.65	24675	12057	5887
BRA	Brazil	11192	Guaruja	0.08	0.40	0.55	223505	123313	76632
BRA	Brazil	11193	Guarulhos	0.08	0.45	0.65	981658	480276	202561
BRA	Brazil	11194	Guatapara	0.31	0.73	0.99	3606	538	0
BRA	Brazil	11195	Guzolandia	0.42	0.79	0.98	2004	68	0
BRA	Brazil	11196	Herculandia	0.17	0.49	0.65	5909	2887	1410
BRA	Brazil	11197	Holambra	0.08	0.40	0.55	9502	5242	3258
BRA	Brazil	11198	Hortolandia	0.08	0.42	0.60	151567	78888	43006
BRA	Brazil	11199	Iacanga	0.28	0.70	0.95	5941	1110	0

BRA	Brazil	11200	Iacri	0.34	0.81	0.97	2977	0	0
BRA	Brazil	11201	Iaras	0.53	0.77	0.94	2617	272	0
BRA	Brazil	11202	Ibate	0.93	0.96	0.99	0	0	0
BRA	Brazil	11203	Ibira	0.52	0.85	0.99	3431	0	0
BRA	Brazil	11204	Ibirarema	0.35	0.68	0.88	3404	886	0
BRA	Brazil	11205	Ibitinga	0.08	0.40	0.55	42296	23336	14502
BRA	Brazil	11206	Ibiuna	0.55	0.75	0.90	19152	4115	0
BRA	Brazil	11207	Icem	0.38	0.79	0.98	3415	73	0
BRA	Brazil	11208	Iepe	0.37	0.84	0.99	3484	0	0
BRA	Brazil	11209	Igaracu Do Tiete	0.37	0.75	0.97	10378	1212	0
BRA	Brazil	11210	Igarapava	0.17	0.49	0.65	19005	9287	4534
BRA	Brazil	11211	Igarata	0.17	0.49	0.65	5906	2886	1409
BRA	Brazil	11212	Iguape	0.08	0.40	0.55	22048	12165	7560
BRA	Brazil	11213	Ilha Comprida	0.08	0.43	0.62	7472	3798	1888
BRA	Brazil	11214	Ilha Solteira	0.17	0.49	0.65	16666	8144	3976
BRA	Brazil	11215	Ilhabela	0.08	0.40	0.55	23045	12714	7901
BRA	Brazil	11216	Indaiatuba	0.08	0.43	0.61	175859	90425	46988
BRA	Brazil	11217	Indiana	0.37	0.83	0.98	2115	0	0
BRA	Brazil	11218	Indiapora	0.48	0.87	0.98	1252	0	0
BRA	Brazil	11219	Inubia Paulista	0.36	0.83	0.98	1712	0	0
BRA	Brazil	11220	Ipaucu	0.17	0.49	0.65	9304	4546	2220
BRA	Brazil	11221	Ipero	0.08	0.40	0.55	25388	14007	8705
BRA	Brazil	11222	Ipeuna	0.08	0.46	0.67	5261	2485	936
BRA	Brazil	11223	Ipigua	0.17	0.49	0.65	3226	1577	770
BRA	Brazil	11224	Iporanga	0.17	0.52	0.71	2825	1265	417
BRA	Brazil	11225	Ipua	0.17	0.49	0.65	10045	4908	2396
BRA	Brazil	11226	Iracemapolis	0.08	0.46	0.67	16894	7938	2973
BRA	Brazil	11227	Irapua	0.55	0.85	0.99	2000	0	0
BRA	Brazil	11228	Irapuru	0.75	0.91	0.97	388	0	0
BRA	Brazil	11229	Itabera	0.95	0.96	0.96	0	0	0
BRA	Brazil	11230	Itai	0.91	0.96	1.00	0	0	0
BRA	Brazil	11231	Itajobi	0.46	0.80	0.96	5214	37	0
BRA	Brazil	11232	Itaju	0.17	0.49	0.65	2349	1148	560

BRA	Brazil	11233	Itanhaem	0.08	0.42	0.58	70952	37644	21429
BRA	Brazil	11234	Itaoca	0.08	0.40	0.55	2520	1390	864
BRA	Brazil	11235	Itapecerica Da Serra	0.08	0.42	0.58	128784	68117	38372
BRA	Brazil	11236	Itapetininga	0.67	0.84	0.96	20962	0	0
BRA	Brazil	11237	Itapeva	0.17	0.49	0.65	58646	28656	13991
BRA	Brazil	11238	Itapevi	0.08	0.41	0.57	173541	93691	55782
BRA	Brazil	11239	Itapira	0.18	0.55	0.78	46115	18222	1556
BRA	Brazil	11240	Itapirapua Paulista	0.39	0.69	0.93	1725	459	0
BRA	Brazil	11241	Itapolis	0.36	0.73	0.96	18778	2862	0
BRA	Brazil	11242	Itaporanga	0.17	0.49	0.65	9504	4644	2267
BRA	Brazil	11243	Itapui	0.30	0.72	0.97	6850	1127	0
BRA	Brazil	11244	Itapura	0.17	0.49	0.65	3027	1479	722
BRA	Brazil	11245	Itaquaquecetuba	0.08	0.41	0.57	249677	133657	77880
BRA	Brazil	11246	Itarare	0.17	0.49	0.65	31644	15462	7549
BRA	Brazil	11247	Itariri	0.08	0.40	0.55	13972	7709	4790
BRA	Brazil	11248	Itatiba	0.08	0.44	0.62	84508	42705	21043
BRA	Brazil	11249	Itatinga	0.17	0.49	0.65	12717	6214	3034
BRA	Brazil	11250	Itirapina	0.09	0.52	0.76	12501	4925	624
BRA	Brazil	11251	Itirapua	0.35	0.78	0.99	2875	140	0
BRA	Brazil	11252	Itobi	0.08	0.40	0.55	5689	3139	1950
BRA	Brazil	11253	Itu	0.08	0.40	0.55	122472	67571	41992
BRA	Brazil	11254	Itupeva	0.08	0.42	0.59	43951	22962	12520
BRA	Brazil	11255	Ituverava	0.35	0.77	0.98	18378	1080	0
BRA	Brazil	11256	Jaborandi	0.17	0.49	0.65	4347	2124	1037
BRA	Brazil	11257	Jaboticabal	0.17	0.49	0.65	48331	23617	11531
BRA	Brazil	11258	Jacarei	0.08	0.43	0.60	167201	86592	46115
BRA	Brazil	11259	Jaci	0.17	0.49	0.65	4303	2102	1026
BRA	Brazil	11260	Jacupiranga	0.08	0.40	0.55	12995	7170	4455
BRA	Brazil	11261	Jaguariuna	0.08	0.43	0.61	40897	20848	10599
BRA	Brazil	11262	Jales	0.17	0.49	0.65	30803	15052	7349
BRA	Brazil	11263	Jambeiro	0.08	0.40	0.55	4647	2564	1593
BRA	Brazil	11264	Jandira	0.08	0.42	0.58	91603	48591	27734
BRA	Brazil	11265	Jardinopolis	0.17	0.49	0.65	27459	13418	6551

BRA	Brazil	11266	Jarinu	0.08	0.42	0.60	21180	11047	6004
BRA	Brazil	11267	Jau	0.26	0.66	0.91	78757	20824	0
BRA	Brazil	11268	Jeriquara	0.17	0.49	0.65	2018	986	482
BRA	Brazil	11269	Joanopolis	0.17	0.51	0.69	8224	3785	1476
BRA	Brazil	11270	Joao Ramalho	0.59	0.89	0.99	972	0	0
BRA	Brazil	11271	Jose Bonifacio	0.70	0.86	0.97	3725	0	0
BRA	Brazil	11272	Julio Mesquita	0.17	0.49	0.65	2972	1452	709
BRA	Brazil	11273	Jumirim	0.08	0.40	0.55	2390	1319	820
BRA	Brazil	11274	Jundiai	0.08	0.44	0.64	296764	146541	66011
BRA	Brazil	11275	Junqueiropolis	0.17	0.49	0.65	12785	6247	3050
BRA	Brazil	11276	Juquia	0.08	0.40	0.55	14211	7841	4873
BRA	Brazil	11277	Juquitiba	0.08	0.40	0.55	22449	12386	7697
BRA	Brazil	11278	Lagoinha	0.08	0.43	0.61	3561	1833	949
BRA	Brazil	11279	Laranjal Paulista	0.75	0.89	1.00	1384	0	0
BRA	Brazil	11280	Lavinia	0.17	0.49	0.65	7903	3862	1885
BRA	Brazil	11281	Lavrinhas	0.17	0.49	0.65	4898	2393	1169
BRA	Brazil	11282	Leme	0.10	0.50	0.73	70741	29801	6961
BRA	Brazil	11283	Lencois Paulista	0.17	0.49	0.65	42223	20632	10073
BRA	Brazil	11284	Limeira	0.08	0.40	0.55	217587	120048	74604
BRA	Brazil	11285	Lindoia	0.17	0.52	0.71	4912	2203	736
BRA	Brazil	11286	Lins	0.17	0.49	0.65	48449	23674	11559
BRA	Brazil	11287	Lorena	0.08	0.43	0.61	63276	32672	16981
BRA	Brazil	11288	Lourdes	0.17	0.49	0.65	1423	695	340
BRA	Brazil	11289	Louveira	0.08	0.43	0.60	33172	17249	9253
BRA	Brazil	11290	Lucelia	0.34	0.81	0.99	9962	0	0
BRA	Brazil	11291	Lucianopolis	0.44	0.79	0.96	854	29	0
BRA	Brazil	11292	Luis Antonio	0.48	0.82	0.99	4649	0	0
BRA	Brazil	11293	Luiziania	0.37	0.82	0.99	2442	0	0
BRA	Brazil	11294	Lupercio	0.35	0.77	0.96	2042	152	0
BRA	Brazil	11295	Lutecia	0.40	0.76	0.90	1097	122	0
BRA	Brazil	11296	Macatuba	0.45	0.78	0.98	5925	380	0
BRA	Brazil	11297	Macaubal	0.45	0.78	0.96	2854	130	0
BRA	Brazil	11298	Macedonia	0.47	0.79	0.97	1218	19	0

BRA	Brazil	11299	Magda	0.17	0.49	0.65	2020	987	482
BRA	Brazil	11300	Mairinque	0.08	0.40	0.55	33784	18639	11583
BRA	Brazil	11301	Mairipora	0.08	0.41	0.58	68902	36883	21399
BRA	Brazil	11302	Manduri	0.87	0.93	0.98	0	0	0
BRA	Brazil	11303	Maraba Paulista	0.31	0.73	0.93	2781	422	0
BRA	Brazil	11304	Maracai	0.32	0.77	0.98	6708	352	0
BRA	Brazil	11305	Marapoama	0.54	0.82	0.95	761	0	0
BRA	Brazil	11306	Mariapolis	0.33	0.82	0.97	1900	0	0
BRA	Brazil	11307	Marilia	0.29	0.71	0.95	120645	20566	0
BRA	Brazil	11308	Marinopolis	0.59	0.88	0.96	445	0	0
BRA	Brazil	11309	Martinopolis	0.38	0.82	0.97	10963	0	0
BRA	Brazil	11310	Matao	0.17	0.49	0.65	51682	25254	12330
BRA	Brazil	11311	Maua	0.08	0.42	0.59	332917	174697	96736
BRA	Brazil	11312	Mendonca	0.73	0.90	0.98	346	0	0
BRA	Brazil	11313	Meridiano	0.51	0.86	0.98	1140	0	0
BRA	Brazil	11314	Mesopolis	0.63	0.88	0.98	325	0	0
BRA	Brazil	11315	Miguelopolis	0.40	0.78	0.98	8724	525	0
BRA	Brazil	11316	Mineiros Do Tiete	0.17	0.49	0.65	8038	3928	1918
BRA	Brazil	11317	Mira Estrela	0.45	0.82	0.96	1055	0	0
BRA	Brazil	11318	Miracatu	0.08	0.43	0.60	14287	7323	3882
BRA	Brazil	11319	Mirandopolis	0.43	0.85	0.98	10426	0	0
BRA	Brazil	11320	Mirante Do Paranapanema	0.36	0.77	0.97	7915	477	0
BRA	Brazil	11321	Mirassol	0.38	0.76	0.97	24688	2107	0
BRA	Brazil	11322	Mirassolandia	0.35	0.78	0.98	2124	115	0
BRA	Brazil	11323	Mococa	0.17	0.49	0.65	43332	21174	10338
BRA	Brazil	11324	Moji Das Cruzes	0.08	0.43	0.60	332551	171723	90566
BRA	Brazil	11325	Moji-guacu	0.10	0.49	0.71	102238	45882	12725
BRA	Brazil	11326	Moji-mirim	0.08	0.40	0.55	68533	37811	23498
BRA	Brazil	11327	Mombuca	0.17	0.51	0.75	2189	999	171
BRA	Brazil	11328	Moncoes	0.17	0.49	0.65	1415	691	338
BRA	Brazil	11329	Mongagua	0.08	0.40	0.55	39566	21829	13566
BRA	Brazil	11330	Monte Alegre Do Sul	0.08	0.42	0.58	5546	2947	1673
BRA	Brazil	11331	Monte Alto	0.37	0.78	0.97	21543	748	0

BRA	Brazil	11332	Monte Aprazivel	0.38	0.80	0.97	10290	118	0
BRA	Brazil	11333	Monte Azul Paulista	0.35	0.77	0.97	8586	507	0
BRA	Brazil	11334	Monte Castelo	0.17	0.49	0.65	2639	1289	630
BRA	Brazil	11335	Monte Mor	0.08	0.42	0.58	45615	24197	13716
BRA	Brazil	11336	Monteiro Lobato	0.17	0.49	0.65	2924	1429	698
BRA	Brazil	11337	Morro Agudo	0.17	0.49	0.65	20278	9909	4838
BRA	Brazil	11338	Morungaba	0.08	0.42	0.58	9835	5217	2959
BRA	Brazil	11339	Motuca	0.25	0.64	0.90	2555	743	0
BRA	Brazil	11340	Murutinga Do Sul	0.37	0.79	0.96	1881	36	0
BRA	Brazil	11341	Nantes	0.40	0.86	0.99	1213	0	0
BRA	Brazil	11342	Narandiba	0.17	0.49	0.65	2963	1448	707
BRA	Brazil	11343	Natividade Da Serra	0.08	0.40	0.55	4867	2685	1669
BRA	Brazil	11344	Nazare Paulista	0.08	0.41	0.57	12904	6966	4127
BRA	Brazil	11345	Neves Paulista	0.35	0.79	0.95	4103	79	0
BRA	Brazil	11346	Nhandeara	0.32	0.74	0.94	5371	689	0
BRA	Brazil	11347	Nipoa	0.33	0.83	0.99	2398	0	0
BRA	Brazil	11348	Nova Alianca	0.51	0.82	0.96	1972	0	0
BRA	Brazil	11349	Nova Campina	0.84	0.92	0.99	0	0	0
BRA	Brazil	11350	Nova Canaa Paulista	0.38	0.79	0.95	842	20	0
BRA	Brazil	11351	Nova Castilho	0.33	0.71	0.95	582	111	0
BRA	Brazil	11352	Nova Europa	0.41	0.80	0.97	4187	29	0
BRA	Brazil	11353	Nova Granada	0.38	0.79	0.97	8850	164	0
BRA	Brazil	11354	Nova Guataporanga	0.37	0.77	0.97	969	75	0
BRA	Brazil	11355	Nova Independencia	0.40	0.86	0.98	1550	0	0
BRA	Brazil	11356	Nova Luzitania	0.17	0.49	0.65	2492	1218	594
BRA	Brazil	11357	Nova Odessa	0.08	0.42	0.59	48534	25570	14257
BRA	Brazil	11358	Novais	0.46	0.86	0.99	1918	0	0
BRA	Brazil	11359	Novo Horizonte	0.17	0.49	0.65	25287	12356	6033
BRA	Brazil	11360	Nuporanga	0.33	0.80	0.97	3427	0	0
BRA	Brazil	11361	Ocaucu	0.42	0.80	0.98	1625	0	0
BRA	Brazil	11362	Oleo	0.97	0.98	0.99	0	0	0
BRA	Brazil	11363	Olimpia	0.36	0.80	0.98	23567	90	0
BRA	Brazil	11364	Onda Verde	0.34	0.80	0.98	2000	4	0

BRA	Brazil	11365	Oriente	0.38	0.77	0.97	2691	166	0
BRA	Brazil	11366	Orindiuva	0.37	0.80	0.98	2909	0	0
BRA	Brazil	11367	Orlandia	0.17	0.49	0.65	27204	13293	6490
BRA	Brazil	11368	Osasco	0.08	0.42	0.59	498136	260342	142027
BRA	Brazil	11369	Oscar Bressane	0.29	0.71	0.90	1332	240	0
BRA	Brazil	11370	Osvaldo Cruz	0.32	0.81	0.97	15640	0	0
BRA	Brazil	11371	Ourinhos	0.29	0.72	0.97	56641	9397	0
BRA	Brazil	11372	Ouro Verde	0.17	0.49	0.65	5293	2586	1263
BRA	Brazil	11373	Ouroeste	0.35	0.80	0.98	4476	0	0
BRA	Brazil	11374	Pacaembu	0.31	0.77	0.95	6812	358	0
BRA	Brazil	11375	Palestina	0.17	0.49	0.65	7906	3863	1886
BRA	Brazil	11376	Palmares Paulista	0.38	0.85	0.99	5213	0	0
BRA	Brazil	11377	Palmeira D'oeste	0.38	0.78	0.95	3982	191	0
BRA	Brazil	11378	Palmital	0.08	0.40	0.55	15999	8827	5485
BRA	Brazil	11379	Panorama	0.42	0.84	1.00	5855	0	0
BRA	Brazil	11380	Paraguacu Paulista	0.17	0.49	0.65	28379	13867	6771
BRA	Brazil	11381	Paraibuna	0.08	0.42	0.59	13084	6871	3767
BRA	Brazil	11382	Paraiso	0.17	0.49	0.65	4288	2095	1023
BRA	Brazil	11383	Paranapanema	0.98	0.99	1.00	0	0	0
BRA	Brazil	11384	Paranapua	0.43	0.84	0.98	1492	0	0
BRA	Brazil	11385	Parapua	0.30	0.79	0.97	5536	75	0
BRA	Brazil	11386	Pardinho	0.17	0.49	0.65	3944	1927	941
BRA	Brazil	11387	Pariquera-acu	0.08	0.43	0.61	13895	7065	3653
BRA	Brazil	11388	Parisi	0.48	0.88	0.99	681	0	0
BRA	Brazil	11389	Patrocinio Paulista	0.32	0.74	0.99	6932	814	0
BRA	Brazil	11390	Pauliceia	0.17	0.49	0.65	4497	2198	1073
BRA	Brazil	11391	Paulinia	0.08	0.43	0.62	76029	38624	19291
BRA	Brazil	11392	Paulistania	0.61	0.83	0.98	346	0	0
BRA	Brazil	11393	Paulo De Faria	0.48	0.80	0.98	2893	15	0
BRA	Brazil	11394	Pederneiras	0.17	0.49	0.65	28831	14088	6878
BRA	Brazil	11395	Pedra Bela	0.17	0.50	0.66	4115	1982	919
BRA	Brazil	11396	Pedranopolis	0.35	0.78	0.94	1152	54	0
BRA	Brazil	11397	Pedregulho	0.17	0.49	0.65	10394	5079	2480

BRA	Brazil	11398	Pedreira	0.08	0.42	0.58	33479	17859	10239
BRA	Brazil	11399	Pedrinhas Paulista	0.17	0.49	0.65	1935	945	462
BRA	Brazil	11400	Pedro De Toledo	0.08	0.42	0.59	8077	4241	2328
BRA	Brazil	11401	Penapolis	0.31	0.76	0.98	30609	2219	0
BRA	Brazil	11402	Pereira Barreto	0.35	0.79	0.98	11592	295	0
BRA	Brazil	11403	Pereiras	0.08	0.40	0.55	6154	3395	2110
BRA	Brazil	11404	Peruibe	0.08	0.42	0.59	46207	24224	13308
BRA	Brazil	11405	Piacatu	0.17	0.49	0.65	3671	1794	876
BRA	Brazil	11406	Piedade	0.08	0.40	0.55	39889	22008	13677
BRA	Brazil	11407	Pilar Do Sul	0.17	0.49	0.65	18046	8818	4305
BRA	Brazil	11408	Pindamonhangaba	0.17	0.52	0.70	102289	46048	15783
BRA	Brazil	11409	Pindorama	0.17	0.49	0.65	10336	5051	2466
BRA	Brazil	11410	Pinhalzinho	0.08	0.40	0.55	10710	5909	3672
BRA	Brazil	11411	Piquerobi	0.17	0.49	0.65	2322	1135	554
BRA	Brazil	11412	Piquete	0.17	0.49	0.65	8894	4346	2122
BRA	Brazil	11413	Piracaia	0.17	0.49	0.65	16944	8280	4042
BRA	Brazil	11414	Piracicaba	0.08	0.46	0.67	284384	134288	52111
BRA	Brazil	11415	Piraju	0.96	0.98	0.99	0	0	0
BRA	Brazil	11416	Pirajui	0.32	0.76	0.95	12049	934	0
BRA	Brazil	11417	Pirangi	0.17	0.49	0.65	7110	3474	1696
BRA	Brazil	11418	Pirapora Do Bom Jesus	0.08	0.43	0.61	13467	6874	3563
BRA	Brazil	11419	Pirapozinho	0.17	0.49	0.65	17086	8349	4076
BRA	Brazil	11420	Pirassununga	0.20	0.62	0.85	45335	13680	0
BRA	Brazil	11421	Piratininga	0.31	0.74	0.96	6575	831	0
BRA	Brazil	11422	Pitangueiras	0.17	0.49	0.65	24427	11936	5828
BRA	Brazil	11423	Planalto	0.17	0.49	0.65	3209	1568	766
BRA	Brazil	11424	Platina	0.08	0.40	0.55	2514	1387	862
BRA	Brazil	11425	Poa	0.08	0.43	0.61	84057	42909	22176
BRA	Brazil	11426	Poloni	0.53	0.86	0.98	1579	0	0
BRA	Brazil	11427	Pompeia	0.29	0.73	0.95	11054	1516	0
BRA	Brazil	11428	Pongai	0.53	0.81	0.97	942	0	0
BRA	Brazil	11429	Pontal	0.38	0.79	0.99	20441	570	0
BRA	Brazil	11430	Pontalinda	0.58	0.85	0.98	1014	0	0

BRA	Brazil	11431	Pontes Gestal	0.34	0.80	0.99	1206	0	0
BRA	Brazil	11432	Populina	0.35	0.84	0.98	1885	0	0
BRA	Brazil	11433	Porangaba	0.17	0.49	0.65	6032	2947	1439
BRA	Brazil	11434	Porto Feliz	0.08	0.45	0.64	37522	18514	8514
BRA	Brazil	11435	Porto Ferreira	0.17	0.49	0.65	34794	17001	8301
BRA	Brazil	11436	Potim	0.08	0.40	0.55	17010	9383	5829
BRA	Brazil	11437	Potirendaba	0.54	0.83	0.98	4427	0	0
BRA	Brazil	11438	Pracinha	0.31	0.70	0.90	2060	416	0
BRA	Brazil	11439	Pradopolis	0.17	0.49	0.65	13075	6389	3119
BRA	Brazil	11440	Praia Grande	0.08	0.40	0.55	225863	124615	77441
BRA	Brazil	11441	Pratania	0.90	0.96	1.00	0	0	0
BRA	Brazil	11442	Presidente Alves	0.59	0.81	0.97	865	0	0
BRA	Brazil	11443	Presidente Bernardes	0.17	0.49	0.65	8485	4146	2024
BRA	Brazil	11444	Presidente Epitacio	0.37	0.81	1.00	18912	0	0
BRA	Brazil	11445	Presidente Prudente	0.77	0.92	0.99	6325	0	0
BRA	Brazil	11446	Presidente Venceslau	0.35	0.79	0.98	17635	440	0
BRA	Brazil	11447	Promissao	0.32	0.77	0.97	18736	1175	0
BRA	Brazil	11448	Quadra	0.96	0.97	0.98	0	0	0
BRA	Brazil	11449	Quata	0.47	0.83	0.98	4528	0	0
BRA	Brazil	11450	Queiroz	0.17	0.49	0.65	2075	1014	495
BRA	Brazil	11451	Queluz	0.17	0.50	0.67	8165	3858	1660
BRA	Brazil	11452	Quintana	0.17	0.49	0.65	4103	2005	979
BRA	Brazil	11453	Rafard	0.10	0.49	0.72	7028	3095	766
BRA	Brazil	11454	Rancharia	0.17	0.49	0.65	18744	9159	4472
BRA	Brazil	11455	Redencao Da Serra	0.08	0.41	0.57	2886	1558	919
BRA	Brazil	11456	Regente Feijo	0.41	0.81	0.98	7767	0	0
BRA	Brazil	11457	Reginopolis	0.17	0.49	0.65	5912	2889	1410
BRA	Brazil	11458	Registro	0.08	0.43	0.60	40613	21050	11263
BRA	Brazil	11459	Restinga	0.33	0.75	0.98	3491	401	0
BRA	Brazil	11460	Ribeira	0.73	0.86	0.98	266	0	0
BRA	Brazil	11461	Ribeirao Bonito	0.22	0.66	0.92	7583	1884	0
BRA	Brazil	11462	Ribeirao Branco	0.99	0.99	1.00	0	0	0
BRA	Brazil	11463	Ribeirao Corrente	0.35	0.83	0.99	2107	0	0

BRA	Brazil	11464	Ribeirao Do Sul	0.62	0.83	0.97	742	0	0
BRA	Brazil	11465	Ribeirao Dos Indios	0.18	0.54	0.75	13017	5424	1085
BRA	Brazil	11466	Ribeirao Grande	0.17	0.49	0.65	4727	2310	1128
BRA	Brazil	11467	Ribeirao Pires	0.08	0.42	0.59	101615	53272	29227
BRA	Brazil	11468	Ribeirao Preto	0.34	0.78	0.98	312129	15200	0
BRA	Brazil	11469	Rifaina	0.40	0.80	0.99	1450	3	0
BRA	Brazil	11470	Rincao	0.76	0.90	0.99	400	0	0
BRA	Brazil	11471	Rinopolis	0.17	0.49	0.65	6363	3109	1518
BRA	Brazil	11472	Rio Claro	0.08	0.40	0.55	148317	81830	50853
BRA	Brazil	11473	Rio Das Pedras	0.08	0.40	0.55	25375	14000	8700
BRA	Brazil	11474	Rio Grande Da Serra	0.08	0.42	0.59	33874	17765	9972
BRA	Brazil	11475	Riolandia	0.43	0.79	0.98	4528	168	0
BRA	Brazil	11476	Riversul	0.78	0.89	0.99	119	0	0
BRA	Brazil	11477	Rosana	0.17	0.49	0.65	11543	5640	2754
BRA	Brazil	11478	Roseira	0.08	0.40	0.55	7721	4255	2638
BRA	Brazil	11479	Rubiacea	0.35	0.79	0.98	1369	18	0
BRA	Brazil	11480	Rubineia	0.35	0.77	0.95	1388	83	0
BRA	Brazil	11481	Sabino	0.28	0.67	0.94	2889	704	0
BRA	Brazil	11482	Sagres	0.30	0.79	0.97	1224	22	0
BRA	Brazil	11483	Sales	0.89	0.95	0.98	0	0	0
BRA	Brazil	11484	Sales Oliveira	0.17	0.49	0.65	7303	3568	1742
BRA	Brazil	11485	Salesopolis	0.08	0.43	0.61	11993	6115	3079
BRA	Brazil	11486	Salmourao	0.17	0.49	0.65	3274	1600	781
BRA	Brazil	11487	Saltinho	0.08	0.40	0.55	5127	2829	1758
BRA	Brazil	11488	Salto	0.08	0.40	0.55	83574	46110	28655
BRA	Brazil	11489	Salto De Pirapora	0.19	0.54	0.78	26836	11360	1026
BRA	Brazil	11490	Salto Grande	0.49	0.78	0.97	2884	147	0
BRA	Brazil	11491	Sandovalina	0.47	0.85	0.99	1386	0	0
BRA	Brazil	11492	Santa Adelia	0.39	0.80	0.97	6310	50	0
BRA	Brazil	11493	Santa Albertina	0.17	0.49	0.65	3770	1842	899
BRA	Brazil	11494	Santa Barbara D'oeste	0.08	0.40	0.55	145102	80057	49751
BRA	Brazil	11495	Santa Branca	0.08	0.43	0.61	10515	5419	2813
BRA	Brazil	11496	Santa Clara D'oeste	0.17	0.49	0.65	1347	658	321

BRA	Brazil	11497	Santa Cruz Da Conceicao	0.66	0.76	0.85	618	153	0
BRA	Brazil	11498	Santa Cruz Da Esperanca	0.41	0.78	0.98	849	54	0
BRA	Brazil	11499	Santa Cruz Das Palmeiras	0.19	0.67	0.87	20561	4269	0
BRA	Brazil	11500	Santa Cruz Do Rio Pardo	0.36	0.76	0.98	20798	2046	0
BRA	Brazil	11501	Santa Ernestina	0.46	0.76	0.97	1899	202	0
BRA	Brazil	11502	Santa Fe Do Sul	0.17	0.49	0.65	19962	9754	4762
BRA	Brazil	11503	Santa Gertrudes	0.08	0.40	0.55	16682	9204	5720
BRA	Brazil	11504	Santa Isabel	0.08	0.40	0.55	40931	22582	14034
BRA	Brazil	11505	Santa Lucia	0.17	0.49	0.65	5511	2693	1315
BRA	Brazil	11506	Santa Maria Da Serra	0.10	0.52	0.76	4219	1680	248
BRA	Brazil	11507	Santa Mercedes	0.47	0.88	1.00	975	0	0
BRA	Brazil	11508	Santa Rita D'oeste	0.37	0.82	0.95	1099	0	0
BRA	Brazil	11509	Santa Rita Do Passa Quatro	0.76	0.86	0.95	1162	0	0
BRA	Brazil	11510	Santa Rosa De Viterbo	0.38	0.81	0.99	10993	0	0
BRA	Brazil	11511	Santa Salete	0.47	0.85	0.96	498	0	0
BRA	Brazil	11512	Santana Da Ponte Pensa	0.17	0.49	0.65	1003	490	239
BRA	Brazil	11513	Santana De Parnaiba	0.08	0.42	0.59	107285	55883	30525
BRA	Brazil	11514	Santo Anastacio	0.17	0.49	0.65	1417	692	338
BRA	Brazil	11515	Santo Andre	0.08	0.40	0.55	519157	286432	178002
BRA	Brazil	11516	Santo Antonio Da Alegria	0.17	0.49	0.65	4242	2073	1012
BRA	Brazil	11517	Santo Antonio De Posse	0.08	0.43	0.60	16561	8600	4609
BRA	Brazil	11518	Santo Antonio Do Aracangua	0.39	0.80	0.99	3423	38	0
BRA	Brazil	11519	Santo Antonio Do Jardim	0.17	0.49	0.65	3803	1858	907
BRA	Brazil	11520	Santo Antonio Do Pinhal	0.17	0.49	0.65	4195	2050	1001
BRA	Brazil	11521	Santo Expedito	0.40	0.82	0.98	1214	0	0
BRA	Brazil	11522	Santopolis Do Aguapei	0.17	0.49	0.65	2948	1441	703
BRA	Brazil	11523	Santos	0.08	0.40	0.55	306400	169049	105054
BRA	Brazil	11524	Sao Bento Do Sapucai	0.17	0.49	0.65	6885	3364	1643
BRA	Brazil	11525	Sao Bernardo Do Campo	0.08	0.40	0.55	600491	331306	205889
BRA	Brazil	11526	Sao Caetano Do Sul	0.08	0.51	0.75	119691	48923	8592
BRA	Brazil	11527	Sao Carlos	0.17	0.49	0.65	154767	75625	36924
BRA	Brazil	11528	Sao Francisco	0.17	0.49	0.65	1778	869	424
BRA	Brazil	11529	Sao Joao Da Boa Vista	0.18	0.58	0.82	55831	20116	0

BRA	Brazil	11530	Sao Joao Das Duas Pontes	0.70	0.87	0.96	260	0	0
BRA	Brazil	11531	Sao Joao De Iracema	0.37	0.87	0.98	808	0	0
BRA	Brazil	11532	Sao Joao Do Pau D'alho	0.35	0.83	0.98	948	0	0
BRA	Brazil	11533	Sao Joaquim Da Barra	0.34	0.77	0.98	23513	1283	0
BRA	Brazil	11534	Sao Jose Da Bela Vista	0.17	0.49	0.65	5592	2732	1334
BRA	Brazil	11535	Sao Jose Do Barreiro	0.08	0.40	0.55	3058	1685	1044
BRA	Brazil	11536	Sao Jose Do Rio Pardo	0.17	0.54	0.74	34113	14417	3383
BRA	Brazil	11537	Sao Jose Do Rio Preto	0.17	0.49	0.65	283233	138399	67573
BRA	Brazil	11538	Sao Jose Dos Campos	0.17	0.49	0.65	440218	215108	105025
BRA	Brazil	11539	Sao Lourenco Da Serra	0.08	0.40	0.55	11229	6196	3850
BRA	Brazil	11540	Sao Luis Do Paraitinga	0.08	0.42	0.59	7708	4072	2266
BRA	Brazil	11541	Sao Manuel	0.52	0.78	0.97	11523	811	0
BRA	Brazil	11542	Sao Miguel Arcanjo	0.17	0.49	0.65	20571	10052	4908
BRA	Brazil	11543	Sao Paulo	0.08	0.40	0.55	8666559	4781559	2971477
BRA	Brazil	11544	Sao Pedro	0.08	0.40	0.55	26156	14431	8968
BRA	Brazil	11545	Sao Pedro Do Turvo	0.08	0.40	0.55	5620	3100	1927
BRA	Brazil	11546	Sao Roque	0.08	0.40	0.55	65137	35937	22333
BRA	Brazil	11547	Sao Sebastiao	0.08	0.40	0.55	61872	34137	21214
BRA	Brazil	11548	Sao Sebastiao Da Grama	0.34	0.62	0.83	5671	2166	0
BRA	Brazil	11549	Sao Simao	0.17	0.49	0.65	9560	4671	2281
BRA	Brazil	11550	Sao Vicente	0.08	0.40	0.55	268340	148050	92005
BRA	Brazil	11551	Sarapui	0.77	0.88	0.98	344	0	0
BRA	Brazil	11552	Sarutaia	0.99	0.99	0.99	0	0	0
BRA	Brazil	11553	Sebastianopolis Do Sul	0.45	0.81	0.96	1224	0	0
BRA	Brazil	11554	Serra Azul	0.29	0.72	0.96	7152	1056	0
BRA	Brazil	11555	Serra Negra	0.17	0.51	0.69	18066	8280	3125
BRA	Brazil	11556	Serrana	0.17	0.49	0.65	27673	13522	6602
BRA	Brazil	11557	Sertaozinho	0.17	0.49	0.65	77317	37780	18446
BRA	Brazil	11558	Sete Barras	0.17	0.51	0.68	8179	3810	1559
BRA	Brazil	11559	Severinia	0.17	0.49	0.65	10801	5278	2577
BRA	Brazil	11560	Silveiras	0.08	0.40	0.55	4471	2467	1533
BRA	Brazil	11561	Socorro	0.17	0.49	0.65	25245	12336	6023
BRA	Brazil	11562	Sorocaba	0.08	0.46	0.66	477143	228055	92397

BRA	Brazil	11563	Sud Menucci	0.17	0.49	0.65	4807	2349	1147
BRA	Brazil	11564	Sumare	0.08	0.40	0.55	192258	106074	65919
BRA	Brazil	11565	Suzanapolis	0.37	0.77	0.97	1637	109	0
BRA	Brazil	11566	Suzano	0.08	0.43	0.60	193838	100627	54050
BRA	Brazil	11567	Tabapua	0.75	0.90	0.99	651	0	0
BRA	Brazil	11568	Tabatinga	0.17	0.49	0.65	10152	4961	2422
BRA	Brazil	11569	Taboao Da Serra	0.08	0.41	0.57	198771	106727	62624
BRA	Brazil	11570	Taciba	0.40	0.85	0.99	2472	0	0
BRA	Brazil	11571	Taguai	0.86	0.93	1.00	0	0	0
BRA	Brazil	11572	Taiacu	0.30	0.74	0.98	3123	353	0
BRA	Brazil	11573	Taiuva	0.38	0.81	0.96	2326	0	0
BRA	Brazil	11574	Tambau	0.24	0.60	0.85	13088	4613	0
BRA	Brazil	11575	Tanabi	0.17	0.49	0.65	16162	7897	3856
BRA	Brazil	11576	Tapirai	0.17	0.49	0.65	5052	2468	1205
BRA	Brazil	11577	Tapiratiba	0.27	0.62	0.86	6876	2355	0
BRA	Brazil	11578	Taquaral	0.35	0.81	0.98	1270	0	0
BRA	Brazil	11579	Taquaritinga	0.38	0.77	0.99	23933	1679	0
BRA	Brazil	11580	Taquarituba	0.93	0.97	1.00	0	0	0
BRA	Brazil	11581	Taquarivai	0.96	0.98	0.99	0	0	0
BRA	Brazil	11582	Tarabai	0.42	0.84	0.99	2755	0	0
BRA	Brazil	11583	Taruma	0.38	0.77	0.97	6145	463	0
BRA	Brazil	11584	Tatui	0.17	0.49	0.65	75373	36830	17982
BRA	Brazil	11585	Taubate	0.08	0.43	0.62	220912	112199	55475
BRA	Brazil	11586	Tejupa	1.00	1.00	1.00	0	0	0
BRA	Brazil	11587	Teodoro Sampaio	0.17	0.49	0.65	14375	7024	3429
BRA	Brazil	11588	Terra Roxa	0.17	0.49	0.65	5794	2831	1382
BRA	Brazil	11589	Tiete	0.09	0.50	0.74	29195	12180	2417
BRA	Brazil	11590	Timburi	0.96	0.97	0.98	0	0	0
BRA	Brazil	11591	Torre De Pedra	0.81	0.92	1.00	0	0	0
BRA	Brazil	11592	Torrinha	0.29	0.69	0.91	5019	1132	0
BRA	Brazil	11593	Trabiju	0.08	0.40	0.55	1251	690	429
BRA	Brazil	11594	Tremembe	0.08	0.40	0.55	34615	19098	11868
BRA	Brazil	11595	Tres Fronteiras	0.17	0.49	0.65	3627	1772	865

BRA	Brazil	11596	Tuiuti	0.08	0.41	0.58	4839	2590	1504
BRA	Brazil	11597	Tupa	0.38	0.81	0.97	27852	0	0
BRA	Brazil	11598	Tupi Paulista	0.30	0.81	0.95	7593	0	0
BRA	Brazil	11599	Turiuba	0.42	0.80	0.94	764	5	0
BRA	Brazil	11600	Turmalina	0.17	0.49	0.65	1203	588	287
BRA	Brazil	11601	Ubarana	0.17	0.49	0.65	3857	1885	920
BRA	Brazil	11602	Ubatuba	0.08	0.42	0.59	63199	33026	18118
BRA	Brazil	11603	Ubirajara	0.38	0.76	0.98	1979	192	0
BRA	Brazil	11604	Uchoa	0.39	0.82	0.99	4145	0	0
BRA	Brazil	11605	Uniao Paulista	0.45	0.89	1.00	624	0	0
BRA	Brazil	11606	Urania	0.38	0.81	0.97	3824	0	0
BRA	Brazil	11607	Uru	0.75	0.89	0.97	61	0	0
BRA	Brazil	11608	Urupes	0.51	0.82	0.96	3911	0	0
BRA	Brazil	11609	Valentim Gentil	0.17	0.49	0.65	8117	3966	1937
BRA	Brazil	11610	Valinhos	0.08	0.44	0.63	91292	45778	22118
BRA	Brazil	11611	Valparaiso	0.17	0.49	0.65	16200	7916	3865
BRA	Brazil	11612	Vargem	0.17	0.49	0.65	6501	3177	1551
BRA	Brazil	11613	Vargem Grande Do Sul	0.08	0.40	0.55	30423	16785	10431
BRA	Brazil	11614	Vargem Grande Paulista	0.08	0.40	0.55	28010	15454	9604
BRA	Brazil	11615	Varzea Paulista	0.08	0.42	0.59	84166	44419	25010
BRA	Brazil	11616	Vera Cruz	0.08	0.40	0.55	8057	4445	2762
BRA	Brazil	11617	Vinhedo	0.08	0.45	0.65	55081	26622	11189
BRA	Brazil	11618	Viradouro	0.36	0.83	1.00	8134	0	0
BRA	Brazil	11619	Vista Alegre Do Alto	0.47	0.83	0.97	2826	0	0
BRA	Brazil	11620	Vitoria Brasil	0.17	0.49	0.65	1147	561	274
BRA	Brazil	11621	Votorantim	0.08	0.43	0.60	84649	43777	23238
BRA	Brazil	11622	Votuporanga	0.32	0.79	0.98	44261	613	0
BRA	Brazil	11623	Zacarias	0.44	0.81	0.97	959	0	0
BRA	Brazil	11624	Amparo De Sao Francisco	0.08	0.40	0.55	1480	816	507
BRA	Brazil	11625	Aquidaba	0.08	0.40	0.55	16087	8876	5516
BRA	Brazil	11626	Aracaju	0.08	0.40	0.55	470346	259502	161266
BRA	Brazil	11627	Araua	0.08	0.40	0.55	7421	4094	2544
BRA	Brazil	11628	Areia Branca	0.08	0.40	0.55	12687	7000	4350

BRA	Brazil	11629	Barra Dos Coqueiros	0.08	0.40	0.55	21223	11709	7277
BRA	Brazil	11630	Boquim	0.08	0.40	0.55	20580	11354	7056
BRA	Brazil	11631	Brejo Grande	0.08	0.40	0.55	6032	3328	2068
BRA	Brazil	11632	Campo Do Brito	0.08	0.40	0.55	13433	7412	4606
BRA	Brazil	11634	Canhoba	0.08	0.40	0.55	2794	1542	958
BRA	Brazil	11635	Caninde De Sao Francisco	0.08	0.40	0.55	21609	11922	7409
BRA	Brazil	11636	Capela	0.08	0.40	0.55	24072	13281	8254
BRA	Brazil	11637	Carira	0.08	0.40	0.55	15588	8600	5344
BRA	Brazil	11638	Carmopolis	0.08	0.40	0.55	12184	6722	4177
BRA	Brazil	11639	Cedro De Sao Joao	0.08	0.40	0.55	4301	2373	1475
BRA	Brazil	11640	Cristinapolis	0.08	0.40	0.55	13579	7492	4656
BRA	Brazil	11641	Cumbe	0.08	0.40	0.55	3049	1682	1045
BRA	Brazil	11642	Divina Pastora	0.08	0.40	0.55	4307	2377	1477
BRA	Brazil	11643	Estancia	0.08	0.40	0.55	49713	27428	17045
BRA	Brazil	11644	Feira Nova	0.08	0.40	0.55	4090	2257	1402
BRA	Brazil	11645	Frei Paulo	0.08	0.40	0.55	11241	6202	3854
BRA	Brazil	11646	Gararu	0.08	0.40	0.55	8313	4587	2850
BRA	Brazil	11647	General Maynard	0.08	0.40	0.55	2139	1180	733
BRA	Brazil	11648	Gracho Cardoso	0.08	0.40	0.55	4169	2300	1429
BRA	Brazil	11649	Ilha Das Flores	0.08	0.40	0.55	6102	3367	2092
BRA	Brazil	11650	Indiaroba	0.08	0.40	0.55	12696	7005	4353
BRA	Brazil	11651	Itabaiana	0.08	0.40	0.55	68719	37914	23562
BRA	Brazil	11652	Itabaianinha	0.08	0.40	0.55	29833	16460	10229
BRA	Brazil	11653	Itabi	0.08	0.40	0.55	4014	2214	1376
BRA	Brazil	11654	Itaporanga D'ajuda	0.08	0.40	0.55	25196	13901	8639
BRA	Brazil	11655	Japaratuba	0.08	0.40	0.55	14012	7731	4804
BRA	Brazil	11656	Japoata	0.08	0.40	0.55	9653	5326	3310
BRA	Brazil	11657	Lagarto	0.08	0.40	0.55	75158	41467	25769
BRA	Brazil	11658	Laranjeiras	0.08	0.40	0.55	21463	11842	7359
BRA	Brazil	11659	Macambira	0.08	0.40	0.55	4990	2753	1711
BRA	Brazil	11660	Malhada Dos Bois	0.08	0.40	0.55	1977	1091	678
BRA	Brazil	11661	Malhador	0.08	0.40	0.55	9102	5022	3121
BRA	Brazil	11662	Maruim	0.08	0.40	0.55	12602	6953	4321

BRA	Brazil	11663	Moita Bonita	0.08	0.40	0.55	8231	4541	2822
BRA	Brazil	11664	Monte Alegre De Sergipe	0.08	0.40	0.55	11291	6230	3871
BRA	Brazil	11665	Muribeca	0.08	0.40	0.55	5560	3068	1906
BRA	Brazil	11666	Neopolis	0.08	0.40	0.55	13143	7251	4506
BRA	Brazil	11667	Nossa Senhora Aparecida	0.08	0.40	0.55	6602	3642	2263
BRA	Brazil	11668	Nossa Senhora Da Gloria	0.08	0.40	0.55	26190	14450	8980
BRA	Brazil	11669	Nossa Senhora Das Dores	0.08	0.40	0.55	19136	10558	6561
BRA	Brazil	11670	Nossa Senhora De Lourdes	0.08	0.40	0.55	4368	2410	1498
BRA	Brazil	11671	Nossa Senhora Do Socorro	0.08	0.40	0.55	134639	74284	46163
BRA	Brazil	11672	Pacatuba	0.08	0.40	0.55	10110	5578	3466
BRA	Brazil	11673	Pedra Mole	0.08	0.40	0.55	2245	1239	770
BRA	Brazil	11674	Pedrinhas	0.08	0.40	0.55	6363	3511	2182
BRA	Brazil	11675	Pinhao	0.08	0.40	0.55	4757	2625	1631
BRA	Brazil	11676	Pirambu	0.08	0.40	0.55	5687	3137	1950
BRA	Brazil	11677	Poco Redondo	0.08	0.40	0.55	25135	13868	8618
BRA	Brazil	11678	Poco Verde	0.08	0.40	0.55	15707	8666	5386
BRA	Brazil	11679	Porto Da Folha	0.08	0.40	0.55	21079	11630	7227
BRA	Brazil	11680	Propria	0.08	0.40	0.55	21011	11592	7204
BRA	Brazil	11681	Riachao Do Dantas	0.08	0.40	0.55	14262	7869	4890
BRA	Brazil	11682	Riachuelo	0.08	0.40	0.55	6839	3773	2345
BRA	Brazil	11683	Ribeiropolis	0.08	0.40	0.55	13564	7484	4651
BRA	Brazil	11684	Rosario Do Catete	0.08	0.40	0.55	7449	4110	2554
BRA	Brazil	11685	Salgado	0.08	0.40	0.55	15131	8348	5188
BRA	Brazil	11686	Santa Luzia Do Itanhy	0.08	0.40	0.55	10709	5908	3672
BRA	Brazil	11687	Santa Rosa De Lima	0.08	0.40	0.55	2858	1577	980
BRA	Brazil	11688	Santana Do Sao Francisco	0.08	0.40	0.55	5043	2782	1729
BRA	Brazil	11689	Santo Amaro Das Brotas	0.08	0.40	0.55	9039	4987	3099
BRA	Brazil	11690	Sao Cristovao	0.08	0.40	0.55	65437	36103	22436
BRA	Brazil	11691	Sao Domingos	0.08	0.40	0.55	7887	4351	2704
BRA	Brazil	11692	Sao Francisco	0.08	0.40	0.55	3703	2043	1270
BRA	Brazil	11693	Sao Miguel Do Aleixo	0.08	0.40	0.55	2859	1577	980
BRA	Brazil	11694	Simao Dias	0.08	0.40	0.55	29452	16249	10098
BRA	Brazil	11695	Siriri	0.08	0.40	0.55	7170	3956	2458

BRA	Brazil	11696	Telha	0.08	0.40	0.55	2412	1331	827
BRA	Brazil	11697	Tobias Barreto	0.08	0.40	0.55	40389	22284	13848
BRA	Brazil	11698	Tomar Do Geru	0.08	0.40	0.55	9532	5259	3268
BRA	Brazil	11699	Umbauba	0.08	0.40	0.55	17890	9870	6134
BRA	Brazil	11700	Abreulandia	0.43	0.82	1.00	971	0	0
BRA	Brazil	11701	Aguiarnopolis	0.41	0.79	0.99	2767	65	0
BRA	Brazil	11702	Alianca Do Tocantins	0.38	0.84	1.00	2389	0	0
BRA	Brazil	11703	Almas	0.39	0.82	1.00	3083	0	0
BRA	Brazil	11704	Alvorada	0.17	0.49	0.65	5407	2642	1290
BRA	Brazil	11705	Ananas	0.39	0.83	1.00	3904	0	0
BRA	Brazil	11706	Angico	0.41	0.80	1.00	1409	0	0
BRA	Brazil	11707	Aparecida Do Rio Negro	0.43	0.81	1.00	1789	0	0
BRA	Brazil	11708	Aragominas	0.50	0.84	1.00	1731	0	0
BRA	Brazil	11709	Araguacema	0.37	0.79	0.99	3025	46	0
BRA	Brazil	11710	Araguacu	0.45	0.82	0.98	3066	0	0
BRA	Brazil	11711	Araguaina	0.46	0.81	1.00	59230	0	0
BRA	Brazil	11712	Araguana	0.17	0.49	0.65	3424	1673	817
BRA	Brazil	11713	Araguatins	0.36	0.79	1.00	15966	191	0
BRA	Brazil	11714	Arapoema	0.44	0.79	1.00	2497	80	0
BRA	Brazil	11715	Arraias	0.62	0.93	1.00	1944	0	0
BRA	Brazil	11716	Augustinopolis	0.35	0.81	1.00	8224	0	0
BRA	Brazil	11717	Aurora Do Tocantins	0.50	0.81	0.98	1141	0	0
BRA	Brazil	11718	Axixa Do Tocantins	0.41	0.86	1.00	3524	0	0
BRA	Brazil	11719	Babaculandia	0.25	0.67	0.94	9489	2205	0
BRA	Brazil	11720	Bandeirantes Do Tocantins	0.40	0.77	0.98	1479	101	0
BRA	Brazil	11721	Barra Do Ouro	0.38	0.81	1.00	1941	0	0
BRA	Brazil	11722	Barrolandia	0.49	0.84	1.00	1736	0	0
BRA	Brazil	11723	Bernardo Sayao	0.40	0.84	1.00	1754	0	0
BRA	Brazil	11724	Bom Jesus Do Tocantins	0.17	0.49	0.65	3203	1565	764
BRA	Brazil	11725	Brasilandia Do Tocantins	0.48	0.87	0.99	707	0	0
BRA	Brazil	11726	Brejinho De Nazare	0.36	0.78	0.99	2466	133	0
BRA	Brazil	11727	Buriti Do Tocantins	0.34	0.80	1.00	4288	35	0
BRA	Brazil	11728	Cachoeirinha	0.17	0.49	0.65	1466	716	350

BRA	Brazil	11729	Campos Lindos	0.34	0.78	1.00	4578	231	0
BRA	Brazil	11730	Cariri Do Tocantins	0.42	0.82	0.99	1454	0	0
BRA	Brazil	11731	Carmolandia	0.39	0.77	1.00	1072	74	0
BRA	Brazil	11732	Carrasco Bonito	0.38	0.84	1.00	1387	0	0
BRA	Brazil	11733	Caseara	0.35	0.82	1.00	2306	0	0
BRA	Brazil	11734	Centenario	0.17	0.49	0.65	1772	866	423
BRA	Brazil	11735	Chapada Da Natividade	0.47	0.84	1.00	1136	0	0
BRA	Brazil	11736	Chapada De Areia	0.42	0.85	0.98	532	0	0
BRA	Brazil	11737	Colinas Do Tocantins	0.43	0.80	1.00	13020	67	0
BRA	Brazil	11738	Colmeia	0.42	0.84	1.00	3240	0	0
BRA	Brazil	11739	Combinado	0.51	0.84	1.00	1471	0	0
BRA	Brazil	11740	Conceicao Do Tocantins	0.39	0.86	1.00	1565	0	0
BRA	Brazil	11741	Couto De Magalhaes	0.35	0.78	1.00	2511	89	0
BRA	Brazil	11742	Cristalandia	0.38	0.81	1.00	3122	0	0
BRA	Brazil	11743	Crixas Do Tocantins	0.48	0.81	0.99	523	0	0
BRA	Brazil	11744	Darcinopolis	0.37	0.80	1.00	2575	30	0
BRA	Brazil	11745	Dianopolis	0.42	0.85	1.00	8535	0	0
BRA	Brazil	11746	Divinopolis Do Tocantins	0.43	0.85	1.00	2550	0	0
BRA	Brazil	11747	Dois Irmaos Do Tocantins	0.32	0.78	0.98	3548	112	0
BRA	Brazil	11748	Duere	0.40	0.80	0.99	1867	20	0
BRA	Brazil	11749	Esperantina	0.17	0.49	0.65	6853	3349	1635
BRA	Brazil	11750	Fatima	0.17	0.49	0.65	2452	1198	585
BRA	Brazil	11751	Figueiropolis	0.43	0.82	0.99	2001	0	0
BRA	Brazil	11752	Filadelfia	0.17	0.49	0.65	5915	2891	1411
BRA	Brazil	11753	Formoso Do Araguaia	0.36	0.81	1.00	8366	0	0
BRA	Brazil	11754	Fortaleza Do Tabocao	0.39	0.83	1.00	1070	0	0
BRA	Brazil	11755	Goianorte	0.35	0.83	0.98	2308	0	0
BRA	Brazil	11756	Goiatins	0.36	0.79	1.00	5666	106	0
BRA	Brazil	11757	Guarai	0.44	0.85	1.00	9155	0	0
BRA	Brazil	11758	Gurupi	0.40	0.83	1.00	34195	0	0
BRA	Brazil	11759	Ipueiras	0.17	0.49	0.65	1284	628	306
BRA	Brazil	11760	Itacaja	0.42	0.80	1.00	3018	0	0
BRA	Brazil	11761	Itaguatins	0.39	0.74	0.98	2831	408	0

BRA	Brazil	11762	Itapiratins	0.37	0.81	1.00	1566	0	0
BRA	Brazil	11763	Itapora Do Tocantins	0.46	0.86	1.00	817	0	0
BRA	Brazil	11764	Jau Do Tocantins	0.37	0.80	0.98	1648	0	0
BRA	Brazil	11765	Juarina	0.43	0.83	0.99	828	0	0
BRA	Brazil	11766	Lagoa Da Confusao	0.31	0.74	0.98	6636	823	0
BRA	Brazil	11767	Lagoa Do Tocantins	0.34	0.78	1.00	1976	76	0
BRA	Brazil	11768	Lajeado	0.17	0.49	0.65	947	463	226
BRA	Brazil	11769	Lavandeira	0.72	0.94	1.00	132	0	0
BRA	Brazil	11770	Lizarda	0.39	0.75	0.99	1605	177	0
BRA	Brazil	11771	Luzinopolis	0.38	0.79	1.00	1282	22	0
BRA	Brazil	11772	Marianopolis Do Tocantins	0.32	0.77	0.99	2464	168	0
BRA	Brazil	11773	Mateiros	0.33	0.78	1.00	1170	58	0
BRA	Brazil	11774	Maurilandia Do Tocantins	0.36	0.80	0.99	1552	0	0
BRA	Brazil	11775	Miracema Do Tocantins	0.36	0.84	1.00	8779	0	0
BRA	Brazil	11776	Miranorte	0.43	0.83	0.99	5014	0	0
BRA	Brazil	11777	Monte Do Carmo	0.31	0.76	0.99	3787	289	0
BRA	Brazil	11778	Monte Santo Do Tocantins	0.37	0.75	0.98	983	105	0
BRA	Brazil	11779	Mosquito	0.30	0.72	0.98	3345	518	0
BRA	Brazil	11780	Muricilandia	0.31	0.78	0.98	1932	97	0
BRA	Brazil	11781	Natividade	0.17	0.49	0.65	5884	2875	1404
BRA	Brazil	11782	Nazare	0.17	0.49	0.65	2588	1265	617
BRA	Brazil	11783	Nova Olinda	0.17	0.49	0.65	7374	3603	1759
BRA	Brazil	11784	Nova Rosalandia	0.39	0.83	0.99	1649	0	0
BRA	Brazil	11785	Novo Acordo	0.40	0.79	1.00	1456	54	0
BRA	Brazil	11786	Novo Alegre	0.40	0.83	0.99	853	0	0
BRA	Brazil	11787	Novo Jardim	0.38	0.82	1.00	1144	0	0
BRA	Brazil	11788	Oliveira De Fatima	0.48	0.86	1.00	361	0	0
BRA	Brazil	11789	Palmas	0.17	0.49	0.65	192283	93957	45874
BRA	Brazil	11790	Palmeirante	0.42	0.77	0.97	2285	180	0
BRA	Brazil	11791	Palmeiropolis	0.43	0.86	1.00	2858	0	0
BRA	Brazil	11792	Paraiso Do Tocantins	0.41	0.80	1.00	20130	0	0
BRA	Brazil	11793	Parana	0.17	0.49	0.65	7169	3503	1710
BRA	Brazil	11794	Pau D'arco	0.17	0.49	0.65	2945	1439	703

BRA	Brazil	11795	Pedro Afonso	0.39	0.83	1.00	5224	0	0
BRA	Brazil	11796	Peixe	0.35	0.78	0.96	5046	205	0
BRA	Brazil	11797	Pequizeiro	0.35	0.78	0.99	2469	132	0
BRA	Brazil	11798	Pindorama Do Tocantins	0.34	0.78	1.00	2109	113	0
BRA	Brazil	11799	Piraque	0.35	0.71	0.96	1469	299	0
BRA	Brazil	11800	Pium	0.38	0.80	0.99	3290	0	0
BRA	Brazil	11801	Ponte Alta Do Bom Jesus	0.42	0.81	0.99	1746	0	0
BRA	Brazil	11802	Ponte Alta Do Tocantins	0.36	0.81	1.00	3618	0	0
BRA	Brazil	11803	Porto Alegre Do Tocantins	0.42	0.82	1.00	1040	0	0
BRA	Brazil	11804	Porto Nacional	0.36	0.82	1.00	23066	0	0
BRA	Brazil	11805	Praia Norte	0.32	0.76	0.99	4115	302	0
BRA	Brazil	11806	Presidente Kennedy	0.17	0.49	0.65	2371	1158	566
BRA	Brazil	11807	Pugmil	0.42	0.82	1.00	1024	0	0
BRA	Brazil	11808	Recursolandia	0.42	0.86	1.00	1273	0	0
BRA	Brazil	11809	Riachinho	0.17	0.49	0.65	3200	1564	764
BRA	Brazil	11810	Rio Da Conceicao	0.42	0.83	1.00	796	0	0
BRA	Brazil	11811	Rio Dos Bois	0.66	0.86	0.99	399	0	0
BRA	Brazil	11812	Rio Sono	0.30	0.74	0.98	3369	382	0
BRA	Brazil	11813	Sampaio	0.40	0.77	0.99	2051	152	0
BRA	Brazil	11814	Sandolandia	0.43	0.82	0.99	1284	0	0
BRA	Brazil	11815	Santa Fe Do Araguaia	0.44	0.83	1.00	2548	0	0
BRA	Brazil	11816	Santa Maria Do Tocantins	0.36	0.83	1.00	1505	0	0
BRA	Brazil	11817	Santa Rita Do Tocantins	0.37	0.75	0.97	1050	121	0
BRA	Brazil	11818	Santa Rosa Do Tocantins	0.35	0.82	1.00	2168	0	0
BRA	Brazil	11819	Santa Tereza Do Tocantins	0.39	0.84	1.00	1158	0	0
BRA	Brazil	11820	Santa Terezinha Do Tocantins	0.40	0.83	0.99	1028	0	0
BRA	Brazil	11821	Sao Bento Do Tocantins	0.33	0.78	0.99	3133	128	0
BRA	Brazil	11822	Sao Felix Do Tocantins	0.40	0.82	1.00	605	0	0
BRA	Brazil	11823	Sao Miguel Do Tocantins	0.37	0.76	0.98	5795	572	0
BRA	Brazil	11824	Sao Salvador Do Tocantins	0.66	0.89	0.99	427	0	0
BRA	Brazil	11825	Sao Sebastiao Do Tocantins	0.39	0.84	1.00	1980	0	0
BRA	Brazil	11826	Sao Valerio Da Natividade	0.47	0.91	1.00	1339	0	0
BRA	Brazil	11827	Silvanopolis	0.37	0.79	1.00	2372	37	0

BRA	Brazil	11828	Sitio Novo Do Tocantins	0.51	0.84	1.00	2548	0	0
BRA	Brazil	11829	Sucupira	0.40	0.74	0.96	1050	162	0
BRA	Brazil	11830	Taguatinga	0.36	0.79	0.98	7367	148	0
BRA	Brazil	11831	Taipas Do Tocantins	0.40	0.82	0.99	899	0	0
BRA	Brazil	11832	Talisma	0.49	0.83	0.99	857	0	0
BRA	Brazil	11833	Tocantinia	0.36	0.81	1.00	3451	0	0
BRA	Brazil	11834	Tocantinopolis	0.38	0.81	1.00	9734	0	0
BRA	Brazil	11835	Tupirama	0.39	0.76	0.96	1048	105	0
BRA	Brazil	11836	Tupiratins	0.31	0.77	0.97	1379	95	0
BRA	Brazil	11837	Wanderlandia	0.34	0.73	0.97	5469	863	0
BRA	Brazil	11838	Xambioa	0.48	0.84	1.00	3859	0	0
CAF	Central African Republic	12835	Bamingui	0.28	0.29	0.30	4546	4388	4307
CAF	Central African Republic	12836	Ndele	0.28	0.29	0.30	23867	23036	22609
CAF	Central African Republic	12837	Alindao	0.28	0.29	0.30	44679	43124	42325
CAF	Central African Republic	12838	Kembe	0.28	0.29	0.30	38595	37252	36562
CAF	Central African Republic	12839	Mingala	0.28	0.29	0.30	16889	16301	15999
CAF	Central African Republic	12840	Mobaye	0.28	0.29	0.30	38055	36730	36050
CAF	Central African Republic	12841	Zangba	0.28	0.29	0.30	21798	21039	20649
CAF	Central African Republic	12842	Bambouti	0.28	0.29	0.30	1933	1866	1831
CAF	Central African Republic	12843	Djemah	0.28	0.29	0.30	1350	1303	1279
CAF	Central African Republic	12844	Obo	0.28	0.29	0.30	23360	22547	22129
CAF	Central African Republic	12845	Zemio	0.28	0.29	0.30	11254	10862	10661
CAF	Central African Republic	12846	Bria	0.59	0.66	0.73	18046	11854	5915
CAF	Central African Republic	12847	Ouadda	0.59	0.66	0.73	4783	3142	1568
CAF	Central African Republic	12848	Yalinga	0.59	0.66	0.73	1369	899	449
CAF	Central African Republic	12849	Dekoa	0.28	0.29	0.30	23763	22936	22511
CAF	Central African Republic	12850	Mala	0.28	0.29	0.30	8780	8475	8317
CAF	Central African Republic	12851	Ndjoukou	0.28	0.29	0.30	18782	18128	17792
CAF	Central African Republic	12852	Sibut	0.28	0.29	0.30	26529	25606	25131
CAF	Central African Republic	12853	Boda	0.60	0.67	0.74	6188	4019	1916
CAF	Central African Republic	12854	Boganangone	0.60	0.67	0.74	6857	4454	2123
CAF	Central African Republic	12855	Boganda	0.60	0.67	0.74	3494	2269	1082
CAF	Central African Republic	12856	Mbaiki	0.60	0.67	0.74	39577	25704	12255

CAF	Central African Republic	12857	Mongoumba	0.60	0.67	0.74	4869	3162	1508
CAF	Central African Republic	12858	Amada Gaza	0.28	0.29	0.30	9704	9367	9193
CAF	Central African Republic	12859	Berberati	0.28	0.29	0.30	92793	89564	87903
CAF	Central African Republic	12860	Carnot	0.28	0.29	0.30	58521	56484	55437
CAF	Central African Republic	12861	Dede Mokouba	0.28	0.29	0.30	11116	10729	10530
CAF	Central African Republic	12862	Gadzi	0.28	0.29	0.30	41211	39776	39039
CAF	Central African Republic	12863	Gamboula	0.28	0.29	0.30	17909	17286	16965
CAF	Central African Republic	12864	Sosso Nakombo	0.28	0.29	0.30	7543	7281	7146
CAF	Central African Republic	12865	Bakouma	0.28	0.29	0.30	13549	13078	12835
CAF	Central African Republic	12866	Bangassou	0.28	0.29	0.30	43365	41856	41080
CAF	Central African Republic	12867	Gambo	0.28	0.29	0.30	14040	13551	13300
CAF	Central African Republic	12868	Ouango	0.28	0.29	0.30	25752	24856	24395
CAF	Central African Republic	12869	Rafai	0.28	0.29	0.30	8970	8657	8497
CAF	Central African Republic	12870	Kaga Bandoro	0.28	0.29	0.30	63706	61489	60349
CAF	Central African Republic	12871	Mbres	0.28	0.29	0.30	13140	12683	12448
CAF	Central African Republic	12872	Abba	0.28	0.29	0.30	12949	12498	12266
CAF	Central African Republic	12873	Baboua	0.28	0.29	0.30	33171	32016	31423
CAF	Central African Republic	12874	Baoro	0.28	0.29	0.30	22093	21324	20928
CAF	Central African Republic	12875	Bouar	0.28	0.29	0.30	85403	82431	80903
CAF	Central African Republic	12876	Bimbo	0.28	0.29	0.30	426464	411623	403992
CAF	Central African Republic	12877	Boali	0.71	0.75	0.79	3297	1773	229
CAF	Central African Republic	12878	Bogangolo	0.28	0.29	0.30	6444	6220	6104
CAF	Central African Republic	12879	Bossembele	0.47	0.57	0.66	15375	10924	6835
CAF	Central African Republic	12880	Damara	0.71	0.75	0.79	3787	2036	263
CAF	Central African Republic	12881	Yaloke	0.47	0.59	0.69	19061	12434	6401
CAF	Central African Republic	12882	Bakala	0.28	0.29	0.30	5855	5651	5547
CAF	Central African Republic	12883	Bambari	0.28	0.29	0.30	77087	74404	73025
CAF	Central African Republic	12884	Grimari	0.28	0.29	0.30	25859	24959	24497
CAF	Central African Republic	12885	Ippy	0.28	0.29	0.30	27701	26737	26241
CAF	Central African Republic	12886	Kouango	0.28	0.29	0.30	45225	43651	42842
CAF	Central African Republic	12887	Bocaranga	0.28	0.29	0.30	53924	52047	51082
CAF	Central African Republic	12888	Bozoum	0.69	0.74	0.78	9880	5639	1346
CAF	Central African Republic	12889	Koui	0.28	0.29	0.30	18158	17526	17201

CAF	Central African Republic	12890	Ngaoundaye	0.28	0.29	0.30	52305	50485	49549
CAF	Central African Republic	12891	Paoua	0.28	0.29	0.30	107589	103844	101919
CAF	Central African Republic	12892	Batangafo	0.28	0.29	0.30	38617	37273	36582
CAF	Central African Republic	12893	Boguila	0.28	0.29	0.30	15477	14938	14661
CAF	Central African Republic	12894	Bossangoa	0.28	0.29	0.30	83474	80569	79075
CAF	Central African Republic	12895	Bouca	0.28	0.29	0.30	37178	35884	35219
CAF	Central African Republic	12896	Kabo	0.28	0.29	0.30	29047	28036	27517
CAF	Central African Republic	12897	Markounda	0.28	0.29	0.30	11382	10986	10783
CAF	Central African Republic	12898	Nana Bakassa	0.28	0.29	0.30	26476	25555	25081
CAF	Central African Republic	12899	Bambio	0.60	0.67	0.74	2713	1762	840
CAF	Central African Republic	12900	Bayanga	0.60	0.67	0.74	2407	1563	745
CAF	Central African Republic	12901	Nola	0.60	0.67	0.74	19874	12907	6154
CAF	Central African Republic	12902	Birao	0.28	0.29	0.30	32598	31464	30881
CAF	Central African Republic	12903	Ouanda-djalle	0.28	0.29	0.30	2517	2430	2384
CAF	Central African Republic	67155	Bangui	0.28	0.29	0.30	120959	116749	114585
CIV	CÃ´te d'Ivoire	14900	Nawa	0.37	0.39	0.39	117201	113164	111559
CIV	CÃ´te d'Ivoire	14909	Belier	0.37	0.39	0.39	66222	63942	63034
CIV	CÃ´te d'Ivoire	14910	Yamoussoukro	0.37	0.39	0.39	174085	168089	165704
CIV	CÃ´te d'Ivoire	14911	Abidjan	0.82	0.87	0.88	0	0	0
CIV	CÃ´te d'Ivoire	14928	Tchologo	0.37	0.39	0.39	168979	163159	160844
CIV	CÃ´te d'Ivoire	14929	Poro	0.76	0.79	0.81	30356	7011	0
CIV	CÃ´te d'Ivoire	14941	Bere	0.43	0.49	0.55	101182	84751	69147
CIV	CÃ´te d'Ivoire	14942	Worodougou	0.37	0.39	0.39	98111	94732	93388
CIV	CÃ´te d'Ivoire	14943	Bafing	0.37	0.39	0.39	75857	73245	72206
CIV	CÃ´te d'Ivoire	14945	Bounkani	0.53	0.60	0.68	70169	50290	31318
CIV	CÃ´te d'Ivoire	16846	Agneby-Tiassa	0.37	0.39	0.39	313253	302464	298173
CIV	CÃ´te d'Ivoire	16847	Bagoue	0.37	0.39	0.39	150832	145637	143571
CIV	CÃ´te d'Ivoire	16848	Cavally	0.37	0.39	0.39	137574	132836	130952
CIV	CÃ´te d'Ivoire	16849	Folon	0.44	0.51	0.58	33350	26598	20246
CIV	CÃ´te d'Ivoire	16850	Gbêke	0.43	0.49	0.55	480825	402744	328594
CIV	CÃ´te d'Ivoire	16851	Gbôkle	0.37	0.39	0.39	56280	54342	53571
CIV	CÃ´te d'Ivoire	16852	Gôh	0.50	0.57	0.66	247265	184737	117928
CIV	CÃ´te d'Ivoire	16853	Gontougo	0.53	0.61	0.69	197063	134866	77877

CIV	CÃ´te d'Ivoire	16854	Grands ponts	0.37	0.39	0.39	157134	151722	149570
CIV	CÃ´te d'Ivoire	16855	Guemon	0.37	0.39	0.39	232523	224515	221329
CIV	CÃ´te d'Ivoire	16856	Hambol	0.43	0.49	0.55	146047	122330	99808
CIV	CÃ´te d'Ivoire	16857	Haut-Sassandra	0.37	0.39	0.39	605514	584659	576364
CIV	CÃ´te d'Ivoire	16858	Iffou	0.37	0.39	0.39	159887	154380	152190
CIV	CÃ´te d'Ivoire	16859	Indenie-Djuablin	0.37	0.39	0.39	252859	244150	240686
CIV	CÃ´te d'Ivoire	16860	Kabadougou	0.44	0.59	0.72	83845	48056	18671
CIV	CÃ´te d'Ivoire	16861	Lôh-Djiboua	0.37	0.39	0.39	379306	366242	361046
CIV	CÃ´te d'Ivoire	16862	Marahoue	0.37	0.39	0.39	325484	314274	309815
CIV	CÃ´te d'Ivoire	16863	Me	0.37	0.39	0.39	264841	255720	252091
CIV	CÃ´te d'Ivoire	16864	N'Zi	0.37	0.39	0.39	305391	294873	290690
CIV	CÃ´te d'Ivoire	16865	San-pedro	0.37	0.39	0.39	109480	105710	104210
CIV	CÃ´te d'Ivoire	16866	Sud-comoe	0.37	0.39	0.39	341804	330032	325349
CIV	CÃ´te d'Ivoire	16867	Tonkpi	0.37	0.39	0.39	428385	413631	407762
CIV	CÃ´te d'Ivoire	16868	Belier	0.37	0.39	0.39	76190	73566	72522
CMR	Cameroon	12462	Djerem	0.86	0.90	0.97	0	0	0
CMR	Cameroon	12463	Faro - Et - Déo	0.86	0.90	0.97	0	0	0
CMR	Cameroon	12464	Mayo-Banyo	0.86	0.90	0.97	0	0	0
CMR	Cameroon	12465	Mbéré	0.86	0.90	0.97	0	0	0
CMR	Cameroon	12466	Vina	0.86	0.90	0.97	0	0	0
CMR	Cameroon	12467	Haute - Sanaga	0.91	0.94	0.99	0	0	0
CMR	Cameroon	12468	Lékié	0.91	0.94	0.99	0	0	0
CMR	Cameroon	12469	Mbam - Et - Inoubou	0.91	0.94	0.99	0	0	0
CMR	Cameroon	12470	Mbam - Et - Kim	0.91	0.94	0.99	0	0	0
CMR	Cameroon	12471	Méfou - Et - Afamba	0.91	0.94	0.99	0	0	0
CMR	Cameroon	12472	Méfou - Et - Akono	0.91	0.94	0.99	0	0	0
CMR	Cameroon	12473	Mfoundi	0.91	0.94	0.99	0	0	0
CMR	Cameroon	12474	Nyong - Et - Kéllé	0.91	0.94	0.99	0	0	0
CMR	Cameroon	12475	Nyong - Et - Mfoumou	0.91	0.95	0.99	0	0	0
CMR	Cameroon	12476	Nyong - Et - So'o	0.91	0.94	0.99	0	0	0
CMR	Cameroon	12477	Boumba - Et - Ngoko	0.93	0.95	0.99	0	0	0
CMR	Cameroon	12478	Haut - Nyong	0.93	0.95	0.99	0	0	0
CMR	Cameroon	12479	Kadeï	0.93	0.95	0.99	0	0	0

CMR	Cameroon	12480	Lom - Et - Djerem	0.93	0.95	0.99	0	0	0
CMR	Cameroon	12481	Diamaré	0.31	0.36	0.38	426854	385643	365902
CMR	Cameroon	12482	Logone - Et - Chari	0.29	0.33	0.34	292222	270493	260684
CMR	Cameroon	12483	Mayo-Danay	0.29	0.33	0.34	369488	342014	329611
CMR	Cameroon	12484	Mayo-Kani	0.29	0.33	0.34	274019	253644	244446
CMR	Cameroon	12485	Mayo-Sava	0.29	0.33	0.34	199627	184783	178082
CMR	Cameroon	12486	Mayo-Tsanaga	0.29	0.33	0.34	419193	388023	373952
CMR	Cameroon	12487	Moungo	0.67	0.75	0.82	105540	39495	0
CMR	Cameroon	12488	Nkam	0.67	0.75	0.82	14747	5519	0
CMR	Cameroon	12489	Sanaga - Maritime	0.67	0.75	0.82	49822	18644	0
CMR	Cameroon	12490	Wouril	0.29	0.33	0.34	1169425	1082471	1043216
CMR	Cameroon	12491	Boyo	0.86	0.90	0.97	0	0	0
CMR	Cameroon	12492	Bui	0.86	0.90	0.97	0	0	0
CMR	Cameroon	12493	Donga - Mantung	0.86	0.90	0.97	0	0	0
CMR	Cameroon	12494	Menchum	0.86	0.90	0.97	0	0	0
CMR	Cameroon	12495	Mezam	0.86	0.90	0.97	0	0	0
CMR	Cameroon	12496	Momo	0.86	0.90	0.97	0	0	0
CMR	Cameroon	12497	Ngo-Ketunjia	0.86	0.90	0.97	0	0	0
CMR	Cameroon	12498	Bénoue	0.92	0.94	0.99	0	0	0
CMR	Cameroon	12499	Faro	0.92	0.94	0.99	0	0	0
CMR	Cameroon	12500	Mayo-Louti	0.92	0.94	0.99	0	0	0
CMR	Cameroon	12501	Mayo - Rey	0.92	0.94	0.99	0	0	0
CMR	Cameroon	12502	Bamboutos	0.93	0.98	0.99	0	0	0
CMR	Cameroon	12503	Haut-Nkam	0.93	0.98	0.99	0	0	0
CMR	Cameroon	12504	Hauts-Plateaux	0.93	0.98	0.99	0	0	0
CMR	Cameroon	12505	Koung-Khi	0.93	0.99	0.99	0	0	0
CMR	Cameroon	12506	Ménoua	0.93	0.99	0.99	0	0	0
CMR	Cameroon	12507	Mifi	0.93	0.98	0.99	0	0	0
CMR	Cameroon	12508	Nde	0.93	0.98	0.99	0	0	0
CMR	Cameroon	12509	Noun	0.93	0.98	0.99	0	0	0
CMR	Cameroon	12510	Fako	0.83	0.93	0.98	0	0	0
CMR	Cameroon	12511	Kupé Manenguba	0.83	0.88	0.94	0	0	0
CMR	Cameroon	12512	Lebialem	0.83	0.88	0.94	0	0	0

CMR	Cameroon	12513	Manyu	0.83	0.88	0.94	0	0	0
CMR	Cameroon	12514	Meme	0.83	0.93	0.98	0	0	0
CMR	Cameroon	12515	Ndian	0.83	0.88	0.94	0	0	0
CMR	Cameroon	12516	Dja - Et - Lobo	0.84	0.91	0.99	0	0	0
CMR	Cameroon	12517	Mvila	0.84	0.89	0.95	0	0	0
CMR	Cameroon	12518	Océan	0.84	0.89	0.95	0	0	0
CMR	Cameroon	12519	Vallée - Du - Ntem	0.84	0.89	0.95	0	0	0
COD	Democratic Republic of the Congo	14959	Bandundu (city)	0.24	0.24	0.24	94955	94955	94955
COD	Democratic Republic of the Congo	14960	Kwango	0.54	0.63	0.71	451282	297290	147151
COD	Democratic Republic of the Congo	14961	Kwilu	0.24	0.24	0.24	2318148	2318148	2318148
COD	Democratic Republic of the Congo	14965	Cataractes	0.54	0.63	0.71	266529	175581	86908
COD	Democratic Republic of the Congo	14966	Lukaya	0.54	0.63	0.71	156584	103153	51058
COD	Democratic Republic of the Congo	14967	Matadi (city)	0.24	0.24	0.24	81913	81913	81913
COD	Democratic Republic of the Congo	14968	Equateur	0.24	0.24	0.24	820593	820593	820593
COD	Democratic Republic of the Congo	14969	Mbandaka (city)	0.24	0.24	0.24	26804	26804	26804
COD	Democratic Republic of the Congo	14970	Mongala	0.24	0.24	0.24	858258	858258	858258
COD	Democratic Republic of the Congo	14971	Nord-Ubangi	0.24	0.24	0.24	566552	566552	566552
COD	Democratic Republic of the Congo	14972	Sud-Ubangi	0.32	0.32	0.32	975245	971082	964719
COD	Democratic Republic of the Congo	14973	Tshuapa	0.24	0.24	0.24	589842	589842	589842
COD	Democratic Republic of the Congo	14974	Kananga (city)	0.24	0.24	0.24	139388	139388	139388
COD	Democratic Republic of the Congo	14976	Lulua	0.54	0.63	0.71	739267	487006	241055
COD	Democratic Republic of the Congo	14978	Mbuji-Mayi (city)	0.24	0.24	0.24	517613	517613	517613
COD	Democratic Republic of the Congo	14979	Sankuru	0.24	0.24	0.24	1249662	1249662	1249662
COD	Democratic Republic of the Congo	14980	Tshilenge	0.24	0.24	0.24	1147667	1147667	1147667
COD	Democratic Republic of the Congo	14981	Haut-Lomami	0.41	0.58	0.70	1165325	647100	284235
COD	Democratic Republic of the Congo	14982	Haut-Katanga	0.24	0.24	0.24	1065345	1065345	1065345
COD	Democratic Republic of the Congo	14983	Kolwezi	0.24	0.24	0.24	495098	495098	495098
COD	Democratic Republic of the Congo	14984	Lualaba	0.54	0.63	0.71	236871	156043	77237
COD	Democratic Republic of the Congo	14985	Lubumbashi (city)	0.24	0.24	0.24	227500	227500	227500
COD	Democratic Republic of the Congo	14986	Tanganyika	0.24	0.24	0.24	1121929	1121929	1121929
COD	Democratic Republic of the Congo	14987	Kinshasa	0.54	0.63	0.71	1957811	1289744	638388
COD	Democratic Republic of the Congo	14988	Under 1st level administration	0.24	0.24	0.24	983367	983367	983367
COD	Democratic Republic of the Congo	14989	Under 1st level administration	0.24	0.24	0.24	2443552	2443552	2443552

COD	Democratic Republic of the Congo	14990	Bas-Uele	0.35	0.57	0.71	446147	229503	92082
COD	Democratic Republic of the Congo	14991	Haut-Uele	0.24	0.24	0.24	3527370	3527370	3527370
COD	Democratic Republic of the Congo	14992	Ituri	0.24	0.24	0.24	2242436	2242436	2242436
COD	Democratic Republic of the Congo	14993	Kisangani (city)	0.24	0.24	0.24	623837	623837	623837
COD	Democratic Republic of the Congo	14994	Tshopo	0.24	0.24	0.24	924078	924078	924078
COD	Democratic Republic of the Congo	14995	Under 1st level administration	0.24	0.24	0.24	2518426	2518426	2518426
COD	Democratic Republic of the Congo	74352	Bas-Fleuve	0.54	0.63	0.71	270088	177925	88068
COD	Democratic Republic of the Congo	74353	Boma (city)	0.24	0.24	0.24	81503	81503	81503
COD	Democratic Republic of the Congo	74354	Bukavu (city)	0.24	0.24	0.24	112902	112902	112902
COD	Democratic Republic of the Congo	74355	Gbadolite (city)	0.24	0.24	0.24	61638	61638	61638
COD	Democratic Republic of the Congo	74356	Goma (city)	0.24	0.24	0.24	137822	137822	137822
COD	Democratic Republic of the Congo	74357	Kikwit (city)	0.24	0.24	0.24	159889	159889	159889
COD	Democratic Republic of the Congo	74358	Kindu (city)	0.24	0.24	0.24	113997	113997	113997
COD	Democratic Republic of the Congo	74359	Kolwezi (city)	0.24	0.24	0.24	130827	130827	130827
COD	Democratic Republic of the Congo	74360	Likasi (city)	0.24	0.24	0.24	19190	19190	19190
COD	Democratic Republic of the Congo	74361	Mai-Ndombe	0.24	0.24	0.24	618782	618782	618782
COD	Democratic Republic of the Congo	74362	Mwene-Ditu (city)	0.24	0.24	0.24	75632	75632	75632
COD	Democratic Republic of the Congo	74363	Plateaux	0.24	0.24	0.24	250198	250198	250198
COD	Democratic Republic of the Congo	74364	Tshikapa (city)	0.24	0.24	0.24	314272	314272	314272
COD	Democratic Republic of the Congo	74365	Zongo (city)	0.24	0.24	0.24	43456	43456	43456
COD	Democratic Republic of the Congo	74366	Kabinda	0.41	0.45	0.50	933330	822827	704918
COD	Democratic Republic of the Congo	74367	Kasai	0.54	0.63	0.71	591638	389753	192917
COG	Congo	14423	Boko-songho	0.16	0.34	0.43	3569	2571	2062
COG	Congo	14424	Loudima	0.16	0.34	0.43	46945	33822	27122
COG	Congo	14425	Madingou	0.16	0.34	0.43	45209	32571	26119
COG	Congo	14426	Mfouati	0.16	0.34	0.43	4444	3202	2568
COG	Congo	14427	Mouyondzi	0.16	0.34	0.43	66773	48107	38578
COG	Congo	14428	Nkayi District	0.16	0.34	0.43	29415	21193	16994
COG	Congo	14429	Ewo	0.58	0.70	0.76	1568	686	271
COG	Congo	14430	Kelle	0.58	0.70	0.76	1594	697	275
COG	Congo	14431	Mbomo	0.58	0.70	0.76	506	221	87
COG	Congo	14432	Boundji	0.16	0.34	0.43	3356	2418	1939
COG	Congo	14433	Loukela	0.16	0.34	0.43	1793	1292	1036

COG	Congo	14434	Makoua	0.16	0.34	0.43	32106	23131	18549
COG	Congo	14435	Mossaka	0.16	0.34	0.43	31121	22422	17980
COG	Congo	14436	Okoyo	0.16	0.34	0.43	1926	1387	1113
COG	Congo	14437	Owando	0.16	0.34	0.43	45281	32623	26161
COG	Congo	14438	Kakamoeka	0.16	0.34	0.43	5240	3776	3028
COG	Congo	14439	Loandjili (pointe Noire)	0.16	0.34	0.43	720654	519204	416355
COG	Congo	14440	Madingo-kayes	0.16	0.34	0.43	3389	2442	1958
COG	Congo	14441	Mvouti	0.16	0.34	0.43	8147	5869	4707
COG	Congo	14442	Bambama	0.16	0.34	0.43	1041	750	601
COG	Congo	14443	Komono	0.16	0.34	0.43	2827	2037	1633
COG	Congo	14444	Sibiti	0.16	0.34	0.43	34564	24902	19969
COG	Congo	14445	Zanaga	0.16	0.34	0.43	3470	2500	2005
COG	Congo	14446	Dongou	0.16	0.34	0.43	9345	6733	5399
COG	Congo	14447	Epena	0.16	0.34	0.43	4746	3420	2742
COG	Congo	14448	Impfondo	0.16	0.34	0.43	26654	19203	15399
COG	Congo	14449	Divenie	0.16	0.34	0.43	6310	4546	3646
COG	Congo	14450	Kibangou	0.16	0.34	0.43	4961	3575	2866
COG	Congo	14451	Kimongo	0.16	0.34	0.43	7308	5265	4222
COG	Congo	14452	Louvakou (loubomo)	0.16	0.34	0.43	117557	84696	67918
COG	Congo	14453	Mayoko	0.16	0.34	0.43	3140	2262	1814
COG	Congo	14454	Mossendjo	0.16	0.34	0.43	16049	11563	9272
COG	Congo	14455	Abala	0.16	0.34	0.43	8876	6395	5128
COG	Congo	14456	Djambala	0.16	0.34	0.43	24618	17736	14223
COG	Congo	14457	Gamboma	0.16	0.34	0.43	53455	38512	30883
COG	Congo	14458	Lekana	0.16	0.34	0.43	4159	2996	2403
COG	Congo	14459	Boko	0.16	0.34	0.43	15151	10915	8753
COG	Congo	14460	Kindamba	0.16	0.34	0.43	11116	8008	6422
COG	Congo	14461	Kinkala	0.16	0.34	0.43	44627	32152	25783
COG	Congo	14462	Mayama	0.16	0.34	0.43	7241	5217	4183
COG	Congo	14463	Mindouli	0.16	0.34	0.43	16078	11584	9289
COG	Congo	14464	Ngabe	0.16	0.34	0.43	9853	7099	5693
COG	Congo	14465	Ngamaba (brazzaville)	0.16	0.34	0.43	1497420	1078835	865128
COG	Congo	14466	Ouesso	0.16	0.34	0.43	24557	17693	14188

COG	Congo	14467	Sembe	0.16	0.34	0.43	4889	3522	2825
COG	Congo	14468	Souanke	0.16	0.34	0.43	4630	3336	2675
COL	Colombia	13333	El Encanto	0.14	0.14	0.14	2405	2405	2405
COL	Colombia	13334	La Chorrera	0.14	0.14	0.14	6128	6128	6128
COL	Colombia	13335	La Pedrera	0.14	0.14	0.14	4390	4390	4390
COL	Colombia	13336	Leticia	0.14	0.14	0.14	24828	24828	24828
COL	Colombia	13337	Miriti Parana	0.14	0.14	0.14	2877	2877	2877
COL	Colombia	13338	Puerto Narino	0.14	0.14	0.14	9779	9779	9779
COL	Colombia	13339	Puerto Santander	0.14	0.14	0.14	1153	1153	1153
COL	Colombia	13340	Tarapaca	0.14	0.14	0.14	12993	12993	12993
COL	Colombia	13341	Abejorral	0.14	0.14	0.14	15600	15600	15600
COL	Colombia	13342	Abriaqui	0.14	0.14	0.14	2069	2069	2069
COL	Colombia	13343	Alejandria	0.14	0.14	0.14	3204	3204	3204
COL	Colombia	13344	Amaga	0.14	0.14	0.14	18204	18204	18204
COL	Colombia	13345	Amalfi	0.14	0.14	0.14	14877	14877	14877
COL	Colombia	13346	Andes	0.14	0.14	0.14	27036	27036	27036
COL	Colombia	13347	Angelopolis	0.14	0.14	0.14	5685	5685	5685
COL	Colombia	13348	Angostura	0.14	0.14	0.14	10451	10451	10451
COL	Colombia	13349	Anori	0.14	0.14	0.14	13291	13291	13291
COL	Colombia	13350	Antioquia	0.14	0.14	0.14	14308	14308	14308
COL	Colombia	13351	Anza	0.14	0.14	0.14	4885	4885	4885
COL	Colombia	13352	Apartado	0.14	0.14	0.14	89694	89694	89694
COL	Colombia	13353	Arboletes	0.14	0.14	0.14	14919	14919	14919
COL	Colombia	13354	Argelia	0.14	0.14	0.14	6717	6717	6717
COL	Colombia	13355	Armenia	0.14	0.14	0.14	5146	5146	5146
COL	Colombia	13356	Barbosa	0.14	0.14	0.14	34610	34610	34610
COL	Colombia	13357	Bello	0.14	0.14	0.14	289264	289264	289264
COL	Colombia	13358	Belmira	0.14	0.14	0.14	7176	7176	7176
COL	Colombia	13359	Betania	0.14	0.14	0.14	8427	8427	8427
COL	Colombia	13360	Betulia	0.14	0.14	0.14	10217	10217	10217
COL	Colombia	13361	Bolivar	0.14	0.14	0.14	20859	20859	20859
COL	Colombia	13362	Briceno	0.14	0.14	0.14	6378	6378	6378
COL	Colombia	13363	Buritica	0.14	0.14	0.14	4488	4488	4488

COL	Colombia	13364	Caceres	0.14	0.14	0.14	23958	23958	23958
COL	Colombia	13365	Caicedo	0.14	0.14	0.14	6278	6278	6278
COL	Colombia	13366	Caldas	0.14	0.14	0.14	46517	46517	46517
COL	Colombia	13367	Campamento	0.14	0.14	0.14	7714	7714	7714
COL	Colombia	13368	Canasgordas	0.14	0.14	0.14	13920	13920	13920
COL	Colombia	13369	Caracoli	0.14	0.14	0.14	5046	5046	5046
COL	Colombia	13370	Caramanta	0.14	0.14	0.14	5566	5566	5566
COL	Colombia	13371	Carepa	0.14	0.14	0.14	21595	21595	21595
COL	Colombia	13372	Carmen De Viboral	0.14	0.14	0.14	32378	32378	32378
COL	Colombia	13373	Carolina	0.14	0.14	0.14	3058	3058	3058
COL	Colombia	13374	Caucasia	0.14	0.14	0.14	56980	56980	56980
COL	Colombia	13375	Chigorodo	0.14	0.14	0.14	42006	42006	42006
COL	Colombia	13376	Cisneros	0.14	0.14	0.14	4639	4639	4639
COL	Colombia	13377	Cocorna	0.14	0.14	0.14	13956	13956	13956
COL	Colombia	13378	Concepcion	0.14	0.14	0.14	4119	4119	4119
COL	Colombia	13379	Concordia	0.14	0.14	0.14	12527	12527	12527
COL	Colombia	13380	Copacabana	0.14	0.14	0.14	65250	65250	65250
COL	Colombia	13381	Dabeiba	0.14	0.14	0.14	16132	16132	16132
COL	Colombia	13382	Don Matias	0.14	0.14	0.14	14823	14823	14823
COL	Colombia	13383	Ebejico	0.14	0.14	0.14	7884	7884	7884
COL	Colombia	13384	El Bagre	0.14	0.14	0.14	38614	38614	38614
COL	Colombia	13385	Entrerrios	0.14	0.14	0.14	5414	5414	5414
COL	Colombia	13386	Envigado	0.14	0.14	0.14	123977	123977	123977
COL	Colombia	13387	Fredonia	0.14	0.14	0.14	14440	14440	14440
COL	Colombia	13388	Frontino	0.14	0.14	0.14	14882	14882	14882
COL	Colombia	13389	Giraldo	0.14	0.14	0.14	2437	2437	2437
COL	Colombia	13390	Girardota	0.14	0.14	0.14	36316	36316	36316
COL	Colombia	13391	Gomez Plata	0.14	0.14	0.14	9743	9743	9743
COL	Colombia	13392	Granada	0.14	0.14	0.14	12912	12912	12912
COL	Colombia	13393	Guadalupe	0.14	0.14	0.14	2958	2958	2958
COL	Colombia	13394	Guarne	0.14	0.14	0.14	139968	139968	139968
COL	Colombia	13395	Guatape	0.14	0.14	0.14	4076	4076	4076
COL	Colombia	13396	Heliconia	0.14	0.14	0.14	4632	4632	4632

COL	Colombia	13397	Hispania	0.14	0.14	0.14	2365	2365	2365
COL	Colombia	13398	Itagui	0.14	0.14	0.14	139935	139935	139935
COL	Colombia	13399	Ituango	0.14	0.14	0.14	18214	18214	18214
COL	Colombia	13400	Jardin	0.14	0.14	0.14	13860	13860	13860
COL	Colombia	13401	Jerico	0.14	0.14	0.14	8507	8507	8507
COL	Colombia	13402	La Ceja	0.14	0.14	0.14	26605	26605	26605
COL	Colombia	13403	La Estrella	0.14	0.14	0.14	30901	30901	30901
COL	Colombia	13404	La Union	0.14	0.14	0.14	19680	19680	19680
COL	Colombia	13405	Liborina	0.14	0.14	0.14	7131	7131	7131
COL	Colombia	13406	Maceo	0.14	0.14	0.14	6447	6447	6447
COL	Colombia	13407	Marinilla	0.14	0.14	0.14	29630	29630	29630
COL	Colombia	13408	Medellin	0.14	0.14	0.14	1371317	1371317	1371317
COL	Colombia	13409	Montebello	0.14	0.14	0.14	5814	5814	5814
COL	Colombia	13410	Murindo	0.14	0.14	0.14	3854	3854	3854
COL	Colombia	13411	Mutata	0.14	0.14	0.14	13953	13953	13953
COL	Colombia	13412	Narino	0.14	0.14	0.14	9897	9897	9897
COL	Colombia	13413	Nechi	0.14	0.14	0.14	8606	8606	8606
COL	Colombia	13414	Necocli	0.14	0.14	0.14	33265	33265	33265
COL	Colombia	13416	Olaya	0.14	0.14	0.14	3342	3342	3342
COL	Colombia	13417	Penol	0.14	0.14	0.14	18485	18485	18485
COL	Colombia	13418	Peque	0.14	0.14	0.14	6139	6139	6139
COL	Colombia	13419	Pto Nare (la Magd.)	0.14	0.14	0.14	11487	11487	11487
COL	Colombia	13420	Pueblorrico	0.14	0.14	0.14	8373	8373	8373
COL	Colombia	13421	Puerto Berrio	0.14	0.14	0.14	26031	26031	26031
COL	Colombia	13422	Puerto Triunfo	0.14	0.14	0.14	10828	10828	10828
COL	Colombia	13423	Remedios	0.14	0.14	0.14	17058	17058	17058
COL	Colombia	13424	Retiro	0.14	0.14	0.14	92365	92365	92365
COL	Colombia	13425	Rionegro	0.14	0.14	0.14	90578	90578	90578
COL	Colombia	13426	Sabanalarga	0.14	0.14	0.14	5490	5490	5490
COL	Colombia	13427	Sabaneta	0.14	0.14	0.14	30203	30203	30203
COL	Colombia	13428	Salgar	0.14	0.14	0.14	14936	14936	14936
COL	Colombia	13429	San Andres	0.14	0.14	0.14	4245	4245	4245
COL	Colombia	13430	San Carlos	0.14	0.14	0.14	12831	12831	12831

COL	Colombia	13431	San Francisco	0.14	0.14	0.14	5422	5422	5422
COL	Colombia	13432	San Jeronimo	0.14	0.14	0.14	7493	7493	7493
COL	Colombia	13433	San Jose De La Montana	0.14	0.14	0.14	3563	3563	3563
COL	Colombia	13434	San Juan De Uraba	0.14	0.14	0.14	14340	14340	14340
COL	Colombia	13435	San Luis	0.14	0.14	0.14	7173	7173	7173
COL	Colombia	13436	San Pedro	0.14	0.14	0.14	28800	28800	28800
COL	Colombia	13437	San Pedro De Uraba	0.14	0.14	0.14	25928	25928	25928
COL	Colombia	13438	San Rafael	0.14	0.14	0.14	10356	10356	10356
COL	Colombia	13439	San Roque	0.14	0.14	0.14	13011	13011	13011
COL	Colombia	13440	San Vicente	0.14	0.14	0.14	17831	17831	17831
COL	Colombia	13441	Santa Barbara	0.14	0.14	0.14	19586	19586	19586
COL	Colombia	13442	Santa Rosa De Osos	0.14	0.14	0.14	23673	23673	23673
COL	Colombia	13443	Santo Domingo	0.14	0.14	0.14	9473	9473	9473
COL	Colombia	13444	Santuario	0.14	0.14	0.14	13510	13510	13510
COL	Colombia	13445	Segovia	0.14	0.14	0.14	25531	25531	25531
COL	Colombia	13446	Sonson	0.14	0.14	0.14	28403	28403	28403
COL	Colombia	13447	Sopetran	0.14	0.14	0.14	8148	8148	8148
COL	Colombia	13448	Tamesis	0.14	0.14	0.14	12452	12452	12452
COL	Colombia	13449	Taraza	0.14	0.14	0.14	21799	21799	21799
COL	Colombia	13450	Tarso	0.14	0.14	0.14	6845	6845	6845
COL	Colombia	13451	Titiribi	0.14	0.14	0.14	9858	9858	9858
COL	Colombia	13452	Toledo	0.14	0.14	0.14	4374	4374	4374
COL	Colombia	13453	Turbo	0.14	0.14	0.14	99256	99256	99256
COL	Colombia	13455	Uramita	0.14	0.14	0.14	3813	3813	3813
COL	Colombia	13456	Urrao	0.14	0.14	0.14	31409	31409	31409
COL	Colombia	13457	Valdivia	0.14	0.14	0.14	11463	11463	11463
COL	Colombia	13458	Valparaiso	0.14	0.14	0.14	4850	4850	4850
COL	Colombia	13459	Vegachi	0.14	0.14	0.14	7913	7913	7913
COL	Colombia	13460	Venecia	0.14	0.14	0.14	9513	9513	9513
COL	Colombia	13461	Vigia Del Fuerte	0.14	0.14	0.14	5222	5222	5222
COL	Colombia	13462	Yali	0.14	0.14	0.14	6390	6390	6390
COL	Colombia	13463	Yarumal	0.14	0.14	0.14	28049	28049	28049
COL	Colombia	13464	Yolombo	0.14	0.14	0.14	15756	15756	15756

COL	Colombia	13465	Yondo	0.14	0.14	0.14	12502	12502	12502
COL	Colombia	13466	Zaragoza	0.14	0.14	0.14	16791	16791	16791
COL	Colombia	13467	Arauca	0.14	0.14	0.14	69549	69549	69549
COL	Colombia	13468	Arauquita	0.14	0.14	0.14	14428	14428	14428
COL	Colombia	13469	Cravo Norte	0.14	0.14	0.14	4783	4783	4783
COL	Colombia	13470	Fortul	0.14	0.14	0.14	23869	23869	23869
COL	Colombia	13471	Puerto Rondon	0.14	0.14	0.14	27731	27731	27731
COL	Colombia	13472	Saravena	0.14	0.14	0.14	33936	33936	33936
COL	Colombia	13473	Tame	0.14	0.14	0.14	39089	39089	39089
COL	Colombia	13474	Baranoa	0.14	0.14	0.14	35758	35758	35758
COL	Colombia	13475	Barranquilla (dist.port.	0.14	0.14	0.14	877153	877153	877153
COL	Colombia	13476	Campo De La Cruz	0.14	0.14	0.14	10246	10246	10246
COL	Colombia	13477	Candelaria	0.14	0.14	0.14	8425	8425	8425
COL	Colombia	13478	Galapa	0.14	0.14	0.14	63131	63131	63131
COL	Colombia	13479	Juan De Acosta	0.14	0.14	0.14	11989	11989	11989
COL	Colombia	13480	Luruaco	0.14	0.14	0.14	18624	18624	18624
COL	Colombia	13481	Malambo	0.14	0.14	0.14	85416	85416	85416
COL	Colombia	13482	Manati	0.14	0.14	0.14	12303	12303	12303
COL	Colombia	13483	Palmar De Varela	0.14	0.14	0.14	11330	11330	11330
COL	Colombia	13484	Piojo	0.14	0.14	0.14	3515	3515	3515
COL	Colombia	13485	Polo Nuevo	0.14	0.14	0.14	11464	11464	11464
COL	Colombia	13486	Ponedera	0.14	0.14	0.14	14532	14532	14532
COL	Colombia	13487	Puerto Colombia	0.14	0.14	0.14	47001	47001	47001
COL	Colombia	13488	Repelon	0.14	0.14	0.14	21205	21205	21205
COL	Colombia	13489	Sabanagrande	0.14	0.14	0.14	14501	14501	14501
COL	Colombia	13490	Sabanalarga	0.14	0.14	0.14	62759	62759	62759
COL	Colombia	13491	Santa Lucia	0.14	0.14	0.14	6302	6302	6302
COL	Colombia	13492	Santo Tomas	0.14	0.14	0.14	13059	13059	13059
COL	Colombia	13493	Soledad	0.14	0.14	0.14	299296	299296	299296
COL	Colombia	13494	Suan	0.14	0.14	0.14	2910	2910	2910
COL	Colombia	13495	Tubara	0.14	0.14	0.14	12054	12054	12054
COL	Colombia	13496	Usiacuri	0.14	0.14	0.14	4995	4995	4995
COL	Colombia	13497	Achi	0.14	0.14	0.14	27336	27336	27336

COL	Colombia	13498	Altos Del Rosario	0.14	0.14	0.14	11949	11949	11949
COL	Colombia	13499	Arjona	0.14	0.14	0.14	40745	40745	40745
COL	Colombia	13500	Barranco De Loba	0.14	0.14	0.14	12184	12184	12184
COL	Colombia	13501	Calamar	0.14	0.14	0.14	18112	18112	18112
COL	Colombia	13502	Cantagallo	0.14	0.14	0.14	9433	9433	9433
COL	Colombia	13503	Cartagena (dist. Esp.)	0.14	0.14	0.14	616735	616735	616735
COL	Colombia	13504	Cartagena (dist. Esp.) (i	0.14	0.14	0.14	22757	22757	22757
COL	Colombia	13505	Cicuco	0.14	0.14	0.14	3538	3538	3538
COL	Colombia	13506	Cordoba	0.14	0.14	0.14	6700	6700	6700
COL	Colombia	13507	El Carmen De Bolivar	0.14	0.14	0.14	35689	35689	35689
COL	Colombia	13508	El Guamo	0.14	0.14	0.14	5069	5069	5069
COL	Colombia	13509	Hatillo De Loba	0.14	0.14	0.14	5836	5836	5836
COL	Colombia	13510	Magangue	0.14	0.14	0.14	81958	81958	81958
COL	Colombia	13511	Mahates	0.14	0.14	0.14	19324	19324	19324
COL	Colombia	13512	Margarita	0.14	0.14	0.14	13366	13366	13366
COL	Colombia	13513	Maria La Baja	0.14	0.14	0.14	26105	26105	26105
COL	Colombia	13514	Mompos	0.14	0.14	0.14	24750	24750	24750
COL	Colombia	13515	Montecristo	0.14	0.14	0.14	17264	17264	17264
COL	Colombia	13516	Morales	0.14	0.14	0.14	45854	45854	45854
COL	Colombia	13517	Pinillos	0.14	0.14	0.14	67981	67981	67981
COL	Colombia	13518	Rio Viejo	0.14	0.14	0.14	8106	8106	8106
COL	Colombia	13519	San Estanislao	0.14	0.14	0.14	9392	9392	9392
COL	Colombia	13520	San Fernando	0.14	0.14	0.14	19158	19158	19158
COL	Colombia	13521	San Jacinto	0.14	0.14	0.14	13007	13007	13007
COL	Colombia	13522	San Juan Nepomuceno	0.14	0.14	0.14	19601	19601	19601
COL	Colombia	13523	San Martin De Loba	0.14	0.14	0.14	8085	8085	8085
COL	Colombia	13524	San Pablo	0.14	0.14	0.14	13715	13715	13715
COL	Colombia	13525	Santa Catalina	0.14	0.14	0.14	31030	31030	31030
COL	Colombia	13526	Santa Rosa	0.14	0.14	0.14	19049	19049	19049
COL	Colombia	13527	Santa Rosa Del Sur	0.14	0.14	0.14	30027	30027	30027
COL	Colombia	13528	Simiti	0.14	0.14	0.14	17001	17001	17001
COL	Colombia	13529	Soplaviento	0.14	0.14	0.14	7349	7349	7349
COL	Colombia	13530	Talaigua Nuevo	0.14	0.14	0.14	3831	3831	3831

COL	Colombia	13531	Tiquisio	0.14	0.14	0.14	69596	69596	69596
COL	Colombia	13532	Turbaco	0.14	0.14	0.14	41357	41357	41357
COL	Colombia	13533	Turbana	0.14	0.14	0.14	16986	16986	16986
COL	Colombia	13534	Villanueva	0.14	0.14	0.14	16076	16076	16076
COL	Colombia	13535	Zambrano	0.14	0.14	0.14	6587	6587	6587
COL	Colombia	13536	Almeida	0.14	0.14	0.14	738	738	738
COL	Colombia	13537	Aquitania	0.14	0.14	0.14	11277	11277	11277
COL	Colombia	13538	Arcabuco	0.14	0.14	0.14	6656	6656	6656
COL	Colombia	13539	Belen	0.14	0.14	0.14	3905	3905	3905
COL	Colombia	13540	Berbeo	0.14	0.14	0.14	1333	1333	1333
COL	Colombia	13541	Beteitiva	0.14	0.14	0.14	2627	2627	2627
COL	Colombia	13542	Boavita	0.14	0.14	0.14	3837	3837	3837
COL	Colombia	13543	Boyaca	0.14	0.14	0.14	4315	4315	4315
COL	Colombia	13544	Brice¥o	0.14	0.14	0.14	1509	1509	1509
COL	Colombia	13545	Buenavista	0.14	0.14	0.14	2139	2139	2139
COL	Colombia	13546	Busbanza	0.14	0.14	0.14	892	892	892
COL	Colombia	13547	Caldas	0.14	0.14	0.14	2612	2612	2612
COL	Colombia	13548	Campohermoso	0.14	0.14	0.14	2669	2669	2669
COL	Colombia	13549	Cerinza	0.14	0.14	0.14	3405	3405	3405
COL	Colombia	13550	Chinavita	0.14	0.14	0.14	3870	3870	3870
COL	Colombia	13551	Chiquinquira	0.14	0.14	0.14	18045	18045	18045
COL	Colombia	13552	Chiquiza	0.14	0.14	0.14	1877	1877	1877
COL	Colombia	13553	Chiscas	0.14	0.14	0.14	5210	5210	5210
COL	Colombia	13554	Chita	0.14	0.14	0.14	4166	4166	4166
COL	Colombia	13555	Chitaraque	0.14	0.14	0.14	3369	3369	3369
COL	Colombia	13556	Chivata	0.14	0.14	0.14	4083	4083	4083
COL	Colombia	13557	Chivor	0.14	0.14	0.14	1110	1110	1110
COL	Colombia	13558	Cienega	0.14	0.14	0.14	2558	2558	2558
COL	Colombia	13559	Combita	0.14	0.14	0.14	14996	14996	14996
COL	Colombia	13560	Coper	0.14	0.14	0.14	2783	2783	2783
COL	Colombia	13561	Corrales	0.14	0.14	0.14	7442	7442	7442
COL	Colombia	13562	Covarachia	0.14	0.14	0.14	885	885	885
COL	Colombia	13563	Cubara	0.14	0.14	0.14	5386	5386	5386

COL	Colombia	13564	Cucaita	0.14	0.14	0.14	2585	2585	2585
COL	Colombia	13565	Cuitiva	0.14	0.14	0.14	661	661	661
COL	Colombia	13566	Duitama	0.14	0.14	0.14	56277	56277	56277
COL	Colombia	13567	El Cocuy	0.14	0.14	0.14	1991	1991	1991
COL	Colombia	13568	El Espino	0.14	0.14	0.14	1880	1880	1880
COL	Colombia	13569	Firavitoba	0.14	0.14	0.14	2730	2730	2730
COL	Colombia	13570	Floresta	0.14	0.14	0.14	8635	8635	8635
COL	Colombia	13571	Gachantiva	0.14	0.14	0.14	2196	2196	2196
COL	Colombia	13572	Gameza	0.14	0.14	0.14	2844	2844	2844
COL	Colombia	13573	Garagoa	0.14	0.14	0.14	9465	9465	9465
COL	Colombia	13574	Guacamayas	0.14	0.14	0.14	1079	1079	1079
COL	Colombia	13575	Guateque	0.14	0.14	0.14	2504	2504	2504
COL	Colombia	13576	Guayata	0.14	0.14	0.14	1663	1663	1663
COL	Colombia	13577	Guican	0.14	0.14	0.14	4668	4668	4668
COL	Colombia	13578	Iza	0.14	0.14	0.14	1326	1326	1326
COL	Colombia	13579	Jenesano	0.14	0.14	0.14	2929	2929	2929
COL	Colombia	13580	Jerico	0.14	0.14	0.14	1677	1677	1677
COL	Colombia	13581	La Capilla	0.14	0.14	0.14	2437	2437	2437
COL	Colombia	13582	La Uvita	0.14	0.14	0.14	2081	2081	2081
COL	Colombia	13583	La Victoria	0.14	0.14	0.14	780	780	780
COL	Colombia	13584	Labranzagrande	0.14	0.14	0.14	3625	3625	3625
COL	Colombia	13585	Macanal	0.14	0.14	0.14	2558	2558	2558
COL	Colombia	13586	Maripi	0.14	0.14	0.14	5029	5029	5029
COL	Colombia	13587	Miraflores	0.14	0.14	0.14	6852	6852	6852
COL	Colombia	13588	Mongua	0.14	0.14	0.14	9160	9160	9160
COL	Colombia	13589	Mongui	0.14	0.14	0.14	15331	15331	15331
COL	Colombia	13590	Moniquira	0.14	0.14	0.14	13272	13272	13272
COL	Colombia	13591	Motavita	0.14	0.14	0.14	2781	2781	2781
COL	Colombia	13592	Muzo	0.14	0.14	0.14	4793	4793	4793
COL	Colombia	13593	Nobsa	0.14	0.14	0.14	9864	9864	9864
COL	Colombia	13594	Nuevo Colon	0.14	0.14	0.14	2129	2129	2129
COL	Colombia	13595	Oicata	0.14	0.14	0.14	22596	22596	22596
COL	Colombia	13596	Otanche	0.14	0.14	0.14	7483	7483	7483

COL	Colombia	13597	Pachavita	0.14	0.14	0.14	2171	2171	2171
COL	Colombia	13598	Paez	0.14	0.14	0.14	2037	2037	2037
COL	Colombia	13599	Paipa	0.14	0.14	0.14	14769	14769	14769
COL	Colombia	13600	Pajarito	0.14	0.14	0.14	7956	7956	7956
COL	Colombia	13601	Panqueba	0.14	0.14	0.14	665	665	665
COL	Colombia	13602	Pauna	0.14	0.14	0.14	7782	7782	7782
COL	Colombia	13603	Paya	0.14	0.14	0.14	4717	4717	4717
COL	Colombia	13604	Paz De Rio	0.14	0.14	0.14	2207	2207	2207
COL	Colombia	13605	Pesca	0.14	0.14	0.14	5022	5022	5022
COL	Colombia	13606	Pisva	0.14	0.14	0.14	883	883	883
COL	Colombia	13607	Puerto Boyaca	0.14	0.14	0.14	42376	42376	42376
COL	Colombia	13608	Quipama	0.14	0.14	0.14	1786	1786	1786
COL	Colombia	13609	Ramiriqui	0.14	0.14	0.14	6004	6004	6004
COL	Colombia	13610	Raquira	0.14	0.14	0.14	7921	7921	7921
COL	Colombia	13611	Rondon	0.14	0.14	0.14	2880	2880	2880
COL	Colombia	13612	Saboya	0.14	0.14	0.14	21764	21764	21764
COL	Colombia	13613	Sachica	0.14	0.14	0.14	2807	2807	2807
COL	Colombia	13614	Samaca	0.14	0.14	0.14	11446	11446	11446
COL	Colombia	13615	San Eduardo	0.14	0.14	0.14	1328	1328	1328
COL	Colombia	13616	San Jose De Pare	0.14	0.14	0.14	10411	10411	10411
COL	Colombia	13617	San Luis De Gaceno	0.14	0.14	0.14	1867	1867	1867
COL	Colombia	13618	San Mateo	0.14	0.14	0.14	1961	1961	1961
COL	Colombia	13619	San Miguel De Sema	0.14	0.14	0.14	8070	8070	8070
COL	Colombia	13620	San Pablo De Borbur	0.14	0.14	0.14	5318	5318	5318
COL	Colombia	13621	Santa Rosa De Viterbo	0.14	0.14	0.14	12778	12778	12778
COL	Colombia	13622	Santa Sofia	0.14	0.14	0.14	2141	2141	2141
COL	Colombia	13623	Santamaria	0.14	0.14	0.14	4147	4147	4147
COL	Colombia	13624	Santana	0.14	0.14	0.14	4208	4208	4208
COL	Colombia	13625	Sativanorte	0.14	0.14	0.14	1447	1447	1447
COL	Colombia	13626	Sativasur	0.14	0.14	0.14	1085	1085	1085
COL	Colombia	13627	Siachoque	0.14	0.14	0.14	5499	5499	5499
COL	Colombia	13628	Soata	0.14	0.14	0.14	2379	2379	2379
COL	Colombia	13629	Socha	0.14	0.14	0.14	4227	4227	4227

COL	Colombia	13630	Socota	0.14	0.14	0.14	5885	5885	5885
COL	Colombia	13631	Sogamoso	0.14	0.14	0.14	42813	42813	42813
COL	Colombia	13632	Somondoco	0.14	0.14	0.14	2048	2048	2048
COL	Colombia	13633	Sora	0.14	0.14	0.14	1064	1064	1064
COL	Colombia	13634	Soraca	0.14	0.14	0.14	4870	4870	4870
COL	Colombia	13635	Sotaquira	0.14	0.14	0.14	7161	7161	7161
COL	Colombia	13636	Susacon	0.14	0.14	0.14	1802	1802	1802
COL	Colombia	13637	Sutamarchan	0.14	0.14	0.14	4719	4719	4719
COL	Colombia	13638	Sutatenza	0.14	0.14	0.14	3013	3013	3013
COL	Colombia	13639	Tasco	0.14	0.14	0.14	4300	4300	4300
COL	Colombia	13640	Tenza	0.14	0.14	0.14	3255	3255	3255
COL	Colombia	13641	Tibabosa	0.14	0.14	0.14	8725	8725	8725
COL	Colombia	13642	Tibana	0.14	0.14	0.14	5383	5383	5383
COL	Colombia	13643	Tinjaca	0.14	0.14	0.14	5063	5063	5063
COL	Colombia	13644	Tipacoque	0.14	0.14	0.14	1432	1432	1432
COL	Colombia	13645	Toca	0.14	0.14	0.14	6488	6488	6488
COL	Colombia	13646	Togui	0.14	0.14	0.14	3536	3536	3536
COL	Colombia	13647	Topaga	0.14	0.14	0.14	3653	3653	3653
COL	Colombia	13648	Tota	0.14	0.14	0.14	3136	3136	3136
COL	Colombia	13649	Tunja	0.14	0.14	0.14	100394	100394	100394
COL	Colombia	13650	Tunungua	0.14	0.14	0.14	814	814	814
COL	Colombia	13651	Turmeque	0.14	0.14	0.14	4292	4292	4292
COL	Colombia	13652	Tuta	0.14	0.14	0.14	5688	5688	5688
COL	Colombia	13653	Tutasa	0.14	0.14	0.14	2491	2491	2491
COL	Colombia	13654	Umbita	0.14	0.14	0.14	6642	6642	6642
COL	Colombia	13655	Ventaquemada	0.14	0.14	0.14	11030	11030	11030
COL	Colombia	13656	Villa De Leyva	0.14	0.14	0.14	7271	7271	7271
COL	Colombia	13657	Viracacha	0.14	0.14	0.14	2864	2864	2864
COL	Colombia	13658	Zetaquira	0.14	0.14	0.14	4230	4230	4230
COL	Colombia	13659	Isla	0.09	0.09	0.09	0	0	0
COL	Colombia	13660	Aguadas	0.14	0.14	0.14	13102	13102	13102
COL	Colombia	13661	Anserma	0.14	0.14	0.14	17265	17265	17265
COL	Colombia	13662	Aranzazu	0.14	0.14	0.14	5828	5828	5828

COL	Colombia	13663	Belalcazar	0.14	0.14	0.14	10580	10580	10580
COL	Colombia	13664	Chinchina	0.14	0.14	0.14	18988	18988	18988
COL	Colombia	13665	Filadelfia	0.14	0.14	0.14	11774	11774	11774
COL	Colombia	13666	La Dorada	0.14	0.14	0.14	37508	37508	37508
COL	Colombia	13667	La Merced	0.14	0.14	0.14	5520	5520	5520
COL	Colombia	13668	Manizales	0.14	0.14	0.14	196275	196275	196275
COL	Colombia	13669	Manzanares	0.14	0.14	0.14	8658	8658	8658
COL	Colombia	13670	Marmato	0.14	0.14	0.14	5632	5632	5632
COL	Colombia	13671	Marquetalia	0.14	0.14	0.14	7200	7200	7200
COL	Colombia	13672	Marulanda	0.14	0.14	0.14	7064	7064	7064
COL	Colombia	13673	Neira	0.14	0.14	0.14	62452	62452	62452
COL	Colombia	13674	Pacora	0.14	0.14	0.14	13079	13079	13079
COL	Colombia	13675	Palestina	0.14	0.14	0.14	12367	12367	12367
COL	Colombia	13676	Pensilvania	0.14	0.14	0.14	17067	17067	17067
COL	Colombia	13677	Riosucio	0.14	0.14	0.14	31810	31810	31810
COL	Colombia	13678	Risaralda	0.14	0.14	0.14	14808	14808	14808
COL	Colombia	13679	Salamina	0.14	0.14	0.14	11022	11022	11022
COL	Colombia	13680	Samana	0.14	0.14	0.14	23420	23420	23420
COL	Colombia	13681	Supia	0.14	0.14	0.14	13024	13024	13024
COL	Colombia	13682	Victoria	0.14	0.14	0.14	9555	9555	9555
COL	Colombia	13683	Villamaria	0.14	0.14	0.14	49102	49102	49102
COL	Colombia	13684	Viterbo	0.14	0.14	0.14	6468	6468	6468
COL	Colombia	13685	Albania	0.14	0.14	0.14	2514	2514	2514
COL	Colombia	13686	Belen De Los Andaquies	0.14	0.14	0.14	6449	6449	6449
COL	Colombia	13687	Cartagena Del Chaira	0.14	0.14	0.14	22444	22444	22444
COL	Colombia	13688	Curillo	0.14	0.14	0.14	5056	5056	5056
COL	Colombia	13689	El Doncello	0.14	0.14	0.14	30953	30953	30953
COL	Colombia	13690	El Paujil	0.14	0.14	0.14	14391	14391	14391
COL	Colombia	13691	Florencia	0.14	0.14	0.14	81368	81368	81368
COL	Colombia	13692	La Montanita	0.14	0.14	0.14	18054	18054	18054
COL	Colombia	13693	Milan	0.14	0.14	0.14	8531	8531	8531
COL	Colombia	13694	Morelia	0.14	0.14	0.14	1581	1581	1581
COL	Colombia	13695	Puerto Rico	0.14	0.14	0.14	21044	21044	21044

COL	Colombia	13696	San Jose De Fragua	0.14	0.14	0.14	5990	5990	5990
COL	Colombia	13697	San Vicente Del Caguan	0.14	0.14	0.14	61424	61424	61424
COL	Colombia	13698	Solano	0.14	0.14	0.14	41889	41889	41889
COL	Colombia	13699	Valparaiso	0.14	0.14	0.14	10826	10826	10826
COL	Colombia	13700	Aguazul	0.50	0.50	0.50	13070	13070	13070
COL	Colombia	13701	Chameza	0.50	0.50	0.50	1467	1467	1467
COL	Colombia	13702	Hato Corozal	0.50	0.50	0.50	4197	4197	4197
COL	Colombia	13703	La Salina	0.50	0.50	0.50	396	396	396
COL	Colombia	13704	Mani	0.50	0.50	0.50	5128	5128	5128
COL	Colombia	13705	Monterrey	0.50	0.50	0.50	4685	4685	4685
COL	Colombia	13706	Nunchia	0.50	0.50	0.50	4315	4315	4315
COL	Colombia	13707	Orocue	0.50	0.50	0.50	3500	3500	3500
COL	Colombia	13708	Paz De Ariporo	0.50	0.50	0.50	11221	11221	11221
COL	Colombia	13709	Pore	0.50	0.50	0.50	2358	2358	2358
COL	Colombia	13710	Recetor	0.50	0.50	0.50	6389	6389	6389
COL	Colombia	13711	Sabanalarga	0.50	0.50	0.50	1610	1610	1610
COL	Colombia	13712	Sacama	0.50	0.50	0.50	400	400	400
COL	Colombia	13713	San Luis De Palenque	0.50	0.50	0.50	12470	12470	12470
COL	Colombia	13714	Tamara	0.50	0.50	0.50	2331	2331	2331
COL	Colombia	13715	Tauramena	0.50	0.50	0.50	11584	11584	11584
COL	Colombia	13716	Trinidad	0.50	0.50	0.50	6409	6409	6409
COL	Colombia	13717	Villanueva	0.50	0.50	0.50	5921	5921	5921
COL	Colombia	13718	Yopal	0.50	0.50	0.50	55111	55111	55111
COL	Colombia	13719	Almaguer	0.14	0.14	0.14	13550	13550	13550
COL	Colombia	13720	Argelia	0.14	0.14	0.14	8633	8633	8633
COL	Colombia	13721	Balboa	0.14	0.14	0.14	15424	15424	15424
COL	Colombia	13722	Bolivar	0.14	0.14	0.14	34449	34449	34449
COL	Colombia	13723	Buenos Aires	0.14	0.14	0.14	26723	26723	26723
COL	Colombia	13724	Cajibio	0.14	0.14	0.14	33225	33225	33225
COL	Colombia	13725	Caldono	0.14	0.14	0.14	38303	38303	38303
COL	Colombia	13726	Caloto	0.14	0.14	0.14	34202	34202	34202
COL	Colombia	13727	Corinto	0.14	0.14	0.14	21295	21295	21295
COL	Colombia	13728	El Tambo	0.14	0.14	0.14	34593	34593	34593

COL	Colombia	13729	Florencia	0.14	0.14	0.14	1349	1349	1349
COL	Colombia	13730	Gorgona (is.)	0.14	0.14	0.14	118	118	118
COL	Colombia	13731	Guapi	0.14	0.14	0.14	18898	18898	18898
COL	Colombia	13732	Guapi (is.)	0.14	0.14	0.14	17	17	17
COL	Colombia	13733	Inza	0.14	0.14	0.14	62487	62487	62487
COL	Colombia	13734	Jambalo	0.14	0.14	0.14	16378	16378	16378
COL	Colombia	13735	La Sierra	0.14	0.14	0.14	10139	10139	10139
COL	Colombia	13736	La Vega	0.14	0.14	0.14	30808	30808	30808
COL	Colombia	13737	Lopez De Micay	0.14	0.14	0.14	11688	11688	11688
COL	Colombia	13738	Mercaderes	0.14	0.14	0.14	12843	12843	12843
COL	Colombia	13739	Miranda	0.14	0.14	0.14	23643	23643	23643
COL	Colombia	13740	Morales	0.14	0.14	0.14	12620	12620	12620
COL	Colombia	13741	Padilla	0.14	0.14	0.14	12494	12494	12494
COL	Colombia	13742	Paez	0.14	0.14	0.14	21057	21057	21057
COL	Colombia	13743	Patia (el Bordo)	0.14	0.14	0.14	23536	23536	23536
COL	Colombia	13744	Piendamo	0.14	0.14	0.14	13327	13327	13327
COL	Colombia	13745	Popayan	0.14	0.14	0.14	148036	148036	148036
COL	Colombia	13746	Puerto Tejada	0.14	0.14	0.14	51454	51454	51454
COL	Colombia	13747	Purace	0.14	0.14	0.14	27736	27736	27736
COL	Colombia	13748	Rosas	0.14	0.14	0.14	6082	6082	6082
COL	Colombia	13749	San Sebastian	0.14	0.14	0.14	17486	17486	17486
COL	Colombia	13750	Santa Rosa	0.14	0.14	0.14	19497	19497	19497
COL	Colombia	13751	Santander De Quilichao	0.14	0.14	0.14	63201	63201	63201
COL	Colombia	13752	Silvia	0.14	0.14	0.14	24368	24368	24368
COL	Colombia	13753	Sotara	0.14	0.14	0.14	19876	19876	19876
COL	Colombia	13754	Suarez	0.14	0.14	0.14	5413	5413	5413
COL	Colombia	13755	Timbio	0.14	0.14	0.14	15433	15433	15433
COL	Colombia	13756	Timbiqui	0.14	0.14	0.14	10296	10296	10296
COL	Colombia	13757	Timbiqui (is.)	0.14	0.14	0.14	21	21	21
COL	Colombia	13758	Toribio	0.14	0.14	0.14	19193	19193	19193
COL	Colombia	13759	Aguachica	0.53	0.72	0.90	21872	6403	0
COL	Colombia	13760	Agustin Codazzi	0.53	0.72	0.90	11527	3374	0
COL	Colombia	13761	Astrea	0.53	0.72	0.90	4382	1283	0

COL	Colombia	13762	Becerril	0.53	0.72	0.90	5200	1522	0
COL	Colombia	13763	Bosconia	0.53	0.72	0.90	8882	2600	0
COL	Colombia	13764	Chimichagua	0.53	0.72	0.90	8806	2578	0
COL	Colombia	13765	Chiriguana	0.53	0.72	0.90	5805	1699	0
COL	Colombia	13766	Curumani	0.53	0.72	0.90	5462	1599	0
COL	Colombia	13767	El Copey	0.53	0.72	0.90	6909	2023	0
COL	Colombia	13768	El Paso	0.53	0.72	0.90	5863	1716	0
COL	Colombia	13769	Gamarra	0.53	0.72	0.90	4931	1444	0
COL	Colombia	13770	Gonzalez	0.53	0.72	0.90	1703	498	0
COL	Colombia	13771	La Gloria	0.53	0.72	0.90	3627	1062	0
COL	Colombia	13772	La Jagua De Iribico	0.53	0.72	0.90	4960	1452	0
COL	Colombia	13773	La Paz	0.53	0.72	0.90	8544	2501	0
COL	Colombia	13774	Manaure Balcon Del Cesar	0.53	0.72	0.90	2869	840	0
COL	Colombia	13775	Pailitas	0.53	0.72	0.90	3641	1066	0
COL	Colombia	13776	Pelaya	0.53	0.72	0.90	4187	1226	0
COL	Colombia	13777	Rio De Oro	0.53	0.72	0.90	2528	740	0
COL	Colombia	13778	San Alberto	0.53	0.72	0.90	7476	2189	0
COL	Colombia	13779	San Diego	0.53	0.72	0.90	1538	450	0
COL	Colombia	13780	San Martin	0.53	0.72	0.90	4584	1342	0
COL	Colombia	13781	Tamalameque	0.53	0.72	0.90	5197	1521	0
COL	Colombia	13782	Valledupar	0.53	0.72	0.90	117187	34304	0
COL	Colombia	13783	Acandi	0.14	0.14	0.14	6572	6572	6572
COL	Colombia	13784	Alto Baudo (pie De Pato)	0.14	0.14	0.14	21992	21992	21992
COL	Colombia	13785	Bagado	0.14	0.14	0.14	11261	11261	11261
COL	Colombia	13786	Bahia Solano (ciudad Muti	0.14	0.14	0.14	6035	6035	6035
COL	Colombia	13787	Bahia Solano (is.)	0.14	0.14	0.14	0	0	0
COL	Colombia	13789	Bajo Baudo (pizarro)	0.14	0.14	0.14	20071	20071	20071
COL	Colombia	13790	Bojaya (bella Vista)	0.14	0.14	0.14	5695	5695	5695
COL	Colombia	13791	Canton De San Pablo	0.14	0.14	0.14	7762	7762	7762
COL	Colombia	13792	Condoto	0.14	0.14	0.14	14540	14540	14540
COL	Colombia	13793	El Carmen	0.14	0.14	0.14	15922	15922	15922
COL	Colombia	13794	Istmina	0.14	0.14	0.14	11691	11691	11691
COL	Colombia	13795	Jurado	0.14	0.14	0.14	2075	2075	2075

COL	Colombia	13796	Jurado (is.)	0.14	0.14	0.14	1	1	1
COL	Colombia	13797	Litoral Del San Juan	0.14	0.14	0.14	24953	24953	24953
COL	Colombia	13798	Litoral Del San Juan (is.	0.14	0.14	0.14	4	4	4
COL	Colombia	13799	Lloro	0.14	0.14	0.14	8691	8691	8691
COL	Colombia	13800	Novita	0.14	0.14	0.14	5411	5411	5411
COL	Colombia	13801	Nuqui	0.14	0.14	0.14	6248	6248	6248
COL	Colombia	13802	Quibdo	0.14	0.14	0.14	107023	107023	107023
COL	Colombia	13803	Riosucio	0.14	0.14	0.14	21867	21867	21867
COL	Colombia	13804	San Jose Del Palmar	0.14	0.14	0.14	819	819	819
COL	Colombia	13805	Sipi	0.14	0.14	0.14	3669	3669	3669
COL	Colombia	13806	Tado	0.14	0.14	0.14	13653	13653	13653
COL	Colombia	13807	Unguia	0.14	0.14	0.14	8973	8973	8973
COL	Colombia	13808	Ayapel	0.14	0.14	0.14	43565	43565	43565
COL	Colombia	13809	Buenavista	0.14	0.14	0.14	16382	16382	16382
COL	Colombia	13810	Canalete	0.14	0.14	0.14	19536	19536	19536
COL	Colombia	13811	Cerete	0.14	0.14	0.14	48702	48702	48702
COL	Colombia	13812	Chima	0.14	0.14	0.14	14122	14122	14122
COL	Colombia	13813	Chinu	0.14	0.14	0.14	36819	36819	36819
COL	Colombia	13814	Cienaga De Oro	0.14	0.14	0.14	42496	42496	42496
COL	Colombia	13815	Lorica	0.14	0.14	0.14	83169	83169	83169
COL	Colombia	13816	Los Cordobas	0.14	0.14	0.14	12788	12788	12788
COL	Colombia	13817	Momil	0.14	0.14	0.14	8459	8459	8459
COL	Colombia	13819	Montelibano	0.14	0.14	0.14	67675	67675	67675
COL	Colombia	13820	Monteria	0.14	0.14	0.14	276818	276818	276818
COL	Colombia	13821	Mo¥itos	0.14	0.14	0.14	22262	22262	22262
COL	Colombia	13822	Planeta Rica	0.14	0.14	0.14	36435	36435	36435
COL	Colombia	13823	Pueblo Nuevo	0.14	0.14	0.14	27867	27867	27867
COL	Colombia	13824	Puerto Escondido	0.14	0.14	0.14	24028	24028	24028
COL	Colombia	13825	Puerto Libertador	0.14	0.14	0.14	32534	32534	32534
COL	Colombia	13826	Purisima	0.14	0.14	0.14	8069	8069	8069
COL	Colombia	13827	Sahagun	0.14	0.14	0.14	127932	127932	127932
COL	Colombia	13828	San Andres De Sotavento	0.14	0.14	0.14	41010	41010	41010
COL	Colombia	13829	San Antero	0.14	0.14	0.14	9150	9150	9150

COL	Colombia	13830	San Bernardo El Viento	0.14	0.14	0.14	28248	28248	28248
COL	Colombia	13832	San Carlos	0.14	0.14	0.14	18406	18406	18406
COL	Colombia	13833	San Pelayo	0.14	0.14	0.14	36771	36771	36771
COL	Colombia	13834	Tierralta	0.14	0.14	0.14	82362	82362	82362
COL	Colombia	13835	Valencia	0.14	0.14	0.14	29114	29114	29114
COL	Colombia	13836	Agua De Dios	0.14	0.14	0.14	5725	5725	5725
COL	Colombia	13837	Alban	0.14	0.14	0.14	3223	3223	3223
COL	Colombia	13838	Anapoima	0.14	0.14	0.14	6991	6991	6991
COL	Colombia	13839	Anolaima	0.14	0.14	0.14	7162	7162	7162
COL	Colombia	13840	Apulo (rafael Reyes)	0.14	0.14	0.14	4634	4634	4634
COL	Colombia	13841	Arbelaez	0.14	0.14	0.14	6352	6352	6352
COL	Colombia	13842	Beltran	0.14	0.14	0.14	3216	3216	3216
COL	Colombia	13843	Bituima	0.14	0.14	0.14	1437	1437	1437
COL	Colombia	13844	Bojaca	0.14	0.14	0.14	7474	7474	7474
COL	Colombia	13845	Cabrera	0.14	0.14	0.14	2947	2947	2947
COL	Colombia	13846	Cachipay	0.14	0.14	0.14	5979	5979	5979
COL	Colombia	13847	Cajica	0.14	0.14	0.14	41687	41687	41687
COL	Colombia	13848	Caparrapi	0.14	0.14	0.14	10350	10350	10350
COL	Colombia	13849	Caqueza	0.14	0.14	0.14	8136	8136	8136
COL	Colombia	13850	Carmen De Carupa	0.14	0.14	0.14	5355	5355	5355
COL	Colombia	13851	Chaguani	0.14	0.14	0.14	2288	2288	2288
COL	Colombia	13852	Chia	0.14	0.14	0.14	116972	116972	116972
COL	Colombia	13853	Chipaque	0.14	0.14	0.14	93961	93961	93961
COL	Colombia	13854	Choachi	0.14	0.14	0.14	56015	56015	56015
COL	Colombia	13855	Choconta	0.14	0.14	0.14	21005	21005	21005
COL	Colombia	13856	Cogua	0.14	0.14	0.14	28418	28418	28418
COL	Colombia	13857	Cota	0.14	0.14	0.14	23485	23485	23485
COL	Colombia	13858	Cucunuba	0.14	0.14	0.14	3519	3519	3519
COL	Colombia	13859	El Colegio	0.14	0.14	0.14	12172	12172	12172
COL	Colombia	13860	El Pe¥on	0.14	0.14	0.14	2892	2892	2892
COL	Colombia	13861	Facatativa	0.14	0.14	0.14	68959	68959	68959
COL	Colombia	13862	Fomeque	0.14	0.14	0.14	8494	8494	8494
COL	Colombia	13863	Fosca	0.14	0.14	0.14	3346	3346	3346

COL	Colombia	13864	Funza	0.14	0.14	0.14	52355	52355	52355
COL	Colombia	13865	Fuquene	0.14	0.14	0.14	6673	6673	6673
COL	Colombia	13866	Fusagasuga	0.14	0.14	0.14	79368	79368	79368
COL	Colombia	13867	Gachala	0.14	0.14	0.14	3677	3677	3677
COL	Colombia	13868	Gachancipa	0.14	0.14	0.14	14385	14385	14385
COL	Colombia	13869	Gacheta	0.14	0.14	0.14	8103	8103	8103
COL	Colombia	13870	Gama	0.14	0.14	0.14	1893	1893	1893
COL	Colombia	13871	Girardot	0.14	0.14	0.14	43115	43115	43115
COL	Colombia	13872	Guacheta	0.14	0.14	0.14	9168	9168	9168
COL	Colombia	13873	Guaduas	0.14	0.14	0.14	27371	27371	27371
COL	Colombia	13874	Guasca	0.14	0.14	0.14	11644	11644	11644
COL	Colombia	13875	Guataqui	0.14	0.14	0.14	1742	1742	1742
COL	Colombia	13876	Guatavita	0.14	0.14	0.14	3477	3477	3477
COL	Colombia	13877	Guayabal De Siquima	0.14	0.14	0.14	1906	1906	1906
COL	Colombia	13878	Guayabetal	0.14	0.14	0.14	4060	4060	4060
COL	Colombia	13879	Gutierrez	0.14	0.14	0.14	490374	490374	490374
COL	Colombia	13880	Jerusalen	0.14	0.14	0.14	1932	1932	1932
COL	Colombia	13881	Junin	0.14	0.14	0.14	4697	4697	4697
COL	Colombia	13882	La Calera	0.14	0.14	0.14	120277	120277	120277
COL	Colombia	13883	La Mesa	0.14	0.14	0.14	18875	18875	18875
COL	Colombia	13884	La Palma	0.14	0.14	0.14	4703	4703	4703
COL	Colombia	13885	La Pe¥a	0.14	0.14	0.14	4819	4819	4819
COL	Colombia	13886	La Vega	0.14	0.14	0.14	13032	13032	13032
COL	Colombia	13887	Lenguazaque	0.14	0.14	0.14	7622	7622	7622
COL	Colombia	13888	Macheta	0.14	0.14	0.14	6637	6637	6637
COL	Colombia	13889	Madrid	0.14	0.14	0.14	48452	48452	48452
COL	Colombia	13890	Manta	0.14	0.14	0.14	2048	2048	2048
COL	Colombia	13891	Medina	0.14	0.14	0.14	10253	10253	10253
COL	Colombia	13892	Mosquera	0.14	0.14	0.14	78884	78884	78884
COL	Colombia	13893	Nari¥o	0.14	0.14	0.14	8641	8641	8641
COL	Colombia	13894	Nemocon	0.14	0.14	0.14	15515	15515	15515
COL	Colombia	13895	Nilo	0.14	0.14	0.14	19842	19842	19842
COL	Colombia	13896	Nimaima	0.14	0.14	0.14	4766	4766	4766

COL	Colombia	13897	Nocaima	0.14	0.14	0.14	4006	4006	4006
COL	Colombia	13898	Pacho	0.14	0.14	0.14	15066	15066	15066
COL	Colombia	13899	Paime	0.14	0.14	0.14	2890	2890	2890
COL	Colombia	13900	Pandi	0.14	0.14	0.14	3684	3684	3684
COL	Colombia	13901	Paratebueno	0.14	0.14	0.14	5773	5773	5773
COL	Colombia	13902	Pasca	0.14	0.14	0.14	6029	6029	6029
COL	Colombia	13903	Puerto Salgar	0.14	0.14	0.14	19886	19886	19886
COL	Colombia	13904	Puli	0.14	0.14	0.14	1480	1480	1480
COL	Colombia	13905	Quebradanegra	0.14	0.14	0.14	3441	3441	3441
COL	Colombia	13906	Quetame	0.14	0.14	0.14	5573	5573	5573
COL	Colombia	13907	Quipile	0.14	0.14	0.14	3754	3754	3754
COL	Colombia	13908	Ricaurte	0.14	0.14	0.14	17833	17833	17833
COL	Colombia	13909	San Antonio Del Tequendam	0.14	0.14	0.14	8811	8811	8811
COL	Colombia	13910	San Bernardo	0.14	0.14	0.14	58420	58420	58420
COL	Colombia	13911	San Cayetano	0.14	0.14	0.14	4022	4022	4022
COL	Colombia	13912	San Francisco	0.14	0.14	0.14	11907	11907	11907
COL	Colombia	13913	San Juan De Rio Seco	0.14	0.14	0.14	5116	5116	5116
COL	Colombia	13914	Santafe De Bogota D.c.	0.14	0.14	0.14	4392570	4392570	4392570
COL	Colombia	13915	Sasaima	0.14	0.14	0.14	9838	9838	9838
COL	Colombia	13916	Sesquile	0.14	0.14	0.14	20942	20942	20942
COL	Colombia	13917	Sibate	0.14	0.14	0.14	22038	22038	22038
COL	Colombia	13918	Silvania	0.14	0.14	0.14	17416	17416	17416
COL	Colombia	13919	Simijaca	0.14	0.14	0.14	8406	8406	8406
COL	Colombia	13920	Soacha	0.14	0.14	0.14	236482	236482	236482
COL	Colombia	13921	Sopo	0.14	0.14	0.14	67118	67118	67118
COL	Colombia	13922	Subachoque	0.14	0.14	0.14	33112	33112	33112
COL	Colombia	13923	Suesca	0.14	0.14	0.14	15518	15518	15518
COL	Colombia	13924	Supata	0.14	0.14	0.14	3938	3938	3938
COL	Colombia	13925	Susa	0.14	0.14	0.14	7939	7939	7939
COL	Colombia	13926	Sutatausa	0.14	0.14	0.14	2404	2404	2404
COL	Colombia	13927	Tabio	0.14	0.14	0.14	16070	16070	16070
COL	Colombia	13928	Tausa	0.14	0.14	0.14	10225	10225	10225
COL	Colombia	13929	Tena	0.14	0.14	0.14	9347	9347	9347

COL	Colombia	13930	Tenjo	0.14	0.14	0.14	22479	22479	22479
COL	Colombia	13931	Tibacuy	0.14	0.14	0.14	5127	5127	5127
COL	Colombia	13932	Tibirita	0.14	0.14	0.14	1413	1413	1413
COL	Colombia	13933	Tocaima	0.14	0.14	0.14	19681	19681	19681
COL	Colombia	13934	Tocancipa	0.14	0.14	0.14	20845	20845	20845
COL	Colombia	13935	Topaipi	0.14	0.14	0.14	2728	2728	2728
COL	Colombia	13936	Ubala	0.14	0.14	0.14	4577	4577	4577
COL	Colombia	13937	Ubaque	0.14	0.14	0.14	18569	18569	18569
COL	Colombia	13938	Ubate	0.14	0.14	0.14	18112	18112	18112
COL	Colombia	13939	Une	0.14	0.14	0.14	21249	21249	21249
COL	Colombia	13940	Utica	0.14	0.14	0.14	2933	2933	2933
COL	Colombia	13941	Venecia (ospina Perez)	0.14	0.14	0.14	2118	2118	2118
COL	Colombia	13942	Vergara	0.14	0.14	0.14	4871	4871	4871
COL	Colombia	13943	Viani	0.14	0.14	0.14	1799	1799	1799
COL	Colombia	13944	Villagomez	0.14	0.14	0.14	2238	2238	2238
COL	Colombia	13945	Villapinzon	0.14	0.14	0.14	9869	9869	9869
COL	Colombia	13946	Villeta	0.14	0.14	0.14	12482	12482	12482
COL	Colombia	13947	Viota	0.14	0.14	0.14	8199	8199	8199
COL	Colombia	13948	Yacopi	0.14	0.14	0.14	10290	10290	10290
COL	Colombia	13949	Zipacon	0.14	0.14	0.14	4719	4719	4719
COL	Colombia	13950	Zipaquira	0.14	0.14	0.14	66663	66663	66663
COL	Colombia	13951	Barranco Minas	0.14	0.14	0.14	12750	12750	12750
COL	Colombia	13952	Cacahual	0.14	0.14	0.14	2538	2538	2538
COL	Colombia	13953	Inirida	0.14	0.14	0.14	20530	20530	20530
COL	Colombia	13954	La Guadalupe	0.14	0.14	0.14	289	289	289
COL	Colombia	13955	Pana Pana	0.14	0.14	0.14	2743	2743	2743
COL	Colombia	13956	Puerto Colombia	0.14	0.14	0.14	3808	3808	3808
COL	Colombia	13957	San Felipe	0.14	0.14	0.14	1872	1872	1872
COL	Colombia	13958	Barrancas	0.53	0.72	0.90	6551	1918	0
COL	Colombia	13959	El Molino	0.53	0.72	0.90	774	226	0
COL	Colombia	13960	Fonseca	0.53	0.72	0.90	8740	2558	0
COL	Colombia	13961	Hato Nuevo	0.53	0.72	0.90	5872	1719	0
COL	Colombia	13962	Maicao	0.53	0.72	0.90	45619	13354	0

COL	Colombia	13963	Manaure	0.53	0.72	0.90	42849	12543	0
COL	Colombia	13964	Riohacha	0.53	0.72	0.90	96883	28361	0
COL	Colombia	13965	San Juan Del Cesar	0.53	0.72	0.90	11470	3358	0
COL	Colombia	13966	Uribia	0.53	0.72	0.90	53343	15615	0
COL	Colombia	13967	Urumita	0.53	0.72	0.90	7029	2058	0
COL	Colombia	13968	Villanueva	0.53	0.72	0.90	7411	2169	0
COL	Colombia	13969	Calamar	0.14	0.14	0.14	7235	7235	7235
COL	Colombia	13970	El Retorno	0.14	0.14	0.14	9291	9291	9291
COL	Colombia	13971	Miraflores	0.14	0.14	0.14	7257	7257	7257
COL	Colombia	13972	San Jose Del Guaviare	0.14	0.14	0.14	59192	59192	59192
COL	Colombia	13973	Acevedo	0.14	0.14	0.14	15479	15479	15479
COL	Colombia	13974	Agrado	0.14	0.14	0.14	6657	6657	6657
COL	Colombia	13975	Aipe	0.14	0.14	0.14	30882	30882	30882
COL	Colombia	13976	Algeciras	0.14	0.14	0.14	18386	18386	18386
COL	Colombia	13977	Altamira	0.14	0.14	0.14	4897	4897	4897
COL	Colombia	13978	Baraya	0.14	0.14	0.14	3104	3104	3104
COL	Colombia	13979	Campo Alegre	0.14	0.14	0.14	15524	15524	15524
COL	Colombia	13980	Colombia	0.14	0.14	0.14	8023	8023	8023
COL	Colombia	13981	Elias	0.14	0.14	0.14	5455	5455	5455
COL	Colombia	13982	Garzon	0.14	0.14	0.14	44007	44007	44007
COL	Colombia	13983	Gigante	0.14	0.14	0.14	26767	26767	26767
COL	Colombia	13984	Guadalupe	0.14	0.14	0.14	27782	27782	27782
COL	Colombia	13985	Hobo	0.14	0.14	0.14	4239	4239	4239
COL	Colombia	13986	Iquira	0.14	0.14	0.14	9371	9371	9371
COL	Colombia	13987	Isnos	0.14	0.14	0.14	14685	14685	14685
COL	Colombia	13988	La Argentina	0.14	0.14	0.14	7394	7394	7394
COL	Colombia	13989	La Plata	0.14	0.14	0.14	40382	40382	40382
COL	Colombia	13990	Nataga	0.14	0.14	0.14	6321	6321	6321
COL	Colombia	13991	Neiva	0.14	0.14	0.14	190695	190695	190695
COL	Colombia	13992	Oporapa	0.14	0.14	0.14	10305	10305	10305
COL	Colombia	13993	Paicol	0.14	0.14	0.14	3057	3057	3057
COL	Colombia	13994	Palermo	0.14	0.14	0.14	16910	16910	16910
COL	Colombia	13995	Palestina	0.14	0.14	0.14	14030	14030	14030

COL	Colombia	13996	Pital	0.14	0.14	0.14	8260	8260	8260
COL	Colombia	13997	Pitalito	0.14	0.14	0.14	64574	64574	64574
COL	Colombia	13998	Rivera	0.14	0.14	0.14	26576	26576	26576
COL	Colombia	13999	Salado Blanco	0.14	0.14	0.14	10068	10068	10068
COL	Colombia	14000	San Agustin	0.14	0.14	0.14	28869	28869	28869
COL	Colombia	14001	Santa Maria	0.14	0.14	0.14	6800	6800	6800
COL	Colombia	14002	Suaza	0.14	0.14	0.14	10690	10690	10690
COL	Colombia	14003	Tarqui	0.14	0.14	0.14	11689	11689	11689
COL	Colombia	14004	Tello	0.14	0.14	0.14	20838	20838	20838
COL	Colombia	14005	Teruel	0.14	0.14	0.14	9201	9201	9201
COL	Colombia	14006	Tesalia	0.14	0.14	0.14	9215	9215	9215
COL	Colombia	14007	Timana	0.14	0.14	0.14	18504	18504	18504
COL	Colombia	14008	Villa Vieja	0.14	0.14	0.14	7189	7189	7189
COL	Colombia	14009	Yaguara	0.14	0.14	0.14	3837	3837	3837
COL	Colombia	14010	Aracataca	0.53	0.72	0.90	17105	5007	0
COL	Colombia	14011	Ariguani	0.53	0.72	0.90	10832	3171	0
COL	Colombia	14012	Cerro San Antonio	0.53	0.72	0.90	4425	1295	0
COL	Colombia	14013	Chivolo	0.53	0.72	0.90	4867	1425	0
COL	Colombia	14014	Cienaga	0.53	0.72	0.90	43931	12860	0
COL	Colombia	14015	El Banco	0.53	0.72	0.90	12215	3576	0
COL	Colombia	14016	El Pi¥on	0.53	0.72	0.90	5150	1507	0
COL	Colombia	14017	Fundacion	0.53	0.72	0.90	15558	4554	0
COL	Colombia	14018	Guamal	0.53	0.72	0.90	7269	2128	0
COL	Colombia	14019	Pedraza	0.53	0.72	0.90	3650	1068	0
COL	Colombia	14020	Pivijay	0.53	0.72	0.90	13008	3808	0
COL	Colombia	14021	Plato	0.53	0.72	0.90	20382	5966	0
COL	Colombia	14022	Pueblo Viejo	0.53	0.72	0.90	6832	2000	0
COL	Colombia	14023	Remolino	0.53	0.72	0.90	2964	868	0
COL	Colombia	14024	Salamina	0.53	0.72	0.90	2016	590	0
COL	Colombia	14025	San Sebastian De Buenavis	0.53	0.72	0.90	4578	1340	0
COL	Colombia	14026	San Zenon	0.53	0.72	0.90	2980	872	0
COL	Colombia	14027	Santa Ana	0.53	0.72	0.90	15400	4508	0
COL	Colombia	14028	Santa Marta (dist. Esp.)	0.53	0.72	0.90	127588	37349	0

COL	Colombia	14030	Tenerife	0.53	0.72	0.90	4929	1443	0
COL	Colombia	14031	Acacias	0.14	0.14	0.14	38377	38377	38377
COL	Colombia	14032	Barranca De Upia	0.14	0.14	0.14	2068	2068	2068
COL	Colombia	14033	Cabuyaro	0.14	0.14	0.14	2615	2615	2615
COL	Colombia	14034	Castilla La Nueva	0.14	0.14	0.14	7001	7001	7001
COL	Colombia	14035	Cubarral	0.14	0.14	0.14	79535	79535	79535
COL	Colombia	14036	Cumaral	0.14	0.14	0.14	6451	6451	6451
COL	Colombia	14037	El Calvario	0.14	0.14	0.14	2068	2068	2068
COL	Colombia	14038	El Castillo	0.14	0.14	0.14	6084	6084	6084
COL	Colombia	14039	El Dorado	0.14	0.14	0.14	862	862	862
COL	Colombia	14040	Fuente De Oro	0.14	0.14	0.14	13188	13188	13188
COL	Colombia	14041	Granada	0.14	0.14	0.14	24141	24141	24141
COL	Colombia	14042	Guamal	0.14	0.14	0.14	16394	16394	16394
COL	Colombia	14043	La Macarena	0.14	0.14	0.14	29214	29214	29214
COL	Colombia	14044	La Uribe	0.14	0.14	0.14	10856	10856	10856
COL	Colombia	14045	Lejanias	0.14	0.14	0.14	3960	3960	3960
COL	Colombia	14046	Mapiripan	0.14	0.14	0.14	15800	15800	15800
COL	Colombia	14047	Mesetas	0.14	0.14	0.14	5218	5218	5218
COL	Colombia	14048	Puerto Concordia	0.14	0.14	0.14	8930	8930	8930
COL	Colombia	14049	Puerto Gaitan	0.14	0.14	0.14	11630	11630	11630
COL	Colombia	14050	Puerto Lleras	0.14	0.14	0.14	5342	5342	5342
COL	Colombia	14051	Puerto Lopez	0.14	0.14	0.14	20944	20944	20944
COL	Colombia	14052	Puerto Rico	0.14	0.14	0.14	14745	14745	14745
COL	Colombia	14053	Restrepo	0.14	0.14	0.14	46466	46466	46466
COL	Colombia	14054	San Carlos De Guaroa	0.14	0.14	0.14	12931	12931	12931
COL	Colombia	14055	San Juan De Arama	0.14	0.14	0.14	3403	3403	3403
COL	Colombia	14056	San Juanito	0.14	0.14	0.14	1312	1312	1312
COL	Colombia	14057	San Martin	0.14	0.14	0.14	25620	25620	25620
COL	Colombia	14058	Villavicencio	0.14	0.14	0.14	266771	266771	266771
COL	Colombia	14059	Vistahermosa	0.14	0.14	0.14	14025	14025	14025
COL	Colombia	14060	Alban	0.14	0.14	0.14	7641	7641	7641
COL	Colombia	14061	Aldana	0.14	0.14	0.14	4010	4010	4010
COL	Colombia	14062	Ancuya	0.14	0.14	0.14	4471	4471	4471

COL	Colombia	14063	Arboleda	0.14	0.14	0.14	4207	4207	4207
COL	Colombia	14064	Barbacoas	0.14	0.14	0.14	31427	31427	31427
COL	Colombia	14065	Belen	0.14	0.14	0.14	3075	3075	3075
COL	Colombia	14066	Buesaco	0.14	0.14	0.14	19661	19661	19661
COL	Colombia	14067	Chachagui	0.14	0.14	0.14	2817	2817	2817
COL	Colombia	14068	Colon	0.14	0.14	0.14	4560	4560	4560
COL	Colombia	14069	Consaca	0.14	0.14	0.14	5640	5640	5640
COL	Colombia	14070	Contadero	0.14	0.14	0.14	5179	5179	5179
COL	Colombia	14071	Cordoba	0.14	0.14	0.14	7997	7997	7997
COL	Colombia	14072	Cuaspud	0.14	0.14	0.14	4044	4044	4044
COL	Colombia	14073	Cumbal	0.14	0.14	0.14	19432	19432	19432
COL	Colombia	14074	Cumbitara	0.14	0.14	0.14	6192	6192	6192
COL	Colombia	14075	El Charco	0.14	0.14	0.14	15740	15740	15740
COL	Colombia	14076	El Charco (is.)	0.14	0.14	0.14	326	326	326
COL	Colombia	14077	El Rosario	0.14	0.14	0.14	3751	3751	3751
COL	Colombia	14078	El Tablon	0.14	0.14	0.14	28611	28611	28611
COL	Colombia	14079	El Tambo	0.14	0.14	0.14	7924	7924	7924
COL	Colombia	14080	Francisco Pizarro	0.14	0.14	0.14	15607	15607	15607
COL	Colombia	14081	Funes	0.14	0.14	0.14	8123	8123	8123
COL	Colombia	14082	Guachucal	0.14	0.14	0.14	4912	4912	4912
COL	Colombia	14083	Guaitarilla	0.14	0.14	0.14	9196	9196	9196
COL	Colombia	14084	Gualmatan	0.14	0.14	0.14	4639	4639	4639
COL	Colombia	14085	Iles	0.14	0.14	0.14	7072	7072	7072
COL	Colombia	14086	Imues	0.14	0.14	0.14	4406	4406	4406
COL	Colombia	14087	Ipiales	0.14	0.14	0.14	88872	88872	88872
COL	Colombia	14088	La Cruz	0.14	0.14	0.14	16774	16774	16774
COL	Colombia	14089	La Florida	0.14	0.14	0.14	7134	7134	7134
COL	Colombia	14090	La Llanada	0.14	0.14	0.14	6456	6456	6456
COL	Colombia	14091	La Tola	0.14	0.14	0.14	6465	6465	6465
COL	Colombia	14092	La Union	0.14	0.14	0.14	11669	11669	11669
COL	Colombia	14093	Leiva	0.14	0.14	0.14	10851	10851	10851
COL	Colombia	14094	Linares	0.14	0.14	0.14	3349	3349	3349
COL	Colombia	14095	Los Andes	0.14	0.14	0.14	6306	6306	6306

COL	Colombia	14096	Magui	0.14	0.14	0.14	37902	37902	37902
COL	Colombia	14097	Mallama	0.14	0.14	0.14	6968	6968	6968
COL	Colombia	14098	Mosquera	0.14	0.14	0.14	7458	7458	7458
COL	Colombia	14099	Mosquera (is.)	0.14	0.14	0.14	720	720	720
COL	Colombia	14100	Olaya Herrera	0.14	0.14	0.14	18076	18076	18076
COL	Colombia	14101	Ospina	0.14	0.14	0.14	6577	6577	6577
COL	Colombia	14102	Pasto	0.14	0.14	0.14	263905	263905	263905
COL	Colombia	14103	Policarpa	0.14	0.14	0.14	8353	8353	8353
COL	Colombia	14104	Potosi	0.14	0.14	0.14	8939	8939	8939
COL	Colombia	14105	Providencia	0.14	0.14	0.14	9439	9439	9439
COL	Colombia	14106	Puerres	0.14	0.14	0.14	5331	5331	5331
COL	Colombia	14107	Pupiales	0.14	0.14	0.14	12323	12323	12323
COL	Colombia	14108	Ricaurte	0.14	0.14	0.14	24771	24771	24771
COL	Colombia	14109	Roberto Payan	0.14	0.14	0.14	17013	17013	17013
COL	Colombia	14110	Samaniego	0.14	0.14	0.14	19739	19739	19739
COL	Colombia	14111	San Bernardo	0.14	0.14	0.14	7695	7695	7695
COL	Colombia	14112	San Lorenzo	0.14	0.14	0.14	12707	12707	12707
COL	Colombia	14113	San Pablo	0.14	0.14	0.14	8060	8060	8060
COL	Colombia	14114	San Pedro De Cartago	0.14	0.14	0.14	4198	4198	4198
COL	Colombia	14115	Sandona	0.14	0.14	0.14	13296	13296	13296
COL	Colombia	14116	Santa Barbara	0.14	0.14	0.14	12330	12330	12330
COL	Colombia	14117	Santa Cruz	0.14	0.14	0.14	39067	39067	39067
COL	Colombia	14118	Sapuyes	0.14	0.14	0.14	6479	6479	6479
COL	Colombia	14119	Taminango	0.14	0.14	0.14	11159	11159	11159
COL	Colombia	14120	Tangua	0.14	0.14	0.14	6219	6219	6219
COL	Colombia	14121	Tumaco	0.14	0.14	0.14	88936	88936	88936
COL	Colombia	14122	Tumaco (is.)	0.14	0.14	0.14	214	214	214
COL	Colombia	14123	Tuquerres	0.14	0.14	0.14	20283	20283	20283
COL	Colombia	14124	Yacuanquer	0.14	0.14	0.14	5823	5823	5823
COL	Colombia	14125	Abrego	0.54	0.54	0.54	12220	12220	12220
COL	Colombia	14126	Arboledas	0.54	0.54	0.54	2014	2014	2014
COL	Colombia	14127	Bochalema	0.54	0.54	0.54	1523	1523	1523
COL	Colombia	14128	Bucarasica	0.54	0.54	0.54	877	877	877

COL	Colombia	14129	Cachira	0.54	0.54	0.54	3542	3542	3542
COL	Colombia	14130	Cacota	0.54	0.54	0.54	447	447	447
COL	Colombia	14131	Chinacota	0.54	0.54	0.54	3114	3114	3114
COL	Colombia	14132	Chitaga	0.54	0.54	0.54	2655	2655	2655
COL	Colombia	14133	Convencion	0.54	0.54	0.54	4115	4115	4115
COL	Colombia	14134	Cucuta	0.54	0.54	0.54	137750	137750	137750
COL	Colombia	14135	Cucutilla	0.54	0.54	0.54	2907	2907	2907
COL	Colombia	14136	Durania	0.54	0.54	0.54	835	835	835
COL	Colombia	14137	El Carmen	0.54	0.54	0.54	7387	7387	7387
COL	Colombia	14138	El Tarra	0.54	0.54	0.54	2366	2366	2366
COL	Colombia	14139	El Zulia	0.54	0.54	0.54	4964	4964	4964
COL	Colombia	14140	Gramalote	0.54	0.54	0.54	1205	1205	1205
COL	Colombia	14141	Hacari	0.54	0.54	0.54	2204	2204	2204
COL	Colombia	14142	Herran	0.54	0.54	0.54	1496	1496	1496
COL	Colombia	14143	La Esperanza	0.54	0.54	0.54	2507	2507	2507
COL	Colombia	14144	La Playa	0.54	0.54	0.54	3550	3550	3550
COL	Colombia	14145	Labateca	0.54	0.54	0.54	3168	3168	3168
COL	Colombia	14146	Los Patios	0.54	0.54	0.54	7948	7948	7948
COL	Colombia	14147	Lourdes	0.54	0.54	0.54	779	779	779
COL	Colombia	14148	Mutiscua	0.54	0.54	0.54	718	718	718
COL	Colombia	14149	Oca¥a	0.54	0.54	0.54	16232	16232	16232
COL	Colombia	14150	Pamplona	0.54	0.54	0.54	8106	8106	8106
COL	Colombia	14151	Pamplonita	0.54	0.54	0.54	3610	3610	3610
COL	Colombia	14152	Puerto Santander	0.54	0.54	0.54	18299	18299	18299
COL	Colombia	14153	Ragonvalia	0.54	0.54	0.54	1840	1840	1840
COL	Colombia	14154	Salazar	0.54	0.54	0.54	1919	1919	1919
COL	Colombia	14155	San Calixto	0.54	0.54	0.54	3817	3817	3817
COL	Colombia	14156	San Cayetano	0.54	0.54	0.54	1169	1169	1169
COL	Colombia	14157	Santiago	0.54	0.54	0.54	682	682	682
COL	Colombia	14158	Sardinata	0.54	0.54	0.54	5364	5364	5364
COL	Colombia	14159	Silos	0.54	0.54	0.54	1717	1717	1717
COL	Colombia	14160	Teorama	0.54	0.54	0.54	5610	5610	5610
COL	Colombia	14161	Tibu	0.54	0.54	0.54	15330	15330	15330

COL	Colombia	14162	Toledo	0.54	0.54	0.54	5042	5042	5042
COL	Colombia	14163	Villa Caro	0.54	0.54	0.54	1434	1434	1434
COL	Colombia	14164	Villa Del Rosario	0.54	0.54	0.54	39260	39260	39260
COL	Colombia	14165	Colon	0.14	0.14	0.14	5429	5429	5429
COL	Colombia	14166	Mocoa	0.14	0.14	0.14	23006	23006	23006
COL	Colombia	14167	Orito	0.14	0.14	0.14	43618	43618	43618
COL	Colombia	14168	Puerto Asis	0.14	0.14	0.14	41365	41365	41365
COL	Colombia	14169	Puerto Caicedo	0.14	0.14	0.14	18926	18926	18926
COL	Colombia	14170	Puerto Guzman	0.14	0.14	0.14	14850	14850	14850
COL	Colombia	14171	Puerto Leguizamo	0.14	0.14	0.14	9590	9590	9590
COL	Colombia	14172	San Francisco	0.14	0.14	0.14	16543	16543	16543
COL	Colombia	14173	Santiago	0.14	0.14	0.14	30398	30398	30398
COL	Colombia	14174	Sibundoy	0.14	0.14	0.14	3959	3959	3959
COL	Colombia	14175	Valle Del Guamuez (la Hor	0.14	0.14	0.14	54504	54504	54504
COL	Colombia	14176	Villagarzon	0.14	0.14	0.14	10222	10222	10222
COL	Colombia	14177	Armenia	0.14	0.14	0.14	141946	141946	141946
COL	Colombia	14178	Buenavista	0.14	0.14	0.14	8962	8962	8962
COL	Colombia	14179	Calarca	0.14	0.14	0.14	65780	65780	65780
COL	Colombia	14180	Circasia	0.14	0.14	0.14	11903	11903	11903
COL	Colombia	14181	Cordoba	0.14	0.14	0.14	2702	2702	2702
COL	Colombia	14182	Filandia	0.14	0.14	0.14	19467	19467	19467
COL	Colombia	14183	Genova	0.14	0.14	0.14	5714	5714	5714
COL	Colombia	14184	La Tebaida	0.14	0.14	0.14	21624	21624	21624
COL	Colombia	14185	Montenegro	0.14	0.14	0.14	40254	40254	40254
COL	Colombia	14186	Pijao	0.14	0.14	0.14	4908	4908	4908
COL	Colombia	14187	Quimbaya	0.14	0.14	0.14	11507	11507	11507
COL	Colombia	14188	Salento	0.14	0.14	0.14	39620	39620	39620
COL	Colombia	14189	Apia	0.14	0.14	0.14	8152	8152	8152
COL	Colombia	14190	Balboa	0.14	0.14	0.14	5629	5629	5629
COL	Colombia	14191	Belen De Umbria	0.14	0.14	0.14	13568	13568	13568
COL	Colombia	14192	Dos Quebradas	0.14	0.14	0.14	81165	81165	81165
COL	Colombia	14193	Guatica	0.14	0.14	0.14	5230	5230	5230
COL	Colombia	14194	La Celia	0.14	0.14	0.14	1882	1882	1882

COL	Colombia	14195	La Virginia	0.14	0.14	0.14	10151	10151	10151
COL	Colombia	14196	Marsella	0.14	0.14	0.14	31328	31328	31328
COL	Colombia	14197	Mistrato	0.14	0.14	0.14	8216	8216	8216
COL	Colombia	14198	Pereira	0.14	0.14	0.14	273484	273484	273484
COL	Colombia	14199	Pueblo Rico	0.14	0.14	0.14	10338	10338	10338
COL	Colombia	14200	Quinchia	0.14	0.14	0.14	17041	17041	17041
COL	Colombia	14201	Santa Rosa De Cabal	0.14	0.14	0.14	116669	116669	116669
COL	Colombia	14202	Santuario	0.14	0.14	0.14	7709	7709	7709
COL	Colombia	14206	Aguada	0.14	0.14	0.14	913	913	913
COL	Colombia	14207	Albania	0.14	0.14	0.14	5199	5199	5199
COL	Colombia	14208	Aratoca	0.14	0.14	0.14	4933	4933	4933
COL	Colombia	14209	Barbosa	0.14	0.14	0.14	10429	10429	10429
COL	Colombia	14210	Barichara	0.14	0.14	0.14	2709	2709	2709
COL	Colombia	14211	Barrancabermeja	0.14	0.14	0.14	114832	114832	114832
COL	Colombia	14212	Betulia	0.14	0.14	0.14	3745	3745	3745
COL	Colombia	14213	Bolivar	0.14	0.14	0.14	20051	20051	20051
COL	Colombia	14214	Bucaramanga	0.14	0.14	0.14	287380	287380	287380
COL	Colombia	14215	Cabrera	0.14	0.14	0.14	8424	8424	8424
COL	Colombia	14216	California	0.14	0.14	0.14	1251	1251	1251
COL	Colombia	14217	Capitanejo	0.14	0.14	0.14	3408	3408	3408
COL	Colombia	14218	Carcasi	0.14	0.14	0.14	3196	3196	3196
COL	Colombia	14219	Cepita	0.14	0.14	0.14	3411	3411	3411
COL	Colombia	14220	Cerrito	0.14	0.14	0.14	4001	4001	4001
COL	Colombia	14221	Charala	0.14	0.14	0.14	8361	8361	8361
COL	Colombia	14222	Charta	0.14	0.14	0.14	10892	10892	10892
COL	Colombia	14223	Chima	0.14	0.14	0.14	2323	2323	2323
COL	Colombia	14224	Chipata	0.14	0.14	0.14	3067	3067	3067
COL	Colombia	14225	Cimitarra	0.14	0.14	0.14	27339	27339	27339
COL	Colombia	14226	Concepcion	0.14	0.14	0.14	8944	8944	8944
COL	Colombia	14227	Confines	0.14	0.14	0.14	1466	1466	1466
COL	Colombia	14228	Contratacion	0.14	0.14	0.14	1398	1398	1398
COL	Colombia	14229	Coromoro	0.14	0.14	0.14	4452	4452	4452
COL	Colombia	14230	Curiti	0.14	0.14	0.14	11098	11098	11098

COL	Colombia	14231	El Carmen	0.14	0.14	0.14	15076	15076	15076
COL	Colombia	14232	El Guacamayo	0.14	0.14	0.14	1154	1154	1154
COL	Colombia	14233	El Pe¥on	0.14	0.14	0.14	4720	4720	4720
COL	Colombia	14234	El Playon	0.14	0.14	0.14	11900	11900	11900
COL	Colombia	14235	Encino	0.14	0.14	0.14	12965	12965	12965
COL	Colombia	14236	Enciso	0.14	0.14	0.14	5749	5749	5749
COL	Colombia	14237	Florian	0.14	0.14	0.14	4053	4053	4053
COL	Colombia	14238	Floridablanca	0.14	0.14	0.14	153079	153079	153079
COL	Colombia	14239	Galan	0.14	0.14	0.14	1690	1690	1690
COL	Colombia	14240	Gambita	0.14	0.14	0.14	4611	4611	4611
COL	Colombia	14241	Giron	0.14	0.14	0.14	96208	96208	96208
COL	Colombia	14242	Guaca	0.14	0.14	0.14	4407	4407	4407
COL	Colombia	14243	Guadalupe	0.14	0.14	0.14	3111	3111	3111
COL	Colombia	14244	Guapota	0.14	0.14	0.14	1396	1396	1396
COL	Colombia	14245	Guavata	0.14	0.14	0.14	2202	2202	2202
COL	Colombia	14246	Guepsa	0.14	0.14	0.14	2360	2360	2360
COL	Colombia	14247	Hato	0.14	0.14	0.14	1409	1409	1409
COL	Colombia	14248	Jesus Maria	0.14	0.14	0.14	2579	2579	2579
COL	Colombia	14249	Jordan	0.14	0.14	0.14	1077	1077	1077
COL	Colombia	14250	La Belleza	0.14	0.14	0.14	6073	6073	6073
COL	Colombia	14251	La Paz	0.14	0.14	0.14	6249	6249	6249
COL	Colombia	14252	Landazuri	0.14	0.14	0.14	8918	8918	8918
COL	Colombia	14253	Lebrija	0.14	0.14	0.14	34121	34121	34121
COL	Colombia	14254	Los Santos	0.14	0.14	0.14	8249	8249	8249
COL	Colombia	14255	Macaravita	0.14	0.14	0.14	1961	1961	1961
COL	Colombia	14256	Malaga	0.14	0.14	0.14	4467	4467	4467
COL	Colombia	14257	Matanza	0.14	0.14	0.14	45527	45527	45527
COL	Colombia	14258	Mogotes	0.14	0.14	0.14	9228	9228	9228
COL	Colombia	14259	Molagavita	0.14	0.14	0.14	2831	2831	2831
COL	Colombia	14260	Ocamonte	0.14	0.14	0.14	1752	1752	1752
COL	Colombia	14261	Oiba	0.14	0.14	0.14	6546	6546	6546
COL	Colombia	14262	Onzaga	0.14	0.14	0.14	2377	2377	2377
COL	Colombia	14263	Palmar	0.14	0.14	0.14	877	877	877

COL	Colombia	14264	Palmas Del Socorro	0.14	0.14	0.14	2062	2062	2062
COL	Colombia	14265	Paramo	0.14	0.14	0.14	3753	3753	3753
COL	Colombia	14266	Piedecuesta	0.14	0.14	0.14	93832	93832	93832
COL	Colombia	14267	Pinchote	0.14	0.14	0.14	3807	3807	3807
COL	Colombia	14268	Puente Nacional	0.14	0.14	0.14	8099	8099	8099
COL	Colombia	14269	Puerto Parra	0.14	0.14	0.14	6776	6776	6776
COL	Colombia	14270	Puerto Wilches	0.14	0.14	0.14	30633	30633	30633
COL	Colombia	14271	Rionegro	0.14	0.14	0.14	20718	20718	20718
COL	Colombia	14272	Sabana De Torres	0.14	0.14	0.14	11055	11055	11055
COL	Colombia	14273	San Andres	0.14	0.14	0.14	4458	4458	4458
COL	Colombia	14274	San Benito	0.14	0.14	0.14	1808	1808	1808
COL	Colombia	14275	San Gil	0.14	0.14	0.14	24569	24569	24569
COL	Colombia	14276	San Joaquin	0.14	0.14	0.14	1455	1455	1455
COL	Colombia	14277	San Jose Miranda	0.14	0.14	0.14	1614	1614	1614
COL	Colombia	14278	San Miguel	0.14	0.14	0.14	1963	1963	1963
COL	Colombia	14279	San Vicente De Chucuri	0.14	0.14	0.14	22148	22148	22148
COL	Colombia	14280	Santa Barbara	0.14	0.14	0.14	16391	16391	16391
COL	Colombia	14281	Santa Helena De Opon	0.14	0.14	0.14	3370	3370	3370
COL	Colombia	14282	Simacota	0.14	0.14	0.14	6321	6321	6321
COL	Colombia	14283	Socorro	0.14	0.14	0.14	17442	17442	17442
COL	Colombia	14284	Suaita	0.14	0.14	0.14	7900	7900	7900
COL	Colombia	14285	Sucre	0.14	0.14	0.14	4341	4341	4341
COL	Colombia	14286	Surata	0.14	0.14	0.14	2493	2493	2493
COL	Colombia	14287	Tona	0.14	0.14	0.14	63770	63770	63770
COL	Colombia	14288	Valle De San Jose	0.14	0.14	0.14	2497	2497	2497
COL	Colombia	14289	Velez	0.14	0.14	0.14	7675	7675	7675
COL	Colombia	14290	Vetas	0.14	0.14	0.14	2385	2385	2385
COL	Colombia	14291	Villanueva	0.14	0.14	0.14	3634	3634	3634
COL	Colombia	14292	Zapatoca	0.14	0.14	0.14	5891	5891	5891
COL	Colombia	14293	Buenavista	0.14	0.14	0.14	6206	6206	6206
COL	Colombia	14294	Caimito	0.14	0.14	0.14	7777	7777	7777
COL	Colombia	14295	Chalan	0.14	0.14	0.14	3013	3013	3013
COL	Colombia	14296	Coloso (ricaurte)	0.14	0.14	0.14	3638	3638	3638

COL	Colombia	14297	Corozal	0.14	0.14	0.14	45580	45580	45580
COL	Colombia	14298	Galeras	0.14	0.14	0.14	11378	11378	11378
COL	Colombia	14299	Guaranda	0.14	0.14	0.14	15004	15004	15004
COL	Colombia	14300	La Union	0.14	0.14	0.14	9837	9837	9837
COL	Colombia	14301	Los Palmitos	0.14	0.14	0.14	11948	11948	11948
COL	Colombia	14302	Majagual	0.14	0.14	0.14	12669	12669	12669
COL	Colombia	14303	Morroa	0.14	0.14	0.14	10300	10300	10300
COL	Colombia	14304	Ovejas	0.14	0.14	0.14	14517	14517	14517
COL	Colombia	14305	Palmito	0.14	0.14	0.14	18794	18794	18794
COL	Colombia	14306	Sampues	0.14	0.14	0.14	26074	26074	26074
COL	Colombia	14307	San Benito Abad	0.14	0.14	0.14	20435	20435	20435
COL	Colombia	14308	San Juan De Betulia	0.14	0.14	0.14	12676	12676	12676
COL	Colombia	14309	San Marcos	0.14	0.14	0.14	41472	41472	41472
COL	Colombia	14310	San Onofre	0.14	0.14	0.14	31052	31052	31052
COL	Colombia	14312	San Pedro	0.14	0.14	0.14	9527	9527	9527
COL	Colombia	14313	Since	0.14	0.14	0.14	17838	17838	17838
COL	Colombia	14314	Sincelejo	0.14	0.14	0.14	172247	172247	172247
COL	Colombia	14315	Sucre	0.14	0.14	0.14	12734	12734	12734
COL	Colombia	14316	Tolu	0.14	0.14	0.14	30603	30603	30603
COL	Colombia	14317	Toluviejo	0.14	0.14	0.14	11741	11741	11741
COL	Colombia	14318	Alpujarra	0.14	0.14	0.14	3754	3754	3754
COL	Colombia	14319	Alvarado	0.14	0.14	0.14	19650	19650	19650
COL	Colombia	14320	Ambalema	0.14	0.14	0.14	5697	5697	5697
COL	Colombia	14321	Anzoategui	0.14	0.14	0.14	41054	41054	41054
COL	Colombia	14322	Armero (guayabal)	0.14	0.14	0.14	11981	11981	11981
COL	Colombia	14323	Ataco	0.14	0.14	0.14	14389	14389	14389
COL	Colombia	14324	Cajamarca	0.14	0.14	0.14	18822	18822	18822
COL	Colombia	14325	Carmen De Apicala	0.14	0.14	0.14	5206	5206	5206
COL	Colombia	14326	Casabianca	0.14	0.14	0.14	4413	4413	4413
COL	Colombia	14327	Chaparral	0.14	0.14	0.14	40950	40950	40950
COL	Colombia	14328	Coello	0.14	0.14	0.14	22288	22288	22288
COL	Colombia	14329	Coyaima	0.14	0.14	0.14	15391	15391	15391
COL	Colombia	14330	Cunday	0.14	0.14	0.14	8393	8393	8393

COL	Colombia	14331	Dolores	0.14	0.14	0.14	5547	5547	5547
COL	Colombia	14332	Espinal	0.14	0.14	0.14	41104	41104	41104
COL	Colombia	14333	Falan	0.14	0.14	0.14	11476	11476	11476
COL	Colombia	14334	Flandes	0.14	0.14	0.14	19205	19205	19205
COL	Colombia	14335	Fresno	0.14	0.14	0.14	16818	16818	16818
COL	Colombia	14336	Guamo	0.14	0.14	0.14	21908	21908	21908
COL	Colombia	14337	Herveo	0.14	0.14	0.14	13411	13411	13411
COL	Colombia	14338	Honda	0.14	0.14	0.14	14327	14327	14327
COL	Colombia	14339	Ibague	0.14	0.14	0.14	281191	281191	281191
COL	Colombia	14340	Icononzo	0.14	0.14	0.14	12594	12594	12594
COL	Colombia	14341	Lerida	0.14	0.14	0.14	15994	15994	15994
COL	Colombia	14342	Libano	0.14	0.14	0.14	16487	16487	16487
COL	Colombia	14343	Mariquita	0.14	0.14	0.14	21648	21648	21648
COL	Colombia	14344	Melgar	0.14	0.14	0.14	16498	16498	16498
COL	Colombia	14345	Murillo	0.14	0.14	0.14	2929	2929	2929
COL	Colombia	14346	Natagaima	0.14	0.14	0.14	16732	16732	16732
COL	Colombia	14347	Ortega	0.14	0.14	0.14	19858	19858	19858
COL	Colombia	14348	Piedras	0.14	0.14	0.14	8869	8869	8869
COL	Colombia	14349	Planadas	0.14	0.14	0.14	15987	15987	15987
COL	Colombia	14350	Prado	0.14	0.14	0.14	4547	4547	4547
COL	Colombia	14351	Purificacion	0.14	0.14	0.14	18985	18985	18985
COL	Colombia	14352	Rioblanco	0.14	0.14	0.14	24551	24551	24551
COL	Colombia	14353	Roncesvalles	0.14	0.14	0.14	3798	3798	3798
COL	Colombia	14354	Rovira	0.14	0.14	0.14	37002	37002	37002
COL	Colombia	14355	Salda¥a	0.14	0.14	0.14	5319	5319	5319
COL	Colombia	14356	San Antonio	0.14	0.14	0.14	7909	7909	7909
COL	Colombia	14357	San Luis	0.14	0.14	0.14	12171	12171	12171
COL	Colombia	14358	Santa Isabel	0.14	0.14	0.14	12080	12080	12080
COL	Colombia	14359	Suarez	0.14	0.14	0.14	7824	7824	7824
COL	Colombia	14360	Valle De San Juan	0.14	0.14	0.14	3979	3979	3979
COL	Colombia	14361	Venadillo	0.14	0.14	0.14	9159	9159	9159
COL	Colombia	14362	Villa Hermosa	0.14	0.14	0.14	5311	5311	5311
COL	Colombia	14363	Villarrica	0.14	0.14	0.14	4483	4483	4483

COL	Colombia	14364	Alcala	0.14	0.14	0.14	22799	22799	22799
COL	Colombia	14365	Andalucia	0.14	0.14	0.14	24041	24041	24041
COL	Colombia	14366	Ansermanuevo	0.14	0.14	0.14	10635	10635	10635
COL	Colombia	14367	Argelia	0.14	0.14	0.14	4771	4771	4771
COL	Colombia	14368	Bolivar	0.14	0.14	0.14	9989	9989	9989
COL	Colombia	14369	Buenaventura	0.14	0.14	0.14	250282	250282	250282
COL	Colombia	14370	Buenaventura (is.)	0.14	0.14	0.14	2304	2304	2304
COL	Colombia	14371	Buga	0.14	0.14	0.14	92042	92042	92042
COL	Colombia	14372	Bugalagrande	0.14	0.14	0.14	14221	14221	14221
COL	Colombia	14373	Caicedonia	0.14	0.14	0.14	10624	10624	10624
COL	Colombia	14374	Cali	0.14	0.14	0.14	1181123	1181123	1181123
COL	Colombia	14375	Calima (darien)	0.14	0.14	0.14	10595	10595	10595
COL	Colombia	14376	Candelaria	0.14	0.14	0.14	179407	179407	179407
COL	Colombia	14377	Cartago	0.14	0.14	0.14	62212	62212	62212
COL	Colombia	14378	Dagua	0.14	0.14	0.14	35182	35182	35182
COL	Colombia	14379	El Aguila	0.14	0.14	0.14	4543	4543	4543
COL	Colombia	14380	El Cairo	0.14	0.14	0.14	1014	1014	1014
COL	Colombia	14381	El Cerrito	0.14	0.14	0.14	48479	48479	48479
COL	Colombia	14382	El Dovio	0.14	0.14	0.14	3164	3164	3164
COL	Colombia	14383	Florida	0.14	0.14	0.14	48381	48381	48381
COL	Colombia	14384	Ginebra	0.14	0.14	0.14	12732	12732	12732
COL	Colombia	14385	Guacari	0.14	0.14	0.14	20858	20858	20858
COL	Colombia	14386	Jamundi	0.14	0.14	0.14	125066	125066	125066
COL	Colombia	14387	La Cumbre	0.14	0.14	0.14	5798	5798	5798
COL	Colombia	14388	La Union	0.14	0.14	0.14	16551	16551	16551
COL	Colombia	14389	La Victoria	0.14	0.14	0.14	16808	16808	16808
COL	Colombia	14391	Obando	0.14	0.14	0.14	8751	8751	8751
COL	Colombia	14392	Palmira	0.14	0.14	0.14	259249	259249	259249
COL	Colombia	14393	Pradera	0.14	0.14	0.14	44604	44604	44604
COL	Colombia	14394	Restrepo	0.14	0.14	0.14	7797	7797	7797
COL	Colombia	14395	Riofrio	0.14	0.14	0.14	5950	5950	5950
COL	Colombia	14396	Roldanillo	0.14	0.14	0.14	14636	14636	14636
COL	Colombia	14397	San Pedro	0.14	0.14	0.14	19125	19125	19125

COL	Colombia	14398	Sevilla	0.14	0.14	0.14	52744	52744	52744
COL	Colombia	14399	Toro	0.14	0.14	0.14	8791	8791	8791
COL	Colombia	14400	Trujillo	0.14	0.14	0.14	6581	6581	6581
COL	Colombia	14401	Tulua	0.14	0.14	0.14	74149	74149	74149
COL	Colombia	14402	Ulloa	0.14	0.14	0.14	17016	17016	17016
COL	Colombia	14403	Versalles	0.14	0.14	0.14	4306	4306	4306
COL	Colombia	14404	Vijes	0.14	0.14	0.14	16729	16729	16729
COL	Colombia	14405	Yotoco	0.14	0.14	0.14	7889	7889	7889
COL	Colombia	14406	Yumbo	0.14	0.14	0.14	84093	84093	84093
COL	Colombia	14407	Zarzal	0.14	0.14	0.14	25943	25943	25943
COL	Colombia	14408	Caruru	0.14	0.14	0.14	4401	4401	4401
COL	Colombia	14409	Mitu	0.14	0.14	0.14	25486	25486	25486
COL	Colombia	14410	Pacoa	0.14	0.14	0.14	6192	6192	6192
COL	Colombia	14411	Papunahua	0.14	0.14	0.14	4462	4462	4462
COL	Colombia	14412	Taraira	0.14	0.14	0.14	1196	1196	1196
COL	Colombia	14413	Yavarate	0.14	0.14	0.14	1822	1822	1822
COL	Colombia	14414	Cumaribo	0.14	0.14	0.14	7983	7983	7983
COL	Colombia	14415	La Primavera	0.14	0.14	0.14	10983	10983	10983
COL	Colombia	14416	Puerto Carreno	0.14	0.14	0.14	9044	9044	9044
COL	Colombia	14417	San Jose De Ocune	0.14	0.14	0.14	10291	10291	10291
COL	Colombia	14418	Santa Rita	0.14	0.14	0.14	7834	7834	7834
COL	Colombia	14419	Santa Rosalia	0.14	0.14	0.14	2725	2725	2725
ECU	Ecuador	15320	Chordeleg	0.26	0.26	0.26	7107	7107	7107
ECU	Ecuador	15321	Cuenca	0.26	0.26	0.26	301368	301368	301368
ECU	Ecuador	15322	El Pan	0.26	0.26	0.26	2956	2956	2956
ECU	Ecuador	15323	Giron	0.26	0.26	0.26	7858	7858	7858
ECU	Ecuador	15324	Gualaceo	0.26	0.26	0.26	27508	27508	27508
ECU	Ecuador	15325	Nabon	0.26	0.26	0.26	8636	8636	8636
ECU	Ecuador	15326	Ona	0.26	0.26	0.26	2048	2048	2048
ECU	Ecuador	15327	Paute	0.26	0.26	0.26	21638	21638	21638
ECU	Ecuador	15328	Pucara	0.26	0.26	0.26	15960	15960	15960
ECU	Ecuador	15329	San Fernando	0.26	0.26	0.26	2424	2424	2424
ECU	Ecuador	15330	Santa Isabel	0.26	0.26	0.26	11876	11876	11876

ECU	Ecuador	15331	Sevilla De Oro	0.26	0.26	0.26	4207	4207	4207
ECU	Ecuador	15332	Sissig	0.26	0.26	0.26	14636	14636	14636
ECU	Ecuador	15333	Caluma	0.26	0.26	0.26	7408	7408	7408
ECU	Ecuador	15334	Chillanes	0.26	0.26	0.26	9677	9677	9677
ECU	Ecuador	15335	Chimbo	0.26	0.26	0.26	9184	9184	9184
ECU	Ecuador	15336	Echeandia	0.26	0.26	0.26	6997	6997	6997
ECU	Ecuador	15337	Guaranda	0.26	0.26	0.26	54751	54751	54751
ECU	Ecuador	15338	Las Naves	0.26	0.26	0.26	3452	3452	3452
ECU	Ecuador	15339	San Miguel	0.26	0.26	0.26	15191	15191	15191
ECU	Ecuador	15340	Azogues	0.26	0.26	0.26	38457	38457	38457
ECU	Ecuador	15341	Biblian	0.26	0.26	0.26	11922	11922	11922
ECU	Ecuador	15342	Canar	0.26	0.26	0.26	40749	40749	40749
ECU	Ecuador	15343	Deleg	0.26	0.26	0.26	3692	3692	3692
ECU	Ecuador	15344	El Tambo	0.26	0.26	0.26	4103	4103	4103
ECU	Ecuador	15345	La Troncal	0.26	0.26	0.26	30669	30669	30669
ECU	Ecuador	15346	Bolivar	0.26	0.26	0.26	8327	8327	8327
ECU	Ecuador	15347	Espejo	0.26	0.26	0.26	7662	7662	7662
ECU	Ecuador	15348	Mira	0.26	0.26	0.26	6817	6817	6817
ECU	Ecuador	15349	Montufar	0.26	0.26	0.26	18144	18144	18144
ECU	Ecuador	15350	Tulcan	0.26	0.26	0.26	52631	52631	52631
ECU	Ecuador	15351	Alausi	0.26	0.26	0.26	25719	25719	25719
ECU	Ecuador	15352	Chambo	0.26	0.26	0.26	7108	7108	7108
ECU	Ecuador	15353	Chunchi	0.26	0.26	0.26	8163	8163	8163
ECU	Ecuador	15354	Colta	0.26	0.26	0.26	26803	26803	26803
ECU	Ecuador	15355	Cumanda	0.26	0.26	0.26	6950	6950	6950
ECU	Ecuador	15356	Guamote	0.26	0.26	0.26	25512	25512	25512
ECU	Ecuador	15357	Guano	0.26	0.26	0.26	34455	34455	34455
ECU	Ecuador	15358	Pallatanga	0.26	0.26	0.26	6447	6447	6447
ECU	Ecuador	15359	Penipe	0.26	0.26	0.26	3852	3852	3852
ECU	Ecuador	15360	Riobamba	0.26	0.26	0.26	127162	127162	127162
ECU	Ecuador	15361	La Mana	0.26	0.26	0.26	20217	20217	20217
ECU	Ecuador	15362	Latacunga	0.26	0.26	0.26	94018	94018	94018
ECU	Ecuador	15363	Pangua	0.26	0.26	0.26	13421	13421	13421

ECU	Ecuador	15364	Pujili	0.26	0.26	0.26	48788	48788	48788
ECU	Ecuador	15365	Salcedo	0.26	0.26	0.26	37123	37123	37123
ECU	Ecuador	15366	Saquisili	0.26	0.26	0.26	16520	16520	16520
ECU	Ecuador	15367	Sigchos	0.26	0.26	0.26	13078	13078	13078
ECU	Ecuador	15368	Arenillas	0.26	0.26	0.26	22311	22311	22311
ECU	Ecuador	15369	Atahualpa	0.26	0.26	0.26	4990	4990	4990
ECU	Ecuador	15370	Balsas	0.26	0.26	0.26	3131	3131	3131
ECU	Ecuador	15371	Chilla	0.26	0.26	0.26	3443	3443	3443
ECU	Ecuador	15372	El Guabo	0.26	0.26	0.26	28178	28178	28178
ECU	Ecuador	15373	Huaquillas	0.26	0.26	0.26	23524	23524	23524
ECU	Ecuador	15374	Isla Correa	0.26	0.26	0.26	12	12	12
ECU	Ecuador	15375	Las Lajas	0.26	0.26	0.26	3246	3246	3246
ECU	Ecuador	15376	Machala	0.26	0.26	0.26	137073	137073	137073
ECU	Ecuador	15377	Marcabeli	0.26	0.26	0.26	3291	3291	3291
ECU	Ecuador	15379	Pasaje	0.26	0.26	0.26	46398	46398	46398
ECU	Ecuador	15380	Pinas	0.26	0.26	0.26	15849	15849	15849
ECU	Ecuador	15381	Portovelo	0.26	0.26	0.26	5953	5953	5953
ECU	Ecuador	15382	Santa Rosa	0.26	0.26	0.26	41159	41159	41159
ECU	Ecuador	15383	Zaruma	0.26	0.26	0.26	14312	14312	14312
ECU	Ecuador	15384	Atacames	0.26	0.26	0.26	23473	23473	23473
ECU	Ecuador	15385	Eloy Alfaro	0.26	0.26	0.26	20627	20627	20627
ECU	Ecuador	15386	Isla Luis Vargas	0.26	0.26	0.26	128480	128480	128480
ECU	Ecuador	15387	La Isla (munisme	0.26	0.26	0.26	64	64	64
ECU	Ecuador	15388	Muisne	0.26	0.26	0.26	17188	17188	17188
ECU	Ecuador	15389	Quininde	0.26	0.26	0.26	63717	63717	63717
ECU	Ecuador	15390	San Lorenzo	0.26	0.26	0.26	28966	28966	28966
ECU	Ecuador	15391	San Lorenzo (is)	0.26	0.26	0.26	1286	1286	1286
ECU	Ecuador	15414	Alfredo Baqueriz	0.26	0.26	0.26	22700	22700	22700
ECU	Ecuador	15415	Balao	0.26	0.26	0.26	13145	13145	13145
ECU	Ecuador	15416	Balzar	0.26	0.26	0.26	33348	33348	33348
ECU	Ecuador	15417	Colimes	0.26	0.26	0.26	14925	14925	14925
ECU	Ecuador	15418	Coronel Marcelin	0.26	0.26	0.26	10092	10092	10092
ECU	Ecuador	15419	Daule	0.26	0.26	0.26	74168	74168	74168

ECU	Ecuador	15420	Duran	0.26	0.26	0.26	128791	128791	128791
ECU	Ecuador	15421	El Empalme	0.26	0.26	0.26	58684	58684	58684
ECU	Ecuador	15422	El Triunfo	0.26	0.26	0.26	26823	26823	26823
ECU	Ecuador	15423	Guayaquil	0.26	0.26	0.26	1382043	1382043	1382043
ECU	Ecuador	15424	La Libertad	0.26	0.26	0.26	44483	44483	44483
ECU	Ecuador	15425	Lomas De Sargent	0.26	0.26	0.26	16589	16589	16589
ECU	Ecuador	15426	Milagro	0.26	0.26	0.26	101466	101466	101466
ECU	Ecuador	15427	Naranjal	0.26	0.26	0.26	60645	60645	60645
ECU	Ecuador	15428	Naranjito	0.26	0.26	0.26	21656	21656	21656
ECU	Ecuador	15429	Nobol	0.26	0.26	0.26	12249	12249	12249
ECU	Ecuador	15430	Palestina	0.26	0.26	0.26	9441	9441	9441
ECU	Ecuador	15431	Pedro Carbo	0.26	0.26	0.26	24927	24927	24927
ECU	Ecuador	15432	Playas	0.26	0.26	0.26	31704	31704	31704
ECU	Ecuador	15433	Salinas	0.26	0.26	0.26	56367	56367	56367
ECU	Ecuador	15434	Samborondon	0.26	0.26	0.26	39954	39954	39954
ECU	Ecuador	15435	Santa Elena	0.26	0.26	0.26	99615	99615	99615
ECU	Ecuador	15436	Santa Lucia	0.26	0.26	0.26	19407	19407	19407
ECU	Ecuador	15437	Simon Bolivar	0.26	0.26	0.26	15429	15429	15429
ECU	Ecuador	15438	Urbina Jado	0.26	0.26	0.26	31773	31773	31773
ECU	Ecuador	15439	Yaguachi	0.26	0.26	0.26	47866	47866	47866
ECU	Ecuador	15440	Antonio Ante	0.26	0.26	0.26	26854	26854	26854
ECU	Ecuador	15441	Cotacachi	0.26	0.26	0.26	24433	24433	24433
ECU	Ecuador	15442	Ibarra	0.26	0.26	0.26	104112	104112	104112
ECU	Ecuador	15443	Otavalo	0.26	0.26	0.26	61234	61234	61234
ECU	Ecuador	15444	Pimampiro	0.26	0.26	0.26	6653	6653	6653
ECU	Ecuador	15445	San Miguel Urcuq	0.26	0.26	0.26	11779	11779	11779
ECU	Ecuador	15446	Calvas	0.26	0.26	0.26	16690	16690	16690
ECU	Ecuador	15447	Catamayo	0.26	0.26	0.26	21227	21227	21227
ECU	Ecuador	15448	Celica	0.26	0.26	0.26	8568	8568	8568
ECU	Ecuador	15449	Chaguarpamba	0.26	0.26	0.26	4750	4750	4750
ECU	Ecuador	15450	Espindola	0.26	0.26	0.26	8234	8234	8234
ECU	Ecuador	15451	Gonzanama	0.26	0.26	0.26	7899	7899	7899
ECU	Ecuador	15452	Loja	0.26	0.26	0.26	122352	122352	122352

ECU	Ecuador	15453	Macara	0.26	0.26	0.26	10887	10887	10887
ECU	Ecuador	15454	Paltas	0.26	0.26	0.26	17295	17295	17295
ECU	Ecuador	15455	Pindal	0.26	0.26	0.26	4889	4889	4889
ECU	Ecuador	15456	Puyango	0.26	0.26	0.26	8735	8735	8735
ECU	Ecuador	15457	Quilanga	0.26	0.26	0.26	2565	2565	2565
ECU	Ecuador	15458	Saraguro	0.26	0.26	0.26	15398	15398	15398
ECU	Ecuador	15459	Sozoranga	0.26	0.26	0.26	4358	4358	4358
ECU	Ecuador	15460	Zapotillo	0.26	0.26	0.26	7260	7260	7260
ECU	Ecuador	15461	Baba	0.26	0.26	0.26	24154	24154	24154
ECU	Ecuador	15462	Babahoyo	0.26	0.26	0.26	83985	83985	83985
ECU	Ecuador	15463	Buena Fe	0.26	0.26	0.26	35918	35918	35918
ECU	Ecuador	15464	Montalvo	0.26	0.26	0.26	14651	14651	14651
ECU	Ecuador	15465	Palenque	0.26	0.26	0.26	13213	13213	13213
ECU	Ecuador	15466	Puebloviejo	0.26	0.26	0.26	22614	22614	22614
ECU	Ecuador	15467	Quevedo	0.26	0.26	0.26	141323	141323	141323
ECU	Ecuador	15468	Urdaneta	0.26	0.26	0.26	20690	20690	20690
ECU	Ecuador	15469	Ventanas	0.26	0.26	0.26	51228	51228	51228
ECU	Ecuador	15470	Vinces	0.26	0.26	0.26	41621	41621	41621
ECU	Ecuador	15471	24 De Mayo	0.26	0.26	0.26	17144	17144	17144
ECU	Ecuador	15472	Bolivar	0.26	0.26	0.26	23615	23615	23615
ECU	Ecuador	15473	Chone	0.26	0.26	0.26	74698	74698	74698
ECU	Ecuador	15474	El Carmen	0.26	0.26	0.26	53064	53064	53064
ECU	Ecuador	15475	Flavio Alfaro	0.26	0.26	0.26	15043	15043	15043
ECU	Ecuador	15476	Isla Del Medio	0.26	0.26	0.26	33175	33175	33175
ECU	Ecuador	15477	Jipijapa	0.26	0.26	0.26	55247	55247	55247
ECU	Ecuador	15478	Junin	0.26	0.26	0.26	11721	11721	11721
ECU	Ecuador	15479	Manta	0.26	0.26	0.26	131466	131466	131466
ECU	Ecuador	15480	Montecristi	0.26	0.26	0.26	58048	58048	58048
ECU	Ecuador	15481	Pajan	0.26	0.26	0.26	22541	22541	22541
ECU	Ecuador	15482	Pichincha	0.26	0.26	0.26	19191	19191	19191
ECU	Ecuador	15483	Portoviejo	0.26	0.26	0.26	153811	153811	153811
ECU	Ecuador	15484	Rocafuerte	0.26	0.26	0.26	24985	24985	24985
ECU	Ecuador	15485	Santa Ana	0.26	0.26	0.26	38447	38447	38447

ECU	Ecuador	15486	Sucre	0.26	0.26	0.26	60250	60250	60250
ECU	Ecuador	15487	Tosagua	0.26	0.26	0.26	21848	21848	21848
ECU	Ecuador	15488	Gualaquiza	0.26	0.26	0.26	10572	10572	10572
ECU	Ecuador	15489	Huamboya	0.26	0.26	0.26	6734	6734	6734
ECU	Ecuador	15490	Limon Indanza	0.26	0.26	0.26	7900	7900	7900
ECU	Ecuador	15491	Morona	0.26	0.26	0.26	42791	42791	42791
ECU	Ecuador	15492	Palora	0.26	0.26	0.26	4369	4369	4369
ECU	Ecuador	15493	San Juan Bosco	0.26	0.26	0.26	3360	3360	3360
ECU	Ecuador	15494	Santiago	0.26	0.26	0.26	8842	8842	8842
ECU	Ecuador	15495	Sucua	0.26	0.26	0.26	11294	11294	11294
ECU	Ecuador	15496	Zamora	0.26	0.26	0.26	19490	19490	19490
ECU	Ecuador	15497	Archidona	0.55	0.55	0.55	7850	7850	7850
ECU	Ecuador	15498	El Chaco	0.55	0.55	0.55	2940	2940	2940
ECU	Ecuador	15499	Quijos	0.55	0.55	0.55	2034	2034	2034
ECU	Ecuador	15500	Tena	0.55	0.55	0.55	22059	22059	22059
ECU	Ecuador	15501	Aguarico	0.26	0.26	0.26	3536	3536	3536
ECU	Ecuador	15502	La Joya De Los S	0.26	0.26	0.26	32780	32780	32780
ECU	Ecuador	15503	Loreto	0.26	0.26	0.26	14476	14476	14476
ECU	Ecuador	15504	Orellana	0.26	0.26	0.26	40695	40695	40695
ECU	Ecuador	15505	Mera	0.26	0.26	0.26	8750	8750	8750
ECU	Ecuador	15506	Pastaza	0.26	0.26	0.26	46114	46114	46114
ECU	Ecuador	15507	Santa Clara	0.26	0.26	0.26	2386	2386	2386
ECU	Ecuador	15508	Cayambe	0.26	0.26	0.26	52323	52323	52323
ECU	Ecuador	15509	Mejia	0.26	0.26	0.26	64635	64635	64635
ECU	Ecuador	15510	Pedro Moncayo	0.26	0.26	0.26	22396	22396	22396
ECU	Ecuador	15511	Pedro Vicente M.	0.26	0.26	0.26	8219	8219	8219
ECU	Ecuador	15512	Quito	0.26	0.26	0.26	1364683	1364683	1364683
ECU	Ecuador	15513	Ruminahui	0.26	0.26	0.26	54310	54310	54310
ECU	Ecuador	15514	Santo Domingo	0.26	0.26	0.26	225762	225762	225762
ECU	Ecuador	15515	Sn Miguel Bancos	0.26	0.26	0.26	32932	32932	32932
ECU	Ecuador	15516	Cascales	0.26	0.26	0.26	7160	7160	7160
ECU	Ecuador	15517	Gonzalo Pizarro	0.26	0.26	0.26	5752	5752	5752
ECU	Ecuador	15518	Lago Agrio	0.26	0.26	0.26	69590	69590	69590

ECU	Ecuador	15519	Putumayo	0.26	0.26	0.26	7634	7634	7634
ECU	Ecuador	15520	Shushufindi	0.26	0.26	0.26	25108	25108	25108
ECU	Ecuador	15521	Sucumbios	0.26	0.26	0.26	3489	3489	3489
ECU	Ecuador	15522	Ambato	0.26	0.26	0.26	194507	194507	194507
ECU	Ecuador	15523	Banos	0.26	0.26	0.26	10110	10110	10110
ECU	Ecuador	15524	Cevallos	0.26	0.26	0.26	4472	4472	4472
ECU	Ecuador	15525	Mocha	0.26	0.26	0.26	3901	3901	3901
ECU	Ecuador	15526	Patate	0.26	0.26	0.26	8094	8094	8094
ECU	Ecuador	15527	Quero	0.26	0.26	0.26	10769	10769	10769
ECU	Ecuador	15528	Santiago Pillaro	0.26	0.26	0.26	24592	24592	24592
ECU	Ecuador	15529	Sn Pedro Pelileo	0.26	0.26	0.26	35156	35156	35156
ECU	Ecuador	15530	Tisaleo	0.26	0.26	0.26	7656	7656	7656
ECU	Ecuador	15531	Chinchipe	0.26	0.26	0.26	11844	11844	11844
ECU	Ecuador	15532	El Pangui	0.26	0.26	0.26	5527	5527	5527
ECU	Ecuador	15533	Nangaritza	0.26	0.26	0.26	4394	4394	4394
ECU	Ecuador	15534	Yacuambi	0.26	0.26	0.26	3901	3901	3901
ECU	Ecuador	15535	Yantzaza	0.26	0.26	0.26	11381	11381	11381
ECU	Ecuador	15536	El Piedrero	0.26	0.26	0.26	4497	4497	4497
ECU	Ecuador	15537	La Concordia	0.26	0.26	0.26	34587	34587	34587
ECU	Ecuador	15538	Las Golondrinas	0.26	0.26	0.26	2232	2232	2232
ECU	Ecuador	15539	Manga De Cura	0.26	0.26	0.26	14417	14417	14417
ERI	Eritrea	15835	Adi Tekeliezan	0.00	0.00	0.00	77428	77428	77428
ERI	Eritrea	15836	Asmat	0.00	0.00	0.00	165835	165835	165835
ERI	Eritrea	15837	Elabered	0.00	0.00	0.00	62225	62225	62225
ERI	Eritrea	15838	Geleb	0.00	0.00	0.00	38202	38202	38202
ERI	Eritrea	15839	Habero	0.00	0.00	0.00	61537	61537	61537
ERI	Eritrea	15840	Hagaz	0.00	0.00	0.00	43885	43885	43885
ERI	Eritrea	15841	Halhal	0.00	0.00	0.00	71752	71752	71752
ERI	Eritrea	15842	Hamelmalo	0.00	0.00	0.00	45453	45453	45453
ERI	Eritrea	15843	Keren	0.00	0.00	0.00	10520	10520	10520
ERI	Eritrea	15844	Kerkebet	0.00	0.00	0.00	52966	52966	52966
ERI	Eritrea	15845	Sel'a	0.00	0.00	0.00	99396	99396	99396
ERI	Eritrea	15847	Adi Keih	0.00	0.00	0.00	70820	70820	70820

ERI	Eritrea	15848	Adi Kuala	0.00	0.00	0.00	68011	68011	68011
ERI	Eritrea	15849	Areza	0.00	0.00	0.00	125926	125926	125926
ERI	Eritrea	15850	Dbarwa	0.00	0.00	0.00	384637	384637	384637
ERI	Eritrea	15851	Dekemhare	0.00	0.00	0.00	233579	233579	233579
ERI	Eritrea	15852	Emni Haili	0.00	0.00	0.00	25622	25622	25622
ERI	Eritrea	15853	Mai Aini	0.00	0.00	0.00	54388	54388	54388
ERI	Eritrea	15854	Mai Mne	0.00	0.00	0.00	90114	90114	90114
ERI	Eritrea	15855	Mendefera	0.00	0.00	0.00	109804	109804	109804
ERI	Eritrea	15856	Segeneiti	0.00	0.00	0.00	75902	75902	75902
ERI	Eritrea	15857	Senafe	0.00	0.00	0.00	65836	65836	65836
ERI	Eritrea	15858	Tsorona	0.00	0.00	0.00	43194	43194	43194
ERI	Eritrea	15863	Akurdet	0.00	0.00	0.00	7118	7118	7118
ERI	Eritrea	15864	Barentu	0.00	0.00	0.00	18569	18569	18569
ERI	Eritrea	15865	Dge	0.00	0.00	0.00	46432	46432	46432
ERI	Eritrea	15866	Forto	0.00	0.00	0.00	45235	45235	45235
ERI	Eritrea	15867	Gonei	0.00	0.00	0.00	87934	87934	87934
ERI	Eritrea	15868	Haikota	0.00	0.00	0.00	36153	36153	36153
ERI	Eritrea	15869	La'elay Gash	0.00	0.00	0.00	107840	107840	107840
ERI	Eritrea	15870	Logo Anseba	0.00	0.00	0.00	209186	209186	209186
ERI	Eritrea	15871	Mensura	0.00	0.00	0.00	185072	185072	185072
ERI	Eritrea	15872	Mogolo	0.00	0.00	0.00	51881	51881	51881
ERI	Eritrea	15873	Molqi	0.00	0.00	0.00	282668	282668	282668
ERI	Eritrea	15874	Omhajer	0.00	0.00	0.00	93601	93601	93601
ERI	Eritrea	15875	Shambqo	0.00	0.00	0.00	80252	80252	80252
ERI	Eritrea	15876	Tesseney	0.00	0.00	0.00	22258	22258	22258
ERI	Eritrea	15877	Berik	0.00	0.00	0.00	130118	130118	130118
ERI	Eritrea	15878	Gala Nefhi	0.00	0.00	0.00	185727	185727	185727
ERI	Eritrea	15879	Northern Asmara	0.00	0.00	0.00	7013	7013	7013
ERI	Eritrea	15880	Northern Merab	0.00	0.00	0.00	18565	18565	18565
ERI	Eritrea	15881	Serejeqa	0.00	0.00	0.00	129419	129419	129419
ERI	Eritrea	15882	Southern Asmara	0.00	0.00	0.00	17645	17645	17645
ERI	Eritrea	15883	Southern Merab	0.00	0.00	0.00	28656	28656	28656
ERI	Eritrea	15884	Adobhe	0.00	0.00	0.00	81512	81512	81512

ERI	Eritrea	15885	Afabet	0.00	0.00	0.00	288763	288763	288763
ERI	Eritrea	15886	Foro	0.00	0.00	0.00	220950	220950	220950
ERI	Eritrea	15887	Gel'alo	0.00	0.00	0.00	158377	158377	158377
ERI	Eritrea	15888	Gindae	0.00	0.00	0.00	237372	237372	237372
ERI	Eritrea	15889	Massawa	0.00	0.00	0.00	17068	17068	17068
ERI	Eritrea	15890	Nakfa	0.00	0.00	0.00	89671	89671	89671
ERI	Eritrea	15891	Quarura	0.00	0.00	0.00	217159	217159	217159
ERI	Eritrea	15892	Sheb	0.00	0.00	0.00	118466	118466	118466
ETH	Ethiopia	40782	Addis Ababa Zone1	0.00	0.00	0.00	451004	451004	451004
ETH	Ethiopia	40783	Addis Ababa Zone2	0.00	0.00	0.00	713512	713512	713512
ETH	Ethiopia	40784	Addis Ababa Zone3	0.00	0.00	0.00	639391	639391	639391
ETH	Ethiopia	40785	Addis Ababa Zone4	0.00	0.00	0.00	834745	834745	834745
ETH	Ethiopia	40786	Addis Ababa Zone5	0.00	0.00	0.00	781055	781055	781055
ETH	Ethiopia	40788	Afar Zone 1	0.00	0.00	0.00	468686	468686	468686
ETH	Ethiopia	40789	Afar Zone 2	0.00	0.00	0.00	305418	305418	305418
ETH	Ethiopia	40790	Afar Zone 4	0.00	0.00	0.00	177844	177844	177844
ETH	Ethiopia	40791	Afar Zone 5	0.00	0.00	0.00	388093	388093	388093
ETH	Ethiopia	40792	Afar Zone 3	0.00	0.00	0.00	222726	222726	222726
ETH	Ethiopia	40793	Admin unit not available	0.00	0.00	0.00	32854	32854	32854
ETH	Ethiopia	40794	Awi	0.00	0.00	0.00	1068991	1068991	1068991
ETH	Ethiopia	40796	East Gojam	0.00	0.00	0.00	2569428	2569428	2569428
ETH	Ethiopia	40797	North Gonder	0.00	0.00	0.00	3133196	3133196	3133196
ETH	Ethiopia	40799	North Wollo	0.00	0.00	0.00	1875584	1875584	1875584
ETH	Ethiopia	40800	Oromia Zone	0.00	0.00	0.00	699372	699372	699372
ETH	Ethiopia	40801	South Gonder	0.00	0.00	0.00	2630872	2630872	2630872
ETH	Ethiopia	40802	South Wollo	0.00	0.00	0.00	3187493	3187493	3187493
ETH	Ethiopia	40803	Wag Hemira	0.00	0.00	0.00	409841	409841	409841
ETH	Ethiopia	40804	West Gojam	0.00	0.00	0.00	2629300	2629300	2629300
ETH	Ethiopia	40805	Asosa	0.00	0.00	0.00	347878	347878	347878
ETH	Ethiopia	40807	Metekel	0.00	0.00	0.00	297352	297352	297352
ETH	Ethiopia	40809	Dire Dawa	0.00	0.00	0.00	448875	448875	448875
ETH	Ethiopia	40814	Harari	0.00	0.00	0.00	219402	219402	219402
ETH	Ethiopia	40820	East Wellega	0.00	0.00	0.00	1885361	1885361	1885361

ETH	Ethiopia	40821	Illubabor	0.00	0.00	0.00	1278368	1278368	1278368
ETH	Ethiopia	40822	Jimma	0.00	0.00	0.00	2945777	2945777	2945777
ETH	Ethiopia	40824	West Harerghe	0.00	0.00	0.00	1968588	1968588	1968588
ETH	Ethiopia	40826	West Wellega	0.00	0.00	0.00	2294059	2294059	2294059
ETH	Ethiopia	40827	Amaro Special Woreda	0.00	0.00	0.00	147352	147352	147352
ETH	Ethiopia	40829	Burji Special Woreda	0.00	0.00	0.00	59364	59364	59364
ETH	Ethiopia	40830	Derashe Special Woreda	0.00	0.00	0.00	139245	139245	139245
ETH	Ethiopia	40831	Gedeo	0.00	0.00	0.00	858293	858293	858293
ETH	Ethiopia	40833	Hadiya	0.00	0.00	0.00	1604035	1604035	1604035
ETH	Ethiopia	40836	Konso Special Woreda	0.00	0.00	0.00	235523	235523	235523
ETH	Ethiopia	40838	Sidama	0.00	0.00	0.00	3186409	3186409	3186409
ETH	Ethiopia	40839	South Omo	0.37	0.47	0.54	267026	205069	163224
ETH	Ethiopia	40840	Yem Special Woreda	0.00	0.00	0.00	99052	99052	99052
ETH	Ethiopia	40841	Afder	0.00	0.00	0.00	479247	479247	479247
ETH	Ethiopia	40842	Deghabur	0.00	0.00	0.00	417325	417325	417325
ETH	Ethiopia	40844	Gode	0.00	0.00	0.00	444934	444934	444934
ETH	Ethiopia	40845	Jijiga	0.00	0.00	0.00	930441	930441	930441
ETH	Ethiopia	40848	Shinile	0.00	0.00	0.00	434475	434475	434475
ETH	Ethiopia	40849	Warder	0.00	0.00	0.00	458265	458265	458265
ETH	Ethiopia	40850	Central Tigray	0.00	0.00	0.00	1402618	1402618	1402618
ETH	Ethiopia	40851	Easetern Tigray	0.00	0.00	0.00	866047	866047	866047
ETH	Ethiopia	40852	Mekele	0.00	0.00	0.00	174931	174931	174931
ETH	Ethiopia	40853	Southern Tigray	0.00	0.00	0.00	1184690	1184690	1184690
ETH	Ethiopia	47680	Addis Ababa Zone6	0.00	0.00	0.00	137937	137937	137937
ETH	Ethiopia	47681	Bahir Dar Special Zone	0.00	0.00	0.00	138607	138607	138607
ETH	Ethiopia	47682	North Shewa (K3)	0.00	0.00	0.00	2328416	2328416	2328416
ETH	Ethiopia	47683	Kemashi	0.00	0.00	0.00	110350	110350	110350
ETH	Ethiopia	47684	Zone 1	0.00	0.00	0.00	171886	171886	171886
ETH	Ethiopia	47685	Zone 2	0.00	0.00	0.00	100239	100239	100239
ETH	Ethiopia	47686	Arsi	0.00	0.00	0.00	3391333	3391333	3391333
ETH	Ethiopia	47687	Bale	0.00	0.00	0.00	1829828	1829828	1829828
ETH	Ethiopia	47688	Borena	0.00	0.00	0.00	843584	843584	843584
ETH	Ethiopia	47689	East Harerghe	0.00	0.00	0.00	2871814	2871814	2871814

ETH	Ethiopia	47690	East Shewa	0.00	0.00	0.00	2618483	2618483	2618483
ETH	Ethiopia	47691	Guji	0.00	0.00	0.00	1307193	1307193	1307193
ETH	Ethiopia	47692	North Shewa (K4)	0.00	0.00	0.00	1748276	1748276	1748276
ETH	Ethiopia	47693	South West Shewa	0.00	0.00	0.00	1234001	1234001	1234001
ETH	Ethiopia	47694	West Shewa	0.00	0.00	0.00	2293278	2293278	2293278
ETH	Ethiopia	47695	Alaba Special Wereda	0.00	0.00	0.00	290514	290514	290514
ETH	Ethiopia	47696	Basketo Special Woreda	0.00	0.00	0.00	50870	50870	50870
ETH	Ethiopia	47697	Benchi Maji	0.00	0.00	0.00	496988	496988	496988
ETH	Ethiopia	47698	Dawuro	0.00	0.00	0.00	413564	413564	413564
ETH	Ethiopia	47699	Gamo Gofa	0.00	0.00	0.00	1652442	1652442	1652442
ETH	Ethiopia	47700	Guraghe	0.00	0.00	0.00	1770430	1770430	1770430
ETH	Ethiopia	47701	Kaffa	0.00	0.00	0.00	901310	901310	901310
ETH	Ethiopia	47702	Kembata Alaba Tembaro	0.00	0.00	0.00	813783	813783	813783
ETH	Ethiopia	47703	Konta Special Woreda	0.00	0.00	0.00	80686	80686	80686
ETH	Ethiopia	47704	Shaka	0.00	0.00	0.00	202220	202220	202220
ETH	Ethiopia	47705	Siltie	0.00	0.00	0.00	596581	596581	596581
ETH	Ethiopia	47706	Wolayita	0.00	0.00	0.00	1763121	1763121	1763121
ETH	Ethiopia	47707	Fiq	0.00	0.00	0.00	346945	346945	346945
ETH	Ethiopia	47708	Korahe(Kebri Dehar)	0.00	0.00	0.00	350646	350646	350646
ETH	Ethiopia	47709	Liben	0.00	0.00	0.00	687598	687598	687598
ETH	Ethiopia	47710	North Western Tigray	0.00	0.00	0.00	780952	780952	780952
ETH	Ethiopia	47711	Western Tigray	0.00	0.00	0.00	293499	293499	293499
GAB	Gabon	16357	Komo	0.23	0.25	0.26	6925	6632	6481
GAB	Gabon	16358	Komo-Mondah	0.23	0.25	0.26	61010	58424	57095
GAB	Gabon	16359	Libreville	0.23	0.25	0.26	379373	363292	355028
GAB	Gabon	16360	Noya	0.23	0.25	0.26	3369	3226	3152
GAB	Gabon	16361	Bayi-Brikolo	0.23	0.25	0.26	2515	2409	2354
GAB	Gabon	16362	Djoue	0.23	0.25	0.26	1881	1801	1760
GAB	Gabon	16363	Djouori-Agnili	0.23	0.25	0.26	2372	2272	2220
GAB	Gabon	16364	Lebombi-Leyou	0.23	0.25	0.26	40110	38409	37536
GAB	Gabon	16365	Lekabi-Lewolo	0.23	0.25	0.26	3399	3255	3181
GAB	Gabon	16366	Lekoko	0.23	0.25	0.26	1868	1788	1748
GAB	Gabon	16367	Lekoni-Lekori	0.23	0.25	0.26	4788	4585	4481

GAB	Gabon	16368	Ogooue-Letili	0.23	0.25	0.26	2460	2356	2303
GAB	Gabon	16369	Passa	0.23	0.25	0.26	91699	87812	85815
GAB	Gabon	16370	Plateaux	0.23	0.25	0.26	4816	4612	4507
GAB	Gabon	16371	Sebe-Brikolo	0.23	0.25	0.26	6619	6339	6195
GAB	Gabon	16372	Abanga-Bigne	0.23	0.25	0.26	7273	6965	6806
GAB	Gabon	16373	Ogooue et Lacs	0.23	0.25	0.26	31690	30347	29657
GAB	Gabon	16374	Boumi-Louetsi	0.23	0.25	0.26	12257	11738	11471
GAB	Gabon	16375	Dola	0.23	0.25	0.26	4593	4398	4298
GAB	Gabon	16376	Douya-Onoye	0.23	0.25	0.26	14227	13624	13314
GAB	Gabon	16377	Louetsi-Bibaka	0.23	0.25	0.26	1979	1895	1852
GAB	Gabon	16378	Louetsi-Wano	0.23	0.25	0.26	8368	8013	7831
GAB	Gabon	16379	Mougalaba	0.23	0.25	0.26	1442	1381	1350
GAB	Gabon	16380	Ndolou	0.23	0.25	0.26	3106	2974	2906
GAB	Gabon	16381	Ogoulou	0.23	0.25	0.26	5460	5229	5110
GAB	Gabon	16382	Tsamba-Magotsi	0.23	0.25	0.26	8656	8289	8100
GAB	Gabon	16383	Basse-Banio	0.23	0.25	0.26	4999	4787	4678
GAB	Gabon	16384	Douigni	0.23	0.25	0.26	3946	3779	3693
GAB	Gabon	16385	Doutsila	0.23	0.25	0.26	2163	2071	2024
GAB	Gabon	16386	Haute-Banio	0.23	0.25	0.26	991	949	927
GAB	Gabon	16387	Mongo	0.23	0.25	0.26	2037	1950	1906
GAB	Gabon	16388	Mougoutsi	0.23	0.25	0.26	14822	14193	13870
GAB	Gabon	16389	Ivindo	0.23	0.25	0.26	18911	18110	17698
GAB	Gabon	16390	Lope	0.23	0.25	0.26	9855	9437	9222
GAB	Gabon	16391	Mvoung	0.23	0.25	0.26	3133	3000	2932
GAB	Gabon	16392	Zadie	0.23	0.25	0.26	9467	9065	8859
GAB	Gabon	16393	Lolo-Bouenguidi	0.23	0.25	0.26	19399	18577	18155
GAB	Gabon	16394	Lombo-Bouenguidi	0.23	0.25	0.26	2949	2824	2760
GAB	Gabon	16395	Mouloundou	0.23	0.25	0.26	16440	15743	15385
GAB	Gabon	16396	Offoue-Onoye	0.23	0.25	0.26	2957	2832	2768
GAB	Gabon	16397	Bendje	0.23	0.25	0.26	87839	84115	82202
GAB	Gabon	16398	Etimboue	0.23	0.25	0.26	3798	3637	3554
GAB	Gabon	16399	Ndougou	0.23	0.25	0.26	5297	5073	4957
GAB	Gabon	16400	Haut-Komo	0.23	0.25	0.26	3322	3181	3108

GAB	Gabon	16401	Haut-Ntem	0.23	0.25	0.26	9128	8741	8542
GAB	Gabon	16402	Ntem	0.23	0.25	0.26	26228	25116	24545
GAB	Gabon	16403	Okano	0.23	0.25	0.26	9936	9514	9298
GAB	Gabon	16404	Woleu	0.23	0.25	0.26	49006	46929	45861
GHA	Ghana	16564	Adansi East	0.49	0.53	0.54	44895	40112	37786
GHA	Ghana	16565	Adansi West	0.49	0.53	0.54	82707	73896	69611
GHA	Ghana	16566	Afigya Sekyere	0.49	0.53	0.54	71162	63581	59894
GHA	Ghana	16567	Ahafo Ano North	0.49	0.53	0.54	35133	31390	29570
GHA	Ghana	16568	Ahafo Ano South	0.49	0.53	0.54	36160	32308	30434
GHA	Ghana	16569	Amansie East	0.49	0.53	0.54	96160	85916	80934
GHA	Ghana	16570	Amansie West	0.49	0.53	0.54	40020	35757	33683
GHA	Ghana	16571	Asante Akim North	0.49	0.53	0.54	51028	45592	42948
GHA	Ghana	16572	Asante Akim South	0.49	0.53	0.54	39346	35155	33116
GHA	Ghana	16573	Atwima	0.49	0.53	0.54	101200	90419	85175
GHA	Ghana	16574	Bosomtwi Kwanwoma	0.49	0.53	0.54	126777	113272	106703
GHA	Ghana	16575	Ejura-Sekyedumasi	0.49	0.53	0.54	31855	28462	26811
GHA	Ghana	16576	Ejusu/Juaben	0.49	0.53	0.54	66031	58997	55575
GHA	Ghana	16577	Kumasi	0.49	0.53	0.54	661414	590953	556682
GHA	Ghana	16578	Kwabere	0.49	0.53	0.54	57600	51464	48479
GHA	Ghana	16579	Offinso	0.49	0.53	0.54	47100	42082	39642
GHA	Ghana	16580	Sekyere East	0.49	0.53	0.54	49689	44396	41821
GHA	Ghana	16581	Sekyere West	0.49	0.53	0.54	49690	44397	41822
GHA	Ghana	16582	Asunafo	0.62	0.65	0.67	41341	35782	30279
GHA	Ghana	16583	Asutifi	0.62	0.65	0.67	22628	19585	16573
GHA	Ghana	16584	Atebubu	0.62	0.65	0.67	44034	38112	32250
GHA	Ghana	16585	Berekum	0.62	0.65	0.67	27156	23504	19889
GHA	Ghana	16586	Dormaa	0.62	0.65	0.67	41719	36109	30555
GHA	Ghana	16587	Jaman	0.62	0.65	0.67	32749	28345	23986
GHA	Ghana	16588	Kintampo	0.89	0.94	0.96	0	0	0
GHA	Ghana	16589	Nkoranza	0.62	0.65	0.67	33765	29224	24729
GHA	Ghana	16590	Sene	0.94	0.95	0.96	0	0	0
GHA	Ghana	16591	Sunyani	0.62	0.65	0.67	40966	35457	30004
GHA	Ghana	16592	Tano	0.62	0.65	0.67	29605	25624	21683

GHA	Ghana	16593	Techiman	0.62	0.65	0.67	39219	33945	28724
GHA	Ghana	16594	Wenchi	0.62	0.65	0.67	37440	32405	27421
GHA	Ghana	16595	Abura/Asebu/Kwamankese	0.49	0.53	0.54	42324	37815	35622
GHA	Ghana	16596	Agona	0.49	0.53	0.54	77708	69430	65403
GHA	Ghana	16597	Ajumako Enyan	0.49	0.53	0.54	43408	38784	36535
GHA	Ghana	16598	Asikuma/Odoben/Brakwa	0.49	0.53	0.54	37624	33616	31667
GHA	Ghana	16599	Assin	0.49	0.53	0.54	92722	82844	78040
GHA	Ghana	16600	Awutu/Efutu/Senya	0.49	0.53	0.54	115803	103466	97466
GHA	Ghana	16601	Cape Coast	0.49	0.53	0.54	60788	54312	51162
GHA	Ghana	16602	Komenda/Edina Aguafo	0.49	0.53	0.54	46924	41926	39494
GHA	Ghana	16603	Mfantsiman	0.49	0.53	0.54	59441	53109	50029
GHA	Ghana	16604	Twifu Heman/Lower Denkyira	0.49	0.53	0.54	36186	32331	30456
GHA	Ghana	16605	Upper Denkyira	0.49	0.53	0.54	42133	37645	35462
GHA	Ghana	16606	Afram Plains	0.67	0.67	0.68	30792	29817	29225
GHA	Ghana	16607	Akwapim North	0.67	0.67	0.68	21356	20680	20269
GHA	Ghana	16608	Akwapim South	0.67	0.67	0.68	21836	21145	20724
GHA	Ghana	16609	Asuogyaman	0.67	0.67	0.68	14884	14413	14127
GHA	Ghana	16610	Birim North	0.67	0.67	0.68	24876	24089	23610
GHA	Ghana	16611	Birim South	0.67	0.67	0.68	37600	36410	35686
GHA	Ghana	16612	East Akim	0.67	0.67	0.68	39838	38578	37811
GHA	Ghana	16613	Fanteakwa	0.67	0.67	0.68	15401	14914	14617
GHA	Ghana	16614	Kwaebibirem	0.67	0.67	0.68	26751	25905	25390
GHA	Ghana	16615	Kwahu South	0.67	0.67	0.68	35160	34047	33370
GHA	Ghana	16616	Manya Krobo	0.67	0.67	0.68	20847	20188	19786
GHA	Ghana	16617	New Juaben	0.67	0.67	0.68	27913	27029	26492
GHA	Ghana	16618	Suhum/Kraboa/Coaltar	0.67	0.67	0.68	25030	24238	23756
GHA	Ghana	16619	West Akim	0.67	0.67	0.68	29186	28262	27700
GHA	Ghana	16620	Yilo Krobo	0.67	0.67	0.68	15860	15358	15052
GHA	Ghana	16621	Accra	0.49	0.53	0.54	802375	716898	675322
GHA	Ghana	16622	Dangbe East	0.49	0.53	0.54	44884	40102	37777
GHA	Ghana	16623	Dangbe West	0.49	0.53	0.54	37638	33629	31679
GHA	Ghana	16624	Ga	0.49	0.53	0.54	305337	272809	256988
GHA	Ghana	16625	Tema	0.49	0.53	0.54	242073	216285	203742

GHA	Ghana	16626	Bole	0.54	0.60	0.65	47426	35936	26362
GHA	Ghana	16627	East Gonja	0.54	0.60	0.65	71683	54316	39846
GHA	Ghana	16628	East Mamprusi	0.54	0.60	0.65	68306	51757	37969
GHA	Ghana	16629	Gushiegu/Karaga	0.54	0.60	0.65	53017	40172	29470
GHA	Ghana	16630	Nanumba	0.54	0.60	0.65	68391	51821	38016
GHA	Ghana	16631	Saboba/Chereponi	0.54	0.60	0.65	34731	26316	19305
GHA	Ghana	16632	Savelgu/Nanton	0.54	0.60	0.65	43158	32701	23990
GHA	Ghana	16633	Tamale	0.54	0.60	0.65	108496	82209	60308
GHA	Ghana	16634	Tolon/Kumbungu	0.54	0.60	0.65	31039	23519	17253
GHA	Ghana	16635	West Gonja	0.54	0.60	0.65	48316	36610	26857
GHA	Ghana	16636	West Mamprusi	0.54	0.60	0.65	48019	36385	26692
GHA	Ghana	16637	Yendi	0.54	0.60	0.65	58987	44695	32788
GHA	Ghana	16638	Zabzugu/Tatale	0.54	0.60	0.65	34975	26501	19441
GHA	Ghana	16639	Bawku East	0.65	0.73	0.76	55739	26477	14127
GHA	Ghana	16640	Bawku West	0.65	0.73	0.76	14113	6704	3577
GHA	Ghana	16641	Bolgatanga	0.65	0.73	0.76	38397	18239	9732
GHA	Ghana	16642	Bongo	0.65	0.73	0.76	14527	6901	3682
GHA	Ghana	16643	Builsa	0.89	0.95	0.99	0	0	0
GHA	Ghana	16644	Kasena/Nankani	0.89	0.95	0.99	0	0	0
GHA	Ghana	16645	Jirapa/Lambussie	0.62	0.64	0.67	26671	22948	19181
GHA	Ghana	16646	Lawra	0.62	0.64	0.67	18959	16313	13635
GHA	Ghana	16647	Nadowli	0.62	0.64	0.67	18906	16267	13597
GHA	Ghana	16648	Sissala	0.62	0.64	0.67	20866	17954	15007
GHA	Ghana	16649	Wa	0.62	0.64	0.67	50816	43723	36546
GHA	Ghana	16650	Akatsi	0.67	0.67	0.68	16504	15982	15664
GHA	Ghana	16651	Ho	0.67	0.67	0.68	48273	46745	45816
GHA	Ghana	16652	Hohoe	0.67	0.67	0.68	36687	35526	34819
GHA	Ghana	16653	Jasikan	0.67	0.67	0.68	17524	16969	16632
GHA	Ghana	16654	Kadjebi	0.67	0.67	0.68	9806	9495	9307
GHA	Ghana	16655	Kete-Krachi	0.67	0.67	0.68	35275	34158	33479
GHA	Ghana	16656	Ketu	0.67	0.67	0.68	47222	45728	44818
GHA	Ghana	16657	Kpandu	0.67	0.67	0.68	18359	17778	17424
GHA	Ghana	16658	Nkwanta	0.67	0.67	0.68	23834	23080	22621

GHA	Ghana	16659	North Tongu	0.67	0.67	0.68	21078	20410	20005
GHA	Ghana	16661	South Tongu	0.67	0.67	0.68	11748	11376	11150
GHA	Ghana	16662	Ahanta West	0.49	0.53	0.54	29477	26337	24810
GHA	Ghana	16663	Aowin Suaman	0.49	0.53	0.54	39222	35044	33011
GHA	Ghana	16664	Bibiani Anhwiaso-Bekwai	0.49	0.53	0.54	44307	39587	37291
GHA	Ghana	16665	Jomoro	0.49	0.53	0.54	47766	42678	40203
GHA	Ghana	16666	Juabeso-Bia	0.49	0.53	0.54	77838	69546	65513
GHA	Ghana	16667	Mpohor Wassa	0.49	0.53	0.54	49090	43860	41317
GHA	Ghana	16668	Nzema East	0.49	0.53	0.54	47643	42568	40099
GHA	Ghana	16669	Sefwi-Wiawso	0.49	0.53	0.54	75302	67280	63378
GHA	Ghana	16670	Shama Ahanta East	0.49	0.53	0.54	218240	194991	183683
GHA	Ghana	16671	Wassa Amenfi	0.49	0.53	0.54	85487	76380	71950
GHA	Ghana	16672	Wassa West	0.49	0.53	0.54	84377	75389	71017
GHA	Ghana	33108	Keta	0.67	0.67	0.68	24118	23355	22891
GHA	Ghana	33109	Gomoa	0.49	0.53	0.54	82027	73289	69038
GIN	Guinea	40708	Boffa	0.57	0.67	0.78	52824	29751	4745
GIN	Guinea	40709	Boke	0.57	0.67	0.78	126676	71346	11379
GIN	Guinea	40710	Gaoual	0.57	0.67	0.78	46528	26206	4179
GIN	Guinea	40711	Koundara	0.57	0.67	0.78	30166	16990	2710
GIN	Guinea	40712	Dixinn	0.57	0.67	0.78	86711	48837	7789
GIN	Guinea	40713	Kaloum	0.57	0.67	0.78	77324	43550	6946
GIN	Guinea	40714	Matam	0.57	0.67	0.78	96099	54125	8632
GIN	Guinea	40715	Matoto	0.57	0.67	0.78	104725	58983	9407
GIN	Guinea	40716	Ratoma	0.57	0.67	0.78	89893	50630	8075
GIN	Guinea	40717	Dabola	0.57	0.67	0.78	43004	24221	3863
GIN	Guinea	40718	Dinguiraye	0.57	0.67	0.78	48291	27198	4338
GIN	Guinea	40719	Faranah	0.57	0.72	0.91	52273	18698	0
GIN	Guinea	40720	Kissidougou	0.57	0.67	0.78	76981	43357	6915
GIN	Guinea	40721	Kankan	0.57	0.67	0.78	105697	59531	9494
GIN	Guinea	40722	Kerouane	0.57	0.67	0.78	69227	38990	6218
GIN	Guinea	40723	Kouroussa	0.57	0.67	0.78	53293	30016	4787
GIN	Guinea	40724	Mandiana	0.87	0.90	0.91	0	0	0
GIN	Guinea	40725	Siguiri	0.57	0.67	0.78	114852	64687	10317

GIN	Guinea	40726	Coyah	0.57	0.67	0.78	100952	56858	9068
GIN	Guinea	40727	Dubreka	0.57	0.67	0.78	47304	26643	4249
GIN	Guinea	40728	Forecariah	0.57	0.67	0.78	102279	57605	9187
GIN	Guinea	40729	Fria	0.57	0.67	0.78	33638	18946	3022
GIN	Guinea	40730	Kindia	0.57	0.67	0.78	130267	73369	11702
GIN	Guinea	40731	Telemele	0.57	0.67	0.78	70363	39630	6321
GIN	Guinea	40732	Koubia	0.57	0.67	0.78	31115	17524	2795
GIN	Guinea	40733	Labe	0.57	0.67	0.78	88010	49569	7906
GIN	Guinea	40734	Lelouma	0.57	0.67	0.78	38580	21729	3466
GIN	Guinea	40735	Mali	0.57	0.67	0.78	57240	32239	5142
GIN	Guinea	40736	Tougue	0.57	0.67	0.78	43535	24520	3911
GIN	Guinea	40737	Dalaba	0.57	0.67	0.78	49941	28128	4486
GIN	Guinea	40738	Mamou	0.57	0.67	0.78	101750	57308	9140
GIN	Guinea	40739	Pita	0.57	0.67	0.78	72220	40675	6487
GIN	Guinea	40740	Beyla	0.57	0.67	0.78	58944	33198	5295
GIN	Guinea	40741	Gueckedou	0.57	0.67	0.78	191638	107934	17214
GIN	Guinea	40742	Lola	0.57	0.67	0.78	57852	32583	5197
GIN	Guinea	40743	Macenta	0.57	0.69	0.83	132825	65123	0
GIN	Guinea	40744	N'Zerekore	0.57	0.69	0.83	106815	52371	0
GIN	Guinea	40745	Yomou	0.57	0.67	0.78	77888	43868	6996
GMB	Gambia	16405	Banjul	0.76	0.77	0.77	711	565	558
GMB	Gambia	16406	Fulladu West	0.76	0.77	0.77	3773	3001	2960
GMB	Gambia	16407	Janjanbureh	0.76	0.77	0.77	139	110	109
GMB	Gambia	16408	Lower Saloum	0.76	0.77	0.77	712	566	558
GMB	Gambia	16409	Niamina Dankunku	0.76	0.77	0.77	364	289	285
GMB	Gambia	16410	Niamina East	0.76	0.77	0.77	1028	818	807
GMB	Gambia	16411	Niamina West	0.76	0.77	0.77	371	295	291
GMB	Gambia	16412	Niani	0.76	0.77	0.77	1489	1184	1168
GMB	Gambia	16413	Nianija	0.76	0.77	0.77	424	337	333
GMB	Gambia	16414	Sami	0.76	0.77	0.77	1278	1017	1003
GMB	Gambia	16415	Upper Saloum	0.76	0.77	0.77	853	679	669
GMB	Gambia	16416	Kombo Saint Mary	0.76	0.77	0.77	23938	19040	18782
GMB	Gambia	16417	Jarra Central	0.76	0.77	0.77	339	270	266

GMB	Gambia	16418	Jarra East	0.76	0.77	0.77	745	593	585
GMB	Gambia	16419	Jarra West	0.76	0.77	0.77	1372	1091	1076
GMB	Gambia	16420	Kiang Central	0.76	0.77	0.77	379	302	298
GMB	Gambia	16421	Kiang East	0.76	0.77	0.77	347	276	273
GMB	Gambia	16422	Kiang West	0.76	0.77	0.77	867	689	680
GMB	Gambia	16423	Central Baddibu	0.76	0.77	0.77	1119	890	878
GMB	Gambia	16424	Jokadu	0.76	0.77	0.77	1131	900	888
GMB	Gambia	16425	Lower Baddibu	0.76	0.77	0.77	1061	844	833
GMB	Gambia	16426	Lower Niumi	0.76	0.77	0.77	2569	2044	2016
GMB	Gambia	16427	Upper Baddibu	0.76	0.77	0.77	4052	3223	3179
GMB	Gambia	16428	Upper Niumi	0.76	0.77	0.77	1341	1067	1052
GMB	Gambia	16429	Fulladu East	0.76	0.77	0.77	5614	4465	4405
GMB	Gambia	16430	Kantora	0.76	0.77	0.77	2027	1612	1590
GMB	Gambia	16431	Sandu	0.76	0.77	0.77	940	747	737
GMB	Gambia	16432	Wuli	0.76	0.77	0.77	2081	1655	1633
GMB	Gambia	16433	Foni Bintang-karenai	0.76	0.77	0.77	827	658	649
GMB	Gambia	16434	Foni Bondali	0.76	0.77	0.77	312	248	245
GMB	Gambia	16435	Foni Brefet	0.76	0.77	0.77	634	504	498
GMB	Gambia	16436	Foni Jarrol	0.76	0.77	0.77	304	242	238
GMB	Gambia	16437	Foni Kansala	0.76	0.77	0.77	665	529	522
GMB	Gambia	16438	Kombo Central	0.76	0.77	0.77	6011	4781	4716
GMB	Gambia	16439	Kombo East	0.76	0.77	0.77	1472	1170	1155
GMB	Gambia	16440	Kombo North	0.76	0.77	0.77	10632	8456	8342
GMB	Gambia	16441	Kombo South	0.76	0.77	0.77	3708	2949	2909
GNB	Guinea-Bissau	17068	Bafata	0.18	0.18	0.18	63562	63562	63562
GNB	Guinea-Bissau	17069	Bambadinca	0.18	0.18	0.18	23455	23455	23455
GNB	Guinea-Bissau	17070	Contuboel	0.18	0.18	0.18	32465	32465	32465
GNB	Guinea-Bissau	17071	Galomaro/cosse	0.18	0.18	0.18	11049	11049	11049
GNB	Guinea-Bissau	17072	Gamamudo/ganadu	0.18	0.18	0.18	18417	18417	18417
GNB	Guinea-Bissau	17073	Xitole	0.18	0.18	0.18	13539	13539	13539
GNB	Guinea-Bissau	17074	Prabis	0.18	0.18	0.18	21688	21688	21688
GNB	Guinea-Bissau	17075	Quinhamel	0.18	0.18	0.18	31761	31761	31761
GNB	Guinea-Bissau	17076	Safim	0.18	0.18	0.18	15441	15441	15441

GNB	Guinea-Bissau	17077	Bolama	0.18	0.18	0.18	6403	6403	6403
GNB	Guinea-Bissau	17078	Bubaque	0.18	0.18	0.18	7321	7321	7321
GNB	Guinea-Bissau	17079	Caravela	0.18	0.18	0.18	3028	3028	3028
GNB	Guinea-Bissau	17080	Uno	0.18	0.18	0.18	5031	5031	5031
GNB	Guinea-Bissau	17081	Bigene	0.18	0.18	0.18	39741	39741	39741
GNB	Guinea-Bissau	17082	Bula	0.18	0.18	0.18	20985	20985	20985
GNB	Guinea-Bissau	17083	Cacheu/calequisse	0.18	0.18	0.18	16801	16801	16801
GNB	Guinea-Bissau	17084	Caio	0.18	0.18	0.18	9661	9661	9661
GNB	Guinea-Bissau	17085	Canchungo	0.18	0.18	0.18	31965	31965	31965
GNB	Guinea-Bissau	17086	Sao Domingos	0.18	0.18	0.18	25238	25238	25238
GNB	Guinea-Bissau	17087	Boe	0.18	0.18	0.18	7968	7968	7968
GNB	Guinea-Bissau	17088	Gabu	0.18	0.18	0.18	61441	61441	61441
GNB	Guinea-Bissau	17089	Pirada	0.18	0.18	0.18	22931	22931	22931
GNB	Guinea-Bissau	17090	Pitche	0.18	0.18	0.18	32209	32209	32209
GNB	Guinea-Bissau	17091	Sonaco	0.18	0.18	0.18	27793	27793	27793
GNB	Guinea-Bissau	17092	Bissora	0.18	0.18	0.18	39992	39992	39992
GNB	Guinea-Bissau	17093	Farim	0.18	0.18	0.18	33867	33867	33867
GNB	Guinea-Bissau	17094	Mansaba	0.18	0.18	0.18	36417	36417	36417
GNB	Guinea-Bissau	17095	Mansoa	0.18	0.18	0.18	35381	35381	35381
GNB	Guinea-Bissau	17096	Nhacra	0.18	0.18	0.18	15020	15020	15020
GNB	Guinea-Bissau	17097	Buba	0.18	0.18	0.18	12402	12402	12402
GNB	Guinea-Bissau	17098	Empada	0.18	0.18	0.18	11466	11466	11466
GNB	Guinea-Bissau	17099	Fulacunda	0.18	0.18	0.18	10823	10823	10823
GNB	Guinea-Bissau	17100	Tite	0.18	0.18	0.18	10484	10484	10484
GNB	Guinea-Bissau	17101	Sector Autonomo De Bissau	0.18	0.18	0.18	305434	305434	305434
GNB	Guinea-Bissau	17102	Bedanda	0.18	0.18	0.18	19647	19647	19647
GNB	Guinea-Bissau	17103	Cacine	0.18	0.18	0.18	10805	10805	10805
GNB	Guinea-Bissau	17104	Catio	0.18	0.18	0.18	24532	24532	24532
GNB	Guinea-Bissau	17105	Komo	0.18	0.18	0.18	7216	7216	7216
GNB	Guinea-Bissau	17106	Quebo	0.18	0.18	0.18	11096	11096	11096
GNQ	Equatorial Guinea	15829	Admin unit not available	0.00	0.00	0.00	145100	145100	145100
GNQ	Equatorial Guinea	15830	Admin unit not available	0.00	0.00	0.00	19716	19716	19716
GNQ	Equatorial Guinea	15831	Admin unit not available	0.00	0.00	0.00	83204	83204	83204

GNQ	Equatorial Guinea	15832	Admin unit not available	0.00	0.00	0.00	109932	109932	109932
GNQ	Equatorial Guinea	15833	Admin unit not available	0.00	0.00	0.00	190273	190273	190273
GNQ	Equatorial Guinea	15834	Admin unit not available	0.00	0.00	0.00	104070	104070	104070
GUF	French Guiana	16334	Camopi	0.00	0.00	0.00	1722	1722	1722
GUF	French Guiana	16335	Iracoubo	0.00	0.00	0.00	1923	1923	1923
GUF	French Guiana	16336	Kourou	0.00	0.00	0.00	23244	23244	23244
GUF	French Guiana	16337	Matoury	0.00	0.00	0.00	26132	26132	26132
GUF	French Guiana	16338	Montsinery	0.00	0.00	0.00	3233	3233	3233
GUF	French Guiana	16339	Ouanary	0.00	0.00	0.00	72	72	72
GUF	French Guiana	16340	Regina	0.00	0.00	0.00	739	739	739
GUF	French Guiana	16341	Remire (montjoly)	0.00	0.00	0.00	61298	61298	61298
GUF	French Guiana	16342	Roura	0.00	0.00	0.00	2887	2887	2887
GUF	French Guiana	16343	Saint Georges	0.00	0.00	0.00	6067	6067	6067
GUF	French Guiana	16344	Saint-elie	0.00	0.00	0.00	782	782	782
GUF	French Guiana	16345	Sinnamary	0.00	0.00	0.00	2715	2715	2715
GUF	French Guiana	16346	Tonate (macouria)	0.00	0.00	0.00	9793	9793	9793
GUF	French Guiana	16347	Apatou	0.00	0.00	0.00	7392	7392	7392
GUF	French Guiana	16348	Awala-yalimapo	0.00	0.00	0.00	1233	1233	1233
GUF	French Guiana	16349	Grand-santi	0.00	0.00	0.00	4475	4475	4475
GUF	French Guiana	16350	Mana	0.00	0.00	0.00	9278	9278	9278
GUF	French Guiana	16351	Maripasoula	0.00	0.00	0.00	9086	9086	9086
GUF	French Guiana	16352	Papaichton (pompidou)	0.00	0.00	0.00	5352	5352	5352
GUF	French Guiana	16353	Saint-laurent-du-maroni	0.00	0.00	0.00	43069	43069	43069
GUF	French Guiana	16354	Saul	0.00	0.00	0.00	158	158	158
GUY	Guyana	17140	Barima/amakura	0.25	0.25	0.25	9403	9403	9403
GUY	Guyana	17141	Waini	0.25	0.25	0.25	5975	5975	5975
GUY	Guyana	17142	Cuyuni	0.25	0.25	0.25	2501	2501	2501
GUY	Guyana	17143	Mazaruni/left Bank Essequibo River	0.25	0.25	0.25	9610	9610	9610
GUY	Guyana	17144	Buxton/mahaica	0.25	0.25	0.25	43071	43071	43071
GUY	Guyana	17145	Moblissa/la Reconnaissance	0.25	0.25	0.25	129131	129131	129131
GUY	Guyana	17146	Black Bush Polder Frontlands And Extension	0.25	0.25	0.25	12575	12575	12575
GUY	Guyana	17147	East Berbice/west Canje	0.25	0.25	0.25	10790	10790	10790
GUY	Guyana	17148	East Canje/east Coast Berbice	0.25	0.25	0.25	23006	23006	23006

GUY	Guyana	17149	Left Bank Upper Canje/ikuruwa/corentyne	0.25	0.25	0.25	314	314	314
GUY	Guyana	17150	Lower Corentyne River/canje	0.25	0.25	0.25	9426	9426	9426
GUY	Guyana	17151	Upper Canje/corentyne	0.25	0.25	0.25	540	540	540
GUY	Guyana	17152	Upper Corentyne	0.25	0.25	0.25	582	582	582
GUY	Guyana	17153	Bonasika/boerasirie	0.25	0.25	0.25	23584	23584	23584
GUY	Guyana	17154	Essequibo Islands/left Bank Essequibo River	0.25	0.25	0.25	6761	6761	6761
GUY	Guyana	17155	Lower West Demerara River	0.25	0.25	0.25	33530	33530	33530
GUY	Guyana	17156	Mahaica/mahaicony	0.25	0.25	0.25	4884	4884	4884
GUY	Guyana	17157	Mahaicony/berbice	0.25	0.25	0.25	25351	25351	25351
GUY	Guyana	17158	Moruka/pomeroon	0.25	0.25	0.25	6173	6173	6173
GUY	Guyana	17159	Somerset And Berks/supenaam River	0.25	0.25	0.25	19229	19229	19229
GUY	Guyana	17160	Ireng/upper Potaro	0.25	0.25	0.25	3307	3307	3307
GUY	Guyana	17161	Lower Potaro/lower Lady Smith Creek	0.25	0.25	0.25	3243	3243	3243
GUY	Guyana	17162	Right Bank Essequibo/upper Demerara	0.25	0.25	0.25	18549	18549	18549
GUY	Guyana	17163	Torani/bulletwood	0.25	0.25	0.25	3659	3659	3659
GUY	Guyana	17164	Rupununi West	0.25	0.25	0.25	12929	12929	12929
GUY	Guyana	17165	Rewai/upper Essequibo	0.25	0.25	0.25	1359	1359	1359
KEN	Kenya	61034	Admin unit not available	0.00	0.00	0.00	14809	14809	14809
KEN	Kenya	51334	Kirinyaga	0.00	0.00	0.00	520224	520224	520224
KEN	Kenya	51337	Nyandarua	0.00	0.00	0.00	578584	578584	578584
KEN	Kenya	51338	Nyeri	0.00	0.00	0.00	685906	685906	685906
KEN	Kenya	51341	Kwale	0.00	0.00	0.00	626147	626147	626147
KEN	Kenya	51342	Lamu	0.00	0.00	0.00	89187	89187	89187
KEN	Kenya	51343	Malindi	0.00	0.00	0.00	388851	388851	388851
KEN	Kenya	51345	Taita Taveta	0.00	0.00	0.00	284844	284844	284844
KEN	Kenya	51346	Tana River	0.00	0.00	0.00	234071	234071	234071
KEN	Kenya	51348	Isiolo	0.00	0.00	0.00	142907	142907	142907
KEN	Kenya	51350	Machakos	0.00	0.00	0.00	1087505	1087505	1087505
KEN	Kenya	51351	Makueni	0.00	0.00	0.00	858963	858963	858963
KEN	Kenya	51360	Nairobi	0.00	0.00	0.00	3487590	3487590	3487590
KEN	Kenya	51363	Mandera	0.00	0.00	0.00	984372	984372	984372
KEN	Kenya	51364	Wajir	0.00	0.00	0.00	652102	652102	652102
KEN	Kenya	51372	Nyamira	0.00	0.00	0.00	581362	581362	581362

KEN	Kenya	51380	Kajiado	0.00	0.00	0.00	678246	678246	678246
KEN	Kenya	51382	Kericho	0.00	0.00	0.00	576395	576395	576395
KEN	Kenya	51384	Laikipia	0.00	0.00	0.00	398425	398425	398425
KEN	Kenya	51386	Nakuru	0.00	0.00	0.00	1624299	1624299	1624299
KEN	Kenya	51389	Samburu	0.00	0.00	0.00	218820	218820	218820
KEN	Kenya	51391	Trans Nzoia	0.00	0.00	0.00	814027	814027	814027
KEN	Kenya	51392	Turkana	0.00	0.00	0.00	785345	785345	785345
KEN	Kenya	51393	Uasin Gishu	0.00	0.00	0.00	916044	916044	916044
KEN	Kenya	51394	West Pokot	0.00	0.00	0.00	498760	498760	498760
KEN	Kenya	61001	Admin unit not available	0.00	0.00	0.00	1503	1503	1503
KEN	Kenya	61005	Admin unit not available	0.00	0.00	0.00	7370	7370	7370
KEN	Kenya	61009	Admin unit not available	0.00	0.00	0.00	56880	56880	56880
KEN	Kenya	1013969	Baringo	0.63	0.63	0.63	112712	112712	112712
KEN	Kenya	1013970	Bomet	0.00	0.00	0.00	865355	865355	865355
KEN	Kenya	1013971	Bungoma	0.00	0.00	0.00	1358527	1358527	1358527
KEN	Kenya	1013972	Busia	0.00	0.00	0.00	673078	673078	673078
KEN	Kenya	1013973	Elgeyo Marakwet	0.63	0.63	0.63	75283	75283	75283
KEN	Kenya	1013974	Embu	0.00	0.00	0.00	508229	508229	508229
KEN	Kenya	1013975	Garissa	0.00	0.00	0.00	617117	617117	617117
KEN	Kenya	1013976	Homa Bay	0.00	0.00	0.00	899250	899250	899250
KEN	Kenya	1013977	Kiambu	0.00	0.00	0.00	1865247	1865247	1865247
KEN	Kenya	1013978	Kisumu	0.00	0.00	0.00	974966	974966	974966
KEN	Kenya	1013979	Kitui	0.00	0.00	0.00	991566	991566	991566
KEN	Kenya	1013980	Marsabit	0.00	0.00	0.00	279292	279292	279292
KEN	Kenya	1013981	Mombasa	0.00	0.00	0.00	1600430	1600430	1600430
KEN	Kenya	1013982	Muranga	0.00	0.00	0.00	733644	733644	733644
KEN	Kenya	1013983	Narok	0.00	0.00	0.00	830680	830680	830680
KEN	Kenya	1013984	Siaya	0.00	0.00	0.00	789973	789973	789973
KEN	Kenya	1013985	Tharaka	0.00	0.00	0.00	359024	359024	359024
KEN	Kenya	1013986	Nandi	0.00	0.00	0.00	747614	747614	747614
KEN	Kenya	1013987	Meru	0.00	0.00	0.00	1323922	1323922	1323922
KEN	Kenya	1013988	Migori	0.00	0.00	0.00	878164	878164	878164
KEN	Kenya	1013989	Kisii	0.00	0.00	0.00	1123555	1123555	1123555

KEN	Kenya	1013990	Kakamega	0.00	0.00	0.00	1394543	1394543	1394543
KEN	Kenya	1013991	Vihiga	0.00	0.00	0.00	771923	771923	771923
LBR	Liberia	39172	Klay	0.22	0.22	0.22	82961	82961	82961
LBR	Liberia	39173	Mecca	0.22	0.22	0.22	5754	5754	5754
LBR	Liberia	39174	Fuamah	0.37	0.38	0.38	17888	17385	17219
LBR	Liberia	39175	Jorquelleh	0.37	0.38	0.38	45008	43744	43325
LBR	Liberia	39176	Kokoyah	0.37	0.38	0.38	15335	14904	14762
LBR	Liberia	39177	Panta-Kpa	0.37	0.38	0.38	22442	21811	21603
LBR	Liberia	39178	Salala	0.37	0.38	0.38	21157	20562	20366
LBR	Liberia	39179	Sanayea	0.37	0.38	0.38	17432	16943	16780
LBR	Liberia	39180	Suakoko	0.37	0.38	0.38	34992	34009	33684
LBR	Liberia	39181	Zota	0.37	0.38	0.38	9932	9653	9561
LBR	Liberia	39182	Belle Yella	0.22	0.22	0.22	13946	13946	13946
LBR	Liberia	39183	Bokomu	0.22	0.22	0.22	14056	14056	14056
LBR	Liberia	39184	Bopolu	0.22	0.22	0.22	14707	14707	14707
LBR	Liberia	39185	Gbarma	0.22	0.22	0.22	12111	12111	12111
LBR	Liberia	39186	Kongba	0.22	0.22	0.22	9130	9130	9130
LBR	Liberia	39187	District #1	0.85	0.85	0.86	0	0	0
LBR	Liberia	39188	District #2	0.85	0.85	0.86	0	0	0
LBR	Liberia	39189	District #3	0.85	0.85	0.86	0	0	0
LBR	Liberia	39190	District #4	0.85	0.85	0.86	0	0	0
LBR	Liberia	39191	Garwula	0.22	0.22	0.22	26916	26916	26916
LBR	Liberia	39192	Gola Konneh	0.22	0.22	0.22	22236	22236	22236
LBR	Liberia	39193	Porkpa	0.22	0.22	0.22	28684	28684	28684
LBR	Liberia	39194	Robertsport	0.22	0.22	0.22	4541	4541	4541
LBR	Liberia	39195	Tewor	0.22	0.22	0.22	11773	11773	11773
LBR	Liberia	39196	Gbarzon	0.85	0.85	0.85	0	0	0
LBR	Liberia	39197	Konobo	0.85	0.85	0.85	0	0	0
LBR	Liberia	39198	Tchien	0.85	0.85	0.85	0	0	0
LBR	Liberia	39199	Buah	0.22	0.22	0.22	6102	6102	6102
LBR	Liberia	39200	Sasstown	0.22	0.22	0.22	9569	9569	9569
LBR	Liberia	39201	Trehn	0.22	0.22	0.22	10912	10912	10912
LBR	Liberia	39202	Upper Kru Coast	0.22	0.22	0.22	18206	18206	18206

LBR	Liberia	39203	Foya	0.50	0.50	0.50	25651	25651	25651
LBR	Liberia	39204	Kolahun	0.50	0.50	0.50	28872	28872	28872
LBR	Liberia	39205	Salayea	0.22	0.22	0.22	21929	21929	21929
LBR	Liberia	39206	Vahun	0.22	0.22	0.22	12919	12919	12919
LBR	Liberia	39207	Voinjama	0.82	0.85	0.88	0	0	0
LBR	Liberia	39208	Zorzor	0.65	0.68	0.71	8179	6502	4840
LBR	Liberia	39209	Firestone	0.85	0.85	0.85	0	0	0
LBR	Liberia	39210	Gibi	0.85	0.85	0.85	0	0	0
LBR	Liberia	39211	Kakata	0.85	0.85	0.85	0	0	0
LBR	Liberia	39212	Mambah-Kaba	0.85	0.85	0.85	0	0	0
LBR	Liberia	39213	Barrobo	0.22	0.22	0.22	21813	21813	21813
LBR	Liberia	39214	Pleebo/Sodeken	0.22	0.22	0.22	77015	77015	77015
LBR	Liberia	39215	Careysburg	0.22	0.22	0.22	13205	13205	13205
LBR	Liberia	39216	Greater Monrovia	0.22	0.22	0.22	499053	499053	499053
LBR	Liberia	39217	St Paul River	0.22	0.22	0.22	164332	164332	164332
LBR	Liberia	39218	Todee	0.22	0.22	0.22	43525	43525	43525
LBR	Liberia	39219	Gbehlageh	0.82	0.86	0.90	0	0	0
LBR	Liberia	39220	Saclepea-Mah	0.82	0.86	0.90	0	0	0
LBR	Liberia	39221	Sanniquelleh-Mahn	0.82	0.86	0.90	0	0	0
LBR	Liberia	39222	Tappita	0.82	0.86	0.90	0	0	0
LBR	Liberia	39223	Yarwein-Mehnsohnnoh	0.82	0.86	0.90	0	0	0
LBR	Liberia	39224	Zoegeh	0.82	0.86	0.90	0	0	0
LBR	Liberia	39225	Gbeapo	0.22	0.22	0.22	40755	40755	40755
LBR	Liberia	39226	Webbo	0.22	0.22	0.22	20428	20428	20428
LBR	Liberia	39227	Morweh	0.22	0.22	0.22	17674	17674	17674
LBR	Liberia	39228	Timbo	0.22	0.22	0.22	36337	36337	36337
LBR	Liberia	39229	Butaw	0.22	0.22	0.22	12830	12830	12830
LBR	Liberia	39230	Dugbe River	0.22	0.22	0.22	3821	3821	3821
LBR	Liberia	39231	Greenville	0.22	0.22	0.22	11172	11172	11172
LBR	Liberia	39232	Jaedae Jaedepo	0.22	0.22	0.22	14683	14683	14683
LBR	Liberia	39233	Juarzon	0.22	0.22	0.22	15892	15892	15892
LBR	Liberia	39234	Kpayan	0.22	0.22	0.22	7257	7257	7257
LBR	Liberia	39235	Pyneston	0.22	0.22	0.22	7229	7229	7229

MLI	Mali	19366	Commune 1	0.38	0.45	0.48	168035	138290	125699
MLI	Mali	19367	Commune 2	0.38	0.45	0.48	79327	65285	59341
MLI	Mali	19368	Commune 3	0.38	0.45	0.48	95016	78197	71077
MLI	Mali	19369	Commune 4	0.38	0.45	0.48	137071	112807	102536
MLI	Mali	19370	Commune 5	0.38	0.45	0.48	179536	147755	134303
MLI	Mali	19371	Commune 6	0.38	0.45	0.48	285976	235354	213925
MLI	Mali	19372	Ansongo	0.38	0.45	0.48	63378	52159	47410
MLI	Mali	19373	Bourem	0.38	0.45	0.48	53917	44373	40333
MLI	Mali	19374	Gao	0.38	0.45	0.48	119084	98005	89081
MLI	Mali	19375	Menaka	0.38	0.45	0.48	23618	19437	17667
MLI	Mali	19376	Bafoulabe	0.70	0.76	0.85	26893	10614	0
MLI	Mali	19377	Diema	0.50	0.57	0.60	72121	56825	49783
MLI	Mali	19378	Kayes	0.69	0.75	0.86	66875	30350	0
MLI	Mali	19379	Kenieba	0.73	0.78	0.86	14673	4163	0
MLI	Mali	19380	Kita	0.76	0.83	0.86	17953	0	0
MLI	Mali	19381	Nioro	0.50	0.57	0.60	77654	61185	53602
MLI	Mali	19382	Yelimane	0.50	0.57	0.60	57948	45658	39999
MLI	Mali	19383	Abeibara	0.38	0.45	0.48	4300	3539	3217
MLI	Mali	19384	Kidal	0.38	0.45	0.48	13228	10886	9895
MLI	Mali	19385	Tessalit	0.38	0.45	0.48	9329	7677	6978
MLI	Mali	19386	Tin-essako	0.38	0.45	0.48	3848	3167	2878
MLI	Mali	19387	Banamba	0.50	0.57	0.60	61501	48458	42452
MLI	Mali	19388	Dioila	0.50	0.57	0.60	164645	129727	113649
MLI	Mali	19389	Kangaba	0.76	0.80	0.86	4128	54	0
MLI	Mali	19390	Kati	0.76	0.80	0.86	44505	583	0
MLI	Mali	19391	Kolokani	0.60	0.64	0.66	54921	43787	37674
MLI	Mali	19392	Koulikoro	0.50	0.57	0.60	74291	58535	51280
MLI	Mali	19393	Nara	0.50	0.57	0.60	80956	63786	55881
MLI	Mali	19394	Bandiagara	0.50	0.57	0.60	105232	82914	72638
MLI	Mali	19395	Bankass	0.50	0.57	0.60	85074	67031	58724
MLI	Mali	19396	Djenne	0.50	0.57	0.60	67836	53449	46825
MLI	Mali	19397	Douentza	0.50	0.57	0.60	85864	67653	59269
MLI	Mali	19398	Koro	0.50	0.57	0.60	118361	93259	81701

MLI	Mali	19399	Mopti	0.50	0.57	0.60	126677	99811	87441
MLI	Mali	19400	Tenenkou	0.50	0.57	0.60	59862	47167	41321
MLI	Mali	19401	Youwarou	0.50	0.57	0.60	34996	27574	24157
MLI	Mali	19402	Baroueli	0.50	0.57	0.60	71241	56132	49175
MLI	Mali	19403	Bla	0.50	0.57	0.60	93726	73848	64696
MLI	Mali	19404	Ke-macina	0.50	0.57	0.60	75747	59682	52285
MLI	Mali	19405	Niono	0.50	0.57	0.60	126214	99446	87121
MLI	Mali	19406	San	0.50	0.57	0.60	112573	88698	77705
MLI	Mali	19407	Segou	0.50	0.57	0.60	231626	182502	159884
MLI	Mali	19408	Tominian	0.50	0.57	0.60	71349	56217	49250
MLI	Mali	19409	Bougouni	0.50	0.57	0.60	152066	119815	104966
MLI	Mali	19410	Kadiolo	0.50	0.58	0.61	80326	60335	50881
MLI	Mali	19411	Kolondieba	0.50	0.58	0.61	65233	48998	41320
MLI	Mali	19412	Koutiala	0.50	0.57	0.60	191650	151004	132290
MLI	Mali	19413	Sikasso	0.50	0.57	0.60	247261	194821	170676
MLI	Mali	19414	Yanfolila	0.58	0.63	0.68	53599	39737	27976
MLI	Mali	19415	Yorosso	0.50	0.57	0.60	69976	55136	48302
MLI	Mali	19416	Dire	0.38	0.45	0.48	50312	41406	37636
MLI	Mali	19417	Goundam	0.38	0.45	0.48	73449	60447	54943
MLI	Mali	19418	Gourma-rharous	0.38	0.45	0.48	54484	44839	40757
MLI	Mali	19419	Niafunke	0.38	0.45	0.48	85409	70290	63891
MLI	Mali	19420	Tombouctou	0.38	0.45	0.48	62163	51159	46501
MRT	Mauritania	19490	Aoujeft	0.02	0.04	0.04	25804	25274	25072
MRT	Mauritania	19491	Atar	0.02	0.04	0.04	49511	48495	48107
MRT	Mauritania	19492	Chinguetti	0.02	0.04	0.04	8549	8374	8307
MRT	Mauritania	19493	Ouadane	0.02	0.04	0.04	4842	4743	4705
MRT	Mauritania	19494	Aftout (barkewol)	0.02	0.04	0.04	79541	77909	77285
MRT	Mauritania	19495	Boumdeid	0.02	0.04	0.04	11124	10895	10808
MRT	Mauritania	19496	Guerou	0.02	0.04	0.04	39959	39139	38825
MRT	Mauritania	19497	Kankossa	0.02	0.04	0.04	80598	78944	78312
MRT	Mauritania	19498	Kiffa	0.02	0.04	0.04	97785	95778	95011
MRT	Mauritania	19499	Aleg	0.02	0.04	0.04	86816	85034	84353
MRT	Mauritania	19500	Bababe	0.02	0.04	0.04	43144	42258	41920

MRT	Mauritania	19501	Boghe	0.02	0.04	0.04	80080	78436	77808
MRT	Mauritania	19502	M Bagne	0.02	0.04	0.04	46130	45183	44821
MRT	Mauritania	19503	Magta-lahjar	0.02	0.04	0.04	60399	59160	58686
MRT	Mauritania	19504	Nouadhibou	0.02	0.04	0.04	99923	97872	97088
MRT	Mauritania	19505	Kaedi	0.02	0.04	0.04	106167	103988	103155
MRT	Mauritania	19506	M Bout	0.02	0.04	0.04	99270	97232	96453
MRT	Mauritania	19507	Maghama	0.02	0.04	0.04	56170	55017	54576
MRT	Mauritania	19508	Monguel	0.02	0.04	0.04	41226	40380	40056
MRT	Mauritania	19509	Ould Yenge	0.02	0.04	0.04	62724	61436	60944
MRT	Mauritania	19510	Selibaby	0.02	0.04	0.04	160411	157118	155860
MRT	Mauritania	19511	Amourj	0.02	0.04	0.04	88969	87143	86445
MRT	Mauritania	19512	Bassikounou	0.02	0.04	0.04	45641	44704	44346
MRT	Mauritania	19513	Djigueni	0.02	0.04	0.04	56048	54897	54457
MRT	Mauritania	19514	Nema	0.02	0.04	0.04	80631	78976	78344
MRT	Mauritania	19515	Oualata	0.02	0.04	0.04	15511	15193	15071
MRT	Mauritania	19516	Timbedra	0.02	0.04	0.04	72131	70650	70084
MRT	Mauritania	19517	Aioun Al Atrous	0.02	0.04	0.04	59251	58035	57570
MRT	Mauritania	19518	Kobenni	0.02	0.04	0.04	90786	88923	88211
MRT	Mauritania	19519	Tamchekket	0.02	0.04	0.04	39465	38655	38345
MRT	Mauritania	19520	Tintane	0.02	0.04	0.04	81012	79350	78714
MRT	Mauritania	19521	Akjoujt	0.02	0.04	0.04	14965	14658	14541
MRT	Mauritania	19522	Nouakchott	0.02	0.04	0.04	771418	755585	749532
MRT	Mauritania	19523	Moudjeria	0.02	0.04	0.04	46914	45951	45583
MRT	Mauritania	19524	Tichit	0.02	0.04	0.04	6130	6004	5956
MRT	Mauritania	19525	Tidjikja	0.02	0.04	0.04	45053	44128	43775
MRT	Mauritania	19526	Bir Mogrein	0.02	0.04	0.04	3513	3441	3414
MRT	Mauritania	19527	F Derik	0.02	0.04	0.04	7219	7071	7014
MRT	Mauritania	19528	Zouerate	0.02	0.04	0.04	41736	40879	40552
MRT	Mauritania	19529	Boutilimit	0.02	0.04	0.04	72552	71063	70493
MRT	Mauritania	19530	Keur Massene	0.02	0.04	0.04	36950	36191	35901
MRT	Mauritania	19531	Mederdra	0.02	0.04	0.04	39852	39034	38721
MRT	Mauritania	19532	Ouad Naga	0.02	0.04	0.04	34547	33838	33567
MRT	Mauritania	19533	R Kiz	0.02	0.04	0.04	90317	88463	87754

MRT	Mauritania	19534	Rosso	0.02	0.04	0.04	72018	70540	69975
NER	Niger	22551	Arlit	0.31	0.32	0.33	101365	98765	97723
NER	Niger	22552	Bilma	0.31	0.32	0.33	16850	16417	16244
NER	Niger	22553	Tchighozerine	0.31	0.32	0.33	209061	203699	201550
NER	Niger	22554	Diffa	0.31	0.32	0.33	110494	107660	106524
NER	Niger	22555	Maine-soroa	0.31	0.32	0.33	104400	101723	100649
NER	Niger	22556	N'guigmi	0.31	0.32	0.33	49487	48217	47709
NER	Niger	22557	Birni N'gaoure	0.31	0.32	0.33	230672	224755	222384
NER	Niger	22558	Dogondoutchi	0.31	0.32	0.33	450316	438766	434137
NER	Niger	22559	Dosso	0.31	0.32	0.33	268965	262066	259302
NER	Niger	22560	Gaya	0.31	0.32	0.33	214023	208534	206334
NER	Niger	22561	Loga	0.31	0.32	0.33	170171	165806	164057
NER	Niger	22562	Aguie	0.31	0.32	0.33	307784	299890	296726
NER	Niger	22563	Dakoro	0.31	0.32	0.33	343318	334513	330983
NER	Niger	22564	Guidan Roumji	0.31	0.32	0.33	285581	278256	275320
NER	Niger	22565	Madarounfa	0.31	0.32	0.33	452074	440479	435832
NER	Niger	22566	Mayahi	0.31	0.32	0.33	330978	322489	319087
NER	Niger	22567	Tessaoua	0.31	0.32	0.33	320467	312248	308954
NER	Niger	22568	Commune 1	0.31	0.32	0.33	380416	370659	366749
NER	Niger	22569	Commune 2	0.31	0.32	0.33	354260	345174	341533
NER	Niger	22570	Commune 3	0.31	0.32	0.33	121942	118814	117561
NER	Niger	22571	Birni N'konni	0.31	0.32	0.33	306713	298847	295694
NER	Niger	22572	Bouza	0.31	0.32	0.33	238777	232653	230198
NER	Niger	22573	Illela	0.31	0.32	0.33	228338	222482	220134
NER	Niger	22574	Keita	0.31	0.32	0.33	211544	206119	203944
NER	Niger	22575	Madaoua	0.31	0.32	0.33	220842	215178	212908
NER	Niger	22576	Tahoua	0.31	0.32	0.33	280358	273168	270286
NER	Niger	22577	Tchin Tabaradene	0.31	0.32	0.33	161415	157276	155616
NER	Niger	22578	Filingue	0.31	0.32	0.33	342111	333337	329820
NER	Niger	22579	Kollo	0.31	0.32	0.33	258879	252239	249578
NER	Niger	22580	Ouallam	0.31	0.32	0.33	238867	232740	230285
NER	Niger	22581	Say	0.31	0.32	0.33	233848	227850	225446
NER	Niger	22582	Tera	0.31	0.32	0.33	359474	350254	346559

NER	Niger	22583	Tillaberi	0.31	0.32	0.33	187500	182691	180764
NER	Niger	22584	Goure	0.31	0.32	0.33	200195	195060	193002
NER	Niger	22585	Magaria	0.31	0.32	0.33	399297	389056	384951
NER	Niger	22586	Matamey	0.31	0.32	0.33	218189	212593	210350
NER	Niger	22587	Miria	0.31	0.32	0.33	683437	665909	658883
NER	Niger	22588	Tanout	0.31	0.32	0.33	247130	240792	238251
NGA	Nigeria	22589	Aba	0.15	0.15	0.15	449993	449993	449993
NGA	Nigeria	22590	Afikpo	0.15	0.15	0.15	131509	131509	131509
NGA	Nigeria	22591	Afikposo	0.15	0.15	0.15	132841	132841	132841
NGA	Nigeria	22592	Arochukw	0.15	0.15	0.15	135145	135145	135145
NGA	Nigeria	22593	Bende	0.15	0.15	0.15	159461	159461	159461
NGA	Nigeria	22594	Ikwuano	0.15	0.15	0.15	118249	118249	118249
NGA	Nigeria	22595	Isiala-n	0.15	0.15	0.15	172776	172776	172776
NGA	Nigeria	22596	Isuikwua	0.15	0.15	0.15	197923	197923	197923
NGA	Nigeria	22597	Obioma-n	0.15	0.15	0.15	457677	457677	457677
NGA	Nigeria	22598	Ohafia Abia	0.15	0.15	0.15	197728	197728	197728
NGA	Nigeria	22599	Ohaozara	0.15	0.15	0.15	194942	194942	194942
NGA	Nigeria	22600	Onicha	0.15	0.15	0.15	164838	164838	164838
NGA	Nigeria	22601	Ukwa	0.15	0.15	0.15	84892	84892	84892
NGA	Nigeria	22602	Ukwaeast	0.15	0.15	0.15	143303	143303	143303
NGA	Nigeria	22603	Umuahia	0.15	0.15	0.15	337953	337953	337953
NGA	Nigeria	22604	Fufore	0.20	0.27	0.30	138110	121237	114353
NGA	Nigeria	22605	Ganye	0.20	0.27	0.30	158421	139066	131170
NGA	Nigeria	22606	Gombi	0.20	0.27	0.30	98093	86108	81219
NGA	Nigeria	22607	Guyuk	0.20	0.27	0.30	123501	108412	102256
NGA	Nigeria	22608	Hong	0.20	0.27	0.30	142564	125147	118041
NGA	Nigeria	22609	Jada	0.20	0.27	0.30	98458	86429	81521
NGA	Nigeria	22610	Madagali	0.20	0.27	0.30	93907	82433	77753
NGA	Nigeria	22611	Maiha	0.20	0.27	0.30	100432	88162	83156
NGA	Nigeria	22612	Mayo-bel	0.20	0.27	0.30	110622	97106	91593
NGA	Nigeria	22613	Michika	0.20	0.27	0.30	99625	87453	82487
NGA	Nigeria	22614	Mubi	0.20	0.27	0.30	176467	154907	146111
NGA	Nigeria	22615	Numan	0.20	0.27	0.30	302867	265864	250768

NGA	Nigeria	22616	Shelleng	0.20	0.27	0.30	125401	110080	103830
NGA	Nigeria	22617	Song	0.20	0.27	0.30	260730	228876	215880
NGA	Nigeria	22618	Yola	0.20	0.27	0.30	355150	311759	294057
NGA	Nigeria	22619	Abak	0.56	0.65	0.76	32699	19995	5429
NGA	Nigeria	22620	Eket	0.56	0.65	0.76	51360	31406	8527
NGA	Nigeria	22621	Ekpe-ata	0.56	0.65	0.76	32628	19952	5417
NGA	Nigeria	22622	Essien-u	0.56	0.65	0.76	49305	30149	8186
NGA	Nigeria	22623	Etimekpo	0.56	0.65	0.76	36351	22228	6035
NGA	Nigeria	22624	Etinan	0.56	0.65	0.76	38499	23542	6392
NGA	Nigeria	22625	Ikono	0.56	0.65	0.76	48703	29781	8086
NGA	Nigeria	22626	Ikot-aba	0.56	0.65	0.76	51199	31307	8501
NGA	Nigeria	22627	Ikot-ekp	0.56	0.65	0.76	77558	47426	12877
NGA	Nigeria	22628	Ini	0.56	0.65	0.76	34859	21316	5788
NGA	Nigeria	22629	Itu	0.56	0.65	0.76	94972	58074	15768
NGA	Nigeria	22630	Mbo	0.56	0.65	0.76	33929	20747	5633
NGA	Nigeria	22631	Mkpat-en	0.56	0.65	0.76	40233	24602	6680
NGA	Nigeria	22632	Nsit-ibo	0.56	0.65	0.76	51249	31338	8509
NGA	Nigeria	22633	Nsit-ubi	0.56	0.65	0.76	35653	21801	5920
NGA	Nigeria	22634	Okobo	0.56	0.65	0.76	47390	28979	7868
NGA	Nigeria	22635	Onna	0.56	0.65	0.76	41093	25128	6823
NGA	Nigeria	22636	Oron	0.56	0.65	0.76	56807	34737	9432
NGA	Nigeria	22637	Oruk-ana	0.56	0.65	0.76	47970	29333	7965
NGA	Nigeria	22638	Ukanafun	0.56	0.65	0.76	40601	24827	6741
NGA	Nigeria	22639	Uquoiben	0.56	0.65	0.76	46850	28648	7779
NGA	Nigeria	22640	Uruan	0.56	0.65	0.76	64364	39358	10687
NGA	Nigeria	22641	Urueoffo	0.56	0.65	0.76	20046	12258	3328
NGA	Nigeria	22642	Uyo	0.56	0.65	0.76	126819	77548	21056
NGA	Nigeria	22643	Aguata	0.20	0.28	0.32	319952	276319	256630
NGA	Nigeria	22644	Anambra	0.20	0.28	0.32	117868	101794	94540
NGA	Nigeria	22645	Anaocha	0.20	0.28	0.32	243179	210016	195051
NGA	Nigeria	22646	Awkanort	0.20	0.28	0.32	124405	107440	99784
NGA	Nigeria	22647	Awkasout	0.20	0.28	0.32	207411	179125	166362
NGA	Nigeria	22648	Idemili	0.20	0.28	0.32	558269	482135	447781

NGA	Nigeria	22649	Ihiala	0.20	0.28	0.32	254891	220131	204445
NGA	Nigeria	22650	Njikoka	0.20	0.28	0.32	245417	211948	196846
NGA	Nigeria	22651	Nnewinort	0.20	0.28	0.32	185078	159838	148449
NGA	Nigeria	22652	Nnewisou	0.20	0.28	0.32	211025	182246	169260
NGA	Nigeria	22653	Ogbaru	0.20	0.28	0.32	61775	53350	49549
NGA	Nigeria	22654	Onitshan	0.20	0.28	0.32	320050	276403	256708
NGA	Nigeria	22655	Orumbano	0.20	0.28	0.32	193325	166961	155064
NGA	Nigeria	22656	Orumbaso	0.20	0.28	0.32	147830	127669	118572
NGA	Nigeria	22657	Oyi	0.20	0.28	0.32	299751	258872	240427
NGA	Nigeria	22658	Akko	0.13	0.13	0.13	248444	248444	248444
NGA	Nigeria	22659	Alkaleri	0.20	0.28	0.32	346747	299342	277908
NGA	Nigeria	22660	Balanga	0.13	0.13	0.13	188504	188504	188504
NGA	Nigeria	22661	Bauchi	0.20	0.28	0.32	459766	396910	368489
NGA	Nigeria	22662	Billiri	0.13	0.13	0.13	176591	176591	176591
NGA	Nigeria	22663	Darazo	0.20	0.28	0.32	193854	167352	155369
NGA	Nigeria	22664	Dass	0.20	0.28	0.32	63221	54578	50670
NGA	Nigeria	22665	Dukku	0.13	0.13	0.13	179970	179970	179970
NGA	Nigeria	22666	Gamawa	0.20	0.28	0.32	214933	185549	172263
NGA	Nigeria	22667	Gamjuwa	0.20	0.28	0.32	214059	184794	171562
NGA	Nigeria	22668	Gombe	0.13	0.13	0.13	424228	424228	424228
NGA	Nigeria	22669	Itas/gad	0.20	0.28	0.32	135864	117289	108891
NGA	Nigeria	22670	Jama'are	0.20	0.28	0.32	100123	86435	80246
NGA	Nigeria	22671	Kaltungo	0.13	0.13	0.13	251620	251620	251620
NGA	Nigeria	22672	Katagum	0.20	0.28	0.32	233560	201629	187192
NGA	Nigeria	22673	Misau	0.20	0.28	0.32	309644	267312	248171
NGA	Nigeria	22674	Nafada/b	0.13	0.13	0.13	357150	357150	357150
NGA	Nigeria	22675	Ningi	0.20	0.28	0.32	346946	299514	278068
NGA	Nigeria	22676	Shira	0.20	0.28	0.32	306235	264368	245438
NGA	Nigeria	22677	Tafawa-b	0.20	0.28	0.32	185889	160475	148984
NGA	Nigeria	22678	Toro	0.20	0.28	0.32	285065	246093	228472
NGA	Nigeria	22679	Yamaltu/	0.13	0.13	0.13	228703	228703	228703
NGA	Nigeria	22680	Zaki	0.20	0.28	0.32	142395	122928	114126
NGA	Nigeria	22681	Ado	0.20	0.27	0.30	162360	142524	134431

NGA	Nigeria	22682	Apa	0.20	0.27	0.30	142396	124998	117901
NGA	Nigeria	22683	Buruku	0.20	0.27	0.30	172264	151217	142631
NGA	Nigeria	22684	Gboko	0.20	0.27	0.30	378809	332528	313647
NGA	Nigeria	22685	Guma	0.20	0.27	0.30	129007	113246	106816
NGA	Nigeria	22686	Gwer	0.20	0.27	0.30	124131	108965	102778
NGA	Nigeria	22687	Gwerwest	0.20	0.27	0.30	92128	80873	76281
NGA	Nigeria	22688	Katsina (benue)	0.20	0.27	0.30	296789	260529	245735
NGA	Nigeria	22689	Konshish	0.20	0.27	0.30	201008	176450	166431
NGA	Nigeria	22690	Kwande	0.20	0.27	0.30	210909	185141	174629
NGA	Nigeria	22691	Makurdi	0.20	0.27	0.30	283901	249216	235065
NGA	Nigeria	22692	Ogbadibo	0.20	0.27	0.30	86002	75495	71208
NGA	Nigeria	22693	Oju	0.20	0.27	0.30	218698	191979	181078
NGA	Nigeria	22694	Okpokwu	0.20	0.27	0.30	127826	112209	105838
NGA	Nigeria	22695	Oturkpo	0.20	0.27	0.30	302447	265495	250420
NGA	Nigeria	22696	Ukum	0.20	0.27	0.30	151814	133266	125699
NGA	Nigeria	22697	Ushongo	0.20	0.27	0.30	131122	115102	108566
NGA	Nigeria	22698	Vandeiky	0.20	0.27	0.30	175816	154336	145572
NGA	Nigeria	22699	Askira/u	0.20	0.27	0.30	115106	101043	95306
NGA	Nigeria	22700	Bama	0.20	0.27	0.30	282346	247850	233777
NGA	Nigeria	22701	Biu	0.20	0.27	0.30	279489	245343	231412
NGA	Nigeria	22702	Damboa	0.20	0.27	0.30	250098	219542	207076
NGA	Nigeria	22703	Dikwa	0.20	0.27	0.30	75570	66338	62571
NGA	Nigeria	22704	Gwoza	0.20	0.27	0.30	167644	147162	138806
NGA	Nigeria	22705	Kaga	0.20	0.27	0.30	194781	170984	161275
NGA	Nigeria	22706	Konduga	0.20	0.27	0.30	217968	191338	180474
NGA	Nigeria	22707	Kukawa	0.20	0.27	0.30	269467	236545	223114
NGA	Nigeria	22708	Maidugur	0.20	0.27	0.30	671837	589756	556269
NGA	Nigeria	22709	Mobbar	0.20	0.27	0.30	291111	255544	241034
NGA	Nigeria	22710	Monguno	0.20	0.27	0.30	178657	156829	147924
NGA	Nigeria	22711	Ngala	0.20	0.27	0.30	241843	212296	200242
NGA	Nigeria	22712	Shani	0.20	0.27	0.30	176401	154849	146057
NGA	Nigeria	22713	Abi	0.56	0.65	0.76	78056	47730	12960
NGA	Nigeria	22714	Akamkpa	0.56	0.65	0.76	46792	28613	7769

NGA	Nigeria	22715	Akpabuyo	0.56	0.65	0.76	77869	47616	12929
NGA	Nigeria	22716	Biase	0.56	0.65	0.76	57048	34884	9472
NGA	Nigeria	22717	Boki	0.56	0.65	0.76	51122	31261	8488
NGA	Nigeria	22718	Calabar	0.56	0.65	0.76	134928	82507	22402
NGA	Nigeria	22719	Ikom	0.56	0.65	0.76	84953	51947	14105
NGA	Nigeria	22720	Obanliku	0.56	0.65	0.76	31482	19251	5227
NGA	Nigeria	22721	Obubra	0.56	0.65	0.76	48379	29583	8032
NGA	Nigeria	22722	Obudu	0.56	0.65	0.76	43495	26597	7222
NGA	Nigeria	22723	Odukpani	0.56	0.65	0.76	72777	44502	12083
NGA	Nigeria	22724	Ogoja	0.56	0.65	0.76	85978	52575	14275
NGA	Nigeria	22725	Yakurr	0.56	0.65	0.76	59128	36156	9817
NGA	Nigeria	22726	Yala Cross	0.56	0.65	0.76	50652	30973	8410
NGA	Nigeria	22727	Aniochan	0.20	0.28	0.32	79993	69084	64162
NGA	Nigeria	22728	Aniochas	0.20	0.28	0.32	105491	91105	84613
NGA	Nigeria	22729	Bomadi	0.20	0.28	0.32	88367	76316	70878
NGA	Nigeria	22730	Burutu	0.20	0.28	0.32	189562	163711	152046
NGA	Nigeria	22731	Ethiopee	0.20	0.28	0.32	86243	74482	69175
NGA	Nigeria	22732	Ethiopew	0.20	0.28	0.32	140317	121181	112547
NGA	Nigeria	22733	Ikanorth	0.20	0.28	0.32	159585	137822	128001
NGA	Nigeria	22734	Ikasouth	0.20	0.28	0.32	101831	87944	81677
NGA	Nigeria	22735	Isokonor	0.20	0.28	0.32	80970	69928	64945
NGA	Nigeria	22736	Isokosou	0.20	0.28	0.32	177713	153477	142541
NGA	Nigeria	22737	Ndokwaea	0.20	0.28	0.32	105307	90946	84466
NGA	Nigeria	22738	Ndokwawe	0.20	0.28	0.32	174260	150495	139772
NGA	Nigeria	22739	Okpe	0.20	0.28	0.32	287825	248573	230861
NGA	Nigeria	22740	Oshimill	0.20	0.28	0.32	178553	154203	143215
NGA	Nigeria	22741	Sapele	0.20	0.28	0.32	182035	157210	146008
NGA	Nigeria	22742	Ughelino	0.20	0.28	0.32	314454	271570	252220
NGA	Nigeria	22743	Ugheliso	0.20	0.28	0.32	169593	146465	136028
NGA	Nigeria	22744	Warrinorth	0.20	0.28	0.32	115609	99843	92729
NGA	Nigeria	22745	Warrisouth	0.20	0.28	0.32	419746	362503	336673
NGA	Nigeria	22746	Akoko-ed	0.20	0.27	0.30	191041	167701	158178
NGA	Nigeria	22747	Esancent	0.20	0.27	0.30	111554	97925	92365

NGA	Nigeria	22748	Esannort	0.20	0.27	0.30	102140	89661	84570
NGA	Nigeria	22749	Esansout	0.20	0.27	0.30	143493	125962	118809
NGA	Nigeria	22750	Esanwest	0.20	0.27	0.30	126424	110978	104676
NGA	Nigeria	22751	Etsakoea	0.20	0.27	0.30	167619	147140	138785
NGA	Nigeria	22752	Etsakowe	0.20	0.27	0.30	213435	187359	176720
NGA	Nigeria	22753	Oredo Edo	0.20	0.27	0.30	965075	847167	799064
NGA	Nigeria	22754	Orhionmw	0.20	0.27	0.30	260355	228546	215569
NGA	Nigeria	22755	Ovianort	0.20	0.27	0.30	118548	104065	98156
NGA	Nigeria	22756	Oviasouth-west	0.20	0.27	0.30	127020	111501	105170
NGA	Nigeria	22757	Owanwest	0.20	0.27	0.30	176289	154751	145964
NGA	Nigeria	22758	Abakalik	0.15	0.15	0.15	320903	320903	320903
NGA	Nigeria	22759	Awgu	0.15	0.15	0.15	286160	286160	286160
NGA	Nigeria	22760	Enugusou	0.15	0.15	0.15	694015	694015	694015
NGA	Nigeria	22761	Ezeagu	0.15	0.15	0.15	151239	151239	151239
NGA	Nigeria	22762	Ezza	0.15	0.15	0.15	193557	193557	193557
NGA	Nigeria	22763	Igbo-eti	0.15	0.15	0.15	189654	189654	189654
NGA	Nigeria	22764	Igbo-eze	0.15	0.15	0.15	298493	298493	298493
NGA	Nigeria	22765	Ikwo	0.15	0.15	0.15	193921	193921	193921
NGA	Nigeria	22766	Ishielu	0.15	0.15	0.15	101767	101767	101767
NGA	Nigeria	22767	Isi-uzo	0.15	0.15	0.15	279338	279338	279338
NGA	Nigeria	22768	Izzi	0.15	0.15	0.15	191656	191656	191656
NGA	Nigeria	22769	Nkanu	0.15	0.15	0.15	280928	280928	280928
NGA	Nigeria	22770	Nsukka	0.15	0.15	0.15	291508	291508	291508
NGA	Nigeria	22771	Ohaukwu	0.15	0.15	0.15	150842	150842	150842
NGA	Nigeria	22772	Oji-river	0.15	0.15	0.15	95805	95805	95805
NGA	Nigeria	22773	Udi	0.15	0.15	0.15	206096	206096	206096
NGA	Nigeria	22774	Uzo-uwani	0.15	0.15	0.15	75697	75697	75697
NGA	Nigeria	22775	Abaji	0.20	0.27	0.30	32315	28367	26757
NGA	Nigeria	22776	Abujamun	0.20	0.27	0.30	702959	617074	582036
NGA	Nigeria	22777	Gwagwala	0.20	0.27	0.30	179634	157687	148733
NGA	Nigeria	22778	Kuje	0.20	0.27	0.30	72521	63661	60046
NGA	Nigeria	22779	Aboh-mba	0.20	0.28	0.32	123252	106443	98859
NGA	Nigeria	22780	Ahizu-mb	0.20	0.28	0.32	92224	79647	73972

NGA	Nigeria	22781	Ehime-mb	0.20	0.28	0.32	107167	92552	85957
NGA	Nigeria	22782	Ezinihit	0.20	0.28	0.32	143354	123804	114983
NGA	Nigeria	22783	Ideato	0.20	0.28	0.32	122020	105380	97871
NGA	Nigeria	22784	Ideatono	0.20	0.28	0.32	156160	134864	125254
NGA	Nigeria	22785	Ihitte/u	0.20	0.28	0.32	171911	148466	137888
NGA	Nigeria	22786	Ikeduru	0.20	0.28	0.32	105398	91024	84538
NGA	Nigeria	22787	Isialamb	0.20	0.28	0.32	181586	156823	145648
NGA	Nigeria	22788	Isu	0.20	0.28	0.32	100967	87198	80985
NGA	Nigeria	22789	Mbaitoli	0.20	0.28	0.32	169228	146150	135736
NGA	Nigeria	22790	Ngor-okp	0.20	0.28	0.32	148264	128045	118921
NGA	Nigeria	22791	Nkwerre	0.20	0.28	0.32	198051	171042	158854
NGA	Nigeria	22792	Obowo	0.20	0.28	0.32	120406	103986	96577
NGA	Nigeria	22793	Oguta	0.20	0.28	0.32	58811	50790	47171
NGA	Nigeria	22794	Ohaji/eg	0.20	0.28	0.32	159873	138070	128232
NGA	Nigeria	22795	Okigwe	0.20	0.28	0.32	267101	230675	214238
NGA	Nigeria	22796	Orlu	0.20	0.28	0.32	172376	148869	138261
NGA	Nigeria	22797	Orsu	0.25	0.36	0.44	70911	57023	46726
NGA	Nigeria	22798	Oru	0.20	0.28	0.32	150733	130177	120901
NGA	Nigeria	22799	Owerri	0.20	0.28	0.32	277083	239296	222245
NGA	Nigeria	22800	Babura	0.13	0.13	0.13	203085	203085	203085
NGA	Nigeria	22801	Biriniwa	0.13	0.13	0.13	147204	147204	147204
NGA	Nigeria	22802	Birninku	0.13	0.13	0.13	522743	522743	522743
NGA	Nigeria	22803	Dutse	0.13	0.13	0.13	245360	245360	245360
NGA	Nigeria	22804	Garki	0.13	0.13	0.13	125328	125328	125328
NGA	Nigeria	22805	Gumel	0.13	0.13	0.13	187913	187913	187913
NGA	Nigeria	22806	Gwaram	0.13	0.13	0.13	201112	201112	201112
NGA	Nigeria	22807	Hadejia	0.13	0.13	0.13	256988	256988	256988
NGA	Nigeria	22808	Jahun	0.13	0.13	0.13	285031	285031	285031
NGA	Nigeria	22809	Kafinhau	0.13	0.13	0.13	180876	180876	180876
NGA	Nigeria	22810	Kazaure	0.13	0.13	0.13	242923	242923	242923
NGA	Nigeria	22811	Kirikasa	0.13	0.13	0.13	181421	181421	181421
NGA	Nigeria	22812	Kiyawa	0.13	0.13	0.13	125469	125469	125469
NGA	Nigeria	22813	Malammad	0.13	0.13	0.13	153100	153100	153100

NGA	Nigeria	22814	Ringim	0.13	0.13	0.13	303179	303179	303179
NGA	Nigeria	22815	Roni	0.13	0.13	0.13	167320	167320	167320
NGA	Nigeria	22816	Sule-tan	0.13	0.13	0.13	301253	301253	301253
NGA	Nigeria	22817	Birnin-g	0.20	0.27	0.30	205898	180742	170480
NGA	Nigeria	22818	Chikun	0.20	0.27	0.30	465564	408684	385478
NGA	Nigeria	22819	Doka/kaw	0.20	0.27	0.30	454610	399068	376408
NGA	Nigeria	22820	Giwa	0.20	0.27	0.30	208235	182794	172415
NGA	Nigeria	22821	Igabi	0.20	0.27	0.30	390770	343028	323550
NGA	Nigeria	22822	Ikara	0.20	0.27	0.30	432897	380008	358431
NGA	Nigeria	22823	Jaba	0.20	0.27	0.30	107115	94028	88689
NGA	Nigeria	22824	Jema'a	0.20	0.27	0.30	317772	278948	263109
NGA	Nigeria	22825	Kachia	0.20	0.27	0.30	417485	366479	345670
NGA	Nigeria	22826	Kaura	0.20	0.27	0.30	166217	145909	137624
NGA	Nigeria	22827	Kauru	0.20	0.27	0.30	82983	72844	68708
NGA	Nigeria	22828	Lere	0.20	0.27	0.30	276736	242925	229132
NGA	Nigeria	22829	Makarfi	0.20	0.27	0.30	259651	227928	214986
NGA	Nigeria	22830	Sabon-ga	0.20	0.27	0.30	402221	353079	333031
NGA	Nigeria	22831	Soba	0.20	0.27	0.30	193348	169725	160088
NGA	Nigeria	22832	Tudun	0.20	0.27	0.30	152028	133454	125876
NGA	Nigeria	22833	Zangonka	0.20	0.27	0.30	261385	229450	216422
NGA	Nigeria	22834	Zaria	0.20	0.27	0.30	50819	44610	42077
NGA	Nigeria	22835	Albasu	0.20	0.27	0.30	102272	89777	84679
NGA	Nigeria	22836	Bagwai	0.20	0.27	0.30	121390	106559	100509
NGA	Nigeria	22837	Bebeji	0.20	0.27	0.30	333947	293147	276502
NGA	Nigeria	22838	Bichi	0.20	0.27	0.30	243996	214186	202024
NGA	Nigeria	22839	Bunkure	0.20	0.27	0.30	139831	122747	115777
NGA	Nigeria	22840	Dala	0.20	0.27	0.30	439664	385948	364033
NGA	Nigeria	22841	Dambatta	0.20	0.27	0.30	328922	288736	272341
NGA	Nigeria	22842	Dawakink	0.20	0.27	0.30	266376	233831	220554
NGA	Nigeria	22843	Dawakint	0.20	0.27	0.30	199152	174820	164894
NGA	Nigeria	22844	Gabasawa	0.20	0.27	0.30	181869	159649	150584
NGA	Nigeria	22845	Gaya	0.20	0.27	0.30	386426	339214	319953
NGA	Nigeria	22846	Gezawa	0.20	0.27	0.30	288394	253160	238785

NGA	Nigeria	22847	Gwarzo	0.20	0.27	0.30	128733	113005	106588
NGA	Nigeria	22848	Kabo	0.20	0.27	0.30	100857	88535	83508
NGA	Nigeria	22849	Kano	0.20	0.27	0.30	210820	185063	174555
NGA	Nigeria	22850	Karaye	0.20	0.27	0.30	276207	242461	228694
NGA	Nigeria	22851	Kumbotso	0.20	0.27	0.30	141232	123977	116937
NGA	Nigeria	22852	Kura	0.20	0.27	0.30	257564	226096	213258
NGA	Nigeria	22853	Minjibir	0.20	0.27	0.30	168720	148107	139697
NGA	Nigeria	22854	Nassaraw	0.20	0.27	0.30	1210419	1062536	1002204
NGA	Nigeria	22855	Rano	0.20	0.27	0.30	214122	187961	177289
NGA	Nigeria	22856	Rimingad	0.20	0.27	0.30	204452	179473	169283
NGA	Nigeria	22857	Shanono	0.20	0.27	0.30	127065	111541	105207
NGA	Nigeria	22858	Sumaila	0.20	0.27	0.30	170818	149948	141434
NGA	Nigeria	22859	Takai	0.20	0.27	0.30	194822	171020	161309
NGA	Nigeria	22860	Tsanyawa	0.20	0.27	0.30	179233	157336	148402
NGA	Nigeria	22861	Tudunwad	0.20	0.27	0.30	290713	255195	240705
NGA	Nigeria	22862	Ungogo	0.20	0.27	0.30	608184	533879	503565
NGA	Nigeria	22863	Wudil	0.20	0.27	0.30	273797	240346	226699
NGA	Nigeria	22864	Bakori	0.20	0.27	0.30	242103	212524	200457
NGA	Nigeria	22865	Batsari	0.20	0.27	0.30	118156	103720	97831
NGA	Nigeria	22866	Bindawa	0.20	0.27	0.30	122427	107470	101368
NGA	Nigeria	22867	Daura	0.20	0.27	0.30	509289	447066	421682
NGA	Nigeria	22868	Dutsin-m	0.20	0.27	0.30	297932	261532	246682
NGA	Nigeria	22869	Faskari	0.20	0.27	0.30	265599	233150	219911
NGA	Nigeria	22870	Funtua	0.20	0.27	0.30	296721	260469	245679
NGA	Nigeria	22871	Ingawa	0.20	0.27	0.30	132952	116709	110082
NGA	Nigeria	22872	Jibia	0.20	0.27	0.30	133953	117587	110910
NGA	Nigeria	22873	Kaita	0.20	0.27	0.30	115003	100952	95220
NGA	Nigeria	22874	Kankara	0.20	0.27	0.30	192740	169192	159585
NGA	Nigeria	22875	Kankiya	0.20	0.27	0.30	239306	210069	198141
NGA	Nigeria	22876	Katsina (k)	0.20	0.27	0.30	409083	359103	338713
NGA	Nigeria	22877	Malumfas	0.20	0.27	0.30	354196	310922	293268
NGA	Nigeria	22878	Mani	0.20	0.27	0.30	157179	137976	130142
NGA	Nigeria	22879	Mashi	0.20	0.27	0.30	217966	191336	180472

NGA	Nigeria	22880	Musawa	0.20	0.27	0.30	167487	147024	138676
NGA	Nigeria	22881	Rimi	0.20	0.27	0.30	211205	185401	174874
NGA	Nigeria	22882	Safana	0.20	0.27	0.30	209276	183708	173277
NGA	Nigeria	22883	Zango	0.20	0.27	0.30	315441	276902	261179
NGA	Nigeria	22884	Arewa	0.13	0.13	0.13	233448	233448	233448
NGA	Nigeria	22885	Argungu	0.13	0.13	0.13	298624	298624	298624
NGA	Nigeria	22886	Bagudo	0.13	0.13	0.13	237871	237871	237871
NGA	Nigeria	22887	Birninke	0.13	0.13	0.13	239105	239105	239105
NGA	Nigeria	22888	Bunza	0.13	0.13	0.13	149905	149905	149905
NGA	Nigeria	22889	Dandi	0.13	0.13	0.13	144662	144662	144662
NGA	Nigeria	22890	Gwandu	0.13	0.13	0.13	76078	76078	76078
NGA	Nigeria	22891	Jega	0.13	0.13	0.13	220053	220053	220053
NGA	Nigeria	22892	Koko/bes	0.13	0.13	0.13	132039	132039	132039
NGA	Nigeria	22893	Maiyama	0.13	0.13	0.13	160737	160737	160737
NGA	Nigeria	22894	Ngaski	0.13	0.13	0.13	113959	113959	113959
NGA	Nigeria	22895	Sakaba	0.13	0.13	0.13	288451	288451	288451
NGA	Nigeria	22896	Suru	0.13	0.13	0.13	149560	149560	149560
NGA	Nigeria	22897	Yauri	0.13	0.13	0.13	183091	183091	183091
NGA	Nigeria	22898	Zuru	0.13	0.13	0.13	208585	208585	208585
NGA	Nigeria	22899	Adavi	0.13	0.13	0.13	129809	129809	129809
NGA	Nigeria	22900	Ajaokuta	0.13	0.13	0.13	480674	480674	480674
NGA	Nigeria	22901	Ankpa	0.13	0.13	0.13	348680	348680	348680
NGA	Nigeria	22902	Bassa	0.13	0.13	0.13	111076	111076	111076
NGA	Nigeria	22903	Dekina	0.13	0.13	0.13	230123	230123	230123
NGA	Nigeria	22904	Ijumu	0.13	0.13	0.13	88480	88480	88480
NGA	Nigeria	22905	Kabba/bu	0.13	0.13	0.13	130124	130124	130124
NGA	Nigeria	22906	Kogi	0.13	0.13	0.13	92757	92757	92757
NGA	Nigeria	22907	Kotonkar	0.13	0.13	0.13	157726	157726	157726
NGA	Nigeria	22908	Ofu	0.13	0.13	0.13	113605	113605	113605
NGA	Nigeria	22909	Okehi	0.13	0.13	0.13	220308	220308	220308
NGA	Nigeria	22910	Okene	0.13	0.13	0.13	360167	360167	360167
NGA	Nigeria	22911	Olamabor	0.13	0.13	0.13	117117	117117	117117
NGA	Nigeria	22912	Yagbaeas	0.13	0.13	0.13	145496	145496	145496

NGA	Nigeria	22913	Yagbawes	0.13	0.13	0.13	131270	131270	131270
NGA	Nigeria	22914	Asa	0.20	0.27	0.30	116057	101877	96093
NGA	Nigeria	22915	Baruten	0.20	0.27	0.30	150063	131729	124249
NGA	Nigeria	22916	Edu	0.20	0.27	0.30	239640	210362	198417
NGA	Nigeria	22917	Ifelodun	0.20	0.27	0.30	158730	139337	131426
NGA	Nigeria	22918	Ilorinwe	0.20	0.27	0.30	699640	614161	579289
NGA	Nigeria	22919	Irepodun	0.20	0.27	0.30	257530	226066	213230
NGA	Nigeria	22920	Irepodun (b)	0.13	0.13	0.13	196083	196083	196083
NGA	Nigeria	22921	Kaiama	0.20	0.27	0.30	95355	83705	78952
NGA	Nigeria	22922	Moro	0.20	0.27	0.30	86001	75493	71207
NGA	Nigeria	22923	Offa	0.20	0.27	0.30	102080	89608	84520
NGA	Nigeria	22924	Oyun	0.20	0.27	0.30	62257	54650	51547
NGA	Nigeria	22925	Agege	0.20	0.27	0.30	698463	613128	578315
NGA	Nigeria	22926	Badagary	0.20	0.27	0.30	254351	223275	210598
NGA	Nigeria	22927	Epe	0.20	0.27	0.30	154110	135281	127600
NGA	Nigeria	22928	Eti-osa	0.20	0.27	0.30	185914	163200	153934
NGA	Nigeria	22929	Ibeju/lekki	0.20	0.27	0.30	87807	77079	72703
NGA	Nigeria	22930	Ikeja	0.20	0.27	0.30	2077376	1823572	1720028
NGA	Nigeria	22931	Ikorodu	0.20	0.27	0.30	431563	378837	357326
NGA	Nigeria	22932	Lagosisland	0.20	0.27	0.30	853456	749185	706646
NGA	Nigeria	22933	Mainland	0.20	0.27	0.30	892060	783073	738609
NGA	Nigeria	22934	Mushin	0.20	0.27	0.30	914516	802784	757202
NGA	Nigeria	22935	Ojo	0.20	0.27	0.30	1912953	1679238	1583889
NGA	Nigeria	22936	Shomolu	0.20	0.27	0.30	358870	315025	297137
NGA	Nigeria	22937	Agaie	0.20	0.27	0.30	96753	84932	80110
NGA	Nigeria	22938	Agwara	0.20	0.27	0.30	38099	33444	31545
NGA	Nigeria	22939	Bida	0.20	0.27	0.30	261258	229339	216317
NGA	Nigeria	22940	Borgu	0.20	0.27	0.30	131339	115292	108746
NGA	Nigeria	22941	Bosso	0.20	0.27	0.30	283003	248427	234322
NGA	Nigeria	22942	Gbako	0.20	0.27	0.30	164219	144156	135970
NGA	Nigeria	22943	Gurara	0.20	0.27	0.30	177816	156091	147228
NGA	Nigeria	22944	Kontogur	0.20	0.27	0.30	288725	253450	239059
NGA	Nigeria	22945	Lapai	0.20	0.27	0.30	90693	79612	75092

NGA	Nigeria	22946	Lavun	0.20	0.27	0.30	251497	220770	208234
NGA	Nigeria	22947	Magama	0.20	0.27	0.30	137152	120396	113559
NGA	Nigeria	22948	Mokwa	0.20	0.27	0.30	151526	133014	125461
NGA	Nigeria	22949	Paikoro	0.20	0.27	0.30	120191	105506	99516
NGA	Nigeria	22950	Rafi	0.20	0.27	0.30	128144	112488	106100
NGA	Nigeria	22951	Rijau	0.20	0.27	0.30	116326	102114	96316
NGA	Nigeria	22952	Shiroro	0.20	0.27	0.30	277330	243447	229624
NGA	Nigeria	22953	Suleja	0.20	0.27	0.30	231689	203382	191834
NGA	Nigeria	22954	Wushishi	0.20	0.27	0.30	233685	205135	193487
NGA	Nigeria	22955	Abeokutanorth	0.20	0.27	0.30	395143	346866	327171
NGA	Nigeria	22956	Adoodo/ota	0.20	0.27	0.30	286992	251928	237624
NGA	Nigeria	22957	Egbadonorth	0.20	0.27	0.30	203870	178962	168801
NGA	Nigeria	22958	Egbadosouth	0.20	0.27	0.30	214287	188106	177426
NGA	Nigeria	22959	Ifo	0.20	0.27	0.30	366223	321479	303225
NGA	Nigeria	22960	Ijebueast	0.20	0.27	0.30	85391	74959	70702
NGA	Nigeria	22961	Ijebunorth	0.20	0.27	0.30	237154	208180	196359
NGA	Nigeria	22962	Ijebuode	0.20	0.27	0.30	281799	247371	233325
NGA	Nigeria	22963	Obafemi-owode	0.20	0.27	0.30	156291	137197	129406
NGA	Nigeria	22964	Odeda	0.20	0.27	0.30	84713	74363	70141
NGA	Nigeria	22965	Ogunwaterside	0.20	0.27	0.30	60342	52970	49962
NGA	Nigeria	22966	Shagamu	0.20	0.27	0.30	335043	294109	277410
NGA	Nigeria	22967	Ado-ekiti	0.13	0.13	0.13	296056	296056	296056
NGA	Nigeria	22968	Akokonorth	0.20	0.27	0.30	170265	149463	140977
NGA	Nigeria	22969	Akokonorthwest	0.20	0.27	0.30	144888	127186	119965
NGA	Nigeria	22970	Akokosou	0.20	0.27	0.30	230805	202607	191103
NGA	Nigeria	22971	Akure	0.20	0.27	0.30	437280	383856	362060
NGA	Nigeria	22972	Ekitieas	0.13	0.13	0.13	311481	311481	311481
NGA	Nigeria	22973	Ekitisouth-west	0.13	0.13	0.13	117722	117722	117722
NGA	Nigeria	22974	Ekitiwest	0.13	0.13	0.13	237814	237814	237814
NGA	Nigeria	22975	Emure/ise/orun	0.13	0.13	0.13	244990	244990	244990
NGA	Nigeria	22976	Idanre	0.20	0.27	0.30	94514	82966	78256
NGA	Nigeria	22977	Ido/osi	0.13	0.13	0.13	141405	141405	141405
NGA	Nigeria	22978	Ifedore	0.20	0.27	0.30	128946	113192	106765

NGA	Nigeria	22979	Ijero	0.13	0.13	0.13	226467	226467	226467
NGA	Nigeria	22980	Ikale	0.20	0.27	0.30	220442	193509	182521
NGA	Nigeria	22981	Ikere	0.13	0.13	0.13	142621	142621	142621
NGA	Nigeria	22982	Ikole	0.13	0.13	0.13	148951	148951	148951
NGA	Nigeria	22983	Ilajeeseodo	0.20	0.27	0.30	304565	267354	252174
NGA	Nigeria	22984	Ileoluji/okeigbo	0.20	0.27	0.30	148420	130287	122889
NGA	Nigeria	22985	Irele	0.20	0.27	0.30	92867	81521	76892
NGA	Nigeria	22986	Moba	0.13	0.13	0.13	137042	137042	137042
NGA	Nigeria	22987	Odigbo	0.20	0.27	0.30	180724	158644	149636
NGA	Nigeria	22988	Ondo	0.20	0.27	0.30	330144	289809	273353
NGA	Nigeria	22989	Ose	0.20	0.27	0.30	119806	105169	99197
NGA	Nigeria	22990	Owo	0.20	0.27	0.30	194293	170555	160871
NGA	Nigeria	22991	Oye	0.13	0.13	0.13	110472	110472	110472
NGA	Nigeria	22992	Atakumosa	0.13	0.13	0.13	177853	177853	177853
NGA	Nigeria	22993	Ayedaade	0.13	0.13	0.13	86744	86744	86744
NGA	Nigeria	22994	Ayedire	0.13	0.13	0.13	55080	55080	55080
NGA	Nigeria	22995	Boripe	0.13	0.13	0.13	166999	166999	166999
NGA	Nigeria	22996	Ede	0.13	0.13	0.13	92160	92160	92160
NGA	Nigeria	22997	Egbedore	0.13	0.13	0.13	79057	79057	79057
NGA	Nigeria	22998	Ejigbo	0.13	0.13	0.13	127313	127313	127313
NGA	Nigeria	22999	Ifecentral	0.13	0.13	0.13	418044	418044	418044
NGA	Nigeria	23000	Ifelodun	0.13	0.13	0.13	134978	134978	134978
NGA	Nigeria	23001	Ifenorth	0.13	0.13	0.13	95206	95206	95206
NGA	Nigeria	23002	Ifesouth	0.13	0.13	0.13	102783	102783	102783
NGA	Nigeria	23003	Ila	0.13	0.13	0.13	126925	126925	126925
NGA	Nigeria	23004	Ilesa	0.13	0.13	0.13	144254	144254	144254
NGA	Nigeria	23005	Irewole	0.13	0.13	0.13	274215	274215	274215
NGA	Nigeria	23006	Iwo	0.13	0.13	0.13	201354	201354	201354
NGA	Nigeria	23007	Obokun	0.13	0.13	0.13	113427	113427	113427
NGA	Nigeria	23008	Odo0tin	0.13	0.13	0.13	134894	134894	134894
NGA	Nigeria	23009	Ola-oluwa	0.13	0.13	0.13	63719	63719	63719
NGA	Nigeria	23010	Olorunda	0.13	0.13	0.13	131437	131437	131437
NGA	Nigeria	23011	Oriade	0.13	0.13	0.13	226406	226406	226406

NGA	Nigeria	23012	Osogbo	0.13	0.13	0.13	138767	138767	138767
NGA	Nigeria	23013	Afijio	0.20	0.27	0.30	88959	78090	73656
NGA	Nigeria	23014	Akinyele	0.20	0.27	0.30	114074	100137	94451
NGA	Nigeria	23015	Egbeda	0.20	0.27	0.30	64461	56585	53372
NGA	Nigeria	23016	Ibadannorth	0.20	0.27	0.30	769886	675825	637451
NGA	Nigeria	23017	Ibadannorth-east	0.20	0.27	0.30	666524	585091	551869
NGA	Nigeria	23018	Ibadannorth-west	0.20	0.27	0.30	31320	27493	25932
NGA	Nigeria	23019	Ibadansouth-east	0.20	0.27	0.30	179921	157939	148971
NGA	Nigeria	23020	Ibadansouth-west	0.20	0.27	0.30	115398	101299	95547
NGA	Nigeria	23021	Ibarapa	0.20	0.27	0.30	84439	74123	69914
NGA	Nigeria	23022	Ido	0.20	0.27	0.30	42336	37164	35054
NGA	Nigeria	23023	Ifedapo	0.20	0.27	0.30	395661	347321	327600
NGA	Nigeria	23024	Ifeloju	0.20	0.27	0.30	167021	146615	138290
NGA	Nigeria	23025	Irepo	0.20	0.27	0.30	162384	142544	134451
NGA	Nigeria	23026	Iseyin	0.20	0.27	0.30	300343	263648	248678
NGA	Nigeria	23027	Kajola	0.20	0.27	0.30	230662	202481	190984
NGA	Nigeria	23028	Lagelu	0.20	0.27	0.30	90686	79606	75086
NGA	Nigeria	23029	Ogbomoso	0.20	0.27	0.30	277557	243646	229812
NGA	Nigeria	23030	Ogo-oluw	0.20	0.27	0.30	51896	45556	42969
NGA	Nigeria	23031	Oluyole	0.20	0.27	0.30	71500	62764	59201
NGA	Nigeria	23032	Ona-ara	0.20	0.27	0.30	123659	108551	102387
NGA	Nigeria	23033	Orelope	0.20	0.27	0.30	79052	69394	65454
NGA	Nigeria	23034	Orire	0.20	0.27	0.30	106308	93320	88021
NGA	Nigeria	23035	Oyo	0.20	0.27	0.30	362317	318051	299992
NGA	Nigeria	23036	Surulere	0.20	0.27	0.30	94000	82516	77830
NGA	Nigeria	23037	Akwanga	0.54	0.57	0.62	47938	41825	32353
NGA	Nigeria	23038	Awe	0.54	0.57	0.62	62078	54161	41896
NGA	Nigeria	23039	Barinkil	0.20	0.28	0.32	222668	192226	178462
NGA	Nigeria	23040	Bassa	0.20	0.28	0.32	133928	115619	107340
NGA	Nigeria	23041	Doma	0.54	0.57	0.62	46959	40971	31692
NGA	Nigeria	23042	Josnorth	0.20	0.28	0.32	718496	620268	575854
NGA	Nigeria	23043	Kanam	0.20	0.28	0.32	137610	118797	110290
NGA	Nigeria	23044	Keffi	0.54	0.57	0.62	144949	126464	97825

NGA	Nigeria	23045	Lafia	0.54	0.57	0.62	170897	149104	115337
NGA	Nigeria	23046	Langtang	0.20	0.28	0.32	116241	100349	93164
NGA	Nigeria	23047	Langtang (b)	0.20	0.28	0.32	81055	69974	64963
NGA	Nigeria	23048	Mangu	0.20	0.28	0.32	361592	312158	289806
NGA	Nigeria	23049	Nasarawa	0.54	0.57	0.62	146525	127839	98888
NGA	Nigeria	23050	Pankshin	0.20	0.28	0.32	235335	203161	188614
NGA	Nigeria	23051	Qua'anpa	0.20	0.28	0.32	146851	126774	117697
NGA	Nigeria	23052	Shendam	0.20	0.28	0.32	230473	198964	184717
NGA	Nigeria	23053	Wase	0.20	0.28	0.32	116594	100654	93447
NGA	Nigeria	23054	Abua/odu	0.20	0.28	0.32	218202	188445	175017
NGA	Nigeria	23055	Ahoada	0.20	0.28	0.32	289185	249748	231952
NGA	Nigeria	23056	Akukutor	0.20	0.28	0.32	106453	91936	85385
NGA	Nigeria	23057	Andoni/o	0.20	0.28	0.32	123542	106694	99092
NGA	Nigeria	23058	Asari-to	0.20	0.28	0.32	60160	51955	48253
NGA	Nigeria	23059	Bonny	0.20	0.28	0.32	148502	128250	119112
NGA	Nigeria	23060	Brass	0.13	0.13	0.13	385967	385967	385967
NGA	Nigeria	23061	Degema	0.20	0.28	0.32	241442	208515	193658
NGA	Nigeria	23062	Ekeremor	0.13	0.13	0.13	174662	174662	174662
NGA	Nigeria	23063	Emuoha	0.20	0.28	0.32	148899	128593	119430
NGA	Nigeria	23064	Etche	0.20	0.28	0.32	321630	277768	257975
NGA	Nigeria	23065	Gokana	0.20	0.28	0.32	81012	69964	64979
NGA	Nigeria	23066	Ikwerre	0.20	0.28	0.32	212154	183222	170166
NGA	Nigeria	23067	Khana	0.20	0.28	0.32	522403	451160	419013
NGA	Nigeria	23068	Northern	0.13	0.13	0.13	358632	358632	358632
NGA	Nigeria	23069	Obio/akp	0.20	0.28	0.32	636896	550039	510847
NGA	Nigeria	23070	Ogba/egbe	0.20	0.28	0.32	203561	175800	163274
NGA	Nigeria	23071	Okrika	0.20	0.28	0.32	164513	142078	131954
NGA	Nigeria	23072	Portharcourt	0.20	0.28	0.32	144693	124960	116056
NGA	Nigeria	23073	Sagbama	0.13	0.13	0.13	136026	136026	136026
NGA	Nigeria	23074	Southernijaw	0.13	0.13	0.13	288400	288400	288400
NGA	Nigeria	23075	Tai/ekem	0.20	0.28	0.32	408541	352826	327686
NGA	Nigeria	23076	Anka	0.13	0.13	0.13	368454	368454	368454
NGA	Nigeria	23077	Bakura	0.13	0.13	0.13	185782	185782	185782

NGA	Nigeria	23078	Binji	0.20	0.27	0.30	83816	73575	69398
NGA	Nigeria	23079	Bodinga	0.20	0.27	0.30	111800	98141	92568
NGA	Nigeria	23080	Bungudu	0.13	0.13	0.13	392930	392930	392930
NGA	Nigeria	23081	Dange-sh	0.20	0.27	0.30	100987	88649	83615
NGA	Nigeria	23082	Gada	0.20	0.27	0.30	220018	193137	182170
NGA	Nigeria	23083	Goronyo	0.20	0.27	0.30	94389	82857	78152
NGA	Nigeria	23084	Gummi	0.13	0.13	0.13	471300	471300	471300
NGA	Nigeria	23085	Gusau	0.13	0.13	0.13	254698	254698	254698
NGA	Nigeria	23086	Gwadabaw	0.20	0.27	0.30	254461	223372	210689
NGA	Nigeria	23087	Illela	0.20	0.27	0.30	156375	137270	129475
NGA	Nigeria	23088	Isa	0.13	0.13	0.13	292183	292183	292183
NGA	Nigeria	23089	Kaura-na	0.13	0.13	0.13	370764	370764	370764
NGA	Nigeria	23090	Kware	0.20	0.27	0.30	192942	169369	159752
NGA	Nigeria	23091	Maradun	0.13	0.13	0.13	149364	149364	149364
NGA	Nigeria	23092	Rabah	0.20	0.27	0.30	124218	109041	102850
NGA	Nigeria	23093	Sabonbir	0.20	0.27	0.30	161641	141892	133836
NGA	Nigeria	23094	Silame	0.20	0.27	0.30	49312	43288	40830
NGA	Nigeria	23095	Sokoto	0.20	0.27	0.30	348472	305897	288528
NGA	Nigeria	23096	Talatar	0.13	0.13	0.13	177241	177241	177241
NGA	Nigeria	23097	Tambawal	0.20	0.27	0.30	258681	227076	214183
NGA	Nigeria	23098	Tangazar	0.20	0.27	0.30	182578	160272	151171
NGA	Nigeria	23099	Tsafe	0.13	0.13	0.13	166359	166359	166359
NGA	Nigeria	23100	Wamakko	0.20	0.27	0.30	240666	211262	199267
NGA	Nigeria	23101	Wurno	0.20	0.27	0.30	47537	41729	39359
NGA	Nigeria	23102	Yabo	0.20	0.27	0.30	181384	159224	150183
NGA	Nigeria	23103	Zurmi	0.13	0.13	0.13	240812	240812	240812
NGA	Nigeria	23104	Bali	0.20	0.27	0.30	333884	293092	276450
NGA	Nigeria	23105	Donga	0.20	0.27	0.30	95155	83530	78787
NGA	Nigeria	23106	Gashaka	0.20	0.27	0.30	69056	60619	57177
NGA	Nigeria	23107	Ibi	0.20	0.27	0.30	47288	41510	39153
NGA	Nigeria	23108	Jalingo	0.20	0.27	0.30	137563	120756	113899
NGA	Nigeria	23109	Karim-la	0.20	0.27	0.30	130497	114553	108049
NGA	Nigeria	23110	Lau	0.20	0.27	0.30	40682	35711	33684

NGA	Nigeria	23111	Sardauna	0.20	0.27	0.30	235867	207050	195293
NGA	Nigeria	23112	Takum	0.20	0.27	0.30	137546	120742	113886
NGA	Nigeria	23113	Wukari	0.20	0.27	0.30	201008	176450	166431
NGA	Nigeria	23114	Yorro	0.20	0.27	0.30	164428	144339	136144
NGA	Nigeria	23115	Zing	0.20	0.27	0.30	95521	83851	79090
NGA	Nigeria	23116	Bade	0.13	0.13	0.13	412764	412764	412764
NGA	Nigeria	23117	Damaturu	0.13	0.13	0.13	229273	229273	229273
NGA	Nigeria	23118	Fika	0.13	0.13	0.13	177115	177115	177115
NGA	Nigeria	23119	Fune	0.13	0.13	0.13	257392	257392	257392
NGA	Nigeria	23120	Geidam	0.13	0.13	0.13	135349	135349	135349
NGA	Nigeria	23121	Gujba	0.13	0.13	0.13	168499	168499	168499
NGA	Nigeria	23122	Nangere	0.13	0.13	0.13	294709	294709	294709
NGA	Nigeria	23123	Nguru	0.13	0.13	0.13	221141	221141	221141
NGA	Nigeria	23124	Yunusari	0.13	0.13	0.13	100615	100615	100615
NGA	Nigeria	23125	Yusufari	0.13	0.13	0.13	83990	83990	83990
PAN	Panama	23719	Colon	0.16	0.16	0.16	88361	88361	88361
PAN	Panama	23721	Portobelo	0.16	0.16	0.16	24418	24418	24418
PAN	Panama	23722	Santa Isabel	0.16	0.16	0.16	6679	6679	6679
PAN	Panama	23723	Comarca De San Blas	0.16	0.16	0.16	21507	21507	21507
PAN	Panama	23724	Cemaco	0.16	0.16	0.16	5956	5956	5956
PAN	Panama	23725	Chepigana	0.16	0.16	0.16	22334	22334	22334
PAN	Panama	23726	Pinogana	0.16	0.16	0.16	13469	13469	13469
PAN	Panama	23727	Sambu	0.00	0.00	0.00	2707	2707	2707
PAN	Panama	23746	Chepo	0.16	0.16	0.16	37480	37480	37480
PAN	Panama	23747	Chiman	0.16	0.16	0.16	2634	2634	2634
PAN	Panama	23749	Panama	0.16	0.16	0.16	770227	770227	770227
PER	Peru	1011922	CARAVELI	0.15	0.15	0.15	29156	29156	29156
PER	Peru	1011848	SAN PABLO	0.15	0.15	0.15	16107	16107	16107
PER	Peru	1011849	LIMA	0.15	0.15	0.15	5878766	5878766	5878766
PER	Peru	1011850	OXAPAMPA	0.51	0.77	0.92	28519	2634	0
PER	Peru	1011851	CALLAO	0.15	0.15	0.15	470454	470454	470454
PER	Peru	1011854	LA UNION	0.15	0.15	0.15	9560	9560	9560
PER	Peru	1011855	CASTILLA	0.15	0.15	0.15	31649	31649	31649

PER	Peru	1011857	SANTIAGO DE CHUCO	0.15	0.15	0.15	40309	40309	40309
PER	Peru	1011860	CANAS	0.15	0.15	0.15	24368	24368	24368
PER	Peru	1011863	MANU	0.32	0.68	0.88	14209	3642	0
PER	Peru	1011864	ANDAHUAYLAS	0.15	0.15	0.15	100203	100203	100203
PER	Peru	1011866	MARISCAL LUZURIAGA	0.15	0.15	0.15	14987	14987	14987
PER	Peru	1011867	VIRU	0.15	0.15	0.15	78279	78279	78279
PER	Peru	1011868	CANTA	0.15	0.15	0.15	13813	13813	13813
PER	Peru	1011869	TOCACHE	0.32	0.68	0.88	35197	9022	0
PER	Peru	1011872	LA CONVENCION	0.32	0.68	0.88	83410	21380	0
PER	Peru	1011873	TAYACAJA	0.32	0.68	0.88	53977	13835	0
PER	Peru	1011874	HUANCAYO	0.15	0.15	0.15	334116	334116	334116
PER	Peru	1011878	VICTOR FAJARDO	0.15	0.15	0.15	16220	16220	16220
PER	Peru	1011880	PUERTO INCA	0.32	0.68	0.88	14797	3793	0
PER	Peru	1011881	CHINCHA	0.15	0.15	0.15	144815	144815	144815
PER	Peru	1011883	YAROWILCA	0.15	0.15	0.15	21065	21065	21065
PER	Peru	1011886	SANDIA	0.32	0.68	0.88	32530	8338	0
PER	Peru	1011887	PISCO	0.15	0.15	0.15	91739	91739	91739
PER	Peru	1011888	SAN ANTONIO DE PUTIN	0.15	0.15	0.15	45890	45890	45890
PER	Peru	1011892	ESPINAR	0.15	0.15	0.15	42664	42664	42664
PER	Peru	1011893	HUAURA	0.15	0.15	0.15	143013	143013	143013
PER	Peru	1011897	MAYNAS	0.33	0.69	0.88	426970	98284	0
PER	Peru	1011898	HUANTA	0.32	0.68	0.88	55877	14322	0
PER	Peru	1011900	GRAN CHIMU	0.15	0.15	0.15	20178	20178	20178
PER	Peru	1011901	AZANGARO	0.15	0.15	0.15	87902	87902	87902
PER	Peru	1011920	SAN MARCOS	0.15	0.15	0.15	34080	34080	34080
PER	Peru	1011924	SIHUAS	0.15	0.15	0.15	19339	19339	19339
PER	Peru	1011925	CELENDIN	0.15	0.15	0.15	59896	59896	59896
PER	Peru	1011926	TARMA	0.51	0.77	0.92	31579	2916	0
PER	Peru	1011927	CHURCAMPA	0.32	0.68	0.88	22221	5696	0
PER	Peru	1011930	CAJATAMBO	0.15	0.15	0.15	5268	5268	5268
PER	Peru	1011933	CHACHAPOYAS	0.32	0.68	0.88	25310	6487	0
PER	Peru	1011935	TAHUAMANU	0.32	0.68	0.88	6891	1766	0
PER	Peru	1011936	MOYOBAMBA	0.32	0.68	0.88	72732	18643	0

PER	Peru	1011938	CAJABAMBA	0.15	0.15	0.15	49714	49714	49714
PER	Peru	1011940	YAUYOS	0.15	0.15	0.15	18643	18643	18643
PER	Peru	1011943	RECUAY	0.15	0.15	0.15	12663	12663	12663
PER	Peru	1011945	PACASMAYO	0.15	0.15	0.15	65276	65276	65276
PER	Peru	1011961	TACNA	0.15	0.15	0.15	206800	206800	206800
PER	Peru	1011962	CARLOS F. FITZCARRAL	0.15	0.15	0.15	13752	13752	13752
PER	Peru	1011965	ANTA	0.15	0.15	0.15	35853	35853	35853
PER	Peru	1011966	EL COLLAO	0.15	0.15	0.15	54080	54080	54080
PER	Peru	1011968	JULCAN	0.15	0.15	0.15	20413	20413	20413
PER	Peru	1011969	BARRANCA	0.15	0.15	0.15	94141	94141	94141
PER	Peru	1011970	FERREÂ¥AFE	0.32	0.68	0.88	49627	12720	0
PER	Peru	1011971	AIJA	0.15	0.15	0.15	5174	5174	5174
PER	Peru	1011977	EL DORADO	0.32	0.68	0.88	21374	5478	0
PER	Peru	1011978	CAMANA	0.15	0.15	0.15	44773	44773	44773
PER	Peru	1011979	ISLAY	0.15	0.15	0.15	35154	35154	35154
PER	Peru	1011980	MORROPON	0.32	0.68	0.88	74668	19139	0
PER	Peru	1011981	SATIPO	0.51	0.77	0.92	87266	8059	0
PER	Peru	1011985	SULLANA	0.32	0.68	0.88	154486	39598	0
PER	Peru	1011986	POMABAMBA	0.15	0.15	0.15	18588	18588	18588
PER	Peru	1011988	TRUJILLO	0.15	0.15	0.15	630614	630614	630614
PER	Peru	1011990	ANTONIO RAYMONDI	0.15	0.15	0.15	10253	10253	10253
PER	Peru	1011991	CUTERVO	0.32	0.68	0.88	63947	16391	0
PER	Peru	1012011	GENERAL SANCHEZ CERR	0.15	0.15	0.15	19358	19358	19358
PER	Peru	1012012	CORONEL PORTILLO	0.32	0.68	0.88	194589	49877	0
PER	Peru	1012013	MARISCAL RAMON CASTI	0.32	0.68	0.88	34178	8760	0
PER	Peru	1012014	CHUCUITO	0.15	0.15	0.15	104949	104949	104949
PER	Peru	1012015	MARISCAL NIETO	0.15	0.15	0.15	53690	53690	53690
PER	Peru	1012016	OCROS	0.15	0.15	0.15	8598	8598	8598
PER	Peru	1012018	HUARMEY	0.15	0.15	0.15	19914	19914	19914
PER	Peru	1012019	SAN ROMAN	0.15	0.15	0.15	186461	186461	186461
PER	Peru	1012021	CARHUAZ	0.15	0.15	0.15	29993	29993	29993
PER	Peru	1012022	AMBO	0.15	0.15	0.15	37128	37128	37128
PER	Peru	1012023	PALLASCA	0.15	0.15	0.15	19699	19699	19699

PER	Peru	1012025	PAITA	0.32	0.68	0.88	63893	16377	0
PER	Peru	1012038	REQUENA	0.33	0.69	0.88	34091	7847	0
PER	Peru	1012039	HUARI	0.15	0.15	0.15	40434	40434	40434
PER	Peru	1012040	ATALAYA	0.32	0.68	0.88	27015	6924	0
PER	Peru	1012041	UTCUBAMBA	0.32	0.68	0.88	53537	13723	0
PER	Peru	1012043	ANTABAMBA	0.15	0.15	0.15	8040	8040	8040
PER	Peru	1012044	CHUMBIVILCAS	0.15	0.15	0.15	50937	50937	50937
PER	Peru	1012045	HUAROCHIRI	0.15	0.15	0.15	78721	78721	78721
PER	Peru	1012047	MARISCAL CACERES	0.32	0.68	0.88	24652	6319	0
PER	Peru	1012049	MELGAR	0.15	0.15	0.15	49671	49671	49671
PER	Peru	1012051	SANTA	0.15	0.15	0.15	275248	275248	275248
PER	Peru	1012052	SAN IGNACIO	0.32	0.68	0.88	67531	17310	0
PER	Peru	1012053	CONTUMAZA	0.15	0.15	0.15	20707	20707	20707
PER	Peru	1012055	PASCO	0.15	0.15	0.15	109250	109250	109250
PER	Peru	1012057	BAGUA	0.32	0.68	0.88	34578	8863	0
PER	Peru	1012058	PACHITEA	0.32	0.68	0.88	33365	8552	0
PER	Peru	1011823	VILCAS HUAMAN	0.15	0.15	0.15	15978	15978	15978
PER	Peru	1011824	QUISPICANCHI	0.32	0.68	0.88	42144	10802	0
PER	Peru	1011826	HUAMANGA	0.15	0.15	0.15	176626	176626	176626
PER	Peru	1011827	ILO	0.15	0.15	0.15	46285	46285	46285
PER	Peru	1011841	HUANCA SANCOS	0.15	0.15	0.15	7023	7023	7023
PER	Peru	1011844	CANDARAVE	0.15	0.15	0.15	5106	5106	5106
PER	Peru	1011862	PALPA	0.15	0.15	0.15	8069	8069	8069
PER	Peru	1011877	HUANCAVELICA	0.15	0.15	0.15	104796	104796	104796
PER	Peru	1011894	SANTA CRUZ	0.32	0.68	0.88	20960	5372	0
PER	Peru	1011905	CHANCHAMAYO	0.32	0.68	0.88	103810	26609	0
PER	Peru	1011948	CARABAYA	0.32	0.68	0.88	48822	12514	0
PER	Peru	1011952	BONGARA	0.32	0.68	0.88	15715	4028	0
PER	Peru	1011958	LA MAR	0.32	0.68	0.88	45061	11550	0
PER	Peru	1011976	TARATA	0.15	0.15	0.15	5309	5309	5309
PER	Peru	1011982	SAN MARTIN	0.32	0.68	0.88	94121	24125	0
PER	Peru	1011983	CORONGO	0.15	0.15	0.15	5355	5355	5355
PER	Peru	1011984	RIOJA	0.32	0.68	0.88	63709	16330	0

PER	Peru	1012000	URUBAMBA	0.15	0.15	0.15	40906	40906	40906
PER	Peru	1012028	MOHO	0.15	0.15	0.15	14014	14014	14014
PER	Peru	1011833	HUACAYBAMBA	0.32	0.68	0.88	10555	2705	0
PER	Peru	1011836	CAJAMARCA	0.15	0.15	0.15	246031	246031	246031
PER	Peru	1011871	LAMPA	0.15	0.15	0.15	38880	38880	38880
PER	Peru	1011876	JUNIN	0.32	0.68	0.88	12430	3186	0
PER	Peru	1011879	CHEPEN	0.15	0.15	0.15	56804	56804	56804
PER	Peru	1011891	GRAU	0.15	0.15	0.15	15752	15752	15752
PER	Peru	1011904	TUMBES	0.32	0.68	0.88	73812	18919	0
PER	Peru	1011913	JAUJA	0.15	0.15	0.15	56215	56215	56215
PER	Peru	1011921	SUCRE	0.15	0.15	0.15	8247	8247	8247
PER	Peru	1011923	YUNGUYO	0.15	0.15	0.15	29679	29679	29679
PER	Peru	1011931	PAUCARTAMBO	0.32	0.68	0.88	23461	6013	0
PER	Peru	1011820	LUYA	0.32	0.68	0.88	23969	6144	0
PER	Peru	1011834	PADRE ABAD	0.32	0.68	0.88	30120	7720	0
PER	Peru	1011843	HUANCABAMBA	0.32	0.68	0.88	60612	15536	0
PER	Peru	1011852	LAMAS	0.32	0.68	0.88	42773	10964	0
PER	Peru	1011853	CHOTA	0.32	0.68	0.88	75650	19391	0
PER	Peru	1011865	DOS DE MAYO	0.15	0.15	0.15	35476	35476	35476
PER	Peru	1011912	AYMARAES	0.15	0.15	0.15	19745	19745	19745
PER	Peru	1011914	CONTRALMIRANTE VILLA	0.32	0.68	0.88	9620	2466	0
PER	Peru	1011932	ACOMAYO	0.15	0.15	0.15	17190	17190	17190
PER	Peru	1011942	RODRIGUEZ DE MENDO	0.32	0.68	0.88	16184	4148	0
PER	Peru	1011944	UCAYALI	0.32	0.68	0.88	36431	9338	0
PER	Peru	1011974	TAMBOPATA	0.32	0.68	0.88	48868	12526	0
PER	Peru	1012017	AYABACA	0.32	0.68	0.88	68762	17625	0
PER	Peru	1012036	PUNO	0.15	0.15	0.15	157588	157588	157588
PER	Peru	1012037	CAYLLOMA	0.15	0.15	0.15	73844	73844	73844
PER	Peru	1011801	SANCHEZ CARRION	0.15	0.15	0.15	99855	99855	99855
PER	Peru	1011804	PIURA	0.32	0.68	0.88	368655	94494	0
PER	Peru	1011809	HUARAZ	0.15	0.15	0.15	106116	106116	106116
PER	Peru	1011847	HUAYTARA	0.15	0.15	0.15	15822	15822	15822
PER	Peru	1011856	ABANCAY	0.15	0.15	0.15	62352	62352	62352

PER	Peru	1011957	HUAMALIES	0.32	0.68	0.88	38131	9774	0
PER	Peru	1011964	PURUS	0.32	0.68	0.88	2246	576	0
PER	Peru	1011967	COTABAMBAS	0.15	0.15	0.15	30811	30811	30811
PER	Peru	1011996	LAMBAYEQUE	0.32	0.68	0.88	141928	36379	0
PER	Peru	1011998	ANGARAES	0.15	0.15	0.15	45967	45967	45967
PER	Peru	1011802	YUNGAY	0.15	0.15	0.15	37502	37502	37502
PER	Peru	1011805	ACOBAMBA	0.15	0.15	0.15	54946	54946	54946
PER	Peru	1011806	CALCA	0.32	0.68	0.88	35778	9171	0
PER	Peru	1011811	CUSCO	0.15	0.15	0.15	288578	288578	288578
PER	Peru	1011812	CAÂ¥ETE	0.15	0.15	0.15	152620	152620	152620
PER	Peru	1011813	YAULI	0.15	0.15	0.15	28331	28331	28331
PER	Peru	1011816	CANGALLO	0.15	0.15	0.15	22645	22645	22645
PER	Peru	1011819	ICA	0.15	0.15	0.15	240972	240972	240972
PER	Peru	1011821	OYON	0.15	0.15	0.15	14673	14673	14673
PER	Peru	1011822	CONDESUYOS	0.15	0.15	0.15	12530	12530	12530
PER	Peru	1011825	HUALLAGA	0.32	0.68	0.88	12909	3309	0
PER	Peru	1011828	CONCEPCION	0.15	0.15	0.15	37425	37425	37425
PER	Peru	1011830	CASTROVIRREYNA	0.15	0.15	0.15	12901	12901	12901
PER	Peru	1011831	JAEN	0.32	0.68	0.88	91769	23522	0
PER	Peru	1011835	SECHURA	0.32	0.68	0.88	37051	9497	0
PER	Peru	1011837	JORGE BASADRE	0.15	0.15	0.15	6438	6438	6438
PER	Peru	1011838	PARINACOCHAS	0.15	0.15	0.15	23533	23533	23533
PER	Peru	1011839	LEONCIO PRADO	0.32	0.68	0.88	60663	15549	0
PER	Peru	1011840	BOLOGNESI	0.15	0.15	0.15	23053	23053	23053
PER	Peru	1011845	AREQUIPA	0.15	0.15	0.15	652112	652112	652112
PER	Peru	1011902	CHINCHEROS	0.32	0.68	0.88	25333	6493	0
PER	Peru	1011903	LORETO	0.32	0.68	0.88	34902	8946	0
PER	Peru	1011906	HUARAL	0.15	0.15	0.15	124038	124038	124038
PER	Peru	1011910	MARAÂ¥ON	0.32	0.68	0.88	15776	4044	0
PER	Peru	1011911	OTUZCO	0.15	0.15	0.15	58802	58802	58802
PER	Peru	1011917	TALARA	0.32	0.68	0.88	63208	16201	0
PER	Peru	1011918	ZARUMILLA	0.32	0.68	0.88	26093	6688	0
PER	Peru	1011919	HUAYLAS	0.15	0.15	0.15	36393	36393	36393

PER	Peru	1011946	HUANCANE	0.15	0.15	0.15	41471	41471	41471
PER	Peru	1011950	CHUPACA	0.15	0.15	0.15	35855	35855	35855
PER	Peru	1011951	DANIEL ALCIDES CARRI	0.15	0.15	0.15	38148	38148	38148
PER	Peru	1011953	CANCHIS	0.15	0.15	0.15	62924	62924	62924
PER	Peru	1011954	PARURO	0.15	0.15	0.15	20887	20887	20887
PER	Peru	1011956	LAURICOCHA	0.15	0.15	0.15	23254	23254	23254
PER	Peru	1011959	BOLIVAR	0.32	0.68	0.88	7923	2031	0
PER	Peru	1011992	ALTO AMAZONAS	0.32	0.68	0.88	90263	23136	0
PER	Peru	1011993	ASUNCION	0.15	0.15	0.15	5795	5795	5795
PER	Peru	1011997	SAN MIGUEL	0.15	0.15	0.15	35004	35004	35004
PER	Peru	1011999	PICOTA	0.32	0.68	0.88	22067	5656	0
PER	Peru	1012003	NAZCA	0.15	0.15	0.15	39035	39035	39035
PER	Peru	1012005	CASMA	0.15	0.15	0.15	30180	30180	30180
PER	Peru	1012006	PAUCAR DEL SARA SARA	0.15	0.15	0.15	7534	7534	7534
PER	Peru	1012008	ASCOPE	0.15	0.15	0.15	77762	77762	77762
PER	Peru	1012027	CHICLAYO	0.32	0.68	0.88	395021	101252	0
PER	Peru	1012029	CONDORCANQUI	0.32	0.68	0.88	24890	6380	0
PER	Peru	1012030	HUANUCO	0.32	0.68	0.88	141301	36218	0
PER	Peru	1012031	PATAZ	0.32	0.68	0.88	42655	10933	0
PER	Peru	1012032	LUCANAS	0.15	0.15	0.15	48073	48073	48073
PER	Peru	1012033	HUALGAYOC	0.15	0.15	0.15	63027	63027	63027
PER	Peru	1012034	BELLAVISTA	0.33	0.69	0.88	30936	7121	0
PER	Peru	68764	Admin unit not available	0.15	0.15	0.15	673	673	673
PER	Peru	24177	Admin unit not available	0.15	0.15	0.15	2446	2446	2446
PRY	Paraguay	23785	Fuerte Olimpo	0.73	0.73	0.74	163	152	128
PRY	Paraguay	23786	La Victoria	0.73	0.73	0.74	450	418	353
PRY	Paraguay	23787	Mayor Pablo La Gerenza	0.73	0.73	0.74	320	298	251
PRY	Paraguay	23788	Ciudad Del Este	0.73	0.73	0.74	20862	19406	16366
PRY	Paraguay	23789	D. M. Irala	0.73	0.73	0.74	908	845	712
PRY	Paraguay	23790	Hernandarias	0.73	0.73	0.74	7454	6933	5847
PRY	Paraguay	23791	Itakyry	0.73	0.73	0.74	2600	2419	2040
PRY	Paraguay	23792	J.e. Oleary	0.73	0.73	0.74	1745	1624	1369
PRY	Paraguay	23793	Los Cedrales	0.73	0.73	0.74	1481	1378	1162

PRY	Paraguay	23794	Mallorquin	0.73	0.73	0.74	1795	1670	1409
PRY	Paraguay	23795	Mbaracayu	0.73	0.73	0.74	1248	1161	979
PRY	Paraguay	23796	Minga Guazu	0.73	0.73	0.74	5110	4754	4009
PRY	Paraguay	23797	Minga Pora	0.73	0.73	0.74	1350	1256	1059
PRY	Paraguay	23798	Nacunday	0.73	0.73	0.74	1532	1425	1201
PRY	Paraguay	23799	Naranjal	0.73	0.73	0.74	1109	1031	870
PRY	Paraguay	23800	Pte. Franco	0.73	0.73	0.74	5133	4775	4027
PRY	Paraguay	23801	San Alberto	0.73	0.73	0.74	1429	1330	1121
PRY	Paraguay	23802	San Cristobal	0.73	0.73	0.74	919	855	721
PRY	Paraguay	23803	Santa Rita	0.73	0.73	0.74	1499	1394	1176
PRY	Paraguay	23804	Santa Rosa	0.73	0.73	0.74	1440	1339	1129
PRY	Paraguay	23805	Yguazu	0.73	0.73	0.74	1156	1075	907
PRY	Paraguay	23806	Bella Vista	0.73	0.73	0.74	802	746	629
PRY	Paraguay	23807	Capitan Bado	0.73	0.73	0.74	1381	1284	1083
PRY	Paraguay	23808	Pedro Juan Caballero	0.73	0.73	0.74	6770	6298	5311
PRY	Paraguay	23809	Gral. Eugenio A. Garay	0.73	0.73	0.74	2189	2036	1717
PRY	Paraguay	23810	Mcal. Estigarribia	0.73	0.73	0.74	665	618	521
PRY	Paraguay	23811	Pedro P. Pena	0.73	0.73	0.74	1685	1568	1322
PRY	Paraguay	23812	3 De Febrero	0.73	0.73	0.74	637	593	500
PRY	Paraguay	23813	Caaguazu	0.73	0.73	0.74	7269	6761	5702
PRY	Paraguay	23814	Carayao	0.73	0.73	0.74	1119	1041	878
PRY	Paraguay	23815	Cecilio Baez	0.73	0.73	0.74	447	416	351
PRY	Paraguay	23816	Coronel Oviedo	0.73	0.73	0.74	5983	5566	4694
PRY	Paraguay	23817	J.e. Estigarribia	0.73	0.73	0.74	1764	1641	1384
PRY	Paraguay	23818	Jose D. Ocampos	0.73	0.73	0.74	689	641	540
PRY	Paraguay	23819	Juan M. Frutos	0.73	0.73	0.74	1599	1487	1254
PRY	Paraguay	23820	La Pastora	0.73	0.73	0.74	387	360	304
PRY	Paraguay	23821	Mcal. Fco. Solano Lopez	0.73	0.73	0.74	688	640	540
PRY	Paraguay	23822	Nueva Londres	0.73	0.73	0.74	356	331	280
PRY	Paraguay	23823	R.i. 3 Corrales	0.73	0.73	0.74	716	666	561
PRY	Paraguay	23824	Raul A. Oviedo	0.73	0.73	0.74	2300	2139	1804
PRY	Paraguay	23825	Repatriacion	0.73	0.73	0.74	2292	2132	1798
PRY	Paraguay	23826	San Joaquin	0.73	0.73	0.74	1292	1202	1014

PRY	Paraguay	23827	San Jose De Los Arroyos	0.73	0.73	0.74	1266	1177	993
PRY	Paraguay	23828	Santa Rosa Del Mbutuy	0.73	0.73	0.74	884	822	694
PRY	Paraguay	23829	Simon Bolivar	0.73	0.73	0.74	395	368	310
PRY	Paraguay	23830	Yhu	0.73	0.73	0.74	3208	2984	2517
PRY	Paraguay	23831	Abai	0.73	0.73	0.74	1791	1666	1405
PRY	Paraguay	23832	Buena Vista	0.73	0.73	0.74	401	373	315
PRY	Paraguay	23833	Caazapa	0.73	0.73	0.74	1631	1517	1280
PRY	Paraguay	23834	Dr. Moises Bertoni	0.73	0.73	0.74	348	324	273
PRY	Paraguay	23835	Fulgencio Yegros	0.73	0.73	0.74	482	449	378
PRY	Paraguay	23836	Gral. Higinio Morinigo	0.73	0.73	0.74	437	407	343
PRY	Paraguay	23837	Maciel	0.73	0.73	0.74	307	286	241
PRY	Paraguay	23838	San Juan Nepomuceno	0.73	0.73	0.74	1822	1694	1429
PRY	Paraguay	23839	Tavai	0.73	0.73	0.74	1060	986	832
PRY	Paraguay	23840	Yuty	0.73	0.73	0.74	2152	2002	1689
PRY	Paraguay	23841	Corpus Christi	0.73	0.73	0.74	1576	1466	1236
PRY	Paraguay	23842	Curuguaty	0.73	0.73	0.74	5013	4663	3932
PRY	Paraguay	23843	F. Caballero Alvarez	0.73	0.73	0.74	4182	3890	3281
PRY	Paraguay	23844	Itanara	0.73	0.73	0.74	202	188	158
PRY	Paraguay	23845	Salto Del Guaira	0.73	0.73	0.74	1446	1345	1134
PRY	Paraguay	23846	Villa Igatimi	0.73	0.73	0.74	1422	1322	1115
PRY	Paraguay	23847	Ypejhu	0.73	0.73	0.74	550	511	431
PRY	Paraguay	23848	Aregua	0.73	0.73	0.74	6908	6426	5419
PRY	Paraguay	23849	Asuncion	0.73	0.73	0.74	34683	32262	27209
PRY	Paraguay	23850	Capiata	0.73	0.73	0.74	18105	16842	14204
PRY	Paraguay	23851	Fdo. De La Mora	0.73	0.73	0.74	13756	12796	10792
PRY	Paraguay	23852	Guarambare	0.73	0.73	0.74	2358	2193	1850
PRY	Paraguay	23853	Ita	0.73	0.73	0.74	7687	7151	6031
PRY	Paraguay	23854	Itaugua	0.73	0.73	0.74	8325	7744	6531
PRY	Paraguay	23855	J. A. Saldivar	0.73	0.73	0.74	5250	4884	4119
PRY	Paraguay	23856	Lambare	0.73	0.73	0.74	14269	13273	11194
PRY	Paraguay	23857	Limpio	0.73	0.73	0.74	7513	6989	5894
PRY	Paraguay	23858	Luque	0.73	0.73	0.74	28520	26529	22374
PRY	Paraguay	23859	M. Roque Alonso	0.73	0.73	0.74	7509	6984	5890

PRY	Paraguay	23860	Nemby	0.73	0.73	0.74	6582	6122	5163
PRY	Paraguay	23861	Nueva Italia	0.73	0.73	0.74	1410	1311	1106
PRY	Paraguay	23862	San Antonio	0.73	0.73	0.74	2879	2678	2259
PRY	Paraguay	23863	San Lorenzo	0.73	0.73	0.74	25139	23384	19722
PRY	Paraguay	23864	Villa Elisa	0.73	0.73	0.74	5790	5386	4542
PRY	Paraguay	23865	Villeta	0.73	0.73	0.74	3701	3443	2904
PRY	Paraguay	23866	Ypacarai	0.73	0.73	0.74	3055	2842	2397
PRY	Paraguay	23867	Ypane	0.73	0.73	0.74	3059	2845	2400
PRY	Paraguay	23868	Belen	0.73	0.73	0.74	660	614	518
PRY	Paraguay	23869	Concepcion	0.73	0.73	0.74	4427	4118	3473
PRY	Paraguay	23870	Horqueta	0.73	0.73	0.74	3755	3493	2946
PRY	Paraguay	23871	Loreto	0.73	0.73	0.74	1181	1098	926
PRY	Paraguay	23872	San Carlos	0.73	0.73	0.74	719	669	564
PRY	Paraguay	23873	San Lazaro	0.73	0.73	0.74	585	545	459
PRY	Paraguay	23874	Yby Yau	0.73	0.73	0.74	1490	1386	1169
PRY	Paraguay	23875	1 De Marzo	0.73	0.73	0.74	521	484	408
PRY	Paraguay	23876	Altos	0.73	0.73	0.74	964	896	756
PRY	Paraguay	23877	Arroyos Y Esteros	0.73	0.73	0.74	1743	1621	1367
PRY	Paraguay	23878	Atyra	0.73	0.73	0.74	1177	1095	923
PRY	Paraguay	23879	Caacupe	0.73	0.73	0.74	3281	3052	2574
PRY	Paraguay	23880	Caraguatay	0.73	0.73	0.74	1079	1003	846
PRY	Paraguay	23881	Emboscada	0.73	0.73	0.74	1596	1485	1252
PRY	Paraguay	23882	Eusebio Ayala	0.73	0.73	0.74	1591	1480	1248
PRY	Paraguay	23883	Isla Pucu	0.73	0.73	0.74	593	552	465
PRY	Paraguay	23884	Itacurubi De La Cordiller	0.73	0.73	0.74	742	690	582
PRY	Paraguay	23885	Juan De Mena	0.73	0.73	0.74	513	477	402
PRY	Paraguay	23886	Loma Grande	0.73	0.73	0.74	256	238	201
PRY	Paraguay	23887	Mbacayaty Del Yhaguy	0.73	0.73	0.74	399	372	313
PRY	Paraguay	23888	Nueva Colombia	0.73	0.73	0.74	339	316	266
PRY	Paraguay	23889	Piribebuy	0.73	0.73	0.74	1660	1544	1302
PRY	Paraguay	23890	San Bernardino	0.73	0.73	0.74	1071	996	840
PRY	Paraguay	23891	San Jose Obrero	0.73	0.73	0.74	398	370	312
PRY	Paraguay	23892	Santa Elena	0.73	0.73	0.74	523	486	410

PRY	Paraguay	23893	Tobati	0.73	0.73	0.74	2013	1873	1579
PRY	Paraguay	23894	Valenzuela	0.73	0.73	0.74	514	478	403
PRY	Paraguay	23895	Borja	0.73	0.73	0.74	694	645	544
PRY	Paraguay	23896	Capitan Troche	0.73	0.73	0.74	508	473	399
PRY	Paraguay	23897	Cnel Martinez	0.73	0.73	0.74	176	164	138
PRY	Paraguay	23898	Cnel. Martinez	0.73	0.73	0.74	302	281	237
PRY	Paraguay	23899	Colonia Independencia	0.73	0.73	0.74	3392	3156	2661
PRY	Paraguay	23900	Dr. Bottrell	0.73	0.73	0.74	122	114	96
PRY	Paraguay	23901	Gral Garay	0.73	0.73	0.74	552	513	433
PRY	Paraguay	23902	Itape	0.73	0.73	0.74	506	471	397
PRY	Paraguay	23903	Iturbe	0.73	0.73	0.74	645	600	506
PRY	Paraguay	23904	Jose Fassardi	0.73	0.73	0.74	1103	1026	865
PRY	Paraguay	23905	Mbocayati	0.73	0.73	0.74	529	492	415
PRY	Paraguay	23906	Natalicio Talavera	0.73	0.73	0.74	245	228	192
PRY	Paraguay	23907	Numi	0.73	0.73	0.74	257	239	201
PRY	Paraguay	23908	San Salvador	0.73	0.73	0.74	260	242	204
PRY	Paraguay	23909	Villarica	0.73	0.73	0.74	3631	3377	2848
PRY	Paraguay	23910	Yataity	0.73	0.73	0.74	651	605	510
PRY	Paraguay	23911	Alto Vera	0.73	0.73	0.74	1845	1716	1447
PRY	Paraguay	23912	Bella Vista	0.73	0.73	0.74	782	727	613
PRY	Paraguay	23913	Cambyreta	0.73	0.73	0.74	2056	1912	1613
PRY	Paraguay	23914	Capitan Meza	0.73	0.73	0.74	1459	1357	1145
PRY	Paraguay	23915	Capitan Miranda	0.73	0.73	0.74	841	783	660
PRY	Paraguay	23916	Carlos A Lopez	0.73	0.73	0.74	1457	1356	1143
PRY	Paraguay	23917	Carmen Del Parana	0.73	0.73	0.74	522	486	410
PRY	Paraguay	23918	Coronel Bogado	0.73	0.73	0.74	1361	1266	1067
PRY	Paraguay	23919	Edelira	0.73	0.73	0.74	2089	1943	1639
PRY	Paraguay	23920	Encarnacion	0.73	0.73	0.74	7274	6767	5707
PRY	Paraguay	23921	Fram	0.73	0.73	0.74	662	616	519
PRY	Paraguay	23922	Gral Artigas	0.73	0.73	0.74	1048	974	822
PRY	Paraguay	23923	Gral. Delgado	0.73	0.73	0.74	594	552	466
PRY	Paraguay	23924	Hohenau	0.73	0.73	0.74	799	743	627
PRY	Paraguay	23925	Isla	0.73	0.73	0.74	4	3	3

PRY	Paraguay	23926	Isla Yacyreta	0.73	0.73	0.74	239	222	187
PRY	Paraguay	23927	Jesus	0.73	0.73	0.74	479	446	376
PRY	Paraguay	23928	La Paz	0.73	0.73	0.74	280	261	220
PRY	Paraguay	23929	Leandro Oviedo	0.73	0.73	0.74	367	342	288
PRY	Paraguay	23930	Mayor Otano	0.73	0.73	0.74	986	917	773
PRY	Paraguay	23931	Natalio	0.73	0.73	0.74	1420	1321	1114
PRY	Paraguay	23932	Nueva Alborada	0.73	0.73	0.74	636	592	499
PRY	Paraguay	23933	Obligado	0.73	0.73	0.74	963	895	755
PRY	Paraguay	23934	Pirapo	0.73	0.73	0.74	513	477	402
PRY	Paraguay	23935	San Cosme Y Damian	0.73	0.73	0.74	578	538	453
PRY	Paraguay	23936	San Juan	0.73	0.73	0.74	488	454	383
PRY	Paraguay	23937	San Pedro Del Parana	0.73	0.73	0.74	2631	2447	2064
PRY	Paraguay	23938	San Rafael Del Parana	0.73	0.73	0.74	1826	1698	1432
PRY	Paraguay	23939	Tomas Romero P.	0.73	0.73	0.74	2478	2305	1944
PRY	Paraguay	23940	Trinidad	0.73	0.73	0.74	542	504	425
PRY	Paraguay	23941	Yatytay	0.73	0.73	0.74	1310	1218	1028
PRY	Paraguay	23942	Ayolas	0.73	0.73	0.74	787	732	617
PRY	Paraguay	23944	San Ignacio	0.73	0.73	0.74	1899	1766	1489
PRY	Paraguay	23945	San Juan Bautista	0.73	0.73	0.74	1324	1231	1038
PRY	Paraguay	23946	San Miguel	0.73	0.73	0.74	454	422	356
PRY	Paraguay	23947	San Patricio	0.73	0.73	0.74	267	249	210
PRY	Paraguay	23948	Santa Maria	0.73	0.73	0.74	613	570	481
PRY	Paraguay	23949	Santa Rosa	0.73	0.73	0.74	1482	1379	1163
PRY	Paraguay	23950	Santiago	0.73	0.73	0.74	579	539	454
PRY	Paraguay	23951	Villa Florida	0.73	0.73	0.74	187	174	147
PRY	Paraguay	23952	Yabebyry	0.73	0.73	0.74	260	242	204
PRY	Paraguay	23953	Alberdi	0.73	0.73	0.74	489	455	383
PRY	Paraguay	23954	Cerrito	0.73	0.73	0.74	317	295	249
PRY	Paraguay	23955	Desmochados	0.73	0.73	0.74	141	131	111
PRY	Paraguay	23956	Gral. Jose E. Diaz	0.73	0.73	0.74	281	261	220
PRY	Paraguay	23957	Guazucua	0.73	0.73	0.74	270	251	212
PRY	Paraguay	23958	Humaita	0.73	0.73	0.74	229	213	180
PRY	Paraguay	23959	Isla Umbu	0.73	0.73	0.74	252	235	198

PRY	Paraguay	23960	Laureles	0.73	0.73	0.74	268	249	210
PRY	Paraguay	23961	Mayor Martinez	0.73	0.73	0.74	287	267	225
PRY	Paraguay	23962	Paso De Patria	0.73	0.73	0.74	125	116	98
PRY	Paraguay	23963	Pilar	0.73	0.73	0.74	1839	1710	1442
PRY	Paraguay	23964	San Juan Bta. Del Neembuc	0.73	0.73	0.74	431	401	338
PRY	Paraguay	23965	Tacuaras	0.73	0.73	0.74	261	243	205
PRY	Paraguay	23966	Villa Franca	0.73	0.73	0.74	92	86	73
PRY	Paraguay	23967	Villa Oliva	0.73	0.73	0.74	324	302	254
PRY	Paraguay	23968	Villalvin	0.73	0.73	0.74	173	161	135
PRY	Paraguay	23969	Acahay	0.73	0.73	0.74	1091	1015	856
PRY	Paraguay	23970	Caapucu	0.73	0.73	0.74	557	518	437
PRY	Paraguay	23971	Caballero	0.73	0.73	0.74	488	454	383
PRY	Paraguay	23972	Carapegua	0.73	0.73	0.74	2240	2084	1757
PRY	Paraguay	23973	Escobar	0.73	0.73	0.74	631	587	495
PRY	Paraguay	23974	La Colmena	0.73	0.73	0.74	367	341	288
PRY	Paraguay	23975	Mbuyapey	0.73	0.73	0.74	961	894	754
PRY	Paraguay	23976	Paraguari	0.73	0.73	0.74	1590	1479	1247
PRY	Paraguay	23977	Pirayu	0.73	0.73	0.74	1133	1054	889
PRY	Paraguay	23978	Quiindy	0.73	0.73	0.74	1321	1229	1037
PRY	Paraguay	23979	Quyquyho	0.73	0.73	0.74	518	482	407
PRY	Paraguay	23980	San Roque Gonzalez	0.73	0.73	0.74	786	731	616
PRY	Paraguay	23981	Sapucai	0.73	0.73	0.74	456	424	357
PRY	Paraguay	23982	Tebicuarymi	0.73	0.73	0.74	287	267	225
PRY	Paraguay	23983	Yaguaron	0.73	0.73	0.74	1895	1763	1487
PRY	Paraguay	23984	Ybycui	0.73	0.73	0.74	1579	1469	1239
PRY	Paraguay	23985	Ybytymi	0.73	0.73	0.74	535	498	420
PRY	Paraguay	23986	Benjamin Aceval	0.73	0.73	0.74	1413	1315	1109
PRY	Paraguay	23987	Pozo Colorado	0.73	0.73	0.74	2640	2456	2071
PRY	Paraguay	23988	Puerto Pinasco	0.73	0.73	0.74	390	363	306
PRY	Paraguay	23989	Villa Hayes	0.73	0.73	0.74	4688	4361	3678
PRY	Paraguay	23990	25 De Diciembre	0.73	0.73	0.74	740	688	580
PRY	Paraguay	23991	Antequera	0.73	0.73	0.74	263	245	207
PRY	Paraguay	23992	Chore	0.73	0.73	0.74	2964	2757	2325

PRY	Paraguay	23993	Elizardo Aquino	0.73	0.73	0.74	1755	1633	1377
PRY	Paraguay	23994	Isidoro Resquin	0.73	0.73	0.74	1725	1605	1353
PRY	Paraguay	23995	Itacurubi Del Rosario	0.73	0.73	0.74	933	868	732
PRY	Paraguay	23996	Lima	0.73	0.73	0.74	791	736	621
PRY	Paraguay	23997	Nueva Germania	0.73	0.73	0.74	1807	1681	1417
PRY	Paraguay	23998	S.pedro De Ycuamandyyu	0.73	0.73	0.74	2344	2180	1839
PRY	Paraguay	23999	San Estanislao	0.73	0.73	0.74	8099	7534	6354
PRY	Paraguay	24000	San Pablo	0.73	0.73	0.74	321	299	252
PRY	Paraguay	24001	Tacuati	0.73	0.73	0.74	743	691	583
PRY	Paraguay	24002	Union	0.73	0.73	0.74	470	437	368
PRY	Paraguay	24003	Villa Del Rosario	0.73	0.73	0.74	985	916	773
PRY	Paraguay	24004	Yataity Del Norte	0.73	0.73	0.74	1015	944	796
RWA	Rwanda	21974	Bugesera	0.00	0.00	0.00	349222	349222	349222
RWA	Rwanda	21975	Burera	0.00	0.00	0.00	317177	317177	317177
RWA	Rwanda	21976	Gakenke	0.00	0.00	0.00	326227	326227	326227
RWA	Rwanda	21977	Gasabo	0.00	0.00	0.00	551806	551806	551806
RWA	Rwanda	21978	Gatsibo	0.00	0.00	0.00	418800	418800	418800
RWA	Rwanda	21979	Gicumbi	0.00	0.00	0.00	390287	390287	390287
RWA	Rwanda	21980	Gisagara	0.00	0.00	0.00	311422	311422	311422
RWA	Rwanda	21981	Huye	0.00	0.00	0.00	318639	318639	318639
RWA	Rwanda	21982	Kamonyi	0.00	0.00	0.00	338630	338630	338630
RWA	Rwanda	21983	Karongi	0.00	0.00	0.00	317035	317035	317035
RWA	Rwanda	21984	Kayonza	0.00	0.00	0.00	336016	336016	336016
RWA	Rwanda	21985	Kicukiro	0.00	0.00	0.00	314866	314866	314866
RWA	Rwanda	21986	Kirehe	0.00	0.00	0.00	324479	324479	324479
RWA	Rwanda	21987	Muhanga	0.00	0.00	0.00	304288	304288	304288
RWA	Rwanda	21988	Musanze	0.00	0.00	0.00	361496	361496	361496
RWA	Rwanda	21989	Ngoma	0.00	0.00	0.00	329456	329456	329456
RWA	Rwanda	21990	Ngororero	0.00	0.00	0.00	315944	315944	315944
RWA	Rwanda	21991	Nyabihu	0.00	0.00	0.00	284048	284048	284048
RWA	Rwanda	21992	Nyagatare	0.00	0.00	0.00	442698	442698	442698
RWA	Rwanda	21993	Nyamagabe	0.00	0.00	0.00	326436	326436	326436
RWA	Rwanda	21994	Nyamasheke	0.00	0.00	0.00	369967	369967	369967

RWA	Rwanda	21995	Nyanza	0.00	0.00	0.00	311180	311180	311180
RWA	Rwanda	21996	Nyarugenge	0.00	0.00	0.00	268621	268621	268621
RWA	Rwanda	21997	Nyaruguru	0.00	0.00	0.00	279312	279312	279312
RWA	Rwanda	21998	Rubavu	0.00	0.00	0.00	396133	396133	396133
RWA	Rwanda	21999	Ruhango	0.00	0.00	0.00	311425	311425	311425
RWA	Rwanda	22000	Rulindo	0.00	0.00	0.00	277736	277736	277736
RWA	Rwanda	22001	Rusizi	0.00	0.00	0.00	392096	392096	392096
RWA	Rwanda	22002	Rutsiro	0.00	0.00	0.00	310939	310939	310939
RWA	Rwanda	22003	Rwamagana	0.00	0.00	0.00	302298	302298	302298
SDN	Sudan	125	Admin unit not available	0.07	0.07	0.07	56617	56617	56617
SDN	Sudan	4151	Abyei	0.41	0.44	0.47	134532	125304	112808
SDN	Sudan	37026	Al Hasaheisa	0.07	0.07	0.07	373633	373633	373633
SDN	Sudan	37027	Al Kamlin	0.00	0.00	0.00	317074	317074	317074
SDN	Sudan	37028	Al Managil	0.07	0.07	0.07	474106	474106	474106
SDN	Sudan	37029	East Al Jazeera	0.07	0.07	0.07	503025	503025	503025
SDN	Sudan	37030	North Al Jazeera	0.07	0.07	0.07	321621	321621	321621
SDN	Sudan	37031	South Al Jazeera	0.07	0.07	0.07	311343	311343	311343
SDN	Sudan	37032	Um Al Gura	0.07	0.07	0.07	354774	354774	354774
SDN	Sudan	37037	Ad Damazin	0.07	0.07	0.07	172199	172199	172199
SDN	Sudan	37038	Al Kurumuk	0.07	0.07	0.07	120132	120132	120132
SDN	Sudan	37039	Al Roseires	0.07	0.07	0.07	172626	172626	172626
SDN	Sudan	37040	Baw	0.07	0.07	0.07	117503	117503	117503
SDN	Sudan	37041	Geissan	0.07	0.07	0.07	115276	115276	115276
SDN	Sudan	37046	Al Faw	0.92	0.92	0.96	0	0	0
SDN	Sudan	37048	Al Gadaref	0.92	0.92	0.96	0	0	0
SDN	Sudan	37049	Al Galabat	0.92	0.92	0.96	0	0	0
SDN	Sudan	37050	Al Rahd	0.92	0.92	0.96	0	0	0
SDN	Sudan	37058	Al Gash	0.95	0.95	0.97	0	0	0
SDN	Sudan	37059	Hamashkorieb	0.95	0.95	0.97	0	0	0
SDN	Sudan	37060	Kassala	0.95	0.95	0.97	0	0	0
SDN	Sudan	37061	Nahr Atbara	0.95	0.95	0.97	0	0	0
SDN	Sudan	37062	Butana	0.95	0.95	0.97	0	0	0
SDN	Sudan	37063	Jabal Aulia	0.00	0.00	0.00	1406292	1406292	1406292

SDN	Sudan	37064	Karary	0.00	0.00	0.00	1240811	1240811	1240811
SDN	Sudan	37065	Khartoum	0.00	0.00	0.00	671856	671856	671856
SDN	Sudan	37066	Khartoum Bahri	0.07	0.07	0.07	402157	402157	402157
SDN	Sudan	37067	Sharg En Nile	0.07	0.07	0.07	328480	328480	328480
SDN	Sudan	37068	Um Badda	0.00	0.00	0.00	642484	642484	642484
SDN	Sudan	37069	Um Durman	0.00	0.00	0.00	440142	440142	440142
SDN	Sudan	37070	Abu Hamad	0.00	0.00	0.00	264492	264492	264492
SDN	Sudan	37071	Ad Damer	0.00	0.00	0.00	170458	170458	170458
SDN	Sudan	37072	Al Matammah	0.00	0.00	0.00	255680	255680	255680
SDN	Sudan	37073	Atbara	0.00	0.00	0.00	225835	225835	225835
SDN	Sudan	37074	Barbar	0.00	0.00	0.00	49465	49465	49465
SDN	Sudan	37075	Shendi	0.07	0.07	0.07	380721	380721	380721
SDN	Sudan	37080	Al Fasher	0.40	0.44	0.47	389681	353989	323739
SDN	Sudan	37081	Kabkabiya	0.40	0.44	0.47	125534	114036	104291
SDN	Sudan	37082	Kutum	0.40	0.44	0.47	125160	113696	103980
SDN	Sudan	37083	Mellit	0.40	0.44	0.47	252068	228981	209414
SDN	Sudan	37084	Um Kadada	0.40	0.44	0.47	152065	138137	126333
SDN	Sudan	37085	Bara	0.79	0.80	0.86	5769	0	0
SDN	Sudan	37086	Jebrat Al Sheikh	0.79	0.80	0.86	5006	0	0
SDN	Sudan	37087	Sheikan	0.79	0.80	0.86	7645	0	0
SDN	Sudan	37088	Sowdari	0.79	0.80	0.86	5676	0	0
SDN	Sudan	37089	Um Rawaba	0.79	0.80	0.86	14649	0	0
SDN	Sudan	37090	Addabah	0.00	0.00	0.00	183399	183399	183399
SDN	Sudan	37091	Dongola	0.00	0.00	0.00	446347	446347	446347
SDN	Sudan	37092	Merawi	0.00	0.00	0.00	110623	110623	110623
SDN	Sudan	37093	Wadi Halfa	0.00	0.00	0.00	110140	110140	110140
SDN	Sudan	37095	Port Sudan	0.93	0.93	0.93	0	0	0
SDN	Sudan	37096	Sinkat	0.93	0.93	0.93	0	0	0
SDN	Sudan	37097	Tokar	0.93	0.93	0.93	0	0	0
SDN	Sudan	37098	Ad Dinder	0.07	0.07	0.07	383941	383941	383941
SDN	Sudan	37099	Sennar	0.07	0.07	0.07	967360	967360	967360
SDN	Sudan	37100	Singa	0.07	0.07	0.07	419050	419050	419050
SDN	Sudan	37101	Adayala	0.40	0.44	0.47	54903	49874	45612

SDN	Sudan	37102	Al Deain	0.40	0.44	0.47	312694	284053	259780
SDN	Sudan	37103	Buram	0.40	0.44	0.47	205615	186782	170821
SDN	Sudan	37104	Edd al Fursan	0.40	0.44	0.47	101602	92296	84409
SDN	Sudan	37105	Kas	0.40	0.44	0.47	106731	96955	88670
SDN	Sudan	37106	Nyala	0.40	0.44	0.47	344087	312571	285861
SDN	Sudan	37107	Rehed Al Birdi	0.40	0.44	0.47	97788	88831	81240
SDN	Sudan	37108	Shearia	0.40	0.44	0.47	191767	174202	159316
SDN	Sudan	37109	Tulus	0.40	0.44	0.47	11064	10050	9191
SDN	Sudan	37110	Abu Jubaiyah	0.41	0.44	0.47	110746	103150	92863
SDN	Sudan	37111	Delling	0.41	0.44	0.47	140939	131272	118180
SDN	Sudan	37112	Kadugli	0.41	0.44	0.47	332653	309837	278938
SDN	Sudan	37113	Rashad	0.41	0.44	0.47	97321	90646	81606
SDN	Sudan	37114	Talodi	0.41	0.44	0.47	98160	91427	82309
SDN	Sudan	37134	Al Geneina	0.40	0.44	0.47	244130	221770	202819
SDN	Sudan	37135	Habillah	0.40	0.44	0.47	95300	86572	79174
SDN	Sudan	37136	Jebel Marra	0.40	0.44	0.47	65283	59303	54236
SDN	Sudan	37137	Kulbus	0.40	0.44	0.47	195081	177213	162070
SDN	Sudan	37138	Mukjar	0.40	0.44	0.47	103754	94251	86197
SDN	Sudan	37139	Wadi Salih	0.40	0.44	0.47	91308	82945	75857
SDN	Sudan	37140	Zallingi	0.40	0.44	0.47	146784	133340	121945
SDN	Sudan	37146	As Salam	0.41	0.44	0.47	49391	46003	41415
SDN	Sudan	37147	En Nuhud	0.79	0.80	0.86	11050	0	0
SDN	Sudan	37148	Ghebeish	0.79	0.80	0.86	6033	0	0
SDN	Sudan	37149	Lagawa	0.41	0.44	0.47	135065	125801	113255
SDN	Sudan	37150	Ad Douiem	0.07	0.07	0.07	468717	468717	468717
SDN	Sudan	37151	Al Gutaina	0.07	0.07	0.07	826790	826790	826790
SDN	Sudan	37152	Al Jabalian	0.07	0.07	0.07	301310	301310	301310
SDN	Sudan	37153	Kosti	0.07	0.07	0.07	800531	800531	800531
SDN	Sudan	40771	Halayeb	0.93	0.93	0.93	0	0	0
SDN	Sudan	68803	Seteet	0.95	0.95	0.97	0	0	0
SDN	Sudan	68804	Al Fushqa	0.92	0.92	0.96	0	0	0
SEN	Senegal	1379	Bakel	0.92	0.92	0.92	0	0	0
SEN	Senegal	1380	Birkilane	0.61	0.75	0.86	16731	4165	0

SEN	Senegal	1381	Bounkiling	0.61	0.75	0.86	26803	6672	0
SEN	Senegal	1382	Dakar	0.51	0.69	0.81	917662	338772	0
SEN	Senegal	1383	Gossas	0.61	0.75	0.86	23831	5932	0
SEN	Senegal	1384	Goudiry	0.61	0.75	0.86	25403	6324	0
SEN	Senegal	1394	Goudomp	0.61	0.75	0.86	38905	9685	0
SEN	Senegal	1395	Guinguineo	0.80	0.90	0.94	0	0	0
SEN	Senegal	1396	Kaffrine	0.61	0.75	0.86	47024	11706	0
SEN	Senegal	1397	Kedougou	0.91	0.97	0.97	0	0	0
SEN	Senegal	1408	Kolda	0.61	0.75	0.86	60297	15010	0
SEN	Senegal	1486	Koungueul	0.61	0.75	0.86	32545	8101	0
SEN	Senegal	1488	Koupentoum	0.61	0.75	0.86	35545	8848	0
SEN	Senegal	1497	Maleme hodar	0.61	0.75	0.86	24016	5978	0
SEN	Senegal	1499	Medina yoro foula	0.61	0.75	0.86	23770	5917	0
SEN	Senegal	1510	Salemata	0.91	0.95	0.97	0	0	0
SEN	Senegal	1538	Saraya	0.91	0.97	0.97	0	0	0
SEN	Senegal	1609	Sedhiou	0.61	0.75	0.86	38098	9484	0
SEN	Senegal	1649	Tambacounda	0.61	0.75	0.86	37296	9284	0
SEN	Senegal	25321	Rufisque	0.51	0.69	0.81	112659	41590	0
SEN	Senegal	25322	Bambey	0.90	0.90	0.91	0	0	0
SEN	Senegal	25323	Diourbel	0.51	0.69	0.81	86974	32108	0
SEN	Senegal	25325	Fatick	0.61	0.75	0.86	66988	16675	0
SEN	Senegal	25326	Foundiougne	0.82	0.91	0.94	0	0	0
SEN	Senegal	25329	Kaolack	0.81	0.90	0.94	0	0	0
SEN	Senegal	25330	Nioro du rip	0.61	0.75	0.86	71637	17833	0
SEN	Senegal	25333	Velingara	0.61	0.75	0.86	56547	14076	0
SEN	Senegal	25334	Kebemer	0.51	0.69	0.81	82754	30550	0
SEN	Senegal	25336	Louga	0.80	0.89	0.94	641	0	0
SEN	Senegal	25343	Mbour	0.83	0.90	0.94	0	0	0
SEN	Senegal	25344	Thies	0.84	0.91	0.94	0	0	0
SEN	Senegal	25345	Tivaoune	0.84	0.91	0.94	0	0	0
SEN	Senegal	25346	Bignona	0.61	0.75	0.86	57984	14434	0
SEN	Senegal	25347	Oussouye	0.61	0.75	0.86	8958	2230	0
SEN	Senegal	25348	Ziguinchor	0.61	0.75	0.86	51389	12792	0

SEN	Senegal	47590	Dagana	0.80	0.89	0.94	1027	0	0
SEN	Senegal	47593	Kanel	0.51	0.69	0.81	84044	31026	0
SEN	Senegal	47594	Linguere	0.80	0.89	0.94	0	0	0
SEN	Senegal	47595	Matam	0.51	0.69	0.81	97071	35836	0
SEN	Senegal	47596	Mbacke	0.90	0.90	0.91	0	0	0
SEN	Senegal	47598	Podor	0.81	0.89	0.94	0	0	0
SEN	Senegal	47599	Ranerou	0.51	0.69	0.81	23836	8799	0
SEN	Senegal	47600	Saint louis	0.82	0.90	0.94	0	0	0
SLE	Sierra Leone	25406	Kailahun	0.30	0.30	0.30	235247	235247	235247
SLE	Sierra Leone	25407	Kenema	0.75	0.75	0.75	32263	32263	32263
SLE	Sierra Leone	25408	Kono	0.30	0.30	0.30	251458	251458	251458
SLE	Sierra Leone	25409	Bombali	0.30	0.30	0.30	251116	251116	251116
SLE	Sierra Leone	25410	Kambia	0.30	0.30	0.30	159711	159711	159711
SLE	Sierra Leone	25411	Koinadugu	0.30	0.30	0.30	170102	170102	170102
SLE	Sierra Leone	25412	Port Loko	0.30	0.30	0.30	285514	285514	285514
SLE	Sierra Leone	25413	Tonkolili	0.66	0.67	0.68	57424	53148	50168
SLE	Sierra Leone	25414	Bo	0.71	0.74	0.76	54346	33976	21025
SLE	Sierra Leone	25415	Bonthe	0.30	0.30	0.30	82183	82183	82183
SLE	Sierra Leone	25416	Moyamba	0.30	0.30	0.30	178790	178790	178790
SLE	Sierra Leone	25417	Pujehun	0.30	0.30	0.30	154479	154479	154479
SLE	Sierra Leone	25418	Western Area	0.30	0.30	0.30	606261	606261	606261
SOM	Somalia	25657	Ceel Barde	0.00	0.00	0.00	34312	34312	34312
SOM	Somalia	25658	Rab Dhuure	0.00	0.00	0.00	43933	43933	43933
SOM	Somalia	25659	Tayeeglow	0.00	0.00	0.00	94375	94375	94375
SOM	Somalia	25660	Waajid	0.00	0.00	0.00	81641	81641	81641
SOM	Somalia	25661	Xudur	0.00	0.00	0.00	108565	108565	108565
SOM	Somalia	25668	Baydhaba	0.00	0.00	0.00	398905	398905	398905
SOM	Somalia	25669	Buur Hakaba	0.00	0.00	0.00	146670	146670	146670
SOM	Somalia	25670	Diinsoor	0.00	0.00	0.00	88503	88503	88503
SOM	Somalia	25671	Qansax Dheere	0.00	0.00	0.00	115127	115127	115127
SOM	Somalia	25673	Cabudwaaq	0.00	0.00	0.00	47513	47513	47513
SOM	Somalia	25674	Cadaado	0.00	0.00	0.00	53673	53673	53673
SOM	Somalia	25675	Ceel Buur	0.00	0.00	0.00	92164	92164	92164

SOM	Somalia	25676	Ceel Dheer	0.00	0.00	0.00	85174	85174	85174
SOM	Somalia	25677	Dhuusamarreeb	0.00	0.00	0.00	106871	106871	106871
SOM	Somalia	25678	Baardheere	0.00	0.00	0.00	123727	123727	123727
SOM	Somalia	25679	Belet Xaawo	0.00	0.00	0.00	60757	60757	60757
SOM	Somalia	25680	Ceel Waaq	0.00	0.00	0.00	23243	23243	23243
SOM	Somalia	25681	Doolow	0.00	0.00	0.00	27498	27498	27498
SOM	Somalia	25682	Garbahaarey	0.00	0.00	0.00	66824	66824	66824
SOM	Somalia	25683	Luuq	0.00	0.00	0.00	73148	73148	73148
SOM	Somalia	25684	Belet Weyne	0.00	0.00	0.00	201178	201178	201178
SOM	Somalia	25685	Bulo Burto	0.00	0.00	0.00	129887	129887	129887
SOM	Somalia	25686	Jalalaqsi	0.00	0.00	0.00	54993	54993	54993
SOM	Somalia	25687	Afmadow	0.00	0.00	0.00	60669	60669	60669
SOM	Somalia	25688	Badhaadhe	0.00	0.00	0.00	45941	45941	45941
SOM	Somalia	25689	Jamaame	0.00	0.00	0.00	161270	161270	161270
SOM	Somalia	25690	Kismaayo	0.00	0.00	0.00	194888	194888	194888
SOM	Somalia	25691	Afgooye	0.00	0.00	0.00	303398	303398	303398
SOM	Somalia	25692	Baraawe	0.00	0.00	0.00	66617	66617	66617
SOM	Somalia	25693	Kurtunwaarey	0.00	0.00	0.00	64657	64657	64657
SOM	Somalia	25694	Marka	0.00	0.00	0.00	221467	221467	221467
SOM	Somalia	25695	Qoryooley	0.00	0.00	0.00	159869	159869	159869
SOM	Somalia	25696	Sablaale	0.00	0.00	0.00	50349	50349	50349
SOM	Somalia	25697	Wanla Weyn	0.00	0.00	0.00	181806	181806	181806
SOM	Somalia	25698	Bu'aale	0.00	0.00	0.00	69658	69658	69658
SOM	Somalia	25699	Jilib	0.00	0.00	0.00	132792	132792	132792
SOM	Somalia	25700	Saakow	0.00	0.00	0.00	77192	77192	77192
SOM	Somalia	25701	Adan Yabaal	0.00	0.00	0.00	73232	73232	73232
SOM	Somalia	25702	Balcad	0.00	0.00	0.00	158328	158328	158328
SOM	Somalia	25703	Cadale	0.00	0.00	0.00	53499	53499	53499
SOM	Somalia	25704	Jowhar	0.00	0.00	0.00	327854	327854	327854
SSD	South Sudan	37022	Aliab	0.00	0.00	0.00	59120	59120	59120
SSD	South Sudan	37023	Rumbek	0.00	0.00	0.00	435080	435080	435080
SSD	South Sudan	37024	Shobet	0.00	0.00	0.00	152982	152982	152982
SSD	South Sudan	37025	Yerol	0.00	0.00	0.00	196555	196555	196555

SSD	South Sudan	37033	Bahr Al Jabal	0.00	0.00	0.00	431168	431168	431168
SSD	South Sudan	37034	Kajo Kaji	0.00	0.00	0.00	201984	201984	201984
SSD	South Sudan	37035	Nahr Yei	0.00	0.00	0.00	520006	520006	520006
SSD	South Sudan	37036	Terkaka	0.00	0.00	0.00	176914	176914	176914
SSD	South Sudan	37042	Amatonge	0.00	0.00	0.00	272740	272740	272740
SSD	South Sudan	37043	Kapoeta	0.00	0.00	0.00	347829	347829	347829
SSD	South Sudan	37044	Magwi	0.00	0.00	0.00	208616	208616	208616
SSD	South Sudan	37045	Shokodom	0.00	0.00	0.00	183593	183593	183593
SSD	South Sudan	37051	Akobo	0.00	0.00	0.00	223966	223966	223966
SSD	South Sudan	37052	Ayod	0.00	0.00	0.00	275196	275196	275196
SSD	South Sudan	37053	Bor	0.00	0.00	0.00	178478	178478	178478
SSD	South Sudan	37054	Fam Al Zaraf	0.00	0.00	0.00	149874	149874	149874
SSD	South Sudan	37055	Nahr Atiem	0.00	0.00	0.00	264581	264581	264581
SSD	South Sudan	37056	Pibor	0.00	0.00	0.00	302750	302750	302750
SSD	South Sudan	37057	Wat	0.00	0.00	0.00	235553	235553	235553
SSD	South Sudan	37076	Aryat	0.00	0.00	0.00	59195	59195	59195
SSD	South Sudan	37077	Aweil	0.00	0.00	0.00	401363	401363	401363
SSD	South Sudan	37078	Malek	0.00	0.00	0.00	98109	98109	98109
SSD	South Sudan	37079	Wanjuk	0.00	0.00	0.00	308621	308621	308621
SSD	South Sudan	37115	Al Leiri	0.00	0.00	0.00	258967	258967	258967
SSD	South Sudan	37116	Al Mayom	0.00	0.00	0.00	140536	140536	140536
SSD	South Sudan	37117	Faring	0.00	0.00	0.00	43360	43360	43360
SSD	South Sudan	37118	Rabkona	0.00	0.00	0.00	271242	271242	271242
SSD	South Sudan	37119	Al Mabien	0.00	0.00	0.00	39939	39939	39939
SSD	South Sudan	37120	Al Renk	0.00	0.00	0.00	168625	168625	168625
SSD	South Sudan	37121	Baleit	0.00	0.00	0.00	83436	83436	83436
SSD	South Sudan	37122	Fashooda	0.00	0.00	0.00	75072	75072	75072
SSD	South Sudan	37123	Malut	0.00	0.00	0.00	99208	99208	99208
SSD	South Sudan	37124	Mayot	0.00	0.00	0.00	205268	205268	205268
SSD	South Sudan	37125	Sobat	0.00	0.00	0.00	326144	326144	326144
SSD	South Sudan	37126	Tonga	0.00	0.00	0.00	214109	214109	214109
SSD	South Sudan	37127	Gogrial	0.00	0.00	0.00	370861	370861	370861
SSD	South Sudan	37128	Nahr Lol	0.00	0.00	0.00	270379	270379	270379

SSD	South Sudan	37129	Tonj	0.00	0.00	0.00	255303	255303	255303
SSD	South Sudan	37130	Warab	0.00	0.00	0.00	233629	233629	233629
SSD	South Sudan	37131	Nahr Jur	0.00	0.00	0.00	68785	68785	68785
SSD	South Sudan	37132	Raja	0.00	0.00	0.00	51697	51697	51697
SSD	South Sudan	37133	Wau	0.00	0.00	0.00	245246	245246	245246
SSD	South Sudan	37141	Meridi	0.00	0.00	0.00	176986	176986	176986
SSD	South Sudan	37142	Mundri	0.00	0.00	0.00	159016	159016	159016
SSD	South Sudan	37143	Tombura	0.00	0.00	0.00	148717	148717	148717
SSD	South Sudan	37144	Yambio	0.00	0.00	0.00	255599	255599	255599
STP	Sao Tome and Principe	25303	Admin unit not available	0.32	0.32	0.32	3555	3555	3555
STP	Sao Tome and Principe	25304	Admin unit not available	0.32	0.32	0.32	88005	88005	88005
SUR	Suriname	25880	Brownsweg	0.14	0.14	0.14	1038	1038	1038
SUR	Suriname	25881	Centrum	0.14	0.14	0.14	3215	3215	3215
SUR	Suriname	25882	Klaaskreek	0.14	0.14	0.14	1585	1585	1585
SUR	Suriname	25883	Kwakoegron	0.14	0.14	0.14	203	203	203
SUR	Suriname	25884	Marchalkreek	0.14	0.14	0.14	531	531	531
SUR	Suriname	25885	Sarakreek	0.14	0.14	0.14	1696	1696	1696
SUR	Suriname	25886	Alkmaar	0.14	0.14	0.14	3717	3717	3717
SUR	Suriname	25887	Bakki	0.14	0.14	0.14	321	321	321
SUR	Suriname	25888	Margaretha	0.14	0.14	0.14	804	804	804
SUR	Suriname	25889	Meerzorg	0.14	0.14	0.14	10402	10402	10402
SUR	Suriname	25890	Nieuw Amsterdam	0.14	0.14	0.14	3520	3520	3520
SUR	Suriname	25891	Tamanredjo	0.14	0.14	0.14	4591	4591	4591
SUR	Suriname	25892	Johanna Maria	0.14	0.14	0.14	532	532	532
SUR	Suriname	25893	Totness	0.14	0.14	0.14	1397	1397	1397
SUR	Suriname	25894	Welgelegen	0.14	0.14	0.14	11258	11258	11258
SUR	Suriname	25895	Albina	0.14	0.14	0.14	3299	3299	3299
SUR	Suriname	25896	Galibi	0.14	0.14	0.14	499	499	499
SUR	Suriname	25897	Moengo	0.14	0.14	0.14	7095	7095	7095
SUR	Suriname	25898	Moengotapoe	0.14	0.14	0.14	489	489	489
SUR	Suriname	25899	Patamacca	0.14	0.14	0.14	353	353	353
SUR	Suriname	25900	Wanhatti	0.14	0.14	0.14	353	353	353
SUR	Suriname	25901	Groot Henar	0.14	0.14	0.14	1711	1711	1711

SUR	Suriname	25902	Nieuw Nickerie	0.14	0.14	0.14	6758	6758	6758
SUR	Suriname	25903	Oostelijke Polders	0.14	0.14	0.14	5102	5102	5102
SUR	Suriname	25904	Wageningen	0.14	0.14	0.14	1736	1736	1736
SUR	Suriname	25905	Westelijke Polders	0.14	0.14	0.14	4979	4979	4979
SUR	Suriname	25906	Bigi Poika	0.14	0.14	0.14	434	434	434
SUR	Suriname	25907	Carolina	0.14	0.14	0.14	3445	3445	3445
SUR	Suriname	25908	Noord	0.14	0.14	0.14	7150	7150	7150
SUR	Suriname	25909	Oost	0.14	0.14	0.14	5762	5762	5762
SUR	Suriname	25910	Zuid	0.14	0.14	0.14	4613	4613	4613
SUR	Suriname	25911	Beekhuizen	0.14	0.14	0.14	9476	9476	9476
SUR	Suriname	25912	Blauwgrond	0.14	0.14	0.14	19955	19955	19955
SUR	Suriname	25913	Centrum	0.14	0.14	0.14	10919	10919	10919
SUR	Suriname	25914	Flora	0.14	0.14	0.14	11129	11129	11129
SUR	Suriname	25915	Latour	0.14	0.14	0.14	17457	17457	17457
SUR	Suriname	25916	Livorno	0.14	0.14	0.14	6344	6344	6344
SUR	Suriname	25917	Munder	0.14	0.14	0.14	9571	9571	9571
SUR	Suriname	25918	Pontbuiten	0.14	0.14	0.14	14546	14546	14546
SUR	Suriname	25919	Rainville	0.14	0.14	0.14	13202	13202	13202
SUR	Suriname	25920	Tammenga	0.14	0.14	0.14	9720	9720	9720
SUR	Suriname	25921	Weg Naar Zee	0.14	0.14	0.14	10479	10479	10479
SUR	Suriname	25922	Welgelegen	0.14	0.14	0.14	1641	1641	1641
SUR	Suriname	25923	Calcutta	0.14	0.14	0.14	353	353	353
SUR	Suriname	25924	Groningen	0.14	0.14	0.14	1641	1641	1641
SUR	Suriname	25925	Jarikaba	0.14	0.14	0.14	4375	4375	4375
SUR	Suriname	25926	Kampong Baroe	0.14	0.14	0.14	2140	2140	2140
SUR	Suriname	25927	Tijgerkreek	0.14	0.14	0.14	2186	2186	2186
SUR	Suriname	25928	Wayamboweg	0.14	0.14	0.14	1410	1410	1410
SUR	Suriname	25929	Boven Suriname	0.14	0.14	0.14	12130	12130	12130
SUR	Suriname	25930	Coeroeni	0.14	0.14	0.14	633	633	633
SUR	Suriname	25931	Coppename	0.14	0.14	0.14	431	431	431
SUR	Suriname	25932	Kabalebo	0.14	0.14	0.14	1572	1572	1572
SUR	Suriname	25933	Saramacca	0.14	0.14	0.14	913	913	913
SUR	Suriname	25934	Tapanahony	0.14	0.14	0.14	8917	8917	8917

SUR	Suriname	25935	De Nieuwe Grond	0.14	0.14	0.14	20199	20199	20199
SUR	Suriname	25936	Domburg	0.14	0.14	0.14	3519	3519	3519
SUR	Suriname	25937	Houttuin	0.14	0.14	0.14	11700	11700	11700
SUR	Suriname	25938	Koewarasan	0.14	0.14	0.14	22576	22576	22576
SUR	Suriname	25939	Kwatta	0.14	0.14	0.14	10007	10007	10007
SUR	Suriname	25940	Lelydorp	0.14	0.14	0.14	13897	13897	13897
SUR	Suriname	25941	Saramacca Polder	0.14	0.14	0.14	7672	7672	7672
TCD	Chad	65351	Abker	0.18	0.19	0.19	29053	28518	28242
TCD	Chad	65352	Abou-charib-i	0.39	0.47	0.53	11514	9184	7627
TCD	Chad	65353	Abou-charib-ii	0.39	0.47	0.53	4438	3540	2940
TCD	Chad	65354	Afrouk	0.18	0.19	0.19	11673	11457	11347
TCD	Chad	65355	Alako	0.18	0.19	0.19	8434	8279	8199
TCD	Chad	65356	Am-timan	0.18	0.19	0.19	36734	36057	35709
TCD	Chad	65357	Amdobak	0.18	0.19	0.19	16883	16572	16412
TCD	Chad	65358	Amladoba	0.18	0.19	0.19	17966	17635	17465
TCD	Chad	65359	Andoum	0.18	0.19	0.19	18030	17698	17527
TCD	Chad	65360	Arada	0.39	0.47	0.53	39981	31892	26485
TCD	Chad	65361	Autochtone	0.18	0.19	0.19	19935	19568	19379
TCD	Chad	65362	Ba-illi	0.18	0.19	0.19	42053	41278	40880
TCD	Chad	65363	Bagaye	0.18	0.19	0.19	13682	13430	13301
TCD	Chad	65364	Bah	0.18	0.19	0.19	7104	6973	6906
TCD	Chad	65365	Bakhat	0.18	0.19	0.19	4556	4472	4429
TCD	Chad	65366	Bale	0.18	0.19	0.19	10566	10372	10272
TCD	Chad	65367	Bali	0.39	0.47	0.53	3574	2851	2368
TCD	Chad	65368	Balimba	0.18	0.19	0.19	27531	27023	26762
TCD	Chad	65369	Baltoubaye	0.18	0.19	0.19	7018	6888	6822
TCD	Chad	65370	Banda	0.18	0.19	0.19	7505	7366	7295
TCD	Chad	65371	Bandala	0.18	0.19	0.19	4087	4012	3973
TCD	Chad	65372	Bangoul	0.18	0.19	0.19	6651	6528	6465
TCD	Chad	65373	Bao	0.18	0.19	0.19	12198	11973	11857
TCD	Chad	65374	Barde	0.39	0.47	0.53	21449	17109	14208
TCD	Chad	65375	Barh-azoum-am-timan	0.18	0.19	0.19	9774	9594	9501
TCD	Chad	65376	Barh-azoum-goz-beida	0.39	0.47	0.53	21233	16937	14065

TCD	Chad	65377	Batha-lairi	0.18	0.19	0.19	18871	18523	18345
TCD	Chad	65378	Bebalem	0.18	0.19	0.19	12998	12759	12636
TCD	Chad	65379	Bebedjia	0.18	0.19	0.19	34097	33468	33145
TCD	Chad	65380	Bebo-pen	0.18	0.19	0.19	19902	19535	19347
TCD	Chad	65381	Beboni	0.18	0.19	0.19	22152	21743	21534
TCD	Chad	65382	Beboro	0.18	0.19	0.19	9028	8861	8776
TCD	Chad	65383	Beboto	0.18	0.19	0.19	43692	42886	42473
TCD	Chad	65384	Bedaya	0.18	0.19	0.19	19516	19156	18971
TCD	Chad	65385	Bedjondo	0.18	0.19	0.19	27344	26840	26581
TCD	Chad	65386	Begara	0.18	0.19	0.19	6114	6001	5943
TCD	Chad	65387	Beinamar	0.18	0.19	0.19	23601	23165	22942
TCD	Chad	65388	Beissa	0.18	0.19	0.19	7984	7837	7761
TCD	Chad	65389	Bekamba-koumra	0.18	0.19	0.19	15877	15584	15434
TCD	Chad	65390	Bekamba-moissala	0.18	0.19	0.19	11733	11516	11405
TCD	Chad	65391	Bekan	0.18	0.19	0.19	21378	20984	20782
TCD	Chad	65392	Bekourou	0.18	0.19	0.19	5286	5189	5139
TCD	Chad	65393	Beladjia	0.18	0.19	0.19	4313	4234	4193
TCD	Chad	65394	Bengoro	0.18	0.19	0.19	18269	17932	17759
TCD	Chad	65395	Benoye	0.18	0.19	0.19	44462	43642	43221
TCD	Chad	65396	Bere-tchad	0.18	0.19	0.19	35088	34441	34109
TCD	Chad	65397	Berem	0.18	0.19	0.19	39312	38587	38215
TCD	Chad	65398	Bero	0.18	0.19	0.19	8975	8809	8724
TCD	Chad	65399	Bessada	0.18	0.19	0.19	23331	22901	22680
TCD	Chad	65400	Bessao	0.18	0.19	0.19	23960	23518	23291
TCD	Chad	65401	Bessara	0.18	0.19	0.19	4016	3942	3904
TCD	Chad	65402	Besseye	0.18	0.19	0.19	8328	8175	8096
TCD	Chad	65403	Beti	0.18	0.19	0.19	23435	23003	22781
TCD	Chad	65404	Bidio	0.18	0.19	0.19	18799	18452	18274
TCD	Chad	65405	Binder	0.18	0.19	0.19	47357	46484	46036
TCD	Chad	65406	Bipare	0.18	0.19	0.19	5086	4993	4944
TCD	Chad	65407	Birak	0.39	0.47	0.53	6774	5403	4487
TCD	Chad	65408	Biramanda	0.18	0.19	0.19	13582	13331	13203
TCD	Chad	65409	Birguit	0.18	0.19	0.19	12945	12706	12583

TCD	Chad	65410	Bitkine	0.18	0.19	0.19	25937	25459	25213
TCD	Chad	65411	Bodo	0.18	0.19	0.19	53565	52577	52070
TCD	Chad	65412	Bogomoro	0.18	0.19	0.19	11340	11130	11023
TCD	Chad	65413	Bohobe	0.18	0.19	0.19	2986	2931	2902
TCD	Chad	65414	Bokoro/tania	0.18	0.19	0.19	31225	30649	30353
TCD	Chad	65415	Bol	0.18	0.19	0.19	180982	177644	175931
TCD	Chad	65416	Bongor	0.18	0.19	0.19	47095	46227	45781
TCD	Chad	65417	Borkou	0.18	0.19	0.19	81282	79783	79014
TCD	Chad	65418	Boro	0.18	0.19	0.19	80333	78851	78091
TCD	Chad	65419	Bougoumene	0.18	0.19	0.19	20425	20048	19855
TCD	Chad	65420	Boum-kebir	0.18	0.19	0.19	20757	20374	20178
TCD	Chad	65421	Bourou	0.18	0.19	0.19	5955	5845	5789
TCD	Chad	65422	Bourtail	0.18	0.19	0.19	16107	15810	15658
TCD	Chad	65423	Bousso	0.18	0.19	0.19	59903	58798	58231
TCD	Chad	65424	Boye-bessao	0.18	0.19	0.19	16175	15876	15723
TCD	Chad	65425	Dababa	0.18	0.19	0.19	18585	18242	18066
TCD	Chad	65426	Dadjile	0.18	0.19	0.19	9116	8948	8862
TCD	Chad	65427	Dadjo	0.18	0.19	0.19	5798	5691	5636
TCD	Chad	65428	Dadjo-i	0.18	0.19	0.19	80937	79444	78678
TCD	Chad	65429	Dadjo-ii	0.18	0.19	0.19	30278	29720	29433
TCD	Chad	65430	Dagal	0.18	0.19	0.19	31308	30731	30434
TCD	Chad	65431	Daguela	0.18	0.19	0.19	31784	31198	30897
TCD	Chad	65432	Dangaleat	0.18	0.19	0.19	6534	6414	6352
TCD	Chad	65433	Dar-salim	0.18	0.19	0.19	13391	13144	13017
TCD	Chad	65434	Darbe	0.18	0.19	0.19	3189	3131	3100
TCD	Chad	65435	Dari	0.18	0.19	0.19	15367	15084	14938
TCD	Chad	65436	Dekakire	0.18	0.19	0.19	2560	2513	2489
TCD	Chad	65437	Delbian	0.18	0.19	0.19	23888	23447	23221
TCD	Chad	65438	Deli	0.18	0.19	0.19	10032	9847	9752
TCD	Chad	65439	Dembo	0.18	0.19	0.19	14613	14343	14205
TCD	Chad	65440	Deredia	0.18	0.19	0.19	10911	10710	10607
TCD	Chad	65441	Deressia	0.18	0.19	0.19	17251	16933	16769
TCD	Chad	65442	Dhok/dopdop	0.18	0.19	0.19	9324	9152	9064

TCD	Chad	65443	Dilingala	0.18	0.19	0.19	15479	15193	15047
TCD	Chad	65444	Dindje	0.18	0.19	0.19	5170	5074	5026
TCD	Chad	65445	Djaatne	0.18	0.19	0.19	2663	2614	2588
TCD	Chad	65446	Djarao	0.18	0.19	0.19	11242	11035	10928
TCD	Chad	65447	Djimeze	0.39	0.47	0.53	6300	5026	4174
TCD	Chad	65448	Djoli	0.18	0.19	0.19	7810	7666	7592
TCD	Chad	65449	Djonkor-aboutelfane	0.18	0.19	0.19	25400	24931	24691
TCD	Chad	65450	Djonkor-guera	0.18	0.19	0.19	7157	7025	6958
TCD	Chad	65451	Djoumane	0.18	0.19	0.19	3858	3787	3750
TCD	Chad	65452	Doba	0.18	0.19	0.19	34554	33917	33590
TCD	Chad	65453	Dobo	0.18	0.19	0.19	14180	13919	13784
TCD	Chad	65454	Dodinda	0.18	0.19	0.19	7437	7300	7229
TCD	Chad	65455	Dokora	0.18	0.19	0.19	5592	5489	5436
TCD	Chad	65456	Domo	0.18	0.19	0.19	15421	15137	14991
TCD	Chad	65457	Dormon	0.18	0.19	0.19	3223	3163	3133
TCD	Chad	65458	Doubadene	0.18	0.19	0.19	6615	6493	6430
TCD	Chad	65459	Doudeye	0.18	0.19	0.19	19584	19222	19037
TCD	Chad	65460	Doue	0.18	0.19	0.19	31507	30926	30628
TCD	Chad	65461	Dourbali/abouguerne	0.18	0.19	0.19	54290	53289	52775
TCD	Chad	65462	Dourene	0.39	0.47	0.53	8903	7101	5897
TCD	Chad	65463	El-fass	0.18	0.19	0.19	8996	8830	8745
TCD	Chad	65464	Ennedi	0.18	0.19	0.19	119793	117584	116450
TCD	Chad	65465	Erde-pala	0.18	0.19	0.19	81261	79762	78993
TCD	Chad	65466	Ere	0.18	0.19	0.19	2235	2194	2173
TCD	Chad	65467	Fare	0.39	0.47	0.53	2530	2018	1676
TCD	Chad	65468	Fianga	0.18	0.19	0.19	33125	32514	32200
TCD	Chad	65469	Fitri	0.18	0.19	0.19	90881	89205	88344
TCD	Chad	65470	Fongoro	0.39	0.47	0.53	22874	18246	15152
TCD	Chad	65471	Gabian	0.18	0.19	0.19	16808	16498	16339
TCD	Chad	65472	Gabri-ngolo	0.18	0.19	0.19	6508	6388	6326
TCD	Chad	65473	Gadjibian	0.18	0.19	0.19	18506	18165	17989
TCD	Chad	65474	Gagal	0.18	0.19	0.19	25695	25221	24978
TCD	Chad	65475	Gami	0.18	0.19	0.19	18414	18074	17900

TCD	Chad	65476	Gnere	0.39	0.47	0.53	7974	6361	5282
TCD	Chad	65477	Gogmi	0.18	0.19	0.19	7465	7327	7256
TCD	Chad	65478	Gon	0.18	0.19	0.19	7668	7526	7454
TCD	Chad	65479	Gore	0.18	0.19	0.19	14342	14078	13942
TCD	Chad	65480	Gore-loc	0.18	0.19	0.19	14367	14102	13966
TCD	Chad	65481	Gouey-goudoum	0.18	0.19	0.19	8818	8655	8571
TCD	Chad	65482	Gouin	0.18	0.19	0.19	6929	6801	6736
TCD	Chad	65483	Goulaye	0.18	0.19	0.19	32411	31813	31507
TCD	Chad	65484	Goumadji	0.18	0.19	0.19	9025	8858	8773
TCD	Chad	65485	Goundi	0.18	0.19	0.19	34162	33532	33209
TCD	Chad	65486	Gounou	0.18	0.19	0.19	22370	21958	21746
TCD	Chad	65487	Gounou-gaya	0.18	0.19	0.19	13952	13695	13562
TCD	Chad	65488	Gourouf	0.39	0.47	0.53	4284	3417	2838
TCD	Chad	65489	Goz-beida	0.39	0.47	0.53	9589	7649	6352
TCD	Chad	65490	Guegou	0.18	0.19	0.19	3092	3035	3005
TCD	Chad	65491	Guereda	0.39	0.47	0.53	4198	3349	2781
TCD	Chad	65492	Guergne	0.39	0.47	0.53	25377	20242	16810
TCD	Chad	65493	Guerri	0.18	0.19	0.19	34503	33866	33540
TCD	Chad	65494	Ham	0.18	0.19	0.19	4926	4835	4788
TCD	Chad	65495	Hemat-am-timan	0.18	0.19	0.19	10487	10293	10194
TCD	Chad	65496	Hemat-haraze	0.18	0.19	0.19	7424	7287	7217
TCD	Chad	65497	Illili	0.18	0.19	0.19	5141	5047	4998
TCD	Chad	65498	Kaba-donia	0.18	0.19	0.19	6434	6315	6254
TCD	Chad	65499	Kaba-roangar	0.18	0.19	0.19	8082	7933	7856
TCD	Chad	65500	Kabalaye	0.18	0.19	0.19	12157	11932	11817
TCD	Chad	65501	Kachimel-abeche	0.18	0.19	0.19	104917	102982	101989
TCD	Chad	65502	Kadada	0.18	0.19	0.19	121356	119118	117969
TCD	Chad	65503	Kadjeske-am-dam	0.18	0.19	0.19	45213	44379	43951
TCD	Chad	65504	Kadjeske-goz-beida	0.39	0.47	0.53	9281	7404	6148
TCD	Chad	65505	Kado	0.39	0.47	0.53	21672	17288	14356
TCD	Chad	65506	Kaga	0.18	0.19	0.19	2617	2569	2544
TCD	Chad	65507	Kapka-bakaore	0.39	0.47	0.53	16759	13368	11102
TCD	Chad	65508	Kara/maibo-goulaye	0.18	0.19	0.19	47513	46637	46187

TCD	Chad	65509	Kariade-boum	0.18	0.19	0.19	10801	10602	10500
TCD	Chad	65510	Kassine	0.39	0.47	0.53	7477	5964	4953
TCD	Chad	65511	Katoa	0.18	0.19	0.19	23119	22693	22474
TCD	Chad	65512	Kelo	0.18	0.19	0.19	57616	56554	56008
TCD	Chad	65513	Kenga	0.18	0.19	0.19	82070	80557	79780
TCD	Chad	65514	Kera	0.18	0.19	0.19	29427	28885	28606
TCD	Chad	65515	Keuni	0.18	0.19	0.19	22092	21685	21476
TCD	Chad	65516	Khozam	0.18	0.19	0.19	7994	7846	7770
TCD	Chad	65517	Kibet-angreb	0.18	0.19	0.19	7066	6935	6868
TCD	Chad	65518	Kibet-soum-soum	0.18	0.19	0.19	2953	2899	2871
TCD	Chad	65519	Kim	0.18	0.19	0.19	28940	28406	28132
TCD	Chad	65520	Kimre	0.18	0.19	0.19	5243	5146	5096
TCD	Chad	65521	Kobe-nord-est	0.39	0.47	0.53	24843	19817	16457
TCD	Chad	65522	Kobe-nord-ouest	0.39	0.47	0.53	14408	11493	9544
TCD	Chad	65523	Kobe-sud	0.39	0.47	0.53	17558	14005	11631
TCD	Chad	65524	Kodoye-i	0.39	0.47	0.53	1954	1559	1294
TCD	Chad	65525	Kodoye-ii	0.39	0.47	0.53	689	550	456
TCD	Chad	65526	Koffa	0.18	0.19	0.19	14713	14442	14303
TCD	Chad	65527	Kognere	0.18	0.19	0.19	28353	27830	27562
TCD	Chad	65528	Kokaga	0.18	0.19	0.19	25327	24860	24620
TCD	Chad	65529	Koldaga	0.18	0.19	0.19	14202	13940	13806
TCD	Chad	65530	Kolobo	0.18	0.19	0.19	834	819	811
TCD	Chad	65531	Kolon	0.18	0.19	0.19	30200	29643	29357
TCD	Chad	65532	Kolonga	0.39	0.47	0.53	7571	6039	5015
TCD	Chad	65533	Koloye	0.39	0.47	0.53	14898	11883	9869
TCD	Chad	65534	Kome/ndobele	0.18	0.19	0.19	24701	24245	24011
TCD	Chad	65535	Korbo/kordo	0.18	0.19	0.19	10092	9906	9811
TCD	Chad	65536	Korbol	0.18	0.19	0.19	55269	54249	53726
TCD	Chad	65537	Korio	0.18	0.19	0.19	32188	31594	31289
TCD	Chad	65538	Koro-tchad	0.18	0.19	0.19	3376	3314	3282
TCD	Chad	65539	Koskobo	0.18	0.19	0.19	4534	4451	4408
TCD	Chad	65540	Kotongoro	0.18	0.19	0.19	668	656	649
TCD	Chad	65541	Kouga-tchad	0.18	0.19	0.19	13292	13047	12921

TCD	Chad	65542	Kouka-adjob	0.18	0.19	0.19	11335	11126	11018
TCD	Chad	65543	Kouka-ati	0.18	0.19	0.19	21049	20661	20462
TCD	Chad	65544	Koumi	0.18	0.19	0.19	24019	23576	23348
TCD	Chad	65545	Koumogo	0.18	0.19	0.19	16835	16524	16365
TCD	Chad	65546	Koumra	0.18	0.19	0.19	73160	71811	71118
TCD	Chad	65547	Koursigue	0.39	0.47	0.53	4164	3322	2758
TCD	Chad	65548	Koutoutou	0.18	0.19	0.19	10416	10224	10125
TCD	Chad	65549	Krim-krim	0.18	0.19	0.19	40574	39825	39441
TCD	Chad	65550	Kyabe	0.18	0.19	0.19	21707	21306	21101
TCD	Chad	65551	Lagon	0.18	0.19	0.19	57771	56706	56159
TCD	Chad	65552	Lai	0.18	0.19	0.19	26266	25781	25533
TCD	Chad	65553	Lame	0.18	0.19	0.19	13457	13209	13082
TCD	Chad	65554	Lao-kassi	0.18	0.19	0.19	10637	10441	10341
TCD	Chad	65555	Lele	0.18	0.19	0.19	52952	51975	51474
TCD	Chad	65556	Leo-mbassa	0.18	0.19	0.19	14370	14105	13969
TCD	Chad	65557	Lere-tchad	0.18	0.19	0.19	63593	62420	61818
TCD	Chad	65558	Lima	0.39	0.47	0.53	17147	13677	11358
TCD	Chad	65559	Loumbogo	0.18	0.19	0.19	6841	6715	6650
TCD	Chad	65560	Mabraone	0.39	0.47	0.53	5734	4574	3798
TCD	Chad	65561	Mabrone	0.39	0.47	0.53	20613	16442	13655
TCD	Chad	65562	Madiago	0.18	0.19	0.19	44119	43306	42888
TCD	Chad	65563	Magao	0.18	0.19	0.19	17548	17225	17059
TCD	Chad	65564	Mahim-toki	0.18	0.19	0.19	14593	14324	14185
TCD	Chad	65565	Mai-ache	0.18	0.19	0.19	11125	10920	10815
TCD	Chad	65566	Maibo-mbaye	0.18	0.19	0.19	17194	16877	16714
TCD	Chad	65567	Malboum/hollom	0.18	0.19	0.19	51690	50736	50247
TCD	Chad	65568	Mandjafa	0.18	0.19	0.19	22338	21926	21714
TCD	Chad	65569	Mandjobo	0.18	0.19	0.19	29091	28555	28279
TCD	Chad	65570	Mango	0.18	0.19	0.19	10384	10193	10094
TCD	Chad	65571	Mangueigne	0.18	0.19	0.19	5085	4991	4943
TCD	Chad	65572	Mani	0.18	0.19	0.19	53457	52471	51965
TCD	Chad	65573	Manso	0.18	0.19	0.19	6791	6666	6602
TCD	Chad	65574	Marabe	0.18	0.19	0.19	4234	4156	4116

TCD	Chad	65575	Marba	0.18	0.19	0.19	38774	38059	37692
TCD	Chad	65576	Marfa	0.18	0.19	0.19	26842	26347	26092
TCD	Chad	65577	Maro	0.18	0.19	0.19	81394	79893	79122
TCD	Chad	65578	Massaguet	0.18	0.19	0.19	54999	53984	53464
TCD	Chad	65579	Massakory	0.18	0.19	0.19	148145	145413	144010
TCD	Chad	65580	Massalat-am-dam	0.18	0.19	0.19	9628	9451	9360
TCD	Chad	65581	Massalat-oum-hadjer	0.18	0.19	0.19	40376	39631	39249
TCD	Chad	65582	Massenya	0.18	0.19	0.19	39785	39051	38674
TCD	Chad	65583	Matekaga	0.18	0.19	0.19	6516	6396	6334
TCD	Chad	65584	Mayakne	0.18	0.19	0.19	3654	3587	3552
TCD	Chad	65585	Mbaikoro	0.18	0.19	0.19	36961	36280	35930
TCD	Chad	65586	Mbaissaye	0.18	0.19	0.19	19008	18658	18478
TCD	Chad	65587	Mbalkabra	0.18	0.19	0.19	18246	17909	17737
TCD	Chad	65588	Mballa	0.18	0.19	0.19	32311	31715	31409
TCD	Chad	65589	Mbaouroye	0.18	0.19	0.19	265	260	258
TCD	Chad	65590	Mbikou	0.18	0.19	0.19	6990	6861	6795
TCD	Chad	65591	Mbouroum	0.18	0.19	0.19	7895	7749	7675
TCD	Chad	65592	Medego	0.18	0.19	0.19	10029	9844	9749
TCD	Chad	65593	Melfi	0.18	0.19	0.19	20292	19918	19726
TCD	Chad	65594	Mesme	0.18	0.19	0.19	58277	57202	56650
TCD	Chad	65595	Mesmedje	0.18	0.19	0.19	6914	6787	6721
TCD	Chad	65596	Miandoum	0.18	0.19	0.19	18053	17720	17549
TCD	Chad	65597	Michemire	0.18	0.19	0.19	37103	36419	36067
TCD	Chad	65598	Miladi	0.18	0.19	0.19	8729	8568	8485
TCD	Chad	65599	Miltou-gourgara	0.18	0.19	0.19	46708	45847	45405
TCD	Chad	65600	Mimi	0.39	0.47	0.53	10927	8717	7239
TCD	Chad	65601	Mitau	0.18	0.19	0.19	18332	17994	17820
TCD	Chad	65602	Moal	0.18	0.19	0.19	9598	9421	9330
TCD	Chad	65603	Mogroum	0.18	0.19	0.19	22555	22139	21925
TCD	Chad	65604	Moissala	0.18	0.19	0.19	10955	10753	10649
TCD	Chad	65605	Moito	0.18	0.19	0.19	65250	64046	63429
TCD	Chad	65606	Mokofi	0.18	0.19	0.19	6226	6111	6052
TCD	Chad	65607	Molou	0.39	0.47	0.53	37390	29825	24768

TCD	Chad	65608	Mondo	0.18	0.19	0.19	18961	18612	18432
TCD	Chad	65609	Mont-de-lam	0.18	0.19	0.19	11694	11478	11368
TCD	Chad	65610	Moore	0.39	0.47	0.53	4937	3938	3270
TCD	Chad	65611	Motoa	0.18	0.19	0.19	6614	6492	6429
TCD	Chad	65612	Moubi-goz	0.18	0.19	0.19	13569	13319	13190
TCD	Chad	65613	Moubi-hadaba	0.18	0.19	0.19	9319	9147	9059
TCD	Chad	65614	Moubi-zarga	0.18	0.19	0.19	23113	22687	22468
TCD	Chad	65615	Moufa	0.18	0.19	0.19	6780	6655	6591
TCD	Chad	65616	Moundou/koutou	0.18	0.19	0.19	57922	56854	56306
TCD	Chad	65617	Mouroum-goulaye	0.18	0.19	0.19	12804	12568	12447
TCD	Chad	65618	Mouroum-touloum	0.18	0.19	0.19	4477	4394	4352
TCD	Chad	65619	Mourro	0.39	0.47	0.53	8746	6977	5794
TCD	Chad	65620	Mourzigui	0.18	0.19	0.19	12664	12430	12310
TCD	Chad	65621	Mousmare	0.18	0.19	0.19	6536	6415	6353
TCD	Chad	65622	Moussafoyo	0.18	0.19	0.19	14167	13906	13772
TCD	Chad	65623	Moussoro	0.18	0.19	0.19	116571	114421	113318
TCD	Chad	65624	Nadili	0.18	0.19	0.19	9017	8850	8765
TCD	Chad	65625	Nankesse	0.18	0.19	0.19	2566	2519	2495
TCD	Chad	65626	Nassian	0.18	0.19	0.19	10971	10768	10665
TCD	Chad	65627	Ndam	0.18	0.19	0.19	16325	16024	15870
TCD	Chad	65628	Nderguigui	0.18	0.19	0.19	15646	15357	15209
TCD	Chad	65629	Ndjamena	0.18	0.19	0.19	772438	758191	750878
TCD	Chad	65630	Ndjokou	0.18	0.19	0.19	21472	21076	20872
TCD	Chad	65631	Ngalo	0.18	0.19	0.19	18299	17962	17789
TCD	Chad	65632	Ngam	0.18	0.19	0.19	18182	17847	17675
TCD	Chad	65633	Ngamongo	0.18	0.19	0.19	2649	2600	2575
TCD	Chad	65634	Ngangara	0.18	0.19	0.19	7597	7457	7385
TCD	Chad	65635	Ngondong	0.18	0.19	0.19	18555	18213	18038
TCD	Chad	65636	Ngouri	0.18	0.19	0.19	169105	165986	164385
TCD	Chad	65637	Niellim	0.18	0.19	0.19	9426	9252	9163
TCD	Chad	65638	Ninga	0.18	0.19	0.19	7535	7396	7325
TCD	Chad	65639	Nokou	0.18	0.19	0.19	27866	27352	27088
TCD	Chad	65640	Ntiona	0.18	0.19	0.19	42633	41847	41443

TCD	Chad	65641	Ouadi-chok-abougouda	0.18	0.19	0.19	28512	27986	27716
TCD	Chad	65642	Ouadi-habile	0.39	0.47	0.53	8794	7015	5825
TCD	Chad	65643	Ouadi-hamra	0.18	0.19	0.19	28528	28001	27731
TCD	Chad	65644	Ouadi-kadja	0.39	0.47	0.53	8419	6716	5577
TCD	Chad	65645	Oudoumian	0.18	0.19	0.19	8868	8704	8620
TCD	Chad	65646	Ouled-djema	0.39	0.47	0.53	12453	9934	8249
TCD	Chad	65647	Ouled-himed	0.18	0.19	0.19	33166	32554	32240
TCD	Chad	65648	Ouled-rachid	0.18	0.19	0.19	64439	63251	62641
TCD	Chad	65649	Ouli-koure	0.39	0.47	0.53	6931	5529	4591
TCD	Chad	65650	Pandzangue	0.18	0.19	0.19	7197	7064	6996
TCD	Chad	65651	Pao	0.18	0.19	0.19	15060	14783	14640
TCD	Chad	65652	Peni-tchad	0.18	0.19	0.19	22032	21626	21417
TCD	Chad	65653	Rachid	0.18	0.19	0.19	23427	22995	22773
TCD	Chad	65654	Ratanine-har-djombo	0.18	0.19	0.19	55972	54939	54410
TCD	Chad	65655	Rig-rig	0.18	0.19	0.19	6471	6351	6290
TCD	Chad	65656	Saar	0.18	0.19	0.19	7888	7742	7668
TCD	Chad	65657	Salal	0.18	0.19	0.19	51915	50957	50466
TCD	Chad	65658	Salamat	0.18	0.19	0.19	33967	33341	33019
TCD	Chad	65659	Salamat-am-timan	0.18	0.19	0.19	33891	33266	32945
TCD	Chad	65660	Salamata	0.18	0.19	0.19	23553	23118	22895
TCD	Chad	65661	Sama	0.18	0.19	0.19	10965	10763	10659
TCD	Chad	65662	Sara-arabe	0.18	0.19	0.19	13323	13077	12951
TCD	Chad	65663	Sarh	0.18	0.19	0.19	90038	88377	87525
TCD	Chad	65664	Sedamis-mr-mn-assine	0.18	0.19	0.19	12462	12233	12115
TCD	Chad	65665	Signar	0.39	0.47	0.53	7846	6258	5197
TCD	Chad	65666	Simegotobe	0.18	0.19	0.19	3546	3481	3447
TCD	Chad	65667	Singako	0.18	0.19	0.19	18008	17676	17506
TCD	Chad	65668	Sorki	0.18	0.19	0.19	7804	7661	7587
TCD	Chad	65669	Soumraye	0.18	0.19	0.19	31565	30983	30684
TCD	Chad	65670	Sultanat	0.18	0.19	0.19	127381	125032	123826
TCD	Chad	65671	Tagal	0.18	0.19	0.19	51774	50819	50329
TCD	Chad	65672	Tagobo-foulbe	0.18	0.19	0.19	3972	3898	3861
TCD	Chad	65673	Tagoro	0.18	0.19	0.19	1726	1694	1678

TCD	Chad	65674	Takoua	0.18	0.19	0.19	7916	7770	7695
TCD	Chad	65675	Tamion-ngolo	0.18	0.19	0.19	9182	9013	8926
TCD	Chad	65676	Tapol	0.18	0.19	0.19	22554	22138	21924
TCD	Chad	65677	Tchaguine-ngolo	0.18	0.19	0.19	14224	13961	13827
TCD	Chad	65678	Tchaouen/saoua	0.18	0.19	0.19	2784	2732	2706
TCD	Chad	65679	Tchedoum	0.18	0.19	0.19	5732	5626	5572
TCD	Chad	65680	Tchoa	0.18	0.19	0.19	10346	10155	10057
TCD	Chad	65681	Teleme	0.18	0.19	0.19	3688	3620	3585
TCD	Chad	65682	Tibesti	0.18	0.19	0.19	16474	16170	16014
TCD	Chad	65683	Tikem	0.18	0.19	0.19	31301	30723	30427
TCD	Chad	65684	Timberi	0.18	0.19	0.19	20025	19656	19466
TCD	Chad	65685	Torom	0.18	0.19	0.19	5260	5163	5113
TCD	Chad	65686	Torrok	0.18	0.19	0.19	4154	4078	4038
TCD	Chad	65687	Tougoude	0.18	0.19	0.19	22956	22533	22316
TCD	Chad	65688	Toura	0.18	0.19	0.19	15940	15646	15495
TCD	Chad	65689	Troa	0.39	0.47	0.53	6235	4974	4130
TCD	Chad	65690	Troane	0.39	0.47	0.53	13542	10802	8970
TCD	Chad	65691	Yalnas-abou-deia	0.18	0.19	0.19	2824	2772	2745
TCD	Chad	65692	Yalnass	0.18	0.19	0.19	1283	1259	1247
TCD	Chad	65693	Yamodo	0.18	0.19	0.19	16733	16424	16266
TCD	Chad	65694	Yessie/ngama	0.18	0.19	0.19	35507	34852	34516
TCD	Chad	65695	Yomi	0.18	0.19	0.19	6077	5965	5907
TCD	Chad	65696	Youe	0.18	0.19	0.19	14134	13873	13740
TCD	Chad	65697	Zioud-amsak	0.18	0.19	0.19	11603	11389	11279
TGO	Togo	27386	Tchamba	0.79	0.81	0.83	1166	0	0
TGO	Togo	27387	Tchaoudjo	0.79	0.81	0.83	1740	0	0
TGO	Togo	27388	Assoli	0.77	0.79	0.80	1865	828	0
TGO	Togo	27390	Binah	0.77	0.79	0.80	2345	1042	0
TGO	Togo	27391	Doufelgou	0.77	0.79	0.80	2796	1242	0
TGO	Togo	27392	Keran	0.77	0.79	0.80	3255	1446	0
TGO	Togo	27393	Kozah	0.77	0.79	0.80	8015	3560	0
TGO	Togo	27394	Golfe	0.75	0.77	0.78	89472	56711	30328
TGO	Togo	27395	Lacs	0.75	0.77	0.78	15726	9968	5331

TGO	Togo	27396	Vo	0.75	0.77	0.78	12654	8020	4289
TGO	Togo	27397	Yoto	0.75	0.77	0.78	10086	6393	3419
TGO	Togo	27399	Amou	0.71	0.73	0.75	11229	8409	6268
TGO	Togo	27403	Wawa	0.71	0.73	0.75	16716	12519	9331
TGO	Togo	65272	Agou	0.71	0.73	0.75	8904	6668	4970
TGO	Togo	65273	Ave	0.75	0.77	0.78	5890	3733	1997
TGO	Togo	65274	Bassar	0.77	0.79	0.80	4165	1850	0
TGO	Togo	65275	Blitta	0.79	0.81	0.83	1207	0	0
TGO	Togo	65276	Dankpen	0.77	0.79	0.80	4422	1964	0
TGO	Togo	65277	Danyi	0.71	0.73	0.75	4046	3030	2258
TGO	Togo	65278	Est-Mono	0.71	0.73	0.75	12769	9562	7127
TGO	Togo	65279	Haho	0.71	0.73	0.75	26391	19764	14731
TGO	Togo	65280	Kloto	0.71	0.73	0.75	23295	17445	13003
TGO	Togo	65281	Kpendjal	0.75	0.77	0.78	9535	6155	3439
TGO	Togo	65282	Moyen-Mono	0.71	0.73	0.75	8109	6073	4527
TGO	Togo	65283	Ogou	0.71	0.73	0.75	34942	26167	19504
TGO	Togo	65284	Oti	0.75	0.77	0.78	11870	7662	4281
TGO	Togo	65285	Sotouboua	0.79	0.81	0.83	1411	0	0
TGO	Togo	65286	Tandjouare	0.75	0.77	0.78	7095	4580	2559
TGO	Togo	65287	Tone	0.75	0.77	0.78	22414	14469	8085
TGO	Togo	65288	Zio	0.75	0.77	0.78	20737	13144	7029
TTO	Trinidad and Tobago	27408	Admin unit not available	0.64	0.64	0.64	5253	5253	5253
TTO	Trinidad and Tobago	27409	Admin unit not available	0.64	0.64	0.64	14284	14284	14284
TTO	Trinidad and Tobago	27410	Admin unit not available	0.64	0.64	0.64	30284	30284	30284
TTO	Trinidad and Tobago	27411	Admin unit not available	0.64	0.64	0.64	17322	17322	17322
TTO	Trinidad and Tobago	27412	Admin unit not available	0.64	0.64	0.64	15694	15694	15694
TTO	Trinidad and Tobago	27413	Admin unit not available	0.64	0.64	0.64	3501	3501	3501
TTO	Trinidad and Tobago	27414	Admin unit not available	0.64	0.64	0.64	5465	5465	5465
TTO	Trinidad and Tobago	27415	Admin unit not available	0.64	0.64	0.64	16690	16690	16690
TTO	Trinidad and Tobago	27416	Admin unit not available	0.64	0.64	0.64	6109	6109	6109
TTO	Trinidad and Tobago	27417	Admin unit not available	0.64	0.64	0.64	7184	7184	7184
TTO	Trinidad and Tobago	27418	Admin unit not available	0.64	0.64	0.64	23935	23935	23935
TTO	Trinidad and Tobago	27419	Admin unit not available	0.64	0.64	0.64	12851	12851	12851

TTO	Trinidad and Tobago	27420	Admin unit not available	0.64	0.64	0.64	14371	14371	14371
TTO	Trinidad and Tobago	27421	Admin unit not available	0.64	0.64	0.64	10133	10133	10133
TTO	Trinidad and Tobago	27422	Admin unit not available	0.64	0.64	0.64	36765	36765	36765
TZA	United Republic of Tanzania	48494	Igunga	0.00	0.00	0.00	419142	419142	419142
TZA	United Republic of Tanzania	48492	Singida Rural	0.00	0.00	0.00	464433	464433	464433
TZA	United Republic of Tanzania	48431	Musoma Rural	0.00	0.00	0.00	442951	442951	442951
TZA	United Republic of Tanzania	48400	Makete	0.00	0.00	0.00	115324	115324	115324
TZA	United Republic of Tanzania	48419	Kilwa	0.00	0.00	0.00	169879	169879	169879
TZA	United Republic of Tanzania	48425	Babati	0.00	0.00	0.00	414030	414030	414030
TZA	United Republic of Tanzania	48486	Maswa	0.00	0.00	0.00	416629	416629	416629
TZA	United Republic of Tanzania	48443	Kilombero	0.00	0.00	0.00	377077	377077	377077
TZA	United Republic of Tanzania	48397	Iringa Urban	0.00	0.00	0.00	162517	162517	162517
TZA	United Republic of Tanzania	48441	Mbozi	0.00	0.00	0.00	647666	647666	647666
TZA	United Republic of Tanzania	48501	Kilindi	0.00	0.00	0.00	168594	168594	168594
TZA	United Republic of Tanzania	48436	Ileje	0.00	0.00	0.00	135031	135031	135031
TZA	United Republic of Tanzania	48386	Monduli	0.00	0.00	0.00	239624	239624	239624
TZA	United Republic of Tanzania	48487	Meatu	0.00	0.00	0.00	339517	339517	339517
TZA	United Republic of Tanzania	48402	Njombe	0.00	0.00	0.00	470057	470057	470057
TZA	United Republic of Tanzania	48422	Liwale	0.00	0.00	0.00	86029	86029	86029
TZA	United Republic of Tanzania	48424	Ruangwa	0.00	0.00	0.00	140674	140674	140674
TZA	United Republic of Tanzania	48417	Rombo	0.00	0.00	0.00	267104	267104	267104
TZA	United Republic of Tanzania	48435	Chunya	0.00	0.00	0.00	259792	259792	259792
TZA	United Republic of Tanzania	48394	Kongwa	0.00	0.00	0.00	286256	286256	286256
TZA	United Republic of Tanzania	48476	Sumbawanga Urban	0.00	0.00	0.00	209887	209887	209887
TZA	United Republic of Tanzania	48408	Ngara	0.00	0.00	0.00	403007	403007	403007
TZA	United Republic of Tanzania	48421	Lindi Urban	0.00	0.00	0.00	57064	57064	57064
TZA	United Republic of Tanzania	48459	Nyamagana	0.00	0.00	0.00	243552	243552	243552
TZA	United Republic of Tanzania	48411	Kigoma Rural	0.00	0.00	0.00	482041	482041	482041
TZA	United Republic of Tanzania	48429	Simanjiro	0.00	0.00	0.00	370939	370939	370939
TZA	United Republic of Tanzania	48401	Mufindi	0.00	0.00	0.00	304793	304793	304793
TZA	United Republic of Tanzania	48434	Tarime	0.00	0.00	0.00	663532	663532	663532
TZA	United Republic of Tanzania	48499	Uyui	0.00	0.00	0.00	343882	343882	343882
TZA	United Republic of Tanzania	48437	Kyela	0.00	0.00	0.00	211263	211263	211263

TZA	United Republic of Tanzania	48491	Manyoni	0.00	0.00	0.00	256821	256821	256821
TZA	United Republic of Tanzania	48451	Mtwara urban	0.00	0.00	0.00	121498	121498	121498
TZA	United Republic of Tanzania	48388	Ilala	0.00	0.00	0.00	799816	799816	799816
TZA	United Republic of Tanzania	48406	Karagwe	0.00	0.00	0.00	521464	521464	521464
TZA	United Republic of Tanzania	48423	Nachingwea	0.00	0.00	0.00	185452	185452	185452
TZA	United Republic of Tanzania	48455	Ilemela	0.00	0.00	0.00	376656	376656	376656
TZA	United Republic of Tanzania	48496	Sikonge	0.00	0.00	0.00	171482	171482	171482
TZA	United Republic of Tanzania	48407	Muleba	0.00	0.00	0.00	469021	469021	469021
TZA	United Republic of Tanzania	48420	Lindi Rural	0.00	0.00	0.00	238734	238734	238734
TZA	United Republic of Tanzania	48454	Geita	0.00	0.00	0.00	835068	835068	835068
TZA	United Republic of Tanzania	48384	Arusha	0.00	0.00	0.00	357694	357694	357694
TZA	United Republic of Tanzania	48432	Musoma Urban	0.00	0.00	0.00	169310	169310	169310
TZA	United Republic of Tanzania	48391	Dodoma Rural	0.00	0.00	0.00	491633	491633	491633
TZA	United Republic of Tanzania	48452	Newala	0.00	0.00	0.00	185388	185388	185388
TZA	United Republic of Tanzania	48439	Mbeya Rural	0.00	0.00	0.00	317328	317328	317328
TZA	United Republic of Tanzania	48502	Korogwe	0.00	0.00	0.00	308176	308176	308176
TZA	United Republic of Tanzania	48399	Ludewa	0.00	0.00	0.00	139836	139836	139836
TZA	United Republic of Tanzania	48457	Magu	0.00	0.00	0.00	495366	495366	495366
TZA	United Republic of Tanzania	48468	Kisarawe	0.00	0.00	0.00	107696	107696	107696
TZA	United Republic of Tanzania	48383	Arumeru	0.00	0.00	0.00	679217	679217	679217
TZA	United Republic of Tanzania	48416	Mwanga	0.00	0.00	0.00	133340	133340	133340
TZA	United Republic of Tanzania	48446	Morogoro Urban	0.00	0.00	0.00	318412	318412	318412
TZA	United Republic of Tanzania	48470	Mkuranga	0.00	0.00	0.00	207529	207529	207529
TZA	United Republic of Tanzania	48472	Admin unit not available	0.00	0.00	0.00	1137	1137	1137
TZA	United Republic of Tanzania	48488	Shinyanga Rural	0.00	0.00	0.00	373058	373058	373058
TZA	United Republic of Tanzania	48461	Ukerewe	0.00	0.00	0.00	311937	311937	311937
TZA	United Republic of Tanzania	48490	Iramba	0.00	0.00	0.00	436101	436101	436101
TZA	United Republic of Tanzania	48493	Singida Urban	0.00	0.00	0.00	175955	175955	175955
TZA	United Republic of Tanzania	48398	Kilolo	0.00	0.00	0.00	203782	203782	203782
TZA	United Republic of Tanzania	48449	Masasi	0.00	0.00	0.00	513336	513336	513336
TZA	United Republic of Tanzania	48477	Mbinga	0.00	0.00	0.00	478567	478567	478567
TZA	United Republic of Tanzania	48484	Kahama	0.00	0.00	0.00	818445	818445	818445
TZA	United Republic of Tanzania	48447	Mvomero	0.00	0.00	0.00	290381	290381	290381

TZA	United Republic of Tanzania	48498	Urambo	0.00	0.00	0.00	485694	485694	485694
TZA	United Republic of Tanzania	48442	Rungwe	0.00	0.00	0.00	380394	380394	380394
TZA	United Republic of Tanzania	48483	Bukombe	0.00	0.00	0.00	544446	544446	544446
TZA	United Republic of Tanzania	48385	Karatu	0.00	0.00	0.00	450646	450646	450646
TZA	United Republic of Tanzania	48404	Bukoba Rural	0.00	0.00	0.00	480694	480694	480694
TZA	United Republic of Tanzania	48474	Nkasi	0.00	0.00	0.00	281066	281066	281066
TZA	United Republic of Tanzania	48438	Mbarali	0.00	0.00	0.00	295135	295135	295135
TZA	United Republic of Tanzania	48430	Bunda	0.00	0.00	0.00	345324	345324	345324
TZA	United Republic of Tanzania	48467	Kibaha	0.00	0.00	0.00	169447	169447	169447
TZA	United Republic of Tanzania	48393	Kondoa	0.00	0.00	0.00	478635	478635	478635
TZA	United Republic of Tanzania	48473	Mpanda	0.00	0.00	0.00	532870	532870	532870
TZA	United Republic of Tanzania	48413	Hai	0.00	0.00	0.00	292891	292891	292891
TZA	United Republic of Tanzania	48410	Kibondo	0.00	0.00	0.00	413999	413999	413999
TZA	United Republic of Tanzania	48506	Tanga	0.00	0.00	0.00	304658	304658	304658
TZA	United Republic of Tanzania	48409	Kasulu	0.00	0.00	0.00	634365	634365	634365
TZA	United Republic of Tanzania	48478	Namtumbo	0.00	0.00	0.00	205962	205962	205962
TZA	United Republic of Tanzania	48396	Iringa Rural	0.00	0.00	0.00	257532	257532	257532
TZA	United Republic of Tanzania	48450	Mtwara Rural	0.00	0.00	0.00	214899	214899	214899
TZA	United Republic of Tanzania	48458	Missungwi	0.00	0.00	0.00	298651	298651	298651
TZA	United Republic of Tanzania	48475	Sumbawanga Rural	0.00	0.00	0.00	475918	475918	475918
TZA	United Republic of Tanzania	48412	Kigoma Urban	0.00	0.00	0.00	315365	315365	315365
TZA	United Republic of Tanzania	48403	Biharamulo	0.00	0.00	0.00	532995	532995	532995
TZA	United Republic of Tanzania	48387	Ngorongoro	0.00	0.00	0.00	161680	161680	161680
TZA	United Republic of Tanzania	48460	Sengerema	0.00	0.00	0.00	596956	596956	596956
TZA	United Republic of Tanzania	48389	Kinondoni	0.00	0.00	0.00	1408577	1408577	1408577
TZA	United Republic of Tanzania	48390	Temeke	0.00	0.00	0.00	971995	971995	971995
TZA	United Republic of Tanzania	48504	Muheza	0.00	0.00	0.00	321406	321406	321406
TZA	United Republic of Tanzania	48445	Morogoro Rural	0.00	0.00	0.00	286837	286837	286837
TZA	United Republic of Tanzania	48456	Kwimba	0.00	0.00	0.00	364811	364811	364811
TZA	United Republic of Tanzania	48448	Ulanga	0.00	0.00	0.00	220408	220408	220408
TZA	United Republic of Tanzania	48426	Hanang	0.00	0.00	0.00	275240	275240	275240
TZA	United Republic of Tanzania	48489	Shinyanga Urban	0.00	0.00	0.00	213381	213381	213381
TZA	United Republic of Tanzania	48405	Bukoba Urban	0.00	0.00	0.00	155036	155036	155036

TZA	United Republic of Tanzania	48427	Kiteto	0.00	0.00	0.00	202426	202426	202426
TZA	United Republic of Tanzania	48497	Tabora Urban	0.00	0.00	0.00	405273	405273	405273
TZA	United Republic of Tanzania	48440	Mbeya Urban	0.00	0.00	0.00	376599	376599	376599
TZA	United Republic of Tanzania	48444	Kilosa	0.00	0.00	0.00	584337	584337	584337
TZA	United Republic of Tanzania	48392	Dodoma Urban	0.00	0.00	0.00	508113	508113	508113
TZA	United Republic of Tanzania	48453	Tandahimba	0.00	0.00	0.00	228810	228810	228810
TZA	United Republic of Tanzania	48418	Same	0.00	0.00	0.00	246123	246123	246123
TZA	United Republic of Tanzania	48481	Tunduru	0.00	0.00	0.00	288698	288698	288698
TZA	United Republic of Tanzania	48479	Songea Rural	0.00	0.00	0.00	187890	187890	187890
TZA	United Republic of Tanzania	48428	Mbulu	0.00	0.00	0.00	320473	320473	320473
TZA	United Republic of Tanzania	48500	Handeni	0.00	0.00	0.00	281373	281373	281373
TZA	United Republic of Tanzania	48414	Moshi Rural	0.00	0.00	0.00	449340	449340	449340
TZA	United Republic of Tanzania	48433	Serengeti	0.00	0.00	0.00	225078	225078	225078
TZA	United Republic of Tanzania	48471	Rufiji	0.00	0.00	0.00	226298	226298	226298
TZA	United Republic of Tanzania	48482	Bariadi	0.00	0.00	0.00	826381	826381	826381
TZA	United Republic of Tanzania	48485	Kishapu	0.00	0.00	0.00	323919	323919	323919
TZA	United Republic of Tanzania	48466	Bagamoyo	0.00	0.00	0.00	265019	265019	265019
TZA	United Republic of Tanzania	48503	Lushoto	0.00	0.00	0.00	480597	480597	480597
TZA	United Republic of Tanzania	48495	Nzega	0.00	0.00	0.00	539133	539133	539133
TZA	United Republic of Tanzania	48480	Songea Urban	0.00	0.00	0.00	183856	183856	183856
TZA	United Republic of Tanzania	48395	Mpwapwa	0.00	0.00	0.00	300727	300727	300727
TZA	United Republic of Tanzania	48505	Pangani	0.00	0.00	0.00	48760	48760	48760
UGA	Uganda	28381	Adjumani	0.00	0.00	0.00	423722	423722	423722
UGA	Uganda	28382	Kole	0.00	0.00	0.00	175023	175023	175023
UGA	Uganda	28383	Kwania	0.00	0.00	0.00	124952	124952	124952
UGA	Uganda	28384	Maruzi	0.00	0.00	0.00	113104	113104	113104
UGA	Uganda	28385	Oyam	0.00	0.00	0.00	253090	253090	253090
UGA	Uganda	28386	Aringa	0.00	0.00	0.00	601873	601873	601873
UGA	Uganda	28387	Arua Municipality	0.00	0.00	0.00	44700	44700	44700
UGA	Uganda	28388	Ayivu	0.00	0.00	0.00	128108	128108	128108
UGA	Uganda	28389	Koboko	0.00	0.00	0.00	107413	107413	107413
UGA	Uganda	28390	Madi-okollo	0.00	0.00	0.00	71223	71223	71223
UGA	Uganda	28391	Maracha	0.00	0.00	0.00	108724	108724	108724

UGA	Uganda	28392	Terego	0.00	0.00	0.00	127788	127788	127788
UGA	Uganda	28393	Vurra	0.00	0.00	0.00	71541	71541	71541
UGA	Uganda	28394	Bukooli	0.00	0.00	0.00	744227	744227	744227
UGA	Uganda	28395	Bwamba	0.00	0.00	0.00	287691	287691	287691
UGA	Uganda	28396	Ntoroko	0.00	0.00	0.00	104241	104241	104241
UGA	Uganda	28397	Buhweju	0.00	0.00	0.00	117163	117163	117163
UGA	Uganda	28398	Bunyaruguru	0.00	0.00	0.00	139786	139786	139786
UGA	Uganda	28399	Igara	0.00	0.00	0.00	298417	298417	298417
UGA	Uganda	28400	Ruhinda	0.00	0.00	0.00	227491	227491	227491
UGA	Uganda	28401	Sheema	0.00	0.00	0.00	255994	255994	255994
UGA	Uganda	28402	Samia-bugwe	0.00	0.00	0.00	412502	412502	412502
UGA	Uganda	28403	Aswa	0.00	0.00	0.00	95180	95180	95180
UGA	Uganda	28404	Gulu Municipality	0.00	0.00	0.00	87052	87052	87052
UGA	Uganda	28405	Kilak	0.00	0.00	0.00	126080	126080	126080
UGA	Uganda	28406	Nwoya	0.00	0.00	0.00	39893	39893	39893
UGA	Uganda	28407	Omoro	0.00	0.00	0.00	119891	119891	119891
UGA	Uganda	28408	Bugahya	0.00	0.00	0.00	354691	354691	354691
UGA	Uganda	28409	Buhaguzi	0.00	0.00	0.00	268773	268773	268773
UGA	Uganda	28410	Bugweri	0.00	0.00	0.00	148680	148680	148680
UGA	Uganda	28411	Bunya	0.00	0.00	0.00	517973	517973	517973
UGA	Uganda	28412	Busiki	0.00	0.00	0.00	205955	205955	205955
UGA	Uganda	28413	Kigulu	0.00	0.00	0.00	311011	311011	311011
UGA	Uganda	28414	Luuka	0.00	0.00	0.00	227166	227166	227166
UGA	Uganda	28415	Butembe	0.00	0.00	0.00	233274	233274	233274
UGA	Uganda	28416	Jinja Municipality	0.00	0.00	0.00	121006	121006	121006
UGA	Uganda	28417	Kagoma	0.00	0.00	0.00	254160	254160	254160
UGA	Uganda	28419	Ndorwa	0.00	0.00	0.00	185951	185951	185951
UGA	Uganda	28420	Rubanda	0.00	0.00	0.00	220647	220647	220647
UGA	Uganda	28421	Rukiga	0.00	0.00	0.00	117037	117037	117037
UGA	Uganda	28422	Bunyangabu	0.00	0.00	0.00	169905	169905	169905
UGA	Uganda	28423	Burahya	0.00	0.00	0.00	257936	257936	257936
UGA	Uganda	28424	Fort Portal Municipality	0.00	0.00	0.00	62414	62414	62414
UGA	Uganda	28425	Kibale	0.00	0.00	0.00	225951	225951	225951

UGA	Uganda	28426	Kitagwenda	0.00	0.00	0.00	154433	154433	154433
UGA	Uganda	28427	Kyaka	0.00	0.00	0.00	181896	181896	181896
UGA	Uganda	28428	Mwenge	0.00	0.00	0.00	437406	437406	437406
UGA	Uganda	28429	Bujumba	0.00	0.00	0.00	37365	37365	37365
UGA	Uganda	28430	Kyamuswa	0.00	0.00	0.00	36382	36382	36382
UGA	Uganda	28432	Budiope	0.00	0.00	0.00	237433	237433	237433
UGA	Uganda	28433	Bugabula	0.00	0.00	0.00	287614	287614	287614
UGA	Uganda	28434	Bulamogi	0.00	0.00	0.00	192701	192701	192701
UGA	Uganda	28435	Buzaaya	0.00	0.00	0.00	163260	163260	163260
UGA	Uganda	28441	Amuria	0.00	0.00	0.00	79553	79553	79553
UGA	Uganda	28442	Kapelebyong	0.00	0.00	0.00	42924	42924	42924
UGA	Uganda	28443	Usuk	0.00	0.00	0.00	82562	82562	82562
UGA	Uganda	28444	Bugangaizi	0.00	0.00	0.00	203795	203795	203795
UGA	Uganda	28445	Buyaga	0.00	0.00	0.00	432721	432721	432721
UGA	Uganda	28446	Buyanja	0.00	0.00	0.00	130447	130447	130447
UGA	Uganda	28447	Kiboga	0.00	0.00	0.00	397530	397530	397530
UGA	Uganda	28448	Bufumbira	0.00	0.00	0.00	271245	271245	271245
UGA	Uganda	28449	Agago	0.52	0.52	0.52	120505	120505	120505
UGA	Uganda	28450	Aruu	0.52	0.52	0.52	91203	91203	91203
UGA	Uganda	28451	Chua	0.52	0.52	0.52	100912	100912	100912
UGA	Uganda	28452	Lamwo	0.52	0.52	0.52	71076	71076	71076
UGA	Uganda	28453	Dodoth	0.00	0.00	0.00	170013	170013	170013
UGA	Uganda	28454	Jie	0.00	0.00	0.00	65749	65749	65749
UGA	Uganda	28455	Labwor	0.52	0.52	0.52	8523	8523	8523
UGA	Uganda	28456	Bukedea	0.00	0.00	0.00	147069	147069	147069
UGA	Uganda	28457	Kumi	0.00	0.00	0.00	201972	201972	201972
UGA	Uganda	28458	Ngora	0.00	0.00	0.00	123793	123793	123793
UGA	Uganda	28459	Dokolo	0.00	0.00	0.00	152438	152438	152438
UGA	Uganda	28460	Erute	0.00	0.00	0.00	237869	237869	237869
UGA	Uganda	28461	Kyoga	0.00	0.00	0.00	98096	98096	98096
UGA	Uganda	28462	Lira Municipality	0.00	0.00	0.00	88723	88723	88723
UGA	Uganda	28463	Moroto	0.00	0.00	0.00	187313	187313	187313
UGA	Uganda	28464	Otuke	0.00	0.00	0.00	61468	61468	61468

UGA	Uganda	28465	Katikamu	0.00	0.00	0.00	231658	231658	231658
UGA	Uganda	28466	Nakaseke	0.00	0.00	0.00	142500	142500	142500
UGA	Uganda	28467	Wabusana (bamunanika)	0.00	0.00	0.00	143270	143270	143270
UGA	Uganda	28468	Bukomansimbi	0.00	0.00	0.00	176526	176526	176526
UGA	Uganda	28469	Bukoto	0.00	0.00	0.00	518896	518896	518896
UGA	Uganda	28470	Kalungu	0.00	0.00	0.00	204505	204505	204505
UGA	Uganda	28471	Masaka Municipality	0.00	0.00	0.00	115576	115576	115576
UGA	Uganda	28472	Bujenje	0.00	0.00	0.00	127662	127662	127662
UGA	Uganda	28473	Buliisa	0.00	0.00	0.00	108123	108123	108123
UGA	Uganda	28475	Kibanda	0.00	0.00	0.00	306123	306123	306123
UGA	Uganda	28476	Bubulo	0.00	0.00	0.00	178409	178409	178409
UGA	Uganda	28477	Budadiri	0.00	0.00	0.00	276281	276281	276281
UGA	Uganda	28478	Bulambuli	0.00	0.00	0.00	147551	147551	147551
UGA	Uganda	28479	Bungokho	0.00	0.00	0.00	191988	191988	191988
UGA	Uganda	28480	Manjiya	0.00	0.00	0.00	85695	85695	85695
UGA	Uganda	28481	Mbale Municipality	0.00	0.00	0.00	59792	59792	59792
UGA	Uganda	28482	Bukanga	0.00	0.00	0.00	49955	49955	49955
UGA	Uganda	28483	Ibanda	0.00	0.00	0.00	94510	94510	94510
UGA	Uganda	28484	Isingiro	0.00	0.00	0.00	100699	100699	100699
UGA	Uganda	28485	Kashari	0.00	0.00	0.00	81159	81159	81159
UGA	Uganda	28486	Kazo	0.00	0.00	0.00	48759	48759	48759
UGA	Uganda	28488	Nyabushozi	0.00	0.00	0.00	48908	48908	48908
UGA	Uganda	28489	Rwampara	0.00	0.00	0.00	65964	65964	65964
UGA	Uganda	28490	Bokora	0.00	0.00	0.00	215804	215804	215804
UGA	Uganda	28491	Kadani (chekwii)	0.00	0.00	0.00	101753	101753	101753
UGA	Uganda	28492	Matheniko	0.00	0.00	0.00	146842	146842	146842
UGA	Uganda	28493	Moroto Municipality	0.00	0.00	0.00	10795	10795	10795
UGA	Uganda	28494	Pian	0.00	0.00	0.00	81684	81684	81684
UGA	Uganda	28495	Upe	0.00	0.00	0.00	126960	126960	126960
UGA	Uganda	28496	Obongi	0.00	0.00	0.00	205282	205282	205282
UGA	Uganda	28497	West Moyo	0.00	0.00	0.00	248424	248424	248424
UGA	Uganda	28499	Butambala	0.00	0.00	0.00	114142	114142	114142
UGA	Uganda	28501	Gomba	0.00	0.00	0.00	177705	177705	177705

UGA	Uganda	28503	Mawokota	0.00	0.00	0.00	254451	254451	254451
UGA	Uganda	28504	Busujju	0.00	0.00	0.00	77780	77780	77780
UGA	Uganda	28505	Buwekula	0.00	0.00	0.00	230459	230459	230459
UGA	Uganda	28506	Kassanda	0.00	0.00	0.00	196973	196973	196973
UGA	Uganda	28507	Mityana	0.00	0.00	0.00	203148	203148	203148
UGA	Uganda	28508	Bbaale	0.00	0.00	0.00	154078	154078	154078
UGA	Uganda	28509	Buvuma Islands	0.00	0.00	0.00	62459	62459	62459
UGA	Uganda	28510	Buyikwe	0.00	0.00	0.00	510224	510224	510224
UGA	Uganda	28511	Mukono	0.00	0.00	0.00	416591	416591	416591
UGA	Uganda	28512	Nakifuma	0.00	0.00	0.00	241985	241985	241985
UGA	Uganda	28513	Ntenjeru	0.00	0.00	0.00	263313	263313	263313
UGA	Uganda	28514	Jonam	0.00	0.00	0.00	147981	147981	147981
UGA	Uganda	28515	Okoro	0.00	0.00	0.00	258096	258096	258096
UGA	Uganda	28516	Padyere	0.00	0.00	0.00	240861	240861	240861
UGA	Uganda	28517	Kajara	0.00	0.00	0.00	138314	138314	138314
UGA	Uganda	28518	Ruhaama	0.00	0.00	0.00	281667	281667	281667
UGA	Uganda	28519	Rushenyi	0.00	0.00	0.00	139040	139040	139040
UGA	Uganda	28520	Budaka	0.00	0.00	0.00	162605	162605	162605
UGA	Uganda	28521	Butebo	0.00	0.00	0.00	114399	114399	114399
UGA	Uganda	28522	Kibuku	0.00	0.00	0.00	151773	151773	151773
UGA	Uganda	28523	Pallisa	0.00	0.00	0.00	195201	195201	195201
UGA	Uganda	28524	Kabula	0.00	0.00	0.00	82364	82364	82364
UGA	Uganda	28525	Kakuuto	0.00	0.00	0.00	104689	104689	104689
UGA	Uganda	28526	Kooki	0.00	0.00	0.00	203045	203045	203045
UGA	Uganda	28527	Kyotera	0.00	0.00	0.00	171290	171290	171290
UGA	Uganda	28528	Kinkiizi	0.00	0.00	0.00	291424	291424	291424
UGA	Uganda	28529	Rubabo	0.00	0.00	0.00	161024	161024	161024
UGA	Uganda	28530	Rujumbura	0.00	0.00	0.00	215432	215432	215432
UGA	Uganda	28531	Lwemiyaga	0.00	0.00	0.00	43683	43683	43683
UGA	Uganda	28532	Mawogola	0.00	0.00	0.00	211147	211147	211147
UGA	Uganda	28533	Kaberamaido	0.00	0.00	0.00	107693	107693	107693
UGA	Uganda	28534	Kalaki	0.00	0.00	0.00	119425	119425	119425
UGA	Uganda	28535	Kasilo	0.00	0.00	0.00	130735	130735	130735

UGA	Uganda	28536	Serere	0.00	0.00	0.00	193533	193533	193533
UGA	Uganda	28537	Soroti	0.00	0.00	0.00	291723	291723	291723
UGA	Uganda	28538	Soroti Municipality	0.00	0.00	0.00	78772	78772	78772
UGA	Uganda	28539	Bunyole	0.00	0.00	0.00	161258	161258	161258
UGA	Uganda	28540	Kisoko (west Budama)	0.00	0.00	0.00	216997	216997	216997
UGA	Uganda	28541	Tororo	0.00	0.00	0.00	147748	147748	147748
UGA	Uganda	28542	Tororo Municipality	0.00	0.00	0.00	45370	45370	45370
UGA	Uganda	42202	Central Kampala	0.00	0.00	0.00	192352	192352	192352
UGA	Uganda	42203	Kongasis	0.00	0.00	0.00	55108	55108	55108
UGA	Uganda	42204	Kween	0.00	0.00	0.00	98761	98761	98761
UGA	Uganda	42205	Tingey	0.00	0.00	0.00	98264	98264	98264
UGA	Uganda	42206	Bukonjo	0.00	0.00	0.00	401863	401863	401863
UGA	Uganda	42207	Busongora	0.00	0.00	0.00	455026	455026	455026
UGA	Uganda	42208	Buruli M	0.00	0.00	0.00	218318	218318	218318
UGA	Uganda	42209	Buruli	0.00	0.00	0.00	182240	182240	182240
UGA	Uganda	42210	Busiro	0.00	0.00	0.00	606952	606952	606952
UGA	Uganda	42211	Entebbe Municipality	0.00	0.00	0.00	112227	112227	112227
UGA	Uganda	42212	Kyadondo	0.00	0.00	0.00	1520993	1520993	1520993
UGA	Uganda	42213	Kawempe	0.00	0.00	0.00	492375	492375	492375
UGA	Uganda	42214	Makerere University	0.00	0.00	0.00	21783	21783	21783
UGA	Uganda	42215	Nakawa	0.00	0.00	0.00	455481	455481	455481
UGA	Uganda	42216	Rubaga	0.00	0.00	0.00	510336	510336	510336
VEN	Venezuela	31856	D. Atabapo	0.89	0.91	0.92	0	0	0
VEN	Venezuela	31857	D. Atures	0.89	0.91	0.92	0	0	0
VEN	Venezuela	31858	D. Casiquiare	0.89	0.91	0.92	0	0	0
VEN	Venezuela	31859	D. Rio Negro	0.89	0.91	0.92	0	0	0
VEN	Venezuela	31860	Anaco	0.41	0.46	0.49	49299	42767	38824
VEN	Venezuela	31861	Aragua	0.41	0.46	0.49	19877	17243	15654
VEN	Venezuela	31862	Bolivar	0.41	0.46	0.49	184732	160255	145481
VEN	Venezuela	31863	Bruzual	0.41	0.46	0.49	13506	11717	10637
VEN	Venezuela	31864	Cajigal	0.41	0.46	0.49	5559	4823	4378
VEN	Venezuela	31865	Carvajal	0.41	0.46	0.49	5963	5173	4696
VEN	Venezuela	31866	Freites	0.41	0.46	0.49	31548	27368	24845

VEN	Venezuela	31867	Guanipa	0.41	0.46	0.49	31491	27319	24800
VEN	Venezuela	31868	Guanta	0.41	0.46	0.49	13216	11465	10408
VEN	Venezuela	31869	I. De Adentro	0.41	0.46	0.49	40	34	31
VEN	Venezuela	31870	Independencia	0.41	0.46	0.49	29389	25495	23145
VEN	Venezuela	31871	Isla	0.41	0.46	0.49	130	113	103
VEN	Venezuela	31872	Libertad	0.41	0.46	0.49	7254	6293	5713
VEN	Venezuela	31873	Miranda	0.41	0.46	0.49	18508	16056	14576
VEN	Venezuela	31874	Monagas	0.41	0.46	0.49	7235	6276	5698
VEN	Venezuela	31875	Penalver	0.41	0.46	0.49	17976	15594	14156
VEN	Venezuela	31876	Piritu	0.41	0.46	0.49	9457	8204	7447
VEN	Venezuela	31877	Simon Rodriguez	0.41	0.46	0.49	73336	63619	57754
VEN	Venezuela	31878	Sotillo	0.41	0.46	0.49	94610	82074	74507
VEN	Venezuela	31879	Autonomo Achaguas	0.41	0.46	0.49	23625	20494	18605
VEN	Venezuela	31880	Autonomo Biruaca	0.41	0.46	0.49	22448	19474	17678
VEN	Venezuela	31881	Autonomo Munoz	0.41	0.46	0.49	10717	9297	8440
VEN	Venezuela	31882	Autonomo Paez	0.38	0.72	0.92	43211	7765	0
VEN	Venezuela	31883	Autonomo Pedro Camejo	0.41	0.46	0.49	12405	10761	9769
VEN	Venezuela	31884	Autonomo Romulo Gallegos	0.41	0.46	0.49	10962	9510	8633
VEN	Venezuela	31885	Autonomo San Fernando	0.41	0.46	0.49	68339	59284	53818
VEN	Venezuela	31886	Bolivar	0.41	0.46	0.49	14001	12146	11026
VEN	Venezuela	31887	Camatagua	0.41	0.46	0.49	6752	5858	5318
VEN	Venezuela	31888	Girardot	0.41	0.46	0.49	153967	133567	121253
VEN	Venezuela	31889	Jose Angel Lamas	0.41	0.46	0.49	12914	11203	10170
VEN	Venezuela	31890	Jose Felix Ribas	0.41	0.46	0.49	75850	65800	59734
VEN	Venezuela	31891	Libertador	0.41	0.46	0.49	16692	14481	13146
VEN	Venezuela	31892	Mario Briceno Iragorry	0.41	0.46	0.49	46319	40182	36478
VEN	Venezuela	31893	San Casimiro	0.41	0.46	0.49	10840	9404	8537
VEN	Venezuela	31894	San Sebastian	0.41	0.46	0.49	10134	8791	7980
VEN	Venezuela	31895	Santiago Marino	0.41	0.46	0.49	134635	116796	106029
VEN	Venezuela	31896	Santos Michelena	0.41	0.46	0.49	15344	13311	12084
VEN	Venezuela	31897	Sucre	0.41	0.46	0.49	43054	37350	33906
VEN	Venezuela	31898	Tovar	0.41	0.46	0.49	7215	6259	5682
VEN	Venezuela	31899	Urdaneta	0.41	0.46	0.49	8748	7589	6889

VEN	Venezuela	31900	Zamora	0.41	0.46	0.49	59366	51500	46752
VEN	Venezuela	31901	Alberto Arvelo Torrealba	0.41	0.46	0.49	16803	14576	13233
VEN	Venezuela	31902	Antonio Jose De Sucre	0.41	0.46	0.49	34545	29968	27205
VEN	Venezuela	31903	Arismendi	0.41	0.46	0.49	10184	8834	8020
VEN	Venezuela	31904	Barinas	0.41	0.46	0.49	150036	130157	118157
VEN	Venezuela	31905	Bolivar	0.38	0.72	0.92	24103	4331	0
VEN	Venezuela	31906	Cruz Paredes	0.41	0.46	0.49	11165	9686	8793
VEN	Venezuela	31907	Ezequiel Zamora	0.41	0.46	0.49	29200	25331	22996
VEN	Venezuela	31908	Obispos	0.41	0.46	0.49	17267	14980	13599
VEN	Venezuela	31909	Pedraza	0.41	0.46	0.49	28604	24814	22527
VEN	Venezuela	31910	Rojas	0.41	0.46	0.49	16457	14277	12961
VEN	Venezuela	31911	Sosa	0.41	0.46	0.49	9940	8623	7828
VEN	Venezuela	31912	Caroni	0.41	0.46	0.49	253106	219570	199327
VEN	Venezuela	31913	Cedeno	0.41	0.46	0.49	28956	25120	22804
VEN	Venezuela	31914	El Callao	0.41	0.46	0.49	8919	7737	7024
VEN	Venezuela	31915	Gran Sabana	0.41	0.46	0.49	17022	14766	13405
VEN	Venezuela	31916	Heres	0.41	0.46	0.49	138861	120462	109356
VEN	Venezuela	31917	Piar	0.41	0.46	0.49	47196	40943	37168
VEN	Venezuela	31918	Raul Leoni	0.41	0.46	0.49	18512	16059	14579
VEN	Venezuela	31919	Roscio	0.41	0.46	0.49	8882	7705	6995
VEN	Venezuela	31920	Sifontes	0.41	0.46	0.49	23940	20768	18854
VEN	Venezuela	31921	Sucre	0.41	0.46	0.49	9383	8139	7389
VEN	Venezuela	31922	Bejuma	0.41	0.46	0.49	21752	18870	17130
VEN	Venezuela	31923	Carlos Arevalo	0.41	0.46	0.49	64369	55841	50693
VEN	Venezuela	31924	Diego Ibarra	0.41	0.46	0.49	40332	34988	31763
VEN	Venezuela	31925	Guacara	0.41	0.46	0.49	72170	62608	56836
VEN	Venezuela	31926	Isla	0.41	0.46	0.49	68	59	54
VEN	Venezuela	31927	Juan Jose Mora	0.41	0.46	0.49	28410	24645	22373
VEN	Venezuela	31928	Lago De Valencia	0.41	0.46	0.49	2678	2323	2109
VEN	Venezuela	31929	Miranda	0.41	0.46	0.49	11888	10313	9362
VEN	Venezuela	31930	Montalban	0.41	0.46	0.49	73646	63888	57998
VEN	Venezuela	31931	Puerto Cabello	0.41	0.46	0.49	70433	61101	55468
VEN	Venezuela	31932	San Joaquin	0.41	0.46	0.49	27693	24024	21809

VEN	Venezuela	31933	Valencia	0.41	0.46	0.49	510229	442624	401818
VEN	Venezuela	31934	Anzoategui	0.41	0.46	0.49	7731	6707	6089
VEN	Venezuela	31935	Falcon	0.41	0.46	0.49	42101	36523	33156
VEN	Venezuela	31936	Girardot	0.41	0.46	0.49	4930	4277	3882
VEN	Venezuela	31937	Pao De San Juan Bautista	0.41	0.46	0.49	9773	8478	7696
VEN	Venezuela	31938	Ricaurte	0.41	0.46	0.49	5593	4852	4405
VEN	Venezuela	31939	San Carlos	0.41	0.46	0.49	52246	45323	41145
VEN	Venezuela	31940	Tinaco	0.41	0.46	0.49	17549	15224	13820
VEN	Venezuela	31941	D. Antonio Diaz	0.41	0.46	0.49	27044	23461	21298
VEN	Venezuela	31942	D. Pedernales	0.41	0.46	0.49	3868	3356	3046
VEN	Venezuela	31943	Tucupita	0.41	0.46	0.49	57991	50307	45669
VEN	Venezuela	31959	Libertador	0.41	0.46	0.49	2000773	1735674	1575659
VEN	Venezuela	31960	Vargas	0.41	0.46	0.49	183448	159141	144470
VEN	Venezuela	31961	Acosta	0.21	0.21	0.21	11197	11197	11197
VEN	Venezuela	31962	Bolivar	0.21	0.21	0.21	8507	8507	8507
VEN	Venezuela	31963	Buchivacoa	0.21	0.21	0.21	13925	13925	13925
VEN	Venezuela	31964	Cacique Manaure	0.21	0.21	0.21	6873	6873	6873
VEN	Venezuela	31965	Carirubana	0.21	0.21	0.21	138641	138641	138641
VEN	Venezuela	31966	Colina	0.21	0.21	0.21	25649	25649	25649
VEN	Venezuela	31967	Dabajuro	0.21	0.21	0.21	14707	14707	14707
VEN	Venezuela	31968	Democracia	0.21	0.21	0.21	11659	11659	11659
VEN	Venezuela	31969	Falcon	0.21	0.21	0.21	29642	29642	29642
VEN	Venezuela	31970	Federacion	0.21	0.21	0.21	17208	17208	17208
VEN	Venezuela	31972	Jacura	0.21	0.21	0.21	6569	6569	6569
VEN	Venezuela	31973	Los Tanques	0.21	0.21	0.21	26543	26543	26543
VEN	Venezuela	31974	Mauroa	0.41	0.46	0.49	10379	9004	8174
VEN	Venezuela	31975	Miranda	0.21	0.21	0.21	127917	127917	127917
VEN	Venezuela	31976	Monsenor Iturriza	0.41	0.46	0.49	7631	6620	6010
VEN	Venezuela	31977	Palma Sola	0.21	0.21	0.21	4169	4169	4169
VEN	Venezuela	31978	Petit	0.21	0.21	0.21	8524	8524	8524
VEN	Venezuela	31979	Piritu	0.21	0.21	0.21	7125	7125	7125
VEN	Venezuela	31980	San Francisco	0.21	0.21	0.21	6398	6398	6398
VEN	Venezuela	31981	Silva	0.41	0.46	0.49	12463	10812	9815

VEN	Venezuela	31982	Union	0.21	0.21	0.21	9096	9096	9096
VEN	Venezuela	31983	Zamora	0.21	0.21	0.21	22266	22266	22266
VEN	Venezuela	31984	Camaguan	0.41	0.46	0.49	18497	16046	14567
VEN	Venezuela	31985	Chaguaramas	0.41	0.46	0.49	4978	4318	3920
VEN	Venezuela	31986	El Socorro	0.41	0.46	0.49	6914	5998	5445
VEN	Venezuela	31987	Guacharamas	0.41	0.46	0.49	503	436	396
VEN	Venezuela	31988	Infante	0.41	0.46	0.49	47994	41635	37797
VEN	Venezuela	31989	Las Mercedes	0.41	0.46	0.49	14343	12443	11296
VEN	Venezuela	31990	Mellado	0.41	0.46	0.49	11281	9787	8884
VEN	Venezuela	31991	Miranda	0.41	0.46	0.49	57962	50282	45646
VEN	Venezuela	31992	Monagas	0.41	0.46	0.49	30132	26139	23730
VEN	Venezuela	31993	Ortiz	0.41	0.46	0.49	11209	9724	8828
VEN	Venezuela	31994	Ribas	0.41	0.46	0.49	15052	13057	11853
VEN	Venezuela	31995	Roscio	0.41	0.46	0.49	51745	44889	40750
VEN	Venezuela	31996	San Jose De Guaribe	0.41	0.46	0.49	4723	4097	3720
VEN	Venezuela	31997	Santa Maria De Ipire	0.41	0.46	0.49	5288	4587	4164
VEN	Venezuela	31998	Zaraza	0.41	0.46	0.49	24677	21407	19434
VEN	Venezuela	31999	Andres Eloy Blanco	0.41	0.46	0.49	19301	16743	15200
VEN	Venezuela	32000	Crespo	0.41	0.46	0.49	21187	18380	16685
VEN	Venezuela	32001	Iribarren	0.41	0.46	0.49	392095	340143	308785
VEN	Venezuela	32002	Jimenez	0.41	0.46	0.49	42333	36724	33338
VEN	Venezuela	32003	Moran	0.41	0.46	0.49	49059	42558	38635
VEN	Venezuela	32004	Palavecino	0.41	0.46	0.49	71958	62424	56669
VEN	Venezuela	32005	Simon Planas	0.41	0.46	0.49	16072	13942	12657
VEN	Venezuela	32006	Torres	0.21	0.21	0.21	111129	111129	111129
VEN	Venezuela	32007	Urdaneta	0.21	0.21	0.21	37849	37849	37849
VEN	Venezuela	32008	Alberto Adriani	0.41	0.54	0.62	54016	35136	24119
VEN	Venezuela	32009	Andres Bello	0.41	0.54	0.62	6305	4101	2815
VEN	Venezuela	32010	Antonio Pinto Salinas	0.41	0.54	0.62	9696	6307	4329
VEN	Venezuela	32011	Aricagua	0.41	0.54	0.62	2037	1325	910
VEN	Venezuela	32012	Arzobispo Chacon	0.41	0.54	0.62	5167	3361	2307
VEN	Venezuela	32013	Campo Elias	0.41	0.54	0.62	40704	26478	18176
VEN	Venezuela	32014	Caracciolo Parra Olmedo	0.41	0.54	0.62	11795	7673	5267

VEN	Venezuela	32015	Cardenal Quintero	0.41	0.54	0.62	4019	2614	1795
VEN	Venezuela	32016	Guaraque	0.41	0.54	0.62	3845	2501	1717
VEN	Venezuela	32017	Julio Cesar Salas	0.41	0.54	0.62	5912	3846	2640
VEN	Venezuela	32018	Justo Briceno	0.23	0.34	0.38	3178	2546	2343
VEN	Venezuela	32019	Libertador	0.41	0.54	0.62	83372	54232	37228
VEN	Venezuela	32020	Miranda	0.23	0.34	0.38	12905	10337	9512
VEN	Venezuela	32021	Obispo Ramos De Lora	0.41	0.54	0.62	10006	6509	4468
VEN	Venezuela	32022	Padre Noguera	0.38	0.78	0.96	1420	64	0
VEN	Venezuela	32023	Pueblo Llano	0.41	0.54	0.62	4160	2706	1858
VEN	Venezuela	32024	Rangel	0.41	0.54	0.62	8091	5263	3613
VEN	Venezuela	32025	Rivas Davila	0.41	0.54	0.62	8195	5331	3659
VEN	Venezuela	32026	Santos Marquina	0.41	0.54	0.62	7837	5098	3500
VEN	Venezuela	32027	Sucre	0.41	0.54	0.62	23269	15136	10390
VEN	Venezuela	32028	Tovar	0.41	0.54	0.62	15060	9796	6725
VEN	Venezuela	32029	Tulio Febres Cordero	0.41	0.54	0.62	13761	8951	6145
VEN	Venezuela	32030	Zea	0.41	0.54	0.62	4637	3016	2070
VEN	Venezuela	32031	Acevedo	0.41	0.46	0.49	38134	33081	30031
VEN	Venezuela	32032	Andres Bello	0.41	0.46	0.49	7836	6798	6171
VEN	Venezuela	32033	Baruta	0.41	0.46	0.49	91764	79605	72267
VEN	Venezuela	32034	Brion	0.41	0.46	0.49	24692	21420	19445
VEN	Venezuela	32035	Buroz	0.41	0.46	0.49	11848	10278	9331
VEN	Venezuela	32036	Carrizal	0.41	0.46	0.49	22953	19912	18076
VEN	Venezuela	32037	Chacao	0.41	0.46	0.49	27416	23784	21591
VEN	Venezuela	32038	Cristobal Rojas	0.41	0.46	0.49	51224	44437	40340
VEN	Venezuela	32039	El Hatillo	0.41	0.46	0.49	27225	23618	21441
VEN	Venezuela	32040	Guaicaipuro	0.41	0.46	0.49	119868	103985	94399
VEN	Venezuela	32041	Independencia	0.41	0.46	0.49	53422	46343	42071
VEN	Venezuela	32042	Isla	0.41	0.46	0.49	247	214	194
VEN	Venezuela	32043	Lander	0.41	0.46	0.49	61456	53314	48398
VEN	Venezuela	32044	Los Salias	0.41	0.46	0.49	35035	30393	27591
VEN	Venezuela	32045	Paez	0.41	0.46	0.49	15315	13286	12061
VEN	Venezuela	32046	Paz Castillo	0.41	0.46	0.49	49995	43370	39372
VEN	Venezuela	32047	Pedro Gual	0.41	0.46	0.49	9046	7847	7124

VEN	Venezuela	32048	Plaza	0.41	0.46	0.49	84216	73058	66322
VEN	Venezuela	32049	Simon Bolivar	0.41	0.46	0.49	18722	16241	14744
VEN	Venezuela	32050	Sucre	0.41	0.46	0.49	228855	198532	180229
VEN	Venezuela	32051	Urdaneta	0.41	0.46	0.49	56540	49049	44527
VEN	Venezuela	32052	Zamora	0.41	0.46	0.49	76640	66485	60356
VEN	Venezuela	32053	Acosta	0.41	0.46	0.49	7170	6220	5646
VEN	Venezuela	32054	Bolivar	0.41	0.46	0.49	13879	12040	10930
VEN	Venezuela	32055	Caripe	0.41	0.46	0.49	13278	11519	10457
VEN	Venezuela	32056	Cedeno	0.41	0.46	0.49	14711	12762	11585
VEN	Venezuela	32057	D. Pedernales	0.41	0.46	0.49	5	5	4
VEN	Venezuela	32058	Ezequiel Zamora	0.41	0.46	0.49	24901	21602	19610
VEN	Venezuela	32059	Libertador	0.41	0.46	0.49	18786	16297	14794
VEN	Venezuela	32060	Maturin	0.41	0.46	0.49	239632	207881	188717
VEN	Venezuela	32061	Piar	0.41	0.46	0.49	18915	16409	14896
VEN	Venezuela	32062	Punceres	0.41	0.46	0.49	11226	9739	8841
VEN	Venezuela	32063	Sotillo	0.41	0.46	0.49	18642	16172	14681
VEN	Venezuela	32069	Name Unknown	0.41	0.46	0.49	365	316	287
VEN	Venezuela	32070	Antolin Del Cami	0.41	0.46	0.49	10957	9506	8629
VEN	Venezuela	32071	Arismendi	0.41	0.46	0.49	12365	10727	9738
VEN	Venezuela	32072	Diaz	0.41	0.46	0.49	33049	28670	26027
VEN	Venezuela	32073	Garcia	0.41	0.46	0.49	32080	27830	25264
VEN	Venezuela	32074	Gomez	0.41	0.46	0.49	16912	14671	13319
VEN	Venezuela	32075	Isla	0.41	0.46	0.49	1	1	1
VEN	Venezuela	32076	Isla De Coche	0.41	0.46	0.49	3241	2812	2552
VEN	Venezuela	32077	Isla De Cubagua	0.41	0.46	0.49	13282	11522	10460
VEN	Venezuela	32078	Isla La Tortuga	0.41	0.46	0.49	631	547	497
VEN	Venezuela	32079	Maneiro	0.41	0.46	0.49	20084	17423	15817
VEN	Venezuela	32080	Marcano	0.41	0.46	0.49	12777	11084	10062
VEN	Venezuela	32081	Marino	0.41	0.46	0.49	35549	30839	27995
VEN	Venezuela	32082	Peninsula De Macanao	0.41	0.46	0.49	11178	9697	8803
VEN	Venezuela	32083	Agua Blanca	0.41	0.46	0.49	8815	7647	6942
VEN	Venezuela	32084	Araure	0.41	0.46	0.49	64512	55965	50805
VEN	Venezuela	32085	Esteller	0.41	0.46	0.49	18312	15886	14421

VEN	Venezuela	32086	Guanare	0.41	0.46	0.49	78783	68344	62043
VEN	Venezuela	32087	Guanarito	0.41	0.46	0.49	15288	13262	12040
VEN	Venezuela	32088	Monsenor Jose Vicente De	0.41	0.46	0.49	9263	8035	7295
VEN	Venezuela	32089	Ospino	0.41	0.46	0.49	21122	18323	16634
VEN	Venezuela	32090	Paez	0.41	0.46	0.49	68780	59667	54166
VEN	Venezuela	32091	Papelon	0.41	0.46	0.49	7034	6102	5540
VEN	Venezuela	32092	San Genaro De Boconoito	0.41	0.46	0.49	10452	9067	8231
VEN	Venezuela	32093	San Rafael De Onoto	0.41	0.46	0.49	7146	6199	5628
VEN	Venezuela	32094	Santa Rosalia	0.41	0.46	0.49	7278	6314	5732
VEN	Venezuela	32095	Sucre	0.38	0.72	0.92	17358	3119	0
VEN	Venezuela	32096	Turen	0.41	0.46	0.49	25871	22443	20374
VEN	Venezuela	32097	Andres Eloy Blanco	0.41	0.46	0.49	10105	8766	7958
VEN	Venezuela	32098	Andres Mata	0.41	0.46	0.49	8444	7325	6650
VEN	Venezuela	32099	Arismendi	0.41	0.46	0.49	18534	16078	14596
VEN	Venezuela	32100	Bermudez	0.41	0.46	0.49	52606	45636	41429
VEN	Venezuela	32101	Bolivar	0.41	0.46	0.49	8760	7599	6899
VEN	Venezuela	32102	Cajigal	0.41	0.46	0.49	8307	7206	6542
VEN	Venezuela	32103	Cruz Salmeron Acosta	0.41	0.46	0.49	13717	11899	10802
VEN	Venezuela	32104	Isla	0.41	0.46	0.49	16970	14721	13364
VEN	Venezuela	32105	Libertador	0.41	0.46	0.49	3734	3239	2941
VEN	Venezuela	32106	Marino	0.41	0.46	0.49	8560	7426	6741
VEN	Venezuela	32107	Mejia	0.41	0.46	0.49	5741	4980	4521
VEN	Venezuela	32108	Montes	0.41	0.46	0.49	21811	18921	17176
VEN	Venezuela	32109	Ribero	0.41	0.46	0.49	23131	20066	18216
VEN	Venezuela	32110	Sucre	0.41	0.46	0.49	143282	124297	112838
VEN	Venezuela	32111	Valdez	0.41	0.46	0.49	15045	13051	11848
VEN	Venezuela	32112	Andres Bello	0.41	0.54	0.62	8669	5639	3871
VEN	Venezuela	32113	Ayacucho	0.41	0.54	0.62	25153	16362	11232
VEN	Venezuela	32114	Bolivar	0.41	0.54	0.62	26579	17289	11868
VEN	Venezuela	32115	Cardenas	0.41	0.54	0.62	51801	33696	23131
VEN	Venezuela	32116	Cordoba	0.41	0.54	0.62	14431	9387	6444
VEN	Venezuela	32117	Fernandez Feo	0.41	0.54	0.62	20138	13099	8992
VEN	Venezuela	32118	Garcia De Hevia	0.41	0.54	0.62	19665	12792	8781

VEN	Venezuela	32119	Guasimos	0.41	0.54	0.62	17531	11404	7828
VEN	Venezuela	32120	Independencia	0.41	0.54	0.62	14700	9562	6564
VEN	Venezuela	32121	Jauregui	0.41	0.54	0.62	22033	14332	9839
VEN	Venezuela	32122	Junin	0.38	0.78	0.96	37077	1683	0
VEN	Venezuela	32123	Libertad	0.41	0.54	0.62	11783	7665	5262
VEN	Venezuela	32124	Libertador	0.41	0.54	0.62	9324	6065	4164
VEN	Venezuela	32125	Lobatera	0.41	0.54	0.62	6883	4478	3074
VEN	Venezuela	32126	Michelena	0.41	0.54	0.62	7754	5044	3462
VEN	Venezuela	32127	Panamericano	0.41	0.54	0.62	15818	10289	7063
VEN	Venezuela	32128	Pedro Maria Urena	0.41	0.54	0.62	22612	14709	10097
VEN	Venezuela	32129	Samuel Dario Maldonado	0.41	0.54	0.62	8916	5800	3981
VEN	Venezuela	32130	San Cristobal	0.41	0.54	0.62	120339	78279	53735
VEN	Venezuela	32131	Seboruco	0.41	0.54	0.62	8448	5495	3772
VEN	Venezuela	32132	Sucre	0.41	0.54	0.62	3366	2189	1503
VEN	Venezuela	32133	Uribante	0.41	0.54	0.62	7702	5010	3439
VEN	Venezuela	32134	Bocono	0.41	0.54	0.62	34404	22379	15362
VEN	Venezuela	32135	Candelaria	0.41	0.54	0.62	13043	8484	5824
VEN	Venezuela	32136	Carache	0.41	0.54	0.62	10882	7078	4859
VEN	Venezuela	32137	Escuque	0.41	0.54	0.62	11794	7672	5266
VEN	Venezuela	32138	Miranda	0.41	0.54	0.62	14584	9487	6512
VEN	Venezuela	32139	Monte Carmelo	0.41	0.54	0.62	5521	3591	2465
VEN	Venezuela	32140	Motatan	0.41	0.54	0.62	8865	5767	3958
VEN	Venezuela	32141	Pampan	0.41	0.54	0.62	19277	12540	8608
VEN	Venezuela	32142	Rafael Rangel	0.41	0.54	0.62	15454	10053	6901
VEN	Venezuela	32143	San Rafael De Carvajal	0.41	0.54	0.62	21937	14270	9796
VEN	Venezuela	32144	Sucre	0.41	0.54	0.62	19919	12957	8894
VEN	Venezuela	32145	Trujillo	0.41	0.54	0.62	33123	21546	14790
VEN	Venezuela	32146	Urdaneta	0.23	0.34	0.38	20884	16729	15392
VEN	Venezuela	32147	Valera	0.41	0.54	0.62	51184	33295	22855
VEN	Venezuela	32148	Autonomo Bolivar	0.41	0.46	0.49	19258	16706	15166
VEN	Venezuela	32149	Autonomo Bruzual	0.41	0.46	0.49	27926	24226	21992
VEN	Venezuela	32150	Autonomo Jose Antonio Pae	0.41	0.46	0.49	8218	7129	6472
VEN	Venezuela	32151	Autonomo Nirgua	0.41	0.46	0.49	24838	21547	19561

VEN	Venezuela	32152	Autonomo Pena	0.41	0.46	0.49	42363	36750	33362
VEN	Venezuela	32153	Autonomo San Felipe	0.41	0.46	0.49	92277	80050	72670
VEN	Venezuela	32154	Autonomo Sucre	0.41	0.46	0.49	23200	20126	18270
VEN	Venezuela	32155	Autonomo Urachique	0.41	0.46	0.49	9093	7888	7161
VEN	Venezuela	32156	Almirante Padilla	0.23	0.34	0.38	6046	4843	4456
VEN	Venezuela	32157	Baralt	0.41	0.54	0.62	36799	23937	16432
VEN	Venezuela	32158	Cabimas	0.41	0.54	0.62	122263	79530	54594
VEN	Venezuela	32159	Catatumbo	0.41	0.54	0.62	30476	19824	13608
VEN	Venezuela	32160	Colon	0.41	0.54	0.62	66307	43132	29608
VEN	Venezuela	32161	Isla	0.41	0.54	0.62	476	310	213
VEN	Venezuela	32162	Jesus Enrique Lozada	0.41	0.54	0.62	52155	33926	23289
VEN	Venezuela	32163	La Canada De Urdaneta	0.41	0.54	0.62	38202	24850	17058
VEN	Venezuela	32164	Lagunillas	0.41	0.54	0.62	82284	53525	36742
VEN	Venezuela	32165	Machiques De Perija	0.41	0.54	0.62	52138	33915	23281
VEN	Venezuela	32166	Mara	0.41	0.54	0.62	88436	57526	39489
VEN	Venezuela	32167	Maracaibo	0.41	0.54	0.62	775231	504275	346163
VEN	Venezuela	32168	Miranda	0.41	0.54	0.62	41116	26745	18359
VEN	Venezuela	32169	Paez	0.41	0.54	0.62	36871	23984	16464
VEN	Venezuela	32170	Rosario De Perija	0.41	0.54	0.62	35534	23114	15867
VEN	Venezuela	32171	Santa Rita	0.41	0.54	0.62	25732	16738	11490
VEN	Venezuela	32172	Sucre	0.41	0.54	0.62	24554	15972	10964
VEN	Venezuela	32173	Valmore Rodriguez	0.41	0.54	0.62	21540	14011	9618
ZMB	Zambia	65221	Chavuma	0.00	0.00	0.00	26994	26994	26994
ZMB	Zambia	65222	Kabompo	0.00	0.00	0.00	86272	86272	86272
ZMB	Zambia	65223	Kasempa	0.00	0.00	0.00	63830	63830	63830
ZMB	Zambia	65224	Mufumbwe	0.00	0.00	0.00	50819	50819	50819
ZMB	Zambia	65225	Mwinilunga	0.00	0.00	0.00	126524	126524	126524
ZMB	Zambia	65226	Solwezi	0.00	0.00	0.00	225052	225052	225052
ZMB	Zambia	65227	Zambezi	0.00	0.00	0.00	87125	87125	87125
ZMB	Zambia	65239	Kalabo	0.00	0.00	0.00	122802	122802	122802
ZMB	Zambia	65240	Kaoma	0.00	0.00	0.00	172277	172277	172277
ZMB	Zambia	65241	Lukulu	0.00	0.00	0.00	72905	72905	72905
ZMB	Zambia	65242	Mongu	0.00	0.00	0.00	175429	175429	175429

ZMB	Zambia	65243	Senanga	0.00	0.00	0.00	124908	124908	124908
ZMB	Zambia	65244	Sesheke	0.00	0.00	0.00	90938	90938	90938
ZMB	Zambia	65245	Shang'ombo	0.00	0.00	0.00	88077	88077	88077

Supplementary table 5: Bias correction terms for each country and year calculated as the ratio of survey-derived Global Burden of Disease Study estimates of mean coverage of third dose diphtheria-tetanus-pertussis-containing vaccine (DTP3) and WHO/UNICEF Joint Reporting Form administratively reported estimates. An excel version of this data is also available on Dryad Digital Repository [link to be inserted upon manuscript acceptance].

Country code	Year	GBD mean estimate	Administrative estimate	Bias correction term
AGO	1980	0.14	NA	NA
AGO	1981	0.14	NA	NA
AGO	1982	0.14	NA	NA
AGO	1983	0.14	0.06	2.37
AGO	1984	0.15	NA	NA
AGO	1985	0.15	0.08	1.87
AGO	1986	0.16	0.10	1.55
AGO	1987	0.16	0.10	1.62
AGO	1988	0.17	0.12	1.43
AGO	1989	0.18	0.18	1.02
AGO	1990	0.19	0.24	0.81
AGO	1991	0.21	0.26	0.80
AGO	1992	0.22	0.21	1.04
AGO	1993	0.23	0.30	0.77
AGO	1994	0.24	0.27	0.91
AGO	1995	0.26	0.42	0.62
AGO	1996	0.27	0.28	0.97
AGO	1997	0.29	0.41	0.70
AGO	1998	0.31	0.45	0.68
AGO	1999	0.33	0.22	1.51
AGO	2000	0.37	0.31	1.19
AGO	2001	0.42	0.41	1.01
AGO	2002	0.47	0.47	1.00
AGO	2003	0.52	0.46	1.14
AGO	2004	0.58	0.59	0.99
AGO	2005	0.64	0.47	1.35
AGO	2006	0.69	0.44	1.56
AGO	2007	0.74	0.83	0.89
AGO	2008	0.77	0.81	0.96
AGO	2009	0.80	0.73	1.10
AGO	2010	0.83	0.91	0.91
AGO	2011	0.84	0.86	0.98
AGO	2012	0.85	0.91	0.94
AGO	2013	0.86	0.93	0.93
AGO	2014	0.87	0.80	1.08
AGO	2015	0.87	0.80	1.09
ARG	1980	0.52	0.44	1.18
ARG	1981	0.54	0.49	1.11
ARG	1982	0.57	0.58	0.99
ARG	1983	0.60	0.61	0.99
ARG	1984	0.63	0.62	1.02
ARG	1985	0.67	0.66	1.01
ARG	1986	0.70	0.67	1.04
ARG	1987	0.73	0.75	0.97
ARG	1988	0.75	0.80	0.94
ARG	1989	0.77	0.80	0.97
ARG	1990	0.79	0.87	0.91
ARG	1991	0.80	0.80	1.00
ARG	1992	0.81	0.81	1.01

ARG	1993	0.82	0.81	1.02
ARG	1994	0.83	0.82	1.01
ARG	1995	0.84	0.85	0.99
ARG	1996	0.85	0.83	1.02
ARG	1997	0.85	0.88	0.97
ARG	1998	0.86	0.84	1.02
ARG	1999	0.87	0.86	1.01
ARG	2000	0.88	0.83	1.06
ARG	2001	0.89	0.83	1.07
ARG	2002	0.90	0.93	0.97
ARG	2003	0.91	0.96	0.94
ARG	2004	0.91	0.98	0.93
ARG	2005	0.92	0.98	0.94
ARG	2006	0.92	0.91	1.01
ARG	2007	0.93	0.91	1.02
ARG	2008	0.93	0.93	1.00
ARG	2009	0.93	0.94	0.99
ARG	2010	0.93	0.94	0.99
ARG	2011	0.93	0.91	1.03
ARG	2012	0.94	0.91	1.03
ARG	2013	0.94	0.94	1.00
ARG	2014	0.94	0.94	1.00
ARG	2015	0.94	0.94	1.00
BDI	1980	0.58	NA	NA
BDI	1981	0.58	0.36	1.62
BDI	1982	0.59	0.33	1.80
BDI	1983	0.61	0.44	1.38
BDI	1984	0.62	0.26	2.37
BDI	1985	0.63	0.36	1.75
BDI	1986	0.64	0.53	1.21
BDI	1987	0.65	0.68	0.95
BDI	1988	0.66	0.68	0.97
BDI	1989	0.67	0.82	0.81
BDI	1990	0.67	0.86	0.78
BDI	1991	0.68	0.83	0.82
BDI	1992	0.68	0.80	0.85
BDI	1993	0.68	0.63	1.07
BDI	1994	0.67	0.48	1.40
BDI	1995	0.67	0.63	1.06
BDI	1996	0.67	0.55	1.22
BDI	1997	0.67	0.60	1.12
BDI	1998	0.68	0.50	1.35
BDI	1999	0.69	0.63	1.10
BDI	2000	0.71	0.68	1.05
BDI	2001	0.74	0.59	1.25
BDI	2002	0.77	0.95	0.81
BDI	2003	0.80	0.94	0.85
BDI	2004	0.83	0.83	1.00
BDI	2005	0.86	0.87	0.99
BDI	2006	0.89	0.92	0.97

BDI	2007	0.92	0.99	0.92
BDI	2008	0.93	0.92	1.01
BDI	2009	0.95	0.99	0.96
BDI	2010	0.96	0.96	1.00
BDI	2011	0.96	0.99	0.97
BDI	2012	0.97	0.96	1.01
BDI	2013	0.97	0.96	1.01
BDI	2014	0.98	0.95	1.03
BDI	2015	0.98	0.94	1.04
BEN	1980	0.45	NA	NA
BEN	1981	0.46	NA	NA
BEN	1982	0.47	NA	NA
BEN	1983	0.48	NA	NA
BEN	1984	0.49	NA	NA
BEN	1985	0.50	0.17	2.93
BEN	1986	0.52	0.17	3.04
BEN	1987	0.54	0.25	2.17
BEN	1988	0.57	0.30	1.90
BEN	1989	0.60	0.48	1.26
BEN	1990	0.63	0.78	0.81
BEN	1991	0.66	0.68	0.97
BEN	1992	0.68	0.79	0.86
BEN	1993	0.69	0.77	0.90
BEN	1994	0.71	0.86	0.82
BEN	1995	0.71	0.89	0.80
BEN	1996	0.71	0.80	0.89
BEN	1997	0.71	0.78	0.91
BEN	1998	0.71	0.81	0.88
BEN	1999	0.71	0.90	0.79
BEN	2000	0.71	0.88	0.81
BEN	2001	0.71	0.84	0.84
BEN	2002	0.71	0.93	0.76
BEN	2003	0.71	0.88	0.80
BEN	2004	0.71	0.83	0.85
BEN	2005	0.71	0.93	0.77
BEN	2006	0.72	0.93	0.77
BEN	2007	0.73	0.97	0.75
BEN	2008	0.73	0.93	0.79
BEN	2009	0.74	0.98	0.76
BEN	2010	0.75	0.98	0.76
BEN	2011	0.76	0.99	0.76
BEN	2012	0.76	0.85	0.90
BEN	2013	0.77	0.93	0.83
BEN	2014	0.78	0.90	0.86
BEN	2015	0.78	0.82	0.96
BFA	1980	0.32	NA	NA
BFA	1981	0.32	0.02	16.22
BFA	1982	0.34	NA	NA
BFA	1983	0.35	NA	NA
BFA	1984	0.36	NA	NA

BFA	1985	0.37	NA	NA
BFA	1986	0.38	0.34	1.13
BFA	1987	0.39	0.34	1.15
BFA	1988	0.40	0.23	1.73
BFA	1989	0.41	NA	NA
BFA	1990	0.41	NA	NA
BFA	1991	0.41	NA	NA
BFA	1992	0.41	0.39	1.06
BFA	1993	0.42	0.47	0.89
BFA	1994	0.42	0.41	1.03
BFA	1995	0.43	0.47	0.92
BFA	1996	0.45	0.48	0.93
BFA	1997	0.47	0.70	0.67
BFA	1998	0.51	0.40	1.27
BFA	1999	0.56	0.34	1.64
BFA	2000	0.62	0.57	1.08
BFA	2001	0.67	0.68	0.99
BFA	2002	0.73	0.69	1.05
BFA	2003	0.77	0.84	0.92
BFA	2004	0.81	0.88	0.92
BFA	2005	0.84	0.96	0.88
BFA	2006	0.87	0.95	0.91
BFA	2007	0.89	0.99	0.90
BFA	2008	0.90	0.99	0.91
BFA	2009	0.91	0.99	0.92
BFA	2010	0.92	0.91	1.01
BFA	2011	0.93	0.91	1.02
BFA	2012	0.93	0.90	1.04
BFA	2013	0.94	0.88	1.06
BFA	2014	0.94	0.91	1.03
BFA	2015	0.94	0.91	1.03
BOL	1980	0.30	0.11	2.77
BOL	1981	0.32	0.13	2.46
BOL	1982	0.34	0.12	2.81
BOL	1983	0.35	0.10	3.54
BOL	1984	0.37	0.06	6.19
BOL	1985	0.39	0.33	1.18
BOL	1986	0.40	0.29	1.39
BOL	1987	0.42	0.24	1.74
BOL	1988	0.43	0.39	1.10
BOL	1989	0.45	0.39	1.15
BOL	1990	0.47	0.41	1.15
BOL	1991	0.50	0.58	0.86
BOL	1992	0.53	0.77	0.68
BOL	1993	0.56	0.81	0.69
BOL	1994	0.59	0.81	0.73
BOL	1995	0.63	0.88	0.71
BOL	1996	0.66	0.82	0.80
BOL	1997	0.68	0.78	0.88
BOL	1998	0.71	0.76	0.94

BOL	1999	0.74	0.87	0.85
BOL	2000	0.76	0.89	0.85
BOL	2001	0.78	0.91	0.86
BOL	2002	0.80	0.99	0.81
BOL	2003	0.82	0.99	0.83
BOL	2004	0.83	0.84	0.99
BOL	2005	0.85	0.85	1.00
BOL	2006	0.86	0.83	1.04
BOL	2007	0.88	0.82	1.07
BOL	2008	0.89	0.83	1.07
BOL	2009	0.90	0.85	1.06
BOL	2010	0.91	0.80	1.14
BOL	2011	0.92	0.82	1.13
BOL	2012	0.93	0.80	1.16
BOL	2013	0.94	0.80	1.17
BOL	2014	0.94	0.85	1.11
BOL	2015	0.95	0.89	1.07
BRA	1980	0.58	0.37	1.57
BRA	1981	0.60	0.47	1.29
BRA	1982	0.63	0.56	1.12
BRA	1983	0.65	0.60	1.09
BRA	1984	0.67	0.68	0.99
BRA	1985	0.69	0.66	1.05
BRA	1986	0.71	0.58	1.22
BRA	1987	0.72	0.58	1.25
BRA	1988	0.74	0.58	1.28
BRA	1989	0.76	0.56	1.36
BRA	1990	0.78	0.66	1.18
BRA	1991	0.80	0.78	1.03
BRA	1992	0.83	0.71	1.16
BRA	1993	0.84	0.75	1.13
BRA	1994	0.86	0.74	1.16
BRA	1995	0.87	0.81	1.08
BRA	1996	0.89	0.77	1.15
BRA	1997	0.90	0.79	1.14
BRA	1998	0.92	0.93	0.99
BRA	1999	0.93	0.94	0.99
BRA	2000	0.94	0.98	0.96
BRA	2001	0.95	0.98	0.97
BRA	2002	0.96	0.99	0.97
BRA	2003	0.96	0.99	0.97
BRA	2004	0.97	0.99	0.98
BRA	2005	0.97	0.99	0.98
BRA	2006	0.97	0.99	0.98
BRA	2007	0.98	0.99	0.99
BRA	2008	0.98	0.99	0.99
BRA	2009	0.98	0.99	0.99
BRA	2010	0.98	0.99	0.99
BRA	2011	0.98	0.99	0.99
BRA	2012	0.98	0.95	1.03

BRA	2013	0.98	0.97	1.01
BRA	2014	0.98	0.93	1.06
BRA	2015	0.98	0.96	1.03
CAF	1980	0.32	0.13	2.44
CAF	1981	0.33	0.11	2.99
CAF	1982	0.34	0.14	2.46
CAF	1983	0.36	0.16	2.26
CAF	1984	0.38	0.17	2.25
CAF	1985	0.41	0.20	2.03
CAF	1986	0.43	0.19	2.26
CAF	1987	0.45	0.18	2.51
CAF	1988	0.47	0.30	1.58
CAF	1989	0.49	0.43	1.14
CAF	1990	0.50	0.61	0.82
CAF	1991	0.49	0.66	0.75
CAF	1992	0.48	0.31	1.55
CAF	1993	0.46	NA	NA
CAF	1994	0.44	0.40	1.11
CAF	1995	0.43	0.45	0.95
CAF	1996	0.41	0.53	0.78
CAF	1997	0.40	0.53	0.76
CAF	1998	0.40	0.45	0.89
CAF	1999	0.41	0.27	1.52
CAF	2000	0.43	0.29	1.47
CAF	2001	0.45	0.23	1.94
CAF	2002	0.47	0.23	2.02
CAF	2003	0.48	0.28	1.70
CAF	2004	0.48	0.50	0.96
CAF	2005	0.47	0.46	1.02
CAF	2006	0.46	0.88	0.52
CAF	2007	0.44	0.84	0.52
CAF	2008	0.42	0.51	0.82
CAF	2009	0.41	0.76	0.54
CAF	2010	0.41	0.58	0.70
CAF	2011	0.41	0.64	0.64
CAF	2012	0.42	0.59	0.71
CAF	2013	0.42	0.28	1.51
CAF	2014	0.43	0.45	0.96
CAF	2015	0.44	0.47	0.94
CIV	1980	0.48	NA	NA
CIV	1981	0.48	NA	NA
CIV	1982	0.48	NA	NA
CIV	1983	0.48	NA	NA
CIV	1984	0.48	0.19	2.52
CIV	1985	0.48	0.25	1.93
CIV	1986	0.49	0.30	1.62
CIV	1987	0.49	0.73	0.67
CIV	1988	0.49	0.25	1.97
CIV	1989	0.50	0.42	1.18
CIV	1990	0.50	NA	NA

CIV	1991	0.51	0.54	0.95
CIV	1992	0.52	0.49	1.07
CIV	1993	0.54	0.50	1.08
CIV	1994	0.56	0.41	1.37
CIV	1995	0.58	0.41	1.43
CIV	1996	0.61	0.55	1.11
CIV	1997	0.63	0.70	0.90
CIV	1998	0.65	0.64	1.02
CIV	1999	0.67	0.58	1.15
CIV	2000	0.68	0.62	1.09
CIV	2001	0.68	0.59	1.16
CIV	2002	0.68	0.73	0.94
CIV	2003	0.68	0.48	1.43
CIV	2004	0.68	0.50	1.37
CIV	2005	0.68	0.56	1.22
CIV	2006	0.68	0.77	0.88
CIV	2007	0.68	0.76	0.89
CIV	2008	0.68	0.74	0.92
CIV	2009	0.69	0.81	0.85
CIV	2010	0.70	0.85	0.82
CIV	2011	0.72	0.62	1.16
CIV	2012	0.74	0.94	0.79
CIV	2013	0.77	0.99	0.77
CIV	2014	0.78	0.87	0.90
CIV	2015	0.80	0.91	0.88
CMR	1980	0.39	NA	NA
CMR	1981	0.40	0.05	7.97
CMR	1982	0.41	NA	NA
CMR	1983	0.43	0.25	1.71
CMR	1984	0.44	0.27	1.63
CMR	1985	0.45	0.50	0.90
CMR	1986	0.46	NA	NA
CMR	1987	0.46	NA	NA
CMR	1988	0.45	0.20	2.27
CMR	1989	0.45	0.18	2.51
CMR	1990	0.45	0.36	1.25
CMR	1991	0.45	0.34	1.32
CMR	1992	0.45	0.37	1.21
CMR	1993	0.45	0.34	1.32
CMR	1994	0.45	0.38	1.19
CMR	1995	0.46	0.46	1.00
CMR	1996	0.47	0.44	1.07
CMR	1997	0.49	0.43	1.13
CMR	1998	0.51	0.48	1.06
CMR	1999	0.54	0.48	1.13
CMR	2000	0.57	0.53	1.08
CMR	2001	0.61	0.43	1.41
CMR	2002	0.64	0.63	1.01
CMR	2003	0.66	0.73	0.91
CMR	2004	0.68	0.73	0.93

CMR	2005	0.69	0.80	0.87
CMR	2006	0.70	0.81	0.87
CMR	2007	0.71	0.82	0.87
CMR	2008	0.72	0.84	0.86
CMR	2009	0.73	0.80	0.91
CMR	2010	0.73	0.84	0.87
CMR	2011	0.75	0.82	0.91
CMR	2012	0.76	0.85	0.89
CMR	2013	0.77	0.89	0.86
CMR	2014	0.78	0.87	0.89
CMR	2015	0.79	0.84	0.94
COD	1980	0.24	NA	NA
COD	1981	0.25	0.18	1.37
COD	1982	0.26	NA	NA
COD	1983	0.26	0.16	1.65
COD	1984	0.27	NA	NA
COD	1985	0.28	0.37	0.77
COD	1986	0.29	0.39	0.75
COD	1987	0.30	0.38	0.78
COD	1988	0.30	0.41	0.73
COD	1989	0.30	0.38	0.78
COD	1990	0.29	0.36	0.80
COD	1991	0.28	0.16	1.73
COD	1992	0.27	NA	NA
COD	1993	0.25	0.29	0.87
COD	1994	0.24	0.29	0.83
COD	1995	0.23	0.23	1.00
COD	1996	0.22	0.18	1.24
COD	1997	0.22	0.18	1.24
COD	1998	0.24	0.18	1.31
COD	1999	0.26	0.25	1.05
COD	2000	0.30	0.40	0.75
COD	2001	0.34	0.32	1.06
COD	2002	0.38	0.43	0.89
COD	2003	0.43	0.49	0.87
COD	2004	0.47	0.64	0.73
COD	2005	0.51	0.73	0.70
COD	2006	0.54	0.77	0.71
COD	2007	0.58	0.87	0.66
COD	2008	0.60	0.83	0.73
COD	2009	0.62	0.92	0.68
COD	2010	0.64	0.78	0.82
COD	2011	0.66	0.90	0.73
COD	2012	0.67	0.89	0.75
COD	2013	0.68	0.87	0.78
COD	2014	0.69	0.93	0.74
COD	2015	0.70	0.94	0.74
COG	1980	0.55	NA	NA
COG	1981	0.56	0.42	1.34
COG	1982	0.58	NA	NA

COG	1983	0.60	0.50	1.19
COG	1984	0.61	0.53	1.15
COG	1985	0.62	0.54	1.16
COG	1986	0.64	0.68	0.93
COG	1987	0.64	0.57	1.12
COG	1988	0.64	0.50	1.28
COG	1989	0.64	0.66	0.97
COG	1990	0.63	0.77	0.82
COG	1991	0.61	0.70	0.87
COG	1992	0.59	0.65	0.91
COG	1993	0.56	0.60	0.94
COG	1994	0.53	NA	NA
COG	1995	0.50	0.47	1.07
COG	1996	0.48	0.75	0.63
COG	1997	0.45	0.23	1.94
COG	1998	0.44	NA	NA
COG	1999	0.45	0.29	1.56
COG	2000	0.48	0.33	1.46
COG	2001	0.52	0.31	1.67
COG	2002	0.56	0.41	1.36
COG	2003	0.59	0.50	1.18
COG	2004	0.62	0.67	0.93
COG	2005	0.65	0.65	0.99
COG	2006	0.67	0.79	0.85
COG	2007	0.69	0.80	0.86
COG	2008	0.71	0.89	0.79
COG	2009	0.72	0.91	0.79
COG	2010	0.74	0.90	0.82
COG	2011	0.76	0.90	0.84
COG	2012	0.77	0.85	0.91
COG	2013	0.79	0.85	0.92
COG	2014	0.80	0.90	0.89
COG	2015	0.81	0.80	1.01
COL	1980	0.82	0.16	5.13
COL	1981	0.83	0.20	4.16
COL	1982	0.84	0.26	3.24
COL	1983	0.85	0.42	2.03
COL	1984	0.86	0.61	1.40
COL	1985	0.86	0.60	1.43
COL	1986	0.86	0.48	1.78
COL	1987	0.85	0.58	1.47
COL	1988	0.85	0.74	1.14
COL	1989	0.84	0.78	1.08
COL	1990	0.83	0.88	0.95
COL	1991	0.83	0.84	0.99
COL	1992	0.82	0.78	1.05
COL	1993	0.82	0.83	0.99
COL	1994	0.82	0.88	0.93
COL	1995	0.82	0.94	0.88
COL	1996	0.82	0.92	0.90

COL	1997	0.82	0.84	0.98
COL	1998	0.82	0.73	1.13
COL	1999	0.83	0.74	1.12
COL	2000	0.84	0.79	1.06
COL	2001	0.85	0.80	1.06
COL	2002	0.86	0.81	1.06
COL	2003	0.87	0.92	0.94
COL	2004	0.88	0.89	0.99
COL	2005	0.89	0.93	0.96
COL	2006	0.90	0.93	0.97
COL	2007	0.91	0.93	0.98
COL	2008	0.91	0.92	0.99
COL	2009	0.92	0.92	1.00
COL	2010	0.92	0.88	1.05
COL	2011	0.93	0.85	1.09
COL	2012	0.93	0.91	1.02
COL	2013	0.94	0.91	1.03
COL	2014	0.94	0.90	1.04
COL	2015	0.94	0.91	1.04
ECU	1980	0.66	0.10	6.63
ECU	1981	0.69	0.26	2.67
ECU	1982	0.73	0.35	2.07
ECU	1983	0.75	0.31	2.43
ECU	1984	0.78	0.48	1.63
ECU	1985	0.80	0.41	1.96
ECU	1986	0.82	0.43	1.91
ECU	1987	0.83	0.50	1.67
ECU	1988	0.84	0.54	1.56
ECU	1989	0.85	0.55	1.55
ECU	1990	0.86	0.75	1.14
ECU	1991	0.86	0.69	1.25
ECU	1992	0.86	0.79	1.09
ECU	1993	0.86	0.76	1.13
ECU	1994	0.86	0.80	1.07
ECU	1995	0.85	0.74	1.15
ECU	1996	0.85	0.96	0.89
ECU	1997	0.85	0.76	1.11
ECU	1998	0.84	0.85	0.99
ECU	1999	0.84	0.80	1.05
ECU	2000	0.84	0.88	0.96
ECU	2001	0.84	0.90	0.94
ECU	2002	0.85	0.89	0.95
ECU	2003	0.85	0.89	0.96
ECU	2004	0.86	0.90	0.96
ECU	2005	0.87	0.94	0.93
ECU	2006	0.88	0.98	0.90
ECU	2007	0.90	0.99	0.90
ECU	2008	0.90	0.99	0.91
ECU	2009	0.91	0.99	0.92
ECU	2010	0.92	0.99	0.93

ECU	2011	0.92	0.99	0.93
ECU	2012	0.93	0.99	0.94
ECU	2013	0.94	0.87	1.08
ECU	2014	0.94	0.83	1.14
ECU	2015	0.95	0.78	1.22
ERI	1980	0.39	NA	NA
ERI	1981	0.39	NA	NA
ERI	1982	0.38	NA	NA
ERI	1983	0.37	NA	NA
ERI	1984	0.37	NA	NA
ERI	1985	0.36	NA	NA
ERI	1986	0.36	NA	NA
ERI	1987	0.36	NA	NA
ERI	1988	0.36	NA	NA
ERI	1989	0.37	NA	NA
ERI	1990	0.38	NA	NA
ERI	1991	0.40	NA	NA
ERI	1992	0.42	NA	NA
ERI	1993	0.47	0.28	1.66
ERI	1994	0.52	0.36	1.46
ERI	1995	0.59	0.35	1.69
ERI	1996	0.66	0.46	1.43
ERI	1997	0.72	0.60	1.20
ERI	1998	0.78	0.60	1.29
ERI	1999	0.82	0.56	1.47
ERI	2000	0.86	0.52	1.65
ERI	2001	0.89	0.65	1.37
ERI	2002	0.91	0.70	1.30
ERI	2003	0.93	0.75	1.24
ERI	2004	0.95	0.80	1.18
ERI	2005	0.96	0.80	1.20
ERI	2006	0.96	0.80	1.21
ERI	2007	0.97	0.80	1.21
ERI	2008	0.97	0.85	1.15
ERI	2009	0.98	0.85	1.15
ERI	2010	0.98	0.85	1.15
ERI	2011	0.98	0.94	1.04
ERI	2012	0.98	0.94	1.05
ERI	2013	0.98	0.94	1.05
ERI	2014	0.98	0.94	1.05
ERI	2015	0.98	0.95	1.04
ETH	1980	0.16	NA	NA
ETH	1981	0.17	0.03	5.52
ETH	1982	0.17	0.03	5.74
ETH	1983	0.18	0.04	4.57
ETH	1984	0.20	0.04	4.91
ETH	1985	0.21	0.06	3.57
ETH	1986	0.23	0.07	3.36
ETH	1987	0.26	0.16	1.62
ETH	1988	0.29	0.16	1.79

ETH	1989	0.31	0.26	1.21
ETH	1990	0.34	0.49	0.70
ETH	1991	0.37	0.21	1.75
ETH	1992	0.39	0.13	2.99
ETH	1993	0.41	0.28	1.46
ETH	1994	0.43	0.37	1.15
ETH	1995	0.43	0.57	0.76
ETH	1996	0.43	0.42	1.03
ETH	1997	0.43	0.41	1.05
ETH	1998	0.43	0.37	1.16
ETH	1999	0.43	0.40	1.08
ETH	2000	0.44	0.42	1.04
ETH	2001	0.44	0.51	0.87
ETH	2002	0.45	0.51	0.88
ETH	2003	0.45	0.52	0.86
ETH	2004	0.45	0.66	0.68
ETH	2005	0.44	0.69	0.64
ETH	2006	0.44	0.72	0.61
ETH	2007	0.43	0.73	0.59
ETH	2008	0.44	0.81	0.54
ETH	2009	0.45	0.79	0.57
ETH	2010	0.47	0.86	0.54
ETH	2011	0.49	0.87	0.57
ETH	2012	0.52	0.83	0.63
ETH	2013	0.55	0.82	0.67
ETH	2014	0.58	0.84	0.69
ETH	2015	0.61	0.96	0.63
GAB	1980	0.51	NA	NA
GAB	1981	0.53	NA	NA
GAB	1982	0.54	0.48	1.14
GAB	1983	0.56	0.14	4.02
GAB	1984	0.59	0.31	1.90
GAB	1985	0.61	0.48	1.28
GAB	1986	0.64	0.48	1.34
GAB	1987	0.66	0.59	1.13
GAB	1988	0.68	0.65	1.05
GAB	1989	0.70	0.71	0.98
GAB	1990	0.70	0.78	0.90
GAB	1991	0.69	0.72	0.96
GAB	1992	0.67	0.66	1.02
GAB	1993	0.65	0.65	0.99
GAB	1994	0.61	0.59	1.04
GAB	1995	0.58	0.70	0.82
GAB	1996	0.53	0.61	0.87
GAB	1997	0.49	0.54	0.90
GAB	1998	0.45	0.37	1.21
GAB	1999	0.43	0.31	1.38
GAB	2000	0.42	0.10	4.23
GAB	2001	0.43	0.28	1.55
GAB	2002	0.45	0.33	1.37

GAB	2003	0.48	0.40	1.20
GAB	2004	0.51	0.40	1.26
GAB	2005	0.54	0.40	1.35
GAB	2006	0.57	0.44	1.31
GAB	2007	0.61	0.81	0.76
GAB	2008	0.65	0.82	0.79
GAB	2009	0.68	0.76	0.89
GAB	2010	0.71	0.67	1.05
GAB	2011	0.73	0.75	0.98
GAB	2012	0.75	0.82	0.92
GAB	2013	0.77	0.79	0.98
GAB	2014	0.79	0.70	1.12
GAB	2015	0.80	0.80	1.00
GHA	1980	0.47	0.07	6.72
GHA	1981	0.49	0.22	2.21
GHA	1982	0.50	0.22	2.28
GHA	1983	0.52	0.23	2.24
GHA	1984	0.53	0.19	2.80
GHA	1985	0.55	NA	NA
GHA	1986	0.56	0.15	3.71
GHA	1987	0.57	0.18	3.14
GHA	1988	0.58	0.36	1.60
GHA	1989	0.59	0.35	1.68
GHA	1990	0.60	0.50	1.20
GHA	1991	0.61	0.40	1.53
GHA	1992	0.63	0.40	1.57
GHA	1993	0.65	0.48	1.35
GHA	1994	0.67	0.48	1.40
GHA	1995	0.69	0.51	1.36
GHA	1996	0.72	0.51	1.40
GHA	1997	0.74	0.60	1.23
GHA	1998	0.76	0.68	1.11
GHA	1999	0.77	0.72	1.07
GHA	2000	0.78	0.84	0.93
GHA	2001	0.79	0.76	1.05
GHA	2002	0.81	0.99	0.81
GHA	2003	0.82	0.80	1.02
GHA	2004	0.83	0.80	1.04
GHA	2005	0.85	0.84	1.01
GHA	2006	0.87	0.84	1.04
GHA	2007	0.89	0.94	0.95
GHA	2008	0.91	0.93	0.98
GHA	2009	0.92	0.94	0.98
GHA	2010	0.93	0.94	0.99
GHA	2011	0.94	0.91	1.03
GHA	2012	0.95	0.92	1.03
GHA	2013	0.95	0.90	1.06
GHA	2014	0.96	0.98	0.98
GHA	2015	0.96	0.89	1.08
GIN	1980	0.16	NA	NA

GIN	1981	0.16	NA	NA
GIN	1982	0.16	NA	NA
GIN	1983	0.17	NA	NA
GIN	1984	0.17	NA	NA
GIN	1985	0.17	NA	NA
GIN	1986	0.18	0.02	8.84
GIN	1987	0.19	NA	NA
GIN	1988	0.21	NA	NA
GIN	1989	0.23	0.17	1.33
GIN	1990	0.25	0.20	1.24
GIN	1991	0.27	0.41	0.67
GIN	1992	0.30	0.52	0.58
GIN	1993	0.33	NA	NA
GIN	1994	0.36	0.73	0.49
GIN	1995	0.38	0.73	0.53
GIN	1996	0.40	0.48	0.84
GIN	1997	0.42	0.53	0.80
GIN	1998	0.44	0.56	0.78
GIN	1999	0.45	0.57	0.80
GIN	2000	0.47	0.57	0.82
GIN	2001	0.48	0.57	0.84
GIN	2002	0.49	0.58	0.85
GIN	2003	0.50	0.69	0.73
GIN	2004	0.51	0.69	0.74
GIN	2005	0.51	0.86	0.60
GIN	2006	0.52	0.89	0.58
GIN	2007	0.52	0.93	0.56
GIN	2008	0.52	0.70	0.75
GIN	2009	0.53	0.85	0.62
GIN	2010	0.54	0.90	0.60
GIN	2011	0.55	0.85	0.65
GIN	2012	0.57	0.99	0.57
GIN	2013	0.58	0.90	0.64
GIN	2014	0.58	0.60	0.97
GIN	2015	0.59	0.60	0.98
GMB	1980	0.68	NA	NA
GMB	1981	0.69	0.76	0.91
GMB	1982	0.70	NA	NA
GMB	1983	0.70	0.62	1.13
GMB	1984	0.71	NA	NA
GMB	1985	0.72	0.78	0.93
GMB	1986	0.74	0.73	1.01
GMB	1987	0.75	0.85	0.88
GMB	1988	0.76	0.82	0.93
GMB	1989	0.77	0.87	0.88
GMB	1990	0.78	0.92	0.84
GMB	1991	0.78	0.85	0.92
GMB	1992	0.78	0.85	0.92
GMB	1993	0.78	0.90	0.86
GMB	1994	0.78	0.93	0.83

GMB	1995	0.77	0.96	0.81
GMB	1996	0.77	0.96	0.80
GMB	1997	0.77	0.96	0.80
GMB	1998	0.77	0.97	0.80
GMB	1999	0.78	0.88	0.88
GMB	2000	0.79	0.74	1.07
GMB	2001	0.81	0.96	0.84
GMB	2002	0.83	0.80	1.04
GMB	2003	0.85	0.90	0.94
GMB	2004	0.87	0.92	0.94
GMB	2005	0.88	0.89	0.99
GMB	2006	0.90	0.93	0.97
GMB	2007	0.91	0.94	0.97
GMB	2008	0.92	0.96	0.96
GMB	2009	0.93	0.98	0.95
GMB	2010	0.94	0.95	0.98
GMB	2011	0.94	0.96	0.98
GMB	2012	0.94	0.98	0.96
GMB	2013	0.95	0.98	0.97
GMB	2014	0.95	0.96	0.99
GMB	2015	0.95	0.97	0.98
GNB	1980	0.42	NA	NA
GNB	1981	0.43	NA	NA
GNB	1982	0.44	NA	NA
GNB	1983	0.44	0.09	4.90
GNB	1984	0.45	0.18	2.52
GNB	1985	0.47	0.18	2.61
GNB	1986	0.49	0.29	1.69
GNB	1987	0.51	0.55	0.93
GNB	1988	0.53	0.66	0.80
GNB	1989	0.55	0.60	0.91
GNB	1990	0.56	0.61	0.92
GNB	1991	0.57	0.63	0.91
GNB	1992	0.58	0.67	0.86
GNB	1993	0.58	0.65	0.89
GNB	1994	0.58	0.74	0.78
GNB	1995	0.57	0.45	1.27
GNB	1996	0.56	0.53	1.06
GNB	1997	0.55	0.63	0.88
GNB	1998	0.54	NA	NA
GNB	1999	0.53	0.06	8.88
GNB	2000	0.55	NA	NA
GNB	2001	0.57	0.47	1.21
GNB	2002	0.59	0.50	1.18
GNB	2003	0.61	0.77	0.79
GNB	2004	0.63	0.80	0.78
GNB	2005	0.65	0.80	0.81
GNB	2006	0.66	0.77	0.86
GNB	2007	0.68	0.96	0.71
GNB	2008	0.70	0.79	0.88

GNB	2009	0.72	0.82	0.88
GNB	2010	0.74	0.86	0.86
GNB	2011	0.76	0.89	0.86
GNB	2012	0.78	0.90	0.86
GNB	2013	0.79	0.96	0.82
GNB	2014	0.79	0.83	0.95
GNB	2015	0.80	0.90	0.88
GNQ	1980	0.47	NA	NA
GNQ	1981	0.48	NA	NA
GNQ	1982	0.49	NA	NA
GNQ	1983	0.50	NA	NA
GNQ	1984	0.50	NA	NA
GNQ	1985	0.51	0.03	16.84
GNQ	1986	0.51	0.03	17.14
GNQ	1987	0.53	NA	NA
GNQ	1988	0.54	0.18	2.99
GNQ	1989	0.54	0.14	3.88
GNQ	1990	0.54	NA	NA
GNQ	1991	0.53	NA	NA
GNQ	1992	0.51	0.64	0.80
GNQ	1993	0.48	0.60	0.80
GNQ	1994	0.44	NA	NA
GNQ	1995	0.40	0.64	0.62
GNQ	1996	0.36	0.64	0.56
GNQ	1997	0.33	0.81	0.41
GNQ	1998	0.30	NA	NA
GNQ	1999	0.29	0.40	0.72
GNQ	2000	0.29	0.32	0.90
GNQ	2001	0.29	0.32	0.92
GNQ	2002	0.31	0.65	0.48
GNQ	2003	0.33	0.41	0.80
GNQ	2004	0.35	0.46	0.76
GNQ	2005	0.37	0.34	1.09
GNQ	2006	0.40	0.34	1.16
GNQ	2007	0.42	0.41	1.03
GNQ	2008	0.45	0.74	0.61
GNQ	2009	0.47	0.74	0.64
GNQ	2010	0.49	0.44	1.11
GNQ	2011	0.51	0.54	0.94
GNQ	2012	0.52	0.41	1.27
GNQ	2013	0.54	0.24	2.23
GNQ	2014	0.55	0.45	1.23
GNQ	2015	0.57	0.17	3.37
GUY	1980	0.39	0.36	1.09
GUY	1981	0.41	0.41	1.00
GUY	1982	0.44	0.58	0.75
GUY	1983	0.46	0.58	0.80
GUY	1984	0.49	0.70	0.70
GUY	1985	0.52	0.75	0.70
GUY	1986	0.55	0.64	0.87

GUY	1987	0.59	0.67	0.88
GUY	1988	0.62	0.64	0.97
GUY	1989	0.66	0.77	0.85
GUY	1990	0.69	0.82	0.84
GUY	1991	0.73	0.81	0.90
GUY	1992	0.76	0.79	0.96
GUY	1993	0.79	0.93	0.85
GUY	1994	0.82	0.90	0.91
GUY	1995	0.83	0.86	0.97
GUY	1996	0.85	0.83	1.02
GUY	1997	0.86	0.88	0.98
GUY	1998	0.87	0.90	0.96
GUY	1999	0.87	0.83	1.04
GUY	2000	0.87	0.88	0.98
GUY	2001	0.86	0.85	1.02
GUY	2002	0.86	0.91	0.95
GUY	2003	0.86	0.90	0.95
GUY	2004	0.85	0.91	0.94
GUY	2005	0.85	0.93	0.91
GUY	2006	0.85	0.93	0.91
GUY	2007	0.85	0.94	0.91
GUY	2008	0.85	0.93	0.92
GUY	2009	0.86	0.98	0.88
GUY	2010	0.87	0.95	0.91
GUY	2011	0.88	0.93	0.94
GUY	2012	0.89	0.97	0.91
GUY	2013	0.89	0.98	0.91
GUY	2014	0.90	0.98	0.92
GUY	2015	0.91	0.95	0.96
KEN	1980	0.81	NA	NA
KEN	1981	0.82	NA	NA
KEN	1982	0.83	NA	NA
KEN	1983	0.84	NA	NA
KEN	1984	0.85	NA	NA
KEN	1985	0.86	0.70	1.22
KEN	1986	0.86	0.72	1.19
KEN	1987	0.86	NA	NA
KEN	1988	0.85	0.30	2.84
KEN	1989	0.84	0.41	2.06
KEN	1990	0.83	0.42	1.99
KEN	1991	0.82	0.41	2.01
KEN	1992	0.81	0.40	2.03
KEN	1993	0.80	0.42	1.91
KEN	1994	0.79	0.50	1.58
KEN	1995	0.77	0.84	0.92
KEN	1996	0.76	0.77	0.98
KEN	1997	0.74	0.36	2.06
KEN	1998	0.74	0.64	1.16
KEN	1999	0.75	0.79	0.95
KEN	2000	0.77	0.63	1.22

KEN	2001	0.80	0.80	0.99
KEN	2002	0.82	0.84	0.98
KEN	2003	0.86	0.73	1.17
KEN	2004	0.88	0.73	1.21
KEN	2005	0.90	0.76	1.19
KEN	2006	0.92	0.80	1.14
KEN	2007	0.92	0.81	1.14
KEN	2008	0.92	0.85	1.08
KEN	2009	0.91	0.75	1.22
KEN	2010	0.91	0.83	1.09
KEN	2011	0.90	0.88	1.02
KEN	2012	0.89	0.83	1.08
KEN	2013	0.89	0.84	1.06
KEN	2014	0.89	0.81	1.10
KEN	2015	0.90	0.78	1.15
LBR	1980	0.29	NA	NA
LBR	1981	0.29	0.39	0.76
LBR	1982	0.30	NA	NA
LBR	1983	0.30	0.23	1.29
LBR	1984	0.30	NA	NA
LBR	1985	0.30	NA	NA
LBR	1986	0.30	0.15	2.01
LBR	1987	0.31	0.19	1.62
LBR	1988	0.32	NA	NA
LBR	1989	0.33	NA	NA
LBR	1990	0.34	NA	NA
LBR	1991	0.35	NA	NA
LBR	1992	0.36	NA	NA
LBR	1993	0.37	NA	NA
LBR	1994	0.38	NA	NA
LBR	1995	0.38	0.45	0.85
LBR	1996	0.38	0.45	0.85
LBR	1997	0.38	0.26	1.47
LBR	1998	0.39	0.19	2.03
LBR	1999	0.40	0.23	1.74
LBR	2000	0.41	0.48	0.86
LBR	2001	0.42	0.62	0.68
LBR	2002	0.43	0.51	0.85
LBR	2003	0.44	0.38	1.17
LBR	2004	0.46	0.31	1.49
LBR	2005	0.49	0.87	0.56
LBR	2006	0.52	0.88	0.59
LBR	2007	0.55	0.88	0.63
LBR	2008	0.59	0.92	0.64
LBR	2009	0.61	0.92	0.67
LBR	2010	0.64	0.75	0.85
LBR	2011	0.66	0.77	0.86
LBR	2012	0.68	0.93	0.74
LBR	2013	0.70	0.89	0.79
LBR	2014	0.72	0.63	1.14

LBR	2015	0.73	0.65	1.12
MLI	1980	0.26	NA	NA
MLI	1981	0.27	NA	NA
MLI	1982	0.27	NA	NA
MLI	1983	0.27	NA	NA
MLI	1984	0.27	NA	NA
MLI	1985	0.27	0.08	3.40
MLI	1986	0.27	0.03	9.13
MLI	1987	0.28	0.08	3.51
MLI	1988	0.29	0.18	1.62
MLI	1989	0.30	0.26	1.17
MLI	1990	0.32	0.42	0.76
MLI	1991	0.33	0.34	0.97
MLI	1992	0.34	0.38	0.91
MLI	1993	0.36	0.46	0.78
MLI	1994	0.37	0.39	0.95
MLI	1995	0.38	0.49	0.78
MLI	1996	0.39	0.53	0.74
MLI	1997	0.41	0.52	0.78
MLI	1998	0.42	0.56	0.76
MLI	1999	0.45	0.47	0.95
MLI	2000	0.48	0.54	0.89
MLI	2001	0.52	0.61	0.85
MLI	2002	0.56	0.74	0.76
MLI	2003	0.60	0.79	0.76
MLI	2004	0.64	0.86	0.74
MLI	2005	0.66	0.95	0.69
MLI	2006	0.67	0.95	0.71
MLI	2007	0.67	0.91	0.74
MLI	2008	0.67	0.99	0.68
MLI	2009	0.66	0.89	0.74
MLI	2010	0.65	0.92	0.71
MLI	2011	0.64	0.88	0.73
MLI	2012	0.64	0.90	0.72
MLI	2013	0.65	0.76	0.85
MLI	2014	0.65	0.81	0.80
MLI	2015	0.65	0.90	0.72
MRT	1980	0.20	NA	NA
MRT	1981	0.20	0.18	1.12
MRT	1982	0.21	NA	NA
MRT	1983	0.21	NA	NA
MRT	1984	0.21	NA	NA
MRT	1985	0.22	NA	NA
MRT	1986	0.22	0.11	2.00
MRT	1987	0.23	0.10	2.28
MRT	1988	0.24	0.24	1.00
MRT	1989	0.25	0.28	0.91
MRT	1990	0.27	0.33	0.81
MRT	1991	0.28	0.29	0.98
MRT	1992	0.30	0.39	0.77

MRT	1993	0.32	0.44	0.72
MRT	1994	0.33	0.50	0.66
MRT	1995	0.34	0.50	0.69
MRT	1996	0.36	0.50	0.71
MRT	1997	0.37	0.28	1.31
MRT	1998	0.38	NA	NA
MRT	1999	0.41	0.26	1.57
MRT	2000	0.44	0.31	1.41
MRT	2001	0.47	0.61	0.77
MRT	2002	0.51	0.83	0.61
MRT	2003	0.54	0.76	0.71
MRT	2004	0.56	0.70	0.80
MRT	2005	0.59	0.71	0.83
MRT	2006	0.61	0.68	0.90
MRT	2007	0.64	0.75	0.85
MRT	2008	0.66	0.74	0.89
MRT	2009	0.68	0.64	1.06
MRT	2010	0.70	0.64	1.09
MRT	2011	0.72	0.75	0.96
MRT	2012	0.74	0.80	0.92
MRT	2013	0.75	0.80	0.94
MRT	2014	0.76	0.84	0.91
MRT	2015	0.77	0.73	1.05
NER	1980	0.16	NA	NA
NER	1981	0.16	0.06	2.62
NER	1982	0.16	NA	NA
NER	1983	0.17	NA	NA
NER	1984	0.17	NA	NA
NER	1985	0.18	NA	NA
NER	1986	0.19	0.05	3.78
NER	1987	0.20	NA	NA
NER	1988	0.22	0.09	2.39
NER	1989	0.23	0.13	1.79
NER	1990	0.25	0.35	0.72
NER	1991	0.27	0.39	0.70
NER	1992	0.29	0.37	0.77
NER	1993	0.29	0.38	0.77
NER	1994	0.30	0.39	0.76
NER	1995	0.30	0.42	0.70
NER	1996	0.29	0.32	0.90
NER	1997	0.28	0.33	0.85
NER	1998	0.27	0.25	1.10
NER	1999	0.28	0.24	1.15
NER	2000	0.29	0.25	1.14
NER	2001	0.30	0.31	0.97
NER	2002	0.32	0.21	1.54
NER	2003	0.36	0.52	0.68
NER	2004	0.40	0.62	0.64
NER	2005	0.45	0.89	0.51
NER	2006	0.51	0.99	0.51

NER	2007	0.55	0.78	0.71
NER	2008	0.60	0.89	0.68
NER	2009	0.65	0.93	0.69
NER	2010	0.68	0.92	0.74
NER	2011	0.72	0.97	0.74
NER	2012	0.74	0.96	0.77
NER	2013	0.76	0.92	0.83
NER	2014	0.77	0.93	0.83
NER	2015	0.78	0.90	0.87
NGA	1980	0.22	NA	NA
NGA	1981	0.23	NA	NA
NGA	1982	0.24	NA	NA
NGA	1983	0.25	NA	NA
NGA	1984	0.26	0.05	5.13
NGA	1985	0.27	0.09	2.98
NGA	1986	0.28	0.16	1.77
NGA	1987	0.29	0.21	1.40
NGA	1988	0.30	0.36	0.85
NGA	1989	0.31	0.47	0.66
NGA	1990	0.31	0.56	0.56
NGA	1991	0.31	0.39	0.80
NGA	1992	0.31	0.43	0.72
NGA	1993	0.30	0.29	1.05
NGA	1994	0.30	0.44	0.68
NGA	1995	0.29	0.34	0.84
NGA	1996	0.28	0.26	1.06
NGA	1997	0.26	0.21	1.26
NGA	1998	0.26	0.21	1.24
NGA	1999	0.26	NA	NA
NGA	2000	0.27	0.38	0.70
NGA	2001	0.27	NA	NA
NGA	2002	0.28	NA	NA
NGA	2003	0.29	NA	NA
NGA	2004	0.30	0.38	0.78
NGA	2005	0.31	0.38	0.81
NGA	2006	0.32	0.72	0.44
NGA	2007	0.33	0.69	0.48
NGA	2008	0.35	0.57	0.61
NGA	2009	0.36	0.71	0.51
NGA	2010	0.37	0.74	0.50
NGA	2011	0.38	0.61	0.62
NGA	2012	0.39	0.57	0.68
NGA	2013	0.40	0.65	0.62
NGA	2014	0.42	0.70	0.60
NGA	2015	0.43	0.74	0.58
PAN	1980	0.56	0.70	0.81
PAN	1981	0.58	0.72	0.80
PAN	1982	0.59	0.60	0.99
PAN	1983	0.61	0.85	0.72
PAN	1984	0.63	0.66	0.96

PAN	1985	0.66	0.73	0.90
PAN	1986	0.68	0.71	0.96
PAN	1987	0.71	0.73	0.97
PAN	1988	0.73	0.71	1.03
PAN	1989	0.76	0.70	1.08
PAN	1990	0.78	0.86	0.91
PAN	1991	0.80	0.82	0.98
PAN	1992	0.82	0.76	1.08
PAN	1993	0.84	0.82	1.02
PAN	1994	0.85	0.83	1.02
PAN	1995	0.85	0.86	0.99
PAN	1996	0.86	0.92	0.93
PAN	1997	0.86	0.95	0.91
PAN	1998	0.86	0.98	0.88
PAN	1999	0.85	0.92	0.92
PAN	2000	0.84	0.98	0.85
PAN	2001	0.82	0.99	0.83
PAN	2002	0.80	0.99	0.81
PAN	2003	0.78	0.98	0.80
PAN	2004	0.76	0.99	0.77
PAN	2005	0.74	0.88	0.84
PAN	2006	0.73	0.99	0.74
PAN	2007	0.72	0.85	0.84
PAN	2008	0.71	0.86	0.82
PAN	2009	0.71	0.85	0.83
PAN	2010	0.71	0.94	0.75
PAN	2011	0.71	0.87	0.82
PAN	2012	0.71	0.85	0.84
PAN	2013	0.72	0.80	0.90
PAN	2014	0.73	0.80	0.92
PAN	2015	0.75	0.73	1.03
PER	1980	0.31	0.16	1.95
PER	1981	0.34	0.20	1.70
PER	1982	0.37	0.23	1.62
PER	1983	0.41	0.23	1.78
PER	1984	0.45	0.28	1.61
PER	1985	0.49	0.48	1.03
PER	1986	0.54	0.50	1.08
PER	1987	0.58	0.43	1.35
PER	1988	0.62	0.61	1.02
PER	1989	0.66	0.58	1.13
PER	1990	0.69	0.72	0.96
PER	1991	0.72	0.71	1.01
PER	1992	0.75	0.83	0.90
PER	1993	0.78	0.87	0.89
PER	1994	0.80	0.87	0.92
PER	1995	0.82	0.95	0.86
PER	1996	0.84	0.99	0.85
PER	1997	0.85	0.98	0.87
PER	1998	0.85	0.98	0.87

PER	1999	0.84	0.99	0.85
PER	2000	0.83	0.98	0.85
PER	2001	0.81	0.90	0.90
PER	2002	0.80	0.95	0.84
PER	2003	0.79	0.94	0.84
PER	2004	0.78	0.91	0.85
PER	2005	0.77	0.89	0.86
PER	2006	0.77	0.94	0.82
PER	2007	0.77	0.99	0.78
PER	2008	0.79	0.99	0.79
PER	2009	0.80	0.92	0.87
PER	2010	0.82	0.93	0.88
PER	2011	0.84	0.91	0.92
PER	2012	0.85	0.95	0.90
PER	2013	0.87	0.88	0.98
PER	2014	0.88	0.88	1.00
PER	2015	0.89	0.90	0.99
PRY	1980	0.50	0.17	2.95
PRY	1981	0.52	0.28	1.86
PRY	1982	0.54	0.34	1.59
PRY	1983	0.56	0.44	1.27
PRY	1984	0.58	0.67	0.87
PRY	1985	0.60	0.57	1.05
PRY	1986	0.62	0.53	1.17
PRY	1987	0.64	0.58	1.10
PRY	1988	0.66	0.56	1.17
PRY	1989	0.68	0.61	1.11
PRY	1990	0.71	0.80	0.88
PRY	1991	0.73	0.80	0.91
PRY	1992	0.76	0.87	0.87
PRY	1993	0.78	0.78	1.00
PRY	1994	0.80	0.81	0.98
PRY	1995	0.82	0.80	1.02
PRY	1996	0.83	0.80	1.04
PRY	1997	0.84	0.82	1.03
PRY	1998	0.85	0.81	1.06
PRY	1999	0.86	0.77	1.12
PRY	2000	0.87	0.80	1.09
PRY	2001	0.88	0.89	0.98
PRY	2002	0.88	0.87	1.01
PRY	2003	0.89	0.86	1.03
PRY	2004	0.89	0.88	1.02
PRY	2005	0.90	0.87	1.03
PRY	2006	0.90	0.85	1.06
PRY	2007	0.91	0.78	1.16
PRY	2008	0.91	0.76	1.20
PRY	2009	0.92	0.72	1.27
PRY	2010	0.92	0.76	1.21
PRY	2011	0.92	0.76	1.21
PRY	2012	0.93	0.74	1.25

PRY	2013	0.93	0.73	1.27
PRY	2014	0.93	0.74	1.26
PRY	2015	0.94	0.80	1.17
RWA	1980	0.63	NA	NA
RWA	1981	0.64	0.17	3.79
RWA	1982	0.67	NA	NA
RWA	1983	0.69	0.59	1.17
RWA	1984	0.71	NA	NA
RWA	1985	0.73	0.50	1.47
RWA	1986	0.76	0.77	0.98
RWA	1987	0.77	0.79	0.98
RWA	1988	0.79	0.78	1.01
RWA	1989	0.80	0.68	1.18
RWA	1990	0.81	0.57	1.42
RWA	1991	0.82	0.89	0.92
RWA	1992	0.82	0.85	0.97
RWA	1993	0.83	0.83	0.99
RWA	1994	0.82	NA	NA
RWA	1995	0.82	0.90	0.92
RWA	1996	0.82	0.98	0.84
RWA	1997	0.82	0.77	1.06
RWA	1998	0.82	NA	NA
RWA	1999	0.82	0.85	0.97
RWA	2000	0.83	0.90	0.92
RWA	2001	0.84	0.77	1.09
RWA	2002	0.86	0.88	0.98
RWA	2003	0.88	0.96	0.91
RWA	2004	0.89	0.89	1.00
RWA	2005	0.91	0.95	0.96
RWA	2006	0.93	0.99	0.93
RWA	2007	0.94	0.97	0.97
RWA	2008	0.95	0.97	0.98
RWA	2009	0.96	0.97	0.99
RWA	2010	0.96	0.97	0.99
RWA	2011	0.97	0.97	1.00
RWA	2012	0.97	0.99	0.98
RWA	2013	0.98	0.99	0.99
RWA	2014	0.98	0.98	1.00
RWA	2015	0.98	0.99	0.99
SDN	1980	0.05	0.01	5.38
SDN	1981	0.06	0.01	5.80
SDN	1982	0.06	0.02	3.24
SDN	1983	0.07	0.03	2.46
SDN	1984	0.09	0.04	2.15
SDN	1985	0.10	0.08	1.27
SDN	1986	0.12	0.14	0.87
SDN	1987	0.15	0.27	0.54
SDN	1988	0.17	0.40	0.43
SDN	1989	0.21	0.40	0.51
SDN	1990	0.24	0.62	0.39

SDN	1991	0.28	0.63	0.44
SDN	1992	0.31	0.53	0.59
SDN	1993	0.35	0.51	0.69
SDN	1994	0.39	0.69	0.56
SDN	1995	0.42	0.72	0.59
SDN	1996	0.45	0.79	0.57
SDN	1997	0.46	0.78	0.59
SDN	1998	0.47	0.70	0.67
SDN	1999	0.47	0.79	0.60
SDN	2000	0.47	0.65	0.73
SDN	2001	0.48	0.71	0.67
SDN	2002	0.48	0.64	0.75
SDN	2003	0.49	0.74	0.67
SDN	2004	0.51	0.79	0.64
SDN	2005	0.52	0.83	0.63
SDN	2006	0.54	0.85	0.64
SDN	2007	0.57	0.91	0.62
SDN	2008	0.59	0.93	0.63
SDN	2009	0.61	0.91	0.67
SDN	2010	0.63	0.95	0.67
SDN	2011	0.66	0.93	0.70
SDN	2012	0.67	0.92	0.73
SDN	2013	0.69	0.93	0.75
SDN	2014	0.71	0.94	0.76
SDN	2015	0.72	0.93	0.78
SEN	1980	0.38	NA	NA
SEN	1981	0.38	NA	NA
SEN	1982	0.39	NA	NA
SEN	1983	0.40	NA	NA
SEN	1984	0.42	NA	NA
SEN	1985	0.45	0.54	0.83
SEN	1986	0.48	NA	NA
SEN	1987	0.51	NA	NA
SEN	1988	0.54	0.59	0.91
SEN	1989	0.56	0.71	0.79
SEN	1990	0.58	0.66	0.88
SEN	1991	0.59	0.51	1.16
SEN	1992	0.60	0.46	1.30
SEN	1993	0.61	0.52	1.17
SEN	1994	0.62	0.57	1.08
SEN	1995	0.63	0.80	0.79
SEN	1996	0.64	0.70	0.91
SEN	1997	0.64	0.59	1.09
SEN	1998	0.65	0.59	1.11
SEN	1999	0.67	0.60	1.11
SEN	2000	0.69	0.52	1.32
SEN	2001	0.71	0.52	1.37
SEN	2002	0.74	0.60	1.23
SEN	2003	0.76	0.73	1.04
SEN	2004	0.78	0.87	0.89

SEN	2005	0.79	0.84	0.95
SEN	2006	0.81	0.89	0.91
SEN	2007	0.82	0.94	0.87
SEN	2008	0.83	0.88	0.94
SEN	2009	0.84	0.86	0.97
SEN	2010	0.84	0.70	1.20
SEN	2011	0.85	0.83	1.03
SEN	2012	0.86	0.83	1.03
SEN	2013	0.86	0.92	0.94
SEN	2014	0.87	0.89	0.98
SEN	2015	0.87	0.89	0.98
SLE	1980	0.42	0.13	3.23
SLE	1981	0.44	0.15	2.90
SLE	1982	0.46	0.10	4.56
SLE	1983	0.48	0.24	2.00
SLE	1984	0.51	0.21	2.42
SLE	1985	0.54	NA	NA
SLE	1986	0.56	NA	NA
SLE	1987	0.59	NA	NA
SLE	1988	0.61	0.22	2.75
SLE	1989	0.62	NA	NA
SLE	1990	0.64	0.83	0.77
SLE	1991	0.63	0.56	1.13
SLE	1992	0.62	0.64	0.97
SLE	1993	0.60	0.63	0.96
SLE	1994	0.58	0.41	1.41
SLE	1995	0.55	0.43	1.27
SLE	1996	0.52	0.65	0.79
SLE	1997	0.49	0.26	1.87
SLE	1998	0.47	0.56	0.84
SLE	1999	0.47	NA	NA
SLE	2000	0.47	0.24	1.97
SLE	2001	0.49	0.38	1.29
SLE	2002	0.51	0.52	0.99
SLE	2003	0.54	0.70	0.77
SLE	2004	0.57	0.61	0.94
SLE	2005	0.61	0.64	0.95
SLE	2006	0.64	0.95	0.67
SLE	2007	0.67	0.79	0.85
SLE	2008	0.71	0.87	0.82
SLE	2009	0.75	0.91	0.82
SLE	2010	0.78	0.76	1.02
SLE	2011	0.80	0.76	1.05
SLE	2012	0.82	0.91	0.90
SLE	2013	0.84	0.92	0.91
SLE	2014	0.85	0.83	1.02
SLE	2015	0.85	0.86	0.99
SOM	1980	0.08	0.09	0.90
SOM	1981	0.09	0.02	4.31
SOM	1982	0.10	0.04	2.39

SOM	1983	0.11	0.05	2.18
SOM	1984	0.13	0.10	1.26
SOM	1985	0.15	0.22	0.66
SOM	1986	0.17	0.17	0.97
SOM	1987	0.18	0.25	0.74
SOM	1988	0.20	0.26	0.77
SOM	1989	0.21	0.18	1.16
SOM	1990	0.22	NA	NA
SOM	1991	0.22	NA	NA
SOM	1992	0.22	NA	NA
SOM	1993	0.22	NA	NA
SOM	1994	0.22	NA	NA
SOM	1995	0.21	NA	NA
SOM	1996	0.20	NA	NA
SOM	1997	0.19	0.19	1.02
SOM	1998	0.19	0.24	0.78
SOM	1999	0.18	0.18	1.01
SOM	2000	0.18	0.33	0.55
SOM	2001	0.19	0.33	0.57
SOM	2002	0.19	0.40	0.49
SOM	2003	0.21	0.40	0.52
SOM	2004	0.23	0.30	0.75
SOM	2005	0.25	0.35	0.72
SOM	2006	0.29	0.26	1.12
SOM	2007	0.34	0.40	0.84
SOM	2008	0.38	0.42	0.91
SOM	2009	0.42	0.51	0.83
SOM	2010	0.46	0.64	0.71
SOM	2011	0.47	0.60	0.79
SOM	2012	0.48	0.61	0.79
SOM	2013	0.48	0.34	1.41
SOM	2014	0.47	0.65	0.73
SOM	2015	0.47	0.44	1.07
SSD	1980	0.31	NA	NA
SSD	1981	0.31	NA	NA
SSD	1982	0.31	NA	NA
SSD	1983	0.32	NA	NA
SSD	1984	0.32	NA	NA
SSD	1985	0.32	NA	NA
SSD	1986	0.33	NA	NA
SSD	1987	0.33	NA	NA
SSD	1988	0.33	NA	NA
SSD	1989	0.34	NA	NA
SSD	1990	0.34	NA	NA
SSD	1991	0.34	NA	NA
SSD	1992	0.35	NA	NA
SSD	1993	0.35	NA	NA
SSD	1994	0.35	NA	NA
SSD	1995	0.35	NA	NA
SSD	1996	0.35	NA	NA

SSD	1997	0.35	NA	NA
SSD	1998	0.34	NA	NA
SSD	1999	0.32	NA	NA
SSD	2000	0.29	NA	NA
SSD	2001	0.26	NA	NA
SSD	2002	0.25	NA	NA
SSD	2003	0.23	NA	NA
SSD	2004	0.22	NA	NA
SSD	2005	0.22	NA	NA
SSD	2006	0.21	NA	NA
SSD	2007	0.21	0.30	0.70
SSD	2008	0.21	0.30	0.71
SSD	2009	0.22	0.50	0.44
SSD	2010	0.24	0.43	0.55
SSD	2011	0.25	0.46	0.53
SSD	2012	0.25	0.68	0.37
SSD	2013	0.26	0.56	0.46
SSD	2014	0.26	0.58	0.45
SSD	2015	0.27	0.50	0.53
SUR	1980	0.54	0.25	2.18
SUR	1981	0.57	0.27	2.11
SUR	1982	0.60	0.61	0.98
SUR	1983	0.63	0.85	0.74
SUR	1984	0.65	0.80	0.82
SUR	1985	0.68	0.84	0.80
SUR	1986	0.70	0.80	0.87
SUR	1987	0.71	0.71	1.01
SUR	1988	0.73	0.64	1.14
SUR	1989	0.75	0.72	1.04
SUR	1990	0.77	0.83	0.93
SUR	1991	0.78	0.75	1.05
SUR	1992	0.80	0.74	1.08
SUR	1993	0.81	0.76	1.07
SUR	1994	0.83	0.74	1.12
SUR	1995	0.84	0.84	1.00
SUR	1996	0.85	0.85	1.00
SUR	1997	0.85	0.81	1.06
SUR	1998	0.86	0.90	0.95
SUR	1999	0.86	0.85	1.01
SUR	2000	0.85	0.71	1.20
SUR	2001	0.85	0.68	1.25
SUR	2002	0.84	0.73	1.16
SUR	2003	0.83	0.75	1.11
SUR	2004	0.82	0.85	0.97
SUR	2005	0.80	0.83	0.97
SUR	2006	0.78	0.84	0.93
SUR	2007	0.76	0.84	0.91
SUR	2008	0.75	0.85	0.88
SUR	2009	0.75	0.87	0.86
SUR	2010	0.75	0.96	0.78

SUR	2011	0.75	0.86	0.87
SUR	2012	0.75	0.84	0.90
SUR	2013	0.76	0.87	0.88
SUR	2014	0.77	0.85	0.91
SUR	2015	0.79	0.89	0.88
TCD	1980	0.15	NA	NA
TCD	1981	0.15	NA	NA
TCD	1982	0.15	NA	NA
TCD	1983	0.15	0.01	14.69
TCD	1984	0.15	NA	NA
TCD	1985	0.16	NA	NA
TCD	1986	0.17	0.10	1.66
TCD	1987	0.17	0.14	1.23
TCD	1988	0.18	0.12	1.49
TCD	1989	0.19	0.47	0.39
TCD	1990	0.19	0.20	0.94
TCD	1991	0.19	0.18	1.06
TCD	1992	0.19	0.10	1.91
TCD	1993	0.19	0.13	1.49
TCD	1994	0.20	0.18	1.09
TCD	1995	0.20	0.18	1.11
TCD	1996	0.20	0.20	1.01
TCD	1997	0.21	0.24	0.86
TCD	1998	0.21	0.23	0.91
TCD	1999	0.21	0.33	0.64
TCD	2000	0.21	0.28	0.75
TCD	2001	0.21	0.27	0.78
TCD	2002	0.21	0.40	0.53
TCD	2003	0.21	0.47	0.45
TCD	2004	0.21	0.50	0.42
TCD	2005	0.21	0.58	0.37
TCD	2006	0.22	0.77	0.28
TCD	2007	0.22	0.70	0.32
TCD	2008	0.23	0.43	0.55
TCD	2009	0.25	0.75	0.33
TCD	2010	0.27	0.83	0.32
TCD	2011	0.28	0.70	0.41
TCD	2012	0.30	0.72	0.42
TCD	2013	0.31	0.80	0.39
TCD	2014	0.33	0.83	0.39
TCD	2015	0.34	0.92	0.37
TGO	1980	0.52	NA	NA
TGO	1981	0.53	NA	NA
TGO	1982	0.53	NA	NA
TGO	1983	0.54	NA	NA
TGO	1984	0.55	NA	NA
TGO	1985	0.55	NA	NA
TGO	1986	0.56	NA	NA
TGO	1987	0.57	0.54	1.05
TGO	1988	0.57	0.66	0.87

TGO	1989	0.58	0.65	0.89
TGO	1990	0.58	0.77	0.75
TGO	1991	0.57	0.82	0.70
TGO	1992	0.56	0.76	0.74
TGO	1993	0.55	0.75	0.73
TGO	1994	0.53	0.71	0.75
TGO	1995	0.51	0.47	1.09
TGO	1996	0.50	0.27	1.85
TGO	1997	0.50	0.40	1.24
TGO	1998	0.51	0.36	1.40
TGO	1999	0.53	0.48	1.10
TGO	2000	0.55	0.50	1.10
TGO	2001	0.57	0.43	1.33
TGO	2002	0.60	0.59	1.01
TGO	2003	0.62	0.72	0.86
TGO	2004	0.65	0.71	0.91
TGO	2005	0.67	0.82	0.82
TGO	2006	0.69	0.87	0.80
TGO	2007	0.72	0.88	0.81
TGO	2008	0.74	0.89	0.83
TGO	2009	0.76	0.89	0.85
TGO	2010	0.78	0.92	0.85
TGO	2011	0.80	0.92	0.86
TGO	2012	0.81	0.84	0.96
TGO	2013	0.82	0.84	0.98
TGO	2014	0.83	0.87	0.95
TGO	2015	0.84	0.88	0.95
TTO	1980	0.72	0.26	2.77
TTO	1981	0.74	0.62	1.19
TTO	1982	0.76	0.54	1.40
TTO	1983	0.77	0.60	1.28
TTO	1984	0.78	0.65	1.19
TTO	1985	0.78	0.75	1.04
TTO	1986	0.77	0.70	1.10
TTO	1987	0.76	0.79	0.97
TTO	1988	0.75	0.82	0.92
TTO	1989	0.74	0.77	0.96
TTO	1990	0.73	0.82	0.89
TTO	1991	0.72	0.82	0.87
TTO	1992	0.70	0.87	0.81
TTO	1993	0.69	0.81	0.85
TTO	1994	0.68	0.85	0.80
TTO	1995	0.67	0.89	0.75
TTO	1996	0.66	0.89	0.74
TTO	1997	0.66	0.90	0.73
TTO	1998	0.67	0.91	0.73
TTO	1999	0.68	0.90	0.75
TTO	2000	0.69	0.90	0.77
TTO	2001	0.71	0.91	0.78
TTO	2002	0.72	0.96	0.75

TTO	2003	0.74	0.91	0.81
TTO	2004	0.75	0.94	0.80
TTO	2005	0.77	0.95	0.81
TTO	2006	0.78	0.92	0.85
TTO	2007	0.79	0.88	0.90
TTO	2008	0.80	0.90	0.89
TTO	2009	0.82	0.90	0.91
TTO	2010	0.83	0.90	0.92
TTO	2011	0.84	0.90	0.93
TTO	2012	0.85	0.92	0.92
TTO	2013	0.86	0.92	0.93
TTO	2014	0.87	0.92	0.94
TTO	2015	0.88	0.96	0.91
TZA	1980	0.60	0.59	1.02
TZA	1981	0.61	0.55	1.11
TZA	1982	0.62	0.50	1.24
TZA	1983	0.64	0.52	1.23
TZA	1984	0.66	0.58	1.13
TZA	1985	0.68	0.67	1.01
TZA	1986	0.70	0.74	0.94
TZA	1987	0.72	0.79	0.91
TZA	1988	0.74	0.85	0.87
TZA	1989	0.76	0.77	0.99
TZA	1990	0.78	0.78	1.00
TZA	1991	0.80	0.81	0.98
TZA	1992	0.81	0.83	0.98
TZA	1993	0.82	0.83	0.99
TZA	1994	0.83	0.84	0.98
TZA	1995	0.83	0.81	1.02
TZA	1996	0.83	0.82	1.01
TZA	1997	0.83	0.79	1.05
TZA	1998	0.84	0.79	1.06
TZA	1999	0.84	0.76	1.11
TZA	2000	0.85	0.79	1.08
TZA	2001	0.86	0.87	0.99
TZA	2002	0.87	0.89	0.98
TZA	2003	0.88	0.95	0.93
TZA	2004	0.89	0.95	0.94
TZA	2005	0.89	0.90	0.99
TZA	2006	0.89	0.90	0.99
TZA	2007	0.90	0.83	1.08
TZA	2008	0.90	0.86	1.05
TZA	2009	0.91	0.85	1.07
TZA	2010	0.92	0.91	1.01
TZA	2011	0.92	0.92	1.00
TZA	2012	0.93	0.92	1.01
TZA	2013	0.94	0.91	1.03
TZA	2014	0.94	0.97	0.97
TZA	2015	0.94	0.98	0.96
UGA	1980	0.51	NA	NA

UGA	1981	0.52	0.09	5.73
UGA	1982	0.53	NA	NA
UGA	1983	0.54	0.14	3.82
UGA	1984	0.55	NA	NA
UGA	1985	0.55	0.14	3.95
UGA	1986	0.56	0.21	2.67
UGA	1987	0.57	0.39	1.46
UGA	1988	0.58	0.40	1.45
UGA	1989	0.59	0.60	0.98
UGA	1990	0.60	0.77	0.78
UGA	1991	0.60	0.77	0.78
UGA	1992	0.61	0.71	0.86
UGA	1993	0.61	0.73	0.84
UGA	1994	0.62	0.79	0.79
UGA	1995	0.64	0.74	0.86
UGA	1996	0.66	0.72	0.91
UGA	1997	0.68	0.61	1.11
UGA	1998	0.71	0.56	1.28
UGA	1999	0.76	0.60	1.26
UGA	2000	0.79	0.58	1.37
UGA	2001	0.81	0.61	1.34
UGA	2002	0.82	0.72	1.14
UGA	2003	0.82	0.81	1.02
UGA	2004	0.81	0.87	0.94
UGA	2005	0.81	0.84	0.96
UGA	2006	0.80	0.80	1.00
UGA	2007	0.79	0.85	0.93
UGA	2008	0.79	0.79	1.00
UGA	2009	0.79	0.83	0.95
UGA	2010	0.80	0.80	1.00
UGA	2011	0.81	0.82	0.99
UGA	2012	0.83	0.78	1.06
UGA	2013	0.84	0.97	0.87
UGA	2014	0.85	0.99	0.86
UGA	2015	0.86	0.89	0.97
VEN	1980	0.52	0.56	0.93
VEN	1981	0.52	0.54	0.96
VEN	1982	0.52	0.53	0.98
VEN	1983	0.52	0.58	0.89
VEN	1984	0.52	0.33	1.58
VEN	1985	0.53	0.49	1.08
VEN	1986	0.54	0.58	0.94
VEN	1987	0.56	0.57	0.98
VEN	1988	0.57	0.56	1.02
VEN	1989	0.59	0.57	1.03
VEN	1990	0.60	0.63	0.95
VEN	1991	0.61	0.64	0.95
VEN	1992	0.62	0.67	0.92
VEN	1993	0.62	0.67	0.93
VEN	1994	0.62	0.63	0.98

VEN	1995	0.62	0.68	0.91
VEN	1996	0.61	0.57	1.08
VEN	1997	0.62	0.60	1.03
VEN	1998	0.62	0.39	1.60
VEN	1999	0.64	0.76	0.85
VEN	2000	0.66	0.77	0.86
VEN	2001	0.68	0.70	0.97
VEN	2002	0.69	0.65	1.07
VEN	2003	0.71	0.68	1.04
VEN	2004	0.72	0.86	0.84
VEN	2005	0.72	0.87	0.83
VEN	2006	0.73	0.71	1.02
VEN	2007	0.73	0.62	1.18
VEN	2008	0.74	0.50	1.47
VEN	2009	0.75	0.84	0.89
VEN	2010	0.76	0.78	0.97
VEN	2011	0.77	0.78	0.99
VEN	2012	0.78	0.81	0.96
VEN	2013	0.79	0.82	0.96
VEN	2014	0.80	0.78	1.02
VEN	2015	0.81	0.87	0.93
ZMB	1980	0.60	NA	NA
ZMB	1981	0.61	NA	NA
ZMB	1982	0.63	NA	NA
ZMB	1983	0.65	0.83	0.78
ZMB	1984	0.67	0.58	1.15
ZMB	1985	0.69	NA	NA
ZMB	1986	0.71	0.66	1.08
ZMB	1987	0.73	0.65	1.13
ZMB	1988	0.75	0.63	1.19
ZMB	1989	0.77	0.58	1.32
ZMB	1990	0.78	0.71	1.10
ZMB	1991	0.79	0.91	0.87
ZMB	1992	0.80	0.61	1.31
ZMB	1993	0.80	0.67	1.20
ZMB	1994	0.80	0.86	0.93
ZMB	1995	0.79	0.82	0.97
ZMB	1996	0.79	0.83	0.95
ZMB	1997	0.78	0.70	1.12
ZMB	1998	0.79	NA	NA
ZMB	1999	0.79	0.92	0.86
ZMB	2000	0.79	0.76	1.04
ZMB	2001	0.79	NA	NA
ZMB	2002	0.79	0.88	0.90
ZMB	2003	0.80	0.91	0.88
ZMB	2004	0.81	0.94	0.86
ZMB	2005	0.81	0.91	0.89
ZMB	2006	0.82	0.97	0.85
ZMB	2007	0.83	0.92	0.90
ZMB	2008	0.83	0.95	0.88

ZMB	2009	0.84	0.98	0.86
ZMB	2010	0.85	0.84	1.01
ZMB	2011	0.85	0.81	1.05
ZMB	2012	0.86	0.78	1.10
ZMB	2013	0.86	0.79	1.09
ZMB	2014	0.87	0.86	1.01
ZMB	2015	0.88	0.90	0.97
